# A European randomised controlled trial of the addition of etoposide to standard vincristine and carboplatin induction as part of an 18-month treatment programme for childhood (≤16 years) low grade glioma – A final report

**DOI:** 10.1016/j.ejca.2017.04.019

**Published:** 2017-08

**Authors:** Astrid K. Gnekow, David A. Walker, Daniela Kandels, Susan Picton, Giorgio Perilongo, Jacques Grill, Tore Stokland, Per Eric Sandstrom, Monika Warmuth-Metz, Torsten Pietsch, Felice Giangaspero, René Schmidt, Andreas Faldum, Denise Kilmartin, Angela De Paoli, Gian Luca De Salvo, of the Low Grade Glioma Consortium and the participating centers, Astrid K. Gnekow, Irene Slavc, Giorgio Perilongo, Sue Picton, David Walker, Tore Stokland, Per Erik Sandstrom, Niels Clausen, Mikko Arola, Olafur Gisli Jonsson, Ofelia Cruz, Aurora Navajas, Anna Teijeiro, Jacques Grill, Chantal Kalifa, Marie-Anne Raquin, Joris Verlooy, Volkmar Hans, Torsten Pietsch, Wolfram Scheurlen, Johannes Hainfellner, Felice Giangaspero, James Ironside, Keith Robson, Kari Skullerud, David Scheie, NN, Marie-Madeleine Ruchoux, Anne Jouvet, Dominique Figarella-Branger, Arielle Lellouch-Toubiana, Monika Warmuth-Metz, Daniela Prayer, Milena Calderone, Tim Jaspan, Soren Jacob Bakke, Eli Vazquez, Dominique Couanet, Rolf D. Kortmann, Karin Diekmann, Giovanni Scarzello, Roger Taylor, Knut Lote, Jordi Giralt, Christian Carrie, Jean Louis Habrand, Niels Soerensen, Thomas Czech, Paul Chumas, Bengt Gustavson, Michel Zerah, Bettina Wabbels, Maria Luisa Pinello, Alistair Fielder, Ian Simmons, Terje Christoffersen, Gabriele Calaminus, Knut Brockmann, Ronald Straeter, Friedrich Ebinger, Pablo Hernaiz-Driever, Herwig Lackner, Colin Kennedy, Adam Glaser, Bo Stromberg, Jose Ma Indiano, Chantal Rodary, Eric Bouffet, Didier Frappaz, Andreas Faldum, Angela Emser, Gian Luca De Salvo, Suzanne Stephens, David Machin, Marie-Cécile Le Deley, Thore Egeland, Carolyn Freemann, Martin Schrappe, Richard Sposto

**Affiliations:** bu Pediatric Oncology, GPOH, Germany; n Pediatric Oncology, GPOH, Austria; o Pediatric Oncology, AIEOP, Italy; p Pediatric Oncology, UK; q Pediatric Oncology, NOPHO, Sweden; r Pediatric Oncology, SEOP, Spain; s Pediatric Oncology, SFCE, France; t Pediatric Oncology, BSPHO, Belgium; u Pathology & Molecular Biology, GPOH, Germany; v Pathology & Molecular Biology, GPOH, Austria; w Pathology & Molecular Biology, AIEOP, Italy; x Pathology & Molecular Biology, UK; y Pathology & Molecular Biology, NOPHO, Sweden; z Pathology & Molecular Biology, SEOP, Spain; aa Pathology & Molecular Biology, SFCE, France; ab Radiology, GPOH, Germany; ac Radiology, GPOH, Austria; ad Radiology, AIEOP, Italy; ae Radiology, UK; af Radiology, NOPHO, Sweden; ag Radiology, SEOP, Spain; ah Radiology, SFCE, France; ai Radiotherapy, GPOH, Germany; aj Radiotherapy, GPOH, Austria; ak Radiotherapy, AIEOP, Italy; al Radiotherapy, UK; am Radiotherapy, NOPHO, Sweden; an Radiotherapy, SEOP, Spain; ao Radiotherapy, SFCE, France; ap Neurosurgery, GPOH, Germany; aq Neurosurgery, GPOH, Austria; ar Neurosurgery, AIEOP, Italy; as Neurosurgery, UK; at Neurosurgery, NOPHO, Sweden; au Neurosurgery, SEOP, Spain; av Neurosurgery, SFCE, France; aw Ophthamology, GPOH, Germany; ax Ophthamology, GPOH, Austria; ay Ophthamology, AIEOP, Italy; az Ophthamology, UK; ba Ophthamology, NOPHO, Sweden; bb Ophthamology, SEOP, Spain; bc Ophthamology, SFCE, France; bd Neuropediatrics, Health Status/Quality of Life, GPOH, Germany; be Neuropediatrics, Health Status/Quality of Life, GPOH, Austria; bf Neuropediatrics, Health Status/Quality of Life, AIEOP, Italy; bg Neuropediatrics, Health Status/Quality of Life, UK; bh Neuropediatrics, Health Status/Quality of Life, NOPHO, Sweden; bi Neuropediatrics, Health Status/Quality of Life, SEOP, Spain; bj Neuropediatrics, Health Status/Quality of Life, SFCE, France; bk Associated Research Phase II – Studies, Toronto, Canada; bl Associated Research Phase II – Studies, Lyon, France; bm Biostatistics, GPOH, Germany; bn Biostatistics, AIEOP, Italy; bo Biostatistics, UK; bp Biostatistics, SFCE, France; bq Biostatistics, NOPHO, Sweden; br Data Monitoring & Safety Committee, Montreal, Canada; bs Data Monitoring & Safety Committee, Kiel, Germany; bt Data Monitoring & Safety Committee, Arcadia, CA, USA; a Swabian Children's Cancer Center, Klinikum Augsburg, Germany; b Children's Brain Tumour Research Centre, University of Nottingham, UK; c Leeds Children's Hospital, UK; d Division of Pediatrics, University Hospital of Padua, Italy; e Institut Gustave Roussy, Paris, France; f University Hospital of North Norway, Norway; g Umea University, Sweden; h Department of Neuro-radiology, University of Wurzberg, Germany; i University of Bonn Medical Centre, Germany; j Department of Radiological, Oncological and Anatomo-pathological Sciences, University Sapienza Rome, Rome, Italy; k IRCCS Neuromed, Pozzilli, Italy; l Institute of Biostatistics and Clinical Research, University of Muenster, Germany; m Clinical Trials and Biostatistics Unit, IRCCS Istituto Oncologico Veneto, Padova, Italy

**Keywords:** Low grade glioma, Childhood, Chemotherapy, Randomised trial

## Abstract

**Background:**

The use of chemotherapy to manage newly diagnosed low grade glioma (LGG) was first introduced in the 1980s. One randomised trial has studied two- versus four-drug regimens with a duration of 12 months of treatment after resection.

**Methods:**

Within the European comprehensive treatment strategy for childhood LGG, the International Society of Paediatric Oncology–Low Grade Glioma (SIOP LGG) Committee launched a randomised trial involving 118 institutions and 11 countries to investigate the addition of etoposide (100 mg/m^2^, days 1, 2 & 3) to a four-course induction of vincristine (1.5 mg/m^2^ × 10 wkly) and carboplatin (550 mg/m^2^ q 3 weekly) as part of 18-month continuing treatment programme. Patients were recruited after imaging diagnosis, resection or biopsy with progressive disease/symptoms. Some 497 newly diagnosed patients (M/F 231/266; median age 4.26 years (interquartile range (IQR) 2.02–7.06)) were randomised to receive vincristine carboplatin (VC) (n = 249) or VC plus etoposide (VCE) during induction (n = 248), stratified by age and tumour site.

**Findings:**

No differences between the two arms were found in term of survival and radiological response. Response and non-progression rates at 24 weeks for VC and VCE, were 46% versus 41%, and 93% versus 91% respectively; 5-year Progression-Free Survival (PFS) and Overall Survival (OS) were 46% (StDev 3.5) versus 45% (StDev 3.5) and 89% (StDev 2.1) versus 89% (StDev 2.1) respectively. Age and diencephalic syndrome are adverse clinical risk factors for PFS and OS. 5-year OS for patients in early progression at week 24 were 46% (StDev 13.8) and 49% (StDev 16.5) in the two arms, respectively.

**Interpretation:**

The addition of etoposide to VC did not improve PFS or OS. High non-progression rates at 24 weeks justify retaining VC as standard first-line therapy. Infants with diencephalic syndrome and early progression need new treatments to be tested. Future trials should use neurological/visual and toxicity outcomes and be designed to discriminate between the impact on disease outcomes of ‘duration of therapy’ and ‘age at stopping therapy’.

## Highlights

• A randomised trial in the commonest brain tumour type in children – low grade glioma.
• Tumour response assessment at 6 months identifies those at greatest risk of subsequent progression and death.
• Intensifying induction treatment with etoposide does not improve progression-free survival.
• International consortium successfully recruits from 11 European countries in a childhood brain tumour.
• Carboplatin hypersensitivity is reduced by coadministration of etoposide.

## 1 Introduction

Childhood Low Grade Gliomas (LGGs) arise during brain growth, developing in all areas of the central nervous system (CNS). LGGs are predominantly of grade 1 histology and are generally not anticipated to undergo malignant transformation during childhood. Resectability varies with location; hypothalamic-chiasmatic tumours in particular are generally considered unresectable, they present when the children are very young and can threaten vision, endocrine function and require non-surgical therapy [1], [2].

The SIOP Brain Tumour LGG Subcommittee had developed a comprehensive, multimodality treatment strategy for Childhood LGG, the International Society of Paediatric Oncology–Low Grade Glioma (SIOP LGG) committee, which was piloted and widely accepted clinically [3], [4]. Patients with progressive disease post-surgery or disease threatening neurological function were considered for non-surgical treatment.

Radiotherapy (RT) had previously been the mainstay of treatment for incompletely resected LGG, but RT does not confer a survival benefit or advantage in delaying first progression [5], [6]. Historically, the introduction of chemotherapy in this disease group was aimed at delaying or obviating the need for radiotherapy to minimise cognitive, endocrine and vascular consequences [7], [8], [9]. A variety of agents, given singularly and in combinations, had been tried with a variety of indications in institution-based trials, producing comparable results [1]. High initial tumour non-progression rates (approximately 70–90%) could be achieved, but 5-year PFS rates declined after stopping treatments, especially in children diagnosed who were less than 1 year of age [10].

The first SIOP LGG study was a multinational (UK, Germany & Italy) single arm pilot study, launched to test selection criteria for non-surgical treatments in newly diagnosed patients with LGG. Non-surgical therapy was stratified by age, those <5 years of age were offered VC and those >5 years of age were recommended for radiotherapy.

The 12-month chemotherapy regimen of the first SIOP LGG study comprised VC with an intensified 3-month induction and a 9-month, reduced-intensity continuing phase [3], [4], [11]. An analysis of risk factors confirmed the improved outcome for patients with neurofibromatosis type 1 (NF1)-associated visual pathway glioma (NF1-VPG), whereas NF1-negative patients had a higher risk for progression after stopping chemotherapy.

In launching this trial, we intended to investigate drug intensification in the non-NF1 group by adding etoposide to the standard VC combination, driven by reports of multiagent, Société Française d'Oncologie Pédiatrique (SFOP) regimens [12] and the ongoing Children's Oncology Group (COG 9952) randomised trial comparing two drugs (VC) versus four drugs (thioguanine, procarbazine, CCNU (lomustine), vincristine [TPCV] regimen) [13]. In selecting etoposide we hoped to harness its synergy with the platinum-derived agent [14], [15], [16], [17], [18], [19], [20] and restrict its cumulative dose to control the risk of secondary acute myeloid leukaemia (AML). Treatment duration was extended to 18 months, on the working hypothesis that childhood LGG is a chronic disease process, driven by normal growth across childhood and therefore requiring therapy extended over significant developmental periods. This is the first report of the results of this randomised trial of chemotherapy intensification in childhood LGG.

## 2 Study strategy and methods

### 2.1 Study strategy

The International Society of Paediatric Oncology–Low Grade Glioma subcommittee (SIOP-LGG) 2004 study was a multinational protocol for previously untreated patients aged up to 16 years with the histological diagnosis of a LGG according to the World Health Organisation (WHO) classifications of 2000 and 2007. The radiological diagnosis of an LGG was accepted for visual pathway glioma (VPG) in non-NF1 patients with extensive visual pathway involvement and additional hypodensity of the tumour on a native computed tomography (CT)-scan.

**Fig. 1:**
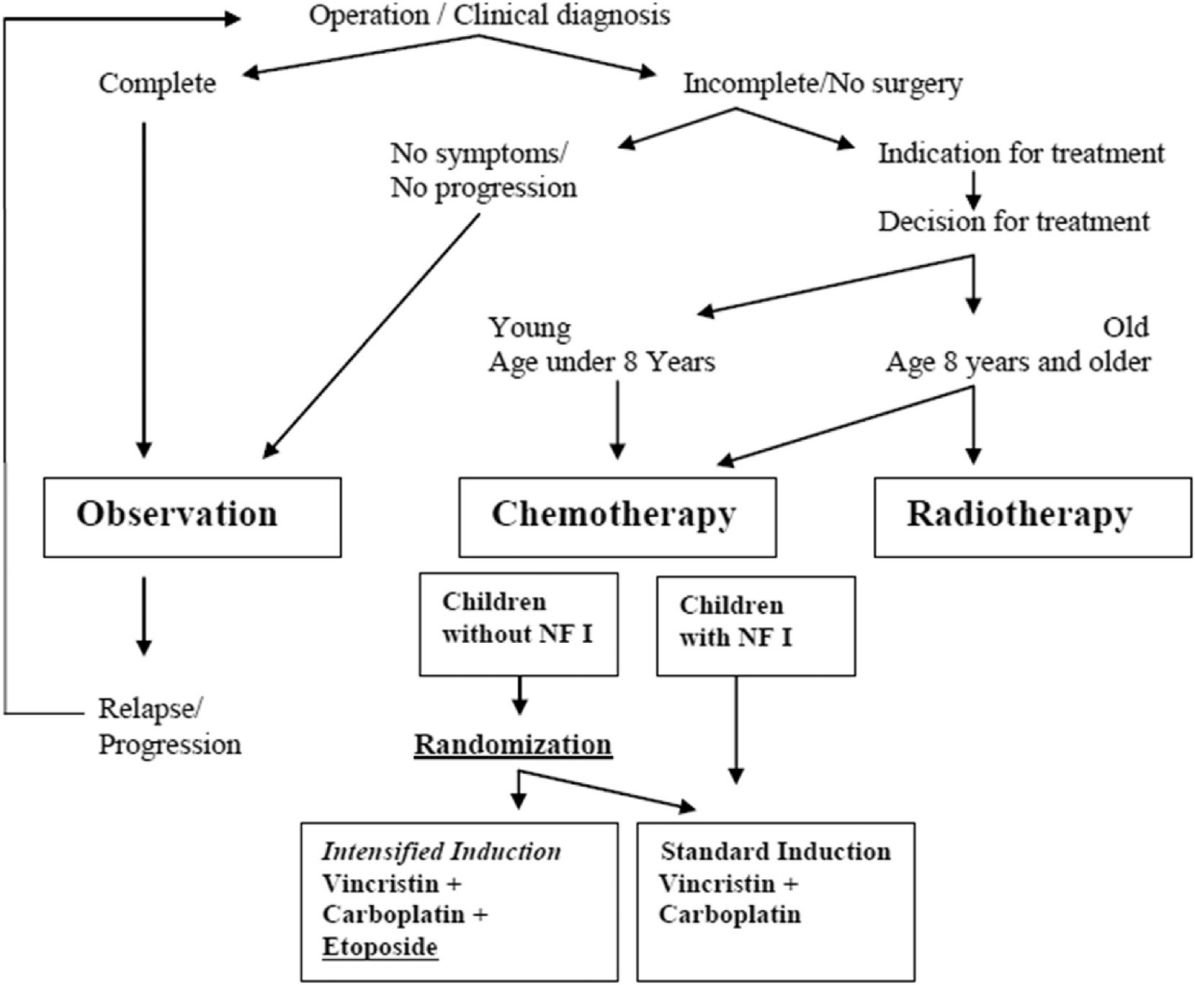
Flow diagram of the study.

At diagnosis, best safe resection of the primary tumour was recommended. Observation was scheduled for all children who were without threatening clinical or ophthalmological symptoms, while non-surgical therapy was indicated at radiological progression following diagnosis or at evidence of threatening symptoms [3], [4]. Thus, patients were entered into the study at diagnosis or after a period of observation (see Fig. 1).

Based on the concern of the side-effects of radiotherapy at a younger age, the age split for chemo- versus radiotherapy was chosen at 8 years within the SIOP-LGG treatment strategy in 2004. Therefore, at the time the study was launched, primary chemotherapy was recommended for all children aged less than 8 years where there was either progressive or symptomatic disease or threat to neurological functions such as loss of vision. Older children could be included to receive chemotherapy by local physician's choice.

Within the chemotherapy arm of the study, non-NF1 patients with an LGG irrespective of histological subtype or location, excluding isolated optic nerve tumours, were eligible for the prospectively randomised trial to receive either ‘standard’ VC or the ‘intensified regimen plus etoposide’ (VCE) during the induction period. Eligibility and exclusion criteria are detailed in Table 1. Randomisation was stratified for age (<1, 1–8, and ≥8 years) and tumour location (pure chiasmatic/Dodge II, chiasmatic-hypothalamic/Dodge III plus other supratentorial midline structures, and tumours outside the supratentorial midline). Randomisation was performed by a centralised, interactive internet-based system that, after a summary check of patient's eligibility, generated the random allocation using a randomised blocked design, accounting for age and primary tumour site.

**Table 1:** Eligibility and exclusion criteria for the randomised part of the SIOP-LGG 2004 trial.

**Eligibility:** • Age: children and adolescents up to the completion of the 16th year of life. • Histology: Low grade glioma according to ICD-O Code ○ Children with chiasmatic-hypothalamic tumours may be eligible without histological diagnosis, if neuroradiologic findings meet unequivocal criteria for the presence of a low grade glioma. • Primary tumour localisation intracranial and/or spinal cord. • Disseminated low grade glioma • Primary tumour diagnosis without pretreatment with chemotherapy or radiotherapy • Informed consent given by the patient and/or his legal guardian (parents)
**Exclusion** • Associated genetic conditions like neurofibromatosis NF I or tuberous sclerosis • Primary diffuse intrinsic tumours of the pons, even if histologically astrocytoma I/II • Low grade, but non-glial, rare intracranial neoplasms • Pretreatment with chemo- or radiotherapy (except for steroids) • Preexisting impairments of health status, making the conduct of the study impossible or ethically unwise. • Evidence of pregnancy or lactation period

**Randomization:** All eligible patients without Neurofibromatosis NF I (receiving chemotherapy as their first non-surgical therapy) were eligible for randomisation.

**Participation in another clinical study:** In case the patient participated in another clinical study simultaneously to being enrolled in the study SIOP-LGG 2004, which was not interfering with the present treatment strategy (e.g. endocrinologic study), this should be known to the national study chairmen.

**Medication:** Concomitant medication for associated or other conditions (e.g. hormone replacement, anticonvulsants), not containing cytostatic drugs, should be recorded, but was no exclusion criteria.

Standard induction consisted of ten weekly doses of vincristine (VCR) 1.5 mg/m^2^ intravenous (i.v.) bolus and four ‘single’ doses of carboplatin (CBDCA) 550 mg/m^2^ as 1-h i.v. infusion at 3-week intervals followed by three cycles of simultaneous VC at 4-week intervals. Etoposide was added for intensification with 100 mg/m^2^ as 1-h i.v. infusion on day 1–3 in weeks 1, 4, 7 and 10. For consolidation, patients of both arms received ten 6-week cycles of vincristine 1.5 mg/m^2^ i.v. day 1, 8 and 15 and carboplatin 550 mg/m^2^ i.v. day 1. Total chemotherapy treatment lasted 18 months, 6 months longer than the pilot regimen from SIOP LGG [3], [4].

Dose modification was advised for children <10 kg of weight to carboplatin 18.3 mg/kg and vincristine 0.05 mg/kg. Further dose reductions of one-third were recommended for children <6 months of age. Dose reductions were prescribed in case of haematological or organ toxicities. Hypersensitivity reaction to carboplatin, where Common Toxicity Criteria (CTC) was grade I (rash/mild fever), permitted the repeated administration under close surveillance, premedication and slowed infusion rate. Following CTC-grade ≥II reactions, replacement of carboplatin was recommended with cycles of cis-platinum (30 mg/m^2^/3 h, day 1 and 2) and cyclophosphamide (1500 mg/m^2^/1 h, day 1).

The protocol offered detailed guidance for the requirements to start subsequent courses of chemotherapy and supportive care. Concomitant medication for associated or other conditions, not containing cytostatic drugs, was permitted.

Radiological and clinical/ophthalmological response assessment was scheduled at week 24, and repeated at weeks 54, 85 and regularly thereafter.

Radiological response assessment followed accepted criteria and was based on a 3-dimensional measurement of the tumour size on the magnetic resonance imaging (MRI) sequence on which the outline of the tumour was best delineated. Volume calculation followed an approximation to the formula of the rotational ellipsoid ((a × b × c)/2). Changes in tumour size were given in % of the reference volume and categorised in progressive disease (PD, >25% increase in volume or new lesion), stable disease (SD, volume changes <25%), improvement (IMP, 25–50% volume reduction), partial (PR, >50% volume reduction) and complete response (disappearance of all lesions). For the individual comparison the respective sequence had to be the same at baseline and follow-up, and if the best sequence changed also measurements had to be repeated. Contrast enhancement had no influence on staging. In non-measurable tumours for whatever reason (very irregular or poor discrimination from surrounding normal brain) volume changes were estimated, and changes were interpreted with caution. The central review process evolved during the trial with technical advances in transmitting scans. Where it was carried out, the reviewers noted that the treatment centres underestimated response, making it unlikely that the response rates are an overestimate.

Assessment of ophthalmological function was detailed in the protocol and response was graded as ‘better, same, worse’, as no internationally consented response criteria had been available.

### 2.2 Statistical considerations

#### 2.2.1 Material and methods

All trial patients who were randomised to be treated with chemotherapy between 1st April 2004, and 14th April 2014 and followed-up until 31st December 2014 are included. Patient data were reviewed and verified between the national trial centres and the international data centre. Reference pathological review was organised nationally, complemented by three international panel meetings. Reference radiological review was also organised nationally. Central radiological and histological review was recommended, but was not a prerequisite for this analysis.

The data bank was provided by CINECA (Casalecchio, Italy). The Clinical Trial Unit of the Istituto Oncologico Veneto, Padua, Italy, where data management and descriptive analyses were performed, served as International Data Centre. Survival analyses, including the main question of the trial, were performed by the Institute of Biostatistics and Clinical Research, Muenster, Germany, which served as Statistical Trial Centre. All statistical analyses were done on an intention-to-treat basis and all protocol violations were analysed with their randomised groups. A sensitivity analysis for the confirmatory main question of the trial was performed excluding two patients for whom reference pathology revealed that eligibility criteria were not fulfilled (one grade III astrocytoma after central review and one case of Alexander disease). Continuous variables were summarised as mean, standard deviation, quartiles, minimum, and maximum values, and categorical variables were reported as counts and percentages. Radiological assessment of tumour response by MRI followed recommended criteria [21]. Clinical response was defined as clinical + imaging non-progression. Considered positive were complete response (CR), partial response (PR), objective response (OR) and stable disease (SD). To verify associations between response rate and treatment randomisation arms, the chi-squared test was computed.

This study is registered with European Union Clinical Trials Register No. 2005-005377-29.

#### 2.2.2 Compliance with clinical strategy

The clinical strategy selecting patients for treatment or observation had been extensively piloted [3], [4]. Timing of commencement of treatment after surgery, an observation decision and randomisation was recorded. Dose intensity compliance for chemotherapy drugs with chemotherapy schedule was estimated proportionally, as was compliance with drug hypersensitivity recommendations.

#### 2.2.3 Survival analysis

Overall Survival (OS) was calculated from date of randomisation until death of any cause or last contact for patients alive. Progression-Free Survival (PFS) was calculated from date of randomisation until an event, defined as progression of residual tumour, relapse following previous complete resection, appearance of new or progression of existing metastasis, death of any cause, or last contact for patients without event. To evaluate the variable response at week 24, PFS (OS) was calculated from date of response assessment until event (death) or until last follow-up, if no event occurred. The distribution of PFS and OS within each randomisation arm was evaluated according to the Kaplan–Meier method [22].

Cox regression models within the complete randomised cohort were used to analyse the prognostic and predictive values of clinical and biologic variables on OS and PFS (See: Supplementary Methods).

Multivariable model building (See: Supplementary Methods) included following variables: randomisation arm, age at randomisation (categorical and continuous with transformations: linear, square, cubic, logarithm), gender, indication for treatment, extent of resection, metastases status, tumour histology, primary tumour site, localisation strata at randomisation, interval from diagnosis to start of chemotherapy. To evaluate response at week 24, further Cox regression models were built with PFS/OS calculated from date of response assessment while additionally including the variables response status at week 24 and interval from randomisation to response assessment.

Given for the final models are estimated hazard ratios of the selected explanatory variables with their respective 95% confidence intervals and likelihood ratio test p-values.

### 2.3 Main question of the SIOP-LGG 2004 trial

The following confirmatory null hypothesis was tested: The PFS of children on intensified induction does not differ from the PFS of children on standard induction. This primary aim was analysed by a two-sided log-rank test on difference on a significance level of 5% according to the intention-to-treat principle using a 3-step adaptive design based on the inverse normal method [23]. The bounds of the adaptive design result from a 3-step group sequential plan according to Pampallona and Tsiatis [24] with futility stop, with Δ = 0, two-sided significance level 5%, power = 90% for hazard ratio (standard versus intensified) 1.609, equidistantly spaced information rates for the analyses, and equal allocation ratio to treatment arms. The original group sequential design was advanced to an adaptive design pursuant to a biostatistical amendment (See: Supplementary Methods).

Besides the main question of the trial, all analyses were regarded as exploratory and p values are given descriptively to detect and study meaningful effects.

The analyses were carried out using SAS statistical package (SAS, rel. 9.2; SAS Institute, Inc., Cary, NC) and SPSS (version 23, SPSS Inc., Chicago, United States of America).

## 3 Results

### 3.1 Patient cohort

**Fig. 2:**
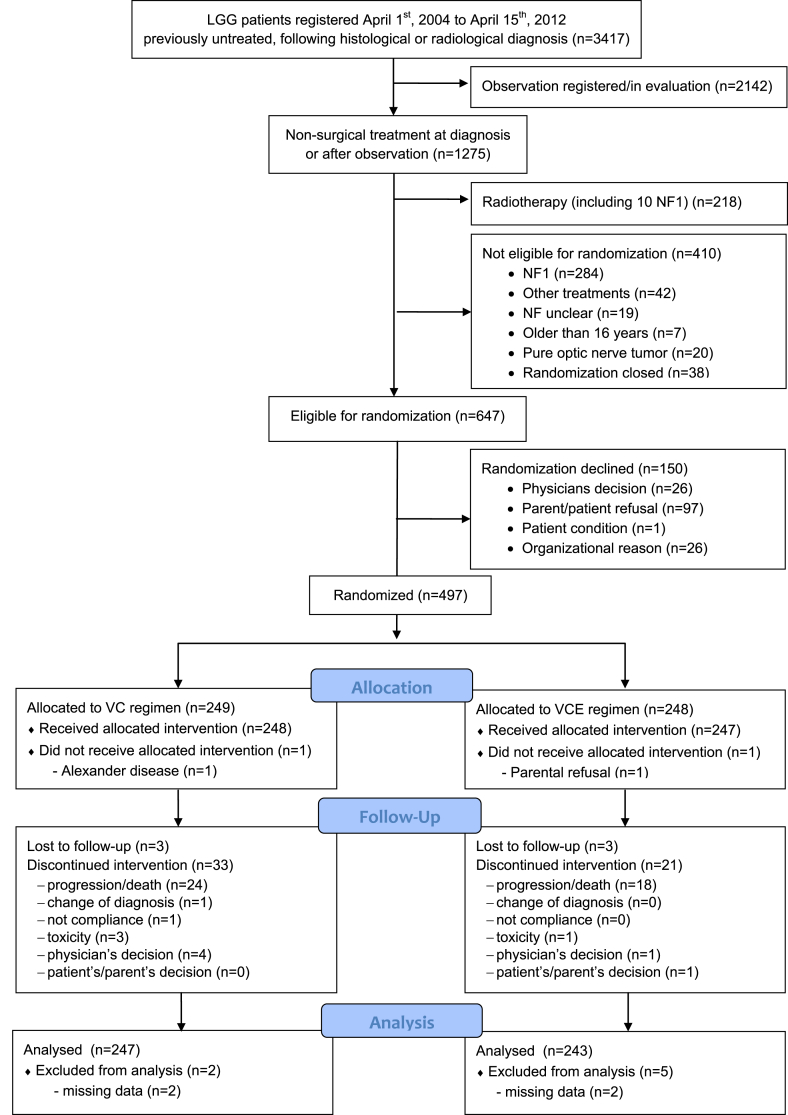
CONSORT diagram.

Between 1st April 2004 and 14th April 2012, 3417 previously untreated patients from 118 institutions in 11 countries were registered at the SIOP-LGG 2004 database following the SIOP-LGG treatment strategy. During the trial period, 1057 patients received chemotherapy. Of these, 497 non-NF1 patients were randomised to receive either VC- (n = 249) or VCE-induction (n = 248) (Fig. 2).

The demographic and clinical characteristics of the two treatment groups of patients are summarised in Table 2. They were balanced for age at diagnosis and tumour location. Mean age was 4.3 (StDev 3.3) years at diagnosis and 5.0 (StDev 3.7) years at randomisation. At start of treatment, 14.7% were younger than 1 year, 66.0% with age ≥1 and <8 years, and 19.3% between 8 and 16 years. The sex ratio showed a slight male preponderance (1.15:1). Diencephalic syndrome (DS) was defined as a secondary failure to thrive with proper caloric intake, and no longitudinal growth impairment was reported in 59 patients [2], [25], [26].

**Table 2:** Patient characteristics.

Patients randomised	All (n = 497)	VC (n = 249)	VCE (n = 248)
Gender (f/m)	231/266	118/131	113/135
Age at randomisation			
Mean	4.98	4.96	5.01
Median	4.26	4.42	4.13
Median range (q1–q3)	2.02–7.06	1.99–7.06	2.14–7.07
Age group – strata at randomisation			
<1 year	73 (14.7%)	38	35
≥1 and <8 years	328 (66.0%)	163	165
≥8 years	96 (19.3%)	48	48
Localisation – strata at randomisation			
Supratentorial midline			
Dodge II (chiasmatic)	47 (9.5%)	25	22
Dodge III and other locations	268 (53.9%)	133	135
All other locations	182 (36.6%)	91	91
Primary tumour site			
Cerebral hemispheres	32 (6.4%)	16	16
Supratentorial midline	315 (63.4%)	158	157
Dodge II	47	25	22
Dodge III	121	64	57
Others	147	69	78
Cerebellum	30 (6.0%)	16	14
Brainstem	80 (16.1%)	41	39
Spinal cord	40 (8.0%)	18	22
Extent of all surgeries prior to start of chemotherapy			
Complete resection	6 (1.2%)	4	2
Subtotal/near total	32 (6.4%)	19	13
Partial resection^a^	184 (37.0%)	85	99
Biopsy (open, stereotactic, endoscopic)^a^	195 (39.2%)	94	101
Not evaluable	12 (2.4%)	6	6
No surgery^b^	68 (13.7%)	41	27
Tumour histology/WHO grade			
Pilocytic astrocytoma I	289 (67.4%)	141	148
Pilomyxoid astrocytoma II	36 (8.4%)	17	19
Diffuse glioma II	49 (11.2%)	25	24
Glioneuronal tumours I	30 (7.0%)	15	15
All others (LGG nos, Astrocytoma nos, RGNT, other mixed glioma)	25 (5.8%)	10	15
Dissemination prior to treatment^c^ (M1/M2/M3/M4/not known)	69 (13.9%) (1/30/30/5/13)	27 (0/10/8/2/7)	42 (1/20/22/3/6)
Interval from diagnosis to start of chemotherapy			
≤3.0 months	299 (60.2%)	148	151
3–6 months	49 (9.9%)	28	21
6–12 months	54 (10.9%)	27	27
12–24 months	51 (10.2%)	24	27
>24 months	44 (8.9%)	22	22
Indication to treatment (multiple recordings/patient possible)			
Diencephalic syndrome	59	28	31
Severe/progressive neurologic symptoms	218	107	111
Severe/progressive visual impairment	183	85	98
Visual deterioration	119	55	64
Borderline vision	107	51	56
Nystagmus in infants	79	32	47
Loss of vision in second eye with first eye blind	22	12	10
Pressure effect of tumour mass	74	34	40
Symptomatic/progressive metastases	20	6	14
Radiological tumour progression	198	99	99
Radiological progression only	97	54	43
Radiological progression + symptoms	101	45	56

a The proportions of partial resection + biopsy are different between the VC- and VCE-arm, p 0.0218.

b There is no significant difference between the proportions without histological diagnosis, p 0.0704. Tumours were located in the VP 58, other supratentorial midline structures 7, cerebellum 2, and caudal brainstem 1.

c The proportions of dissemination between the VC- and VCE-arm are different of borderline significance p 0.0495. Primaries were located: 60.9% supratentorial midline, 13.0% brainstem, and 8.7% each for cerebrum, cerebellum, and spinal cord.

The majority of patients had partial resection or biopsy only (76.2%) prior to the start of chemotherapy. First surgery had been delayed for >3 months from diagnostic MRI in 19 patients. Within the interval between diagnosis and start of chemotherapy, 106 patients underwent up to four further tumour resections (1 n = 84, 2 n = 16, 3 n = 5, 4 n = 1). In the time period between diagnosis and randomisation tumour progression, with or without associated symptoms, occurred in six patients after initial total resection.

The majority of histologies were pilocytic astrocytoma WHO Grade I (67.4%). Histological diagnosis was local; additional central review was available for 54.5%. Sixty-eight patients (13.7%) did not have verification biopsy of the LGG, due to unequivocal neuroradiological appearances.

There was a noticeable difference in the proportion of patients with partial resection/biopsy (VCE > VC, p = 0.0218) and a minor difference in the proportion of patients without biopsy (VC > VCE, p = 0.0704).

Tumour dissemination at start of therapy was present in 69 patients with comparable distribution of primary tumour sites to the entire cohort. Disseminated lesions were equally distributed throughout the intracranial and spinal leptomeningeal space; extra-neural manifestation was reported in five patients, all associated with the presence of a ventriculoperitoneal shunt (VP-shunt).

Following stratification according to localisation and age, primary chemotherapy after diagnosis was started within 3 months for 60.2%, within 1 year for 80.9%, and after more than 1 year for only 19.1%. The majority of those with severe visual or neurological impairment, including diencephalic syndrome (DS), started within the first 3 months from the date of diagnosis.

### 3.2 Administration of treatment

Detailed treatment documentation was obtained for 242 patients receiving VC and 236 receiving VCE for a total of 1574 and 1562 administered induction cycles respectively (Table 3). Treatments administered and toxicity were comparable in both arms over time. Compliance with dose intensity targets for both vincristine and carboplatin were high (68–71.6%). The addition of etoposide did not alter this. Grade 4 haematological toxic events were more common in VCE-arm and were associated with more grade 3 infections. There was no difference in the actual duration of 21-week induction treatment period between the two arms.

**Table 3:** Treatment details for the induction phase and response at 24 weeks (intention-to-treat).

	VC (n = 249)		VCE (n = 248)	
Patients for analysis of treatment details	242		236	
Number of administered cycles	1574		1562	
Treatment duration to week 21 (median, range, in weeks)	22.3 (10.0–36.0)		21.8 (20.0–28.8)	
Actual mean dose intensity for patients who completed induction (mg/m^2^/week)	(n = 206)		(n = 214)	
Vincristine				
Target	0.88		0.88	
Actual (min–max)	0.6 (0.2–1.2)		0.6 (0.1–1.2)	
Carboplatin				
Target	175.0		175.0	
Actual (min–max)	124.2 (35.5–290.9)		125.3 (25.0–361.7)	
Etoposide				
Target	–		54.5	
Actual (min–max)			39.7 (4.1–112.7)	
Interruption of induction	33		21	
Reasons for interruption				
Progression	23		15	
Death	1		3	
Toxicity	3		1	
Non-compliance	6		2	
Change of diagnosis (delayed pathological report)	1		–	
Physician's decision	4		1	
Patient's decision	1		1	
**Worst grade of toxicity** (CTC-criteria)				
CTC-grade	3	4	3	4
Haematological	45	159	26	188
Infection	44	2	73	1
Renal	–	–	1	–
Auditory/hearing	–	–	2	–
Nausea/vomiting	23	–	36	1
Constitutional symptoms	18	5	27	6
Neurology motor	16	2	17	–
Neurology sensory	12	1	13	1
Gastrointestinal	7	2	12	1
Hepatic	12	–	10	–
**Carboplatin allergy**				
Patients with at least one allergic event	31		17	
Number of allergic events	41		22	
Treatment consequence per event				
None (treatment continued)	22		16	
Dose modified	4		1	
Change to protocol alternative	8		2	
Change after induction	1		–	
Other	6		3	
**Response assessment at week 24 post induction** (not included for radiological response: interruption for progression 29, death 4, no information available 43)				
Interval (start of treatment to assessment)				
Median (weeks, q1–q3)	24.4 (23.0–26.3)		24.1 (23.3–25.8)	
Mean (weeks, min–max)	25.0 (12.8–76.0)		24.6 (12.0–36.1)	
Standard deviation	4.89		2.72	
Radiological response	(n = 211)		(n = 210)	
CR	3		3	
PR	59		50	
IMP	36		33	
SD	98		106	
PD	15		18	
Ophthalmological response (vision) for patients with visual pathway tumour	(n = 78)		(n = 68)	
Better	13		11	
Stable	35		24	
Worse	6		7	
Not done/not applicable	24		26	
Neurological response	(n = 215)		(n = 216)	
Better	73		80	
Stable (existing unchanged)	89		88	
Worse (progression of existing or emergence of new symptoms)	9		15	
Not done/not applicable	44		33	

There were more interruptions to therapy in VC- compared to VCE-arm although proportions of interruptions due to tumour progression were similar, 69.7% versus 71% respectively. Four interruptions were due to deaths, 1 in VC-arm and 3 in VCE-arm; three were related to tumour progression, one a toxic death relating to an infant with severe DS who died within the 1st week of treatment from a Rotavirus sepsis that pre-dated commencing therapy. The protocol specified non-infectious status as a prerequisite for starting chemotherapy.

Hypersensitivity reactions occurred in patients in both arms, being less frequent in VCE (31/242 VC and 17/236 VCE). Most allergic events were managed according to protocol recommendations (82%), with the consequences listed in Table 3. During consolidation, the frequency of hypersensitivity events continued to be lower in the group in the VCE arm during induction (VC n = 117, VCE n = 44 patients). Mild hypersensitivity reactions were associated with no consequences, during induction in 53.7% (VC) and 72.2% (VCE), during consolidation in 35.1% (VC) and 33.8% (VCE). A switch of treatment to recommended alternatives occurred in 78/185 events in 117 patients (VC) and 24/71 events in 44 patients (VCE) during the consolidation phase. This difference in hypersensitivity rates between the two arms of the trial was an unexpected finding. The variation in alternative regimens used after discontinuation of carboplatin therapy precluded us from trying to analyse the impact of hypersensitivity on subsequent tumour responsiveness or toxicity.

### 3.3 Response assessment

Response assessment was scheduled at 24 weeks from the start of treatment and performed after a median time of 24.3 weeks for the evaluable patients on an intention-to-treat basis. Comparing VC (n = 211) and VCE (n = 210) using available scans, radiological response comprised volume reduction respectively, in 46% versus 41%, stable disease (SD) in 46% versus 51% and progressive disease (PD) in 7% versus 9% (Table 3).

The study was designed to use PFS as primary end-point. Clinical response/progression were allocated by the treating physician. We checked information about imaging and visual assessments. Compliance was variable across national groups. Central radiological review was performed for more than 50% of MRI controls throughout the treatment phase. The proportion of reviewed scans increased during the course of treatment, as technical solutions for sharing scans improved in national health systems in some countries.

Neurological improvement or stabilisation was reported for three quarters of patients (VC 75.3%, VCE 77.8%), but we lack information as to whether this can be attributed to treatment or to rehabilitation. An accurate assessment of visual function and specifically of visual acuity in response to treatment is available only in a limited number of patients. Overall, ophthalmologic response (predominantly visual acuity) for patients with VPG was reported better/stable in 48/78 VC- (61.5%) and 35/68 VCE-patients (51.5%), and deteriorated in 6/78 (7.7%) versus 7/68 (10.3%). A more detailed analysis of visual responses in national subgroup is in preparation for publication.

### 3.4 Outcome

After a median follow-up of 5.2 years, 430 children are alive with or without evidence of disease (Table 4). Fifty-four patients died (25 in the VC-arm, 29 in the VCE-arm), the majority died of disease (n = 43) or tumour-related complications (n = 9) including the toxic death after induction in a case < 1 year with diencephalic syndrome (DS) and Rotavirus sepsis. One death from second malignant neoplasm in the VCE-arm was reported (no details documented). Only 30 patients in each arm are alive without evidence of disease, while the majority are alive with tumour. During the trial period, 263 events (PD or death) were reported (133 VC versus 130 VCE): Progressive disease was judged by centre assessment by combining radiological, visual and neurological responses. By the beginning of consolidation 67 events had occurred (31 VC versus 36 VCE), 31 events occurred during consolidation treatment (16 VC versus 15 VCE) and 165 since the end of treatment (86 VC versus 79 VCE).

**Table 4:** Patient status.

	All (n = 497)	VC (n = 249)	VCE (n = 248)
Current status			
Alive, disease free	60	30	30
Alive, disease present, regression	18	8	10
Alive, disease present, stable	316	165	151
Alive, progression/relapse	36	16	20
Dead	54	25	29
No information/lost to follow up	6/7	2/3	4/4
Reasons for death			
Tumour progression	38	19	19
Metastases	5	2	3
Complications of tumour or therapy	9	4	5
Relation to tumour not clear	2	–	2
Radiotherapy (RT) for progression	59	33	26
Median age at start of RT (years, q1–q3)	8.0 (6.8–11.4)	7.8 (6.7–10.2)	8.7 (7.0–11.5)
Median time RT was delayed (months, q1–q3)	27.4 (8.3–51.8)	30.5 (11.9–58.4)	15.9 (6.9–51.5)

Details of radiotherapy were given for 59 patients. They had attained a median age of 8.0 years, radiation being delayed for median 2.3 years (range 0.7–4.3 years) from diagnosis. While 54 children were treated at the primary tumour site (41 external beam photons, 9 external beam, protons, 4 brachytherapy), 4 received craniospinal irradiation, 1 not known. During the time of the trial, it became clinical practice to use a variety of salvage treatments including additional drug therapy and repeat surgery before radiotherapy. Data describing these other treatments was not collected, so radiation-free survival cannot be calculated and given this change in practice is unlikely to be a parameter in future trials unless multiple strategies are defined at the outset.

### 3.5 Main question of the trial

After 66 events the first interim analysis was performed with the decision to continue the trial to stage two. After 132 events the second interim analysis was performed resulting in a futility stop (overall p = 0.28). Consequently, accrual to the trial was stopped on 15th April 2012 with non-rejection of the confirmatory null hypothesis. A sensitivity analysis showed that the decision on the futility stop does not depend on the two patients with eligibility criteria not fulfilled.

### 3.6 Analysis of survival and prognostic factors

Kaplan–Meier estimate for 5-year Overall Survival (OS) is 89.2% (StDev 2.1) in the VC- and 88.8% (StDev 2.1) in the VCE-arm, and 5-year PFS is 46.1% (StDev 3.5) versus 45.3% (StDev 3.5), respectively, with a median time to progression of 4.1 years. All data are summarised in Tables 5a–d. (See Supplementary Tables).

Univariable analyses for PFS by treatment-arm (Table 5a) show no differences between VC and VCE for the randomisation strata of tumour location but indicate differences for the randomisation strata for age: PFS is impaired for patients ≥8 years in the VCE-as compared to the VC-arm. Radiological tumour response at week 24, using Complete Response (CR)/Partial Response (PR)/Objective Response (OR) versus Stable Disease (SD) was not prognostic for PFS.

Following 18 months of chemotherapy, Progression-Free Survival (PFS) is poorer in both arms for age <1 year and dissemination at diagnosis, and when diencephalic syndrome was the indication for treatment. OS is poorer in both treatment arms for infants and for those with early progression at week 24. Histological subgroups differ for PFS and OS, but patients with pilocytic astrocytoma have identical OS rates in both arms. Differences observed between smaller subsets are inconsistent, as were those for extent of resection.

**Fig. 3:**
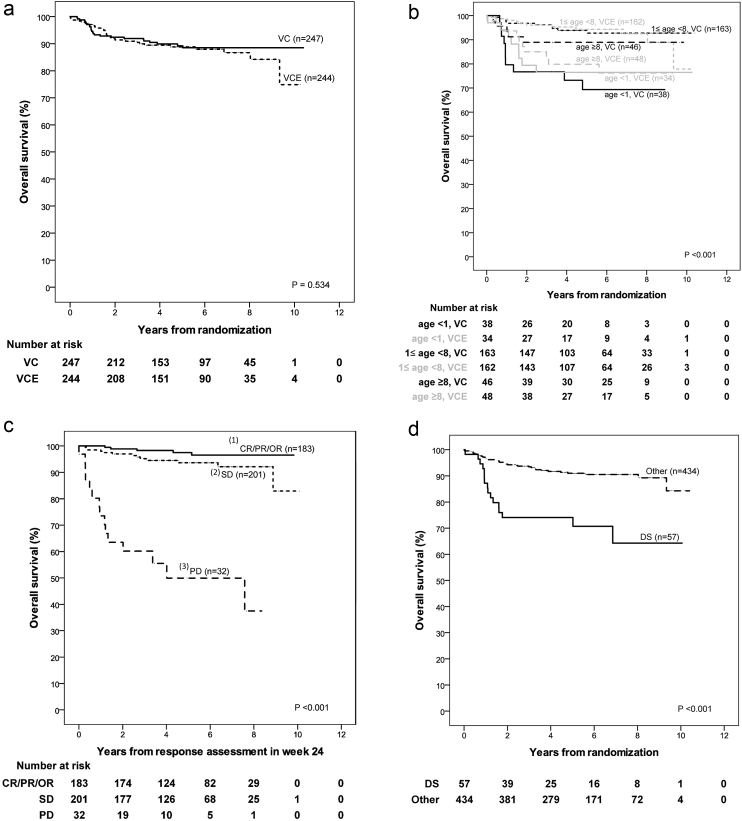
a: Kaplan–Meier estimates and p-value of log-rank test for overall survival stratified according to randomisation arm (VC, VCE). b: Kaplan–Meier estimates and p-value of log-rank test for overall survival stratified according to age group (strata at randomisation: <1 year, ≥1 and <8 years, ≥8 years) by randomisation arm (VC, VCE). c: Kaplan–Meier estimates and p-value of log-rank test for overall survival measured from response assessment in week 24 stratified according to response status at week 24: (1) Complete response (CR), partial response (PR) or objective response (OR), (2) stable disease (SD), (3) progressive disease (PD). d: Kaplan–Meier estimates and p-value of log-rank test for overall survival stratified according to indication to start treatment (diencephalic syndrome (DS), other).

Only the randomisation strata for age and the interval to start of treatment were found to be predictive for PFS (Table 5b and c). Taken together, these infer that minor differences between treatment arms for factors like extent of resection or histological subgroup are rather by chance (Fig. 3).

**Fig. 4:**
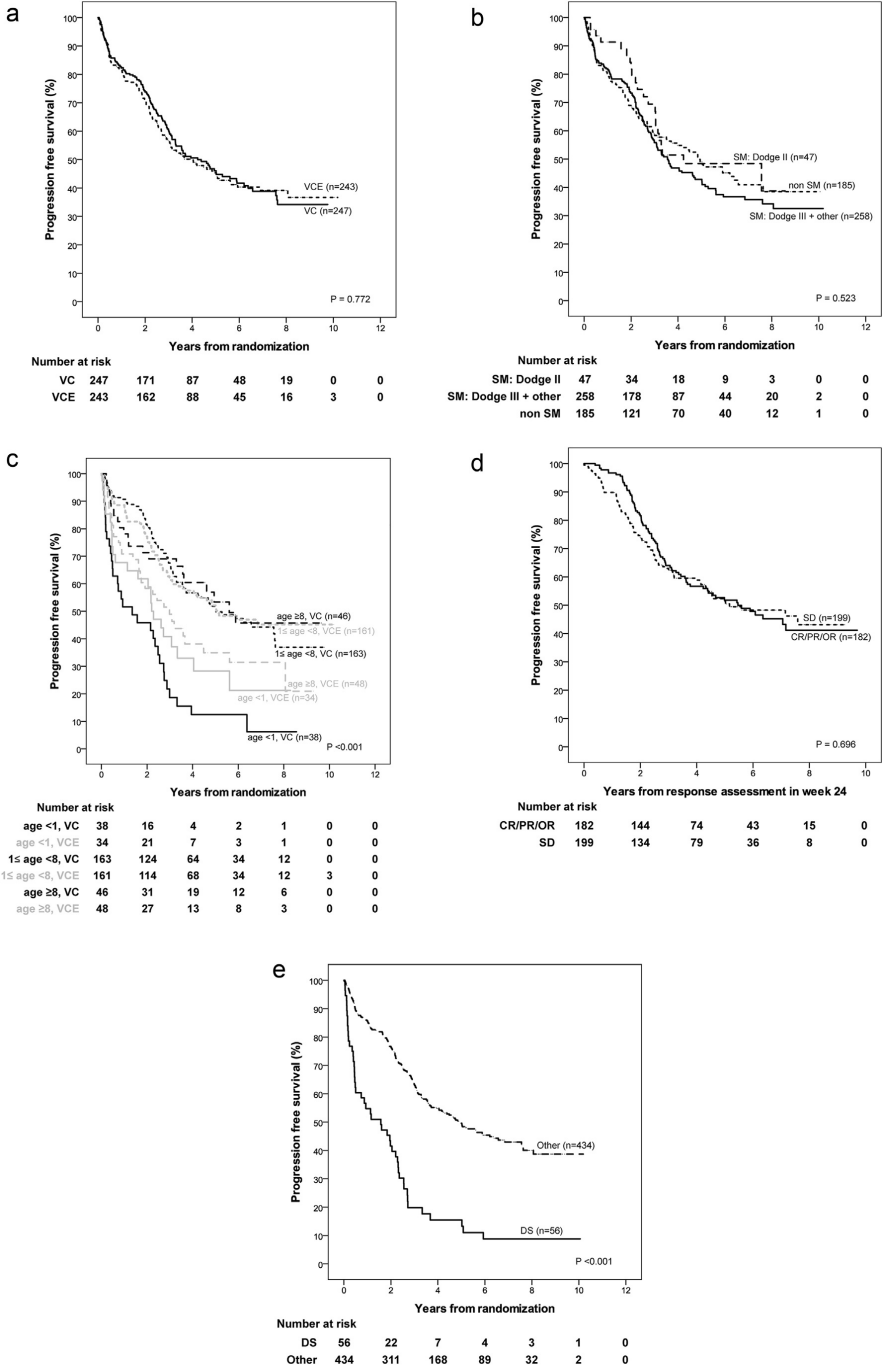
Full legend can be found on the next page. a: Kaplan–Meier estimates and p-value of log-rank test for progression-free survival stratified according to randomisation arm (VC, VCE). b: Kaplan–Meier estimates and p-value of log-rank test for progression-free survival stratified according to localisation strata at randomisation: (i) Pure chiasmatic/Dodge II, (ii) chiasmatic-hypothalamic/Dodge III plus other midline structures, (iii) tumours outside the supratentorial midline. SM: Supratentorial midline. c: Kaplan–Meier estimates and p-value of log-rank test for progression-free survival stratified according to age group (strata at randomisation: <1 year, ≥1 and <8 years, ≥8 years) by randomisation arm (VC, VCE). d: For all patients without event by the time of response assessment at week 24: Kaplan–Meier estimates and p-value of log-rank test for progression-free survival measured from response assessment in week 24 stratified according to response status at week 24: (i) Complete response (CR), partial response (PR) or objective response (OR), (ii) stable disease (SD). e: Kaplan–Meier estimates and p-value of log-rank test for progression-free survival stratified according to indication to start treatment (diencephalic syndrome (DS), other).

Multivariable analysis (Table 5d) confirms the presence of DS, mostly present at young age, as an unfavourable prognostic factor for PFS, relevant at date of randomisation and beyond response assessment at week 24. The interaction of age and treatment group may be seen from Fig. 4c in the observation that the Kaplan–Meier curves for VC and VCE in the age group 1–8 years overlap, whereas PFS in the age group >8 is considerably worse in the VCE group as compared to the VC group.

For OS, patient factors, including DS, age <1 year, >8 years at randomisation were identified as unfavourable. Diffuse gliomas grade 2 account for 11% of biopsied tumours; they are the second commonest histological subgroup after pilocytic astrocytoma. There was a trend towards worse PFS on univariable analysis but this trend was not identified as statistically noticeable in multivariable analysis. When assessing OS however diffuse glioma grade 2 histology did confer a statistically noticeable adverse influence on survival compared to pilocytic astrocytoma in multivariable analysis with a hazard ratio of 5.56 (95% CI: 2.52–12.23). Of all factors, early tumour progression at week 24 predicted for the most unfavourable OS with a hazard ratio of 16.96 (95% CI: 8.21–35.07).

## 4 Discussion

This is the first European randomised trial of chemotherapy in childhood LGG and only the second worldwide. It has recruited patients after diagnosis using standardised selection criteria for high risk or actual, progression. The proposed hypothesis, that the addition of a third drug to intensify treatment will improve response rates and PFS, is not supported.

### 4.1 Main question of the trial

The response rates in the two arms VC and VCE, respectively, were 46% versus 41% for volume reduction (CR, PR, OR), and non-progression rates were 93% versus 91% at 24 weeks after the start of treatment. The 5-year PFS and OS survival rates for VC and VCE arm were 46% and 45% (PFS) and 89% and 89% (OS).

Due to differences in eligibility criteria and treatment details, comparison of our results to the only other randomised trial in this disease (COG 9952) [13] is difficult. Also the time-point of evaluating patient and tumour status differed. Response rates in the COG trial were assessed at the end of one year's chemotherapy and excluded those who had dropped out during treatment. The non-progression rate in the COG 9952 at 1 year was 68% in both arms (CV: complete/partial 35%, minor 15%, stable disease 17%; TPCV: complete/partial 30%, minor 22%, stable disease 16%). Their 5-year Event Free Survival (EFS) was 39% for the CV- and 52% for the TPCV-arm, and 5-year OS was 86% for CV and 87% for TPCV. Their analysis identified significant sustained EFS advantage for the TPCV drug regimen, using a ‘cure model’. Their EFS results bracket our 5-year PFS results, whilst the OS results are similar.

For the CV arm in COG 9952 5-year EFS was 34%, while 5-year PFS in SIOP-LGG 2004 was 46% for the VC-arm. The two protocols differ with respect to length of treatment and dosing scheduling of the drugs. In the SIOP-LGG 2004, carboplatin (550 mg/m^2^) was given 3-weekly in induction, 4-weekly in intensification phase prior to week 24 and 6-weekly during continuation phase for 12 months. In COG study, carboplatin (175 mg/m^2^) was given weekly for induction over 10 weeks and during the continuation phase but at a reduced intensity. Another difference between the studies is the duration of therapy. Only a possible prospective randomised trial could solve the issue, if the difference in carboplatin scheduling and in the duration of therapy could have some impact on EFS or PFS. One of the reasons to prolong the 12-month vincristine–carboplatin therapy of the SIOP-group had been the convincing EFS of the original vincristine-carboplatin regimen, which lasted for 84 weeks [9], [27]. But the question of the ‘optimal’ duration of chemotherapy for LGG has never been addressed properly until this study.

The other significant difference between the two studies is the choice of drugs in this patient group who are very young and who have enhanced familial cancer risk implicated by their tumour diagnosis [28]. The COG 4 drug arm (TPCV) includes three drugs with known genotoxicity at significant cumulative doses (6 thioguanine, 640 mg/m^2^; procarbazine 1600 mg/m^2^; CCNU 880 mg/m^2^). The SIOP VCE arm specified etoposide at a ceiling cumulative dose of 1200 mg/m^2^ in order to limit exposure and the known risk of secondary AML. VC is considered of low genotoxicity although carboplatin is an alkylating agent. The histology of the one secondary malignancy in the VCE-arm in this study could not be retrieved. The higher 5-year EFS for TPCV in COG 9952 is of interest, however it did not translate to improved OS. It is our understanding the TPCV is not in routine use in USA despite the improved EFS rate. Concern about the risk of second malignancy may be a factor.

Definitions of response have been standardised internationally [21], and central review of radiologic response assessment was organised nationally in our trial. The impact of timing of response assessment has not been investigated previously. Our choice of week 24 as main time point for response assessment was driven by the concept of slow growing and thus slowly reacting tumours. In this trial's analysis, progression at week 24 proved to be the most important risk factor for death in the multivariable model, OS being only 46% and 49% for patients in PD at week 24 after both VC and VCE, with a hazard ratio of 16.96. This risk factor has not been reported previously.

The relevance of tumour response to chemotherapy for the functional status has to be considered in conjunction with the primary goal to defer radiotherapy. While there were some reports on the improvement of DS with chemotherapy [12], [25], and the ophthalmologic outcomes were better for responders in the French series [12], no trial had assessed visual or neurological outcome in detail [2], [29], [30]. Our trial reports clinical improvement or stabilisation for the majority of our patients following induction. The difficulties we encountered in assessing visual response, particularly in very young children, were linked to a lack of standardisation of visual and clinical measurements and weak clinical contact with specialists in measurement of visual function by trial participants, which impaired compliance with reporting. Nevertheless, for the majority of patients with visual pathway glioma early stabilisation/improvement of vision, mainly visual acuity, was reported. Single patient histories were rather heterogeneous, rendering it difficult to provide a comprehensive statement regarding neurologic function in view of interim surgery for hydrocephalus or tumour or second and third line non-surgical treatments during long-term follow-up.

Our patient cohort was recruited from 11 European countries, all of them with a unique national health system. Comparing the characteristics of this trial cohort to the published series of Stokland *et al.* and Gnekow *et al.* [3], [4], which were fairly complete for their countries, suggests that the large number of randomised patients is representative.

### 4.2 Toxicity

Overall, there were more haematological toxic events and infections reported in the VCE-arm, although overall treatment time during the induction up to week 21 did not differ between the two arms. On the other hand, fewer drug sensitivity reactions were reported in the VCE-arm. The explanation for this latter significant observation suggests an interaction between etoposide and carboplatin influencing mechanisms of hypersensitivity. Management of hypersensitivity according to protocol recommendations did not jeopardise intention-to-treat analysis. This unexpected finding could not be further analysed for the interaction between etoposide and hypersensitivity on progression risk because of diverse treatment substitutions and variations in duration of ongoing therapy after carboplatin discontinuation.

Detailed reporting of toxicities revealed a significant number of side-effects without differences between the two chemotherapy arms. It cannot be differentiated however, if they rather reflect the overall burden of treatment or the general condition of the patients (e.g. diencephalic syndrome). This relates specifically to neurotoxicity (grade 3 and 4), which was reported during induction for 18 VC- and 17 VCE-patients for motor and for 13 VC- and 14-VCE-patients for sensory impairment (Table 3). The reduced number of relevant toxicities during consolidation most probably followed the extended treatment intervals.

Thirty-eight patients interrupted induction or consolidation therapy without progression or death, (10 during induction, 28 during consolidation). During induction, four interruptions related to toxicity, and one each for non-compliance, visual deterioration, neurological deterioration, physician's and patient's decision and unknown. During consolidation ten incidents interrupted for allergy, eight for toxicity, four due to patient's refusal/intolerance, three attributable to physician's decision, three unknown. This significant proportion of patients interrupting therapy emphasises the importance of considering the tolerability of therapy, particularly for those with non-progressive disease. Selection of patients with the highest risk of progressive disease will be important to justify the use of therapies with significant risks of such toxicities in the future.

### 4.3 Patient tumour and treatment-related risk factors

#### 4.3.1 Age

Early reports of chemotherapy in LGG had focussed on younger children below 5 years, but had included older patients. The COG 9952 trial set the upper age limit at 10 years, whilst this study extended age range to 16 years [13]. The population based series and clinical trials have all identified children younger than 1 year at diagnosis at significantly higher risk for early progression and death [3], [4], [13], especially if diagnosed at age below 6 months with diencephalic syndrome and/or tumour dissemination [10]. A number of small institution-based trials reported conflicting results with regard to the influence of age on response or progression [8], [9], [12], [31]. Our trial confirms a noticeably lower PFS and OS for infants and children with diencephalic syndrome [2], which is associated with large, centrally located tumours. The majority of patients with progression at week 24 are from this very young group with diencephalic syndrome.

This trial does identify an impaired PFS for the greater than 8-year-old group in the VCE-arm, with a lower OS for the older group in both arms. Both these observations were unexpected. We had not intended to randomise between VC and VCE in the entire larger than 8-year age group as radiotherapy was originally proposed as primary treatment for patients with progressive or symptomatic disease. Compliance with treatment allocation across all centres has not been specifically studied in this age group, nor has analysis of anatomical or symptom subtypes being offered RT versus chemotherapy. The numbers of patients from this age group are relatively small with 48 (VC) and 48 (VCE) patients being drawn from the 8- to 16-year-old age group compared to 163 (VC) and 165 (VCE) in the main 1-<8-year age group and 38 (VC) and 35 (VCE) in the <1-year group. Finally there is a greater proportion of non-pilocytic tumours at non-visual pathway supratentorial midline locations in these arms, mirroring age incidence patterns described by Stokland *et al.* [4] Randomisation was not stratified for histology, but non-pilocytic, i.e. diffuse astrocytoma does not predict for PFS in this analysis.

For these reasons the difference between VC and VCE regarding PFS in the older age group is of interest but requires additional data and analysis to suggest an interpretation. The observation of reduced OS in the older age group similarly raises questions related to the impact of tumours arising later in childhood. The previous COG study excluded children over 10 years [13]. Future studies will need to consider carefully, stratification by age groups, obtain biological data about tumour types and recording of pubertal status if the impact of late childhood and adolescence on tumour behaviour and sensitivity to treatment is to be better understood.

#### 4.3.2 Dissemination

Primary dissemination of LGG has been described to be an unfavourable prognostic factor [32], [33], confirmed by this report with an impaired PFS. The impact of dissemination is statistically noticeable, suggesting that patients with disseminated tumours experience progression more frequently even following non-progression at response assessment at week 24.

#### 4.3.3 Tumour site

Our randomised cohort was stratified for tumour location, assuming a favourable prognosis for smaller visual pathway tumours, primarily involving the chiasm, rather than the more extensive chiasmatic-hypothalamic tumours and other supratentorial midline locations [2]. We could not identify tumour site as a prognostic factor for PFS or OS, in contrast to the unfavourable prognosis for thalamic tumours in the COG trial [13]. The issue of the potential role of tumour dimension in predicting response to therapy and more importantly ultimate patients' outcome still remains an open question.

#### 4.3.4 Histology

The impact of the different low grade histologies on response to chemotherapy, PFS and OS has not been systematically investigated in the past. Pilocytic astrocytoma is the majority group in all trials. Clinically diagnosed patients without biopsy are also included in previous reports, particularly where tumours involve visual pathways and hypothalamus. A trend for a higher progression rate was reported for fibrillary/diffuse astrocytoma in the COG 9952 trial [13], but was not confirmed in this multivariable analysis. The analysis of Stokland identified that fibrillary/diffuse astrocytoma had a significantly lower PFS rate, the cohort included treated and untreated as well as NF1 and non-NF1 patients [4]. In this study, histological subtype did not predict PFS but is a relevant prognostic factor for OS. We conclude that patients with diffuse glioma WHO grade 2 have a trend for poorer outcomes and need to be offered further treatment, if they progress after chemotherapy. Glioneuronal and pilocytic histology had the most favourable OS; the other groups including imaging-diagnosed patients had inferior OS. Emerging knowledge of histological and molecular genetic subgrouping will be explored in future trials.

#### 4.3.5 Surgery

In the COG 9952 study, patients were eligible and started chemotherapy, if they were newly diagnosed with less than 95% resection or had residual tumour >1.5 cm^2^ (72% of patients) or had progression after surgery (27% of patients) (unknown 1%) [13]. In our trial, the presence of a postoperative residual was no indication to chemotherapy on its own. Patients also had to have severe tumour-related symptoms or tumour progression. More than 60% of patients started treatment within 3 months of diagnosis. Those starting treatment following a phase of observation had pure radiological progression in 19.5%, while the others had symptomatic plus radiological progression. The actual tumour dimension at the time of starting therapy was not consistently recorded particularly for visual pathway or hypothalamic-chiasmatic glioma in the present study. Thus the impact of tumour dimension on tumour response and PFS could not be reliably studied. The extent of initial tumour resection was not an independent prognostic risk factor at multivariable analysis. Residual disease alone, in our view, is not an indication for non-surgical therapy.

#### 4.3.6 Chemotherapy

With only few formal phase II studies, the introduction and use of chemotherapy for LGG largely based upon results from small patient series, including regimens with vincristine, actinomycin D, cyclophosphamide, cis- or carboplatin, procarbazine, CCNU, thioguanine, etoposide or vinblastine [8], [9], [14], [15], [16], [17], [18], [19], [20], [27], [34], [35], [36]. As well, a small series investigated the combination of bevacizumab and irinotecan, vinorelbine or temozolomide [37], [38], [39], [40], [41], [42]. Efficacy was judged by assessing radiological tumour response, while survival (OS) and progression-free survival (PFS) data were not always available. All drug combinations produce comparable ‘response rates’ without offering any better efficacy over others. A comparative analysis of the respective toxicities is hampered by the heterogeneous use of drugs or combinations as first line or salvage treatments. Despite a large number of publications, only 23 articles on the use of CT for visual pathway glioma met the inclusion criteria for a systematic research in 2006 [30]. Yet, except for two, most cohorts were neither well-defined nor representative or lacked complete follow-up, and thus did not allow analysis of prognostic risk factors. Within the last 30 years only two prospective randomised trials systematically investigated chemotherapy [13], one being this report.

Following the identification of mutations within genes for the MAPKinase pathway a major signalling pathways within LGG [43], [44], [45], tumour biology needs to be assessed by integrating molecular and histological factors, as well as clinical criteria for prognostic impact to be established in prospective clinical trials. Future randomised trials should continue to be stratified by non-NF1 and NF1-associated cases and to examine the impact of the current ‘standard’ drug regimen with new targeted therapies and their effects on tumour response and functional outcomes and toxicities. A revised set of internationally standardised eligibility criteria, response definitions and procedures are in development for the next generation of trials which will include ophthalmologic, neurological and QoL outcomes as primary outcome measures in addition to the standard PFS and OS. Their design will permit serial drug testing as new treatments emerge.

## 5 Conclusions

We conclude that the good early non-progression rates measured at 6 months in newly diagnosed cases justify retaining VC as the standard arm for future phase III trials seeking to explore the use of new first line treatments with less toxicity. The new MAPKinase pathway targeted drugs becoming available offer the opportunity to be tested as part of either primary therapy randomisation in newly diagnosed cases or in patients who are progressing during, or at the end of induction. Trials should be designed to discriminate between the impact of ‘duration of therapy’ and ‘age at stopping therapy’ as being factors that may determine progression risk. Those presenting in the 1st year of life with diencephalic syndrome and progressive disease, despite therapy, need new treatments to be tested as they are at greatest risk of suffering neurological and endocrine damage and are at high risk of death. The >8-year-old group needs further study to understand the interactions between age, pubertal development, histology and sensitivity to chemotherapy.

**Research in context**

**Evidence before this study**

Childhood low grade glioma are predominantly pilocytic grade 1 tumours with a small proportion of grade 2 tumours and other rare entities, a proportion are diagnosed without biopsy on imaging characteristics. Together they represent 40% of all childhood brain tumours and are the commonest solid tumour of childhood requiring active management. A small proportion of these non-malignant tumours is thought to eventually transform to more malignant phenotype in adulthood. Currently there are no established predictive factors for this risk. Most are sporadic, arising within a single location including hypothalamus, chiasm, optic nerves, cerebellum, cerebral cortex, brainstem and spinal cord, with primary and secondary dissemination in 5–10%. About 15% are associated with neurofibromatosis type 1 (NF1), with a majority of tumours affecting the visual pathways, many being multifocal, but also involving brainstem and cerebellum. In NF1, surveillance programmes of visual function are in place to identify children with visual changes justifying brain imaging. Sporadic tumours, on the other hand, present clinically throughout childhood although there are classical age distributions associated with different anatomical locations i.e. hypothalamic chiasmatic tumours most commonly present in the first 2 years of life, the younger the patient the larger the tumour. Cerebellar tumours can occur throughout childhood. Cortical tumours tend to occur in later childhood, as do brainstem and spinal tumours.

Multidisciplinary strategy: Previous multicentre pilot study had tested the applicability and acceptability of a multidisciplinary treatment selection versus observation strategy across Europe and found high clinical compliance. Furthermore, patient, genetic, symptom, anatomical and tumour factors had been identified as stratifying factors for treatment selection.

Treatment: Historically treatment has relied upon primary resection, where clinically feasible, without neurotoxicity. Where not feasible and if progressive, radiotherapy was used leading in many cases to severe neurotoxicity affecting the child's subsequent growth, endocrine and cognitive development and risk of second tumours. In the 1980s chemotherapy was reported to successfully control tumour progression in very young children with progressive, unresectable tumours. Vincristine and actinomycin D were used initially. We had conducted a prolonged international pilot study investigating the role of vincristine carboplatin combination in carefully selected patients which has been reported in two publications studying the impact of selection criteria on progression risk and the interaction between patient and treatment factors on progression free and overall survival.

At the time of launching this trial we knew:

• We would expect high overall survival rates (>85% 5-year OS) (children under 1 year of age at diagnosis with diencephalic syndrome and hypothalamic tumours having the poorest overall survival);
• over 60% of LGGs were pilocytic astrocytoma;
• grade 2 diffuse astrocytoma had poorer progression-free survival;
• Visual pathway glioma and other LGGs associated with NF1 had a favourable prognosis for progression-free and overall survival. In view of their cancer predisposition they should be studied separately;
• genetic biomarkers of LGGs have not been identified.
• radiotherapy had been used and demonstrated to produce both symptomatic and imaging responses, saving vision in a significant proportion;
• cranial radiotherapy to the visual pathways and hypothalamus was associated with significant cognitive and endocrine morbidity that progressed through childhood and adolescence leading to lifelong disability;
• where chemotherapy was used, drug resistance did not seem to occur;
• drug selection was being driven by additional risks of hearing loss (cisplatin) in visually impaired children as well as long-term risk of genotoxicity, particularly in NF1 associated cases
• tumour responses to chemotherapy were possible in about half of all patients, we had observed pseudo-progression at 12-week assessments in our previous pilot trial;
• early true progression occurred in less than 30% and was predominantly associated with early age at onset (<1 year) in patients with hypothalamic chiasmatic tumours, frequently associated with diencephalic syndrome and vision loss;
• late/sustained progression also occurred after stopping treatment, particularly in younger children with hypothalamic chiasmatic tumours, sometimes justifying multiple lines of surgical and non-surgical therapy, including radiotherapy
• it was unresolved whether the tendency for tumours to recur/progress reduces as patients got older. Re-progression was being observed during adolescence;
• the accepted age for consideration of radiotherapy over chemotherapy had risen from 5 years to older ages by the date of launch of this trial;
• saving vision was an important target in the parents' judgement at time of diagnosis and during treatment, influencing case selection for non-surgical therapy;
• methods for vision assessment in children up to adolescence were complex with limited consensus across Europe. Direct contact with vision specialists by those managing the chemotherapy varied considerably across the participating countries;
• methods for recording changes in neurological status linked to tumour effects were complex and remained in development;
• A single randomised trial of two (vincristine and carboplatin) versus four drugs (thioguanine, procarbazine, CCNU and vincristine) given over 12 months was in progress within the Children's Oncology Group (COG) in USA. The VC arm and the TPCV arm included three genotoxic drugs each respectively. The duration of therapy was 12 months.

**Rationale for our trial**

Given these circumstances and our experience of piloting the strategy for patient selection for observation or treatment after primary surgery, and focussing on those with evidence, or at risk, of symptomatic progression, we decided to test whether adding a third drug (etoposide), given at doses selected to limit genotoxicity, and not associated with known long term side-effects, would improve response/non-progression rates, progression-free survival or overall survival. We selected 24 weeks as the time to assess response/progression.

**Added value of this study**

This is the first multicentre, European and largest randomised trial worldwide investigating the role of chemotherapy in children with LGG. The primary hypothesis that adding an additional drug to standard two-drug induction treatment would improve response or non-progression rates was not supported.

As a result of this study we have identified that:

The eligibility criteria are acceptable and applicable for selecting patients with progressive disease and risk of symptomatic progression as well defined criteria for study of patients recruited at diagnosis.

We can expect over 90% non-progression rate at 24 weeks for newly diagnosed patients with progressive disease and those at risk of symptomatic progression, offering reassuring evidence for the use of standard VC chemotherapy for newly diagnosed patients.

Twenty-four-week response/progression assessment identified those with early progression and high risk of mortality (52–54% mortality).

The relevance of proposed clinical risk factors, like young age, diencephalic syndrome, metastatic disease, specific histological subtypes, is corroborated with previous reports.

Late or sustained progression remains a phenomenon with some patients requiring multiple lines of surgical and non-surgical therapy.

Prolonged treatment for LGG is feasible, yet it remains to be shown whether it brings with itself a survival advantage. The question of the optimal duration of treatment should be addressed. Toxicity of the vincristine/carboplatin (VC) regimen remains significant;

The interaction between VC and etoposide, resulting in a reduced risk for carboplatin related hypersensitivity, is unexplained.

Risk of vision loss is a driver for consideration for therapy requiring enhanced consensus on methods of measuring risk of vision loss across childhood.

The lack of strong evidence of the vision sparing qualities of this treatment in patients who are treated for vision threat alone is a topic for future research which has already been initiated in NF1 associated LGG, and will influence the approach to sporadic VPG, as a consensus on methods for vision assessment is now emerging.

Risk of neurological consequences for LGGs in different anatomical locations is predictable and methods for their measurement and therefore correlation with tumour response and toxicity of therapies are now established and are being incorporated into clinical trials platforms for the future.

**Clinical conclusions**

According to our present results, vincristine and carboplatin remains the standard treatment for newly diagnosed patients with LGG with progressive disease or at risk of symptomatic disease in Europe. It of course needs further comparison with other drug combinations.

Early evidence of progression by 24 weeks indicates a high mortality risk (justifying trial of new MAPKinase pathway targeted drugs alone or in combination with existing chemotherapy agents, aimed at testing their effectiveness and toxicity).

Radiotherapy is increasingly being reserved for after second or third line chemotherapy in children who continue to progress on chemotherapy.

Age <1 year at diagnosis, diencephalic syndrome and early progression are markers of risk of sustained progression and higher mortality.

Treatment selections at such progressions should be driven by risk of symptomatic progression rather than imaging.

These patients present with complex problems affecting multiple systems justifying multidisciplinary team decision-making. Increasing complexity may justify centralised decision systems nationally.

**Research conclusions**

Future studies of newly diagnosed patients for non-surgical therapy should be selected by standardised criteria for estimating risk of symptomatic progression. Ideally, these criteria should be standardised internationally to enhance comparability of results. We propose the SIOP LGG criteria as a candidate for such an international consensus.

Basic consideration for the design of a treatment of a new trial are:

a) the high non-progression rates using VC,
b) the lack of evidence of additional benefit of an additional drug,
c) the high hypersensitivity rates for carboplatin and
c) the significant neurotoxicity of vincristine

**We propose that:**

• Future trials aim to identify less toxic and more effective treatments (especially with respect to visual function for visual pathway glioma) in comparison to the standard VC combination. The new agents targeting the MAPKinase pathway are likely to be candidates tested alone or in combination with existing chemotherapy agents. PFS, visual, neurological and QoL measures should be considered for primary outcome in newly diagnosed cases in future trials.
• Those who progress by 24 weeks, despite chemotherapy, can also be considered for new MAPK targeted therapies that are emerging from the current bioresearch agenda.
• Outcome criteria should include non-progression rates at 24 weeks, symptom response assessing vision and neurology and measures of toxicity and tolerability, as well as PFS and OS.
• The optimal duration of therapy remains to be established overall and for different age groups.
• The explanation for sustained progression may be linked to ‘duration of therapy’ or ‘age at discontinuation of therapy’, if age is a proxy for factors linked to brain growth in different anatomical regions.
• Trials designed to assess both ‘duration of therapy hypothesis’ and ‘age at finishing treatment hypothesis’ are candidates for future research.
• Elucidating the mechanism of etoposide modification of carboplatin hypersensitivity may offer a mechanism for managing this consequence of prolonged carboplatin exposure in the future.

Those who progress by 24 weeks, despite chemotherapy, can also be considered for new MAPK targeted therapies that are emerging from the current bioresearch agenda.

Outcome criteria should include non-progression rates at 24 weeks, symptom response assessing vision and neurology and measures of toxicity and tolerability, as well as PFS and OS.

The optimal duration of therapy remains to be established overall and for different age groups.

The explanation for sustained progression may be linked to ‘duration of therapy’ or ‘age at discontinuation of therapy’, if age is a proxy for factors linked to brain growth in different anatomical regions.

Trials designed to assess both ‘duration of therapy hypothesis’ and ‘age at finishing treatment hypothesis’ are candidates for future research.

Elucidating the mechanism of etoposide modification of carboplatin hypersensitivity may offer a mechanism for managing this consequence of prolonged carboplatin exposure in the future.

## Support

The German national group is supported by the German Children's Cancer Foundation. The Reference Centre for Biostatistics (Institute of Biostatistics and Clinical Research, University of Muenster, Germany) was supported by grants of the German Children's Cancer Foundation.

Cancer Research UK Clinical Trials Unit (CRCTU), University of Birmingham provided access to the UK final database. We thank Cancer Research UK for funding the LGG2 (CNS 2004 03) trial (Ref: C13189/A13205).

The Clinical Trial & Biostatistics Unit of Istituto Oncologico Veneto, Padova, Italy, was supported by Citta della Speranza Foundation.

The French national group was supported by the Institut National du Cancer, Programe Hospitalier de Recherche Clinique.

## Conflict of interest statement

None declared.
