# Corrigendum to “A European randomised controlled trial of the addition of etoposide to standard vincristine and carboplatin induction as part of an 18-month treatment programme for childhood (≤16 years) low grade glioma – A final report” [Eur J of Canc (2017) 206–225]

**DOI:** 10.1016/j.ejca.2017.11.017

**Published:** 2018-02

**Authors:** Astrid K. Gnekow, David A. Walker, Daniela Kandels, Susan Picton, Giorgio Perilongo, Jacques Grill, Tore Stokland, Per Eric Sandstrom, Monika Warmuth-Metz, Torsten Pietsch, Felice Giangaspero, René Schmidt, Andreas Faldum, Denise Kilmartin, Angela De Paoli, Gian Luca De Salvo, Low Grade Glioma Consortium and the participating centers, Astrid K. Gnekow, Irene Slavc, Giorgio Perilongo, Sue Picton, David Walker, Tore Stokland, Per Erik Sandstrom, Niels Clausen, Mikko Arola, Olafur Gisli Jonsson, Ofelia Cruz, Aurora Navajas, Anna Teijeiro, Jacques Grill, Chantal Kalifa, Marie-Anne Raquin, Joris Verlooy, Volkmar Hans, Torsten Pietsch, Wolfram Scheurlen, Johannes Hainfellner, Felice Giangaspero, James Ironside, Keith Robson, Kari Skullerud, David Scheie, NN, Marie-Madeleine Ruchoux, Anne Jouvet, Dominique Figarella-Branger, Arielle Lellouch-Toubiana, Monika Warmuth-Metz, Daniela Prayer, Milena Calderone, Tim Jaspan, Soren Jacob Bakke, Eli Vazquez, Dominique Couanet, Rolf D. Kortmann, Karin Diekmann, Giovanni Scarzello, Roger Taylor, Knut Lote, Jordi Giralt, Christian Carrie, Jean Louis Habrand, Niels Soerensen, Thomas Czech, Paul Chumas, Bengt Gustavson, Michel Zerah, Bettina Wabbels, Maria Luisa Pinello, Alistair Fielder, Ian Simmons, Terje Christoffersen, Gabriele Calaminus, Knut Brockmann, Ronald Straeter, Friedrich Ebinger, Pablo Hernaiz-Driever, Herwig Lackner, Colin Kennedy, Adam Glaser, Bo Stromberg, Jose Ma Indiano, Chantal Rodary, Eric Bouffet, Didier Frappaz, Andreas Faldum, Angela Emser, Gian Luca De Salvo, Suzanne Stephens, David Machin, Marie-Cécile Le Deley, Thore Egeland, Carolyn Freemann, Martin Schrappe, Richard Sposto

**Affiliations:** m Pediatric Oncology, GPOH, Germany; n Pediatric Oncology, GPOH, Austria; o Pediatric Oncology, AIEOP, Italy; p Pediatric Oncology, UK; q Pediatric Oncology, NOPHO, Nordic Countries; r Pediatric Oncology, SEOP, Spain; s Pediatric Oncology, SFCE, France; t Pediatric Oncology, BSPHO, Belgium; u Pathology & Molecular Biology, GPOH, Germany; v Pathology & Molecular Biology, GPOH, Austria; w Pathology & Molecular Biology, AIEOP, Italy; x Pathology & Molecular Biology, UK; y Pathology & Molecular Biology, NOPHO, Nordic Countries; z Pathology & Molecular Biology, SEOP, Spain; aa Pathology & Molecular Biology, SFCE, France; ab Radiology, GPOH, Germany; ac Radiology, GPOH, Austria; ad Radiology, AIEOP, Italy; ae Radiology, UK; af Radiology, NOPHO, Nordic Countries; ag Radiology, SEOP, Spain; ah Radiology, SFCE, France; ai Radiotherapy, GPOH, Germany; aj Radiotherapy, GPOH, Austria; ak Radiotherapy, AIEOP, Italy; al Radiotherapy, UK; am Radiotherapy, NOPHO, Nordic Countries; an Radiotherapy, SEOP, Spain; ao Radiotherapy, SFCE, France; ap Neurosurgery, GPOH, Germany; aq Neurosurgery, GPOH, Austria; ar Neurosurgery, AIEOP, Italy; as Neurosurgery, UK; at Neurosurgery, NOPHO, Nordic Countries; au Neurosurgery, SEOP, Spain; av Neurosurgery, SFCE, France; aw Ophthamology, GPOH, Germany; ax Ophthamology, GPOH, Austria; ay Ophthamology, AIEOP, Italy; az Ophthamology, UK; ba Ophthamology, NOPHO, Nordic Countries; bb Ophthamology, SEOP, Spain; bc Ophthamology, SFCE, France; bd Neuropediatrics, Health Status/Quality of Life, GPOH, Germany; be Neuropediatrics, Health Status/Quality of Life, GPOH, Austria; bf Neuropediatrics, Health Status/Quality of Life, AIEOP, Italy; bg Neuropediatrics, Health Status/Quality of Life, UK; bh Neuropediatrics, Health Status/Quality of Life, NOPHO, Nordic Countries; bi Neuropediatrics, Health Status/Quality of Life, SEOP, Spain; bj Neuropediatrics, Health Status/Quality of Life, SFCE, France; bk Associated Research Phase II – Studies, Toronto, Canada; bl Associated Research Phase II – Studies, Lyon, France; bm Biostatistics, GPOH, Germany; bn Biostatistics, AIEOP, Italy; bo Biostatistics, UK; bp Biostatistics, SFCE, France; bq Biostatistics, NOPHO, Nordic Countries; br Data Monitoring & Safety Committee, Montreal, Canada; bs Data Monitoring & Safety Committee, Kiel, Germany; bt Data Monitoring & Safety Committee, Arcadia, CA, USA; a Swabian Children's Cancer Center, Klinikum Augsburg, Germany; b Children's Brain Tumour Research Centre, University of Nottingham, UK; c Leeds Children's Hospital, UK; d Division of Pediatrics, University Hospital of Padua, Italy; e Institut Gustave Roussy, Paris, France; f University Hospital of North Norway, Norway; g Umea University, Sweden; h Department of Neuro-radiology, University of Wurzberg, Germany; i University of Bonn Medical Centre, Germany; j University of Rome Sapienza, Italy; k Institute of Biostatistics and Clinical Research, University of Muenster, Germany; l Clinical Trials and Biostatistics Unit, IRCCS Istituto Oncologico Veneto, Padova, Italy

The authors regret that the original article did not include an acknowledgement of the Dutch Childhood Oncology Group as part of the International Study Committee. The authors would like to add the following acknowledgement:

Netherlands – DCOG: Netteke Schouten

The authors would like to apologise for any inconvenience caused.

