# Supplementary material for: A European randomised controlled trial of the addition of etoposide to standard vincristine and carboplatin induction as part of an 18-month treatment programme for childhood (≤16 years) low grade glioma – A final report
Source: Eur J Cancer. 2017 Aug;81:206–25. doi: 10.1016/j.ejca.2017.04.019 (PMC5517338; doi:10.1016/j.ejca.2017.04.019)
Supplement: Supplementary file 1 [file mmc1.docx]

**Supplementary Methods**

**Survival Analysis**

Analysis of univariable prognostic and predictive impact

To analyse the univariable prognostic and predictive value of clinical and biologic variables on OS and PFS, Cox regression models were fitted for each variable separately while allowing treatment group and their two-fold interaction in a second block, using stepwise forward selection (inclusion: P value of the score test ≤ 0.05, exclusion: P value of the likelihood ratio test > 0.1). Results are summarized in Tables 5b and 5c.

Multivariable Model Building

Multivariable Cox regression models were built using the following inductive variable selection procedure. To settle the base case, all factors were fitted together by a stepwise forward selection of a multivariable Cox regression to test their prognostic impact. The factors chosen in this forward selection define the model at step 1. To settle the induction step, assume that the model at step j for some j ≥ 1 is known. In step j+1, all factors not selected for the model at step j were added to the model at step j in a second block, to test their prognostic impact by a stepwise forward selection in the second block. All factors selected after this step were fitted together by a stepwise backward selection of a multivariable Cox regression. The factors remaining after this backwards selection define the model at step j+1. In this way, model building continued inductively up until the procedure stopped after finitely many steps. Finally, the factors of the last step when the above variable selection procedure stopped were fitted with all pair-wise interactions and all 2-fold interactions with treatment group (to assess their predictive impact) in a second block by a stepwise forward selection. The model found after this step is the final model. At all steps of variable selection, the inclusion criterion was: P value of the score test ≤ 0.05, and the exclusion criterion was: P value of the likelihood ratio test > 0.1. Results are summarized in Table 5d.

**Biostatistical Amendment:**

Based on the decision of the trial committee from December 6, 2009, a biostatistical amendment was performed. The original group sequential design was advanced to an adaptive design[^23^](#_ENREF_23) and, on this basis the accrual period was prolonged by 2 years to a total of 8 years with a subsequent follow-up period of 2 years. The amendment was approved by the data monitoring committee on August 10, 2010, the leading ethics committee on April 27, 2010, and by the national trial groups thereafter.

**Supplementary Tables**

**Table 5a**

**Univariable analysis: Kaplan-Meier estimates of 5-year progression-free survival (PFS) and overall survival (OS) by randomization arm with standard deviation and p value of log-rank test.**

|  | 5 yrs – PFS | | | | 5 yrs – OS | | | |
| --- | --- | --- | --- | --- | --- | --- | --- | --- |
|  | VC | | VCE | | VC | | VCE | |
|  | n | % | n | % | n | % | n | % |
| Total | 247 | 46.1 (3.5) | 243 | 45.3 (3.5) | 247 | 89.2 (2.1) | 244 | 88.8 (2.1) |
| Age group – Strata at randomization |  | **P<0.001** |  | **P=0.002** |  | **P<0.001** |  | **P<0.001** |
| < 1 year | 38 | 12.4 (5.7) | 34 | 28.2 (8.5) | 38 | 69.3 (8.2) | 34 | 76.5 (7.3) |
| 1 to < 8 years | 163 | 51.9 (4.3) | 161 | 52.1 (4.3) | 163 | 93.9 (2.0) | 162 | 94.3 (2.0) |
| >=8 years | 46 | 53.3 (8.2) | 48 | 34.9 (7.5) | 46 | 88.9 (4.7) | 48 | 79.8 (6.1) |
| **Indication to treatment** |  | **P<0.001** |  | **P<0.001** |  | **P<0.001** |  | **P=0.127** |
| DS | 28 | 19.5 (8.1) | 28 | 12.2 (6.5) | 28 | 68.2 (9.3) | 29 | 78.9 (7.7) |
| Visual impairment | 70 | 39.1 (6.4) | 80 | 51.6 (6.1) | 70 | 95.3 (2.7) | 80 | 89.8 (3.7) |
| Radiol. progression | 85 | 59.1 (5.9) | 78 | 44.6 (6.0) | 85 | 94.5 (2.7) | 78 | 93.2 (3.0) |
| Neurol. symptoms | 64 | 48.4 (6.7) | 57 | 56.6 (7.1) | 64 | 84.3 (4.9) | 57 | 87.1 (4.6) |
| Primary tumor site |  | **P=0.930** |  | **P=0.364** |  | **P=0.211** |  | **P=0.433** |
| Caudal brainstem | 41 | 42.7 (8.4) | 37 | 50.5 (8.8) | 41 | 80.0 (7.0) | 37 | 94.3 (3.9) |
| Spinal cord | 18 | 58.2 (13.8) | 22 | 40.7 (12.3) | 18 | 100.0 | 22 | 90.7 (6.3) |
| Cerebellum | 16 | 66.7 (12.2) | 14 | 71.4 (12.1) | 16 | 100.0 | 14 | 100.0 |
| Cerebral hemisphere | 16 | 39.5 (12.9) | 16 | 37.5 (12.1) | 16 | 80.0 (10.3) | 16 | 87.5 (8.3) |
| SM, visual pathway | 89 | 43.5 (5.7) | 80 | 42.2 (6.0) | 89 | 89.7 (3.5) | 80 | 92.1 (3.1) |
| SM, other | 67 | 45.2 (6.8) | 74 | 43.7 (6.4) | 67 | 90.9 (3.5) | 75 | 79.3 (5.2) |
| **Localization – Strata at randomization** |  | **P=0.982** |  | **P=0.138** |  | **P=0.945** |  | **P=0.098** |
| SM Dodge II | 25 | 38.4 (10.8) | 22 | 59.7 (11.1) | 25 | 90.8 (6.2) | 22 | 100.0 |
| SM Dodge III+other | 129 | 44.9 (4.7) | 129 | 40.8 (4.7) | 129 | 90.0 (2.8) | 130 | 83.6 (3.5) |
| All others | 93 | 49.7 (5.7) | 92 | 48.3 (5.6) | 93 | 87.8 (3.7) | 92 | 93.2 (2.7) |
| Extent of resection^a^ |  | **P=0.007** |  | **P=0.846** |  | **P=0.064** |  | **P=0.275** |
| Total /near total | 23 | 81.1 (8.5) | 15 | 42.4 (15.9) | 23 | 100.0 | 15 | 92.3 (7.4) |
| Partial | 85 | 46.5 (6.3) | 96 | 46.6 (5.6) | 85 | 93.3 (2.9) | 97 | 95.4 (2.2) |
| Biopsy | 92 | 37.3 (5.3) | 98 | 47.1 (5.3) | 92 | 83.5 (4.1) | 98 | 86.9 (3.6) |
| No/prior to resection | 41 | 41.7 (8.1) | 28 | 42.2 (10.0) | 41 | 86.5 (6.7) | 28 | 81.6 (7.4) |
| Tumor histology |  | **P=0.754** |  | **P=0.011** |  | **P=0.004** |  | **P<0.001** |
| PA | 139 | 48.7 (4.7) | 144 | 52.8 (4.4) | 139 | 94.1 (2.2) | 145 | 95.3 (1.9) |
| PMA | 17 | 32.9 (12.1) | 19 | 24.8 (11.8) | 17 | 88.2 (7.8) | 19 | 76.0 (10.7) |
| Diffuse glioma | 25 | 47.3 (10.1) | 23 | 38.8 (10.6) | 25 | 80.0 (8.0) | 23 | 65.7 (10.6) |
| Glioneuronal tumors | 15 | 47.5 (14.2) | 14 | 34.3 (13.1) | 15 | 93.3 (6.4) | 14 | 92.9 (6.9) |
| Other histology | 10 | 40.0 (19.3) | 15 | 28.6 (12.1) | 10 | 48.0 (19.1) | 15 | 85.7 (9.4) |
| No histology | 41 | 41.7 (8.1) | 28 | 42.2 (10.0) | 41 | 86.5 (5.6) | 28 | 81.6 (7.4) |
| Metastasis |  | **P=0.002** |  | **P=0.014** |  | **P=0.855** |  | **P=0.931** |
| No | 220 | 49.6 (3.6) | 202 | 49.5 (3.8) | 220 | 89.3 (2.2) | 203 | 88.9 (2.4) |
| Yes | 27 | 9.5 (8.4) | 41 | 26.9 (7.2) | 27 | 88.3 (6.4) | 41 | 87.8 (5.1) |
| Response at week 24 |  | **P=0.377** |  | **P=0.170** |  | **P<0.001** |  | **P<0.001** |
| CR/PR/OR | 97 | 49.8 (5.5) | 85 | 54.0 (5.8) | 97 | 97.3 (1.9) | 86 | 97.7 (1.6) |
| SD | 95 | 54.8 (5.8) | 104 | 48.1 (5.5) | 96 | 94.4 (2.8) | 105 | 92.8 (2.6) |
| PD^b^ | - | - | - | - | 14 | 46.4 (13.8) | 18 | 48.5 (16.5) |

OS and PFS calculated from date of randomization (to evaluate Response Status: Calculated from date of response assessment at week 24).

^a^ Missing values pooled over both therapy groups: Extent of resection: 12 patients with missing information.

^b^ Patients with PD at week 24 are considered for OS, only, since PD is part of the event definition for PFS.

**Table 5b**

**Univariable prognostic and predictive impact of markers with respect to PFS. Cox regression models for the complete randomized cohort considering a single factor together with treatment group and their two-fold interaction in a second block (inclusion:** **P value of the score test ≤ 0.05, exclusion: P value of the likelihood ratio test > 0.1). Hazard ratio (HR), 95% confidence interval (CI), and p value of likelihood ratio test for the final model.**

| **Variable** | **Available cases** | **HR** | **95% CI** | **P*** |
| --- | --- | --- | --- | --- |
| Age – Strata at randomization  (categorical: <1, 1 to <8, >=8 years) | 490 |  |  | <0.001 |
| Age impact in VC | 247 |  |  |  |
| < 1 vs >=1 to <8 years | 38 v 163 | 3.34 | 2.28 to 4.91 |  |
| >=8 vs >=1 to <8 years | 46 v 163 | 0.98 | 0.62 to 1.54 |  |
| Age impact in VCE | 243 |  |  |  |
| < 1 vs >=1 to <8 years | 34 v 161 | 2.09 | 1.36 to 3.22 |  |
| >=8 vs >=1 to <8 years | 48 v 161 | 1.70 | 1.15 to 2.49 |  |
| Treatment group (VCE vs VC) |  |  |  | N/S** |
| 2-fold interaction |  |  |  | 0.031 |
|  | | | | |
| Gender | 490 |  |  | 0.649 |
| Female vs male | 227 v 263 | 0.95 | 0.74 to 1.21 |  |
| Treatment group (VCE vs VC) |  |  |  | N/S** |
| 2-fold interaction |  |  |  | N/S** |
|  | | | | |
| Indication to treatment | 490 |  |  | <0.001 |
| DS vs  Radiological progression | 56 v 163 | 3.33 | 2.32 to 4.78 |  |
| Visual impairment vs  Radiological progression | 150 v 163 | 1.02 | 0.74 to 1.40 |  |
| Neurological symptoms vs Radiological progression | 121 v 163 | 1.17 | 0.84 to 1.63 |  |
| Treatment group (VCE vs VC) |  |  |  | N/S** |
| 2-fold interaction |  |  |  | N/S** |
|  | | | | |
| Primary tumor site | 490 |  |  | 0.336 |
| Caudal brainstem vs SM, visual pathway | 78 v 169 | 1.04 | 0.72 to 1.50 |  |
| Spinal cord vs SM, visual pathway | 40 v 169 | 0.98 | 0.61 to 1.57 |  |
| Cerebellum vs SM, visual pathway | 30 v 169 | 0.57 | 0.30 to 1.10 |  |
| Cerebral hemisphere vs SM, visual pathway | 32 v 169 | 1.24 | 0.76 to 2.03 |  |
| SM, other vs SM, optic pathway | 141 v 169 | 1.13 | 0.84 to 1.53 |  |
| Treatment group (VCE vs VC) |  |  |  | N/S** |
| 2-fold interaction |  |  |  | N/S** |
|  | | | | |
| Localization – Strata at randomization | 490 |  |  | 0.375 |
| All others vs SM Dodge III+other | 185 v 258 | 0.88 | 0.68 to 1.13 |  |
| SM Dodge II vs SM Dodge III+other | 47 v 258 | 0.77 | 0.49 to 1.20 |  |
| Treatment group |  |  |  | N/S** |
| 2-fold interaction |  |  |  | N/S** |
|  | | | | |
| Extent of surgery | 478 |  |  | 0.027 |
| Total/Near Total vs Biopsy | 38 v 190 | 0.48 | 0.26 to 0.87 |  |
| Partial vs Biopsy | 181 v 190 | 0.78 | 0.59 to 1.02 |  |
| No/prior to resection vs Biopsy | 69 v 190 | 0.97 | 0.68 to 1.42 |  |
| Treatment group (VCE vs VC) |  |  |  | N/S** |
| 2-fold interaction |  |  |  | N/S** |
|  | | | | |
| Tumor histology | 490 |  |  | 0.021 |
| PMA vs PA | 36 v 283 | 1.70 | 1.10 to 2.63 |  |
| Diffuse glioma vs PA | 48 v 283 | 1.55 | 1.03 to 2.33 |  |
| Glioneuronal tumors vs PA | 29 v 283 | 1.50 | 0.89 to 2.52 |  |
| Other histology vs PA | 25 v 283 | 1.87 | 1.13 to 3.10 |  |
| No histology vs PA | 69 v 283 | 1.35 | 0.94 to 1.93 |  |
| Treatment group (VCE vs VC) |  |  |  | N/S** |
| 2-fold interaction |  |  |  | N/S** |
|  | | | | |
| Metastases | 490 |  |  | 0.001 |
| Yes vs no | 68 v 422 | 1.80 | 1.32 to 2.45 |  |
| Treatment group (VCE vs VC) |  |  |  | N/S** |
| 2-fold interaction |  |  |  | N/S** |
|  | | | | |
| Interval from diagnosis to start of treatment (months) | 490 |  |  | 0.066 |
| Interval from diagnosis to start of treatment in VC | 247 | 0.987 | 0.973 to 1.002 |  |
| Interval from diagnosis to start of treatment in VCE | 243 | 1.006 | 0.997 to 1.014 |  |
| Treatment group (VCE vs VC) |  |  |  | N/S** |
| 2-fold interaction |  |  |  | 0.017 |
|  | | | | |
| Response at week 24 | 381 |  |  | 0.696 |
| CR/PR/OR vs SD | 182 v 199 | 0.94 | 0.70 to 1.27 |  |
| Treatment group (VCE vs VC) |  |  |  | N/S** |
| 2-fold interaction |  |  |  | N/S** |

PFS calculated from date of randomization (to evaluate Response Status: Calculated from date of response assessment at week 24).

*For pairwise comparisons, confidence intervals instead of p values are given (p value of Wald test ≤ 0.05 if and only if confidence interval does not contain 1).

**N/S: Not locally significant.

**Table 5c**

**Univariable prognostic and predictive impact of markers with respect to OS. Cox regression models for the complete randomized cohort considering a single factor together with treatment group and their two-fold interaction in a second block (inclusion:** **P value of the score test ≤ 0.05, exclusion: P value of the likelihood ratio test > 0.1). Hazard ratio (HR), 95% confidence interval (CI), and p value of likelihood ratio test for the final model.**

| **Variable** | **Available cases** | **HR** | **95% CI** | **P*** |
| --- | --- | --- | --- | --- |
| Age – Strata at randomization  (categorical: <1, 1 to <8, >=8 years) | 491 |  |  | <0.001 |
| < 1 vs >=1 to <8 years | 72 v 325 | 4.58 | 2.44 to 8.62 |  |
| >=8 vs >=1 to <8 years | 94 v 325 | 2.61 | 1.34 to 5.06 |  |
| Treatment group (VCE vs VC) |  |  |  | N/S** |
| 2-fold interaction |  |  |  | N/S** |
|  | | | | |
| Gender | 491 |  |  | 0.315 |
| Female vs male | 228 v 263 | 0.76 | 0.44 to 1.31 |  |
| Treatment group (VCE vs VC) |  |  |  | N/S** |
| 2-fold interaction |  |  |  | N/S** |
|  | | | | |
| Indication to treatment | 491 |  |  | <0.001 |
| DS vs  Radiological progression | 57 v 163 | 5.02 | 2.32 to 10.83 |  |
| Visual impairment vs  Radiological progression | 150 v 163 | 1.01 | 0.43 to 2.36 |  |
| Neurological symptoms vs Radiological progression | 121 v 163 | 2.21 | 1.04 to 4.72 |  |
| Treatment group (VCE vs VC) |  |  |  | N/S** |
| 2-fold interaction |  |  |  | N/S** |
|  | | | | |
| Primary tumor site | 491 |  |  | 0.080 |
| Caudal brainstem vs SM, visual pathway | 78 v 169 | 1.31 | 0.60 to 1.50 |  |
| Spinal cord vs SM, visual pathway | 40 v 169 | 0.76 | 0.22 to 2.62 |  |
| Cerebellum vs SM, visual pathway | 30 v 169 | NE** |  |  |
| Cerebral hemisphere vs SM, visual pathway | 32 v 169 | 1.66 | 0.61 to 4.54 |  |
| SM, other vs SM, visual pathway | 142 v 169 | 1.50 | 0.78 to 2.90 |  |
| Treatment group (VCE vs VC) |  |  |  | N/S** |
| 2-fold interaction |  |  |  | N/S** |
|  | | | | |
| Localization – Strata at randomization | 491 |  |  | 0.189 |
| All others vs SM Dodge III+other | 185 v 259 | 0.79 | 0.45 to 1.38 |  |
| SM Dodge II vs SM Dodge III+other | 47 v 259 | 0.34 | 0.08 to 1.40 |  |
| Treatment group |  |  |  | N/S** |
| 2-fold interaction |  |  |  | N/S** |
|  | | | | |
| Extent of surgery | 479 |  |  | 0.012 |
| Total/Near Total vs Biopsy | 38 v 190 | 0.19 | 0.03 to 1.39 |  |
| Partial vs Biopsy | 182 v 190 | 0.45 | 0.23 to 0.90 |  |
| No/prior to resection vs Biopsy | 69 v 190 | 1.22 | 0.61 to 2.46 |  |
| Treatment group (VCE vs VC) |  |  |  | N/S** |
| 2-fold interaction |  |  |  | N/S** |
|  | | | | |
| Tumor histology | 491 |  |  | <0.001 |
| PMA vs PA | 36 v 284 | 3.53 | 1.10 to 9.19 |  |
| Diffuse glioma vs PA | 48 v 284 | 6.09 | 1.03 to 12.98 |  |
| Glioneuronal tumor vs PA | 29 v 284 | 2.23 | 0.89 to 7.75 |  |
| Other histology vs PA | 25 v 284 | 6.05 | 1.13 to 15.02 |  |
| No histology vs PA | 69 v 284 | 3.61 | 1.64 to 7.96 |  |
| Treatment group (VCE vs VC) |  |  |  | N/S** |
| 2-fold interaction |  |  |  | N/S** |
|  | | | | |
| Metastases | 491 |  |  | 0.867 |
| Yes vs no | 68 v 423 | 1.07 | 0.50 to 2.26 |  |
| Treatment group (VCE vs VC) |  |  |  | N/S** |
| 2-fold interaction |  |  |  | N/S** |
|  | | | | |
| Interval from diagnosis to start of treatment | 491 | 0.989 | 0.967 to 1.011 | 0.292 |
| Treatment group (VCE vs VC) |  |  |  | N/S** |
| 2-fold interaction |  |  |  | N/S** |
|  | | | | |
| Response at week 24 | 416 |  |  | <0.001 |
| CR/PR/OR vs SD | 182 v 201 | 0.39 | 0.14 to 1.10 |  |
| PD vs SD | 32 v 201 | 11.35 | 5.33 to 24.17 |  |
| Treatment group (VCE vs VC) |  |  |  | N/S** |
| 2-fold interaction |  |  |  | N/S** |

PFS calculated from date of randomization (to evaluate Response Status: Calculated from date of response assessment at week 24).

* For pairwise comparisons, confidence intervals instead of p values are given (p value of Wald test ≤ 0.05 if and only if confidence interval does not contain 1)

**N/S: Not locally significant; NE: Not estimable because of any events in this group

**Table 5d**

**Results of multivariable analysis: Factors selected in the final model with hazard ratio (HR), 95% confidence interval (CI), and p value of likelihood ratio test.**

| **Variable** | **Available cases** | **HR** | **95% CI** | **P*** |
| --- | --- | --- | --- | --- |
| **Overall survival (OS) and progression-free survival (PFS) calculated from date of randomization.** | | | | |
| **PROGRESSION-FREE SURVIVAL^a^** | n=497;  262 events |  |  |  |
| **Age group – Strata at randomization** |  |  |  | **<0.001** |
| **2-fold interaction: Age group - Strata at randomization (ref: 1 to <8) and**  **randomization arm (ref: VC)** |  |  |  | **0.028** |
| **Age impact in VC** |  |  |  |  |
| <1 vs 1 - <8 years | 38 vs 163 | 2.61 | 1.72 to 3.96 |  |
| >=8 vs 1 - <8 years | 46 vs 163 | 1.02 | 0.64 to 1.62 |  |
| **Age impact in VCE** |  |  |  |  |
| <1 vs 1 - <8 years | 34 vs 161 | 1.56 | 0.99 to 2.46 |  |
| >=8 vs 1 - <8 years | 48 vs 161 | 1.75 | 1.18 to 2.60 |  |
|  | | | | |
| **Indication to treatment** |  |  |  | **<0.001** |
| DS vs rad. progr. | 56 vs 163 | 3.08 | 2.03 to 4.67 |  |
| Visual impairment vs rad. progr. | 150 vs 163 | 1.01 | 0.71 to 1.43 |  |
| Neurol. symptoms vs rad. progr. | 121 vs 163 | 1.16 | 0.82 to 1.64 |  |
|  | | | | |
| **Tumor histology^b^** |  |  |  | **0.108** |
| PMA vs PA | 36 vs 283 | 1.44 | 0.92 to 2.24 |  |
| Diffuse glioma vs PA | 48 vs 283 | 1.58 | 1.03 to 2.43 |  |
| Glioneuronal tumors vs PA | 29 vs 283 | 1.51 | 0.89 to 2.58 |  |
| Other histology vs PA | 25 vs 283 | 1.63 | 0.98 to 2.73 |  |
| No histology vs PA | 69 vs 283 | 1.03 | 0.69 to 1.53 |  |
|  | | | | |
| **OVERALL SURVIVAL^a^** | n=497;  54 deaths |  |  |  |
| **Age group – Strata at randomization** |  |  |  | **0.002** |
| <1 vs 1 to <8 years | 72 vs 325 | 2.58 | 1.17 to 5.68 |  |
| >=8 vs 1 to <8 years | 94 vs 325 | 3.10 | 1.56 to 6.17 |  |
|  |  |  |  |  |
| **Indication to treatment** |  |  |  | **0.009** |
| DS vs rad. progr. | 57 vs 163 | 3.69 | 1.37 to 9.93 |  |
| Visual impairment vs rad. progr. | 150 vs 163 | 1.04 | 0.40 to 2.65 |  |
| Neurol. symptoms vs rad. progr. | 121 vs 163 | 2.02 | 0.92 to 4.45 |  |
|  |  |  |  |  |
| **Tumor histology** |  |  |  | **<0.001** |
| PMA vs PA | 36 vs 284 | 3.01 | 1.13 to 8.01 |  |
| Diffuse glioma vs PA | 48 vs 284 | 5.56 | 2.52 to 12.23 |  |
| Glioneuronal tumors vs PA | 29 vs 284 | 2.45 | 0.69 to 8.69 |  |
| Other histology vs PA | 25 vs 284 | 4.73 | 1.89 to 11.84 |  |
| No histology vs PA | 69 vs 284 | 2.60 | 1.09 to 6.19 |  |
|  | | | | |
| * For pairwise comparisons, confidence intervals instead of p values are given (P value of Wald test ≤0.05 if and only if confidence interval does not contain 1.0).  ^a^ Missing values to add up to 497: 7/6 patients with missing information regarding PFS/OS.  ^b^ Interaction terms are selected in a second step with previously selected main effects remaining in the model. | | | | |
|  | | | | |
| **Overall survival (OS) and progression-free survival (PFS) calculated from date of response assessment at week 24.** | | | | |
| **PROGRESSION-FREE SURVIVAL^c^** | n=381;  177 events |  |  |  |
| **Metastases** |  |  |  | **0.002** |
| Yes vs no | 52 vs 329 | 1.88 | 1.11 to 2.55 |  |
|  | | | | |
| **Indication to treatment** |  |  |  | **0.015** |
| DS vs rad. progr. | 28 vs 138 | 2.26 | 1.38 to 3.72 |  |
| Visual impairment vs rad. progr. | 125 vs 133 | 1.19 | 0.83 to 1.70 |  |
| Neurol. symptoms vs rad. progr. | 90 vs 133 | 0.95 | 0.63 to 1.44 |  |
|  | | | | |
| **OVERALL SURVIVAL^c,d^** | n=426;  33 deaths |  |  |  |
| **Tumor histology** |  |  |  | **0.004** |
| PMA vs PA | 29 vs 249 | 1.84 | 0.40 to 8.44 |  |
| Diffuse glioma vs PA | 38 vs 249 | 4.83 | 1.88 to 12.39 |  |
| Glioneuronal tumors vs PA | 26 vs 249 | 1.60 | 0.35 to 7.35 |  |
| Other histology vs PA | 19 vs 249 | 8.16 | 2.74 to 24.33 |  |
| No histology vs PA | 55 vs 249 | 2.19 | 0.79 to 6.08 |  |
|  | | | | |
| **Response at week 24** |  |  |  | **<0.001** |
| PD vs CR/PR/OR/SD | 32 vs 384 | 16.96 | 8.21 to 35.07 |  |
|  | | | | |
| * For pairwise comparisons, confidence intervals instead of p values are given (p value of Wald test ≤0.05 if and only if confidence interval does not contain 1.0).  ^c^ For PFS/OS: Patients with non-progressive disease/alive at week 24.  ^d^ Missing values to add up to 426: Response at week 24: 10 patients alive at week 24 with missing information. | | | | |
