# Supplementary material for: A European randomised controlled trial of the addition of etoposide to standard vincristine and carboplatin induction as part of an 18-month treatment programme for childhood (≤16 years) low grade glioma – A final report
Source: Eur J Cancer. 2017 Aug;81:206–25. doi: 10.1016/j.ejca.2017.04.019 (PMC5517338; doi:10.1016/j.ejca.2017.04.019)
Supplement: Supplementary file 2 [file mmc2.zip › Master Protokoll - Version 2010.pdf]

International Consortium on Low Grade Glioma - ICLGG
of the International Society of Pediatric Oncology - SIOP

Cooperative multicenter Study for Children and Adolescents with Low Grade Glioma

SIOP - LGG 2004

Version I, April 2004

Corrections from:
July 2004
January 2006
January 2010

Vertraulichkeitshinweis nach GCP: Dieses Protokoll ist vertraulich. Eine Weitergabe ist weder in schriftlicher noch in elektronischer Form ohne schriftliche Registrierung und Einwilligung der Studienzentrale zulässig.

EudraCT - Nr: 2005-005377-29
NCI-PDQ Database ID Code: SIOP-LGG 2004 EU-20555

gefördert durch:

Deutsche Kinderkrebsstiftung
Adenauerallee
53113 Bonn
www. Kinderkrebsstiftung. de

Cooperative multicenter Study for Children and Adolescents with low grade glioma
SIOP - LGG 2004

Participating Societies:

Gesellschaft für Pädiatrische Onkologie und Hämatologie – GPOH, Germany
Gesellschaft für Pädiatrische Onkologie und Hämatologie – GPOH, Austria
Associazione Italiana Ematologia e Oncologia Pediatrica - AIEOP
United Kingdom Children's Cancer Study Group – UKCCSG
Société Francaise des Cancers d'Enfants - SFCE
Sociedad Espanola de Oncologìa Pediátrica – SEOP
Nordic Organisation of Pediatric Hematology and Oncology – NOPHO
Belgian Society of Pediatric Hemato-Oncology - BSPHO
Dutch Society of Pediatric Hemato-Oncology - DPOH

Protocol activated	01.04.2004
Recruitment phase	01.04.2004 – 31.03.2010, prolonged to 31.03.2012
Observation phase	01.04.2012 – 31.03.2014

EudraCT - Nr: 2005-005377-29

NCI - PDQ Database ID code: SIOP-LGG 2004 EU20555

SIOP-Statement for the SIOP-LGG 2004 Cooperative multicenter Study for Children and Adolescents with Low Grade Glioma

The Scientific Committee of SIOP has reviewed this protocol for scientific validity and has deemed the hypotheses being addressed are scientifically valid. However, SIOP is not the sponsor, as defined by the ICH Harmonised Tripartite Guidelines, of this study, and accepts no legal responsibility for the conduct of this study. In addition, neither the Board nor the Scientific Committee of SIOP accepts responsibility for the overall conduct of this study and has specifically pointed out that implementation of this study requires the approval of the Research Ethics Committee/Institutional Review Board of each participating institution. The responsibility for the management of any individual patient treated with this protocol rests with the treating physician.

The protocol is compiled from contributions of members of the International Consortium on low grade glioma of the SIOP. The master-protocol has been written in Augsburg with the secretarial assistance of Silvia Soellner.

I. International Study Committee SIOP LGG 2004

PEDIATRIC ONCOLOGY
Germany – GPOH: Astrid K. Gnekow
Austria – GPOH: Irene Slavc
Italy – AIEOP: Giorgio Perilongo
UK – CCSG: Sue Picton, David Walker
Nordic Countries - NOPHO: Tore Stokland, Per Erik Sandstrom, Niels Clausen, Mikko Arola, Olafur Gisli Jonsson
Spain - SEOP: Ofelia Cruz, Aurora Navajas, Anna Teijeiro
The Netherlands – Dutch Pediatric Oncology Group: Antoinette Schouten-van Meeteren
France – SFCE: Jacques Grill, Chantal Kalifa, Marie-Anne Raquin
Belgium - BSPHO: Joris Verlooy

PATHOLOGY AND MOLECULAR BIOLOGY
Germany – GPOH: Volkmar Hans, Torsten Pietsch, Wolfram Scheurlen
Austria – GPOH: Johannes Hainfellner
Italy – AIEOP: Felice Giangaspero
UK – CCSG: James Ironside, Keith Robson
Nordic Countries - NOPHO: Kari Skullerud, David Scheie
Spain - SEOP: NN
France – SFCE: Marie-Madeleine Ruchoux, Anne Jouvet, Dominique Figarella-Branger, Arielle Lellouch-Toubiana
Belgium -

RADIOLOGY
Germany – GPOH: Monika Warmuth-Metz
Austria – GPOH: Daniela Prayer
Italy – AIEOP: Milena Calderone
UK – CCSG: Tim Jaspan
Nordic Countries – NOPHO: Soren Jacob Bakke
Spain – SEOP: Eli Vazquez
France – SFCE: Dominique Couanet
Belgium -

RADIOTHERAPY
Germany – GPOH: Rolf D. Kortmann
Austria – GPOH: Karin Diekmann
Italy – AIEOP: Giovanni Scarzello
UK – CCSG: Roger Taylor
Nordic Countries – NOPHO: Knut Lote
Spain – SEOP: Jordi Giralt
France – SFCE: Christian Carrie, Jean Louis Habrand
Belgium -

NEUROSURGERY
Germany – GPOH: Niels Soerensen
Austria – GPOH: Thomas Czech

Italy – AIEOP: NN
UK – CCSG: Paul Chumas
Nordic Countries – NOPHO: Bengt Gustavson
Spain – SEOP: NN
France – SFCE: Michel Zerah
Belgium -

OPHTHALMOLOGY
Germany – GPOH: Bettina Wabbels
Austria – GPOH: NN
Italy – AIEOP: Maria Luisa Pinello
UK – CCSG: Alistair Fielder, Ian Simmons
Nordic Countries – NOPHO: Terje Christoffersen
Spain – SEOP: NN
France – SFCE: NN
Belgium -

NEUROPEDIATRICS, HEALTH STATUS /QUALITY OF LIFE
Germany – GPOH: Gabriele Calaminus, Knut Brockmann, Ronald Straeter, Friedrich Ebinger, Pablo Hernaiz-Driever
Austria – GPOH: Herwig Lackner
Italy – AIEOP: NN
UK - CCSG: Colin Kennedy, Adam Glaser
Nordic Countries – NOPHO: Bo Stromberg
Spain SEOP: Jose Mª Indiano
France – SFCE: Chantal Rodary
Belgium -

ASSOCIATED RESEARCH, PHASE II – STUDIES
Eric Bouffet, Toronto, Canada
Didier Frappaz, Lyon, France
Belgium -

BIOSTATISTICS
Germany - GPOH: Andreas Faldum, Angela Emser
Italy - AIEOP: Gian Luca De Salvo
UK - UKCCSG: Claire Weston, David Machin
France - SFCE: Marie-Cécile Le Deley
Nordic Countries – NOPHO: Thore Egeland
Belgium -

DATA MONITORING AND SAFETY COMMITTEE
Carolyn Freemann, Montreal, Canada
Martin Schrappe, Kiel, Germany
Richard Sposto, Arcadia, Ca., USA

II. Signatures SIOP-LGG 2004
Trial Management Committee

Date: 30. 04. 2004

Gnekow, Astrid, GPOH

03.05.04

Grill, Jacques, SFCE

Date: 29/04/04

Perilongo, Giorgio, AIEOP

29-4-2004

Sandstrom, Per-Erik

Date: 29.04.04

Stokland, Tore

29 April 2004

Walker, David, UK CCSG

Date: 29.4.04

Kortmann, Rolf D., GPOH

19.05.04

Scarzello, Giovanni, AIEOP

Date: 30.4.04

Taylor, Roger, CCSG

19.05.04

De Salvo, Gian Luca, AIEOP

5.5.04

Faldum, Andreas, GPOH

III. Important Note SIOP LGG 2004

WITH THIS PROTOCOL THE INTERNATIONAL CONSORTIUM ON LOW GRADE GLIOMA RESEARCH PRESENTS THE SECOND TRIAL FOR THE TREATMENT OF LOW GRADE GLIOMA IN CHILDREN AND ADOLESCENTS.

The consortium emphasizes that even following approval from the national and/or local ethics committees no legal responsibility for possible consequences resulting from the application of recommendations from this protocol will be taken by the members of the consortium. Treatment and follow-up of patients with low grade glioma requires a high degree of medical competence and humane presence existing only in hospitals with adequate infra-structure. A state of emergency due to complications from the underlying disease or from its treatment can develop in every patient at any time and may require all resources mentioned. In such circumstances increased efforts can not compensate for a lack of experience. Children with brain tumors – even when of "low" grade malignancy only – should thus be treated by an experienced team and interdisciplinary cooperation is a prerequisite for such a team comprising neurosurgeons, neuropathologists, neuroradiologists, radiotherapists, ophthalmologists and pediatricians. Sufficient experience concerning the treatment of pediatric brain tumors and of extracranial malignant tumors in cooperative multicenter trials is implied, as well.

The protocol describes a multicenter study for the treatment of pediatric brain tumors of low grade malignancy in children and adolescents. It contains information regarding registration to the study. The protocol was not written for patients who do not participate in this study. Possible changes or amendments to the protocol will be communicated to participating institutions. Additionally, participating centers are requested to ensure validity and actuality of their available protocols regularly. Before entering patients into the study institutions have to obtain ethical approval of the protocol according to local regulations.

This concerted research action is run by the International Consortium on Childhood LGG which represents the LGG strategy group of the Brain Tumor Sub-Committee of the International Society of Pediatric Oncology (SIOP). This is the second generation of clinical trials run by this consortium.
The contribution of the major European Pediatric Neuro-oncology Groups – e.g. the ones from France, Germany, Italy, the Scandinavian countries, Spain and the United Kingdom – to the study has made possible to conceive a prospective randomised trial as part of the protocol. This is the first prospective randomised trial ever run in Europe on childhood LGG and the second in the world.

IV. Table of contents SIOP LGG 2004

I	INTERNATIONAL STUDY COMMITTEE	3
II	SIGNATURES	5
III	IMPORTANT NOTE	6
IV	TABLE OF CONTENTS	7
V	LIST OF ABBREVIATIONS	11
1.	PREAMBLE	13
2.	SUMMARY	14
2.1.	Flow diagram of the study	14
2.2.	Flow diagram for investigation and treatment (chemotherapy arm)	15
2.3.	Key information on the SIOP - LGG 2004 Study	16
3.	INTRODUCTION, BACKGROUND AND RATIONALE	24
3.1.	Introduction	24
3.2.	Background	27
3.2.1.	Surgery	27
3.2.2.	Radiotherapy	27
3.3.3.	Chemotherapy	29
3.3.	Rationale for the study design of SIOP - LGG 2004	38
3.3.1.	Effective drugs in the treatment of low grade glioma	38
3.3.2.	Rationale for the intensification of induction treatment	41
3.3.3.	Rationale for the differentiation of consolidation treatment	42
3.3.4.	Safety considerations for the choice of drugs	43
3.3.5.	Rationale for a „chemotherapy only" schedule in patients with Neurofibromatosis NF I	45
4.	RESULTS OF SIOP-LGG 1	47
4.1.	Study design of SIOP-LGG 1	47
4.2.	Chemotherapy part of the study SIOP-LGG 1	48
4.2.1.	Patients accrual, time of treatment, clinical characteristics	48
4.2.2.	Results	49
4.2.3.	Toxicity	55
4.3.	Main conclusions	56
5.	CHANGES WITHIN SIOP - LGG 2004 AS COMPARED TO SIOP - LGG 1	57
6.	AIMS OF THE STUDY	59
7.	STUDY QUESTIONS	61
8.	INVESTIGATIONS AT DIAGNOSIS AND DURING FOLLOW-UP	62
8.1.	Primary tumor diagnosis	62
8.2.	Postoperative diagnostic procedures	63
8.3.	Histopathologic diagnosis	64

8.4.	Status assessment during therapy and follow-up	67
8.5.	Guidelines for neuroradiologic assessment	71
8.6.	Ophthalmological assessment	75
8.7.	Health status and Quality of life assessment	79
9.	PATIENT ELIGIBILITY	80
9.1.	Inclusion criteria	80
9.2.	Exclusion criteria	81
10.	INDICATION FOR NON-SURGICAL THERAPY	82
11.	PATIENT REGISTRATION AND RANDOMIZATION	85
12.	OVERALL STUDY DESIGN	87
12.1.	Study I: LGG of the Supratentorial Midline, non-NF I	90
12.2.	Study II: LGG of all other sites, non-NF I	96
12.3.	Study III: LGG of all sites in children affected by NF I	104
12.4.	Disseminated low grade glioma	108
13.	SURGERY AT DIAGNOSIS OR DURING FOLLOW-UP	110
13.1.	LGG of the supratentorial midline, non-NF I	110
13.2	LGG of all other sites, non-NF I	111
13.3.	LGG of all sites in children affected by NF I	112
14.	CHEMOTHERAPY	114
14.1.	Chemotherapy guidelines	115
14.1.1.	Induction	116
14.1.2.	Consolidation	118
14.1.3.	Alternatives for consolidation in case of allergy or progression	119
14.1.4.	Cumulative drug doses	120
14.2.	Drug information	121
14.2.1.	General guidelines for dosing and application	121
14.2.2.	Effects and side effects of drugs used in this protocol	123
14.2.3.	Toxicity and dose modifications	127
14.2.4.	Specific organ toxicities	128
14.2.5.	Allergy to Carboplatin	129
14.3.	Supportive care	131
15.	RADIOTHERAPY	133
15.1.	Introduction and background	134
15.1.1.	Role of radiation therapy	134
15.1.2.	Timing of postoperative radiotherapy	137
15.1.3.	Dose response effects	138
15.1.4.	Tumor volume response to radiotherapy	139
15.1.5.	Treatment fields	141
15.1.6.	Monitoring of integral dose to tumor and organs at risk	142
15.1.7.	Low grade glioma of the spinal cord	143
15.1.8.	Conclusion	144
15.2.	Aims of the radiotherapy protocol	145
15.2.1.	Rationale to maintain dose prescription	145
15.2.2.	Rationale to introduce modern treatment techniques	145

15.2.3.	Rationale for monitoring of integral dose to tumor and organs at risk	146
15.2.4.	Rationale to monitor tumor response to radiotherapy	146
15.2.5.	Rationale to perform craniospinal irradiation in disseminated disease	146
15.2.6.	Rationale for brachytherapy	146
15.3.	End points of the trial	147
15.4.	Eligibility criteria for radiotherapy	148
15.5.	Specific technical outlines for radiotherapy	149
15.5.1.	Pretherapeutic imaging	149
15.5.2.	Treatment technique / intracranial and spinal sites	149
15.5.3.	Target volumes	149
15.5.4.	Dose specification	149
15.5.5.	Dose prescription	150
15.5.6.	Patient positioning	150
15.5.7.	Cranio-spinal irradiation	150
15.5.8.	Documentation	152
15.5.9.	Acute treatment related toxicities	153
15.5.10.	Routine laboratory tests during radiotherapy	153
16.	DEFINITIONS FOR TUMOR STAGING AND EVALUATION OF RESPONSE AND REMISSION	154
16.1.	Tumor staging	154
16.2.	Extent of resection	155
16.3.	Evaluation of response and remission	156
16.3.1.	General assessment of response	156
16.3.2.	Criteria of neuroradiologic response of primary tumor and of disseminated lesions	157
16.3.3.	Considerations for the neuroradiological assessment of response	158
16.4.	Serious adverse event	159
17.	STATISTICS	161
17.1.	Chemotherapy group	161
17.1.1.	Low grade glioma of all sites in children not affected by Neurofibromatosis NF I	161
17.1.2.	Low grade glioma of all sites in children affected by Neurofibromatosis NF I	168
17.2.	Radiotherapy group	171
17.2.1.	Low grade glioma of all sites in children not affected by Neurofibromatosis NF I	171
18.	ORGANISATIONAL AND ADMINISTRATIVE ISSUES	178
19.	ASSOCIATED RESEARCH	183
20.	LITERATURE	184
21.	ADDENDUM (INTERNATIONAL TRIAL)	203
21.1	Document of approval from the Ethics Committee	205
21.2.	Declaration of center participation	206
21.3.	Patient Information	207

21.4.	Consent forms	207
21.5.	Registration forms	208
21.6.	Randomisation forms	212
21.7.	Administration of chemotherapy	214
21.8.	Documentation of chemotherapy	220
21.9.	Documentation of radiotherapy	230
21.10.	Histopathology forms	241
21.11.	Common Toxicity Criteria (CTC)	242
21.12.	Report of serious adverse events (SAE)	246
21.13.	Follow-up forms	247
21.14.	List of participating centers	262
22.	ADDENDUM - NATIONAL PART FOR GERMANY AND AUSTRIA	263
	Part I: National study committee and organisation	263
	SIOP LGG 2004: National study committee	264
	Flow chart therapy	266
	Summary	267
	Signatures	277
	Part II: Addendum 21. specified for national requirements	278
22.1	Document of approval from the Ethics Committee	280
22.2.	Declaration of center participation	283
22.3.	Patient Information	284
22.4.	Consent forms	293
22.5.	Registration forms	298
22.6.	Randomisation forms	302
22.7.	Administration of chemotherapy	304
22.8.	Documentation of chemotherapy	310
22.9.	Documentation of radiotherapy	320
22.10.	Forms for central review: Neuropathology and MRI	331
22.11.	Common Toxicity Criteria (CTC)	339
22.12.	Report of serious adverse events (SAE)	342
22.13.	Follow-up forms	343
22.14.	Associated Research	362
22.15.	List of participating centers	370

V. Abbreviations SIOP LGG 2004

ACTH	Corticotropin
ADH	Antidiuretic hormone
AIEOP	Associazione Italiana Ematologia e Oncologia Pediatrica
AML	Acute myeloic leukemia
BEAR	Brainstem evoked auditory response
BSA	Body surface area
Carbo	Carboplatin
CCSG	Children's Cancer Study Group (USA)
CDDP	Cisplatin
CI	Confidence Interval
CMV	Cytomegalovirus
CNS	Central nervous system
CR	Complete remission and/or response
CSF	Cerebrospinal fluid
CS-RT	Cranio-spinal radiotherapy
CT	Chemotherapy
CTC	Common toxicity criteria
CT-scan	Computer tomography
CTV	Clinical target volume
DIGG/DIA	Desmoplastic infantile ganglioglioma /-astrocytoma
DLGG	Disseminated low grade glioma
DS	Diencephalic syndrome
EEG	Electro-encephalo-gramm
EFS	Event free survival
EORTC	European Organisation for Research and Treatment of Cancer
FSH	Follicle stimulating hormone
F-U	Follow-up
GCS	Glasgow coma scale
G-CSF	Granulocyte-Colony stimulating factor
GFR	Glomerular filtration rate
GH	Growth hormone
GnRH	Gonadotrophin releasing hormone (= LHRH)
GPOH	(German and Austrian) Society of Pediatric Oncology and Hematology
Gy	Gray
HCG	Hypothalamic-chiasmatic glioma
HS	Health status
HUI	Health utility index
iv	Intra-venous
ICD-O	International classification of diseases - Oncology
ICRU	International Commission for Radiation Units, Washington, D. C.
IDMC	International Data Monitoring Committe
IGF BP 3	Insulin-like growth factor binding protein 3
IGF I	Insulin-like growth factor 1
JPA	Juvenile pilocytic astrocytoma
LGG	Low grade glioma

LH	Luteinising releasing hormone
MDS	Myelodysplastic syndrome
MR	Minor response
MR(I)	Magnetic resonance imaging, magnetic resonance tomography
NCI	National Cancer Institute
NF I	Neurofibromatosis type NF I
NOPHO	Nordic Organisation of Pediatric Hematology and Oncology
OAR	Organs at risk
OP	Operation, surgery
OPG	Optic pathway glioma
OR	Objective response
OS	Overall survival
p.o.	per os
PA	Pilocytic astrocytoma
PD	Progressive disease
PF	Posterior fossa
PFS	Progression free survival
PNET	Primitive neuroectodermal tumor
POG	Pediatric Oncology Group
PR	Partial remission and/or response
PTV	Planning target volume
Qol	Quality of life
R	Randomisation
RDE	Remote data entry
RFS	Radiotherapy free survival
RT	Radiotherapy
s.c.	Subcutaneously
SAE	Severe adverse event
SD	Stable disease
SDQ	Strengths and difficulties questionnaire
SEOP	Sociedad Espanola de Oncología Pediátrica
SFCE	Societé Francaise Cancers Enfants
SFOP	Societé Francaise d'Oncologie Pediatrique
SIADH	Syndrome of inadequate secretion of ADH
SIOP	International Society of Pediatric Oncology
SMN	Second malignant neoplasm
SSD	Source to skin distance
TPDCV	Thioguanin-Procarbazin-Dibromodulcitol-CCNU-Vincristin
TR	Tumor response
TRE	Tumor related event
TSH	Thyroid stimulating hormone
UK-CCSG	United Kingdom Children's Cancer Study Group
VCR	Vincristin
VEP	Visual evoked potential
VP 16	Etoposide
WHO°	Degree of malignancy according to world health organisation grading

1. Preamble SIOP LGG 2004

The protocol SIOP - LGG 2004 attempts to offer a comprehensive treatment strategy to all children and adolescents up to an age of 18 years, who are affected by a low grade glioma arising in any part of the central nervous system. Results of the preceding SIOP - LGG trial as well as results from national trials and reports in the literature form the basis of the recommendations and the randomized part(s) of the study.

Considering tumor location and the absence or presence of the associated genetic disorder Neurofibromatosis (NF I) patients are divided into three strategic groups. Within each group the extent of primary resection, the presence or absence of severe neurologic symptoms and the presence or absence of tumor progression determines whether children are to be observed following diagnosis and resection or treated with either chemo- or radiotherapy. Thus, there are basically 9 distinct groups of patients. Differences between histologic entities among the totality of low grade glial tumors and their biologic behavior in different regions of the brain may add to the complexity of the treatment recommendations.

The study committee of the International Consortium of low grade glioma Research has recognized and accepted this complexity within the protocol, which allows a largely individualized therapy within a structured framework and offers the most up-to-date diagnostic and therapeutic approaches for the participating countries. By this, the committee hopes to meet the expectations of the study groups, pediatric cancer treatment centers and the patients and their families.

Scientific questions can only be posed and, hopefully, answered for the largest subgroups of patients, although recruitment rates for these patient groups can only be estimated at the time of writing. Thus a randomized therapy optimizing study is proposed for children not affected by NF I with supratentorial midline tumors. Study arms for all the other groups will undergo descriptive evaluation.

Most subgroups of patients with low grade glial tumors already have an excellent prognosis. The study is designed to improve the level of progression free survival for those children with the poorer long term prognosis. Yet the nature of low grade glial tumors makes it pertinent to not only evaluate short term survival, but to focus on ophthalmologic, neuroendocrine, and quality of life outcome as well. This study aims to investigate more closely into these outcome measures, in order to develop detailed recommendations for such follow-up, which in our view is indispensable for optimal patient rehabilitation.

2. Summary SIOP LGG 2004

2.1. Flow diagram of the study

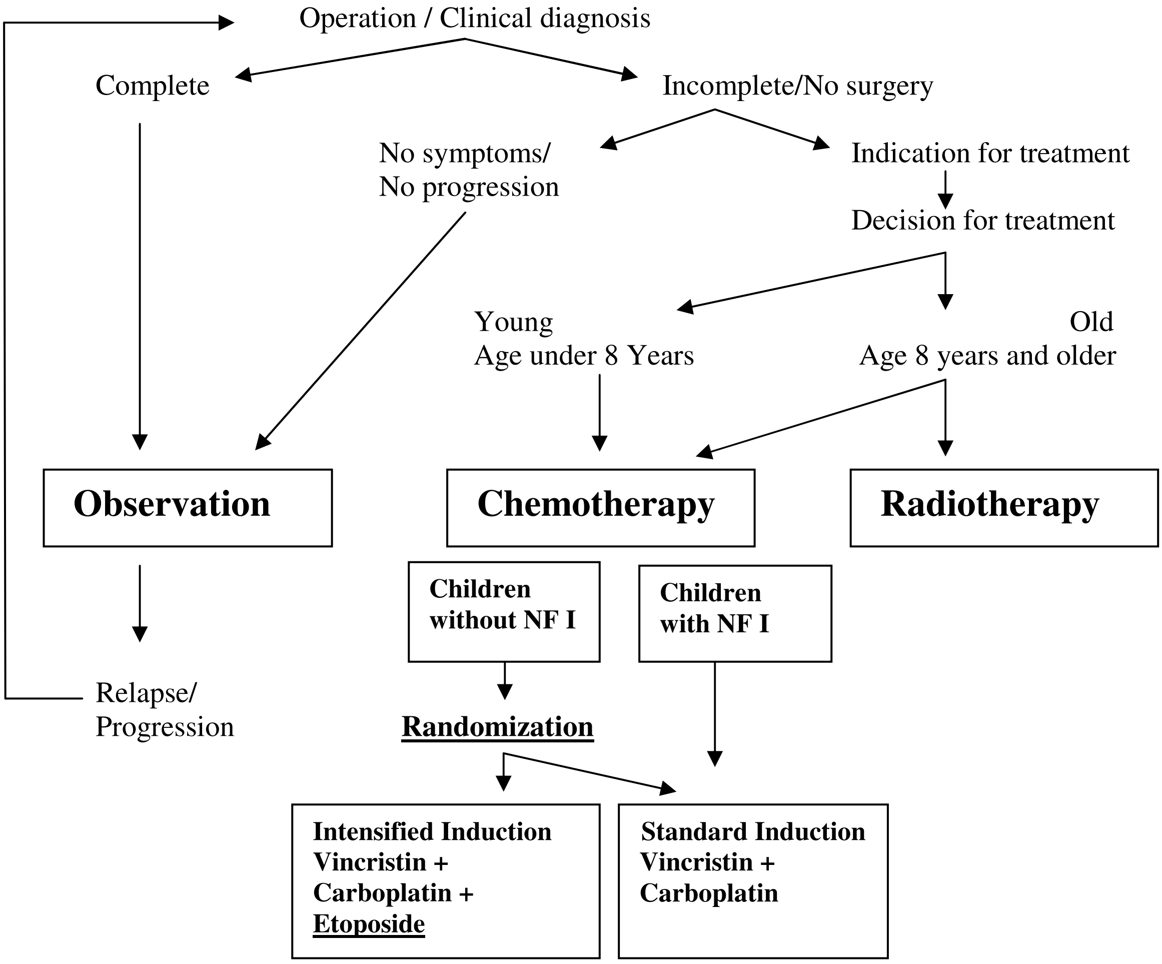

The study SIOP-LGG 2004 offers a common therapy strategy for all children and adolescents with a histologically (WHO criteria) or radiologically confirmed low grade glioma. Following complete resection patients will only be observed, as will be patients without symptoms or progression after incomplete resection or clinical diagnosis. Non-surgical therapy will be instituted at the presence of defined indications following incomplete resection, non-resectable relapse or progression of an unresectable tumor.

Older children (≥ 8 years) receive primary radiotherapy. Modern planning and treatment techniques shall reduce long term side effects upon surrounding tissues and organs at risk. At the presence of specific conditions these children may receive chemotherapy as well. The indication for interstitial radiotherapy is not age restricted. Younger children (< 8 years) receive primary chemotherapy. Children affected by Neurofibromatosis NF I shall be treated with chemotherapy at all ages. The duration of chemotherapy is 18 months. Children without NF I (stratified for age and tumor localization) will be randomized to receive standard induction with Vincristin and Carboplatin or intensified induction with Vincristin, Carboplatin and Etoposide, to test, if there is a difference in progression free survival. Additionally the distribution of tumor response at week 24 shall be investigated. Consolidation consists of ten 6-week cycles of Vincristin/Carboplatin therapy. For all children overall survival, progression free and event free survival will be calculated. The influence of clinical and histologic findings upon these parameters will be investigated. The extent of late effects of primary tumor and therapy shall be documented prospectively.

2.2. Flow diagram for investigation and treatment – Chemotherapy arm

Diagnosis → **Strati-fication** → **Randomized Trial: Chemotherapy** / **Radio-therapy**

Investigations:
Neuroimaging: Chemo- and Radiotherapy group

Time point	**1**	**2**		**3**		**4**		**5**	**6**
**Histology** (if obtained)	**X**	**(X)**							
**Ophthalmology**	**X**	**X**	**X**	**X**	**X**	**X**	**X**	**X**	**X**
**Endocrinology**	**X**	**X**		**X**		**(X)**		**X**	**X**
**Patient status**	**X**	**X**	**X**	**X**	**X**	**X**	**X**	**X**	**X**
Months after start of treatment		0	3	6	9	12	15	18	follow-up

Randomisation (at month 0)

Basic treatment data (at month 0)

Chemotherapy trial: Central Response assessment week 24 (at month 6)

Documentation:

Basic patient data
Central pathologic review and/or
Central neuroradiologic review
Ophthalmology
Neurology
Endocrinology
Education

Chemotherapy documentation

Radiotherapy documentation

Documentation during treatment: neuroimaging, ophthalmology, neurology, endocrinology, status
At month: (3) **6** (9) **12** (15) **18**

Patient status report

If applicable:
Event report form

Post treatment:
Ophthalmology
Neurology
Endocrinology
Education
Late effects

2.3. Key information on the SIOP - LGG 2004 Study

Start of the main phase: 01.04.2004
Prospective end of patient recruitment: 31.03.2010, prolonged to 31.03.2012
Prospective end of the study: 31.03.2014

EudraCT - NR: 2005-005377-29, NCI-PDQ Database ID: SIOP-LGG 2004 EU-20555

1. Organisation

1.1. Title of the study: SIOP - LGG 2004 - Cooperative multicenter Study for Children and Adolescents with low grade glioma

1.2. Trial Management Committee
Chemotherapy protocol:

Dr. Astrid K. Gnekow
I. Klinik fuer Kinder und Jugendliche, Klinikum Augsburg
Stenglinstrasse.4
D-86156 Augsburg, Germany

Dr. Giorgio Perilongo
Clinica di Oncoematologia Pediatrica e Centro Leucemie Infantili
Via Giustiniani 3
I-35128 Padova, Italy

Dr. David A. Walker
Children's Brain Tumor Research Centre - Queen's Medical Centre
University of Nottingham
Nottingham, NG7 2UH, United Kingdom

Dr Jacques Grill
Département de Cancérologie de l'Enfant et de l'Adolescent
Institut Gustave Roussy
39 rue Camille Desmoulins,
F-94805 Villejuif, France

Radiotherapy protocol:

Dr. Roger E. Taylor
Department of Radiotherapy and Oncology - Cookridge Hospital
Hospital Lane
Leeds / Cookridge West Yorkshire LS16 6QB, UK

Dr. Rolf - D. Kortmann.
Department of Radiotherapy - University of Leipzig
Johannisallee 34
D - 04103 Leipzig, Germany

Dr. Giovanni Scarzello
Department of Radiotherapy - Padua General Hospital
Via Giustiniani 2
I-35100 Padua, Italy

Biostaticics:

Lead Data Analysis:
Dr. Andreas Faldum
Institut für Medizinische Biometrie,
Epidemiologie und Informatik
University of Mainz
D-55101 Mainz, Germany

Lead Data Management:
Dr. Gian Luca De Salvo
Clinical Trials & Biostatistic Unit
Istituto Oncologico Veneto
Busonera Hospital
Via Gattemelata 64
I-35128 Padova, Italy

National trial coordinators representating participating national oncology groups:

Austria: Irene Slavc, Vienna
France: Jacques Grill, Villejuif
Germany: Astrid K. Gnekow, Augsburg
Italy: Giorgio Perilongo, Padova
Norway: Tore Stokland, Tromsø
Spain: Ofelia Cruz, Bilbao
Sweden: Per Eric Sandstrom,
United Kingdom: Sue Picton, Leeds

International Data Center:

Clinical Trials & Biostatistic Unit
Istituto Oncologico Veneto
Busonera Hospital
Via Gattemelata 64
I-35128 Padova, Italy
Tel: 0039-049-8215704
Fax: 0039-049-8215706
email: siop-lgg2004@istitutoncologicoveneto.it

1.3. Primary study objectives (section 6 and 7)

1.3.1. Offer of a uniform, standardized concept for the treatment of children and adolescents affected by a low grade glioma.

1.3.2. Improvement of progression free survival following non-surgical therapy for children without NF I with low grade glioma by investigation of standardized treatment recommendations: Group 1: with tumors located in the supratentorial midline
Group 2: with tumors of the cerebral hemispheres, the cerebellum and caudal brain stem and the spinal cord

- Therapy arm: Radiotherapy
  Use of modern techniques for planning and treatment
- Therapy arm: Chemotherapy
  Prolongation of therapy for all children
  Randomized trial of intensification of induction therapy
  Common consolidation for all children with alternative in case of early progression or allergy.

1.3.3. Investigation of standardized treatment recommendations for non-surgical therapy for the study group of children with NF I and low grade glioma of all locations (Group 3).

1.3.4. Reduction of the rate and intensity of possible late effects of therapy:

- by sparing organs of risk through optimized planning and treatment of radiotherapy.
- by deferring the start of or avoiding radiotherapy for young children and children with Neurofibromatosis by choosing a chemotherapy strategy.

2. Eligibility criteria (Section 9.1.)

2.1. Age: children and adolescents up to age 18 years.
2.2. Histology: Glioma of low grade malignancy (ICD O-Code) [1]

Pilocytic Astrocytoma I°	9421/1
Subependymal Giant Cell Astrocytoma I°	9384/1
Dysembryoplastic Neuroepithelial Tumor I° 9413/0	
Desmoplastic Infantile Ganglioglioma I°	9412/1
Ganglioglioma I° and II°	9505/1
Pleomorphic Xanthoastrocytoma II°	9424/3
Oligodendroglioma II°	9450/3
Oligoastrocytoma II°	9382/3
Astrocytoma II°	9400/3
Fibrillary Astrocytoma II°	9420/3
Protoplasmatic Astrocytoma II°	9410/3
[illegible]	[illegible]

Within the randomized part of the study all histologies will be randomized, since up to now there are no data to exclude any of the subgroups, e.g. children with oligodendroglioma, from this study.
Specific neuroradiological criteria may allow to diagnose a low grade chiasmatic-hypothalamic tumor without biopsy (section 8.5.).
2.3. Primary tumor localization: intracranial and spinal cord.
2.4. Dissemination: Children presenting with disseminated low grade glioma will be eligible for the study.
2.5. Associated conditions: Children are eligible for the trial regardless of the presence of associated genetic disease
2.6. Primary tumor diagnosis: The tumor should not be pretreated with chemotherapy or radiotherapy.
2.7. Informed consent: The patient and/or his legal guardian (parents) have to have declared their written informed consent to the study.

Randomization: All eligible patients without Neurofibromatosis NF I receiving chemotherapy as their first non-surgical therapy are eligible for randomization.

3. Exclusion Criteria (section 9.2.)

3.1. Primary tumor localization: diffuse intrinsic tumors of the pons, even if histologically an Astrocytoma II° is diagnosed.
Exception: pontine glioma II° in NF I patients may be entered into the study.
3.2. Special diagnosis: Patients presenting with rare intracranial neoplasms of low grade malignancy, but non-glial origin. Their data may be registered however, to learn about those therapeutic interventions which may prove useful to these patients and to develop separate strategies in the future. Choroid plexus papilloma should be entered on the SIOP-CPT-study.

---
[1] ICD-Codes korrigiert (Juli 2004)

3.3. Pretreatment: Children treated with chemo- or radiotherapy prior to entering the study will be evaluated separately. Previous treatment with steroids is not considered a chemotherapeutic treatment.
3.4. Preexisting impairments of health status, making the conduct of the study impossible or ethically unwise.
3.5. Evidence of pregnancy or lactation period.

In case the patient participates in another clinical study simultaneously to being enrolled in the study SIOP-LGG 2004, but not interfering with the present treatment strategy (e.g. endocrinologic study), this should be known to the national study chairmen.

Concommittant medication for associated or other conditions (e.g. hormone replacement, anticonvulsants) should be recorded, but is no exclusion criteria.

4. Overview of protocol treatment (section 12.).

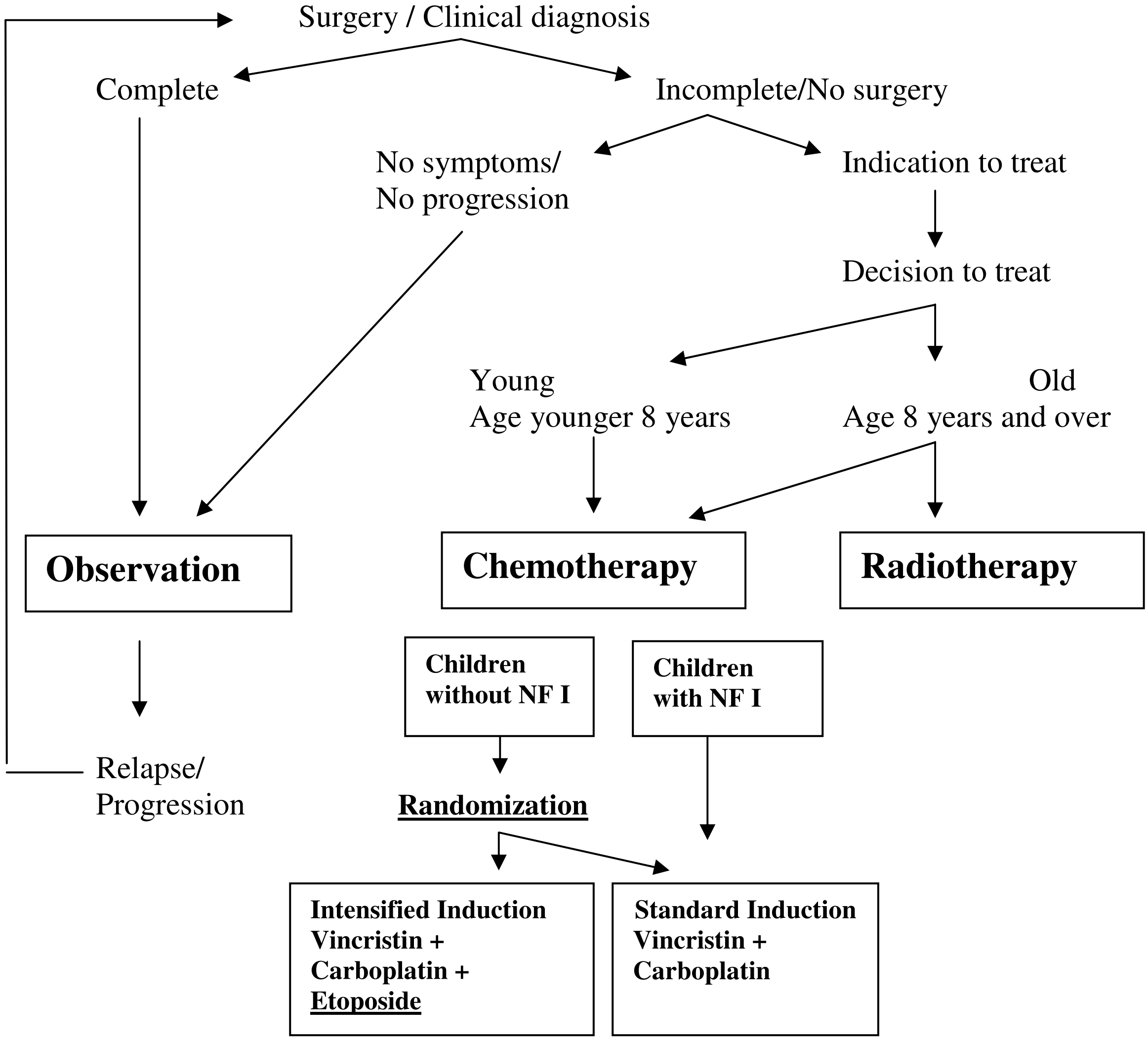

4.1. Basic Protocol Scheme

All patients with low grade glioma, eligible according to the criteria from section 9., should be entered into the current study and follow the same general strategy concerning non-surgical therapy. Dependent upon primary tumor localization and the

presence or absence of Neurofibromatosis NF I patients are devided into three study groups:
Group 1: Non-NF I, supratentorial midline (section 12.1.)
Group 2: Non-NF I, cerebral hemispheres, cerebellum, caudal brainstem, spinal cord, optic nerve (section 12.2.)
Group 3: NF I, all locations (section 12.3.)

4.2. Treatment Subgroups

The indication for non-surgical therapy in a patient with low grade glioma following diagnosis is based upon the extent of surgical resection, the presence or absence of severe neurologic symptoms and the presence or absence of clinical and/or neuroradiological progression during a period of observation. Within all study groups there are thus three "treatment" subgroups:

4.2.1. Observation group: Tumor completely resected
Tumor not or incompletely resected, no severe symptoms
Tumor not or incompletely resected, no progression
4.2.2. Treatment group at diagnosis: Severe neurologic symptoms
Severe ophthalmologic symptoms
4.2.3. Treatment group after observation: Progressive neurologic symptoms
Progressive ophthalmologic symptoms
Neuroradiologic progression, including dissemination

4.3. Stratification of non-surgical therapy

For each study group details for an age-related stratification of non-surgical treatment are provided:
Primary chemotherapy: "young" children, age < 8 years
All children with NF I
Primary Radiotherapy: "old" children, age ≥ 8 years
Children of all ages whose tumor is amenable to interstitial radiotherapy (brachytherapy)

4.4. Indication to start non-surgical therapy (section 10.)

Clinical and ophthalmological symptoms will be recorded and regular neuroradiologic assessment be made to decide following initial diagnosis whether there is an indication for non-surgical therapy.

4.4.1. Indication to start non-surgical therapy at diagnosis following subtotal or partial resection (S2 – S3)
Severe preexisting visual disturbance (section 8.6.)
Borderline vision in both eyes ("threat to vision")
Definite history of visual deterioration
Nystagmus due to poor vision (especially in infants up to two years indicative of visual disturbance)
Clinical indication
Diencephalic Syndrome
Symptomatic metastases
Note: The presence of a postoperative residual tumor is not an indication to therapy on its own.

4.4.2. Indication to start non-surgical therapy at diagnosis without prior tumor resection (following biopsy or radiological diagnosis)

Severe visual symptoms (section 8.6.)
- Borderline vision in both eyes ("threat to vision")
- Definite history of visual deterioration
- Nystagmus due to poor vision (especially in infants up to two years indicative of visual disturbance)

Severe neurologic symptoms
- Diencephalic syndrome
- Focal neurologic deficits secondary to tumor growth
- Symptoms of increased intracranial pressure secondary to tumor growth
- (Focal) Seizures secondary to tumor growth
- Symptomatic metastases

Note: The presence of a tumor is no indication to therapy on its own.

4.4.3. Indication to start non-surgical therapy following observation, if surgery is not feasible

Progressive neurologic symptoms
- Manifestation of new neurologic symptoms
- Manifestation of Diencephalic Syndrome

Progressive visual disturbances
- Reduction / loss of vision or of visual fields
- Any reduction / loss of vision in the second eye, if the other eye is blind

Neuroradiologic progression
- Definite increase of tumor size (Increase of the diameter of the optic nerve)
- Involvement of previously uninvolved areas of the brain
- Manifestation of tumor dissemination (including symptomatic or progressive metastases, symptomatic leptomeningeal dissemination)

4.5.Chemotherapy (section 14.)

4.5.1. Induction therapy

Induction treatment will be randomised between standard and intensified induction for study group 1 and 2 (No NF I, 1: supratentorial midline tumors, 2: LGG of all other locations).
Treatment group 3 (NF I, low grade glioma of any location) will receive standard induction.

<u>Standard Induction:</u>

1	2	3	4	5	6	7	8	9	10	13	17	21	24	week
**V**	**V**	**V**	**V**	**V**	**V**	**V**	**V**	**V**	**V**	**V**	**V**	**V**		
**C**			**C**			**C**			**C**	**C**	**C**	**C**		
													**MRI**	

<u>Intensified Induction</u>

1	2	3	4	5	6	7	8	9	10	13	17	21	24	week
**V**	**V**	**V**	**V**	**V**	**V**	**V**	**V**	**V**	**V**	**V**	**V**	**V**		
**C**			**C**			**C**			**C**	**C**	**C**	**C**		
**Ex3**			**Ex3**			**Ex3**			**Ex3**					
													**MRI**	

V	Vincristin	1,5 mg/m²	iv-bolus - d 1 of treatment week
C	Carboplatin	550 mg/m²	1h iv - d 1 of treatment week
E	Etoposide	100 mg/m²	1h iv - d 1 – 3 of treatment week

The evaluation at 24 weeks is decisional for entry into the consolidation therapy

4.5.2. Consolidation therapy
All patients will receive a common consolidation therapy:

25	31	37	43	49	week
55	61	67	73	79	
**VVV**	**VVV**	**VVV**	**VVV**	**VVV**	
**C**	**C**	**C**	**C**	**C**	

V	Vincristine	1,5 mg/m²	iv-bolus – d 1, 8, 15 of treatment cycle
C	Carboplatin	550 mg/m²	1h iv - d 1 of treatment week

4.5.3. Randomisation
Study group 1 and 2: Patients without NF I and LGG of the supratentorial midline, the cerebral hemispheres, the cerebellum, the caudal brain stem and the spinal cord will be randomised centrally between standard and intensified induction treatment. Randomisation will be stratified according to age (< 1 year, 1-8 years, ≥ 8 years) and primary tumor site (pure chiasmatic tumors (Dodge II), all other supratentorial midline tumors, tumors of all other sites outside the supratentorial midline).

Study group 3: Patients with NF I and tumors of any location will not be randomised, they receive standard induction therapy and consolidation.

4.6. Radiotherapy (section 15.)

Children receiving radiotherapy shall be treated according to modern treatment planning and application recommendations concerning fields and doses (total and per fraction). Stratification of age groups is identical to that for chemotherapy.

	Total dose	Dose per fraction	Treatment time
"Older" children: Brain	54 Gy	1,8 Gy	6 weeks
Spine	50,4 Gy	1,8 Gy	5 ½ weeks
"Young" children: Brain	Contact national radiotherapy chairman		
Spine			

5. Study end points

All study patients:	Feasibility of treatment<br>Overall survival, progression free survival following diagnosis
Observation group:	Long term sequelae, health status, quality of life
Treatment group:	Progression free survival, event free survival, overall survival<br>Response to non-surgical therapy<br>Long term sequelae, health status, quality of life

6. Statistical considerations

6.1. Children with LGG of all sites not affected by Neurofibromatosis NF I.

The aim of the trial is to compare **standard induction therapy** with Vincristine and Carboplatin with the **intensified induction therapy** with Vincristine, Carboplatin and

Etoposide with reference to progression free survival in children, who are not affected by Neurofibromatosis (type NF I), with low grade glioma of all sites necessitating chemotherapy as non-surgical therapy (according to patient eligibility criteria (section 9) and indication for non-surgical therapy (section 10)).

This therapy optimization trial is multi-national, multi-center, non-blinded, randomized and prospective.
The accrual period of the trial is prolonged beyond the initially intended 6 years for 2 additional years (8 years total) followed by an observation period of 2 years.

The main question (PFS) will be analyzed on a significance level of α=0,05. The p-values corresponding to the secondary questions are regarded as explorative.
Defined variables will be checked with reference to their influence upon the survival variables by Cox regression.

6.2. Children affected by Neurofibromatosis NF I with LGG of all sites.

Chemotherapy according to this protocol is applied to delay or obviate the start of radiotherapy compared with a historical control group.
Statistical analysis will be only descriptive.

3.1. Introduction SIOP LGG 2004

The clinically used term of low grade glioma confers to tumors of glial origin, usually astrocytic, but oligodendrocytic as well. Their histological grade corresponds to I° or II° according to the revised system of the WHO of 2000 (Kleihues 2000). For clinical purpose some of the mixed glioneuronal tumors are included as well, if their glial component appears most relevant for biologic behavior.

About 30 to 40 % of all pediatric primary brain tumors are low grade gliomas. Their annual incidence is calculated as 10-12 per 1 000 000 children under the age of 15 years in western countries (France, Germany, USA (white population), Scandinavian countries) (Stiller 1994, Kaatsch 2001, Schütz 2002). Childhood cancer registries assume a systematic underreporting of these neoplasms, which in part is due to the limited patient referral to centers of tertiary care (Michaelis 2000, Stiller 1994).

These tumors occur at all ages. Mean age of diagnosis or operation varies according to the selection of the pediatric cohort, but is mostly between 6 and 11 years. There is no general consensus concerning the impact of age on the risk of disease progression.

The male to female ratio can generally be viewed as 1,1-1,2:1 (Stiller 1994, Kaatsch 2001), although some diagnoses like the DIGG/DIA show a more marked male preponderance.

Associated Predisposing Conditions and Genetics

There is a striking association of specific variants of low grade glioma and heritable diseases, which in part may serve as a model for cancer development.

<u>Neurofibromatosis type I (NF I)</u> in its familial as well as in its sporadic form is caused by mutations within the Neurofibromin-gene, located on the long arm of chromosome 17 (17q 11.2). The NF I gene can primarily be regarded as a histogenesis control gene, which also functions as a tumor suppressor gene (Riccardi 2000). Yet, the occurrence of two independent mutations may not suffice to explain the development of low grade astrocytic lesions in NF I.
In as many as 5 to 15 % of cases (Riccardi 1992, Riccardi 1991, Lewis 1984, Listernick 1997) NF I is associated with low grade gliomas of the optic tract and the hypothalamus, but other regions of the brain as well (Vinchon 2000). The proportion of patients with NF I varies within neurooncological studies from 10 to 20 %, but may rise up to 60 %, if only visual pathway gliomas are considered (Capelli 1998, Castello 1998, Dutton 1994, Packer 1997). Since the presence of an optic pathway glioma puts NF I patients at risk for later development of other, even more malignant brain tumors, a subset of NF I patients may have an increased vulnerability for glial tumors. This may be caused by specific genetic mutations (Vinchon 2000, Friedman 1997) or by the effect of modifying genes, or from other modifying factors.

<u>Tuberous Sclerosis</u> complex is an autosomal-dominantly inherited multisystem disorder characterized by widespread hamartomas in almost every organ, but predominantly in brain, kidneys, liver, heart, skin and eyes. Molecular studies have shown mutations on chromosomes 16p13 and on 9q34. The presence of subependymal giant cell astrocytoma is one of the major

diagnostic criteria (Roach 1998). The tumors appear with increasing frequency throughout childhood reaching an incidence of 15 % in adolescence (Józwiak 2000).

Low grade astrocytoma is a trait of the Li-Fraumeni-syndrome as well, a genetic condition characterized by an excessive aggregation of tumors in more than two generations or in siblings, by the occurrence of tumors at an unusual age for the tumor type or in an atypical gender, as well as the sequential appearance of other cancers in the same individual, associated with genetic disorders and birth defects (Li 1982, Lynch 1985, Malkin 1990). A germline mutation in the p53 locus on chromosome 17p13 triggers the susceptibility to develop multiple tumors throughout life (Ohgaki 2000).

No prospective analysis of the prognostic significance of cytogenetic alterations has been performed. Despite repeated attempts of conventional karyotyping or of comparative genomic hybridisation, specific gene loci with frequent alterations could not be characterized in childhood low grade glioma as opposed to adult glioma, where progressive DNA-alterations within one given tumor representing progressive degrees of malignancy could be found (Miettinen 1999, Orr 2002, Smith 2000).
The association of NF I and juvenile pilocytic astrocytoma (JPA) WHO I° suggests a role for the (altered) NF I gene or its signal transduction pathway in the development of sporadic JPA as well, although this has not been proven yet by specific gene deletions or changes of gene expression. The occasional loss of chromosome 17q, including the region of the NF I gene, in sporadic pilocytic astrocytoma did not go along with specific mutations (von Deimling 1993, Ohgaki 1995). And even the differential expression of some NF I transcripts did not separate reactive and neoplastic astrocytes.

All types of low grade glioma are characterized by a biologically indolent growth pattern, not well explained by histological features. Only the newly characterized subtype of a pilomyxoid JPA seems to go along with an increased progression rate (Tihan 1999). Even after incomplete surgical resections some tumors do not exhibit a growth rate for extended periods of time. Alterations in blood supply, decelerating growth kinetics within the tumor over time due to a change in the ability of the tumor to maintain an adequate level of autocrine growth factors (like EGF-receptor, c-erbB-2 oncoprotein, TGF-alpha) or an increase in the spontaneous rate of apoptosis could contribute to the stable situation (Bodey 1999, von Bossany 1998, Rhodes 1998).
Conversely there are just speculations about factors responsible for tumor growth due to the lack of unequivocal findings concerning the role of the proliferation rate (Ki-67/MIB-1-staining), elevated levels of VEGF or a down-regulation of N-CAM, which may correlate with a higher rate of tumor progression (Abdulrauf 1998, Hoshi 1997, Sasaki 1998).

Natural History

Most children with low grade glioma will survive for long years, so analyzing overall survival (OS) as outcome parameter for the success of a given treatment strategy may not be the best way of discriminating treatment approaches. However, since long phases (10 to 15 years) of patient survival are common and the survivors will experience late effects of all treatments applied, it is pertinent to evaluate the additional damage produced by any therapeutic measure.

A substantial number of children will have recurrences following resection or experience progression following incomplete tumor removal or biopsy. Knowledge about the natural course of low grade gliomas is based on small series collected throughout long periods of

time. Since no clear-cut risk-profiles of either clinical, biologic or histopathologic features have been determined up to now, it cannot be predicted, which low grade tumors will show an indolent clinical behavior, and which will run an aggressive course. It is not known, whether all low grade tumors do possess a proliferative potential - therefore it is undetermined whether all low grade gliomas ultimately may need treatment. On the other hand, spontaneous involution has only rarely been documented unequivocally (Perilongo 1999, Kernan 1998). All of these tumors had been located in the chiasmatic-hypothalamic region. If reported, vision did not improve despite tumor shrinkage. Only for lesions in NF I patients tumor regrowth within the extended follow-up period has been reported (Schmandt 2000, Perilongo 1999).

Following numerous national and institutional trials with various treatment strategies for different subgroups of children with low grade glioma, this protocol aims to present an integrative approach for the treatment of all low grade gliomas irrespective of their location and histological subtype.

3.2. Background SIOP LGG 2004
Treatment strategies for low grade glioma

3.2.1. Surgery

There is general consensus that surgical excision should be considered first at diagnosis or at relapse. A variety of techniques can be used to optimise tumor location and complete resection (Berger 1994, Soo 2000, Pollack 1999).

Recent pediatric reports indicate that total removal is possible in up to 90 % of cerebral hemispheric glioma (Hirsch 1989), and in two thirds to 90 % of cerebellar astrocytoma (Smoots 1998, Due-Tonnessen 2002). Long term follow-up shows survival rates after complete resection above 90 % (Pollack 1995, Hirsch 1989, West 1995, Wallner 1988, Gjerris 1978 , Campbell 1996, Pencalet 1999).
But even in these cohorts a small percentage of progression occurs over time, necessitating further therapy.
Many tumors, however, are not amenable to complete resection either because of anatomical location or metastatic disease, and sometimes they only can be biopsied.
Stable disease for extended periods of time has been described following subtotal resection or less, yet historical data also demonstrate the impaired long term prognosis of 15 - 50 % survival after subtotal resection or less at various locations of the CNS, with a high rate of progression within the first years (Campbell 1996, Smoots 1998, Pencalet 1999, Garvey 1996, Hoffman 1993, Sutton 1995).
For cerebral hemispheric and cerebellar astrocytoma the volume of residual tumor proved to be the best predictor of the hazard of disease progression (Smoots 1998, Berger 1994). But irrespective of the tumor volume the probability of freedom from surgery or cytotoxic therapy after presentation is low for children with hypothalamic or visual pathway gliomas and dropped to 23 % at 2 and 19 % at 5 years for a series of 46 children (Janss 1995). The controversy concerning tumor management with radical or conservative surgery even for children with midline supratentorial glioma in order to reduce the rate of progression has to take postoperative functional status into consideration, as well (Wisoff 1990, Sutton 1995, Hoffman 1993).

Table 1 compiles the results of various neurosurgical reports. They demonstrate that following complete tumor resection relapse or progression are exceptional events, but that the extent of resection depends largely upon tumor location.

3.2.2. Radiotherapy

Introduction and the background concerning the role of radiotherapy in the treatment concept of low grade glioma are presented together with the rationale for the present study design and aims in Section 15.

Table 1: Results of the first neurosurgical intervention for children with low grade glioma

Author	Number of patients. Age	Localisation	Degree of resection	Results
Hirsch et al. 1989	42<br><br>median age:<br>4,25 y	Cerebral Hemispheres	Complete 40<br>incomplete 2<br>(2 irradiated)	1/40 Relapse<br>2/2 Progression<br><br>Probability of "Non-Recurrence"<br>95% at 5 years<br>78,5% at 12 years
Pollack et al. 1995	71	Cerebral Hemispheres	Complete 21<br>subtotal 12<br>partial 26<br>(>50% resected)<br>partial 12<br>(<50% resected)<br><br>(irradiated:<br>2/21 post CR<br>33/50 post <CR)	0/21 Relapse<br>2/12 Progression<br><br>11/38 Progression<br>additionally: 1/38 SMN<br><br>PFS for all Patients:<br>88% at 5 years<br>79% at 10 years<br>76% ar 20 years
Sutton et al. 1995	33<br><br>mean age<br>4,3/4,5 y	Chiasma/ Hypothalamus	Biopsy 27<br>(<20% resected)<br>subtotal 5<br>(20-50% resected)<br>No OP 1<br>(irradiated 29/33<br>Chemoth. 18/33<br>14/18 Chemo- and Radiotherapy)	Survival 28/33<br>(after a mean of 10,9 y)
Vandertop et al. 1992	12	Mesence-phalon	partial 9<br>no OP 3<br>(Radiation at Progression 3/12)	4/12 Progression<br>Survival: 100% at the time of the publication
Pollack et al. 1994	16	Mesence-phalon	No OP 13/16<br>Biopsy 3/16<br>at Progression:<br>Radiation 3/16	4/16 Progression<br>Survival: 100%
Reardon et al. 1998	24<br><br>median age<br>10 y.	Thalamus	"gross total" 4<br>"near total" 2<br>subtotal 2<br>Biopsy 16	All children monothal. bithal. tumor<br>Survival 52% 85% 0%<br>PFS 36% 58% 0%<br>at 4 years
Abdollazadeh et al. 1994	66<br><br>mean age<br>7,3 y	Cerebellum	Complete 61<br>Incomplete 5<br>(irradiated 1/5)	0/61 Relapse<br>5/5 Progression
Campbell et al. 1996	72<br><br>mean age:<br>6,5 y.	Cerebellum	total 57<br>subtotal 15	6/57 Relapse 57/57 Survival<br>7/15 Progression 14/15 Survival
Pencalet et al. 1999	168<br><br>mean age: 6,9 y.	Cerebellum	Complete 149 (88,7%)<br>Incomplete 19 (11,3%)<br>(irradiated 5/168 - 3%)	8/149 Relapse (5,4%)<br>8/19 Progression (42,1%)<br><br>Survival 95,8% at 7,7 y<br>PFS 95% after complete resection<br>45% after incomplete resection

Abbreviations: Degree of resection: CR – complete resection, OP: operation, SMN: second malignant neoplasm, PFS: progression free survival, Localisation: monothal.: monothalamic, bithal.: bithalamic

3.2.3. Chemotherapy

Investigation of chemotherapy treatment (CT) strategies initially focussed on young children under 5 years of age to avoid early radiotherapy (RT), especially for those with visual pathway gliomas. Early reports produced evidence that cytotoxic drugs are active against low grade astrocytic tumors and that they may delay or obviate the need for radiation therapy. Although short term efficacy with transient tumor control has been the primary target for these approaches, no data exist clarifying the role of chemotherapy for long-term outcome, yet. Preliminary reports suggest improvement or stabilisation of vision in children with optic pathway tumors even in the absence of objective tumor shrinkage (Mitchell 2001). Measuring response by conventional criteria like complete or partial response does not seem appropriate for low grade astrocytoma, a phase of prolonged stable disease is an adequate success of therapy (Packer 1997). Reports upon the effectiveness of chemotherapy in low grade glioma have comprised newly diagnosed as well as relapsed patients, treated with single agents or drug combinations for variable length of time.
The studies suggest that chemotherapy may have little or no significant adverse effects on cognitive or endocrine function, but their inherent long term risks concerning organ toxicity, carcinogenic and mutagenic risks have to be closely observed.

Following the termination of several larger, national studies, the role of chemotherapy, within a multidisciplinary approach, for the treatment of young children affected by a surgically unresectable, or progressive or symptomatic low grade glioma can now be considered firmly established in terms of achieving tumor responses including tumor volume reduction and a prolonged progression-free and radiation-free survival. The effects of chemotherapy on improving the actual clinical and neurological function, for example the visual and endocrinological function for supratentorial midline tumors, and ultimately on health status (HS) and quality of life (QoL), however, deserve further investigation.
It is difficult to compare the tumor response rates and ultimately long term results reported by the various trials run on childhood low grade glioma. Characteristics of the patient population, the indication to start therapy, the criteria defining response as well as the timing of tumor response assessment varied between the studies.

Table 2: Indication to therapy in various clinical trials:

Packer et al. 1997	• Within 4 weeks following documentation of clinical or radiographic tumor progression (> 25 % tumor volume)<br>• Within 4 weeks following initial resection of less than 50 % of tumor volume
Prados et al. 1997	• Progressive symptoms like visual loss, intracranial hypertension, obstructive hydrocephalus, endocrinopathy<br>• Radiographic tumor enlargement
Castello et al. 1998	• Incomplete tumor resection/biopsy (no biopsy required for large glioma of visual pathways and NF I)<br>• Severe and/or progressive neurologic symptoms
Laithier et al. 2000	• Newly diagnosed, progressive optic pathway gliomas
Perilongo et al. 2000	• Patients with severe neurologic symptoms at the time of diagnosis (e.g. diencephalic syndrome)<br>• Patients with progressive clinical symptoms and/or neuroradiologic progression following observation

3.2.3.1. The role of chemotherapy in terms of tumor response for low grade glioma

202 of the 204 eligible patients from the SIOP - LGG 1 study are presently evaluable for tumor response. The overall response rate, complete, partial and objective response and including stable disease, is 83.7%, with a complete (CR) and partial response (PR) rate of 50%. No central review of the actual MRI-scans to substantiate the data reported in the forms was carried out. The median time to tumor response evaluation was 3.6 months (range 1-21.5 months). It has not been investigated, if a more consistent timing of tumor response (TR) evaluation results in a different distribution of responses. The analysis of tumor response by clinical patients' characteristics such as age, sex, NF status, histology (astrocytoma nos., fibrillary astrocytoma, pilocytic astrocytoma and histology versus clinical diagnosis), site and the presence of disseminated disease did not reveal a significant influence.

The tumor responses reported by other major clinical trials such as the historical VCR/Actinomycin (Janss 1995) series from Philadelphia, the CCSG regimen, based on Vincristin/Carboplatin as well (Packer 1997), the San Francisco study using 6-Thioguanine, Procarbazine, Dibromodulcitol, Lomustine and Vincristin (Prados 1997) and the French "BB-SFOP"-trial, based on Carboplatin / Procarbazine; Cisplatin / VP 16; Vincristine / Cyclophosphamide (Laithier 2000, and personal communication), are reported below.

Table 3: – Tumor response in children with LGG as reported by the various chemotherapy trials

	Vincristin/ Carboplatin SIOP - LGG 1	TPDCV S. Francisco – regimen	Vincristin/ Carboplatin CCSG	VCR-ACT/D	BB – SFOP series
n	204	42	78	29	85
Complete response (CR)	4.0%	-	5%	-	-
Partial response (PR)	46.0%	-	28%	2*/29 (7%)	56%
Minor response (MR)	-	-	23%	17/29 (59%)	-
Stable disease (SD)	33.7%	-	37%	9/29 (31%)	31%
Progressive disease (PD)	16.3%	-	6%	1/29 (3%)	13%
CR+PR	50% ± 3.5%.	-	33%	7%	-
CR+PR+MR	-	35.7%	56%	66%	56%
CR+PR+MR+SD	83.7% ±2.6%	95.2%	93%	97%	87%

*50% tumor volume reduction
Legend: CCSG - Children's Cancer Study Group; SFOP - French Society of Pediatric Oncology; VCR - Vincristin; ACT-D - Actinomycin D; CR - complete remission; PR - partial remission; MR - minor response; SD - stable disease

The relevance of tumor volume reduction for long term patient outcome and functional status of children with LGG has to be discussed in conjunction with achieving the main primary goal of deferring radiotherapy. Translation of tumor response into progression free survival rates will be discussed below.

However, it should be added that tumor volume reduction may have a beneficial effect on severe neurologic symptoms at presentation, especially diencephalic syndrome (DS). Gropman et al reported the outcome of 7 children presenting with hypothalamic-chiasmatic glioma and DS (aged 9-20 months, median 11 months) treated with chemotherapy. At a median follow of 29 months (range 6-54 months) the patients' weights had increased by 66-95% (median 80%). On MRI four patients had a > 50% reduction of the tumor mass, one a

25-50% reduction and two stable disease. In those patients who showed a tumor volume reduction to CT, weight gain was accomplished by oral feeding in 4 of the 5 patients, whereas those with SD required nasogastric or gastrostomy tube supplementation to maintain weight. Only 2 of those children were censored progression-free at the end of the follow-up time (Gropman 1998).
Laithier investigated the clinical outcome of 14 children (age range 3-25 months) affected by a hypothalamic-chiasmatic glioma (HCG) and diencephalic syndrome. In this series weight gain was observed in those patients who responded to treatment. Tumor progression occurred in 11 of these children. Six of them were irradiated, at a median interval of 30 months from starting chemotherapy. Interestingly the 3-year progression free survival of children with HCG with and without diencephalic syndrome was 17% and 57% respectively (p=0.001) with however an overall survival of 89% in both groups (Laithier 2002).

3.2.3.2. The role of chemotherapy in terms of progression free survival for low grade glioma

Progression free survival would be the easiest parameter to judge the effect of chemotherapy on childhood low grade glioma. When comparing results from different studies, study population characteristics vary and influence the interpretation of results. The BB-SFOP data refer only to children with HCG and OPG, whereas the cohorts of the reports from Packer (1997) and Prados (1997), as well as the SIOP-LGG 1 study, include children with tumors of all sites. The timing and criteria of expressing treatment results are quite different. Most studies calculate 3-year progression free survival, which in terms of delaying radiotherapy is a relevant interval in the sense that: deferring progression for at least three years will allow the young children to reach an age of around 5 years before being irradiated. But it is not possible to compare 5-year PFS rates.

The 5-years progression free survival (PFS) of children affected by a low grade glioma and treated according to the SIOP-LGG 1 study is 48% (95%CI 31,6%-64,2%). As expected, the overall survival of this cohort of children is favorable with a 5-year OS 89,1% (95% CI 84,1-94,0%).
The PFS produced by other concurrent trials on LGG are reported in the table below (table 4). In the San Francisco experience the median time to tumor progression was 132 weeks (95% CI 106-186 weeks).

Table 4: Progression Free Survival in children affected by LGG and treated with chemotherapy

	**SIOP-LGG 1**	**CCGS**	**VCR-ACT/D**	**BB-SFOP**
Survival data	5 years PFS	3 years PFS	PFS at 6 years median F-U	3 years PFS
n	204	78	29	85
All patients	48% (95%CI 31.6%-64.2%)	68% ± 7%	~ 30%	48 % (37-60 %)

Legend: CCSG - Children's Cancer Study Group; SFOP - French Society of Pediatric Oncology; VCR - Vincristin; ACT-D - Actinomycin D; PFS - Progression Free Survival.

It can be concluded, that the PFS-data, particularly after a prolonged follow-up time, are not satisfactory, yet.

3.2.3.3. The role of chemotherapy in terms of radiotherapy-free interval for low grade glioma

One of the main motives to investigate chemotherapy in children with a low grade glioma is to defer the use of radiotherapy (RT) as long as possible (and hopefully forever), and thereby to avoid the deleterious effects of radiotherapy on a developing brain. Thus, the radiotherapy-free interval has been proposed as a criteria for judging the effect of chemotherapy. However, many young patients after having failed first line chemotherapy receive alternative chemotherapy-regimens instead of being irradiated. Thus, especially in very young children, the RT-free interval is the result of multiple interventions and can not be used as an indicator of the effect of one specific chemotherapy regimen, unless the other interventions are recorded and included in the analysis.

In the SIOP - LGG 1 study 41 children ended-up being irradiated. The median time interval between date of beginning chemotherapy and of radiotherapy was 22,2 months (range 1,3-67,6m). The median age of these children at the time of diagnosis was 54,3 months (range 3,5-164,8m) and at the time of beginning RT was 84,0 months (range 7,2-167,3m). In the VCR/ACT-D experience reported by Janss et al (Janss 1995) the RT delay in those children who were ultimately irradiated was of 4 years and 3 months (range 1 months and 10 years). As said, it is very difficult to compare these data, considering the many factors, which can influence the decision of irradiating a child.

Despite these limitations, the intent to delay radiotherapy in children with low grade glioma, specifically visual pathway gliomas, remains the primary goal for any therapeutic intervention.

3.2.3.4. The role of chemotherapy for progression free survival of hypothalamic-chiasmatic and optic pathway glioma

Hypothalamic-chiasmatic glioma (HCG) and optic-pathways glioma (OPG) represent a relatively homogeneous group of childhood low grade glioma, if for nothing else as for the clinical challenge they present. By themselves, the HCG and OPG do not represent a significant prognostic group, but since resection only plays a subordinate role, it is worth analyzing the data for this group of children separately. The PFS rates according to the different series are reported on table 5.

Table 5: Five years Progression Free Survival of children with Hypothalamic-Chiasmatic glioma treated with chemotherapy.

	SIOP - LGG 1	CCGS	VCR-ACT/D	BB-SFOP	St.Jude
n	204	78	29	85	??
Hypothalamic-chiasmatic LGG	39.2% ± 7.2%	77.6% *	~30%	~37%	12.11%

Legend: CCSG - Children Cancer Study Group; SFOP - French Society of Pediatric Oncology; VCR - Vincristin; ACT/D - Actinomycin D.
*3 years PFS

The 77.6% 3-year PFS survival produced by the CCSG cooperative study clearly stands quite significantly over the other treatment results. But the PFS-curve produced in the paper reporting the CCSG experience, does not have any plateau and at 5 years the curves seem to drop in the range achieved by the other study groups. It should also be noted that only three

children with a multicentric/disseminated LGG have been included in the CCSG series, while in the SIOP 14 children (11.3%) with such unusual variety of LGG have been registered and included in the survival curves.

In the VCR/ACT-D experience, 22 out of the 32 evaluable children experienced tumor progression after stabilisation or shrinkage. Median time to progression was 27 months (range 1-92 months). 72% of patients ultimately had tumor progression; CT delayed the use of RT beyond 5 years of age in more than 70% of the patients. Radiation therapy was delayed a median of 4 years and 3 months (range 1 month to longer than 10 years).

In the French experience with the 'BB - SFOP protocol' (including Cisplatin/Etoposide–Carboplatin/Procarbazin - Cyclophosphamide/ Vincristin) in a cohort of 85 hypothalamic-chiasmatic glioma the 3 years PFS was in the range of 37 to 60 %.

Sposto describing the survival data of a series of 18 children treated for a progressive or symptomatic HCG/OPG with a nitrosourea-based regimen, reported that no median time to tumor progression was reached at a median follow-up time of 78 weeks.

In summary the long term PFS of this cohort of LGG patients are not satisfactory results in any of the series. And they are identical to those published for other diencephalic tumors (Gururangan 2002). They justify searching for methods to improve outcomes.

3.2.3.5. Allergy to Carboplatin

Urticaria, eczema, abdominal or thoracic pain, cough, fever and dyspnea are symptoms of Carboplatin hypersensitivity (Chang 1995, Weidmann 1994).

As a whole 43 (21,1%) of the 204 patients entered into the SIOP - LGG study 1 had allergic reaction to Carboplatin at a time interval between the beginning of CT and "allergy" ranging from 1 to 52 weeks (median 33 weeks). However, this could be an underestimation of the real incidence of the problem; in fact, among the Italian patients, 17 out of 47 children (36,2%) actually manifested allergic reaction to Carboplatin. In the CCSG experience only 5 out of the 78 (6%) eligible patients had allergic reactions to Carboplatin, and only 6 out of the 60 (10%) of those treated in the pilot protocol (Packer 1993 and 1997). However, during the on-going randomised trial the number of Carboplatin-allergies exceeds 30 % (J. Ater, personal communication). The schedules within the Carboplatin / Vincristin regimen used by the CCSG group and the one used by SIOP are quite different, but the total doses are in the same range.

Table 6: Comparison of Carboplatin dosage between regimens.

	**SIOP regimen**	**CCSG regimen**
CARBOPLATIN (dose per cycle)	550 mg/m^2/d1	175 mg/m^2/d1 x 4 weeks
No. of doses/cycles	15	12
Duration of therapy, weeks	53	79
Cumulative dose of CARBOPLATIN	8250 mg/m²	8400 mg/m²
Allergic reactions	36,2% Italian population<br>21 % study population	5/78 (6%) institutional trial<br>> 30 % randomised trial

In the literature the occurrence of hypersensitivity to Cisplatin is described in 5 to 20 % (Morgan 1994, Ciesielski-Carlucci 1997), while allergy to Carboplatin is mentioned with less than 5-6 % (Morgan 1994, Charlene 1996, Packer 1997). It is speculated that the number of exposures is responsible for the development of hypersensitivity reactions, since the risk of allergy to Cisplatin rose from 6 % in the 6th cycle to over 67 % in the 10th cycle in a series of adult women with ovarian cancer (Morgan 1994). Repetitive, weekly dosing has been reported to enhance the probability of allergic reaction in brain tumor patients (Yu 2001).

There are no known risk factors (Weidmann 1994, Chang 1995). Reports concerning hypersensitivity have been published following treatment with a variety of schedules and cumulative doses in different tumor types. Concomittant medication was variable. It is interesting to note that some reactions have occurred more than a year following first treatment with Carboplatin in the setting of a new therapy (Weidmann 1994).

The mechanism of the underlying immune reaction could not be elucidated yet. An IgE-mediated immune reaction with Platinum-compounds acting as haptens is possible, as well as an non-specific Histamine-release by platinum-salts (Weidmann 1997). Perhaps there is a reactive metabolite, activated by leucocytes, with infection or inflammation as risk factors for its release (Utrecht).

Some authors have reported successful desensitization in children, who had developed severe hypersensitivity reactions necessitating the interruption of Carboplatin-treatment (Charlene 1996). Desensitization with concurrent steroid and antiallergic medication has to start the morning of the planned treatment with extremely low starting doses. This procedure is only justified in case of unavailability of any alternative treatment. Cases have been described, where alternative therapy with Cisplatin has been successful despite previous allergy to Carboplatin (Weidmann 1994).

There are no data concerning the effectiveness of continuing therapy with Carboplatin once allergic reactions have developed.

Despite the risk for allergy, the use of Carboplatin can be considered one of the backbones of successful therapy for childhood LGG. Monitoring incidence, clinical symptoms and the course of disease after manifestation of allergy are necessary to define its impact for the overall treatment strategy.

3.2.3.6. Analysis of prognostic factors

Results given for the various trials, SIOP - LGG 1 included, are data derived from quite heterogeneous groups of children affected by a low grade glioma.

NF status

Among children affected by a progressive or symptomatic LGG and treated with chemotherapy according to the SIOP - LGG 1 study, the only patient / tumoral characteristic that predicted for tumor behavior was NF I status.

In the SIOP - LGG 1 study the NF I status predicted a prolonged PFS. In fact, the 5 years PFS for children without NF I were 50,9% (95%CI 40%-60%) and with NF I 66,5% (95% CI 53,7% -79,4%; p > 0,*016*) respectively. These data may reflect either a more favorable response to CT or a more indolent and benign course of the LGG associated with NF I than of those occurring in children without NF I. In a recent series of HCG/OPG treated with CT at the St.Jude Research Hospital the NF I status and the initial treatment with RT were the two most significant predictors of a longer PFS, while NF I status, tumor size < 10 cm^3, the lack

of ventriculomegaly and more than 50% tumor enhancement were the clinical findings positively influencing the outcome (Fouladi 2002).
There are certainly inconsistencies between studies with regard to NF status and eligibility criteria for enrollment. In this study NF I status is the major stratification factor. Case ascertainment will be critical, if the results are to be interpretable.

Tumor location
Most published series focus on the chemotherapy of tumors of the supratentorial midline, in particular hypothalamic-chiasmatic glioma. Although many studies have been open for tumors of other locations as well, their numbers have been small, not allowing a systematic analysis.
However, the response rate of Non-OPG tumors is relatively high compared to OPG in the French experience: The best objective response was 7/8 in intramedullary glioma (Doireau 1998), 10/11 in brainstem glioma (Pagnier 2002), while it was 45 % only in the OPG (Laithier, submitted). Few patients have been irradiated. In the CCSG series (Packer 1997) no difference of 3 year PFS was seen between diencephalic tumors, brainstem tumors and other cranial tumors, whereas all 3 tumors with leptomeningeal dissemination were progressive following an initial response in 2. In the SIOP-LGG 1 study, the number of tumors located outside the supratentorial midline is quite large. Responses (CR+PR+SD) were obtained in 9/11 tumors of the cerebral hemispheres, 26/34 of the posterior fossa and 6/7 of the spinal cord. No separate analysis of PFS for tumor location has been performed.
Thus it is not clear whether primary tumor location plays a role for tumor response to chemotherapy or for PFS.

Tumor staging
Surprisingly, in the SIOP - LGG study 1, the presence of multicentric/disseminated disease did not have an influence on the patients' outcome. The 3 years PFS of children presenting with disseminated disease and treated with CT was 49% (95% CI 29,6-70,1%) and for the ones without 59,1% (95% CI 50,8-67,3%).
Timing of treatment
The only other clinical parameter, which in SIOP - LGG 1 seems to predict a different progression free survival, was the interval between diagnosis and the start of treatment. Children, who were treated at diagnosis had a 5 years PFS of 29,4% (95%CI 11,9%-46,8%), which was significantly inferior to the PFS of the children treated after a period of observation, 63,3% (95%CI 45,3%-81,4%; p=0,0063). The interpretation of these data is difficult. It has been assumed that the children treated at diagnosis had worse symptoms and therefore more aggressive tumors. The challenge for future studies would be to precisely identify those patients, by using some other criteria, since the present analysis does not define any clear-cut clinical or pathological risk-factors.
Furthermore, to explain this phenomenon some more imponderable reasons could be advocated such as the fact that after a prolonged period of observation, physicians and parents became "more nervous" in deferring treatment, and were more willing to submit their child to therapy, even in the absence of convincing clinical and neuroradiological evidence of tumor progression. It is hoped that greater standardisation of indications to commence therapy will emerge from the experience of the present study proposal.

Age at diagnosis
The data on the relevance of age at diagnosis as prognostic factor are contradictory.
In the CCSG trial age was pointed out as a possible predictor of different outcome. Children who were younger than 5 years did better than those older than 5; their PFS was 63,3% (45,3-81,4%) versus 29,4% (11,9% -46,8%) (Packer 1997 *)*. The mean age of their study population was 3.08 years. In contrast to the CCSG experience, in the San Francisco series the younger children did worse than the older ones (p=0.004; risk ratio 0.81) (Prados 1997). The

mean age in their study population was 5 years. Similarly, in the SFOP study children younger than 5 did significantly poorer than the older ones (Laithier 2000). Actually in the 10 year review of the LGG treated at the St. Jude Research Children's Hospital age seems to influence the outcome, once again being the children less than 5 doing worse than the over 5 years (Fouladi 2002).

Response to therapy
In the SFOP experience, after the NF I, status the most significant prognostic factor for a better PFS was the response to CT. Patients who had an objective response (PR and/or CR) to CT had a longer PFS than those who had a minor or stable disease after CT (3 year PFS 60% versus 25%). In the SIOP - LGG study 1 the 3 year PFS was 69,7 % (95% CI 58,7-80,7%) for the patients who obtained "some response" and 66,8.% (95%CI 53,9-79,7%) for the ones who had stable disease. Similarly, in the CCSG experience no difference in terms of 3-year PFS was documented between patients who had a major response and the ones who had just a minor response or stable disease: 83% ± 8% and 73% ±9% respectively (Packer 1997).

Pathologic/biologic criteria
No solid data exist on the role of possible pathologic and biological characteristics of childhood LGG in predicting different outcome.
Recently Tihan et al in the pathology file of the Johns Hopkins Hospital identified 18 cases of JPA with a distinctive monomorphous pilomyxoid histological pattern (monomorphous spindle bipolar cells in a fibrillary myxoid background, angiocentric arrangement and no Rosenthal fibers with a low labelling index (LI -Mib-1) in the range of 2% - 5% (Tihan 1999). The majority of the tumors occurred in infants and young children (median age 10 months) and involved the hypothalamic/chiasmatic region. In this cohort of patient the PFS at 1-year was 38,7%. In comparison they identified a control group of 13 classical JPA in the same range and location as the study group with a one year PFS of 69,2%, which was significantly better than that for pilomyxoid tumors (p=0.04). However, the precise definition of monomorphous pilomyxoid pattern is still a matter of discussion.
Data exist on a small cohort of children which seems to indicate that the LI may be prognostically significant as well as the loss of 17p (Prados 1992, Willert 1995). However these data are still awaiting confirmation.

3.2.3.7. Functional/neurological outcome of children with LGG treated with CT

The vast majority of children affected by LGG are expected to be long term survivors. Thus, health status (HS) and the quality of life (QoL) remain significant criteria to judge the effect of a therapeutic approach. More specifically, for children with HCG and OPG the goal of any therapeutic intervention must also include the preservation and hopefully the improvement of the visual function and of the endocrinological status, besides the neurological status as a whole. The contribution of each specific modality of treatment to this functional aspects of children affected by HCG and OPG have not yet been fully investigated.

The fact that these children may have a poor "functional outcome" is outlined by some recent data. Cappelli (Cappelli 1998) studying a cohort of 44 long term survivors of HCG treated with RT, reported that 18 had major academic failures and that 12 were actually institutionalised. Ten of these 44 attended schools for blind. In the series reported by Sutton et al 43% of them required a "special school" including resources room, learning-disabled classes or special education. Of the 27 surviving patients for whom follow-up information was available, six were described as having "few or no friends", three were receiving Methylphenidate for attention deficit disorders, four were described as " passive" and two as

"very emotional" (Sutton 1995). Severe behavioural and also psychological and psychiatric disorders were also observed by Janss et al in a cohort of 46 long term survivors with HCG and OPG (Janss 1995).

Regarding the functional outcome in three large series of patients treated with chemotherapy for a progressive hypothalamic-chiasmatic glioma, it is said respectively that 15 out of 18 (Petronio 1991), 23 out of 24 (Packer 1988) and 19 out of 27 (Janss 1995) had visual stabilisation or improvement, however no more details are provided in the papers. Sutton reported the visual outcome in a cohort of 33 children with hypothalamic-chiasmatic glioma and stated that 5 were functionally blind in both eyes at the end of follow-up. Interestingly all children who were functionally blind had been very young at presentation. One of these children had an initial good response to CT given at the age of 2 and he is reported free of tumor at the age of 13, but blind. The other four children were irradiated at the age of 3 to 6, but it is not clear if this affected their vision (Sutton 1995).

Other investigators reported that RT seems to be more effective than CT in preserving and improving the visual function. Cappelli et al reporting the functional status in a cohort if 44 long term survivors of children with an HC glioma reported that after RT 18 had a visual improvement, 29 a stable vision and 7 some deterioration (Cappelli 1998).

In summary no prospective studies have been so far conducted evaluating carefully the visual outcome of children with HCG and OPG despite the relevance of this treatment outcome criteria. Unquestionably, the functional outcome of these children must become a crucial end-points of any study aiming to evaluate treatment strategies on these children. The data available so far seem to indicate that these children may suffer because of a variety of reasons of severe sequelae.

Endocrine sequelae

Almost all children with HCG need some hormone replacement following treatment, actually 28 out of 33 in the series reported by Sutton et al (Sutton 1995). Needless to say that children with supratentorial midline low grade glioma treated with chemotherapy are expected to have fewer endocrinological sequelae than the ones being treated with radiotherapy. Other than specific hormonal problems, children with HCG may seem to suffer from more complex growth disorders. In Sutton's series of 33 children, it appears that these patients tend to cluster into obese (>90th percentile for weight, 8 patients) and diencephalic (< 10th percentile for weight, 8 patients), with the diencephalic one usually short in stature and the obese ones, tall.

3.2.3.8. Conclusions

Summarising the above data on the role of chemotherapy in low grade glioma, it can be said that:

- CT has a consolidating role in the treatment of children with LGG at least in terms of tumor response, PFS and radiotherapy-free interval;
- the progression free survival data are still unsatisfactory, especially for children with HCG and OPG;
- no reliable prognostic factors have been identified so far other than the NF1 status; the investigations into patient's clinical characteristics and into tumor histological and biological tumor profiles are becoming increasingly urgent;
- the functional outcome of the children treated should become a major end-point of any future studies directed to test specific therapies on children with LGG.

3.3. Rationale for a differentiated chemotherapy schedule for the study SIOP LGG 2004

This protocol aims to present an integrative approach for the treatment of all low grade gliomas irrespective of their location and histological subtype.

Chemotherapy will be the primary non-surgical therapy for several subgroups of children. The chemotherapy strategy for childhood LGG shall be further developed within the context of a controlled prospective trial. The main reasons to launch the new strategy can be summarised as follows:

- ✓ to adopt the therapeutic concepts already proven to be potentially effective in treating these neoplasms (see section 3.2.)
- ✓ to stratify treatment for subgroups according to currently available prognostic criteria (see section 3.2. and 12.)
- ✓ to use as the standard treatment arm the "historical" regimen with Vincristin and Carboplatin from the SIOP - LGG 1 study (see section 4.)
- ✓ to introduce into the induction regimen a new drug which may improve the results for progression free survival and to test it by a randomisation procedure (see section 3.3)
- ✓ to extend the duration of therapy which may increase the number of major responses, as they seem to develop over prolonged periods of time
- ✓ to limit, as much as possible, the risks of long term side effects of chemotherapies employed
- ✓ to define more accurate and relevant end-points for treatment outcome evaluation (see section 17.)

Although no phase III prospective randomised clinical trial comparing different regimens has been completed so far, the combination of Vincristine/Carboplatin, as used in the previous SIOP - LGG 1 trial, represents the standard treatment for childhood low grade glioma in Europe at this time. This combination seems to respect appropriately the risk/benefit ratio for these children with minimal risks for late effects. Due to unsatisfactory progression free survival data in LGG this combination needs to be strengthened to improve outcome. The experience of delayed development of Carboplatin allergy may prevent a more extended schedule of this drug combination and justifies the consideration of adopting alternative regimens being proposed, where there is existing data.

3.3.1. Effective drugs in the treatment of low grade glioma

Except for the trials conducted by the POG on Carboplatin and Iproplatin (Friedman 1992) and by Gururangan on Carboplatin (Gururangan 2002) in progressive or recurrent low grade glioma, no other conventional phase II studies on childhood LGG have been run. For most drugs, used alone or in combination for treating LGG, only results on small series of patients form the basis to evaluate efficacy by response assessment as given in the reports.

Table 7: Chemotherapy trials in low grade glioma: Response assessment

ND: newly diagnosed; R: relapsed; diss: disseminated tumors; w: week(s); CT-scan: computed tomography; MRI: magnetic resonance imaging.
Response: CR: complete response, PR: partial response (tumor volume reduction of > 50 %), MR: minor response (tumor volume reduction of < 50 %, but > 25 % of initial volume), SD: stable disease, PD: progressive disease (tumor volume increase of > 25 %), Response Rate (for single drugs): CR+PR+MR+SD.
MI: missing information; *: Data included in subsequent reports.

DRUG/COMBINATION	NUMBER OF PATIENTS	DOSE / m²	TREATMENT INTERVAL	ASSESSMENT OF RESPONSE BY	TIME OF ASSESSMENT	CR	PR	MR	SD	PD	MI	Response Rate
**CARBOPLATIN**												90/105 – 85 %
Friedman (JCO 1992)	R 7	560 mg	4w	CT-scan/MRI	8w				5	2		
Moghrabi (J Neurosurg 1993)	ND 4, R 2	560 mg	4w	MRI	8w				6			
Aquino (J Neurooncol 1999)	ND 12	560 mg	4w				4		6	2		
Gururangan (JCO 2002)	ND 58, R 23	560 mg	4w	CT-scan/MRI	8w	2	17	4	46	11		
**IPROPLATIN**												11/15 – 73 %
Friedman (JCO 1992)	R 15	270 mg	3w	CT-scanMRI	6w		1		10	4		
**CYCLOPHOSPHAMIDE**												19/25 – 76 %
McCowage (MPO 1996)*	Diss 4	4-5 g	4w	MRI	no information		2	1	1			
Longee (J Neurosurg 1998)	R6, diss 4	4-5 g	4w				5	1	3	1		
Kadota (JPHO 1999)	ND 15	1,2 g	3w	CT-scan/MRI	12w	1			9	5		
**IFOSFAMIDE**												4/6 – 66 %
Heideman (J Neurooncol 1995)	R6	3 x 3g	3w	CT-scan/MRI	6w		1		3	2		
**TEMOZOLOMIDE**												9/20 – 45 %
CCG (unpublished)	R 20	5x200 mg	4w	CT-scan/MRI	8w		1		8		12	
**MTX**												7/10 – 70 %
Mulne (JPHO 2000)	R 10	7,5 mg x8 (q6h)	w	CT-scan/MRI	2 months		2		5	3		
**TOPOTECAN**												7/13 – 54 %
Blaney (Cancer 1996)	R 2	5,5-7,5 mg	3w	CT-scan/MRI	6w		1		1			
Kadota (J Neurooncol 1999)	R 11	3-3,75mg (CI)	3w	CT-scan/MRI	6w				5	6		
**ETOPOSIDE**												14/26 – 54 %
Chamberlain (JCO 1995)	R 14 chiasm-hypot.	50mg x 21 d	5w	CT-scan/MRI	8w	1	4		3	6		
Chamberlain (J Child Neurol 1997)	R 12 cerebellar	50mg x 21 d	5 w	CT-scan/MRI	8w		2		4	6		

DRUG/COMBINATION	NUMBER OF PATIENTS	DOSE / m²	TREATMENT INTERVAL	ASSESSMENT OF RESPONSE BY	TIME OF ASSESSMENT	CR	PR	MR	SD	PD	MI	Response Rate
**VCR + ACTINOMYCIN D**												
Packer (Ann Neurol 1988)	ND 24	1,5mg - 15 mcg	12 weekly		12w		3	6	14	1		
**VCR + Carboplatin**												
Packer (JCO 1993)	ND 37	1,5mg - 175mg	weekly	CT-scan/MRI	10w	1	15	7	13	1		
Packer (JCO 1993)	R 23	1,5mg - 175mg	weekly	CT-scan/MRI	10w		7	5	5	6		
Packer (J Neurosurg 1997)	ND 78	1,5mg - 175mg	weekly	MRI	10w	4	22	18	29	5		
Perilongo (MPO 2000)	ND 132	1,5mg - 550mg	3-4w	CT-scan/MRI	10w	5		56	49	22		
**Carboplatin + VP 16**												
Castello (MPO 1995 / CNS 1998)	ND 17, R2	300-1000mg - 600mg	3-4w	CT-scan/MRI	12-16w	1		6	8	4		
**VP 16 + VCR**												
Pons (J Neurooncol 1992)	R14, ND 6	1,5mg - 5x100mg	6w	CT-scan/MRI			1	3	11	5		
**TDBCV**												
Petronio (J Neuros 1994)*	ND 15	see paper	6w	CT-scan/MRI	no information			11	2	2		
Prados (J Neurooncol 1997)	ND 42	see paper	6w	CT-scan/MRI	6w			15	25	2		
**BB SFOP**												
Laithier (MPO 2000)	ND 84	see paper		CT-scan/MRI			47		26	11		

The compilation in Table 7 focuses upon response only, since survival and progression free or event free survival data are not available for the smaller series and can hardly be compared due to the variable settings.

It is not possible to judge from this data the superiority of a single drug or drug combination of drugs over another. It should be noted that responses have been reported for all drugs tested. It is tempting to say that "these tumors respond to everything". VP 16 was selected by the SIOP - LGG study 2 committee to be added to the historical regimen during the early induction phase in order to evaluate its impact on improving "the effectiveness" of Vincristin/ Carboplatin, mainly for its possible synergism with the platinum derived agents.

3.3.2. Rationale for the intensification of induction treatment

In the SIOP - LGG 1 study, 84 children (41,2%) of the 204 evaluable patients suffered from a tumor-related event. 34,5% of these failures (29/84) occurred in the first 4 months of therapy. Although one of the possible explanations is that, at the time period the study was conducted, clinicians were still not used to treat the low grade glioma with chemotherapy and tended to interpret any tumor enlargement as progression and therefore overstate chemotherapy failure. It has been a common experience to observe some tumor volume increase during the very first weeks of therapy, followed by a stabilisation or by decrease of the tumor dimensions. Since, as in comparable trials as well, there was no central radiologic assessment, a definite conclusion as to the relative importance of this assumption is impossible.

In the CCSG experience only 6% of the patients failed during the first 10 weeks of therapy while the vast majority of the tumor failures were documented after stopping therapy. The median time to tumor progression in the cohort of 42 children they treated with TPDCV was 132 weeks (95% CI 106-186 weeks), thus half of the progressions occurred within the first 24 months of therapy

Table 8: Occurrence of tumor progression; comparison between SIOP low grade glioma Study 1, the CCSG trial and the BB-SFOP study

	**SIOP study**	**CCSG trial**	**BB-SFOP**
n	204	78	85
Median time of follow-up	35,4months	30 months	52 months
Patients with PD	84/204 (41,2%)	27/78 (35%)	46/85 (54 %)
Patients lost in induction	29 (14,2%)	5 (6%)	11 (13 %)
During maintenance	14 (6,9 %)	6 (7%)	7 (8 %)
After stopping therapy	41 (20,1%)	16 (21%)	28 (33 %)

No previous study has investigated the impact of the intensity of induction treatment upon long term tumor control in low grade glioma. As discussed in section 3.2. neither response rate nor progression free survival rates can be compared between studies and no significant differences have been detected between regimens. But as suggested by trials like the rather intensive regimen BB-SFOP a higher rate of objective responses may be expected to prolong progression free survival.

To reduce the high number of early tumor progressions, in the SIOP - LGG 2004 trial the initial phase of chemotherapy (Induction) shall be intensified by adding Etoposide in a prospective, randomised trial. It is expected that a reduction of the early progression rate will result in an improved long term progression free survival. As suggested by some studies, especially the SFOP experience, a more favorable response distribution may also improve the long term PFS.

3.3.3. Rationale for the differentiation of consolidation therapy

1. Overall therapy duration
The problem of the "optimal" time duration of chemotherapeutic treatment for LGG has never been addressed properly. The duration of the various regimens so far published varies quite significantly. In several published series, authors stress that the number of patients with a "major" therapy response is increasing as treatment continues.

Table 9: Duration of therapy in chemotherapy trials for the treatment of low grade glioma.

**Drug combination**	**Duration**
Cisplatin/Vincristin	from 16 to 32 weeks
6-Thioguanine, Procarbazine, Dibromodulcitol, Lomustine, Vincristine	24 weeks
Vincristin / Actinomycin D	48 weeks
Cisplatin/VP 16 –	12 months
Carboplatin/Procarbazin; Cisplatin/VP 16; Vincristin/Cyclophosphamide	12 months
Vincristin/VP 16	18 months
Carboplatin/Vincristin	Up to 79 weeks

Considering the difficulties in asking in a prospective randomised fashion, on top of the Etoposide question (randomisation of induction), also the "duration of therapy" one, it was elected to treat all children for 18 months in the subsequent trial, assuming the working hypothesis that these children are in fact affected by a sort of chronic, slow growing disease which deserves prolonged therapy. The time duration chosen was the one adopted by the CCSG trial, which is 18 months.
Ideally, this change should also be introduced in a prospective, randomized manner. However, due to the fact that recruitment rates even within a large European trial do not allow multiple randomisations, this has been considered unfeasible.
By adopting standardized cycle length and total treatment time to the American CCG trial an international comparability of trial results will be feasible in the future.

2. Prolonged time intervals between courses during the continuation therapy
To avoid increasing substantially the cumulative doses of the drugs chosen for the study when prolonging therapy to 18 months, the time interval between courses during the continuation-therapy phase shall be extended to six weeks, analogous to other so-called maintenance treatments. Yet, to avoid prolonged treatment free intervals (6 weeks) additional Vincristin will be given in weeks 2 and 3 of each cycle.

3. Alternative drug combinations for children developing allergy to Carboplatin
For those children who at some time during their chemotherapy schedule develop allergy to Carboplatin, this has been a major problem to maintain total treatment time. Depending upon

the time of manifestation of allergy a variety of measures have been adopted. Besides premature termination of therapy in individual cases, the majority of children has received "alternative" drugs, with mostly individually chosen schedules and cumulative doses. So no coherent analysis of these various measures can be taken.
Within the SIOP-LGG 2004 trial a uniform approach following Carboplatin allergy is recommended, which has the goal to maintain total treatment time of 18 months.
To ensure this goal the study recommends a standardized approach. Two alternating couples of drugs shall be administered sequentially on a 6-week schedule, as for Carboplatin/Vincristin, with additional Vincristin given in weeks 2 and 3 of each cycle: Cisplatin/VCR and Cyclophosphamide/VCR. Combinations of Cisplatin and Cyclophosphamide have shown efficacy when alternated with Carboplatin in the French "BABY-SFOP" LGG study. They will be combined with Vincristin instead of applying Procarbazin and additional VP 16 to avoid long term toxicity. To limit cumulative doses of Cisplatin and Cyclophosphamide no more than 5 cycles of both combinations shall be given.

4. Alternative drug combinations for children with progression following chemotherapy
Although the primary aim of using chemotherapy in case of a symptomatic and/or progressive low grade glioma has been to defer radiotherapy, and thus it would only appear consequent to start radiotherapy upon tumor progression during or after chemotherapy, various circumstances will make such a choice unwanted.
Many of those children who were very young at diagnosis, will still be young, if they suffer from tumor progression during chemotherapy or within the first years after its terminations. Thus the arguments to defer radiotherapy still hold, especially if the tumor had been responsive to primary chemotherapy.
Especially for children with Neurofibromatosis NF I the rationale to avoid radiotherapy is valid thoughout childhood and thus sequences of alternative chemotherapies are preferred to early institution of radiotherapy.
Despite these basic considerations, a systematic strategy of sequential chemotherapies has not been investigated up to now. Within this study the recommended treatment for unequivocally progressive tumors, in which radiotherapy shall be further delayed, is to alternate the two combinations of Cisplatin/Vincristin and Cyclophosphamide/Vincristin. Dependant upon the time at which progression is diagnosed the duration of therapy has to be determined.

- For children with primary progressive tumors individual strategies have to be designed.
- For those with early progression, following initial response (as measured at week 24) the same strategy can be used as for the children with allergy (see above).
- For children, where progression gradually develops at any time following the end of primary therapy, resuming chemotherapy at a time schedule as presented for the initial Carboplatin/Vincristin therapy in SIOP-LGG 2004, but substituting Carboplatin/Vincristin by alternating Cisplatin/Vincristin and Cyclophosphamide/Vincristin should be considered.

3.3.4. Safety considerations for the choice of drugs

The cumulative dose (expressed in term of mg/m^2) of the drugs used in the regimens of the studies SIOP-LGG 1 and SIOP-LGG 2004 (2) are reported in the table below:

Table 10: Cumulative doses within the chemotherapy regimens of SIOP-LGG 1 and 2004.

	Vincristin	Carboplatin	Etoposide (randomised)	Cisplatin	Cyclophos-phamide
SIOP-LGG 1	**31,5 21 doses**	**8,250 15 doses**	**-**	**-**	**-**
SIOP-LGG 2004 – TRIAL ARM	**64,5 43 doses**	**9350 17 doses**	**1200 4 cycles à 3 doses**	**-**	**-**
SIOP-LGG 2004 - ALLERGY ARM	**64,5 43 doses**	**variable**	**1,200 4 cycles à 3 doses**	**maximum 300 5 cycles à 2 doses**	**maximum 7500 5 doses**

1. VP 16

The risk of VP 16 related secondary leukemia or myelodysplastic syndrome can be considered to be low counting the facts that:

- the cumulative dose is much less than the one potentially associated with the risk of developing secondary leukaemia;
- the schedule is different from the one thought to be related to secondary AML development (weekly or twice-weekly administration) (Smith 1999).

The Cancer Therapy Evaluation Program of the National Cancer Institute developed a monitoring plan to obtain reliable estimates concerning the risk of secondary leukaemia after epipodophyllotoxin treatment. The main conclusions reported are the following:

"..for cumulative doses of Etoposide of 5 grams/m² or less (given primarily on a daily times five schedule), the risk of secondary leukaemia is not inordinately increased above that contributed by other agents used in the regimens (studied)...";

"..within the context of multiagent regimens that include alkylating agents,…., factors other than epipodophyllotoxin cumulative dose are important in determining the risk of secondary leukaemia..";

"..the Etoposide administration schedule associated with the highest cumulative incidence of secondary leukaemia is weekly to twice weekly administration.."

2. Cisplatin

The risk of Cisplatin (CDDP) related organ toxicity should be low considering:

- That in most circumstances the cumulative dose of Cisplatin will be below the maximum possible dose.
- The fact that the cumulative dose of Cisplatin is administered on a low dose daily schedule, a modality ofadministration, which seems to minimise the risk of organ toxicity. Nevertheless, regular surveillance of organ functions is mandatory for all children receiving the alternative arm.

3. Cyclophosphamide

The risk of developing sterility and secondary tumors associated with the use of 3 gr/m2 cumulative dose of Cyclophosphamide seems to be almost negligible. In fact, only doses of Cyclophosphamide in excess of 5 g/m² have been associated with a risk of sterility, estimated in the 10% range. Several reports have shown that the risk of AML and MDS among patients with early breast cancer who received standard dose of Cyclophosphamide-containing adjuvant chemotherapy is not much higher than in the general population (Valagussa 1994;

Tallman 1995; Holdener 1994). In children treated for rhabdomyosarcoma 3 cases of secondary leukaemia were reported among 68 children treated with a cumulative dose of Cyclophosphamide higher than 16,8 g/m2 and none in the group who received a lesser cumulative dose of the drug (Scaradovou 1995.) Only very high doses of Cyclophosphamide seem to be associated with an increased risk of secondary leukaemia (Kushner 1998).

3.3.5. Rationale for a "chemotherapy-only" schedule in patients with Neurofibromatosis NF I.

The occurrence of brain tumors is a trait of Neurofibromatosis NF I, yet the true incidence of symptomatic CNS-tumors is not known (Huson 1994, Listernick 1997), but estimates range from 0,9 to 15 % (Listernick 1989, 1997).
Data support the concept that low grade glioma arising in children with NF1 have a different biological behavior, but within the NF1 population the clinical and biological behavior of LGG can vary quite significantly, although the majority has a particularly indolent clinical course. Within this group of children cases of spontaneous partial regression of hypothalamic and OPG have been clearly described (and none in non-NF1 children). It has been assumed that only a minority of these children will ever have progressive disease, that this will not occur beyond 6 years of age and that only few children thus need therapy (Listernick 1994). Recent studies have shown however, that delayed tumor progression in these patients is not uncommon (Grill 2000) and within the SIOP-LGG 1 study age of NF I patients needing non-surgical intervention ranged from 1-12 years (median 3,5 years) for those receiving chemotherapy and from 4-11,7 years (median 9 years) for those receiving radiotherapy (Garré 2002).
NF I patients have been included in all recent series upon the treatment of low grade glioma and constituted from 14,3 % (Prados 1997) to 19,2 % (Packer 1997) and 27 % (Kalifa, unpublished) in the larger (chemotherapy) series, and 21,1 % in the SIOP-LGG 1 trial. Most often they were treated according to the age related strategies with radiotherapy for the older and chemotherapy for the younger children.

Although the clinical course of children with NF I, even if unaffected by a CNS-tumor, is extremely variable, a third of these patients experience additional learning difficulties and minor to moderate mental retardation (Huson 1994). Children with NF I and optic pathway tumors treated with chemotherapy had a worse neuropsychological outcome due to the preexisting brain dysfunction even in the absence of radiotherapy, whereas children without NF I receiving chemotherapy as first line treatment have preserved intellectual capacities (Lacaze, in press). The use of radiotherapy for the treatment of visual pathway gliomas in NF I-patients, especially if they are extensive and need large radiation portals, increases the risk of intellectual deterioration. Additionally patients with NF I suffer from an enhanced incidence of radiation induced vasculopathy (Grill, 1999).
A certain percentage of children with NF I having a symptomatic visual pathway glioma will develop other tumors of the central nervous system subsequently, some of them malignant, with reports indicating an incidence of 13 to 52 % (Friedman 1997, Riffaud 2002). Since these tumors may need radiation therapy on their own, it is prudent to avoid primary radiation for the OPG.

When treated with chemotherapy for progressive visual pathway gliomas, children with NF I demonstrate comparable high response rates, but significantly longer progression free survival as compared to children without NF I (Packer 1997, Laithier 2000). This was confirmed in the SIOP-LGG 1 study as well with the application of Vincristin and Carboplatin (Garré

2002). Only few children had progression following therapy, thus NF I patients may benefit from prolonging treatment, but intensification of induction treatment does not seem necessary. Additionally, the risk of inducing secondary malignancy by the use of epipodophyllotoxins or alkylating agents in children with an inherent high risk for secondary cancer shall be avoided.

Therefore this protocol proposes a strategy of first line chemotherapy for children affected by NF I with tumors of low grade malignancy of all CNS-sites.

4. Results of SIOP-LGG 1 **SIOP LGG 2004**

4.1. Study design of SIOP-LGG 1:

Clinical diagnosis or complete / partial resection in absence of signs/symptoms of progressive disease significant symptoms severe neurologic conditions	If at diagnosis or during observation: signs/symptoms of progressive disease significant symptoms severe neurologic conditions	
↓	↙	↘
observation only	Age ≤ 5 years	Age > 5 years
	CT	RT

SIOP - LGG 1 (1993) was the first European study to offer a standardised scheme of therapy for children and adolescents with low grade glioma.

Primary objectives were to evaluate results of these treatment criteria and to determine the effectiveness of chemotherapy (Carboplatin and Vincristine) in treating children aged less than 5 years, with severe or progressive symptoms or unequivocal imaging evidence of tumor growth.

> Consistent with the lack of schedule dependency for platinating agents, the entire dose was given in 1 day instead of distributing it over 4 weeks. Intensified Vincristine was given during induction to augment CNS-concentration.

Secondary Objectives were to provide a standardised clinical treatment scheme within which clinical and biological criteria, including NF1 status, may be studied in order to identify prognostic factors for tumor progression, chemosensitivity, radiosensitivity and overall survival;
....to collect clinical, treatment and outcome data centrally within Europe, so that the natural history of a large number of these tumors can be described within a short time period using modern imaging and therapeutic techniques.
It was hoped to provide an international organisational framework for the initiation of studies on biological material from children with "low grade glioma".

Only the data derived from patients treated with chemotherapy and / or radiotherapy have been combined in the central file; the study report will be focused only on the information derived from these children. The data of study opening varied among nations. It was 1992 for Italy, 1993 for the first German patients and 1995 for the United Kingdom. Recruitment to the international study was closed as of December 31st, 1999, and eventually continued on a national base.

4.2. Chemotherapy part of the study

4.2.1. Patients accrual, time of treatment, clinical characteristics

Patient accrual: 244 patients have been intended to be treated with chemotherapy; 40 were not eligible for the study (Table 11). The recruitment rate by nation is the following: Germany 90, Italy 47, United Kingdom 59, Others 8.

Table 11: Eligibility

	N.	%
**Eligible**	**204**	**83.6**
**Not Eligible**	**40**	**16.4**
different CT	11	4.5
no LGG	3	1.2
second tumor	1	0.4
started CT without evidence of disease	3	1.2
malignant tumor	1	0.4
other site **	3	1.2
Protocol closed	8	3.3
Too many missing data	10	4.2

** Pons

Time of treatment - 130 patients (63.7%) have been treated at diagnosis, while 74 (36.3%) started after a period of observation. The time interval between diagnosis and the date of starting chemotherapy ranged from 0.1 to 30.9 months (median: 23 days) while for the patients who were "intended to be observed", it varied between 2.1 – 164.3 months (median 12.7 months). Patients have been also subdivided, if the treatment started before or after the first three months from diagnosis, regardless of how patients were intended to be treated (Table 12).

Table 12: Time of treatment.

	N.	%
≤ 3 months	117	57.4
> 3 months	87	42.6
Total	204	100

Clinical characteristics – (Table 13) As expected the vast majority of the children treated with chemotherapy were young (median age 35.6 months; range 2.4 – 170.3m), without a clear sex prevalence. Almost a quarter of all patients were affected by Neurofibromatosis type I (NF1). Very few children with a fibrillary astrocytoma have been registered into the study. The reasons could be that:

i) these children are older than the ones affected by a juvenile pilocytic astrocytoma (JPA) and

ii) being these patients older, they are preferentially treated with radiotherapy.

This fact also explains, why few hemispheric LGG are treated with chemotherapy.

25 children (12.3%) aged between 4.5-140 m (median 31 m) presented with a multicentric/ disseminated LGG. The spelling out of their main clinical characteristics (sex, age, primary site and NF status) is reported in Table 14. 16 of them had a histological diagnosis of JPA, 3 of Astrocytoma, 1 of Fibrillary Astrocytoma, 1 Desmoplastic Astrocytoma, 1 Ganglioglioma and the other one Xantoastrocytoma. 2 Children had a clinical diagnosis only.

Table 13+14: Distribution of clinical characteristics:

	all patients		patients with disseminated tumors		
	N.	%		N.	%
**Age:**					
≤ 1 year	41	20.1		9	36
> 1 year and ≤ 3 years	62	30.4		5	20
> 3 years and ≤ 5 years	42	20.6		3	12
> 5 years and ≤ 10 years	39	19.1		5	20
> 10 years	20	9.8		3	12
**Sex:**					
Male	99	48.5		16	64
Female	105	51.5		9	36
**NF1 status:**					
Yes	43	21.1		-	-
No	161	78.9		25	100
**Histology:**					
Astrocytoma n.o.s.	23	11.3		3	12
Fibrillary A.	7	3.4		1	4
Pilocytic A.	104	51.0		16	64
Only clinical diagnosis	61	29.9		2	8
Other diagnosis	*9	4.4		**3	12
**Primary site:**					
Cerebral Hemisphere	11	5.5		2	8
Midline, Supratentorial	152	74.2		15	60
Posterior Fossa	34	16.8		7	28
Spine	7	3.5		1	4
**Primary site:**					
Cerbral Hemisphere	11	5.5		2	8
Hypothalamus	19	9.3		1	4
Thalamus	7	3.4		-	-
Chiasma	56	27.5		4	16
Hypothalamus-Chiasma	49	24		10	40
Optic Nerve	12	5.9		-	-
Basal ganglia	2	1		-	-
III Ventricle	6	2.6		-	-
Pineal Gland	1	0.5		-	-
Cerebellum	13	6.4		3	12
Mesencephalon	2	1		1	4
Brain stem	19	9.4		3	12
Medulla	10			1	
Pons	2			1	
Midbrain	5			1	
Nos	2			-	
Spine	7	3.5		1	4
Total	204	100		25	100

* 3 Oligodendroglioma, 1 Desmoplastic A., 4 Ganglioglioma, 1 Xantoastrocytoma
**1 Desmoplastic Astrocytoma, 1 Ganglioglioma, 1 Xantoastrocytoma.

4.2.2. Results

“Best Tumor response” (at any time)

202 of the 204 eligible patients are presently evaluable for tumor response, 1 is not evaluable because of interruption of chemotherapy after 8 days and 1 for parental refusal. The overall positive response rate (including Stable Disease) is 83.7 % ± 2.6, while the Complete and Partial response rate is 50% (Table 15). No central review of the MRI films was requested; thus, more than “complete” or “partial” response, one should talk of “some tumor volume

reduction". The time of response evaluation varied between 1-21.5 m (median 3.6m). The tumor response by age, sex, NF status, histology, site and disseminated (multicentric/metastatic) disease is collectively reported in Table 16. No significant findings emerged.

Table 15: Distribution of primary response.

	N.	%
Complete Response	8	4.0
Tumor Volume Reduction	93	46.0
Stable Disease	68	33.7
Progressive Disease	33	16.3

Table 16: Tumor response as related to age, sex, NF I-status, histology and tumor site.

	Complete Response	T. volume decreased	Stable disease	Progressive disease	Total
**Age:**					
≤ 1 year	1	18	10	11	40
> 1 year and ≤ 3 years	3	33	21	5	62
> 3 years and ≤ 5 years	1	23	15	3	42
> 5 years and ≤ 10 years	2	12	15	9	38
> 10 years	1	7	7	5	20
**Sex:**					
Male	6	43	33	15	97
Female	2	50	35	18	105
**NF1 status:**					
Yes	1	23	14	6	44
No	7	70	54	27	158
**Histology:**					
Astrocytoma n.o.s.	1	7	9	6	23
Fibrillary	2	-	3	2	7
Pilocytic A.	4	53	32	14	103
Only clinical diagnosis	1	31	19	10	61
Other	-	2	5	1	8
**Primary site:**					
Cerebral Hemisphere	2	4	3	2	11
Midline, Supratentorial	5	70	53	22	150
Posterior Fossa	1	15	10	8	34
Spine	-	4	2	1	7

Progression free and overall survival

PFS: For calculating progression free survival the following definitions were applied: Children in complete remission following chemotherapy had an event at the occurrence of relapse or death and the time from start of chemotherapy up to relapse or death was calculated. Children with a residual tumor had an event at the occurrence of progression or death following chemotherapy and time from start of chemotherapy to progression or death was calculated.

OS: Overall survival is calculated from the time of start of chemotherapy to the time of death.

Fig. 1: Low grade glioma Study: Progression free survival in patients treated with chemotherapy.
The 3-year PFS of the entire population is 57.5%(95% CI 49.7-65.3).

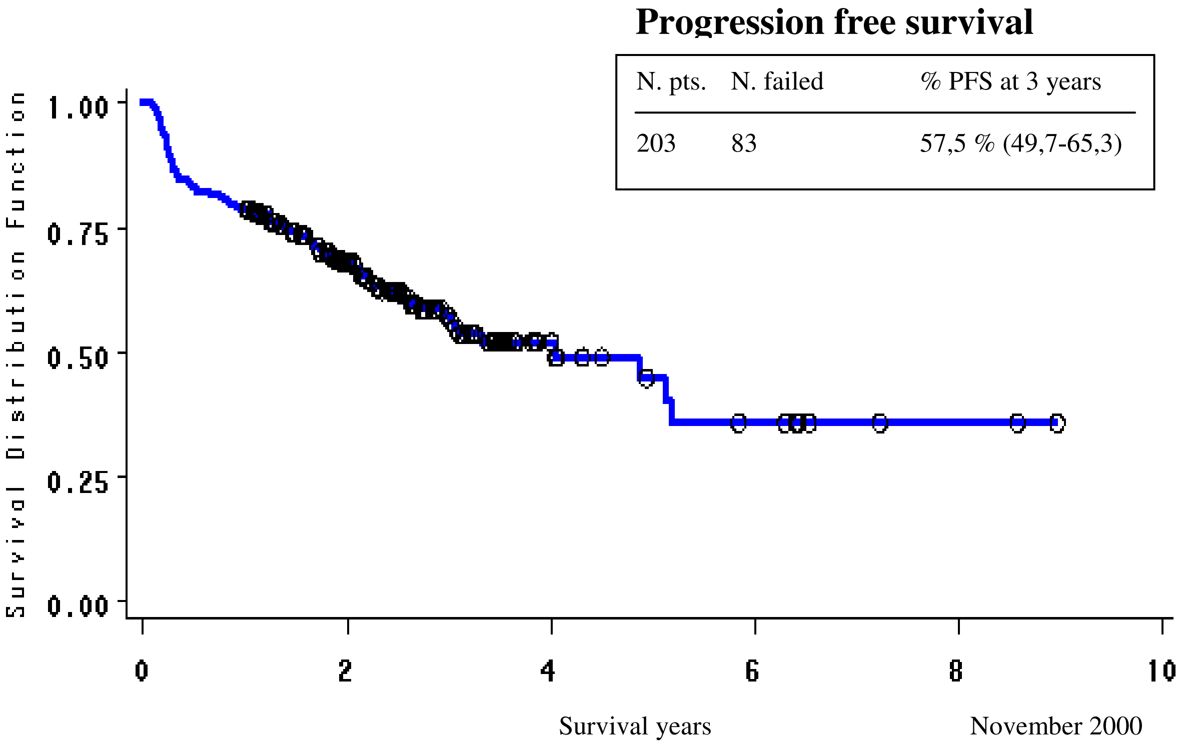

Table 17: PFSs by some patients' clinical characteristics

	No. pts.	No. Failed	% of PFS at 3 years	p-value
**Time of treatment:**				
At diagnosis	130	63	53.0 (43.5-62.5)	0.002
Post event	73	20	66.5 (53.7-79.3)	
**Sex:**				
Male	99	39	59.3 (48.3-70.3)	0.5
Female	104	44	55.9 (45.0-66.9)	
**Neurofibromatosis:**				
Yes	44	12	67.5 (51.9-83.2)	0.02
No	159	71	54.9 (45.9-63.8)	
**Age at diagnosis:**				
≤ 5 years	144	58	56.2 (46.7-65.7)	0.4
> 5 years	59	25	62.4 (49.9-74.8)	
**Metastases at diagnosis:**				
Yes	25	12	49.9 (29.6-70.1)	0.1
No	178	71	59.1 (50.8-67.3)	
**Histology:**				
Pilocytic A.	103	45	56.1 (45.1-67.2)	0.1
Only clininical diagnosis	61	19	66.2 (52.8-79.5)	
**Initial surgery:**				
Only clinical diagnosis	63	19	67.1 (54.0-80.1)	0.004
Biopsy	72	40	41.0 (27.7-54.2)	
**Response to chemotherapy:**				
Stable disease	68	20	66.8 (53.9-79.7)	0.5
Good Response	100	30	69.7 (58.7-80.7)	

Although time of treatment seems to be a highly significant factor for the risk of progression following therapy, this variable is insufficiently precise, since in the SIOP-LGG 1 study the indications for starting therapy were not clearly defined.
The improved outcome for children without initial surgery reflects the high number of children with NF I who entered the study upon clinico-radiological criteria in the majority of cases.

Fig. 2: Low grade glioma Study: Overall free survival in patients treated with chemotherapy.
The 3-year OS of the entire population is very good: 89.1% (95% CI 84.1-94.0).

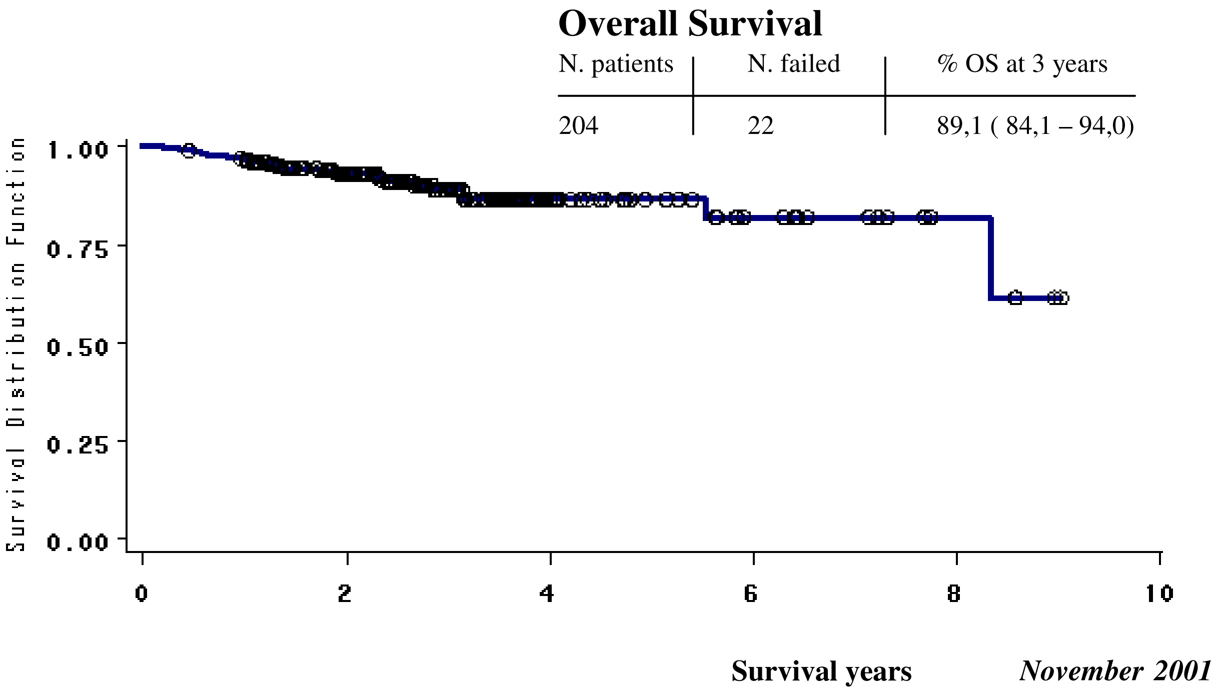

Events and delay of radiotherapy

84 patients (41.2%) of the 204 evaluable patients suffered of a „tumor-related event" (TRE) which was a progressive local disease in 80 (95.2%), a local relapse in 2 (2.4%) and a combined local and distant relapse in the others (2.4%). The median time interval between date of beginning CT and date of event was 11.3 months (range: 1-62.3 m). 12 (14.3 %) of these 84 patients with a TRE had a diagnosis of multicentric/disseminated disease. It is disturbing that the time to failure from beginning of chemotherapy to progressive disease was less than 4 months in a third of the patients, thus immediately following induction (Table 18). In Table 19 the time interval between stopping therapy and date of tumor progression (where known) is reported.

Table 18: Time to failure from the start of chemotherapy

Time to failure from beginning CT to PD	Patients initially "observed"	Patients intended to be treated at diagnosis	Total
≤ 4 months	6	23	**29 (34.5%)**
>4 – ≤ 6 months	2	3	5
>6 – 12	-	9	9
>12 months	13	28	**41 (48.8%)**
**TOTAL**	21	63	84

Table 19: Intervall between the termination of the 12 month chemotherapy and tumor progression (n=23)

	No.	**Time interval between stopping therapy and tumor progression** (months)	MEDIAN (range)
Patients initially "observed"	10	+0, +2, +3, + 7, + 8, +14, +17, +19, +19, +23	**+ 11m** (0 – 23 m)
Patients intended to be treated at diagnosis	13	+0,+1,+2,+7,+8,+8,+9,+11,+14,+16+26,+47, +50	**+ 9m** (0 – 50 m)
TOTAL	23	+0,+0,+1,+2,+2,+3,+7,+7,+8,+8,+8,+9,+11, +14,+14,+16,+17,+19,+19,+23,+26,+47,+50	**+ 9m** (0 – 50 m)

The detailed outcome of the patients who suffered of an event in relationship to the time to progression is spelled out in table 20. An early tumor failure to chemotherapy seems to predict an unfavourable outcome: 12 of the 29 patients (41,4 %) suffering from an early event (within 4 months from the start of chemotherapy) later on died and another 3 continue to be progressive. Another 4 of 14 (28,6 %) with progression during chemotherapy but following induction died and 3 of these 14 are progressive, but only 5 of 41 patients (12,2 %), who completed therapy, succumbed to progression and 10 of 41 are continuously progressive. Since observation time even is longest for the children having completed chemotherapy, this differentiation is not biased by different lengths of time since entering the study.

Table 20: Further course of patients after relapse or progression:

- **Less than 4 months from starting chemotherapy (29 patients):**

**Status**	**n**	**Follow-up (months)**
**Alive with SD**	**12**	**Median follow-up = 46.1 months**
After RT	6	+ 17,9, +40,7, + 44,9, +47,3, +70,5, +118
After surgery followed by RT	1	+ 59,1
After CT followed by RT (for further PD)	3	+ 13,6, + 68,3, + 87,2
After RT followed by CT	1	+ 19,8
After 2 surgeries	1	+ 12,3
**Alive with PD**	**3**	**Median follow-up = 19,2 months**
After RT	3	+ 17,7, + 19,2, + 32,2
**Dead of disease**	**12**	
After RT	4	9, 10,4, 23,3, 32,6
After surgery only	1	40
No further therapy	5	3,3, 4.6, 18, 21,4, 118
After surgery followed by CT	1	16,2
After CT	1	28,9
**Lost to follow-up**	**2**	+ 33,1, + 32,6

- **Between 4 and 6 months from starting chemotherapy (5 patients):**

**Status**	**n**	**Follow-up (months)**
**Alive with no evidence of disease**	**1**	
After CT followed by 2 surgeries	1	+ 46,8
**Alive with stable disease**	**1**	
After partial resection	1	+ 50.1
**Alive with PD**	**1**	
Not known how presently treated	1	+ 16.4
**Dead of disease**	**2**	
After RT followed by CT	1	15,3
No further therapy	1	6,6

- **Between 6 and 12 months from starting chemotherapy (9 patients):**

**Status**	**n**	**Follow-up (months)**
**Alive with SD**	**5**	**Median follow-up = 30.8 months**
After RT	3	+ 22,0, + 30,8, + 33,7
After further CT	2	+ 24,1, + 40,8
**Alivewith PD**	**2**	
After CT followed by RT	1	+ 27,9
After only a biopsy	1	+ 33,7
**Dead of disease**	**2**	
After surgery followed by CT	1	15,2
No further therapy	1	7,9

- **More than 12 months from starting chemotherapy (41 patients):**

**Status**	**n**	**Follow-up (months)**
**Alive with no evidence of disease**	**2**	
After surgery	1	+ 23,3
After RT	1	+ 57,4
**Alive with responding disease on therapy**	**1**	
During other CT	1	+34.6m
**Alive with SD**	**22**	**Median follow-up = 55 months**
After RT	12	+ 31,3, + 35,6, + 45,2, + 47,8, + 48,3, + 54,5, + 55,4, + 62, + 67,8, + 73,5, + 88,4, + 107,8
After further CT	2	+ 37,8, + 69,2
After further CT followed by RT	1	+ 52,3
After surgery	4	+ 35,7, + 37,4, + 64, + 93,1
After surgery followed by CT	1	+ 79,7
No further therapy	2	+ 27,9, + 63,5
**Alive with PD**	**10**	**Median follow-up = 49.1 months**
After surgery	2	+ 49,4, +64
After 2 surgeries	1	+ 44,8
After surgery followed by RT	1	+ 32,4
No further therapy	3	+ 24,6, + 48,7, + 75,4
After RT	1	+ 43,9
After RT followed by CT and RT	1	+ 112,5
After CT	1	+ 71,2
**Alive n.o.s.**	**1**	
After CT	1	+ 38,3
**Dead of disease**	**5**	
After surgery	3	29,0, 37,7, 66,8
After CT followed by RT	1	38,4
No further therapy	1	28,5

Although 39,3 % of all children suffering from progression had been primarily refractory to chemotherapy, there were as many (36,9 %) who had had a complete response or tumor volume reduction.

Table 21: Distribution of events by type of response to primary chemotherapy:

	No.
Complete Response	1
Tumor Volume Reduction	30
Stable disease	20
Progressive Disease	31
Not evaluable, interrupted CT after 8 days of therapy	1
Not evaluable, refusal to continue treatment	1
Total	84

Radiotherapy as treatment post-event

32 children (38%) after having suffered of an event ended-up receiving RT immediately; 8 were treated with secondary chemotherapy (+ surgery in one case and + RT in six cases after further PD), 14 were treated with surgery alone (+ RT in two cases after further PD and + CT in two cases).1 child had received a new biopsy only, 14 were merely observed (+RT in one case after further PD), 2 are lost to follow-up and for one child it is too early for evaluation. As a whole 41 children were irradiated, for 23 of whom detailed information of the treatment were reported.
The time interval between date of beginning of CT and of RT for 37 patiens was 22,2 months median time with a range of 1.3 to 67.6 m. Age at start of chemotherapy for the irradiated children (41 children) had been 54,3 months (range 3,5 – 164,8 m) and their age at the start of radiotherapy (37 patients) was 84,0 months (7,2 – 167,3 months).

Current status following progression/relapse

Obviously, an event after chemotherapy does not necessarily predict a fatal outcome as 43/84 children are alive without evidence of disease or with stable disease on or off therapy. For those alive the time interval between event and last follow-up (62 pts.) ranges from 12.3 to 118.0 months with a median of 45.1 months

Table 22: Current status of 84 children following relapse or progression

		No.	%
Alive	without evidence of disease off therapy	3	3.6
	with stable disease on therapy	1	1.2
	with stable disease off therapy	39	46.4
	with progressive disease	16	19.0
	not otherwise specified	1	1.2
Dead		22	26.2
Lost to follow-up		2	2.4
Total		84	100

4.2.3. Toxicity

Detailed information on the haematological and organ toxicity of the combination Carboplatin and Vincristine was not centrally recorded. The allergy to Carboplatin seems to be a major limiting factor for full compliance to the protocol. As a whole 43 (21.1%) of the 204 patients had allergic reactions to Carboplatin, at a time interval between the beginning of chemotherapy and "allergy" ranging from 1 to 52 week (median 33 weeks). However, this could be an underestimation of the real incidence of the problem; since among the Italian patients 17 out of 47 children (36.2%) actually manifested allergic reactions to Carboplatin at approximately the same time interval between starting CT and "allergy". The further treatment for the 43 patients who had allergic reactions to Carboplatin was: 24 with different CT, 2 with VCR/Carboplatin but with reduced dose of Carboplatin, 4 with only VCR, and 13 no further therapy. The outcome of these patients is listed in table 23.

Table 23 Current status of 43 children following Carboplatin allergy:

		No.	%
Alive	without evidence of disease off therapy	1	2.3
	with stable disease on therapy	1	2.3
	with stable disease off therapy	39	90.8
	with progressive disease	1	2.3
Dead		1	2.3
Total		43	100

4.3. Main conclusions

The SIOP-LGG 1 study must be considered a feasibility study aiming:

a) to demonstrate the actual recruitment rate of children affected by LGG eligible to chemotherapy by the pediatric (neuro-) oncology groups in Europe,
b) to get pediatric oncology used to treat these patients with chemotherapy,
c) to demonstrate advantages and limit of the chemotherapy treatment, trying to duplicate the results produced by the concurrent studies run by the CCSG in U.S.A.,
d) to learn more about the "natural history "of LGG treated with chemotherapy,
e) to pilot a data collection process whith each participating nation collecting own data and then transferring them into a common database.

With respect to these aims it can be stated that:

a) the recruitment rate was representative, but not complete: it is expected to grow,
b) treatment centers gained expertise on how to treat these children,
c) the effect of single dose Carboplatin combined with Vincristin chemotherapy in terms of response and survival is comparable to the results shown by the CCSG experience,
d) the excess of early events (34.5% of the events occurred less than 4 months from diagnosis) calls for modification of therapy,
e) the allergy to Carboplatin seems to be a major problem for proceeding with the same regimen,
f) the data collection process seem to be working effectively.

Inadequate data was collected concerning the quality of care or on the health status of these patients in relationship to the treatment received.

5. Changes within SIOP-LGG 2004 as compared to SIOP-LGG 1996

As in the previous SIOP study, the protocol offers a comprehensive strategy for all children up to an age of 18 years with glial tumors of low grade malignancy. But treatment recommendations differ according to tumor localization and the presence or absence of Neurofibromatosis NF I.

In the previous study the age of 5 years was empirically chosen as the cut-off age for recommending chemotherapy or radiotherapy as non-surgical therapy for symptomatic or progressive tumors. In the light of more data, which have been accumulated on the effect of chemotherapy on low grade glioma, it is possible to extend this cut-off to the age of 8 years. According to individual decisions even older children may receive primary chemotherapy.

Disseminated disease is recorded, but children are treated according to their main therapy subgroup determined by NF I-status and tumor site. Primary chemotherapy is suggested.

A randomized study question is asked for children without NF I stratified for primary tumor location at either the supratentorial midline or the cerebral hemispheres, the cerebellum, the caudal brainstem and the spinal canal, if they are to receive chemotherapy.

Thus changes for the newly defined patient subgroups are the following:

1. Supratentorial midline tumors	No NF I	Age 0 to 18 years

1. Non surgical therapy is stratified for age: young = under 8 years, older = 8 years and older.
2. Chemotherapy group
   Duration of chemotherapy is extended to 18 months for all children.
   Induction therapy is randomized:
   - Standard induction: Vincristin and Carboplatin
   - Intensified induction: Vincristin, Carboplatin and Etoposide
3. Radiotherapy group
   Apply highly focussed radiation at standard dose and fractionation.
   Record and monitor the integral dose to tumor and normal tissue.
   Assess impact of craniospinal irradiation in disseminated disease.
   Assess response of tumor and clinical symptoms.

2. Tumors of all other locations	No NF I	Age 0 to 18 years

1. Non surgical therapy is stratified for age: young = under 8 years, older = 8 years and older.
2. Strategies are adopted to consider the specific conditions for tumor location in the spinal canal, cerebral hemispheres, cerebellum or caudal brain stem.
3. Chemotherapy group
   Duration of chemotherapy is extended to 18 months for all children.

Induction therapy is randomized:
- Standard induction: Vincristin and Carboplatin
- Intensified induction: Vincristin, Carboplatin and Etoposide

4. Radiotherapy group
   Apply highly focussed radiation at standard dose and fractionation.
   Record and monitor the integral dose to tumor and normal tissue.
   Assess impact of craniospinal irradiation in disseminated disease.
   Assess response of tumor and clinical symptoms.

3. Tumors of all locations	NF I present	Age 0 to 18 years

1. All children shall receive primary chemotherapy as non-surgical therapy
2. Chemotherapy group
   Duration of chemotherapy is extended to 18 months for all children.
   All children receive Standard induction and Consolidation with Vincristin and Carboplatin.
   Upon progression successive chemotherapy treatments should be investigated.
3. Radiotherapy
   Primary radiotherapy is <u>not</u> indicated for children with NF I, except in individual patients with optic nerve gliomas restricted to the intraorbital portion of the optic nerve or in the case of progression following (multiple) chemotherapy interventions.

6. Aims of the study SIOP-LGG 2004

6.1. Improve response and progression / event free and overall survival

It is envisaged to arrive at high treatment response rates and improved event free and progression free survival rates for children and adolescents with a central nervous system low grade glioma by:

- applying stringent criteria for diagnostic work-up, guidelines for surgical procedures and clear indications to start non-surgical therapy
- offering an individualized sequence of treatment modalities according to established guidelines for subgroups defined by tumor location and the presence or absence of Neurofibromatosis NF I
- prolonging chemotherapy for all children stratified to receive chemotherapy

Comparison will be made to preceding national and international studies.

6.2. Reduced late effects and improvement of the quality of life at short and long term

It is envisaged that late effects of the central nervous system following radiotherapy will be reduced and the health status and quality of life of long term survivors be improved by:

- avoiding radiotherapy for a larger proportion of young children by raising the cut-off age for primary chemotherapy in non-NF I patients and
- offering primary chemotherapy to all children affected by NF I irrespective of age
- using modern equipment for treatment planning and stereotactic or conformal radiotherapy arriving at reduced doses to organs at risk for children stratified to receive radiotherapy

No prospective or comparative studies evaluating this aspect exist. Short and long term side effects of chemotherapy will be monitored and their impact upon the development of the children be evaluated.
Improvement of progression free survival following initial therapy is only a surrogate parameter of an improvement of the quality of life. The study will try to evaluate whether improvements of PFS translate into quality of life.

6.3. Improvement of individualized patient management

Histopathologic diagnosis, neuroradiologic diagnosis and neuroradiologic indication for therapy shall be centrally reviewed to assure correct assignment of patients to treatment arms.

It is envisaged that the prognosis will be improved by quality control of radiotherapy and chemotherapy as well as by individualized counseling for surgical and non-surgical procedures.
Careful follow-up investigations of the impact of treatment on the development of the children will be carried through.

6.4. Evaluation of prognostic factors

Prognostic factors other than the extent of resection for progression free survival have not been firmly established for low grade glioma. Therefore, factors that might be important for prognosis shall be evaluated prospectively. If their impact can be established reliably, they will serve for a more risk adapted stratification within the framework of a successive trial.

Pathology Tumor type and WHO grade
Markers of proliferation (e.g. Ki 67 / MIB-1)
Molecular-pathologic markers (e.g. p 53 mutation)

Tumor Tumor size preoperatively (Product of the two largest diameters in cm)
Tumor size postoperatively (Product of the two largest diameters in cm)
Extent of surgery
Localization and extent within the supratentorial midline for visual pathway gliomas (Dodge classification II, III)

Dissemination primary/secondary
Type and extent of dissemination

Symptoms Severe, visual or neurologic symptoms relevant for the decision to start non-surgical therapy will be described according to their presence or absence:
Visual symptoms
Neurologic symptoms
Increased intracranial pressure
Diencephalic syndrome

Age < 8 and ≥ 8 years
(To investigate the „young" and „older" age groups)
< 1 year, 1 to 4, 5 to 10, > 10 years
(Comparison to previous trial)
Continuous variable

Sex male / female

Observation time following diagnosis before starting therapy

Therapy related factors: Type of
Induction therapy (I or II)
Response at week 24
Therapy modifications due to allergy

7. Study questions SIOP LGG 2004

7.1. Children not affected by NF I with tumors of all sites (1. the supratentorial midline, 2. all other sites)

Main study question:
To investigate, if adding Etoposide (VP 16) to the standard induction treatment of Carboplatin and Vincristin will lead to a different progression free survival than the induction treatment with Carboplatin and Vincristin only.

Secondary study questions:
To investigate, if the radiological tumor response at week 24 depends upon the type of induction therapy with either standard induction with Vincristin and Carboplatin or intensified induction with Vincristin, Carboplatin and Etoposide.

To investigate, if adding Etoposide (VP 16) to the standard induction treatment of Carboplatin and Vincristin will lead to a different event free survival than the induction treatment with Carboplatin and Vincristin only.

To investigate, if adding Etoposide (VP 16) to the standard induction treatment of Carboplatin and Vincristin will lead to a different overall survival than the induction treatment with Carboplatin and Vincristin only.

The study questions will be analysed for group 1 and 2 together. For explorative reasons these questions will also be analysed separately for the two groups.

7.2. Children affected by NF I with tumors of all sites

For this group of children the study is a documentation study, yet the data shall be compared to the historical series of SIOP - LGG 1.

To investigate, if the prolonged (18 months) chemotherapy with Carboplatin and Vincristin leads to a different progression free survival than the historical treatment with a shorter (12 months) chemotherapy or radiotherapy.

To investigate, if the prolonged chemotherapy with Carboplatin and Vincristin leads to a different event free survival than the historical treatment with a shorter chemotherapy or radiotherapy.

To investigate, if the prolonged chemotherapy with Carboplatin and Vincristin leads to a different overall survival than the historical treatment with a shorter chemotherapy or radiotherapy.

8. Investigations at diagnosis and during follow-up SIOP LGG 2004

8.1. Primary tumor diagnosis – preoperatively

- **Essential investigations:**

1. Neurologic examination

2. Ophthalmologic examination: fundoscopy, if possible visual acuity and visual fields in supratentorial midline tumors (see section 8.6.).

3. Cranial MRI without and with Gadolinium enhancement (see section 8.5.)
(MRI must be done in order to enter patients into the trial, CT-scan only cannot be accepted. CT-scan should only be done, if MRI is not available)

4. Spinal MRI without and with Gadolinium enhancement – if indicated (see section 8.5.)
Indications for a spinal MRI in low grade glioma are:
   1. Multiple lesions demonstrated on cranial MRI
   2. Spinal (cervical) lesions seen on cranial MRI
   3. Clinical symptoms that might relate to spinal lesions

5. General preoperative diagnostic procedures:
   - complete physical examination including anthropometric measurements, assessment of NF I status by thorough skin examination, symptoms of diencephalic syndrome or other symptoms
   - preoperative laboratory investigations: full blood cell count and differential, urea, serum-creatinine, electrolytes, Magnesium, ALT/AST, Bilirubin
   - chest X-ray, ECG/UCG

- **Recommended investigations:**

(pre- or postoperatively, depending on the condition of the child at diagnosis and if relevant)

1. Neurophysiologic investigations
   - EEG
   - Extended Ophthalmologic investigation (see section 8.6.)
   - Visual evoked potential (if available)
   - Audiogram – pure tone where possible (age 3 years or over), otherwise free field testing or otoacustic emissions

2. Neuropsychologic investigations (see section 8.7.)

3. Neuroendocrine investigations
   - Base line endocrinologic investigation (see section 8.4.).
   - Tumor-induced primary hypothalamo-pituitary dysfunction is rare in low grade glioma even in case of chiasmatic-hypothalomic localisation. It should be investigated however in all children with diencephalic syndrome, short stature or relevant clinical findings at diagnosis.

- Pregnancy has to be excluded by HCG-determination in fertile adolescent girls.

4. Health status, quality of life (see section 8.7.).

8.2. Postoperative diagnostic procedures

1. Neurologic examination

2. Cranial MRI without and with Gadolinium enhancement within 24 to 48 (maximum 72) hours postoperatively (see: section 8.5.)
   (CT-scan only, if MRI is not available)

3. Spinal MRI without and with Gadolinium enhancement – only if not done preoperatively, yet indicated (see section 8.5.)

4. Lumbar CSF cytology – if indicated (see section 8.5 and 12.4).
   Lumbar CSF sampling will be performed only, if imaging procedures demonstrate disseminated disease.
   The purpose of CSF sampling is to investigate the presence of CSF neoplastic cells following a process of centrifugation. Intracranial hypertension should be excluded, so that the patient is not put at risk through the performance of a spinal tap.
   The presence of neoplastic cells in the CSF is regarded as stage M 1 (see 16.1. for tumor staging).

   Protein level in the CSF should be recorded in a parallel fashion to follow the patients during treatment.

8.3. Histopathologic diagnosis

The acquisition of histological samples for tissue diagnosis is strongly recommended in all cases. Children with NF1 and hypothalamic/visual pathway glioma and children without NF I, whose tumor shows unequivocal contiguous involvement of the visual pathways (see section 8.5.), may enter the study without biopsy.

NEUROPATHOLOGIC GUIDELINES

The purpose of histological assessment in these tumors is to:

- confirm the presence of tumors corresponding to grade 1 or 2 (WHO) and to exclude anaplastic gliomas and glioblastomas.
- provide a standardised classification, which will facilitate detailed clinicopathological studies, with particular reference to neuroradiological findings.
- investigate the clinical significance of proliferation indices (as determined by immunocytochemistry) in the low grade gliomas of childhood.

It is recognised that the exact classification and histogenetical typing as well as the grading of low grade gliomas in childhood may present difficulties. Therefore it is undispensible that tumor material of all children, registered within the SIOP - LGG trial be classified centrally. A panel of neuropathologists will assess these tumors. Facilities for "fast- track" pathology review will be provided for cases of particular diagnostic difficulty or uncertainty.

Children entering the randomised chemotherapy trial must have had central review of their biopsy specimens, if obtained.

From each patient representative, paraffin embedded tissue and the documentation form should be sent to the national brain tumor reference center. All material will be returned to the sender following handling and final statement, except for proof-slides that will be kept. Central pathologic assessment includes conventional histologic and immunohistochemical staining. In case of unusual and diagnostically difficult tumors, members of the pathology panel and other experts will be consulted. All findings will be documented on a report form designed for this study and sent back to the local pathologist or neuropathologist as well as to the national/international study data center. Standardised histopathological parameter of each patient will be stored in a data base (German Brain Tumor Reference Center: Data base: Filemaker Pro). Study material and the data base will be available for all participating colleagues.
The criteria for classification are based upon the WHO classification in its current, revised version including the grading system (Kleihues 2000).

National brain tumor reference centers:

Germany:
Hirntumorreferenzzentrum
Prof. Dr. T. Pietsch
Institut fuer Neuropathologie
Sigmund-Freud-Strasse 25
D 53105 Bonn

Italy
Prof. Felice Giangaspero
Institute of Anatomical Pathology
Bufalini Hospital
Via Ghirotti 286
I 47023 Cesena

United Kingdom:
James Ironside, Edinburgh

France:
Marie-Madeleine Ruchoux, Lille
Anne Jouvet, Lyon
Dominique Figarella Branger, Marseille
Arielle Lelouch-Tubiana, Paris

NEUROPATHOLOGY – LABORATORY GUIDELINES

Besides warranting a uniform neuropathologic diagnosis, a series of cytologic, histologic and immunophenotypic parameters shall be raised and documented from the materials sent in. A goal of these investigations is to identify parameters of prognostic significance.

Conventional histology
All biopsy specimens for histological evaluation should be fixed in formalin (preferably 10% neutral buffered formalin) and embedded into paraffin wax. Since it is anticipated that many of the histological specimens for this study will be derived from stereotactic biopsy specimens, the material for review will sometimes be limited. The material requested for histological review consists of:

- 4 unstained paraffin embedded sections 5-6 µm in thickness and cut onto poly-l-lysine coated slides (or equivalent) to facilitate immunocytochemistry.
- The original paraffin block (if possible)
- The pathology report from the originating hospital, along with patient details including the age of the patient and site of biopsy.

Investigations to be performed:

1. Staining with haematoxylin and eosin for standard morphological assessment.

2. Immunocytochemistry: glial fibrillary acidic protein, others as needed.

3. Immunocytochemistery of the cellular proliferation rate of the tumor. (e.g. by means of an antibody directed against an epitope of the Ki67/MIB-1-antigen. This will be performed following microwave antigen retrieval.)

4. Immunohistochemical investigation of differentiation antigens.

5. Evaluation of characteristic histological parameters (certain growth patterns, patters of vascularisation, infiltration with inflammatory cells)

Results of this histological review and other investigations will be sent to the submitting pathologist in all cases. Proof-slides submitted into study will be retained for purposes of central review at least until the study is completed.

Scientific investigations

Knowledge concerning molecular pathogenesis of pediatric malignant glioma is scant as compared to the more frequent adult glioma. However, a large proportion of molecular investigations is only possible with unfixed, shock-frozen material.
Therefore additional investigations will be done for limited numbers of patients only, although an increasing number of investigations may be performed on paraffin embedded tissue. It is an aim of the study to obtain fresh frozen material for molecularpathologic studies from as many patients as possible.

In Germany, throughout the recent years the competence network "pediatric oncology" has established a structure facilitating the asservation, the mailing and the storage of tumor probes. Manuals for handling, tumor boxes for shipment and tumor banks for storage are available. The brain tumor bank works under the supervision of an independant scientific council. Material can be made available for scientific investigations following a formalized proposal. The aim of these investigations is to identify prognostic factors and to define the molecular pathogenisis of gliomas.

Patients/parents have to consent to the use of tumor material for these investigations, an appropriate explanation is included into the forms for study participation. Tumor material should be prepared in a standardised manner together with the local pathologist/neuropathologist and sent to the tumor bank accompanied by the documentation forms, which are available at the pediatric oncology units:

Germany (for German patients only):
Hirntumorbank des Kompetenznetzes Paediatrische Onkologie
Prof. Dr. Torsten Pietsch
Institut fuer Neuropathologie
Universitätsklinikum Bonn
Sigmund-Freud-Strasse 25
D 53105 Bonn

8.4. Status assessment

8.4.1. Status evaluation during chemotherapy and early follow-up

1. History at every visit.

2. Complete physical and neurological examination, including anthropometric measurements.

3. Laboratory data: Full blood cell count and differential; urea, serum creatinine, electrolytes, Mg^{++} and Ca^{++}, ALT/AST; Bilirubin.

4. Cranial contrast enhanced MRI
   For children receiving chemotherapy the relevant time points for assessment of cranial MRI are :

Time 1	at diagnosis
Time 2	where applicable after observation to demonstrate progression or measure changes at the time of start of chemotherapy
Time 3	six months after commencement of chemotherapy
Time 4	twelve months after commencement of chemotherapy
Time 5	eighteen months after commencement / at the end of chemotherapy
Time 6	scan of those obtained at six-monthly intervals until progression

5. Spinal contrast enhanced MRI, if previously pathologic at the same time points as 4.

Central review: For assessing response to chemotherapy in the randomised arms of the chemotherapy study all relevant scans (as defined in section 8.5.) have to be sent in for review during the pre-treatment and treatment periods to the national radiodiagnostic reference center (see section 8.5.).

6. CSF sampling to be performed only in case of disseminated disease and if previously positive

7. Ophtalmological examination: every 3 months during chemotherapy (and at least every 6 months during follow-up) (see section 8.6.).

8. Glomerular filtration rate (GFR) as measured by Creatinin and/or 51 Cr-EDTA clearance
   - see guidelines for chemotherapy (14.2.4.)

9. Audiogram – pure tone where possible (age 3 years or over), otherwise free field testing or otoacustic emissions
   - see guidelines for chemotherapy (14.2.4.)

10. Endocrine investigation as detailed below

Minimum requirements for patient follow-up during the chemotherapy study are listed in Addendum 21.13.1.

8.4.2. Follow-up investigations without therapy or following chemo- or radiotherapy

1. Complete physical and neurological examination, including anthropometric measurements, and history.

2. Laboratory data: Full blood cell count and differential; urea, serum creatinine, electrolytes, Mg^{++} and Ca^{++}, ALT/AST; Bilirubin
   - For those having had chemotherapy: every 6 months during the 1st and 2nd year. Later only, if indicated.

3. Brain and / or Spine: Contrast enhanced MRI
   (Spine: in case of evidence of tumor dissemination at the Gd-enhanced cerebral MRI)

4. Ophtalmological examination (see section 8.6.)

5. Glomerular filtration rate (GFR) - for those having had chemotherapy

6. Audiogram – pure tone when possible (age 3 years and over), otherwise free field testing or otoacustic emissions - for those having had chemo- and/or radiotherapy, or where the tumor affects the auditory pathways.

7. Endocrine investigations - as detailed on next page.

Table 25: Follow-up investigations.

	**First, second and third year**	**Fourth and fifth year**	**Sixth to tenth year**
Physical examination and neurological examination, including anthropometric measurements;	Every 3 months	Every 6 months	Annually
Ophthalmological examination	Year 1: 3 monthly<br>Year 2: 3-6 monthly<br>Year 3: 6 monthly	Every 6-12 months	Annually, yet six-monthly in OPG
Contrast enhanced cerebral and spinal (if indicated) MRI	Every 6 months	Every 6 months	Annually
Audiogram – pure tone where possible age 3 years or over, otherwise free field testing or otoacustic emissions	Every 6 months	Not indicated if previously repetitively normal	---
Glomerular filtration rate (GFR)	6 months after CT, then yearly, if not indicated otherwise	Not indicated if previously repetitively normal	---
Endocrinologic investigation and, if indicated, bone age and hypothalamic-pituitary functioning test	Yearly, if not indicated otherwise	As indicated by stage of growth and puberty and previous chemo- or radiotherapy	As indicated by stage of growth and puberty and previous chemo- or radiotherapy

8.4.3. Extended endocrine investigations and monitoring of growth

Depending upon tumor location, the extent of surgery and the effects of non-surgical therapy children may suffer from complex endocrine sequelae. It is essential that an experienced pediatric endocrinologist is involved in the care of these patients. These guidelines are intended to help the oncologist, but the endocrinologist will be needed to advise appropriate tests and their interpretation, and decide upon treatment.

1. Anthropometric Data

At diagnosis:	Mother's height, father's height, gestation (weeks), birth weight (kg).
All assessment points:	Decimal age, standing height, sitting height, weight<br>(These results should be plotted on standard growth charts.)<br>occipitofrontal head circumference (in the young)

2. Pubertal/reproductive Data

All assessment points:	Tanner score for breast development, pubic and axillary hair and genital development (testes volume in ml right and left), record date of menarche and of last menstrual period.

3. Biochemical Data

All assessments:<br>(until growth complete)	LH (IU/ml), FSH (IU/ml), Oestradiol (pmol/l), Testosterone (nmol/l), free T4 and T3 (nmol/l), TSH (mU/l).<br>IGF I and IGF-BP 3 (esp., if body measurements are at or below 3rd percentile)
At growth retardation:	Bone age (esp., if body measurements are at or below 3rd percentile)<br>Growth hormone testing including GnRH, TRH, and measurements of cortisol<br>24 hour urinary Cortisol

If the patient has thirst polyuria (especially at night), persistent or recurrent hypernatraemia or other symptoms suggestive of diabetes insipidus: Water deprivation test with measurement of urine and plasma osmolality.

4. Timing of investigation

At diagnosis investigation should take place before or after surgery, but before radiotherapy and chemotherapy, and preferably the patient should not be receiving dexamethasone.

Table 26: Timing of investigations to monitor endocrine functions

	**Time Points**	**Timing of Investigation**
Diagnosis:	before or after surgery	
Follow-up:		
Observation group	until growth is completed	annually, but more often, if clinically indicated
	after growth is complete	3 (to 5) yearly assessments
Treatment: Chemotherapy	during CT	+6, +12, +18 months / end of CT
	after therapy until growth is complete	annually, but more often, if clinically indicated
	after growth is complete	3 (to 5) yearly assessments
Treatment: Radiotherapy	after completion of RT	at end of radiotherapy one year after end of RT (obligatory)
	until growth is complete	annually, but more often, if clinically indicated
	after growth is completed	annually

5. Documentation

For documentation use Endocrine status forms (Status after registration and post treatment/during follow-up) from Addendum 21.13.5..

8.5. Guidelines for Neuroradiologic assessment (Dr. Warmuth-Metz)

MRI has become the preferred modality for the evaluation of pediatric brain tumors because of its non-ionising nature and superior spatial and contrast resolution. In addition, the multiplanar imaging capabilities of MRI are very valuable in defining the extent and infiltration of complex tumors. The evaluation of primary spinal tumors and CSF-dissemination of CNS tumors by MRI has replaced CT-scan assisted myelography (CAM), although if MRI is not available or there are specific contraindications to MRI (such as metallic foreign bodies) CAM can be used as a substitute. If postoperative examination can only be done by CT-scan (because of local availability or access to MR scanning) preoperative CT scanning should be undertaken additionally to enable better evaluation of the results of surgery, as the two different modalities cannot be directly compared.

8.5.1. MRI

- **Minimum requirements for cranial MRI**

Since MRI imaging is performed at many institutions, the following minimum requirements are defined:

⇨ The standard examination should consist of a T2-weighted SE dual echo sequence preferably in the axial plane. The short echo T2-sequence may be substituted by a FLAIR-sequence. The slice thickness should not exceed (5-) 7 mm and the slice factor should not exceed 20%.

⇨ A T1-weighted sequence, preferably in the axial plane, should be obtained followed by the same scan sequence after intravenous contrast administration. Additional T1-weighted post-contrast sequences in the coronal and sagittal plane are very helpful. In small or irregular tumors slice thickness should be correspondingly small.
Conventional spin echo-techniques are preferred to all kinds of gradient echo sequences, because flow-related enhancement of cerebral vessels by gradient echo- sequences may cause problems in differentiation from meningeal enhancement and the extent and degree of enhancement may be of a lesser order than conventional T1-weighted imaging.

⇨ On all images a ruler must be shown.

⇨ Generally, follow-up scanning should be comparable with prior examinations as it can be very hard to make direct comparisons between studies using different imaging planes and machines.

- **Application of contrast media**

The administration of Gadolinium should follow the general rule of a slow intravenous injection of 0.1mmol/kg bodyweight Gadolinium. The post-contrast scan should not be started until after the full injection of the contrast medium.
Due to the availability of different Gd-containing contrast-media it should be observed to always apply equivalent amounts of Gadolinium.

- **Minimum requirements for spinal MRI (in case of CSF dissemination)**

Indications for a spinal MRI in low grade glioma are:

- Multiple lesions demonstrated on cranial MRI
- Spinal (cervical) lesions seen on cranial MRI
- Clinical symptoms that might relate to spinal lesions

⇨The minimum requirement is a post-contrast T1-weighted sagittal sequence of the entire spinal canal (down to at least S2 as the thecal sac usually ends there, but may be even longer). In many cases the normal enhancement of intradural veins covering the conus and distal cord can be mistaken as pathological leptomeningeal enhancement if only sagittal scans are available. T1-weighted post-contrast imaging of this region in axial direction is often necessary and helpful in evaluating this region.

⇨T2-weighted sequences are rarely required for the evaluation of CSF-dissemination. If necessary, they can be added after the T1-weighted post-contrast MRI has been acquired, without problems associated with artefacts. Generally fast spin echo sequences are preferred because they show less CSF-pulsation artefacts.

⇨Metastatic disease on imaging is defined as the presence of nodular leptomeningeal and/or sub-ependymal enhancing nodules or of a diffuse leptomeningeal enhancement.

- **Post-operative radiologic investigation of primary tumor**

Scanning should be undertaken within 48 hours following surgery to minimise the effects of reactive post-surgical enhancement. Every effort should be made to establish whether foreign material such as surgical or chemotherapeutic wafers was placed in the surgical bed. MRI is the imaging modality of choice. The same sequence parameters should be employed as in the pre-operative diagnostic study to facilitate comparison.
CT-scan is accepted in case MRI is not available and should be performed without and with contrast medium as indicated prior to surgery within a time frame of 48 hours (max. 72 hours post surgery).

- **Spinal MRI after surgery**

If preoperative imaging of the spinal canal in case of a possibly disseminating tumor was not performed, it can be done at any convenient time point after surgery. However, after surgery of the posterior fossa investigators have to be aware of unspecific subdural enhancement of various degrees within the spinal canal. This rarely impedes the exact definition of meningeal dissemination, but must not be misinterpreted for intradural enhancement as a consequence of dissemination. Unspecific enhancement is usually most extensive immediately after surgery and diminishes thereafter.

8.5.2. CT-Scan

- **Requirements for cranial CT-scan (in case MRI is not available or contraindicated)**

The gantry angulation should be adjusted to minimise direct irradiation of the lens of the eye. At least 4 to 5 mm thick contiguous sections should cover the posterior fossa and base of the skull. In the supratentorial compartment 8 to 10 mm thick section are adequate. A spiral

scanning technique should only be used, if secondary reconstruction in the coronal or sagittal plane is planned, because irradiation doses are higher than with sequential imaging. The slice thickness should be approximately 1mm.
Ideally identical slices should be obtained after slow intravenous injection of iodinated contrast medium (up to 2 ml/kg bodyweight of 300mg/ml Iodine concentration).

- **Timing of CT-Scan-Investigations**

If MRI is not available and pre- and postoperative investigations have to be performed using CT-scans, their timing should correspond to the appropriate timing of MRI investigations and use of contrast media.

8.5.3. Imaging requirements for patients recruited, if no histological confirmation of a presumed low grade glioma is planned

If on MRI the tumor is clearly arising from the optic nerve, tract and chiasm and is not confined to only one part of this pathway no additional imaging to MRI is required, especially if the patient is affected by NF I.

If on MRI the tumor is arising from the chiasmal region without contiguous involvement of other structures of the optic pathways, various different processes such as germinoma or craniopharyngioma may mimic a hypothalamic glioma. Differentiation according to MRI signal intensities may not be possible.
As craniopharyngiomas are usually at least partly calcified, CT scanning can be helpful for differential diagnosis as calcifications are not reliably demonstrated by MRI.
In addition, since germinomas are usually iso- to hyperdense due to their intrinsic high cellularity, pre-contrast CT-scan imaging (only covering the tumor region) can be helpful in assessing a suprasellar mass. At present it is not yet clear, if diffusion weighted MRI is able to substitute CT-scan in the assessment of the cellular density of germinomas.

8.5.4. Central radiologic review

Central radiologic review will be organized within the participating national groups. The national radiologic reference centers will follow the guidelines as detailed within the protocol.

The images of any case of tumor not biopsied or resected for diagnosis **should** be seen by a dedicated neuroradiologist and sent in for central review.

The images **must** be sent in for central radiologic review in all children entering the randomised arm of the chemotherapy trial.

For assessing response to chemotherapy in the randomised arms of the chemotherapy study all relevant scans have to be sent in for review during the pre-treatment and treatment periods.

To answer the question of response distribution at week 24 following induction treatment for children entering the chemotherapy arm of the study, it is necessary to review the relevant scans centrally (national radiodiagnostic reference center). Additionally it shall be assessed,

when the "best response" throughout treatment is reached, so scans shall be performed at 6-monthly intervals. Qualitative changes of contrast enhancement will be described and correlated with response.

Definitions of "relevant time points" for the central radiologic review of radiodiagnostic images for children participating in the chemotherapy trial:

Time 1	at diagnosis
Time 2	where applicable after observation to demonstrate progression or measure changes at the time of start of chemotherapy
Time 3	six months after commencement of chemotherapy
Time 4	twelve months after commencement of chemotherapy
Time 5	eighteen months after commencement / at the end of chemotherapy
Time 6	"progression scan": scan during or after therapy showing progression

Scans will need to be centrally reviewed from time-points 1 and 2 in order to validate radiological criteria for tumor progression and to confirm radiological or diagnostic imaging criteria.

At time point 3 scans have to be reviewed to validate the response and assess the distribution of response at week 24 following induction treatment.

At time points 3 to 5 central radiologic review needs to take place to validate the best response during treatment and for comparison against subsequent scans (time point 6), where progression was deemed to have occurred in order to validate the time of progression.

In all cases of neuroradiologic progression during observation/before starting treatment and during or following therapy review should confirm that the criteria for progressive disease have been met (see section 16.3.). Minimal or transient changes of tumor size should not be termed progressive disease. All comparisons of tumor size have to be made

- to the size at diagnosis for those being observed,
- to the size at start of therapy to assess treatment response at the defined time points,
- to the size at "best response" for subsequent assessment of tumor status for those having been treated.

Table 27: Minimum required sequences for central radiologic assessment:

	Cranial MRI preoperatively	Cranial MRI postoperatively 24-48 (-72) hrs and follow-up	Spinal MRI
PD or Flair	X	X	-
T 2 axial	X	X	-
T1 without Gd	X (axial)	X (axial)	(X) (sagittal)
T1 with Gd	X (axial)	X (axial)	X (sagittal)
T1 with Gd (additional planes)	X (coronal or sagittal)	X (coronal or sagittal)	X (axial in areas of suspicious enhancement)

8.6. Ophthalmological assessment

Introduction
Children who have been diagnosed as having optic pathway and hypothalamic gliomata, either with or without Neurofibromatosis Type I, require a regular and structured ophthalmic assessment. No prospective study has tested the various types of assessment of visual function. A decision of whether to commence chemotherapy or radiotherapy is often based on optic nerve function, although there has never been a consensus regarding a structured approach to this testing.

All ophthalmic centres linked to oncology centres participating in the low grade glioma Trial would be expected to perform a standard set of tests of visual function. It is hoped that by performing these tests in a structured prospective manner it will be possible to identify which tests are the most sensitive and consequently the most useful in terms of screening children with optic pathway gliomas.

Aims
The aim of this part of the low grade glioma Study is to introduce a standardised methodology of visual assessment in children of all ages with optic pathway glioma. The data will be collected in order to assess the feasibility of the tests of visual function in an international setting.

It is not possible at this stage to validate these tests as there is no known gold standard with which to compare. Therefore patients can also be entered into a pilot study of visual function testing including the use of visual evoked potential, and comparing formal tests with subjective assessment of visual function by the parent and patient, a vision behaviour check list and with radiology (See section 19).

Tests of visual function
Children should be assessed through a combination of direct and indirect testing pertinent to their ages. The modalities for testing come under the following headings:

1. Visual acuity
2. Visual fields
3. Colour vision
4. Contrast sensitivity
5. Ocular motility assessment
6. Pupil responses
7. Fundoscopy

1) Visual acuity
Visual acuity testing should be recorded using a Logmar chart which, with matching cards, can be used in children as young as 2 ½. The Logmar chart can be used either as a letter format or as LEA symbol format (LEA test done at roomlight). For children under 2 ½ or in those where there are communication problems or other difficulties, acuity card preferential looking should be used. Visual acuity is graded from 8 (best) to 1 (worst):

Grade	LOGMAR/LEA	PL (c/d)
8	0-0.2	≥19.5
7	0.3-0.4	14.2-9.8
6	0.5-0.7	7.5-4.8
5	0.8-1.0	3.6-2.4
4	1.1-1.3	1.8-1.2
3	Hand/Toy movement	
2	Perception of light	
1	No perception of light	

2) Visual Fields

Formal perimetry should be carried out in children who are old enough to co-operate with the test. Certainly children over the age of 6 or 7 should be able and sometimes younger children can also comply. Goldmann visual fields using an experienced examiner are often both more accurate and more possible than static perimetry using an automated system. In young children confrontation testing using a toy or bright object and two examiners is a better technique. Visual field assessment is also graded on an 8-part scale:

Grade	Achievement
8	Monocular Full
7	Monocular Quadrantic
6	Binocular Quadrantic
5	Monocular Hemionopic
4	Binocular Hemionopic
3	Monocular Hemi and Quadrantic
2	Binocular Hemi and Quadrantic
1	Total Loss

3) Colour Vision

The PVC 16 colour vision testing system is likely to be the best option for testing children in this patient group. There is a reduced version of the Farnsworth 100 hue test, which involves a child matching colours. Depending how accurately these colours are matched colour vision can then be assessed and consequently graded. The Isschihara plate system is a historical test, which was devised primarily to identify patients with red/green colour-blindness. The axis of colour loss in children with optic nerve pathology is more likely to be in the blue-yellow spectrum and as a result the Ishihara test is not particularly useful. The grading system for colour vision using the PVC 16 test can be used:

Grade	Chart Results
8	Colour Circle complete
7	Close caps confused
6	One crossing of circle 7 <--> 15
5	Up to 2 crossings (other than 7 <-> 15)
4	Up to 4 crossings (other than 7 <-> 15)
3	5 crossings (other than 7 <-> 15)
2	6 crossings (other than 7 <-> 15)
1	7 crossings (other than 7 <-> 15)

Defect Axis	Protan / Deutan / Tritan / Mixed (please indicate)

The examiner should also document the principal colours that are predominately missed (blue, red, green etc.).

4) Contrast Sensitivity

There is evidence that contrast sensitivity testing can be used to pick up subtle changes in optic pathway function and as such should be incorporated into a standard screening protocol for these children. Contrast sensitivity develops at a faster rate than visual acuity during the first 30 weeks of life. Contrast sensitivity testing has been shown to be more sensitive than acuity, field and colour vision testing in optic neuropathy. It has also proved helpful in-patients with visual pathway glioma. (Day 1997)

The VISTECH vision contrast system or the LEA contrast sensitivity test should be used on all children. With the LEA matching cards it should be possible to test children down to 2 ½ to 3 but the VISTECH system may be difficult in children under the age of 4.

Using the VISTECH colour vision contrast test there is again a grading system on an 8-part scale:

Grade	Column "A" (1.5 c/d)
8	Grating 8
7	Grating 7
6	Grating 6
5	Grating 5
4	Grating 4
3	Grating 3
2	Grating 2
1	Grating 1

Gradings for Lea Contrast Sensitivity:

	VA -0.2 to 0.175 Log MAR test at 3M	VA 0.2 to 0.475 Log MAR test at 3M	VA 0.5 - 0.775 Log MAR test at 1M	VA 0.8 to 1.0 Log MAR test at 1M
**Grade**	No of Symbols seen	No of Symbols seen	No of Symbols seen	No of Symbols seen
**8**	25 - 22	25 - 18	25 -22	25 - 16
**7**	21 - 18	17 - 16	21 - 18	15 - 12
**6**	17 - 14	15 - 13	17 - 14	11 - 10
**5**	13 - 10	12 - 9	13 - 10	9 - 7
**4**	9 - 7	8 - 6	9 - 7	6 - 5
**3**	6 - 4	5 - 3	6 - 4	4 - 3
**2**	3 - 2	2	3 - 2	2
**1**	1	1	1	1

5) Ocular motility assessment

Children with poor vision can develop strabismus and consequently recording of the presence or absence of a squint is important. There is no grading system as such for this although it should be documented whether a squint is convergent or divergent or vertical and a measurement of either the prism cover test or the prism reflection test (this would be in degrees or prism diopters). The presence of nystagmus should be noted which will also include its orientation (horizontal, vertical or rotary) and nature (jerk, pendular etc).

6) Pupil responses
All children should be assessed for a relative afferent pupillary defect. This is achieved using the swinging flash light test. There is no grading for this but it should be noted if a relative afferent pupillary defect is present and if so can this defect be neutralised with neutral density filters. These filters come in an increasing density and consequently mimic a loss of luminance in the eye that is being tested. By putting these filters in front of the good eye an attempt can be made to classify the relative afferent defect in the bad one.

7) Fundoscopy
Whilst not assessing optic pathway function the appearance of the optic nerves is important to document. The appearance of optic atrophy should be noted.

Frequency of examinations
The consensus statement of the NF I optic pathway glioma task force (Listernick 1997) suggests ophthalmological examinations for children with optic pathway gliomas every 3 months during the first year following diagnosis and six-monthly until 36 months and yearly thereafter. But this however relates to surveillance and the frequency will need to be increased for children experiencing visual deterioration or for children under treatment, who will need closer follow-up. During chemotherapy it has been suggested that 3-monthly investigations should take place (Lorenz 2002). Table 27 shows the recommended frequency of ophthalmological examination for children participating in this study.
If there is a change in a child's condition they should be returned to 3 monthly assessment for 12 months and then to 3-6 monthly and then to 6 monthly.

Table 28: Recommended frequency of ophthalmological examination during treatment and follow-up (Lorenz 2002):

At diagnosis				
Surgery	before	after	2 weeks after	each surgical intervention
Chemotherapy	before	3 monthly		during chemotherapy
Radiotherapy	before	3 monthly		after end of radiotherapy
Follow-up	1st year	3 monthly		
	2nd year:	3-6 monthly		More frequently, if indicated
	3rd year	6 monthly		More frequently, if indicated
	4th year and later:	6-12 monthly		More frequently, if indicated

Documentation
For documentation of all findings the Ophthalmology data form in Addendum 21.13.6. should be used and completed forms be sent to the national data collecting center.

8.7. Health status and quality of life assessment

Aim
To determine the quality of survival of children treated for low grade gliomata, and compare this between different trial arms.

The secondary aim is standardisation of morbidity assessments across European pediatric brain tumor clinical trials, in order to enhance compliance and completion of data sets consequent upon familiarity of clinical teams with the system. Comparison of morbidity data between tumor groups will be possible.

Methodology
The UKCCSG and SIOP Brain Tumor Group have agreed upon a standardised framework for monitoring of morbidity burden consequent upon the diagnosis and treatment of brain tumors (Glaser et al, 1999). This will be adopted to national structures with appropriate modifications due to developments in methodologies since its publication. Four of the original questionnaires (Strengths and Difficulties Questionnaire[SDQ], Health Utilities Index[HUI], Medical Examination Form, Medical/Educational/Employment/Social Form) will be used. Additional health-related quality of life measures will be used.

The HUI and SDQ have been widely used and are available in 7 European languages (Goodman 1994, Feeny et al 1995). Their use is supported by the SIOP Brain Tumor Group. The medical examination form and medical/employment/education/social form for patients and parents need to be adapted for individual countries as educational qualifications and support will vary. This system is being adopted in SIOP PNET 4. The same forms will be used in this study as for PNET 4.

Health-related quality of life measures are important in providing information about patients, and their parents, perception of their health and well-being. Few measures are suitably translated, and validated, for inclusion in an international study across Europe. In keeping with SIOP PNET 4, three measures will be available for this study; the PedsQL (Varni et al,1999), PEDQOL (Ravens-Sieberer and Calaminus, 1998) and the Child Health Questionnaire[CHQ] (Landgraf et al, 2000). In the United Kingdom the PedsQL will be used, whilst in Germany the PEDQOL will be the measure of choice. The CHQ is available in multiple European languages and should be adopted by other participating countries (data is only by parental proxy response). Aged 18 + years, the EORTC QLQ-C30 with brain tumor specific add-on module (Aaronson et al, 1993) is recommended for use in all countries.

Schedule of assessments
Both, medical/education/employment/social assessment and Quality of Life, should be assessed at diagnosis, 1 year, 3 years, 5 years, 10 years from diagnosis and at age 20 years.

9. Patient Eligibility SIOP LGG 2004

9.1. Inclusion Criteria

1.1 **Age**: children and adolescents up to the completion of the 18th year of life.

1.2 **Histology**: low grade glioma according to ICD O Code[2]

Pilocytic Astrocytoma I°	9421/1
Subependymal Giant Cell Astrocytoma I°	9384/1
Dysembryoplastic Neuroepithelial Tumor I°	9413/0
Desmoplastic Infantile Ganglioglioma I°	9412/1
Ganglioglioma I° and II°	9505/1
Pleomorphic Xanthoastrocytoma II°	9424/3
Oligodendroglioma II°	9450/3
Oligoastrocytoma II°	9382/3
Astrocytoma II°	9400/3
Fibrillary Astrocytoma II°	9420/3
Protoplasmatic Astrocytoma II°	9410/3
[illegible]	[illegible]

Children with chiasmatic-hypothalamic tumors may be eligible without histological diagnosis, if neuroradiologic findings meet unequivocal criteria for the presence of a low grade glioma.

1.3 **Primary tumor localization**: intracranial and/or spinal cord.

1.4 **Dissemination**: Children presenting with disseminated low grade glioma will be eligible for the study.

1.5 **Associated conditions**: Children are eligible for the trial regardless of the presence of associated genetic disease: Neurofibromatosis NF I will be the prominent one, all children with NF I are entered into the study arm III in case of an indication for non-surgical therapy. Other conditions like Tuberous Sclerosis etc. should be registered and their impact on the course of disease and/or therapy be followed.

1.6 **Primary tumor diagnosis**: The tumor should not be pretreated with chemotherapy or radiotherapy.

1.7 **Informed consent**: The patient and/or his legal guardian (parents) have to have declared their written informed consent to the study.

Randomization: All eligible patients without Neurofibromatosis NF I receiving chemotherapy as their fist non-surgical therapy are eligible for randomization.

[2] ICD-Codes korrigiert (Juli 2004)

9.2. Exclusion Criteria

2.1. **Primary tumor localization**: diffuse intrinsic tumors of the pons, even if histologically an Astrocytoma I° or II° is diagnosed.
Exception: pontine glioma II° in NF I patients may be entered into the study.

2.2. **Special diagnosis**: Patients presenting with rare intracranial neoplasms of low grade malignancy, but non-glial origin may be followed according to the low grade glioma strategy but they are not subject of this therapy trial. Their data may be registered however, to learn about those therapeutic interventions which may prove useful to these patients and to develop separate strategies in the future. Choroid plexus papilloma should be entered into the SIOP-CPT study (PD. Dr. J. Wolff, Children's Hospital, Regensburg, Germany).

2.3. **Pretreatment**: Children treated with chemo- or radiotherapy prior to entering the study will be evaluated separately. (Previous treatment with steroids is not considered a chemotherapeutic treatment).

2.4. **Preexisting impairments** of health status, making the conduct of the study impossible or ethically unwise.

2.5. **Evidence of pregnancy or lactation period**.

Participation in another clinical study.
In case the patient participates in another clinical study simultaneously to being enrolled in the study SIOP-LGG 2004, which is not interfering with the present treatment strategy (e.g. endocrinologic study), this should be known to the national study chairmen.

Medication.
Concommittant medication for associated or other conditions (e.g. hormone replacement, anticonvulsants), not containing cytostatic drugs, should be recorded, but is no exclusion criteria.

10. Indications to start non-surgical therapy SIOP LGG 2004

The indications to start non-surgical therapy are identical for all low grade glioma, with non-surgical therapy being either chemotherapy or radiotherapy. Since a first attempt of resection should be performed, if feasible, while some children will be diagnosed on neuroradiological grounds only, there are three major settings, where the decision to start non-surgical therapy has to be made.

The decision to start non-surgical therapy – differently to tumors of high malignancy – is a critical one. It is difficult to elaborate objective and reproducible criteria. Acknowledging this fact, all physicians entering patients into the trial are requested to verify carefully, if the criteria to start therapy are met, and to specify very clearly the possible reasons in case these criteria are not respected.

I. Indication to start non-surgical therapy at diagnosis following subtotal or partial resection (S2 – S3) (see section 16.2. for definition of extent of resection)

Severe preexisting visual disturbance (see section 8.6.)
- Borderline vision in both eyes (“threat to vision“)
- Definite history of visual deterioration
- Nystagmus due to impaired vision (especially in infants up to two years indicative of visual disturbance)

Clinical indication
- Diencephalic Syndrome
- Symptomatic metastases

Note: Neuroradiological indication
The presence of a postoperative residual tumor is not an indication to therapy on its own.

II. Indication to start non-surgical therapy at diagnosis without prior tumor resection (following biopsy or radiological diagnosis)

Severe visual symptoms
- Borderline vision in both eyes (“threat to vision”)
- Definite history of visual deterioration
- Nystagmus due to impaired vision (especially in infants up to two years indicative of visual disturbance)

Severe neurologic symptoms
- Diencephalic syndrome
- Focal neurologic deficits secondary to tumor growth
- Symptoms of increased intracranial pressure secondary to tumor growth (decompensated hydrocephalus occlusus should be treated by a shunting procedure)
- (Focal) Seizures secondary to tumor growth
- Symptomatic metastases

Note: Neuroradiological indication
The presence of a postoperative residual tumor is no indication to therapy on its own.

III. Indication to start non-surgical therapy following observation, if surgery is not feasible

Progressive neurologic symptoms
- Manifestation of new neurologic symptoms
- Increase of severety of existing neurologic symptoms
- Manifestation of Diencephalic Syndrome

Progressive visual disturbances
- Reduction / loss of vision or of visual fields
- Any reduction / loss of vision in the second eye, if the other eye is blind

Neuroradiologic progression
- Definite increase of tumor size * (Increase of the diameter of the optic nerve)
- Involvement of previously uninvolved areas of the brain
- Manifestation of disseminated disease (including symptomatic or progressive metastases)

* Assessment of tumor size (two- or three-dimensional) should always be performed in the same way in the same patient (see section 8.5.).

Tumor size (volume) progression – Unequivocal increase of tumor size (volume) is a criteria to start therapy. However, pilocytic astrocytoma may have solid and cystic components. If only the cystic component(s) enlarge, while the solid ones remain unchanged, this is no sufficient evidence of tumor progression, although neurosurgical intervention may be necessary to relieve symptoms of local or generalized pressure.

Decrease of the visual function - The evidence of an increasingly compromised visual function (marked decrease of the visual acuity and/ or the visual field) regardless of tumor volume changes, and in the absence of any other overt cause, should be considered a criteria for starting therapy. Clinicians have to be aware, that quite often in young children the results of the ophthalmological examinations may vary according to the child’s compliance to the procedure and the tests. Thus, particularly in face of a radiologically stable disease, any visual function changes should be confirmed by two consecutive ophthalmological tests. This is especially important for children with NF1. Visual evoked potentials may help to confirm clinical findings, but by themselves are not considered a sufficient criteria to evaluate tumor progression.

Diencephalic syndrome –DS in itself is a clinical condition for starting therapy.
Main characteristics are a progressive emaciation and failure to thrive (regarding body weight and less growth !) in an apparently alert, cheerful infant. DS is usually due to a low grade glioma involving the hypothalamus. Treatment with aggressive surgery and / or radiotherapy is variably successful in controlling the disease, but may result in severe neurologic sequelae. Chemotherapy seems effective in controlling the clinical symptoms despite a rather long time period until changes are seen.

Disseminated low grade glioma at diagnosis – the presence of multicentric, disseminated disease by itself is not necessarily an indication to start therapy, if no other criteria to initiate non-surgical therapy are met. A very careful and accurate period of clinical observation may be appropriate.

NF I – Metachronous tumors – Patients with NF I are at risk to develop multiple (brain) tumors, especially if they presented with optic pathway glioma (Friedman 1997). Such metachronous tumors have to be distinguished from secondary dissemination of a LGG. Thus, these tumors have an indication to therapy on their own.

Please contact the study chairmen for any unconventional situation before the start of non-surgical therapy and / or randomisation.

11. Patient Registration and Randomisation SIOP LGG 2004

11.1. Patient registration

All patients diagnosed to have a low grade central nervous system glioma should be registered according to national policies at the national study office and the national children‘s cancer registry.
Forms for registration at the national children’s cancer registry are provided nationally. Where there is no pre-organized national information transfer, registration to the national study office can be done by the form provided in Addendum 21.6.1.

Patients receiving either chemo- or radiotherapy will be centrally registered at the international study office. Data transfer between the national study offices and the international trial office confers to regulations of data security (see section 18.)

The trial coordinating center (international trial office) is located at the:

SIOP-LGG 2004 International Data Centre
Clinical Trials & Biostatistic Unit
Istituto Oncologico Veneto
Busonera Hospital
Via Gattemelata 64
I-35128 Padova, Italy

Telefone: 0039-049-8215704
Fax: 0039-049-8215706
email: siop-lgg2004@istitutoncologicoveneto.it

11.2 Patient randomisation

Randomization is provided centrally, by a computer-based service (supplied by CINECA, Casalecchio ITALY) that is accessible via Internet, for all patients without NF I, for whom it is applicable. Access to the randomisation system is managed according to specific policies adopted by each country (both direct local site and mediated by national data centre access are possible).All eligibility criteria (section 9.) and requirements for randomization have to be fulfilled prior to the randomization process:
The presence of a low grade glioma should be confirmed either by central neuro-pathologic review, if a biopsy has been obtained, or by central neuro-radiologic review, if the diagnosis is made on the basis of MRI / CT investigation only.

Randomization will be stratified according to age(< 1 year, 1-8 years, ≥ 8 years) and primary tumor site (pure chiasmatic tumors (Dodge II, Dodge 1958), all other supratentorial midline tumors, tumors of all other sites outside the supratentorial midline). To reduce possible imbalances in the number of treatment assignments, a randomised blocked design will be used.

Patients for whom randomization is requested have to be registered at their national trial office. The national center will check the eligibility of the patient and then obtain central randomization. The result will be reported back to the patient’s treatment center. This

procedure will require two working days. This should be kept in mind when planning treatment.

Before randomization the patient and/or his/her legal guardian/parents have to be adequately informed about the study, the background of this strategy and the possible therapeutic alternatives. Their written informed consent has to be obtained prior to randomization.

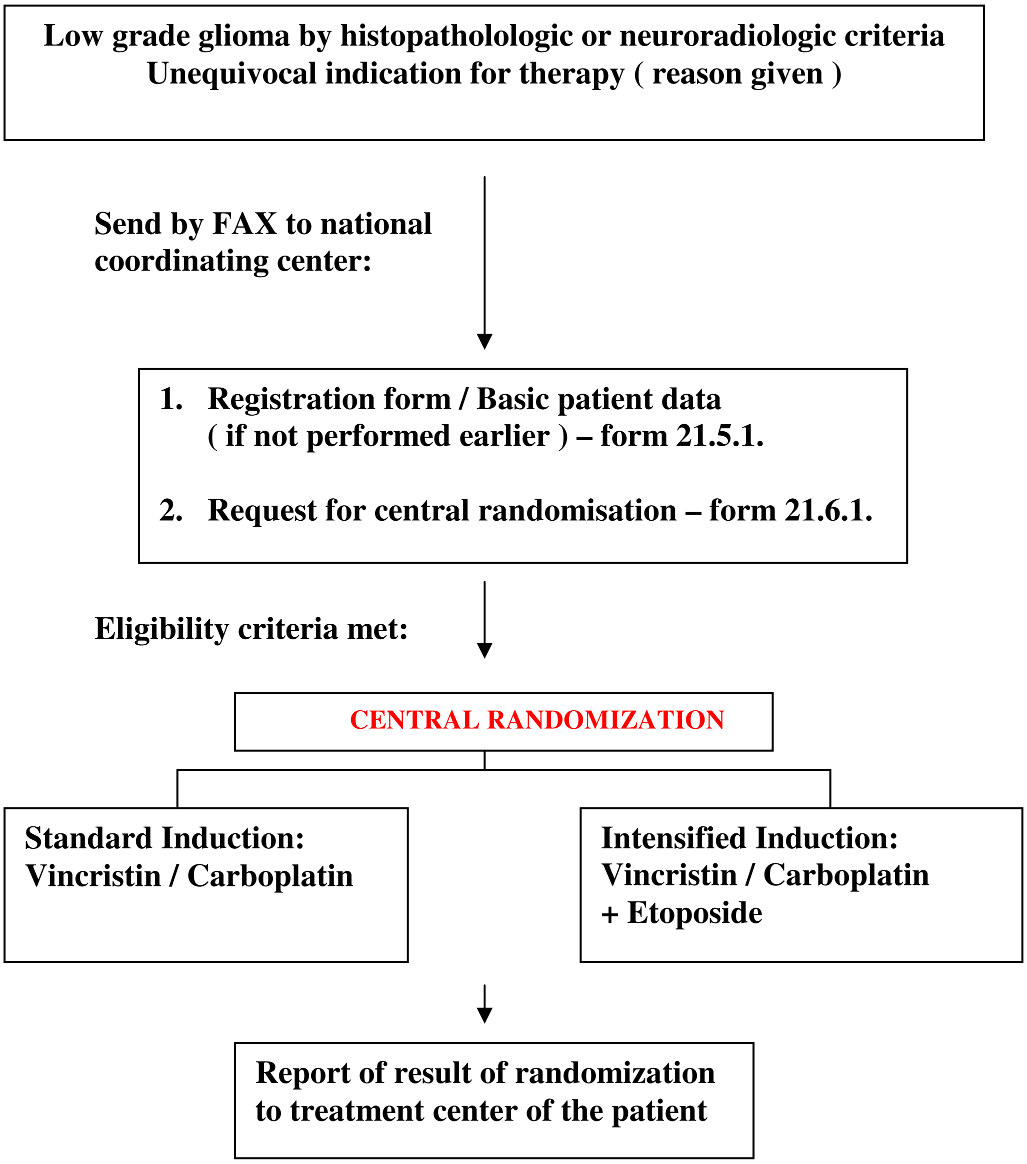

12. Study Overview SIOP LGG 2004

Treatment Scheme:

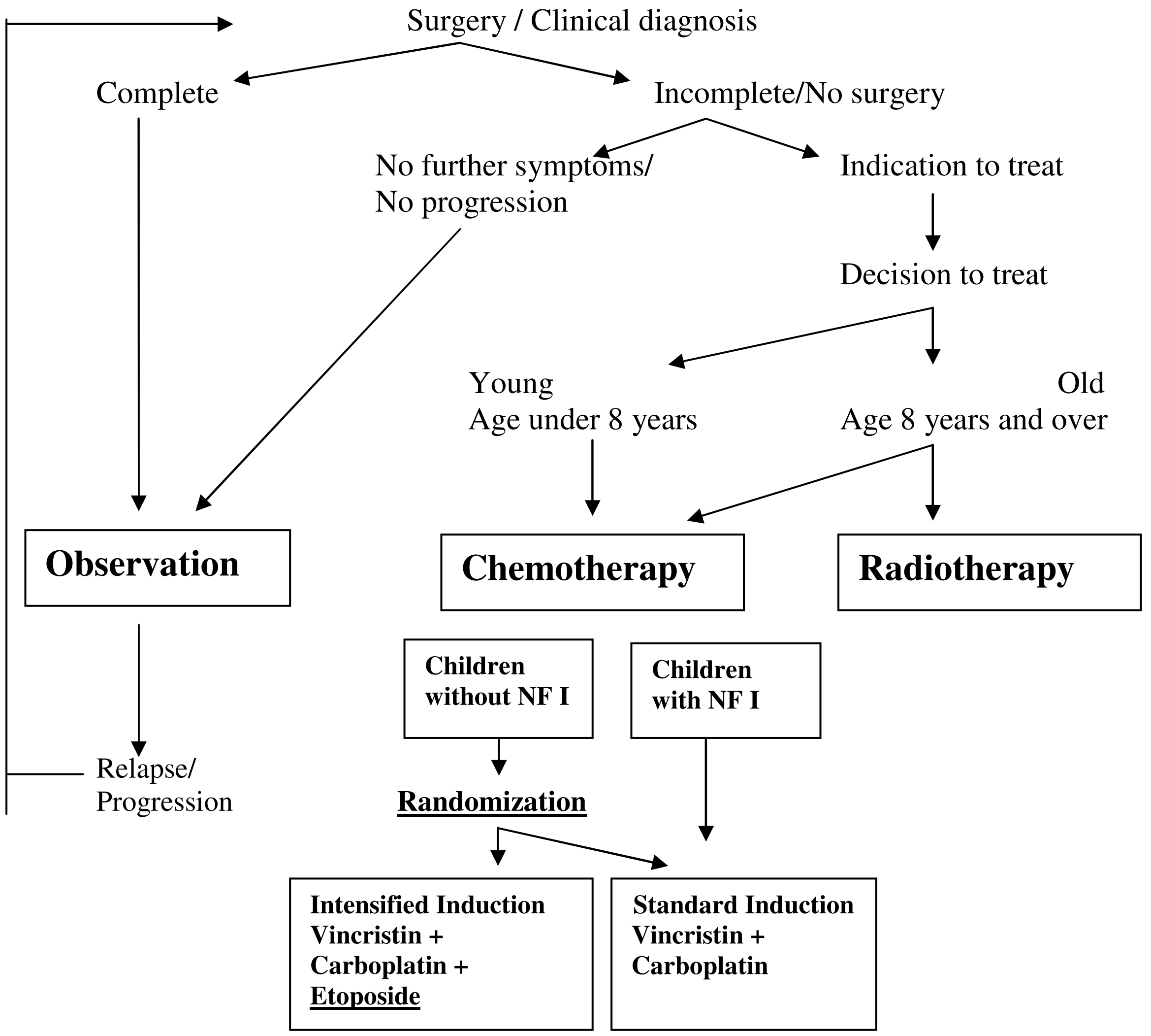

All patients with low grade glioma, eligible according to the criteria from section 9., should be entered into the current study and follow the same general strategy concerning the non-surgical therapy. Dependent upon primary tumor localization and the presence or absence of Neurofibromatosis NF I patients are devided into three therapeutic groups:

12.1.
Group 1: Children not affected by NF I with low grade glioma of the supratentorial midline.
12.2.
Group 2: Children not affected by NF I with low grade gliomas of all other sites.
12.3.
Group 3: Children affected by NF I with low grade glioma of all sites.

Rationale to separate the treatment groups

Considering that childhood low grade glioma are a very heterogeneous group of neoplasms, it is difficult to elaborate detailed common therapeutic guidelines applicable to all children with tumors of all sites. On the other hand the basic strategy of low grade glioma treatment can be applied to all children, if the specific conditions of separate tumor locations, tumor size and age of the child are considered. For example, the present refinements in the radiotherapy techniques (conformal or stereotactic fractionated radiotherapy) allow to conceive that radiotherapy may be delivered safely in selected primary sites and for selected targets even in young children (e.g. small residual of cerebellar astrocytomas). In the previous study the age of 5 was empirically chosen as the cut-off age for recommending chemotherapy or radiotherapy. In the light of more data, which have been accumulated on the effect of chemotherapy on low grade glioma, it is possible to extend this cut-off to the age of 8.

1. Supratentorial midline tumors in children not affected by NF I.

HCG and OPG represent a relatively homogenous group of LGG. Additionally the small number of tumors of the basal ganglia, the thalamus and the upper midbrain pose the identical clinical dilemma of mostly unresectable tumors. Thus, it is conceivable to elaborate detailed common therapeutic guidelines for them. In particular, due to the obvious limitations of any potential surgical acts aiming to remove completely the tumor, the role of CT, as outlined above, is much less controversial and their long term outcome needs to be improved with high priority.
For this subgroup of children the impact of intensifying the induction period will be investigated in a randomized study.

2. Low grade glioma arising at all other sites in children not affected by NF I.

Separate therapeutic guidelines will be elaborated for children with LGG arising from other sites of the CNS. This is true also for children with pure optic nerve glioma.
For this group of patients surgery plays a major prognostic role. After incomplete surgery the progression rate is between 40 and 50 % without adjuvant treatment (Fisher 2001), but for the treatment of relapse, surgery alone can result in long-term progression free survival especially in hemispheric and cerebellar tumors (Bowers 2001). Consequently, adjuvant treatment should be avoided, if second surgery is a complete resection. Even in case of late progression, several years after a first partial resection, a second partial resection can be considered.
Residual pilocytic astrocytoma may regress spontaneously, especially when the residual is small.
Additional prognostic factors may depend upon tumor location:
The interval between first symptoms and diagnosis is inversely correlated with the outcome in children with spinal tumors (Bouffet 1998).
Brainstem involvement is a significant risk factor for incomplete surgery and bad outcome in children with benign cerebellar astrocytoma (Pencalet 1999); these tumors probably need a different treatment strategy than classical cerebellar astrocytoma.

In these locations many other histologic types of low grade glioma are encountered as well, whose natural history is hardly predictable, but may be less favourable. In some locations, e.g. brainstem, focal lesions with a histology of pilocytic astrocytoma can be clearly distinguished from more diffuse tumors of either pilocytic or fibrillary types in terms of biological behaviour and prognosis (Fisher 2001). Therefore diffuse intrinsic pontine glioma, even if astrocytoma WHO I° or II°, has been excluded from the study and should be entered into trials for high grade glioma.
For the small subgroup of children needing chemotherapy the impact of intensifying the induction period upon primary response shall be investigated in a randomized fashion.

3. Low grade glioma of all sites in children affected by NF I.

Diagnosis of NF I should use the criteria published from the consensus conference on glioma in NF1 patients (Listernick 1997). Minor criterias for NF1 can be listed as well according to Cnossen (1998). A special case should be made for UBOs (unidentified bright objects) that are both a new diagnostic criteria and a diagnostic dilemma in some cases.

Almost quite uniformly all the studies run on childhood LGG have documented that the NF1 status is a favorable prognostic factor (see section 3.2.). But children with NF1 have specific problems. They are affected by a cancer-predisposing syndrome and concern exists on treating those children with potentially oncogenic agents (e.g Etoposide, RT…). Radiotherapy can be particularly deleterious for these patients in face of the pre-existing brain dysfunction, in that these children may suffer more sequellae, because of the NF1 status. Furthermore, NF1 children treated with cerebral irradiation may be at a higher risk than the normal population of developing severe and potentially fatal vascular complications (Capelli 1998, Grill 1999).

Within this trial children affected by NF I and necessitating non-surgical therapy will be treated separately according to the historical, but extended regimen with Vincristin / Carboplatin, regardless of their age at presentation. They should not be irradiated unless the chemotherapy and surgery options have failed.

Endpoints for treatment outcome evaluation

As previously stated due to the very long life expectancy of children affected by a LGG it is clear that the health status (HS) and the quality of life (QoL) in general and at least the neurological, visual and endocrinological function must be among the primary end-point of any treatment strategy directed to childhood LGG. The fact that reliable tools for measuring HS and QoL in young children are not available, make it impossible to test those two criteria for therapy effect; however the assessment of the visual, endocrinological and neurological function will be included in the outcome measurement.

12.1. Study overview: Children not affected by NF I (NF I-ve) with low grade glioma of the supratentorial midline. SIOP LGG 2004

This subgroup comprises a relatively homogenous group of low grade glioma. Hypothalamic-chiasmatic glioma and optic pathway and the small number of tumors of the basal ganglia, the thalamus and the upper midbrain pose the identical clinical dilemma of mostly unresectable tumors. Thus, the role of non-surgical therapy, and in particular chemotherapy for the young, is much less controversial and the long term outcome for these children needs to be improved with high priority.
For this subgroup of children the impact of intensifying the induction period of chemotherapy shall be investigated.

Eligibility criteria to this treatment group:

Tumor location: optic pathways/chiasmatic-hypothalamic region, basal ganglia, thalamus, mesencephalon (lamina quadrigemina, tectum mesencephali)

Staging Chiasmatic-hypothalamic and optic pathways gliomas should be classified additionally according to the Dodge classification (Dodge 1958):
Dodge II: tumors of the optic chiasm with or without optic nerve involvement.
Dodge III: tumors of the optic chiasm with extension into the hypothalamus and other diencephalic structures.

Histology: Low grade glioma according to section 9.1.
Histologic diagnosis is primarily made by the local pathologist, yet for all children randomized central pathologic review has to be obtained prior to randomization.

Alternatively:
Clinical diagnosis: Neuroradiologic criteria fulfilled according to section 8.5.
Neuroradiologic criteria have to be fulfilled for all children not biopsied and central neuroradiologic review has to be obtained prior to randomization.

Surgery: Any extent of primary surgery

Neurofibromatosis I: absent.
It should be noted that in very young children the signs of NF I may not be apparent and it is necessary in patients with tumors compatible with Neurofibromatosis that the patient is repeatedly re-evaluated in the first five to seven years of life for signs of emerging criteria (careful examination of skin is recommended).
Standard reassessment will be requested during follow-up at the age of six years.

Age eligibility: If there is an indication for non-surgical treatment and parents and physicians have made the decision to treat, the choice of either radio- or

chemotherapy has to consider the age of the patient (and the size of the tumor):

- It is recommended that all children younger than 8 years will be entered into the chemotherapy study as „young age group".
- Those of eight years and older as the „old age group" could be entered into the chemotherapy study and randomized - or could be entered into the radiotherapy study at the patient / parent / physicians's preference.

Treatment strategy for children unaffected of NF I with low grade glioma of the supratentorial midline :

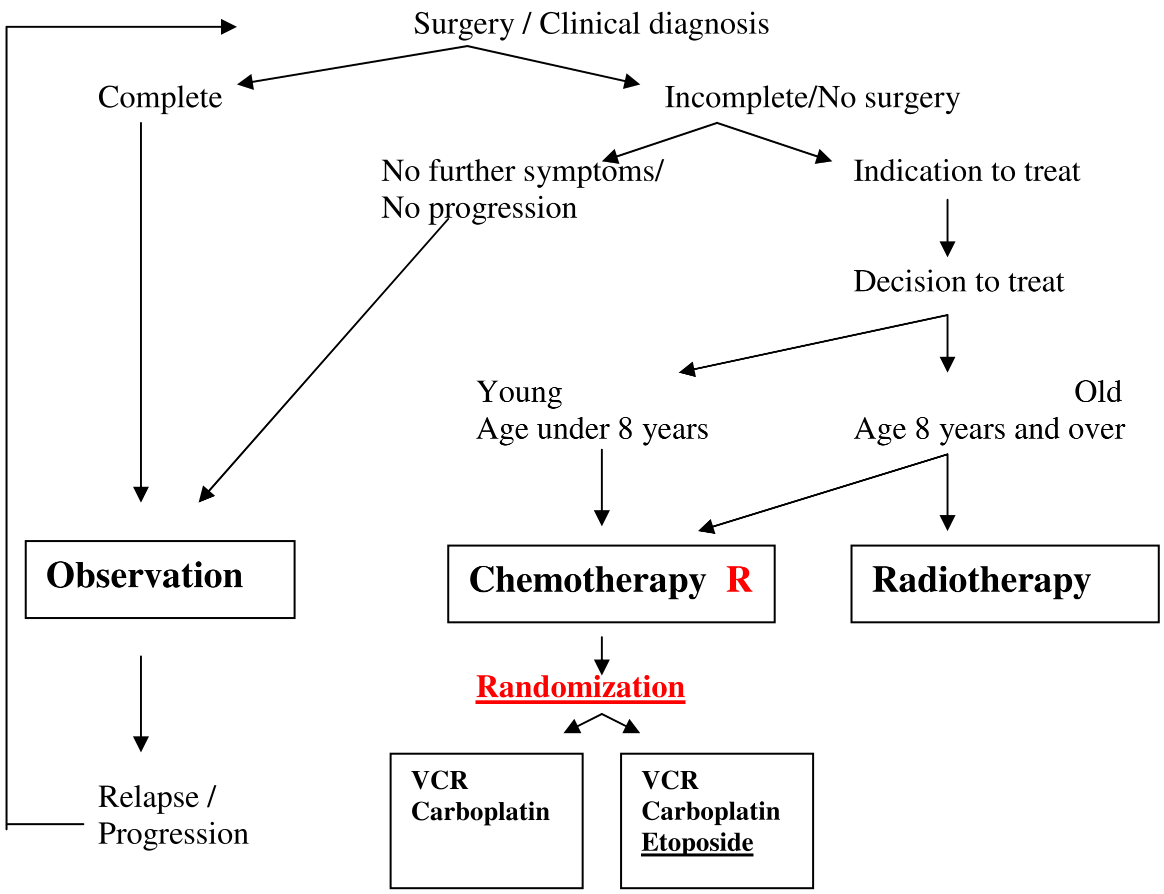

Registration:

Patients shall be registered nationally at diagnosis irrespective of the indication for or type of non-surgical therapy. National data centers will forward information of all treated and non-treated patients into the common international data-bank.
The national data center is responsible for quality assurance of data handling and management.

Randomization:

Request for randomization is forwarded to the national data center, where patient eligibility is checked. Central randomization will be performed only, if the preconditions are fulfilled completely, and the result of central randomization will be communicated to the treatment center as well as to the national study office.

Treatment modalities:

A. Surgery

I. Surgery at diagnosis/biopsy

The extent of surgical removal has to be discussed in the light of tumor location and local or distant tumor extension. In the case of hypothalamic chiasmatic tumors with either severe or progressive reduction of vision and loss of visual fields the aim of surgical debulking has to be carefully considered.

Irrespective of the extent of tumor removal relief of increased intracranial pressure by shunting procedures where indicated, should be performed.

II. Second surgery

If there is the chance for a more complete tumor resection following primary tumor resection, or during observation or during the course of non-surgical therapy, this possibility should be discussed within the local treatment team, with the national study chairman and/or with the reference surgeon. Although tumor resections are recommended, the child should never be endangered by surgical intervention or suffer from severe neurologic / visual impairment postoperatively.

B. Chemotherapy

Children, for whom there is an indication to be treated by chemotherapy and for whom the decision has been made to actually start treatment, shall be randomized and receive either standard induction treatment with Vincristin and Carboplatin or intensified induction with Vincristin and Carboplatin plus Etoposide (see section 14. for details).

> **Note:** Neuroimaging should be obtained prior to start of therapy at an interval less than 4 weeks !

I. Standard Induction:

Vincristin is given once weekly as an iv-bolus at a dose of 1,5 mg/m²/day on day 1 of week 1, 2, 3, 4, 5, 6, 7, 8, 9 and 10 and then week 13, 17 and 21.
(maximum single dose: 2 mg; dose for children < 10 kg body weight: 0,05 mg/kg/day).
Carboplatin is given as an intravenous 1-hour-infusion at a dose of 550 mg/m²/day on day 1 of week 1, 4, 7 and 10, and then week 13, 17 and 21 (dose for children < 10 kg body weight: 18,3 mg/kg/day).

II. Intensified Induction:

Vincristin is given once weekly as an iv-bolus at a dose of 1,5 mg/m²/day on day 1 of week 1, 2, 3, 4, 5, 6, 7, 8, 9 and 10 and then week 13, 17 and 21.
(maximum single dose: 2 mg; dose for children < 10 kg body weight: 0,05 mg/kg/day).
Carboplatin is given as an intravenous 1-hour-infusion at a dose of 550 mg/m²/day on day 1 of week 1, 4, 7 and 10, and then week 13, 17 and 21 (dose for children < 10 kg body weight: 18,3 mg/kg/day).
Etoposide is given as an intravenous 1-hour infusion at a dose of 100 mg/m²/day on day 1 to 3 of week 1, 4, 7 and 10. (no dose adaptation for children < 10 kg body weight).

III. Consolidation

All children will receive consolidation therapy up to week 81 with ten 6-week cycles of Vincristin and Carboplatin. Cycles start in week 25, 31, 37, 43, 49, 55, 61, 67, 73 and 79. Vincristin is given once weekly as an iv-bolus at a dose of 1,5 mg/m²/day on day 1, 8 and 15 of each cycle (maximum single dose: 2 mg; dose for children < 10 kg body weight: 0,05 mg/kg/day).
Carboplatin is given as an intravenous 1-hour-infusion at a dose of 550 mg/m²/day on day 1 of each cycle (dose for children < 10 kg body weight: 18,3 mg/kg/day).

IV. Allergy
In case a patient develops allergy to Carboplatin during consolidation, therapy shall be continued with alternative drug combinations (Cisplatin/Vincristin and Cyclophosphamide/Vincristin) maintaining treatment intervals and total treatment time. A maximum of 5 cycles with both drugs should not be exceded to limit cumulative doses.
Allergy during induction treatment is a rare event, further treatment should be individually planned following discussion with the national study center.
Vincristin is given once weekly as an iv-bolus at a dose of 1,5 mg/m²/day on day 1, 8 and 15 of each cycle (maximum single dose: 2 mg; dose for children < 10 kg body weight: 0,05 mg/kg/day).
Cisplatin is administered at 30 mg/m² as a 3-h infusion on day 1 and 2 of each cycle (dose for children < 10 kg body weight: 1 mg/kg/day).
Cyclophosphamide is given at 1500 mg/m² as a 1-h infusion on day 1 of each cycle (dose for children < 10 kg body weight: 50 mg/kg/day).

V. Recommendation in case of early progression
In case progressive disease is diagnosed and the commencement of radiotherapy shall still be deferred, the recommended chemotherapy is the use of Cisplatin/Vincristin and Cyclophosphamide/Vincristin as in the case of allergy.

C. Radiotherapy

Older children, who upon indication for non-surgical therapy will receive external beam radiotherapy, will be irradiated with 54 Gy tumor dose conventionally fractionated at 1,8 Gy given on five days per week. The specific aims of the radiotherapy study are a maximal sparing of organs at risk by applying radiotherapy with modern planning and technical equipment (see section 15.).

In case, that it is necessary to give radiotherapy to younger children, it is recommended to contact the national study chairmen for radiotherapy (see section 15.).
Interstitial radiotherapy (brachytherapy) may be indicated in tumors amenable for this type of therapy.

D. Central neuroradiologic evaluation

Within the chemotherapy arm of the study central neuroradiologic assesment is mandatory and scans have to be sent in at definite time points (see section 8.5.):
- in order to validate radiological criteria for tumor progression and to confirm radiological or diagnostic imaging criteria (time point 1 and 2).
- to validate the response and assess the distribution of response at week 24 following induction treatment (time point 3),

- to define the point, when the “best response” throughout treatment is reached (time points 3 to 5)
- to compare subsequent scans (time point 6) where progression was deemed to have occurred in order to validate the time of progression.

Scans following radiotherapy will be assessed in a comparable pattern.

Qualitative changes of contrast enhancement will be described and correlated with response. See section 8.5. for radiodiagnostic guidelines and section 16. for tumor response and remission criteria.

E. Treatment recommendations following tumor progression

Despite all efforts to prevent tumor progression by primary therapy, a significant number of children will suffer from progression during or after first line therapy. Thus, the treatment strategy for low-grade gliomas has to incorporate recommendations for second (and third) line treatment approaches. In each case the possibilities for a meaningful surgical intervention should be checked, as well.

I. Progression during chemotherapy (early progression) in a young child (< 8 years)
Chemotherapy in these children is started to postpone radiotherapy. So, if PD occurs at the first evaluation at week 24 or later during consolidation and the child is still young, it is recommended that therapy be continued with the alternative chemotherapy regimen as in the case of Carboplatin allergy (section 14.1.3.). The two drug combinations of Cis-Platin/Vincristin and Cyclophosphmid/Vincristin are expected to offer an effective treatment.

II. Progression during chemotherapy (early progression) in an older child (≥ 8 years)
In case of progression at the first evaluation at week 24 or later during consolidation in a child older than 8 years, it should be assessed, if radiotherapy can be applied as second line therapy. If radiotherapy is no option, the alternative chemotherapy regimen (14.1.3.) should be used.

III. Progression following the end of chemotherapy
For children, who experience tumor progression following the end of therapy, several points have to be considered:
- age: Children still in the young age group, in whom radiotherapy should be further postponed, should receive second line chemotherapy.
  Children still in the young age group, in whom highly focussed radiotherapy appears possible, can go on to receive radiotherapy.
  Children in the older age group receive radiotherapy as second line therapy.
- time since the end of chemotherapy: If the first chemotherapy has been completed for more than a year, and the child has not had Carboplatin allergy, restart of standard Carboplatin/Vincristin chemotherapy may be taken into account. The alternative chemotherapy (14.1.3.) can be used as well.
  If the intervall between the end of first chemotherapy is shorter than a year and/or the child has had Carboplatin allergy, the use of the alternative regimen (14.1.3.) is recommended. The national chairman should be contacted to plan details for the "induction" phase.
- previous allergy: If first line chemotherapy has already been complicated by Carboplatin allergy and the alternative drug combinations thus have already been applied and second line chemotherapy is indicated, it is recommended to contact the national

chairman for the investigation of Phase II-treatment protocols. A trial with Vinblastin will be offered by the Phase II coordinators of the study.

IV. Progression following radiotherapy

For all children, who have received radiotherapy as their first treatment, chemotherapy is the first option in case of tumor progression. They will not be randomized and receive standard induction and consolidation with Vincristin and Carboplatin.

12.2. Study overview: Children not affected by NF I (NF I-ve) with low grade gliomas of all other sites. SIOP LGG 2004

This section will provide guidelines for the treatment of tumors, which have been incompletely resected (primary or following relapse) or which are disseminated at sites other than the supratentorial midline in patients, who have not got the clinical signs of Neurofibromatosis NF I.
For the small subgroup of children receiving chemotherapy the impact of intensifying the induction period of chemotherapy shall be investigated.

Eligibility criteria to this treatment group:

Tumor location: For the purposes of this section five main anatomical groupings are considered:

1. Cortical tumors
2. Cerebellar tumor
3. Brain stem tumors
4. Spinal tumors
5. Optic nerve tumors (intraorbital, anterior N II; Dodge I)

Staging: Staging investigations for supratentorial tumors need only include spinal imaging, if there is evidence of intracranial dissemination or symptomatic spinal disease.
Infratentorial / spinal tumors should have spinal imaging as a routine.

Histology: Low grade glioma according to section 9.1.
All tumors should at least be biopsied, neuroradiological criteria do not allow the differentiation of the various histological subtypes at these locations.
Histologic diagnosis is primarily made by the local pathologist, yet for all children central pathologic review is recommended. It is mandatory for children entering the randomized trial.
Diffuse intrinsic astrocytoma of the brainstem are not elegible (see section 9.2.)

Surgery: Any extent of primary surgery

Neurofibromatosis I: absent.
It should be noted that in very young children the signs of NF I may not be apparent and it is necessary in patients with tumors compatible with Neurofibromatosis that the patient is repeatedly re-evaluated in the first five to seven years of life for signs of emerging criteria (careful examination of skin is recommended).
Standard reassessment will be requested during follow-up at the age of six years.

Registration:
Patients shall be registered nationally at diagnosis irrespective of the indication for or type of non-surgical therapy. National data centers will forward information of all treated and non-

treated patients into the common international data-bank.
The national data center is responsible for quality assurance of data handling and management.

Randomization:
Request for randomization is forwarded to the national data center, where patient eligibility is checked. Central randomization will be performed only, if the preconditions are fulfilled completely, and the result of central randomization will be communicated to the treatment center as well as to the national study office.

Age eligibility: Guidelines for non-surgical therapy

If there is an indication for non-surgical treatment according to section 10. and parents and physicians have made the decision to treat, the choice of either radio- or chemotherapy has to consider the age of the patient (and the size and state of dissemination of the tumor):

Younger patients (under 8 years):
- For younger patients, regardless of their metastatic stage, chemotherapy can be considered as the first adjuvant treatment. Response assessment should be done with scans at six months following the start of treatment and six monthly thereafter until completion of therapy.
- In selected small tumors highly focussed radiotherapy may be considered, if the radiation dose to normal brain can be substantially reduced by this technique.
- Following tumor progression after primary response to Carboplatin/Vincristin chemotherapy in children that are still young, the recommended strategy is a second trial of chemotherapy with alternative drugs before radiotherapy is considered.
- If, during or after second line chemotherapy, the tumor progresses or if disseminated disease develops during or after chemotherapy and the patient is still in the young age category, consideration should be given to entering the patient into a phase II trial of novel agents or the use of alternative chemotherapy strategies from the published literature. Radiotherapy options may be discussed with the national radiotherapy reference center.
- Craniospinal irradiation may be considered for progression of disseminated disease during or following chemotherapy, if no further chemotherapy options are available.

Older patients (8 years and older):
- In older children without disseminated disease radiotherapy is the preferred first adjuvant therapy. Highly focussed fractional techniques should be employed to limit irradiation of uninvolved tissues, where possible. Radiotherapy options should be discussed with the national radiotherapy reference center.
- At the patient / parent / physician's preference entry to the chemotherapy study may be an option for this patient group.
- In older children with disseminated disease beyond conventional involved radiation field boundaries for the primary tumor, or in multifocal disease, chemotherapy should be tried and response assessment should be done with scans at six months and six monthly thereafter until completion of therapy.
- Craniospinal irradiation should be considered for progression of disseminated disease during or following chemotherapy.

I. Treatment strategy for children unaffected of NF I (NF I-ve) with low grade cortical, cerebellar and brain stem tumors

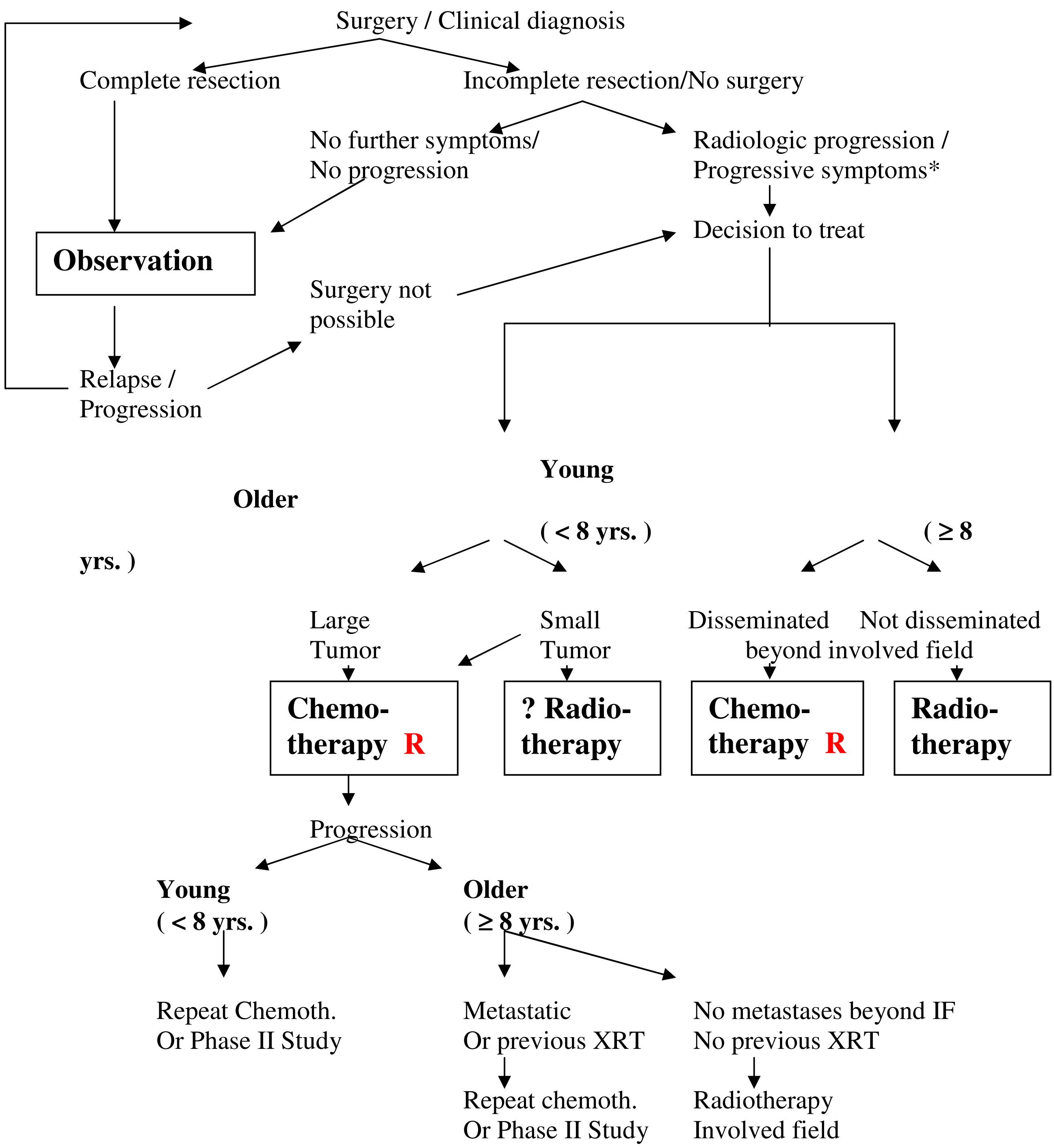

*: In cortical tumors epilepsy controlled by anti-epileptics is not initially considered "symptomatic disease".
IF: involved field.

R: Randomisation of Induction chemotherapy as in 12.1.:
Vincristin/Carboplatin vs. Vincristin/Carboplatin/Etoposide

II. Treatment strategy for children unaffected of NF I (NF I-ve) with low grade spinal tumors:

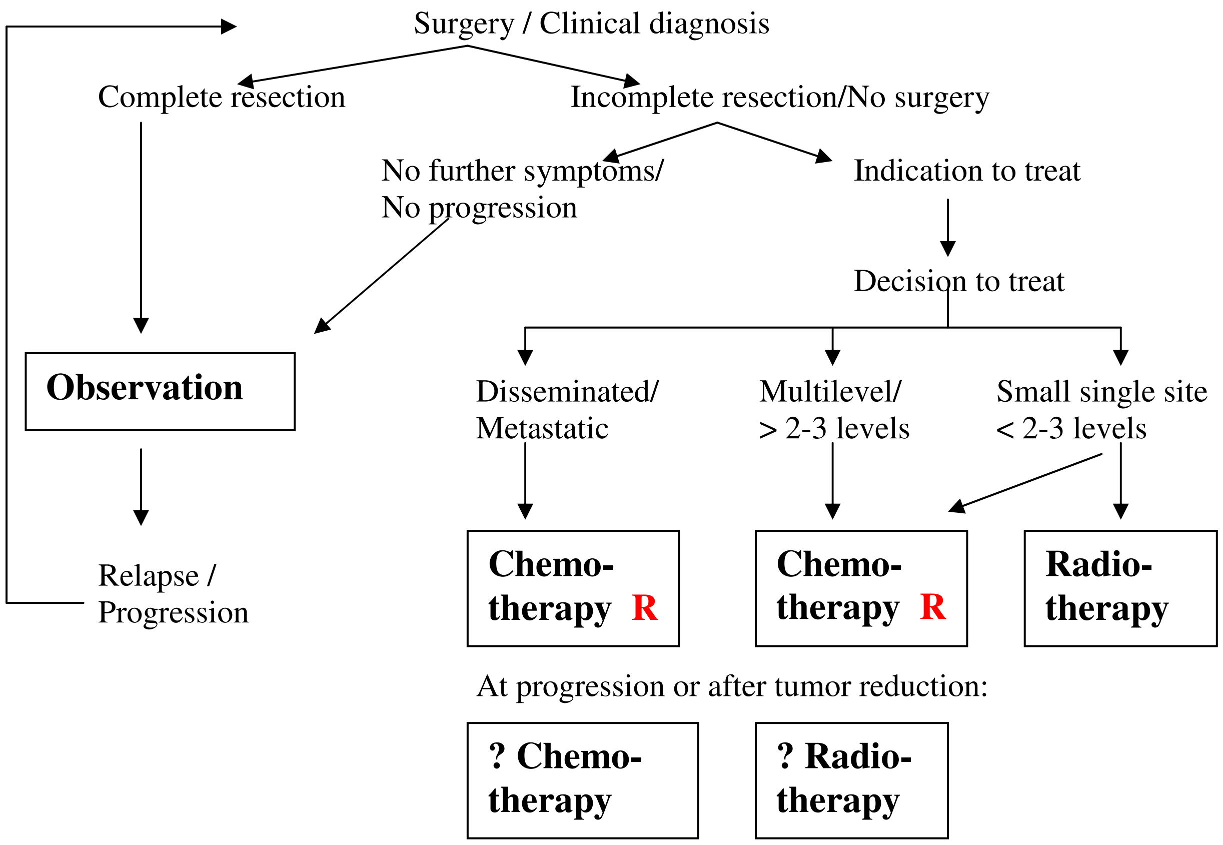

R: Randomisation of Induction chemotherapy as in 12.1.:
Vincristin/Carboplatin vs. Vincristin/Carboplatin/Etoposide

Treatment modalities:

A. Surgery

I. Surgery at diagnosis
The extent of surgical removal has to be discussed in view of tumor location and local or distant tumor extension.

II. Second surgery
If there is the chance for a more complete tumor resection following primary tumor resection, or during observation or during the course of non-surgical therapy, this possibility should be discussed within the local treatment team, with the national study chairman and/or with the reference surgeon. Although tumor resections are recommended, the child should never be endangered by surgical intervention or suffer from additional severe neurologic impairment postoperatively.

Adjuvant treatment is offered to patients with inoperable relapse after complete resection or progression of a residuum not amenable to complete resection.

B. Chemotherapy

In large tumors and/or in young children a trial of chemotherapy should be considered for low grade glioma necessitating non-surgical therapy. Chemotherapy may be given to replace irradiation, but also to reduce the volume of tumor to be irradiated or to make the residual tumor operable.
Children, for whom there is an indication to be treated by chemotherapy and for whom the decision has been made to actually start treatment, shall be randomized and receive either standard induction treatment with Vincristin and Carboplatin or intensified induction with Vincristin and Carboplatin plus Etoposide (see section 14. for details).

> **Note:** Neuroimaging should be obtained prior to start of therapy at an interval less than 4 weeks !

I. Standard Induction:
Vincristin is given once weekly as an iv-bolus at a dose of 1,5 mg/m²/day on day 1 of week 1, 2, 3, 4, 5, 6, 7, 8, 9 and 10 and then week 13, 17 and 21.
(maximum single dose: 2 mg; dose for children < 10 kg body weight: 0,05 mg/kg/day).
Carboplatin is given as an intravenous 1-hour-infusion at a dose of 550 mg/m²/day on day 1 of week 1, 4, 7 and 10, and then week 13, 17 and 21 (dose for children < 10 kg body weight: 18,3 mg/kg/day).

II. Intensified Induction:
Vincristin is given once weekly as an iv-bolus at a dose of 1,5 mg/m²/day on day 1 of week 1, 2, 3, 4, 5, 6, 7, 8, 9 and 10 and then week 13, 17 and 21.
(maximum single dose: 2 mg; dose for children < 10 kg body weight: 0,05 mg/kg/day).
Carboplatin is given as an intravenous 1-hour-infusion at a dose of 550 mg/m²/day on day 1 of week 1, 4, 7 and 10, and then week 13, 17 and 21 (dose for children < 10 kg body weight: 18,3 mg/kg/day).
Etoposide is given as an intravenous 1-hour infusion at a dose of 100 mg/m²/day on day 1 to 3 of week 1, 4, 7 and 10. (no dose adaptation for children < 10 kg body weight).

III. Consolidation
All children will receive consolidation therapy up to week 81 with ten 6-week cycles of Vincristin and Carboplatin. Cycles start in week 25, 31, 37, 43, 49, 55, 61, 67, 73 and 79.
Vincristin is given once weekly as an iv-bolus at a dose of 1,5 mg/m²/day on day 1, 8 and 15 of each cycle (maximum single dose: 2 mg; dose for children < 10 kg body weight: 0,05 mg/kg/day).
Carboplatin is given as an intravenous 1-hour-infusion at a dose of 550 mg/m²/day on day 1 of each cycle (dose for children < 10 kg body weight: 18,3 mg/kg/day).

IV. Allergy
In case a patient develops allergy to Carboplatin during consolidation, therapy shall be continued with alternative drug combinations (Cisplatin/Vincristin and Cyclophosphamide/Vincristin) maintaining treatment intervals and total treatment time. A maximum of 5 cycles with both drugs should not be exceded to limit cumulative doses.

Allergy during induction treatment is a rare event, further treatment should be individually planned following discussion with the national study center.
Vincristin is given once weekly as an iv-bolus at a dose of 1,5 mg/m²/day on day 1, 8 and 15 of each cycle (maximum single dose: 2 mg; dose for children < 10 kg body weight: 0,05 mg/kg/day).
Cisplatin is administered at 30 mg/m² as a 3-h infusion on day 1 and 2 of each cycle (dose for children < 10 kg body weight: 1 mg/kg/day).
Cyclophosphamide is given at 1500 mg/m² as a 1-h infusion on day 1 of each cycle (dose for children < 10 kg body weight: 50 mg/kg/day).

V. Recommendation in case of early progression
In case progressive disease is diagnosed and the commencement of radiotherapy shall still be deferred, the recommended chemotherapy is the use of Cisplatin/Vincristin and Cyclophosphamide/Vincristin as in the case of allergy.

C. Radiotherapy

When adjuvant treatment is indicated, radiotherapy can be the first line treatment in case of a small residuum amenable to stereotactic or conformal irradiation.
Older children, who upon indication for non-surgical therapy will receive external beam radiotherapy, will be irradiated with 54 Gy tumor dose conventionally fractionated at 1,8 Gy given on five days per week. The specific aims of the radiotherapy study are a maximal sparing of organs at risk by applying highly focussed radiotherapy with modern planning and technical equipment (see section 15.).

In case, that it is necessary to give radiotherapy to younger children, it is recommended to contact the national study chairmen for radiotherapy details.

A trial of prior chemotherapy could be considered to reduce the volume of a larger tumor.

D. Central neuroradiologic evaluation

Within the chemotherapy arm of the study central neuroradiologic assesment is mandatory and scans have to be sent in at definite time points (see section 8.5.):
- in order to validate radiological criteria for tumor progression and to confirm radiological or diagnostic imaging criteria (time point 1 and 2).
- to validate the response and assess the distribution of response at week 24 following induction treatment (time point 3),
- to define the point, when the “best response” throughout treatment is reached (time points 3 to 5)
- to compare subsequent scans (time point 6) where progression was deemed to have occurred in order to validate the time of progression.

Scans following radiotherapy will be assessed in a comparable pattern.

Qualitative changes of contrast enhancement will be described and correlated with response.
See section 8.5. for radiodiagnostic guidelines and section 16. for tumor response and remission criteria.

E. Treatment of Pure Optic Nerve Tumors

Where there is symptomatic or progressive tumor associated with demonstrable visual deterioration, and there is a strong need to initiate treatment to control symptoms and attempt to preserve vision, highly focussed radiotherapy should be considered. Primary chemotherapy may be an additional option. Children receiving chemotherapy for an isolated optic nerve glioma will not be eligible for randomization, yet.

F. Treatment recommendations following tumor progression

Despite all efforts to prevent tumor progression by primary therapy, a significant number of children will suffer from progression during or after first line therapy. Thus, the treatment strategy for low-grade gliomas has to incorporate recommendations for second (and third) line treatment approaches. In each case the possibilities for a meaningful surgical intervention should be checked, as well.

I. Progression during chemotherapy (early progression) in a young child (< 8 years)
Chemotherapy in these children is started to postpone radiotherapy. So, if PD occurs at the first evaluation at week 24 or later during consolidation and the child is still young, it is recommended that therapy be continued with the alternative chemotherapy regimen as in the case of Carboplatin allergy (section 14.1.3.). The two drug combinations of Cis-Platin/Vincristin and Cyclophosphmid/Vincristin are expected to offer an effective treatment.

II. Progression during chemotherapy (early progression) in an older child (≥ 8 years)
In case of progression at the first evaluation at week 24 or later during consolidation in a child older than 8 years, it should be assessed, if radiotherapy can be applied as second line therapy. If radiotherapy is no option, the alternative chemotherapy regimen (14.1.3.) should be used.

III. Progression following the end of chemotherapy
For children, who experience tumor progression following the end of therapy, several points have to be considered:
- age: Children still in the young age group, in whom radiotherapy should be further postponed, should receive second line chemotherapy.
  Children still in the young age group, in whom highly focussed radiotherapy appears possible, can go on to receive radiotherapy.
  Children in the older age group receive radiotherapy as second line therapy.
- time since the end of chemotherapy: If the first chemotherapy has been completed for more than a year, and the child has not had Carboplatin allergy, restart of standard Carboplatin/Vincristin chemotherapy may be taken into account. The alternative chemotherapy (14.1.3.) can be used as well.
  If the intervall between the end of first chemotherapy is shorter than a year and/or the child has had Carboplatin allergy, the use of the alternative regimen (14.1.3.) is recommended. The national chairman should be contacted to plan details for the "induction" phase.
- previous allergy: If first line chemotherapy has already been complicated by Carboplatin allergy and the alternative drug combinations thus have already been applied and second line chemotherapy is indicated, it is recommended to contact the national

chairman for the investigation of Phase II-treatment protocols. A trial with Vinblastin will be offered by the Phase II coordinators of the study.

IV. Progression following radiotherapy

For all children, who have received radiotherapy as their first treatment, chemotherapy is the first option in case of tumor progression. They will not be randomized and receive standard induction and consolidation with Vincristin and Carboplatin.

12.3. Study overview: Children affected by NF I (NF I+ve) with low grade glioma of all sites. SIOP LGG 2004

Within this trial all children affected by NF I and necessitating non-surgical therapy will receive the historical, but extended regimen with Vincristin / Carboplatin, regardless of their age at presentation. They should not be irradiated unless chemotherapy and surgery options have failed.

Preconditions to be stratified into this patient group:

Tumor location: All tumor locations

Histology: Low grade glioma according to section 9.
Histologic diagnosis is primarily made by the local pathologist, yet central pathologic review is strongly recommended.

Alternatively:
Clinical diagnosis: Neuroradiologic criteria for tumors of the optic pathways/chiasmatic-hypothalamic region fulfilled according to section 8.5.
Neuroradiologic criteria have to be fulfilled for all children not biopsied and central neuroradiologic review has to be obtained.

Surgery: Any extent of primary surgery

Neurofibromatosis I: present.

It should be noted that in very young children the signs of NF I may not be apparent and it is necessary in patients with tumors compatible with Neurofibromatosis that the patient is repeatedly re-evaluated in the first five to seven years of life for signs of emerging criteria (careful examination of skin is recommended).
Standard reassessment will be requested during follow-up at the age of six years.

Diagnosis of NF I
Diagnostic criteria for NF I are met in an individual, if two or more of the following are found (Definition of neurofibromatosis by NIH consensus statement 1988, Listernick 1997):

- Six or more café-au-lait macules of over 5 mm in greatest diameter in pre-pubertal individuals and over 15 mm in greatest diameter in post-pubertal individuals.
- Two or more neurofibromas of any type or one plexiform neurofibroma
- Freckling in the axillary or inguinal region.
- Optic pathways glioma
- Two or more Lisch nodules (Iris hamartoma) (Lisch 1937, Lubs 1991)
- Distinctive osseous lesion such as sphenoid dysplasia or thinning of the long bony cortex with or without pseudarthrosis
- A first degree relative (parent, sibling or off-spring) with NF I by the above criteria

Metachronous tumors – Patients with NF I are at risk to develop multiple (brain) tumors, especially if they presented with optic pathway glioma (Friedman 1997). Such metachronous

tumors have to be distinguished from secondary dissemination of a LGG. Thus, these tumors have an indication to therapy on their own. Contact with the national study chairman is recommeded.

Age eligibility: For patients with Neurofibromatosis age should not be used as a criteria for primary treatment stratification, since the restrictions for the use of primary radiotherapy apply to all ages.

Treatment strategy for children affected by NF I (NF I+ve) with low grade gliomas of all sites:

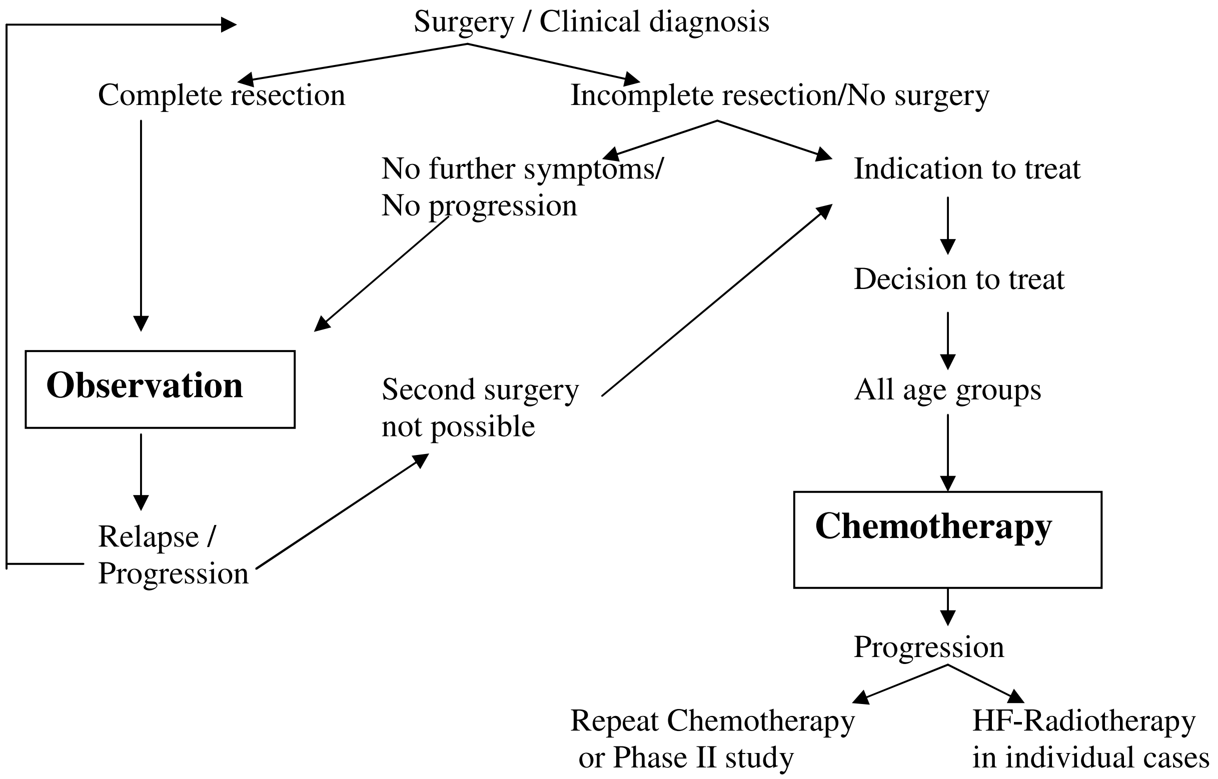

HF: highly focussed.

Treatment modalities:

A. Surgery

I. Surgery at diagnosis
The extent of surgical removal has to be discussed in view of tumor location and local or distant tumor extension and the risk of neurologic sequelae.

II. Second surgery
If there is the chance for a more complete tumor resection following primary tumor resection, or during observation or during the course of non-surgical therapy, this possibility should be discussed within the local treatment team, with the national study chairman and/or with the reference surgeon. Although tumor resections are recommended, the child should never be endangered by surgical intervention or suffer from additional severe neurologic impairment postoperatively.

B. Chemotherapy

Children, for whom there is an indication to be treated by chemotherapy and for whom the decision has been made to actually start treatment, shall receive standard induction and consolidation treatment with Vincristin and Carboplatin:

> **Note:** Neuroimaging should be obtained prior to start of therapy at an interval less than 4 weeks !

I. Standard Induction:
Vincristin is given once weekly as an iv-bolus at a dose of 1,5 mg/m²/day on day 1 of week 1, 2, 3, 4, 5, 6, 7, 8, 9 and 10 and then week 13, 17 and 21.
(maximum single dose: 2 mg, dose for children < 10 kg body weight: 0,05 mg/kg/day).
Carboplatin is given as an intravenous 1-hour-infusion at a dose of 550 mg/m²/day on day 1 of week 1, 4, 7 and 10, and then week 13, 17 and 21 (dose for children < 10 kg body weight: 18,3 mg/kg/day).

II. Consolidation
All children will receive consolidation therapy up to week 81 with ten 6-week cycles of Vincristin and Carboplatin. Cycles start in week 25, 31, 37, 43, 49, 55, 61, 67, 73 and 79.
Vincristin is given once weekly as an iv-bolus at a dose of 1,5 mg/m²/day on day 1, 8 and 15 of each cycle (maximum single dose: 2 mg; dose for children < 10 kg body weight: 0,05 mg/kg/day).
Carboplatin is given as an intravenous 1-hour-infusion at a dose of 550 mg/m²/day on day 1 of each cycle (dose for children < 10 kg body weight: 18,3 mg/kg/day).

III. Allergy
In case of Carboplatin allergy in children with NF I, the individual strategy for continuation of chemotherapy should be discussed with the national study chairman. Reasons that have substantiated the "chemotherapy only" strategy without randomisation of VP 16 still apply, so the choice of alternative chemotherapy should be cautiously made.

IV. Progression
If, during or after first line chemotherapy, the tumor progresses or if disseminated disease develops during or after chemotherapy and the patient is still in the young age category, consideration should be given to entering the patient into a phase II trial of novel agents or the use of alternative chemotherapy strategies from the published literature.

C. Radiotherapy

Note: The use of radiotherapy in NF I+ve patients is associated with increased risk of involved field short term and long term toxicity. If radiotherapy is considered, e.g. in older children with progression following chemotherapy, external beam radiotherapy will be applied with 54 Gy tumor dose conventionally fractionated at 1,8 Gy given on five days per week. The specific aims of the radiotherapy study are a maximal sparing of organs at risk by applying highly focussed radiotherapy with modern planning and technical equipment.

In case, that it is unavoidable to give radiotherapy to younger children, it is recommended to contact the national study chairmen for radiotherapy details.

D. Pure Optic Nerve Glioma

Where there is symptomatic or progressive tumor associated with demonstrable visual deterioration, and there is a strong need to initiate treatment to control symptoms and attempt to preserve vision, highly focussed radiotherapy or primary chemotherapy should be considered.

E. Treatment recommendations following tumor progression

Despite all efforts to prevent tumor progression by primary therapy, a significant number of children will suffer from progression during or after first line therapy. Thus, the treatment strategy for low-grade gliomas in children with NF I has to incorporate recommendations for second (and third) line treatment approaches. In each case the possibilities for a meaningful surgical intervention should be checked, as well.

Chemotherapy in these children is started to avoid or at least postpone radiotherapy. So, if PD occurs at the first evaluation at week 24 or later during consolidation or after the end of therapy, it is recommended that therapy be continued/restarted as chemotherapy either with the alternative chemotherapy regimen as in the case of Carboplatin allergy (section 14.1.3.) or with a Phase II-therapy. A trial with Vinblastin will be offered by the Phase II coordinators of the study.

If first line chemotherapy has already been complicated by Carboplatin allergy and the alternative drug combinations thus have already been applied and second line chemotherapy is indicated, it is recommended to contact the national chairman for the investigation of Phase II-treatment protocols.
If radiotherapy seems appropriate for older children, techniques of highly focussed irradiation should be used.

12.4. Study overview: Disseminated low grade glioma SIOP LGG 2004

Multicentric manifestation of low grade glioma is not infrequent at diagnosis or later during follow-up. All age groups are affected, but nearly one third of the patients is even younger than 1 year. Children with NF I do not seem to be affected, but may present metachronous primary tumors. Pilocytic astrocytomas (PA) with a primary tumor in the chiasmatic-hypothalamic region dominate. The slow-growing potential of PA probably persists even with multicentric spread.
For the purpose of this protocol this variant will be termed disseminated low grade glioma and the diagnosis be based upon MRI criteria and cytology (see 8.5. and 16.1.).
Routine spinal staging procedures are recommended in case of multifocal intracranial tumors, or cervical lesions found on cranial MRI or symptoms relating to spinal metastases.

Patients will be included into strategic group 1, 2 or 3 according to location of the primary tumor and absence or presence of NF I. They will be included in the analysis of the respective groups.

Tumor location: All primary tumor locations

Histology: Low grade glioma according to section 9.1.
Histologic diagnosis is primarily made by the local pathologist, yet central pathologic review is strongly recommended.

Alternatively:
Clinical diagnosis: Neuroradiologic criteria for tumors of the optic pathways/chiasmatic-hypothalamic region fulfilled according to section 8.5.
Neuroradiologic criteria have to be fulfilled for all children not biopsied and central neuroradiologic review has to be obtained.

Surgery: Any extent of primary surgery

Staging: Dissemination according to section 8.5. and 16.1.

Neurofibromatosis I: absent or present, although no dissemination has been reported in NF I patients in SIOP-LGG 1.

It should be noted that in very young children the signs of NF I may not be apparent and it is necessary in patients with tumors compatible with Neurofibromatosis that the patient is repeatedly re-evaluated in the first five to seven years of life for signs of emerging criteria.
Standard reassessment will be requested during follow-up at the age of six years.

Treatment strategy

A. Surgery
If possible, singular lesions should be removed surgically, however: multplicity of deposits or the presence of leptomeningeal lining will limit this approach.

Biopsy of disseminated lesions is encouraged however, especially to investigate histopathologic parameters, which might be associated with leptomeningeal seeding.

B. Non-surgical therapy

The question whether to proceed with radiotherapy or chemotherapy currently remains open and must consider the age of the patient, whether the patient has undergone radiotherapy for the primary tumor, and if so, the interval since the previous irradiation.

The presence of symptomatic multicentric disease at diagnosis or the emergence of multifocal tumors or their progression is considered to be an indication for non-surgical therapy (section 10.).

- Treatment with Vincristin and Carboplatin as scheduled within the SIOP - LGG 1996 protocol achieved response rates and progression free survival comparable to the sum of previous literature experiences. Considering the age of most patients primary chemotherapy for disseminated tumors along the principles applied to all other LGG is recommended.
  Prolonging therapy will probably prevent early progression, the effect of intensifying induction has to be investigated.

- In case of tumor progression following chemotherapy radiotherapy should be considered. Focal radiotherapy follows general guidelines. The concept of cranio-spinal irradiation for selected cases will be investigated. Doses and fractionation are detailed in section 15.

Treatment strategy for children affected by disseminated low grade gliomas:

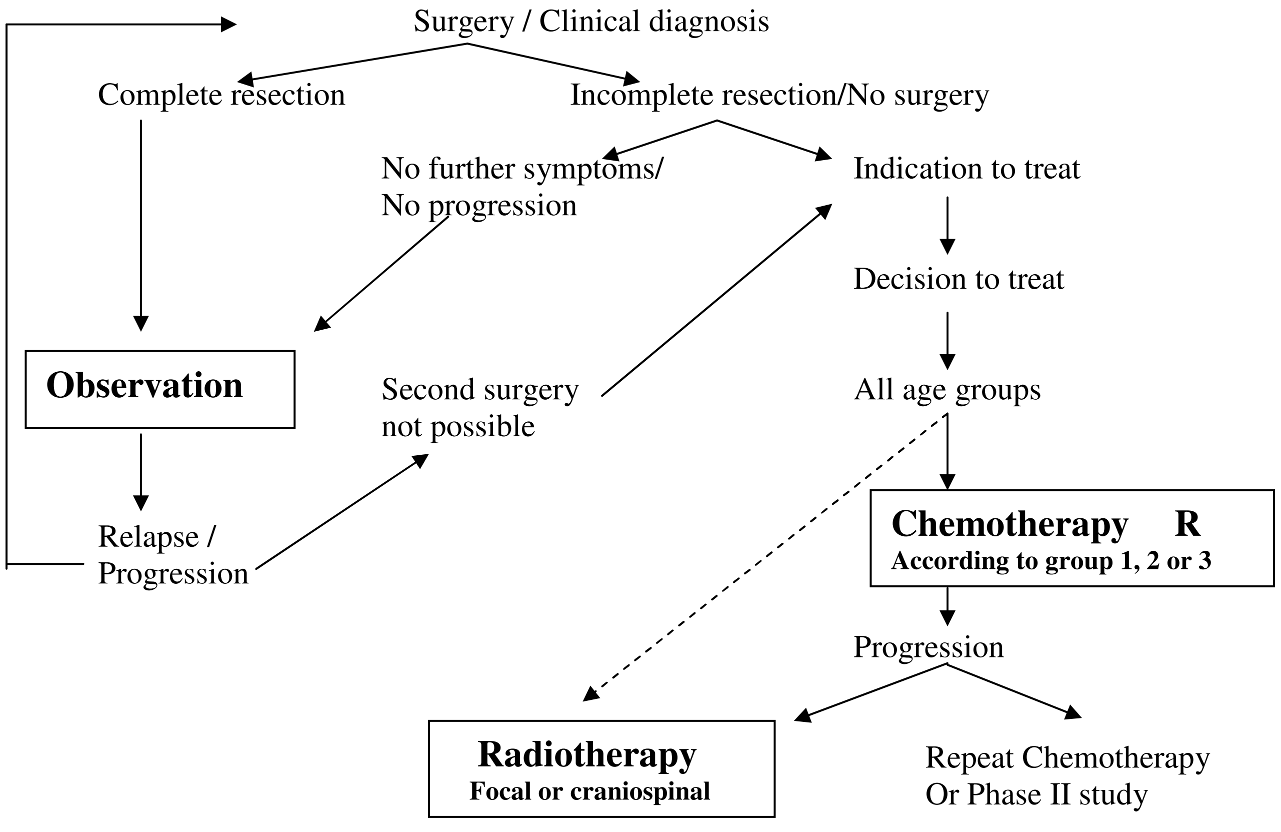

R: Randomisation of induction in treatment group 1 and 2.

13. Surgical guidelines SIOP LGG 2004

It is beyond the scope of this chapter to provide a comprehensive description of surgical approaches for low grade gliomas in different areas of the brain and spinal cord.

Treatment of low grade gliomas in different areas of the child's brain with different biological characteristics is still a challenging task for both: the neurosurgeon and the oncologist. With todays knowledge some guidelines can be established for treatment approaches. Nevertheless there still remain some surgical strategies based on individual decision. However, some recommendations may be useful to define the role of surgery within the treatment concept.

From the oncological point of view the strong association between the extent of resection and progression free survival favors radical surgery, at least for hemispheric, cerebellar and intramedullary tumors (section 3.2.). But, a number of low grade gliomas, like dysembryoplastic neuroepithelial tumors and gangliogliomas, remain quiescent even after incomplete resection. The striking difference in overall outcome between children and adults with low grade gliomas probably results from biologic differences, not well understood yet. Thus the more favorable outcome of children may be due to biological characteristics rather than to aggressive surgical interventions.

Recommended considerations:
1. During the surgical procedure tumor tissue should be sampled not only for conventional histology, but for the tumot tissue bank for future biologic investigations as well (see section 8.3. and details according to national procedures in the addendum 21.10.).

2. Early postoperative imaging is important to determine the extent of resection and has to be secured by the oncological team (see section 8.5. and 16.2.).

3. If postoperative imaging discloses that a potentially resectable lesion has been incompletely removed, second surgery has to be considered for gross total removal before proceeding with any adjunctive therapy.

4. To reduce surgical morbidity the use of technical adjuncts, like intraoperative ultrasound, frameless stereotaxy and neurophysiological methods, which facilitate tumor localization and intraoperative management, is strongly recommended.

13.1. Low grade glioma of the supratentorial midline in children not affected by Neurofibromatosis NF I (NF I-ve)

Management for tumors of these locations still is controversial, but the surgical procedure is determined by the answers to the following questions:
1. Can the tumor be classified by neuroradiological criteria with respect to location (located within the visual pathways) and thus to the possible low grade histology (no unusual findings pointing towards a tumor of higher malignancy) ?
2. Is the tumor potentially resectable without deterioration of the clinical symptoms and without inacceptable late effects ?

3. Is the space-occupying effects mainly determined by a cystic part of the tumor ?
4. Is the interruption of the circulation of cerebrospinal fluid due to the mass effect of the tumor ?

With regard to the location of the main tumor bulk and its potential origin this group of tumors will be divided into tumors originating within the visual pathways and tumors of the hypothalamus, basal ganglia and thalamus.

Tumors of the visual pathways
Extensive resection of optic pathway gliomas are burdened with substantial surgical morbidity with respect to vision and endocrine deficits and severe hypothalamic disturbance. Indications to perform surgery in this group of tumors may be

- to verify a low grade glioma histologically in cases not allowing a definite neuroradiological classification prior to the start of non-surgical therapy. Especially germinoma, Langerhans-cell histiocytosis and craniopharyngioma, but other histologies as well have to be excluded.
- to perform a primary partial resection in cases with a symptomatic exophytic portion of the tumor, which often is partially cystic, with mass effect and hydrocephalus: e.g. from the 3rd ventricle in case of hydrocephalus or from the temporal lobe in case of epilepsy.
- to perform a secondary partial resection or a biopsy upon progression during or following chemotherapy or before radiotherapy.

Tumors restricted to the optic nerve (anterior, intraorbital portion)
Resection of unilateral optic nerve glioma should only be performed in the presence of a blind eye and progressive exophthalmus. Otherwise a cautious wait-and-see policy should be followed and non-surgical options be preferred.

Tumors of the hypothalamus, basal ganglia, thalamus and mesencephalon
In case of a radiologically circumscribed tumor there is a definite indication to perform primary surgery. However, limiting factors for the extent of resection are a bilateral extension of hypothalamic tumors, the localization of a thalamic tumor within the dominant hemisphere or bithalamic involvement. In these cases only a biopsy or limited partial resection are feasable.
Focal tumors of the mesencephalon are often resectable, at least subtotally.
In tectal gliomas of typical radiologic appearance presenting with hydrocephalus due to stenosis of the aqueduct a third ventriculostomy should be performed as primary intervention. An attempt of tumor resection in typical tectal glioma is not indicated. However, if other mesencephalic tumors show progression during radiologic follow-up, histologic verification of a low grade glioma before non-surgical therapy is strongly recommended.

13.2. Low grade glioma of the all other sites in children not affected by Neurofibromatosis NF I (NF I-ve)

The resectability of low grade gliomas of the cerebral hemispheres, the cerebellum, the caudal brain stem and the spinal cord is determined by the exact location and the radiological growth characteristics (diffuse versus focal). For well circumscribed lesions a gross total resection should be the operative goal, if it can be achieved without major risk. Conversely, if following information from imaging, history and symptoms the differential diagnoses include the presence of a lesion not necessitating radical excision, primary stereotactic biopsy may be indicated to verify the histologic nature of the process.

Cortical and subcortical hemispheric tumors
In these locations primary complete surgery should be the goal. Pre- and intraoperative definition of functionally important cortical regions and subcortical tracts should be integral part of the planning procedure.

Deep hemspheric tumors extending towards the basal ganglia
The potential resectability depends upon the extension into adjacing tracts.

Cerebellar tumors
Complete resection is the goal of primary surgery, which, however, may not always be possible in lesions extending into the brain stem and the cerebellar peduncles.
Besides well known coordinative and motor tasks, the cerebellum actively contributes towards high mental function by processing cognitive and linguistic or emotional and social behavior. Surgical approaches to cerebellar tumors should take these facts into account, as well.

Tumors of the caudal brain stem
MR-classification has subdivided tumors of the brain stem into diffuse, focal, exophytic and cervico-medullary brain-stem gliomas. Complete resection should be discussed for focal lesions which may be reached without inacceptable morbidity.
- Focal tumors of the pons are rarely surgically accessible without severe surgical morbidity.
- Dorsally-exophytic lesions are focal tumors typically growing out of the medulla oblongata, extending into the cavity of the 4th ventricle, from where they can be resected.
- In non-exophytic, focal tumors of the medulla oblongata avoidance of permanent functional impairment has absolute priority. Even modern neurophysiologic monitoring during the surgical procedure cannot assure functional integrity in an attempted radical excision.
- Dorsally exophytic tumors of the cervico-medullary junction can often undergo gross total resection with excellent long-term prognosis even concerning morbidity.

Diffuse, intrinsic brain-stem gliomas are “non-surgical” tumors, in case of typical MRI-morphology there is no need for a biopsy. Since these tumors are considered of high-grade malignancy regardless of the exact histologic diagnosis, children and adolescents with such tumors are excluded from the protocol (see section 9.2.).

Spinal tumors
The majority of spinal intramedullary tumors in children are low grade gliomas. In the presence of focal tumors an attempt of radical resection may be performed. Multilevel laminotomy is needed for some of these extended tumors to avoid postoperative severe kypho-skoliosis. Intra-operative electrophysiological monitoring and ultrasonic aspiration of intramedullary tumors should be employed to reduce surgical morbidity.

13.3. Low grade glioma of any location in patients affected by Neurofibromatosis NF I (NF I+ve)

Considering the possible diagnostic categories for tumors in various locations the following recommendations can be made:

Visual pathway gliomas
There is no indication for a biopsy in lesions restricted to the visual pathways. Surgical resection has to be considered with even more reserve than in children without NF I, except in rare cases with space occupying lesions.

Tumors restricted to the optic nerve (anterior, intraorbital portion)
Resection of unilateral optic nerve glioma should only be performed in the presence of a blind eye and progressive exophthalmus. Otherwise a cautious wait-and-see policy should be followed and non-surgical options be preferred. Since an optic nerve glioma in a NF I patient may only be the initial manifestation of a more extensive involvement of the visual pathwas, resection may not prevent progression.

Tumors of all other locations
In case of primary contrast enhancing lesions of any other location, resection should be envisaged for all resectable or potentially life-endangering lesions, e.g. of the Foramina Monroi. Since outside the visual pathways children with NF I may develop tumors of all possible histologies, biopsy or resection have to be performed prior to the start of any non-surgical therapy.

In lesions without contrast enhancement radiological observation is recommended and surgical intervention (biopsy) should only be performed in case of unequivocal progression (MRI and/or MR-spectroscopy).

14. Chemotherapy SIOP - LGG 2004

14.1. Chemotherapy guidelines

14.1.1. Induction
- 14.1.1.1. Induction I.: Vincristin / Carboplatin
- 14.1.1.2. Induction II.: Vincristin / Carboplatin / VP 16

14.1.2. Consolidation: Vincristin / Carboplatin

14.1.3. Consolidation following allergy or early progression

14.1.4. Cumulative drug doses

14.2. Drug information

14.2.1. General guidelines for dosing and application of cytostatic drugs

14.2.2. Effects and side effects of cytostatic drugs, used in this protocol

14.2.3. Toxicity and dose modifications

14.2.4. Specific Organ Toxicities

14.2.5. Allergy to Carboplatin

14.3. Supportive care

14.1. Chemotherapy - Guidelines

- For all children chemotherapy consists of an **induction period** with a more compact schedule **from week 1 to 10** and a less compact phase **from week 13 to 21** and a prolonged **consolidation** therapy starting at **week 25 up to week 81**.
- Due to the facts that tumor response to chemotherapy occurs at a slow pace in low grade glioma with a median time to best response of 5,1 months (range: 0,9 - 25,3 months) in the previous study, determined for a cohort of 84 German patients (Gnekow 2000), and that objective tumor regression occurs even after an initial, often cystic, clinically asymptomatic tumor enlargement, the relevant **response assessment to induction therapy is timed at week 24**.

Week:					
	1 to 10	+	13 to 21	*24*	25 to 81
	Induction			*Response assessment*	Consolidation

- Induction therapy is randomized between Vincristin / Carboplatin and Vincristin / Carboplatin / VP 16:

Group 1: children unaffected by NF I with low grade glioma of the supratentorial midline (see 12.1.).
Group 2: Children unaffected by NF I with low grade cortical, cerebellar, brain stem and spinal glioma (see 12.2.).

- **Group 3**: Children affected by NF I with low grade glioma of all sites.

All children with NF I receiving chemotherapy will not be randomized and are to be treated with Vincristin / Carboplatin chemotherapy for induction (Induction I) and consolidation.

- **Disseminated low grade glioma**: Children with DLGG are part of one of the three treatment groups according to the location of the main/primary tumor and the absence or presence of NF I.

14.1.1 Induction therapy

14.1.1.1. Induction Therapy I - Vincristin / Carboplatin

Standard induction consists of Vincristin weekly and Carboplatin three-weekly for ten weeks as in the previous SIOP-trial. Following the 10 week-induction treatment the combination of Vincristin and Carboplatin is given three times at four week intervals to allow for recovery from hematologic and/or neurologic side effects of the induction phase.
Patient's clinical status evaluation (section 8.3.) should be performed regularly. Tumor status evaluation can be done upon individual decision at week 11 to 12, but moderate increments of the tumor dimension especially with regard to tumor cysts can be observed during the first weeks of therapy. Treatment response evaluation by neuroimaging has to be performed at week 24, deciding on the final response to this initial part of therapy and consequently on the subsequent treatment.

Vincristin is given once weekly as an iv-bolus at a dose of 1,5 mg/m²/day on day 1 of week 1, 2, 3, 4, 5, 6, 7, 8, 9 and 10 and then week 13, 17 and 21.
(maximum single dose: 2 mg, dose for children < 10 kg body weight: 0,05 mg/kg/day).

Carboplatin is given as an intravenous 1-hour-infusion at a dose of 550 mg/m²/day on day 1 of week 1, 4, 7 and 10, and then week 13, 17 and 21 (dose for children < 10 kg body weight: 18,3 mg/kg/day).

1	2	3	4	5	6	7	8	9	10	13	17	21	24	week
**V**	**V**	**V**	**V**	**V**	**V**	**V**	**V**	**V**	**V**	**V**	**V**	**V**		
**C**			**C**			**C**			**C**	**C**	**C**	**C**		
													**MRI**	

**V**	**Vincristin**	1,5 mg/m² (max. 2 mg)	iv-bolus - d 1
**C**	**Carboplatin**	550 mg/m²	1h infusion - d 1
**MRI**	**Neuroradiologic assessment of response by MRI**		

14.1.1.2. Induction Therapy II - Vincristin / Carboplatin / VP 16

Intensified induction consists of Vincristin weekly and Carboplatin three-weekly for ten weeks as in the previous SIOP-trial. Additionally VP 16 is given on day 1 to 3 of week 1, 4, 7 and 10 following the application of Carboplatin (d1 only). Following the 10 week-induction treatment the combination of Vincristin and Carboplatin is given three times at four week intervals to allow for recovery from hematologic and/or neurologic side effects of the induction phase.
Patient’s clinical status evaluation (section 8.3.) should be performed regularly. Tumor status evaluation can be done upon individual decision at week 11 to 12, but moderate increments of the tumor dimension especially with regard to tumor cysts can be observed during the first weeks of therapy. Treatment response evaluation by neuroimaging has to be performed at week 24, deciding on the final response to this initial part of therapy and consequently on the subsequent treatment.

Vincristin is given once weekly as an iv-bolus at a dose of 1,5 mg/m²/day on day 1 of week 1, 2, 3, 4, 5, 6, 7, 8, 9 and 10 and then week 13, 17 and 21.
(maximum single dose: 2 mg, dose for children < 10 kg body weight: 0,05 mg/kg/day).

Carboplatin is given as an intravenous 1-hour-infusion at a dose of 550 mg/m²/day on day 1 of week 1, 4, 7 and 10, and then week 13, 17 and 21 (dose for children < 10 kg body weight: 18,3 mg/kg/day).

Etoposide is given as an intravenous 1-hour infusion at a dose of 100 mg/m²/day on day 1 to 3 of week 1, 4, 7 and 10 (no dose adaptation for children < 10 kg body weight).

1	2	3	4	5	6	7	8	9	10	13	17	21	24	week
**V**	**V**	**V**	**V**	**V**	**V**	**V**	**V**	**V**	**V**	**V**	**V**	**V**		
**C**			**C**			**C**			**C**	**C**	**C**	**C**		
**Ex3**			**Ex3**			**Ex3**			**Ex3**					
													**MRI**	

**V**	**Vincristin**	1,5 mg/m² (max. 2 mg)	iv-bolus - d 1
**C**	**Carboplatin**	550 mg/m²	1h infusion - d 1
**E**	**Etoposide**	100 mg/m²	1h infusion - d 1 - 3
**MRI**	**Neuroradiologic assessment of response by MRI**		

14.1.2. Consolidation Therapy

As in the study SIOP/GPOH LGG 1996 consolidation is achieved by the continuous, simultaneous application of Vincristin and Carboplatin. However, treatment is prolonged up to week 81 by extending treatment intervals to 6 weeks, and Vincristin is given at a more intense schedule on day 1, 8 and 15 of each cycle.

Vincristin is given as an iv-bolus at a dose of 1,5 mg/m²/day on day 1 of week 25 to 27, 31 to 33, 37 to 39, 43 to 45, 49 to 51, 55 to 57, 61 to 63, 67 to 69, 73 to 75 and 79 to 81. (maximum single dose: 2 mg, dose for children < 10 kg body weight: 0,05 mg/kg/day).

Carboplatin is given as an intravenous 1-hour-infusion at a dose of 550 mg/m²/day on day 1 of week 25, 31, 37, 43, 49, 55, 61, 67, 73 and 79 (dose for children < 10 kg body weight: 18,3 mg/kg/day).

25	31	37	43	49	54	week
55	61	67	73	79	85	
**VVV**	**VVV**	**VVV**	**VVV**	**VVV**		
**C**	**C**	**C**	**C**	**C**		
					**MRI**	

**V**	**Vincristine**	1,5 mg/m² (max. 2mg)	iv-bolus<br>d1, 8, 15 of each 6 week cycle
**C**	**Carboplatin**	550 mg/m²	1h infusion<br>d1 of each 6 week cycle
**MRI**	**Neuroradiological assessment of tumor size / response**		

14.1.3. Consolidation therapy following allergy or early progression

Due to its allergic potential prolonged treatment with Carboplatin may not be possible. Since on the other hand, an extended treatment period may carry the potential for an extended progression free interval total treatment time shall be maintained, thus avoiding the necessity for early radiation. Alternative chemotherapy combinations shall be tested within such a continuation schedule. The drug-combinations have been studied for low grade glioma in various previous protocols (section 3).

The two combinations shall be given to a maximum of 5 times, to limit cumulative doses.
Vincristin is given as an iv-bolus at a dose of 1,5 mg/m²/day on day 1, 8 and 15 of each 6 week cycle starting with the first cycle post manifestation of allergy (i.e. weeks 1, 7, 13, 19, 25, 31, 37, 43, 49, and 55)
(maximum single dose: 2 mg, dose for children < 10 kg body weight: 0,05 mg/kg/day).

Cisplatin is given as an intravenous 3-hour-infusion at a dose of 30 mg/m²/day on day 1 and 2 of week 7, 19, 31, 43 and 55 (dose for children < 10 kg body weight: 1 mg/kg/day).

Cyclophosphamide is given as an intravenous 1-hour infusion at a dose of 1500 mg/m²/day on day 1 of week 1, 13, 25, 37 and 49 (dose for children < 10 kg body weight: 50 mg/kg/day).

Week post manifestation of allergy or early progression (maximum 5 cycles each):

1	7	13	19	25	31	37	43	49	55
**VVV**	**VVV**	**VVV**	**VVV**	**VVV**	**VVV**	**VVV**	**VVV**	**VVV**	**VVV**
**Cyc**	**Cisx2**	**Cyc**	**Cisx2**	**Cyc**	**Cisx2**	**Cyc**	**Cisx2**	**Cyc**	**Cisx2**

V: **Vincristine**	1,5 mg/m² (max 2 mg)	iv-bolus	d1, 8, 15 of each 6 week cycle
**Cis: Cisplatin**	30 mg/m²	3h infusion	d1 and 2 of each cycle
**Cyc: Cyclophosphamide**	1500 mg/m²	1h infusion	d1 of each cycle

14.1.4. Cumulative drug doses

Projected cumulative drug doses/m² for the entire length of chemotherapy are listed with respect to the different induction and consolidation regimens.

	Induction I	II	Post-Allergy Induction I	II
**Vincristine** (1,5 mg/m² iv)	64,5 mg	64,5 mg	64,5 mg	64,5 mg
**Carboplatin** (550 mg/m²/1h iv)	9350 mg	9350 mg	variable	variable
**Etoposide** (100 mg/m²/1h iv d1-3)	0 mg	1200 mg	0 mg	1200 mg
**Cisplatin** (30 mg/m²/3h iv d1+2)	0 mg	0 mg	300 mg maximum	300 mg maximum
**Cyclophosphamide** (1500 mg/m²/1h iv d1)	0 mg	0 mg	7500 mg maximum	7500 mg maximum

14.2. Drug Information

This section lists the most relevant drug actions and side effects, information for application and supportive measures. Side effects are mentioned only as far as they can be expected at doses used in this protocol according to current knowledge.
These guidelines do not exempt the treating physician from his/her obligation to inform himself/herself about the latest experiences with the respective drugs by use of the most recent publications and the information material provided by the drug companies, especially concerning the range of possible drug interactions.
The basic recommendations for the application of chemotherapy within the setting of this study may differ from local procedures. It is acknowledged that locally standardised procedures for the combination therapy of this protocol exist to which the details of this protocol may be adopted.

14.2.1. General guidelines for dosing and application of cytostatic drugs.

Despite the fact that chemotherapy within this protocol is designed primarily to postpone the early use of radiotherapy, its use for young children and for children with NF I has a profound impact upon their general prognosis. Especially those with diencephalic syndrome most often present as severely ill children. Thus, the intensity of this protocol is justified, but requires a responsible monitoring to avoid an inadequte amount of side effects.
On the other hand: indiscriminate dose reduction and unnecessary delay of chemotherapy has to be avoided. Each patient should receive the maximum recommended and tolerable dose of drugs at the appropriate time.

Dose modifications

I. <u>by age and weight:</u>
No randomised studies have been conducted to assess the relevance of dose adaptions for infants. Yet, it is considered appropriate that infants with a body weight below 10 kg should receive drug doses based upon body weight with a calculation of 1 m² body surface area equalling 30 kg:

Carboplatin	18,3	mg/kg
Vincristine	0,05	mg/kg
Cisplatin	1,0	mg/kg
Cyclophosphamide	50,0	mg/kg

For children below the age of 6 months further dose reduction of 1/3 is recommended. In case they do not experience relevant toxicity, dose adaption to dose/kg body weight can be considered.
Pharmacologic data for Etoposide have been explored demonstrating that drug reduction for young children above the age of 3 months even when weighing less than 10 kg are not necessary, so dosing as per m² of body surface area is safe (Boos 1992 and 1995).

Note: Infants are at a higher risk of Cis-Platin induced electrolyte imbalances and consequently regular electrolyte monitoring is particularly important in this age group. Correspondingly, the amount of hydration fluid has to be adjusted according to infants' weight and age.

II. by toxicity

The NCI Expanded Common Toxicity Criteria will be used for purposes of grading of toxicity (see Appendix 21.11.). Reqirements for starting therapy at normal dose and schedule are given for each drug below as well as dose modifications according to the extent of toxicity (section 14.2.3.). Yet recommendations within this protocol do not substitute for the responsibility of each treating physician to decide for each individual patient on site.
The national study chairman has to be contacted for any life threatening, lethal unexspected or unusual toxicity within 24 hours (For definition see section 16.4., report form in Addendum 21.12.).

Treatment intervals

Within the protocol treatment intervals lengthen gradually. They should therefore be maintained, if no undue toxicity intervenes. Postponing an element for 1 week is possible without modification. If intercurrent complications necessitate deferral for more than 2 weeks, dose reductions should be foretaken. In case of unexspected toxicity the national study center should be contacted.

Requirements to start therapy

- All elements: ◆Stable general condition.
  (exception: infants with diencephalic syndrome may be treated initially despite poor general condition, since only chemotherapy offers the chance to ameliorate their status.)
  ◆Children with a body weight less than or equal to the 3rd percentile at the time of starting therapy must have adequate enteral or parenteral nutrition.
  ◆No significant infection.

- Carboplatin: No allergy to Carboplatin ≥ grade 2 (alternatives: section 14.2.5.)
  Leucocytes >2,0/nl, Neutrophils >0,5/nl
  Thrombocytes >100/nl (rising)
  No hearing loss above 10-20 dB within the frequency range of 1-4 kHz.
  Normal renal function, nephrotoxicity not >grade 1.

- Cisplatin: Leucocytes >2,0/nl, Neutrophils >0,5/nl.
  Thrombocytes > 80/nl (rising)
  Ototoxicity not above grade 2
  Nephrotoxicity not >grade 1, Kreatinin-clearance not < 70 ml/min/1,73m²
  Peripheral neuropathy not >grade 2.

- Cyclophosphamide: Leucocytes >2,0/nl, Neutrophils >0,5/nl
  Thrombocytes >80/nl (rising)
  Nephrotoxicity not >grade 1

- Etoposide: Parameters of blood count as for Carboplatin.

- Vincristine: Peripheral neuropathy ≤ grade 2.

14.2.2. Effects and side effects of the cytostatic drugs, used in this protocol

Carboplatin (C)

Non-classical alkylating agent, impairment of DNA-synthesis by intra-strand and inter-strand bridging. Reacts as well with RNA, proteins and cell membranes.

Dose: 550 mg/m²/day as a 1 hour infusion
(dose for children < 10 kg body weight: 18,3 mg/kg/day).

Application:
◆ intravenous infusion in 200 ml Glucose 5 %/ m² for 1 hour
◆ a concommitant hydration pre- and postinfusion of the drug with 2000 3000 ml/m²/24 h is recommended with regard to the individual patient and allows to record the development of allergic reactions during the hospital stay
◆ sufficient hydration with careful monitoring of electrolytes, body weight and urine output is essential in infants with diencephalic syndrome
◆ sufficient antiemetic coverage

Side effects: Dose dependent, cumulative myelosuppression with a nadir between day 15 to 21 (Thrombocytopenia is more pronounced than Leucocytopenia)
Nausea, vomiting
Nephrotoxicity
Neurotoxicity
Ototoxicity
Allergy (see below)
Loss of Magnesium.

Interactions: Dexamethasone probably inhibits the effect of Platinum compounds in glial cells.

Monitoring: Severe nephrotoxicity has not been reported during Carboplatin-therapy. Yet, since elimination for the unmetabolised substance relies on glomerular filtration, renal function has to be monitored periodically (reduction of GFR to less than 50 % is less frequent than with Cisplatin). In case of a reduction in GFR the dose of Carboplatin can be calculated according to Calvert's formula: dose in mg = target AUC x (GFR+25). In most instances target AUC is 5-7 mg/ml/min (Calvert 1989).
Audiogramm
Blood count.
Substitution of Magnesium between treatments as with Cis-Platin.

Cisplatin (Cis)

Non-classical alkylating agent. DNA-cross-linkage and -point mutation, inhibition of DNA-repair, alkylation of RNA and proteins. Induction of apoptosis.

Dose: 30 mg/m²/day as 3 hour-infusion on day 1 and 2
(dose for children < 10 kg body weight: 1 mg/kg/day).

Application: ◆ Diuresis 3000 ml/m² from 6-12 hours before the first until 24 hours after the second dose of Cisplatin with adequate substitution of Mg and Ca
◆ Mannitol-bolus 40 ml/m² Mannit 20 % as a 10-15 min.-infusion before each dose of Cisplatin, parallel-infusion of Mannit 20 % 30 ml/500 ml to enforce adequate diuresis, avoid Furosemide
◆ Substitution of Magnesium 7 mg/kg/day p.o. for 2 to 4 weeks following Cisplatin
◆ sufficient antiemetic coverage

Side effects: Tubular-interstitial nephropathy
Neurotoxicity, especially irreversible high frequency auditory impairment, peripheral poly-neuropathy
Nausea, vomiting
Hypocalcemia, hypomagnesemia
Inappropriate secretion of ADH (SIADH)
Coombs-positive hemolytic anemia
Anaphylactic reactions.

Interaction: Synergistic cytotoxicity with Etoposide and other cytotoxic agents. Dexamethasone probably inhibits the effect of Platinum compounds in glial cells.

Monitoring: Renal function
Audiogram
Neurologic status
Electrolyte (Mg, Ca)- and fluid- balance.

Cyclophosphamide (Cyc)

Alkylating agent, Oxazaphosphorin.
Cytotoxic during S-Phase of the cell cycle, liver metabolism to 4-OH-Cyclophosphamid, Phospharamidmustard and Acrolein (urotoxic), metabolites can form covalent bonds to DNA or proteins.

Dose: 1500 mg/m²/day as a 1 hour infusion in 0,9 % NaCl
(dose for children < 10 kg body weight: 50 mg/kg/day).

Application: ◆ Diuresis and prophylaxis of hemorrhagic cystitis: 3000 ml/m² for 24 h
◆ Mesna 500 mg/m² per dose iv., before and 4 and 8 hours after the start of the Cyclophosphamide infusion
◆ 6 hourly registration of fluid balance, Furosemide 0,5 mg/kg iv, if needed
◆ controlling for hematuria (every portion of urine), in case of positive analysis for erythrocytes or dysuria the development of hemorrhagic cystitis is possible: increase hydration, increased/prolonged application of Mesna and pain therapy
◆ sufficient antiemetic coverage

Side effects: Myelosuppression (especially Granulocytopenia and Lymphopenia)

Hemorrhagic cystitis (Mesna !)
Renal water retention, tubular (Fanconi-syndrome) and glomerular nephropathy
Nausea, vomiting
Mucositis
Alopecia
Cytotoxic alveolitis
Cardiotoxicity
Changes of taste
Syndrome of inadequate secretion of ADH (SIADH)
Anaphylaxis, bronchospasm, dermatitis, Stevens-Johnson-syndrom,
Neurotoxicity
Liver toxicity.

As possible late effects infertility (disturbances of spermatogenesis and ovarian dysfunktion) and the development of secondary cancer (carcinogenic agent) have to be mentioned, but are unusual at low cumulative doses.

Interactions: Allopurinol, Cimetidin, Paracetamol, Barbiturates: increase of Cyc-effect and toxicity.
Amphotericin B: hypotension, bronchospasm
Insulin: increase of insulin-effect
Narcotics: increase of effect of narcotics.

Monitoring: Renal function.
Blood count.

Etoposide / VP 16 (E)

Epipodophyllotoxin
Inhibitor of the Topoisomerase II leading to single- and double strand DNA-breaks, reducing the capacity of DNA repair.

Dose: 100 mg/m²/d as a 1 hour infusion on day 1, 2, 3
(no dose adaption for children < 10 kg body weight)
Etoposide-phosphate can be given instead of Etoposide
(113,6 mg Etoposide phosphate equals 100 mg Etoposide).

Application : ◆ 1 hour infusion in normal saline (Na Cl 0,9 %) at a minimum dilution of 0,4 mg Etoposide/ml
◆ during and for 3 hours following the infusion the patient's blood pressure and heart rate should be monitored carefully
Decrease of the blood pressure and cardiac arhythmia can occur during VP 16 infusion. If this occurs, the infusion should be stopped and NaCl 0,9 % be given to restore normal blood pressure. Once symptoms resolve, the patient can be further challenged with VP 16 prolonging the infusion time
◆ sufficient antiemetic coverage

Side effects: Reversible bone marrow depression
Gastrointestinal: nausea and moderate vomiting

Mucositis,
Alopecia,
Rarely mild peripheral neuropathy
Rarely allergic reactions, blood pressure lowing in case of rapid infusion.

At high cumulative doses (above 5 g/m²) the risk for secondary myeloid leucemia is enhanced.

Interactions: Increased clearance at comedication with enzyme-inducing anticonvulsive drugs
Reduced clearance when given with high-dose Carboplatin.

Monitoring: Blood count
Integrity of mucous membranes
Blood pressure, monitoring for skin or respiratory signs of allergic reaction during infusion

Vincristine (VCR)

Vinca-alcaloid, extract from the evergreen Vinca rosea.
Blocking agent during M-phase of the cell cycle, inhibition of intracellular synthesis of tubulin. Disturbance of DNA and RNA-synthesis. Induction of apoptosis.

Dose: 1,5 mg/m²/day, maximum single dose: 2 mg,
(dose in case of body weight < 10 kg: 0,05 mg/kg/day)

Application:
- ◆ strictly intravenous bolus-injection, necrosis upon paravasation.
- ◆ ensure regular defecation.
- ◆ sufficient antiemetic coverage (if necessary)

Side effects: Peripheral neuropathy (reduction of peripheral tendon reflexes), paresis, myopathy, neuralgic pain, paralytic ileus, obstipation
Fever
Inadequate secretion of ADH (SIADH)
Cerebral convulsions
Myelosuppression
Alopecia
Cardiovascular disturbances
Photosensitation
Headache
Dysphagia, polyuria, dysuria
Dysfunction of cranial nerves, rarely atrophy of the optic nerve with amaurosis and transient cortical blindness.

Interactions (some are case reports only):
Cyclosporin A: increased neurotoxicity
Barbiturates: increased clearance of Vincristine
Histamin-2-antagonists: decelerated elimination of Vincristine
Itraconazol: increased polyneuropathy
Etoposide: synergistic effect, increased neurotoxicity (supposed)
Acetyldigoxin: reduced effect of Digoxin

Isoniazid: increased neurotixicity (single cases)
Metronidazol: increased neurotoxicity (case report)

Contraindication: Charcot-Marie-Tooth-syndrome.

Monitoring: Neurologic status (deep tendon reflexes, sensory neuropathy, bowel immotility).

14.2.3. Toxicity and dose modifications

**Carboplatin:**	Leucocytes <2,0/nl or Neutrophils <0,5/nl or Thrombocytes <100/nl at start of treatment	delay treatment for 1 week; if requirements are not met after 1 week delay: 25 % dose reduction for the next dose of Carboplatin.
	repeat sepsis during neutro-penia	25% dose reduction for the next dose of Carboplatin.
	progressive Ototoxicity at 1-4 kHz (> grade 2)	omit Carboplatin.
	Nephrotoxicity > grade 1	dose calculation according to the modified Calvert's formula
**Cisplatin:**	Leucocytes <2,0/nl or Neutrophils <0,5/nl or Thrombocytes <80/nl at start of treatment	delay treatment for 1 week; if requirements are not met after 1 week delay: 25 % dose reduction for the next dose of Cisplatin.
	Ototoxicity >grade 2 or Nephrotoxicity >grade 1 or Kreatinin-clearance: < 70 ml/min/1,73 m²	replace Cisplatin by Carboplatin
**Cyclophosphamide:**	Leucocytes <2,0/nl or Neutrophils <0,5/nl or Thrombocytes <80/nl at start of treatment	delay treatment for 1 week; if requirements are not met after 1 week delay: 25 % dose reduction for the next dose of Cyclophosphamide.
	Nephrotoxicity >grade 1	25 % dose reduction for the next dose of Cyclophosphamide.
**Etoposide:**	Hypotension	Prolong infusion time to 2-3 hours, Premedication with antihistamines.
**Vincristine:**	Peripheral neuropathy grade 3 or 4	omit the following dose/course of VCR; if neuropathy ameliorates resume therapy at 1,0 mg/m² VCR.

Convulsions<br>SIADH	omit the following dose/course of VCR, if no further convulsions or symptoms of SIADH occur, resume therapy at 1,0 mg/m² VCR (continuing any concurrent anticonvulsive treatment). If no further convulsions occur, following doses of VCR can be given according to schedule at 1,5 mg/m² (max. 2 mg)

Convulsions during chemotherapy generally need a diagnostic work-up including neuroimaging to rule out non-neurotoxic etiologies like bleeding or sinus vein thrombosis or tumor progression.

**All drugs:**	following severe neutropenia (ANC < 0,5/nl) associated with fever and sepsis or severe infection and/or severe thrompocytopenia (< 10/nl for > 5 days)	decrease dose 25 % for the next course<br>consider G-CSF for acute severe infection, but routine G-CSF is not recommended (see 14.3.)

14.2.4. Specific Organ Toxicities

Ototoxicity – The grading system for hearing loss proposed by P.R. Brock et al (1991) will be used in SIOP/GPOH LGG 2004 (Table 28). Careful monitoring of children by an expert audiologist and by serial audiometry throughout the treatment with Carboplatin and Cisplatin is recommended. To monitor ototoxicity in infants oto-acoustic emissions, when available, are a preferable technique to BEAR (brainstem evoked auditory response). Pure tone audiometry is the method of choice in children older than 3 years of age. If a child starts to show signs of high frequency hearing loss then he/she should be followed more carefully than the minimum requirement of this protocol. If grade 3 or 4 ototoxicity is documented Cisplatin should be withdrawn and replaced by Carboplatin, but if hearing continues to deteriorate, Carboplatin should be omitted as well.

Grading system for Cisplatin-induced bilateral high-frequency hearing loss

Bilateral hearing loss	Grade	Designation
< 40 dB at all frequencies	0	None
> 40 dB at 8,000 Hz only	1	Mild
> 40 dB at 4,000 Hz only	2	Moderate
> 40 dB at 2,000 Hz only	3	Marked
> 40 dB at 1,000 Hz only	4	Severe

Renal toxicity

a) Glomerular toxicity – Nephrotoxicity of CDDP in children (as in adults) is dose-related and sometimes severe. Plasma creatinine measurements and creatinine clearances are not reliable guides to the degree of CDDP-induced renal damage, particularly in children. Careful measurement of Glomerular Filtration Rate (GFR) by isotope clearance is more accurate. DTPA and other scans are useful for national comparative studies, but for the purpose of this study GFR should be documented.
It should not be done when a child is receiving iv.-hydration. The same technique for assessing GFR should be used at every time point in an individual child.
A standard endogenous creatinine clearance requires a 24 hr urine collection. If the urine collection is not complete, then please repeat it. Cr51 EDTA GFR is the preferred technique during CDDP treatment and involves obtaining the isotope, injecting it into the child and taking 4 blood samples at hourly intervals from an indwelling catheter. It entails less irradiation to the child than daily natural sources. The technique is well described by Chantler et al (Clin Sci 1969; 37:169-180 and Arch Dis Child 1972; 47:613-617).

In cases of severe reduction in CR-51-EDTA GFR (<60ml/min/1.73 m^2), discontinue CDDP and use Carboplatin. If GFR falls below 2 SD of the expected GFR according to age in infants, Carboplatin should be substituted for CDDP.

b) Tubular toxicity – A way of monitoring tubular function is by phosphate clearance and phosphate reabsorption and by pattern of protein excretion and by β2-Micro-globulin.
Renal loss of Magnesium and consequent hypomagnesemia is expected in nearly all children on this study and oral Magnesium supplementation is recommended for all children entered into study. Hypomagnesemia is <u>not</u> a reason to stop CDDP. Children can develop other manifestation of <u>renal tubulopathy</u> at the same time as the GFR is improving. Thus, careful electrolyte monitoring is essential in all children exposed to CDDP treatment. Hypomagnesemia may persist years after stopping therapy.

14.2.5. Allergy to Carboplatin

As a whole 18 (14.5%) of 124 patients of the SIOP - LGG study cohort had allergic reaction to Carboplatin at a time interval between the beginning of chemotherapy and “allergy” ranging from 1 to 45 weeks (median 27 weeks). However, this could be an underestimation of the real incidence of the problem; in fact among the Italian patients 15 out of 35 children (40%) actually manifested allergic reaction to Carboplatin. Changes in the strategy of the present study may reduce the incidence of allergy, but clinicians should be alert at each dose of Carboplatin, that there is a possibility for severe reactions, even if previous doses have been tolerated well.

For hypersensitivity reactions to Carboplatin, reactions of grade I on one occasion would permit the repeated administration of Carboplatin subsequently with close surveillance, pre-medication with anti-histamine and hydrocortisone and slowed infusion rate (e.g. 4 hours). If grade II (or above) reactions occur, Carboplatin should not be used thereafter:

Grade I	Mild rash	Grade III	Bronchospasm
Grade II	Urticaria	Grade IV	Allergic shock

Consolidation therapy following Carboplatin allergy:

- In case of relevant hypersensitivity the study committee discourages the attempt to continue therapy by methods of desensitisation.
- Instead, since in most cases allergy will develop during consolidation, it is recommended to omit Carboplatin and to continue treatment by alternating the two elements Vincristine/Cyclophosphamide and Vincristine/Cisplatin.
- If possible, **total treatment time should be maintained**, however cumulative doses should be observed to avoid untolerable organ toxicity. A maximum of 5 cycles each of Cisplatin and Cyclophosphamide, respectively, shall not be exceeded.
- The following **sequence of cycles** is recommended (for details see 14.1.3.):

**1**	**7**	**13**	**19**	**etc.**	week post manifestation of allergy
**VVV**	**VVV**	**VVV**	**VVV**	V: **Vincristine**	1,5 mg/m² iv-bolus (max. 2 mg) - d1, 8, 15 of each 6 week cycle
**Cyc**	**Cis**	**Cyc**	**Cis**	**Cis: Cisplatin**	30 mg/m² 3h infusion - d1+2
				**Cyc: Cyclophosphamide**	1500 mg/m² 1h infusion, d1

- Another alternative is the substitution of Carboplatin by **Actinomycin D** according to the protocol used by Packer (Packer 1988b), but care should be taken to avoid the occurrence of veno-occlusive disease (see below).

Actinomycin D

Antibiotic
Inhibition of DNA synthesis by intercalation, blocking of replication and transcription of the DNA-template. May also cause topoisomerase-mediated single strand breaks in DNA.

Dose: 15 µg / kg / d as iv.-bolus injection on day 1 to 5

Application:
◆ Intravenous bolus injection
◆ Sufficient antiemetic coverage

Side effects:
Gastrointestinal irritation (nausea vomiting, diarrhoea, ulcerative stomatitis, gastroenteritis)
Hepatotoxicity (venoocclusive disease (VOD), particularly in young children)
Bone marrow depression
Alopecia
Exanthema
Extravasation may cause severe local and regional ulceration

Interactions: Radiation sensitizer and radiation recall effect.

Monitoring:
Hepatic function and portal vein blood flow
Blood count

14.3. Supportive Care

All treatment here, even if tolerated well by the individual patient, has to be considered potentially intense and aggressive. Hence, treatment according to the guidelines of this protocol should be restricted to institutions, who are familiar with the administration of intensive aggressive combination chemotherapy and where the full range of supportive care is available.

Antiemetic therapy
All the chemotherapeutic agents, but VCR, can cause severe nausea and vomiting. Thus an appropriate antiemetic coverage is necessary before instituting therapy and at least for 24 hours after the end of therapy.
Antiemetic therapy should be administered according to institutional policy, e.g. odansetron 5 mg/m² (maximum single dose 8 mg) p.o./i.v. every 12 hours. Especially following the application of Cisplatin late emesis should be considered and the application should be prolonged.

Infection prophylaxis
Pneumocystis carinii prophylaxis is mandatory according to the recommendation of the national groups, which will be most often the prescription of Trimethoprim/ Sulfmethoxazol (5-6 mg/kg TMP or 30 mg/kg SMZ) on two to three days per week.

Central lines
The use of central lines is recommended, especially for small children.

Blood component therapy
Due to the risk of graft versus host reactions in infants as well as in patients under chemotherapy all blood products should be irradiated with at least 20 Gy (regularly 30 Gy) prior to transfusion, according to national policies. The use of leukocyte filters for leucocyte depletion is advised (in CMV negative patients), if there is no in-line filtration at the time the blood is taken.

Granulocytes colony stimulating factors (G-CSF)
The use of Granulocytes stimulating factors is not routinely recommended in children treated according to the protocol.
However, in case of a delay of one or more additional weeks in meeting the hematologic criteria for starting therapy instead of decreasing dosage by 25 % for the next course the use of granulocytes colony stimulating factors can be considered.
Similarly, if a course of chemotherapy is complicated by fever and sepsis or severe infections the use of G-CSF is suggested.
Routine dosage for this purpose is 5 µg/kg body weight sc. Filgrastim or 150 µg/kg body weight sc. Lenograstim. It is suggested to proceed until a stable absolute neutrophil count > 5,0 / nl is documented.

Endocrine function monitoring
Due to the location of the supratentorial midline low grade gliomas a significant portion of patients will either exhibit endocrine disturbances upon diagnosis or develop such during treatment or later follow-up. Regular assessments especially for thyroid function and corticosteroid secretion should be ensured during chemotherapy (section 8.4.).

Contraception
Pregnancy has to be prevented in fertile adolescent girls during chemotherapy by reliable anticonceptive methods, e. g. by hormonal anticonception.

Psycho-social support
Qualified psycho-social support for patients and their families should be an integral part of the treatment strategy. Faced with a tumor that may endanger life not immediately, yet rather throughout many years, but that carries along the risk for severe functional impairment, many adaptive processes have to be coped with. Especially loss of vision necessitates profound educational and rehabilitative measures. Moreover, social issues must be dealt with.Thus, continuous support should be offered to the patient and all other familiy members in cooperation with the medical staff.

15. Radiotherapeutic guidelines SIOP LGG RT 2004
Cooperative, prospective therapy protocol

NATIONAL REFERENCE CENTERS for RADIOTHERAPY:

United Kingdom:
Dr. R.E. Taylor
Department of Radiotherapy
and Oncology
Cookridge Hospital
Hospital Lane
Leeds / Cookridge
West Yorkshire LS16 6QB
Tel.: ++44 113 3924397
Fax: ++44 113 3924052
e-mail :zoea@ulth.northy.nhs.uk

Germany:
Rolf - D. Kortmann.
Department of Radiotherapy
University of Leipzig
Härtelstr. 16
D - 04107 Leipzig
Tel.: ++49 (0)341 9718542
Fax: ++49 (0)341 9718549
e-mail : rolf-dieter.kortmann@medizin.uni-leipzig.de

Spain
Gordi Giralt
Oncologica radiotherapica
Pg Vall d'Hebron 135
E - 08035 Barcelona – Spain
Tel: ++34 93 27 48 086
Fax: ++34 93 274 60 59
e-mail: giralt@hg.vhebron.es

France
Christian Carrie
Radiotherapy Department
Centre Léon Bérard, 28, rue Laënnec
F - 69373 Lyon
Tel: ++33 4 78 78 28 85
Fax: ++33 4 78 78 26 26
e-mail: carrie@lyon.fnclcc.fr

Italy
Giovanni Scarzello
Department of Radiotherapy
Padua General Hospital
Via Giustiniani 2
I - 35100 Padua
Tel.: ++39 0498212960
Fax : ++39 049 8212957
e-mail : g.scarzello@unipd.it

Austria
Karin Dieckmann
Department of Radiotherapy
Universitätsklinikum Wien
Währinger Gürtel 18-20
A - 1090 Wien
Tel.: ++43 1 404 00 96 65
Fax ++43 400 26 90 / 93
e-mail : Karin.Dieckmann@str.akh.magwien.gv.at

Brachytherapy
Prof. Dr. C. Ostertag
Neurochir. Universitätsklinik
Department of stereotactic Neurosurgery
Neurozentrum
Breisacherstr. 64
D - 79106 Freiburg
Tel.: ++49 761 270 5063
Fax: ++49 761 270 5010

15.1. Introduction and Background SIOP LGG RT 2004

15.1.1. ROLE OF RADIATION THERAPY

The role of post-operative radiotherapy in adult low grade glioma now appears clearer following a report from the EORTC study showing that an improvement in progression-free but not overall survival is obtained after immediate post-operative radiotherapy [Karim et al., 2002]. However, a reliable identification of prognostic factors supporting the use of immediate postoperative radiotherapy is still lacking for children. Presently, it is recommended to employ radiotherapy in progressive disease only [Listernick et al., 1997]. In younger children chemotherapy is preferred to defer radiotherapy until further progression. For modern treatment techniques such as fractionated conformal techniques. preliminary data exist though with limited patient numbers yielding promising results [Merchant et al., 2002b;Debus et al., 1999].
For all locations extent of resection of a low grade glioma is the factor associated most strongly with progression-free survival favoring complete tumor removal (see section 3.2.). Following complete tumor removal radiotherapy does not seem necessary.

15.1.1.1. Glioma of the cerebral hemispheres

Disease progression is rarely observed after complete resection of low grade gliomas of the cerebral hemispheres in children [Fisher et al., 2001;Forsyth et al., 1993;Pollack et al., 1995;Sutton et al., 1995], so these children do not need radiotherapy.
However, even with incomplete tumor removal prolonged progression-free survival is commonly achieved [Forsyth et al., 1993].
Radiotherapy is reserved for tumor progression and non-resectable relapse. It offers an additional benefit by improving focal neurological deficits. In the series of Fischer et al. 9 of 15 children demonstrated focal neurological disorders before receiving radiotherapy and 7 of these 9 patients showed significant improvement [Fisher et al., 1998].

15.1.1.2. Cerebellar glioma

Complete surgical resection, as judged by postoperative neuro-imaging and operative record , appears possible in 84 to 90 % of all patients [Gajjar et al., 1997]. Incomplete removal is associated with tumor extension into the brainstem, leptomeningeal infiltration and for tumors encircling cranial nerves. Though extended periods of stable disease, and sporadic cases of tumor regression, following partial resection are reported for small numbers of patients, residual tumor tends to progress over long periods of time, mostly within 4-5 years after initial operation, and progression free survival rates are between 29 to 80 % and 0 to 79 % at 5 and 10 years [Dirven et al., 1997; Garcia et al., 1989; Gjerris et al., 1978; Schneider, Jr. et al., 1992; Smoots et al., 1998]. Small numbers of children have been irradiated with progressive or relapsing tumors only.

15.1.1.3. Gliomas of the supratentorial midline (visual pathways and hypothalamus)

Several series have demonstrated a poor outcome in patients with chiasmal tumor managed conservatively without radiation, demonstrating a survival advantage for children receiving irradiation.. In the report by Tenny et al. only 3 of 14 (21 %) survived.
after biopsy or exploration only compared to 28 of 44 (64 %) who received radiotherapy [Tenny et al., 1982]. In the series of Montgomery et al. of 16 patients undergoing radiation

therapy, 12 patients were alive without evidence of disease at a mean follow up of 6.3 years [Montgomery et al., 1977] (Table 29).

Table 29: Visual function / visual field after radiotherapy of gliomas of the optic pathway

Author	N	Total dose (TD)<br>Daily fraction (FD)	Improved	Stable	Worse
Taveras et al., 1956	22	8 to 15 Gy	Vision<br>11 (50%)	8 (36.4%)	3 (13.6%)
Montgomery et al., 1977	12	TD 35 - 65 Gy<br>(almost all 50 Gy)<br>FD n.m.	Vision<br>3 (25%)	9 (75%)	0
Hoyt and Baghdassarian, 1969	28	n.m.	Acuity<br>4 (14.3%)	18 (28.6%)	6 (21.4%)
Dosoretz et al., 1980	9	TD 37-55.8 Gy<br>FD 1.0-2.0 Gy	Vision<br>1 (11.1%)	8 (88.9%)	0
Kalifa et al, 1981	39	TD : 50 – 60 Gy<br>FD : n.m.	Vision<br>7 (19.9%)	30 (76.9%)	2 (5.1%)
Horwich and Bloom,1985	23	TD 45-50 Gy<br>FD 1.8-2.0 Gy	Acuity (23)<br>10 (43%)<br>Vis.field (23)<br>4 (18%)	11 (48%)<br>19 (82%)	2 (9%)<br>0
Danoff et al, 1980	18	TD 50 – 60 Gy<br>FD 1.8-2.5 Gy	Vision<br>6 (33%)	8 (44%)	4<br>(22%)
Weiss et al., 1987	12	TD : 40 – 56 Gy<br>FD : n.m.	Vision<br>3 (25%)	9 (75%)	0
Flickinger et al.,1988	22	TD 38-56.86 Gy<br>FD 1.4-2.0 Gy	2 (9%)	14 (77%)	3 (14%)
Wong et al., 1987	17	TD 35-61 Gy<br>FD 1.5-2.0 Gy	6 (35%)	9 (53%)	2 (12%)
Pierce et al., 1990	23	TD 45-56,6 Gy<br>FD 1.8-2.0 Gy	23 (30%)	14 (61%)	2 (9%)
Rodriguez et al., 1990	15	TD 43 – 60 Gy<br>FD : n.m.	Vision<br>3(20%)	8 (53.3%)	1 (6.6%)
Bataini et al., 1991	44	TD 40-60 Gy<br>FD 1.45-2.15 Gy	Acuity<br>25 (57%)<br>Vis. Field<br>19 (61%)	16 (36%)<br>11 (35%)	3 (7%)<br>1 (3%)
Tao et al., 1997	29	TD : 50.4.-55.8 Gy<br>FD : 1.8-2.0 Gy	Vision<br>7 (24.1%)	14 (48.3%)	5 (17.2%)
Erkal et al., 1997	13	TD 40-60 Gy<br>FD 1.8-2.0 Gy	9 (34%)	14 (54%)	3 (12%)
Grabenbauer et al, 2000b	25	TD 45-60 Gy<br>FD (1.6 – 2.0 Gy)	Acuity (25)<br>9 (36%)<br>Vis. Field (20)<br>3 (15%)	13 (52%)<br>16 (80%)	3 (12%)<br>1 (5%)

n.m = not mentioned; Vis. field = visual field

Impact on visual function

Radiotherapy has become a standard treatment of optic nerve and chiasmatic gliomas since Taveras et al. reported improvement in visual acuity in 11 of 22 patients without noting any

morbidity associated with irradiation [Taveras et al., 1956]. Numerous reports over the years consistently support the high efficacy (90 %) of radiotherapy in stabilizing and improving visual function (Table 29). In contrast to these studies Dutton in his analysis of 1136 patients failed to confirm a benefit of radiotherapy [Dutton, 1994]. Among 511 patients treated with radiotherapy and followed for up to 10 years, 354 (69 %) showed stable or improved vision. 203 similar patients were followed without radiotherapy. 156 (77 %) showed visual stability or improvement. In this study it can be assumed, that a conservative approach without treatment was taken in the majority of patients with clinically stable tumors, whereas the proportion of patients with progressive tumors probably was higher in the cohort undergoing radiotherapy. This supports the “wait and see”-policy for non-progressive tumors.
The data on visual outcome are often difficult to judge as standards for evaluation are not existing and the description on visual function were cursory only in the majority of series. These shortcomings mandate a standardized approach in evaluating visual function both when deciding for treatment and at the time of assessing response to treatment.

15.1.1.4. The role of brachytherapy

Interstitial brachytherapy is a useful alternative in selected cases (Ostertag, 1989). The purpose of interstitial brachytherapy is to deliver a focal necrotising radiation dose within the tumor while sparing normal surrounding tissue. There is a steep dose gradient at the periphery thereby leaving a high cumulative dose around the implanted radioactive seeds, most commonly Iodine-125 (Ostertag, 1989). The largest series of interstitial brachytherapy in childhood and adult low grade glioma was published by Kreth et al. [Kreth et al., 45]. A total of 455 patients with low grade glioma were treated by using I-125 either as permanent or temporary implants. The 5- and 10-year survival rates in 97 patients with pilocytic astrocytoma were 85 % and 83 % and in patients with WHO grade II astrocytomas (250 patients) 61 % and 51 %, respectively. One hundred and twenty four of 455 patients were children and adolescents, 54 had a WHO grade II glioma, 70 a pilocytic astrocytoma. A 5 year survival rate of 84 % was obtained in astrocytoma WHO II and 90 % in pilocytic astrocytomas. Clinical stability was reported to be maintained throughout the survival time in all children. However, the data were not specifically analysed with respect to the pediatric cohort within this series. Voges treated 19 children with deep seated glioma, 13 of whom had low grade histology. (Voges et al., 1990). Tumor shrinkage could be seen on CT scans in all children and the estimated 4.5 year survival probability was 92%. Transient radiation induced edema was seen in 5 children. Although it is nearly impossible to define precisely which tumors are suitable for interstitial brachytherapy, with the available data it seems that small, circumscribed deep seated tumors with a diameter of less than 4 cm in locations other than the optic nerve and chiasm are preferred cases for interstitial radiosurgery.

15.1.1.5. The role of proton therapy

The major advantage of proton therapy over conventional radiation techniques is the high degree of dose conformity around the tumor that can be achieved, since protons have no exit dose beyond the target. Only one report has been published. The working group of Loma Linda treated 27 pediatric patients with progressive or recurrent gliomas at various sites [Hug et al., 2002](Table 4). Target doses were between 50.4 and 63.0 CGE (Cobalt Gray Equivalent) at 1.8 Gy per fraction. At a mean follow-up period of 3.3 years 6 patients experienced local failure and 4 died of disease. By anatomic sites these data translated into rates of local control and survival of 87%/93% for midline tumors, 71 % / 86 % for hemispheric tumors and 60 % / 60 % for brainstem tumors. The authors stated that their results were very encouraging especially for larger, irregular shaped tumors along the visual

pathway, where dose conformity is of particular importance. The limited access to proton therapy is the major disadvantage. However, intensity modulated radiotherapy will achieve similar dose conformity and it is most likely that this modern technique can be performed in the majority of institutions in not too distant future.

15.1.2. TIMING OF POSTOPERATIVE RADIOTHERAPY

Several retrospective studies have indicated an advantage for immediate postoperative radiotherapy regarding overall survival and progression - free survival in adults [Garcia et al., 1985;Shaw et al., 1989;Shibamoto et al., 1993], although there are opposite observations [Grabenbauer et al., 2000a]. Recent results of an EORTC/MRC study have shown that immediate postoperative radiotherapy in low grade glioma improved progression-free survival over that seen with observation only (5-year progression-free survival rates : 44% versus 37%, p=0.02). This benefit, however, was not translated into an improvement in overall survival [Karim et al., 2002].

15.1.2.1. Hemispheric and cerebellar low grade glioma

Forsyth et al. observed that immediate postoperative radiotherapy had an impact on overall survival in 39 patients with supratentorial pilocytic astrocytoma [Forsyth et al., 1993]. A policy of surveillance alone after surgical management was retrospectively analyzed in most series. In the series of Fisher tumor progression occurred in 12 of 48 patients (25 %) receiving immediate postoperative irradiation after incomplete resection, whereas the rate of progression was 42 % among 55 patients in whom radiotherapy was deferred (Fisher 2001). Postoperative radiotherapy has been employed for patients with residual, progressive or recurrent cerebellar astrocytoma in a rather unsystematic pattern. Garcia et al. noted that of 21 patients locally controlled after incomplete resection 16 were irradiated [Garcia et al., 1989]**.** In a previous analysis on the same patients, the cohort of 26 patients receiving immediate radiotherapy experienced a prolonged progression-free survival which was translated into a trend towards a better overall survival as compared to 16 patients undergoing surgery alone (70 % versus 60 % survival rate) [Garcia et al., 1990]. In other series, however, this observation could not be confirmed [Dirven et al., 1997;Gjerris et al., 1978;Schneider, Jr. et al., 1992;Smoots et al., 1998].

Table 30: Impact of immediate, delayed or no radiotherapy on progression or overall survival.

Author	N (age)	Tumor location	Extent of resection	Result
Pollack et al 1995	49	Cerebral hemisphere	Subtotal	10 y PFS<br>82 % immediate RT (n=33)<br>40 % no RT (n=16) p 0,014
Fisher et al 2001	128<br><br><18y	Cerebral and cerebellar hemisphere<br><br>(median follow-up: 7,3 y)	Complete (25)<br><br><br>Subtotal (103)	PFS 5y 100 %<br>OS 5y 100 %<br><br>RT deferred postOP<br>(N: 55 48)<br>PFS 5y 69 81 %<br>10y 55 68 %<br>OS 5y 87 81 %<br>10y 83 73 %

Thus, it is justifiable to defer radiotherapy for cerebral and cerebellar tumors until non-resectable relapse or tumor progression is observed.

15.1.2.2. Low grade gliomas of the supratentorial midline (visual pathway)

Jenkin et al. addressed this question in a retrospective analysis [Jenkin et al., 1993]. For thirty-eight patients receiving postoperative radiotherapy and 49 patients undergoing surveillance. No difference in progression free and overall survival rates could be detected (65 % versus 65 % and 69 % versus 80 % at 15 years), although more residual disease in the radiotherapy group may have adversely influenced outcome. In the study from St Jude's hospital, radiotherapy was used only in case of progressive disease [Gajjar et al., 1997]. One hundred and seven out of 142 children with tumors of all sites were observed, while 31 patients received radiotherapy and 4 patients chemotherapy (they were younger than 5 years of age), respectively, when showing progressive disease. The progression-free survival and overall survival rates of all patients were 70 % and 90 %, respectively, whereas the overall survival rate was only 65 % at 4 years in children after treatment for progressive disease. By contrast, in the series of 29 patients reported by Tao et al the policy to treat with radiation therapy as determined by clinical progression or increase in tumor size on imaging achieved a better result with a 15 year progression-free survival rate of 82.1 % and overall survival rate of 85.1 % [Tao et al., 1997]. The strategy to postpone the necessity for radiotherapy until time to progression was investigated in the SIOP / GPOH LGG trial 1996. Children 5 years of age and older received radiotherapy as first line non-surgical treatment, whereas children younger than 5 received chemotherapy in progressive disease. Preliminary data in 96 patients show that a 3 year progression – free survival rate of 87.1 % and an overall survival rate of 95.7 % can be obtained by radiotherapy [Kortmann et al., 2000b].

15.1.2.3. Radiotherapy following chemotherapy

The effect of radiotherapy after chemotherapy has failed is unclear. In the series of Janss et al. 46 children under the age of 5 years received first line chemotherapy [Janss et al., 1995]. Seventeen children finally received radiotherapy because of progressive disease. Seven of 17 children who required radiation after chemotherapy have incurred a third progression and the second progression free survival was 29 % at 10 years. It appears that this subset of patients represents a cohort with biologically more aggressive tumors and the additional question of whether chemotherapy renders the tumors more radio-resistant needs to be considered. By contrast, in an interim analysis of the SIOP - LGG trial a reduced efficacy after chemotherapy could not be observed [Kortmann et al., 2000b]. In this study 23 of 96 patients received radiotherapy after chemotherapy had failed. Although the follow-up was too short to draw reliable conclusions the progression free survival and overall survival rates did not differ from patients having received radiotherapy as first line treatment (91.3 % versus 87.3 % and 100% versus 96.8 %).

15.1.3. DOSE-RESPONSE EFFECTS

The optimum dose for radiation therapy in childhood low grade glioma has not been well established (Table 31). In children, no prospective randomized studies of radiotherapy dose/response have been performed. Retrospective analyses are rare comprising small patient numbers and very heterogeneous dose prescriptions and the selection of dose prescriptions was strongly influenced by patient age, extent and site of tumor with a tendency to a lower dose in younger children with larger tumors (larger treatment portals).

Although it is difficult to define an adequate dose prescription, the recently recommended and generally accepted dose prescription ranges between 45 and 54 Gy in 1.8 Gy fractions depending on age at treatment, extent of disease and location of tumor.

Table 31 : Progression-free survival in children and adults with low grade glioma / dose - response relationship.

Author	patients	total dose	Fractionated dose	PFS (5 years)	PFS (10 years)	p-value
Karim et al., 1996	171<br>172	45.0 Gy<br>59.4 Gy	1,8 Gy	47%<br>50%	Not reached	p : n.s.
Montgomery et al., 1977	 7<br>9	 </= 42 Gy<br>>/= 50 Gy	n.m.	Overall<br>43%<br>100%	n.m.	n.m.
Sung et al., 1982	 13<br>29	 35 - 45 Gy<br>50 - 60 Gy	n.m.	Relapse rate :<br>11 / 13<br>8 / 29	n.m.	n.m.
Alvord, Jr and Lofton, 1988	52<br>62	> 45.0 Gy<br>< 45.0 Gy	n. m.	80%<br>65%	65%<br>55%	n. m.
Flickinger et al., 1988	12<br>12	> 45.0 Gy<br>< 45.0 Gy	Calculation according nominal standard dose	100%<br>75%		P=0.045
Kovalic et al., 1990	3<br>30	< 40.0 Gy<br>> 40.0 Gy	n. m.	0<br>90%	0%<br>79%	<0.0001
Garcia et al., 1990	8<br>17	< 40 Gy<br>=/> 40 Gy	n.m.	4/8 recurred<br>2/17 recurred	n.m.	n.m.
Jenkin et al., 1993	19<br>15	> 50.0Gy<br>< 50.0 Gy	n. m.	88%<br>72%	88%<br>57%	0.37, n.s.
Grabenbauer et al., 2000b	9<br>16	44 – 45 Gy<br>45.1 - 60 Gy	1.6 – 2.0 Gy	87%<br>90%	36%<br>85%	0.04

n.s. : not significant, n.m. : not mentioned, PFS : progression – free survival

15.1.4. TUMOR VOLUME RESPONSE TO RADIATION

Radiologically determined response of low grade gliomas to radiotherapy has not been well documented because it has been assumed that they are indolent and unresponsive to radiotherapy. The typical biological behavior of a delayed tumor regression assessed clinically and by imaging investigations has often been disregarded. It can be suggested that low grade gliomas in children can demonstrate shrinkage on radiographic studies in response to radiotherapy, but that such shrinkage is not directly related to tumor control or improvement of symptoms.

Table 32: Response assessment following radiotherapy of residual tumor:

Author	N/type of tumor	Dose of RT	Results
Gould et al 1987	20 Optic glioma		10 regression<br>9 SD<br>1 PD
Furuya et al 1986	1 Chiasmatic glioma	RT 51,4 Gy	Regression over 2,5 years
Bataini et al 1991	3/57		3 CR at 6 months after RT
Grabenbauer et al, 2000b	6/25	RT 44-60 Gy	Regression of<br>≥ 50 % after 6 to 24 months

Fisher et al, 1998	19/80 low grade glioma		10 tumor volume reduction<br>5/10 response at 1st follow-up scan<br>median time to response 3,3 months<br>CR: 4 (21%) at 7, 12, 15m, 5y<br>≥ 50 %: 5 (26%)<br>≥ 25 %: 8 (43%)≥
Tao et al, 1997			Great variability in time to response<br>56 % SD over the whole period of follow-up;<br>24 % PR median time to maximal<br>16 % CR response: 62 months, maximum > 10 years.

A great variability in time to (maximal) response was observed. Response to radiation can be very slow taking years in some cases and is therefore not necessarily detectable on the first follow up scan. Many patients continue to display visible residual tumor on imaging many years after therapy. Treatment related changes on MRI imaging might be misleading and should be distinguished from tumor progression. Bakardjiev et al. followed patients with MR imaging at close time intervals between 3 and 26 months after stereotactic fractionated radiotherapy with a total dose between 52.2 and 60 Gy [Bakardjiev et al., 1996]. Twelve of 28 patients developed an increased size of the lesions between 9 and 12 months after radiotherapy which was not accompanied by clinical symptoms. The changes resolved or decreased by 15 to 21 months.

Table 33 : Stereotactic fractionated and proton therapy in childhood low grade gliomas (hemispheric and midline location).

Author	Technique	Patients	Outcome	Follow-up
Dunbar et al., 1994	fractionated convergence therapy<br>(5x 1.8 –2.0 Gy / 45 –54 Gy)<br>+ dose escalation 60 Gy	11<br>(initial RT)<br>9<br>(recurrence)	No acute side effects<br>1 CR<br>19 PR / SD<br>Overall survival 100%	16 months
Bakardjiev et al., 1996	fractionated convergence therapy<br>(5x 1.8 –2.0 Gy<br>/ 52.2 – 60.0 Gy)	28	Overall survival 100%<br>15 pat decrease of tumor size<br>1 pat. Stable tumor size<br>13 pat. Increased tumor size (transient (15-21 months))	24 months
Benk et al., 1999	Hypofractionated convergence therapy<br>(median total dose 39 Gy – 18.0 – 42.0 Gy- in 6 – 10 fractions)	8	1 edema, 1 edema + tumor necrosis, 1 tumor necrosis<br>5 year progression-free survival 60%<br>0verall survival : 100%	42 months
Debus et al., 1999	Fractionated conformal radiotherapy<br>Median total dose<br>52.4 Gy / 1.6-2.0 Gy fractionated dose	10	Progression-free survival at 5 years 90%, overall survival 100%<br>No acute toxicity	12-72 months
Merchant et al., 2002 [	Fractionated conformal radiotherapy<br>Median total dose<br>54 – 59.4 Gy / 1.8 Gy fractionated dose	38	4 failures<br>(3 within CTV and one immediate outside)	17 months<br>(3-44 months)
Hug et al., 2002	Proton therapy<br>50.4 – 63.0 CGE<br>(Cobalt Gray Equivalent), 1.8	Total 27 pat.<br>Hemispheric<br>7 pat.	Local control survival rate<br>Hemispheric	3.3 years<br>(0.6-6.8 y.)

	Gy fractionated dose	Dienceph.<br>15 pat.<br>Brainstem<br>5 pat.	71%<br>Dienceph.<br>87%<br>Brainstem<br>60%	86 %<br><br>93%<br><br>60%	

15.1.5. TREATMENT FIELDS

Advances in neuroimaging enabled new approaches in the management of childhood low grade glioma relating to diagnosis, decision on surgery and treatment planning for radiotherapy as well as assessing response to therapy or for follow-up. An advantage of contemporary (CT/MR-era) over earlier (pre-CT/MR era) seems to lie in better delineation of the tumor site/size. This has led, at least in part, to a significant improvement in survival of adults treated in the CT-era probably due to fewer marginal misses [Kortmann et al., 2000a]. Especially in pilocytic astrocytomas a sharply demarcated contrast enhancing lesion is often seen on imaging. These tumors only rarely infiltrate normal surrounding tissue and it can be anticipated that macroscopic tumor is precisely delineated. Since 60-70% of all low grade gliomas may be non-enhancing on CT it is to be expected that MRI would lead to better and earlier diagnosis, and may also be used for treatment planning [Kortmann et al., 2000a]. Computer assisted (preferable 3D) treatment planning is mandatory because it will reduce possible acute morbidity and late sequelae by reducing the volume of normal tissue exposed to a high RT dose. Whenever feasible image fusion of diagnostic MR and CT scans should be used to determine the target volume. Conformal treatment techniques will also help further reduce irradiation of normal tissue.

Although it has been shown using stereotactic biopsies that tumor cells can extend beyond imaging abnormalities which may suggest wider radiotherapy treatment fields, data from adult patients accumulated over decades support the use of localized fields to treat low grade gliomas [Kortmann et al., 2000a]. In childhood low grade glioma local failure is the predominant feature in progressive or recurrent disease and leptomeningeal spread is a rare event (less than 5%) [Pollack et al., 1995;Pollack et al., 1994]. This implies that treatment fields encompassing the tumor are appropriate in contrast to large lateral opposed fields predominantly used in the pre-CT area. Safety margins for the clinical and planning target volume should be defined according to anatomic borders and the reproducibility of field alignment. It is not necessary to encompass large zone of possible infiltration like in high grade glioma. With the identification of isolated tumor cells beyond the margin of a tumor on a T2 weighted MR image, the appropriate clinical target volume should include the MRI indicated extent of the tumor with a close margin of surrounding brain tissue with respect to anatomical boundaries. Debus et al. (1999) used three-dimensional conformal external beam radiotherapy to treat 10 patients. The clinical target volume included the visible tumor in CT and MRI plus 5 mm, the planning target volume consisted of the clinical target volume plus 2 mm safety margin. With these restricted treatment volumes the median target volume was 14.7 cm^3. No treatment failure was observed suggesting that limiting the high dose volume did not cause an increase in marginal or out-of-field failure rate. Merchant et al. (2002b) concluded that normal tissue sparing through the use of advanced radiation therapy treatment planning and delivery techniques should be beneficial to pediatric patients, if the rate and patterns of failure are similar to conventional techniques at a longer follow-up.
The currently recommended standardized approach is based on the ICRU 50 / 62 report. The clinical target volume (CTV) encompasses the visible tumor as seen on MR (T2 weighted images) with an additional margin of 0.5 cm. If surgery was performed, postoperative delineation of residual disease will be used for treatment planning. The preoperative scans are

used to identify regions of possible tumor infiltration. It is not necessary to entirely encompass areas of cerebral edema. The planning target volume (PTV) encompasses the CTV with an additional margin according to the precision of treatment technique (0.2 - 0.5 cm if rigid head fixation and 0.5 - 1.0 cm if a conventional face masks/head shell is used) depending on the departments policy [Kortmann et al., 1994;Kortmann et al., 1999] (Table 34).

Table 34*:* Geometric precision of current treatment techniques in irradiation of primary tumor site

**Author**	**Technique**	**Fixation system**	**Precision (linear Deviations -mm-)**
Kortmann et al., 1994	conv. 2-D therapy	thermoplastic face mask	2,5mm / max. 5mm
Warrington et al., 1994	fractionated convergence therapy	Gill-Thomas-Cosman Ring	1mm / max 2,3mm
Kortmann et al., 1999	Conformal radiotherapy	rigid face mask (cast)	0,9mm /max.3,0mm

15.1.6. MONITORING OF INTEGRAL DOSE TO TUMOR AND ORAGANS AT RISK

Radiation induced growth hormone deficiencies seems to depend on a dose / volume relationship and the corresponding integral dose distribution. Adan et al. investigated growth hormone (GH) deficiency caused by cranial irradiation during childhood in cohorts of 18, 24, 30 to 40 and 45 to 60 Gy (optic glioma). Growth hormone levels were significantly lower after 18 to 40 Gy (whole brain irradiation) as compared to 45 to 60 Gy (limited volume irradiation) [Adan et al., 2001]. Decrease correlated with dose but not with age at treatment. The relationship between irradiated volume and dose prescription is both a difficult and important issue when attempting to reduce the risk for radiation induced endocrinopathies. Merchant et al. addressed this question in an analysis on growth hormone deficiency in 25 children with primary brain tumors requiring local treatment fields only [Merchant et al., 2002a]. The baseline was normal in all patients. Peak GH levels were modeled as a function of time after radiotherapy and volume of the hypothalamus receiving a dose within the specified intervals of 0-20 Gy, 20-40 Gy, and 40-60 Gy. GH deficiency was observed in 11 children at 6 months and a total of 20 children at 12 months. The effects appeared to depend on hypothalamic dose-volume relationship and may be predicted on the basis of a linear model that sums the effects of the entire distribution of dose. These calculations may in future allow to predict or reduce the risk for endocrine disorders.
MR imaging is a new method and measures tissue spin-lattice relaxation time (T1) with respect to spatial distribution of structural changes. It is sensitive to subtle changes below the resolution of conventional MR imaging. The working group of St. Jude Hospital assessed the effect of ionising radiation to the brain in 29 pediatric patients undergoing fractionated conformal radiotherapy of brain tumors [Steen et al., 2001]**.** Mapping showed that white matter exposed to less than 20 Gy and gray matter to less than 60 Gy does not undergo pathologic changes. The results indicate that conformal techniques, although delivering dose over a larger area of the brain offers a substantial benefit for children.

15.1.7. LOW GRADE GLIOMA OF THE SPINAL CORD

Low grade astrocytomas of the spinal canal are rare, accounting for less than 10 % of spinal cord tumors. They predominantly arise in the intramedullary region and exhibit a typical growth pattern often spanning many vertebral segments resulting in them having an apparent 'pencil shape'. Because of the lack of prospective trials with sufficient follow-up treatment strategies are based on those for intracranial tumors on the assumption that the pathobiologic behavior is comparable. With the ongoing advances in imaging, surgical skills and radiation techniques it becomes difficult to assess the value of each therapeutic intervention. Tumor extension often precludes complete tumor removal, and thus the role of radiotherapy has to be defined with respect to preservation or improvement of neurological function, site and extent of disease, surgical resectability, age and recently chemotherapy.
Prognostic factors are difficult to define. Abdel-Wahab et al. found in a multivariate analysis that involvement of more than five segments of the vertebral column was associated with a significantly inferior outcome [Abdel-Wahab et al., 1999]. Minehan et al. noted in his series comprising 79 children and adult patients that patients with pilocytic astrocytoma fare significantly better than those with diffuse fibrillary astrocytoma, WHO grade II [Minehan et al., 1995]**.** For all patients, the 5 and 10 year survival rates were 55 % and 50 %, respectively. In pilocytic astrocytoma a 5 and 10 year survival rate of 80 % could be achieved as compared to 15 % in fibrillary astrocytoma. There was a trend towards a better survival rate in patients receiving radiotherapy for pilocytic astrocytoma (85 % versus 75 % after surgery alone) and a significant advantage for non pilocytic astrocytoma. The extent of tumor resection did not reveal an impact on survival. However, a more aggressive surgical approach was associated with a poorer outcome as compared to biopsy only [Minehan et al., 1995]. The most favorable outcome was observed by O'Sullivan et al. in 12 patients younger than 17 years suggesting a better prognosis for children [O'Sullivan et al., 1994]. Independent of the extent of surgical resection the 10 and 20 year progression – free and overall survival rates were 83 % and 71 %, respectively. In this analysis, however, the histological subtypes were not clearly stated and the contribution of pilocytic astrocytoma which are associated with a survival advantage as it was demonstrated in the series of Minehan et al., is unknown. In the series of the Princess Margaret Hospital comprising adult and pediatric patients postoperative radiotherapy achieved a 5-year overall, cause-specific, and progression-free survival rates of 54 %, 62 %, and 58 %, respectively [Rodrigues et al., 2000]. Factors predicting improved outcome on univariate analysis were age < 18 years, low grade histology, and length of symptoms prior to diagnosis > 6 months. Bouffet et al. retrospectively analysed 49 consecutive patients with spinal cord astrocytoma [Bouffet et al., 1998]. Twenty-one patients received radiation therapy and achieved a 10 year survival rate of 83 % as compared to 70 % after surgery alone (21 patients) indicating a possible advantage of postoperative radiotherapy. However, the criteria for selecting treatment modalities was not clear in the report.
Control of neurological deficits is a major option for the selection of treatment but due to the paucity of data in the literature the impact of radiotherapy on neurological function is difficult to estimate. In a retrospective analysis of Jyothirmayi et al. 23 patients who received radiotherapy were followed for a mean of 51 months. Partial excision was achieved in 10 patients and surgery was limited to biopsy in 10 patients [Jyothirmayi et al., 1997]**.** At six months after radiotherapy 12 patients had improvement of neurological deficits, 9 had stable disease status and only 2 had deteriorated indicating a benefit of radiotherapy.

15.1.7.1. Treatment volume / dose prescriptions

In the majority of cases spinal low grade glioma recur locally and metastatic spread is a rare event. In all published series radiotherapy to the tumor site was performed. Chun et al. and Linstadt et al. assessed the pattern of relapse and observed no CSF seeding or relapse outside the treatment portals [Chun et al., 1990;Linstadt et al., 1989]**.** With the use of MR imaging the

gross tumor volume according to the ICRU – 50/62 report can be accurately delineated and a safety margin in cranio-caudal direction of one vertebral body is recommended in the literature [Chun et al., 1990;Linstadt et al., 1989]. Although difficult to assess because of small patient numbers and a presumed shallow dose – response curve it appears that doses in excess of 45 Gy are sufficient for tumor control [Linstadt et al., 1989]. Doses less than 40 Gy may be associated with an increased failure rate [Chun et al., 1990]. Two of three patients died of locally recurrent disease after doses between 20 and 38 Gy. Also, beyond 50 Gy no additional benefit in terms of progression – free survival was observed by Minehan [Minehan et al., 1995]. With respect to the presumed dose - response relationship of their intracranial counterparts doses between 45 and 54 Gy are currently recommended.

15.1.8. CONCLUSION

Current knowledge about the use and effect of radiotherapy in childhood low grade glioma results from small series, in which indication to therapy, doses and fields were highly variable. Nevertheless the results allow to define guidelines for its employment. But improvement of treatment techniques allow to spare normal tissue more consequently. Thus it shall be investigated, if these advances translate into a benefit for the patients. The aims can only gain clinical importance if the follow-up will be closely monitored in terms of assessment of quality of survival.

15.2. Aims of the Radiotherapy protocol

The following aims will be addressed in the protocol.

- To utilize modern treatment techniques to reduce the integral radiation dose given to normal tissue compared with the previous protocol.
- To record and monitor the integral dose to tumor and normal tissue as a basis for future assessment of quality of life of long term survivors
- To assess response of tumor and clinical symptoms to radiotherapy (intracranial and spinal tumors) with respect to primary treatment or after chemotherapy has failed.
- To assess the pattern of relapse, when using modern treatment techniques
- To assess the efficacy of cranio-spinal irradiation in metastatic disease
- To assess clinical outcome after brachytherapy
- To assess efficacy of brachytherapy

15.2.1. Rationale to maintain dose perscription

RT is an effective treatment for LGG in children. In the previous SIOP trial the irradiation of the tumor site in case of progressive disease revealed response rates in excess of 90% on imaging at a dose of 50.4 to 54.0 Gy at a median follow-up of 48 months. In view of these high response rates it seems to be justified to attempt to modify treatment, aiming to reduce acute side effects and late sequelae of treatment. Data on dose response effects are conflicting. In children they are essentially based on heterogeneous patient cohorts with small numbers. A lower dose has often been used in larger tumors and younger children. Although the data for adults might be promising (in the prospective, randomized EORTC study no difference was seen between 45.0 and 59.6 Gy in terms of survival). Data in children suggest, that a dose level of 54 Gy appears to be more effective than lower dose prescriptions [Karim et al., 1996;Horwich and Bloom, 1985]. Taking into consideration potentially hazardous effects on the developing central nervous system, it appears to be more important to reduce the dose to normal tissue rather than to lower the dose to tumor.

15.2.2. Rationale to introduce modern treatment techniques

Data on long term effects caused by radiotherapy are based on patient series who were treated in the sixties to the seventies in the majority of cases. Precise delineation of tumor was not possible and treatment techniques available then mainly comprised large portals given as an isocentric oposed fields. It could not be avoided to irradiate large areas of normal tissue. Additionally, high single doses were often used [Chadderton et al., 1995]. The development of modern imaging and treatment techniques in radiotherapy (“stereotactic radiotherapy”) opened the approach to effectively conform the dose to tumor while sparing normal surrounding tissue. Today stereotactic facilities are widely spread and allow an application of stereotactic radiotherapy in all children, who will go on to radiotherapy according to the entry

criteria. Although some experience has been acquired for stereotactic radiosurgery given with a high single or hypofractionated schedule, the conventionally fractionated approach is more convincing because the previously performed dose prescription can be continued and larger tumors can be treated better. The new techniques are able to reduce the integral dose to normal tissue. Consequently, it s indispensable to record and monitor the integral dose to tumor and normal tissue to obtain information as to what extent the dose to normal tissue can be reduced and as to whether the dose reduction will be reflected by an acceptable acute and long term toxicity.
Exception : Interstitial radiotherapy (Brachytherapy) can also be applied in selected cases. Patients treated with this technique will undergo a separate surveillance.

15.2.3. Rationale for monitoring of integral dose to tumor and organs at risk

Radiation induced endocrine disorders and structural changes of brain parenchyma seem to depend on a dose / volume relationship and the corresponding integral dose distribution. These calculations may allow to predict such late effects and appropriate selection of adequate plans will help to reduce the risk for their development.
IMRI and Protontherapy are also allowed (see documentation forms)

15.2.4. Rationale to monitor tumor response to radiotherapy

Since the response of tumor size and clinical symptoms to radiotherapy are known only in very few patients (see 15.1.5.), it is therefore important to obtain detailed information about the natural course of disease after end of treatment and to assess the impact for subsequent supportive care.
Increase in size after end of treatment seems to be not an uncommon effect and it appears that an increase in size is not accompanied by clinical signs and symptoms. However, increase in size might nevertheless be misleading and misinterpreted as recurrent disease.

15.2.5. Rationale to perform cranio – spinal irradiation in metastatic disease

Although reports on the efficacy and feasibility of cranio-spinal irradiation are scarce in the literature and this therapy has been given in very different settings and with varying dose prescriptions, there are convincing data that a positive effect can be expected. It is therefore necessary to assess acute toxicity and progression-free and overall survival as well as long-term toxicity prospectively with a definite dose prescription.

15.2.6. Rationale for brachytherapy

The role of brachytherapy has until now only be retrospectively investigated in single institutions often including adult patients. Data for children in larger cohorts are lacking. It is therefore intended to prospectively investigate the role of brachytherapy in the management of low grade glioma and to obtain information in terms of tumor control and side effects. The choice for brachytherapy is not depending on the eligibility criteria within the chemotherapy and radiotherapy study. The therapeutic decision will be made at the discretion of the participating institution. Biometric evaluation, however will be subject to the statistical analysis described in section 17. In case of progressive disease after brachytherapy the decision on subsequent treatment (fractionated, external radiotherapy or chemotherapy according to this protocol) should be made after contact with the national coordinating center.

15.3. Endpoints of trial

Primary endpoint
is the assessment of progression-free survival

Secondary end-points are :

- Overall survival
- Assessment of integral dose to tumor and normal tissue and evaluation on the impact on long – term toxicity
- Assessment of tumor response to radiotherapy by imaging and clinical investigations
- Assessment of progression-free and overall survival, acute and long-term toxicity of cranio – spinal irradiation in metastatic disease.

15.4. Eligibility criteria for Radiotherapy

1. Eligibility criteria for this study are listed in section 9., the indications to start non-surgical therapy are detailed in section 10. The indications to start radiotherapy are identical with the criteria to start chemotherapy respecting the age-related strategy.
Detailed information upon treatment strategies for the therapy groups 1 to 3 is given in section 12.

2. All children with the age of eight years or older with a histologically proven low grade glioma of intracranial and spinal sites, fulfilling the criteria for the start of non-surgical therapy, and for whom patients/parents and physician decide to give radiotherapy as non-surgical therapy, will be included. Diagnoses made by imaging is also allowed for chiasmatic-hypothalamic tumors, provided, that imaging, clinical course of disease and tumor location make the diagnosis of a low grade glioma most probable (section 8.5.).

3. Children younger than eight will also be included upon individual indication, e.g. if (successive) chemotherapies have failed and the children reveal signs of progressive disease clinically or on imaging.

4. Children with disseminated disease may be irradiated upon individual indication. These cases should be discussed with the national study coordinators.

5. Exception : brachytherapy
Indication for treatment with brachytherapy is at the discretion of participating institution irrespective of the indications for treatment as defined in section 10. However the specific limitations of brachytherapy as described in paragraph 15.1.1.4. should be observed.

15.5. Specific and technical outlines for Radiotherapy SIOP LGG 2004

15.5.1. Pretherapeutic imaging

In order to assess the precise extent of tumor growth MR scanning including contrast enhanced T1 and T2 weighted imaging is necessary. Areas of blood brain barrier disruptions should be recorded and monitored during follow-up, as these areas might indicate malignant transformation. For treatment planning preoperative and postoperative imaging is necessary. For spinal tumors MR imaging pre- and postoperatively is indispensable to delineate extent of disease.

15.5.2. Treatment technique / intracranial and spinal sites

15.5.2.1. Intracranial sites :

When aiming to reduce possible acute morbidity and late sequelae it is necessary to reduce the volume of normal tissue exposed to a high RT dose. Computer assisted treatment planning is therefore mandatory. Three dimensional treatment planning should be used if possible. Conformal treatment techniques will help to further reduce irradiation of normal tissue. In addition, the dose to critical organs must be recorded (see documentation sheets). Whenever feasible image fusion of diagnostic MRI and CT-scans should be used to determine the target volume.

15.5.2.2. Spinal sites

Computer assisted treatment planning should be used in order to obtain a reproducible dose distribution.

15.5.3. Target volumes

Target volumes will be defined according to the ICRU 50/62. The clinical target volume (CTV) encompasses the visible tumor as seen on MR (T2 weighted images) with an additional margin of 0.5 cm. If surgery was performed, postoperative delineation of residual disease will be used for treatment planning. The preoperative scans are used to identify regions of possible tumor infiltration. It is not necessary to entirely encompass areas of cerebral edema. The planning target volume (PTV) encompasses the CTV with an additional margin according to the precision of treatment technique (0.2 - 0.5 cm if rigid head fixation and 0.5 - 1.0 cm if a conventional face masks/head shell is used) depending on the departments policy (Kortmann et al., 1994, 1999). When defining the clinical target volume anatomical borders must be considered.
For spinal sites the safety margins to visible tumor in cranio-caudal direction should be the lenght of one vertebral body. It is not necessary to entirely encompass a syrinx if present or the entire zone of edema. Postoperative imaging should be used in case of surgical resection. Laterally the filed border should encompass the pedicles.

15.5.4. Dose specification

RT dose is specified according to the ICRU 50/62 report. The ICRU reference point by definition is located in the center of the target volume (100 %). Dose inhomogeneity within the target volume should not exceed the tolerance limits of 95 % and 107 %.

For spinal sites dose specification should be located at the dorsal border of the vertebrae.

15.5.5. Dose prescription

For cranial sites a total dose of 54.0 Gy should be administered in a fractionated dose of 1.8 Gy, 5 times per week. All fields should be treated daily. For spinal sites the dose is limited to 50.4 Gy.

Table 35: Dose prescription for radiotherapy of low grade glioma

Target volume	Number of fractions	Dose per fraction	Total dose	Duration (weeks)
Intracranial tumor site	30	1.8Gy	54.0Gy	6
Spinal tumor site	28	1.8	50.4 Gy	5 ½

In case children under 5 years shall be irradiated, the national radiotherapy coordinator should be contacted. Doses should be limited to 45,0 Gy at 1,8 Gy per fraction.

15.5.6. Patient positioning

It is recommended that an individualized face mask (head shell) is used to guarantee the reproducibility of head positioning. If possible a rigid head fixation should be used to reduce the planning target volume (Kortmann et al., 1999).

15.5.7. Cranio-spinal irradiation (CS-RT)

Planning CT is strongly recommended for definition of the target volume for the cranio-spinal axis, posterior fossa and tumor bed volumes. It is recommended that the CT slice thickness should be no greater than 0.5 cm in the region of the cribriform fossa, base of skull, posterior fossa and cranio-cervical field junction, and no greater than 1.0 cm elsewhere within the cranio-spinal axis. TVs and OAR shall be outlined:

Target Volumes (TVs)	Organs At Risk (OAR)
Craniospinal axis<br>Metastatic deposits	Eyes<br>Pituitary<br>Inner ear<br>Hypothalamus<br>Optic chiasm

Dose Volume Histograms (DVHs), if available should be constructed for the planning target volumes (PTVs) and OAR.
If the spinal field is treated with electron beams the dose along the entire spinal axis should be calculated with an appropriate correction for tissue heterogeneity.
If CT planning is not available then conventional planning of the target volumes is acceptable.
Planning CT exam is strongly recommended, particularly for the posterior fossa and tumor bed target volumes.

15.5.7.1. Three-dimensional planning

It is strongly recommended that 3-D planning should be used to determine the target volume

for metastatic deposits. Some centres may wish to consider 3-D planning for determination of CS-RT target volume.

15.5.7.2. Treatment volume anatomical description and dose

Craniospinal Axis:
The clinical target volume (CTV) for CS-RT comprises the whole brain as well as the spinal cord and thecal sac.

Whole Brain Volume
The whole brain CTV should extend anteriorly to include the entire frontal lobe and cribriform plate region. The superior orbital tissue should be included in the treatment volume, but not the posterior globe. The treatment volume should extend at least 0.5 cm inferiorly below the cribriform plate and at least 1 cm elsewhere below the base of the skull (paying particular attention to the margin around the inferior aspect of the temporal lobes). The margin between the shielding and the anterior border of the upper cervical vertebrae should be 0.5 cm. The lower border of the cranial fields should form a precise match with the upper border of the spinal field.

Cervical Spinal Volume
As much as possible of the cervical spinal volume is included in the lateral cranial fields with the junction between the cranial and spinal fields kept as inferior as possible. This is advised for two reasons:
Avoidance of as much thyroid tissue irradiation as possible, by shielding this within the cranial volume.
To minimise the risk of the junction being close to the primary tumor and thus the risk of a ‘cold spot’ in this region the spinal field should extend superiorly to form an accurate match with the lower borders of the cranial fields.

Dorso-Lumbar Spine Volume
The inferior limit of the spinal CTV must be determined by imaging the lower limit of the thecal sac on a spinal MR scan and will usually extend inferiorly to at least the lower border of the second sacral vertebra.

Width of the Spinal Volume
The aim is to include the entire subarachnoid space including the extensions along the nerve roots as far as the intervertebral foramina. The spinal CTV should extend laterally to cover the intervertebral foramina with at least 1 cm margin on either side. The use of a ‘spade’ shaped field to treat the lumbo-sacral spine is not recommended.

Metastatic deposits
It is strongly recommended that the CTV for metastatic deposits should be determined on a planning CT. For PTV, an additional margin should be allowed according to departmental policy. This will generally be a margin of 0.5 cm. The field arrangement will be chosen to provide a high conformity index, avoiding OAR where possible.

15.5.7.3. Dose Specification

Dose Definition **:** All doses will be specified according to ICRU 50/ICRU 62.
Reference Point:

Brain
If the brain is treated by a pair of parallel opposed fields, the dose should be defined at the midpoint of the central axis.

Spine
The dose to the spine should be prescribed along the central axis at a depth representing the posterior margin of the vertebral bodies.
In the case of electron RT to the spine the anterior border of the target volume (posterior aspect of the vertebral bodies) must be encompassed within the 85% isodose.

Metastatic deposits
The prescription point should be in the center of the target volume, i.e. at the intersection point of oblique fields or along the central axis of the opposed beams, midway between the two entrance points.

Table 36: Total Treatment Dose

Brain :	35.2 Gy in 22 fractions of 1.60 Gy
Spine :	35.2 Gy in 22 fractions of 1.60 Gy
Metastatic deposits : (Intracranial sites)	55.0 Gy cumulative dose , 1.8 Gy fractionated dose
Metastatic deposits : (spinal sites)	49.6 Gy cumulative dose , 1.8 Gy fractionated dose
Dose restriction (maximal cumulative dose) > 50% intracranial volume and or > 2/3 of spinal canal : 45 Gy	

15.5.8. Documentation

It is mandatory to document the field alignment using simulator films and polaroid photographs. At the start of radiotherapy verification films should be obtained of each irradiated field.
Portal films should be repeated once a week. Precise application of radiotherapy is essential for both tumor control and reduction of side-effects.
To develop recommendations for optimal treatment techniques it is necessary to analyze the radiation protocols, the prescription of target volumes, doses and the accuracy of treatment delivery. Therefore, it is requested that the following data (copies) be sent to the reference center for radiotherapy.

-Radiation protocols
-Simulation films
-Portal films
-Computer assisted treatment plans
-Polaroid pictures of patient positioning and field allignment
-Evaluation forms (Addendum 21.9.) Patient data
Toxicity
Treatment technique / dose

15.5.9. Acute treatment related toxicity

Steroid prophylaxis of cerebral edema is not mandatory during radiotherapy. If cerebral oedema occurs dexamethasone should be given orally or iv, if necessary.
The acute maximal toxicity during irradiation should be documented on the evaluation forms.

15.5.10. Routine laboratory tests during radiotherapy

- Red and white blood cell counts, platelet counts: 2x weekly in CXA, 1x weekly in limited volume radiotherapy..
- If the patient is receiving steroid medication: blood glucose 1x weekly.
- Before and at the end of radiotherapy: sodium, potassium, calcium, GOT, GPT, Gamma-GT, LDH, creatinine, BUN, hormones of the pituitary axis (TSH, growth hormone, ACTH, FSH/LH - see endocrine guidelines section 8.4.).

16. Definitions for: Tumor staging Extent of resection Response and remission Serious adverse event
SIOP LGG 2004

16.1. Tumor staging

Primary solitary tumors and disseminated tumors

No validated **staging system** exists for childhood LGG. Thus, the assessment of tumor extension will be based on descriptive terms aiming to define:

- tumor site: Main region of the brain:
  1. cerebral hemispheres
  2. supratentorial midline
  3. cerebellum
  4. caudal brain stem
  5. spine

- structures involved – Definition of local extension (supplementing the main tumor site)
  - ad 1. exact hemispheric lobes,
  - ad 2. visual pathways: right and/or left optic nerve; chiasma; hypothalamus; right and/or left (posterior) optic tracts; basal ganglia, thalamus or other midline structures, midbrain
  - ad 3. cerebellar hemispheres, vermis, cerebellar-pontine angle
  - ad 4. upper/middle/lower pons, medulla
  - ad 5. region and number of spinal segments involved

- tumor "volume":
  2 diameter surface area calculation or
  a third diameter creating an ellipsoid, giving an indication of volume
  (horizontal x vertical x sagittal x 0,5)

The relevance of "multi"-dimensional tumor volume assessment has been reviewed for tumors outside the central nervous system especially with respect to response assessment (Therasse et al., 2000). Within this study tumor "volume" should preferably be recorded by three dimensions, but to document two dimensions is the minimum requirement. For a given patient the documentation of tumor "volume" should always apply the same diameters in comparable MRI/CT planes (section 8.5.).

- evidence of leptomeningeal and/or sub-ependymal tumor dissemination
  1. number of lesions
  2. localisation within brain and spine
  3. morphologic description and size of multifocal tumor

<u>Classification of leptomeningeal dissemination:</u>

In an attempt to classify meningeal dissemination the classification of Chang (Harisiadis and Chang 1977) will be adopted. It will be investigated whether this staging system is appropriate for low grade glioma:

M 0: no dissemination

M 1: positive proof of tumor cells in the lumbar cerebro-spinal fluid, more than 14 days following an operative intervention, but no concurrent meningeal enhancement on MRI or CT. If possible, immunohistochemical staining (GFAP) should be performed

M 2: meningeal dissemination in the cerebral area in form of
a. laminary thickening
b. nodular deposits or very thick laminary layers

M 3: meningeal dissemination in the spinal canal in form of
a. laminary thickening
b. nodular deposits or very thick laminary layers

M 4: extraneural metastases (related to shunt or not)

16.2. Extent of resection

The minimal modified criteria to define extent of resection and response as elaborated by the Brain Tumor Sub-Committee are adopted for the study (Gnekow 1995).
The classification of the extent of resection should be based upon the results of the surgical report and of the postoperative neuroradiologic assessment, but be primarily a radiological classification aided by the surgeon's report. Four categories have been defined for each field:

<u>Extent of resection – Surgical judgement</u>

S1 - Total resection, no recognizable residues
S2 - Remaining tumor of less than 1,5 cm^3, possible local invasion
S3 - Residual tumor of more than 1,5 cm^3
S4 - Tumor volume unchanged, biopsy

<u>Extent of resection – radiological judgement</u>
(on early (24 to max. 72 h) post-operative MRI or CT without and with contrast enhancement)

R1 – No visible tumor ("Total")
R2 – Rim enhancement at the operation site only ("RIM")
R3 – Residual tumor of a measurable size (product of two/three diameters – "Lump")
R4 – No significant chance to preoperative tumor size ("minimal change")

Extent of resection combining surgical and radiological judgement:

	Radiology	Surgery
**I "Total"**	R1 - Total	S1 - Total
**II Near Total**	R1/R2 - Total or Rim	S2 - Small residue<br>? localised invasion
	R2 - Rim	S1 - Total
**III "Partial"**	R3 - Distinct lump	S1/S2/S3 - Any residual disease
**IV "Biopsy"**	R4 - Minimal change	S4 – Biopsy

A **total resection** can only be stated, when surgical and radiographic judgement agree (S1-R1).

Near total resection - Leaving a small residual of tumor behind, which may be invading, can result in a rim enhancement at radiologic investigation or not be visible (S2 - R1/2, R2 - S1).

Partial Resection - In case the post-surgical scan reveals measurable tumor of any size the surgical estimate may agree or may not (S1/2/3 - R3).

Biopsy - In case only a biopsy is performed, the surgical report and radio-diagnostic finding should be identical (S4 – R4).

Thus the **definitions of the extent of resection** will be as follow

Total resection / near total (subtotal) resection = no visible tumor is left at the time of surgery (according the neurosurgeon' s operative note) and this is confirmed by post-operative contrast enhanced CT or MRI scan performed within 48-72 hours from the operation. The presence of tumor at the margins of the resection specimen will be noted.

Incomplete resection/ partial resection = any residual tumor after surgery which is confirmed by post-operative contrast enhanced CT or MRI scan performed within 48-72 hours from the operation. In this case the extent of tumor removal must be established by comparing the pre- and post-contrast enhanced CT or MRI scan.

Biopsy = when the surgical procedure is done for the sole purpose of establishing the pathological diagnosis. Depending upon the site of the tumor and other relevant individual circumstances, biopsies can be taken during an "open" operation or via stereotactic approaches.

16.3. Response and Remission

16.3.1. General assessment of response

To evaluate tumor response in low grade glioma is a complex endeavor. It involves the objective clinical responses to therapy, measured according to criteria suggested below, as well as tumor size/volume changes, measured by the conventional neuroradiological techniques, which will be carefully monitored during therapy. A descriptive multifactorial system will be adopted to cover the scope of possible combinations of tumor response to therapy. Since up to now no study has followed clinical and ophthalmological findings in

relation to radiological response, there are no data to substantiate the relevance of clinical (ophthalmological and other symptoms) and radiologic response or progression. The following components to measure treatment effects will be monitored:

Clinical findings – in particular body weight changes in children presenting with diencephalic syndrome, and the ophthalmologic parameters will be studied along with any relevant neurological and endocrinological signs. Significant visual deterioration (confirmed at two consecutive exams) must be considered as a clear signal for progression.

Lumbar CSF cytologic findings – At the level of current knowledge, lumbar cytologic CSF findings can not be considered a criteria for judging tumor response; however - if previously positive in case of disseminated LGG – it is recommended to follow this parameter during treatment, including the protein level, absolute cell count and cellular morphology on cytospin preparations.

Neuroradiological findings – Changes in tumor size/volume, especially concerning involvement of adjacent structures (right and/or left optic nerve; chiasm; hypothalamus; right and/or left posterior optic tracts; midbrain; others) and evidence of leptomeningeal and/or sub-ependymal tumor dissemination will be monitored to measure and describe neuroradiological tumor response. Changes of the intensity of post-gadolinium contrast enhancement will be recorded, but not used as a parameter to judge response.

Table 35: Definitions of response with respect to:

	Parameters to be studied	Definition
CLINICAL FINDINGS	body weight changes in children presenting with diencephalic syndrome<br><br>neurological signs	Gain<br>Stable<br>Loss<br><br>Better<br>Stable<br>Worse
OPHTALMOLOGICAL FINDINGS	Visual acuity<br>Visual field	Better<br>Stable<br>Worse
CYTOLOGY	Lumbar CSF: number of tumor cells	Decrease<br>Stable<br>Increase
NEURORADIOLOGICAL INVESTIGATIONS	MRI without and with Gadolinium enhancement for primary tumor and/or multifocal lesions	Tumor size/volume change see definition below

16.3.2. Criteria of neuroradiologic response of primary tumor and of disseminated lesions

Complete response : No radiological evidence of tumor on contrast enhanced CT or MRI scan. Disappearance of multifocal lesions and tumor cells from the CSF in the case of disseminated disease.

Partial response : Reduction of the size of the solid parts of the tumor of more than 50% (product of the two largest perpendicular diameters) radiographically. A calculation according

to the formula axial x coronal x sagittal /2, referring to the largest diameter in every direction will be performed centrally, but is not directly comparable. If the tumor consists of solid and cystic parts they should be evaluated separately. In significantly polycyclic tumors separate representative nodules should be added to one volume to make the calculation as exact as possible.
In disseminated disease, the distant lesions show reduction in size or a stable size and there is no appearance of new tumor lesions or development of malignant cells in the CSF.

Objective response : Reduction in size of unequivocal residual tumor manifestation between 50 and 25% (product of the two largest perpendicular diameters) radiographically referring to last evaluation. A calculation according to the formula axial x coronal x sagittal /2, referring to the largest diameter in every direction will be performed centrally, but is not directly comparable. If the tumor consists of solid and cystic parts they should be evaluated separately. In significantly polycyclic tumors separate representative nodules should be added to one volume to make the calculation as exact as possible.

There is no tumor progression and no appearance of new tumor lesions or development of malignant cells in the CSF.

Stable disease : Reduction of the size of the solid parts of the tumor of less than 25% (product of the two largest perpendicular diameters) radiographically. A calculation according to the formula axial x coronal x sagittal /2, referring to the largest diameter in every direction will be performed centrally, but is not directly comparable. If the tumor consists of solid and cystic parts they should be evaluated separately. Several tumors should be added to one volume to make the calculation to be as exact as possible.
There is no tumor progression of more than 25 % and no appearance of new tumor lesions or development of malignant cells in the CSF.

Tumor progression : Enlargement of the primary of more than 25 % (product of the two largest perpendicular diameters) radiographically or appearance of new tumor manifestations such as new lesions or tumor cells in the CSF. A calculation according to the formula axial x coronal x sagittal /2, referring to the largest diameter in every direction will be performed centrally, but is not directly comparable.

Complete, partial, objective responses and stable disease will be considered positive responses in this protocol.

16.3.3. Considerations for the neuroradiological assessment of response
Caution: Please be aware that:

♦ For the neuroradiological evaluation of tumor response the contrast behavior will not be taken into consideration, although a reduction in contrast uptake can often be seen following chemotherapy. Contrast behavior of a tumor is very much dependent upon the performance of imaging (dosage of contrast medium, time course after the application and field strength of the magnet) and the relevance of enhancement for progression or regression in low grade glioma is not defined, especially not for grade I astrocytomas.

♦ Pilocytic astrocytoma can have a solid and cystic component of the tumor. Sometimes

only the cystic components enlarge while the solid ones remain unchanged. The isolated enlargement of the cysts is not a secure evidence of tumor progression. It should not be considered for response, because the dynamics of cystic parts do not relate to the proliferative behavior of the tumor, even though the mass effect and the indication for its treatment might immediately be influenced by the cysts.

However, changes of cystic parts as well as contrast behavior should be registered on the evaluation forms/status forms (Addendum 21.8.4. response assessment, 21.13.1. patient status report) to increase information on the dynamics of tumor behavior during or after treatment.

♦ A moderate increment of the tumor dimension can be observed during the first weeks of therapy and more specifically between week 11-12. It is strongly recommended to await the definite treatment response evaluation performed between weeks 22-24 before deciding on the final response to this initial part of therapy and consequently on the subsequent treatment.

♦ Unequivocal progressive visual function deterioration even in face of an unchanged tumor volume, as determined by contrast enhanced brain studies (CT or MRI), has to be considered as tumor progression.

♦ The development of hydrocephalus in isolation without any other radiological evidence of tumor progression should not be taken necessarily as evidence of tumor progression.

♦ Tumor progression by either clinical, ophthalmological or radiological criteria is an indication to start therapy in a child who is observed (see section 10.) or to change therapy if the child is on chemotherapy or has received radiotherapy.

♦ Care should be taken in case of neurologic deterioration, which may be related to steroid withdrawal, coexisting systemic diseases, unrelated intracranial causes (e.g. sub – dural haematoma), delayed seizures or post-ictal findings.

16.4. Severe adverse events, including second malignant neoplasm.

1. All life-threatening treatment-related complications, i.e. WHO/CTC grade 4 toxicities, of the following categories are regarded as a serious adverse event (SAE):
- Peripheral nervous system
- Central nervous system
- Renal
- Hepatic
- Cardiac
- Skin

Additionally the following conditions are regarded as SAE:
- Permanent, relevant handicap following any other toxicity
- Drug overdose

2. The development of allergy to Carboplatin has to be closely monitored in all patients receiving Carboplatin. If early signs do go unnoticed, life-threatening allergic shock may

manifest. This is regarded as a SAE, yet allergy is monitored separately from all other forms of toxicities.

3. WHO/CTC grade 4 hematologic toxicities have to be expected with the protocol presented here. If they resolve and do not have life-threatening consequences, they are not considered as a life-threatening event in the context of this protocol. They are documented routinely concommittant to regular therapy documentation.

4. Death under treatment will be considered an adverse event regardless of its cause. Death, other than death of disease, within 12 months from the end of treatment will be regarded as adverse event, unless it is proven that there is no relation to therapy (e.g. traffic accident).

5. Any solitary, and histologically distinct, malignant neoplasm occurring after the date of diagnosis of the initial tumor and not counting disseminated low grade glioma, is regarded as a secondary malignant neoplasm (SMN). This designation bears no implication for the possible causal mechanism, which especially in patients with NF I may be genetic, giving rise to (multiple) metachronous tumors. The development of SMN should be reported as a SAE as well.

Any serious adverse event must be reported immediately to the national data center, i.e. within the next working day, and followed-up by the treating institution, regardless of whether or not it falls within the categories listed above. The information must be forwarded to the international data center and be relayed to the other national data centers for further reporting according to GCP guidelines.
The documentation form from addendum 21.12. shall be used for the reporting of serious adverse events. Any additional important information should be included as copy.

17. Statistics SIOP LGG 2004

17.1. Chemotherapy Group

17.1.1. Low grade glioma of all sites in children not affected by Neurofibromatosis NF I (group 1 and 2 according to section 12.)

Design of the trial

The aim of the trial is to compare **standard induction therapy** with Vincristin and Carboplatin with the **intensified induction therapy** with Vincristin, Carboplatin and Etoposide in children, who are not affected by Neurofibromatosis (type NF I), with low grade glioma of all sites necessitating chemotherapy as non-surgical therapy (according to patient eligibility criteria (section 9.) and indication for non-surgical therapy (section 10.)).

This therapy optimization trial is multinational, multicenter, non-blinded, randomized and prospective.

The accrual period of the trial is 6 years followed by an observation period of 2 years.

Immediately upon the decision for chemotherapy as non-surgical intervention each child will be randomized to one of the two induction regimens.

For this multinational, multicenter trial randomization will be provided by the Istituto Oncologico Veneto, Clinical Trials & Biostatistic Unit, "SIOP-LGG 2004", University Hospital of Padova, I-35128 Padova, Italy, by using blocks.
Randomisation will be stratified according to age (< 1 year, 1-8 years, ≥ 8 years) and primary tumor site (chiasmatic tumors (Dodge II and III), all other supratentorial midline tumors, tumors of all other sites outside the supratentorial midline).

End points

According to the different questions the following end points are defined:
For definition of progression and relapse referral is made to protocol section 16.3.

1. PFS_R: Progression free survival measured from the time of randomization: Time from randomization up to an event:
   Definition of event:
   - death (for all reasons)
   - progression of a residual tumor (section 16.3.)
   - relapse following previous complete remission (section 16.3.)
   - appearance of new or progression of existing metastasis (section 16.3.)

2. Radiological response measured at week 24: Complete, partial, objective responses and stable disease will be considered positive responses in this protocol. Response definitions according to section 16.3. are used.

3. PFS_D: Progression free survival measured from the time of diagnosis: Time from diagnosis up to an event (definitions of event see 1.).

4. $\underline{EFS_R}$: Event free survival measured from the time of randomization: Time from randomization up to an event.
   Definition of event:
   - death (for all reasons)
   - progression of a residual tumor (section 16.3.)
   - relapse following previous complete remission (section 16.3.)
   - appearance of new or progression of existing metastasis (section 16.3.)
   - severe adverse event / toxicity (not counting Carboplatin hypersensitivity and toxicity of regular protocol application) (section 16.4.)
   - appearance of secondary malignant neoplasm (section 16.4.)

5. $\underline{EFS_D}$: Event free survival measured from the time of diagnosis: Time from diagnosis up to an event. (definition of event: see 4.).

6. $\underline{OS_R}$: Overall survival measured from the time of randomization: Interval starting with the day of randomization and ending with the death of the patient independently of its cause.

7. $\underline{OS_D}$: Overall survival measured from the time of diagnosis: Interval starting with the day of diagnosis and ending with the death of the patient independently of its cause.

Questions of the trial

By means of this trial the following questions shall be answered:

Main question of the trial

1. Does intensified induction therapy with additional Etoposide lead to a different progression free survival $\underline{PFS_{\mathbf{R}}}$ measured form the time of randomization than the standard induction therapy?

Secondary questions:

2. Does the radiological response at week 24 depend on the type of induction therapy (standard or intensified induction) ?
3. Does induction therapy with additional Etoposide lead to a different $\underline{PFS_{\mathbf{D}}}$ than the standard induction therapy?
4. Does induction therapy with additional Etoposide lead to a different $\underline{EFS_{\mathbf{R}}}$ than the standard induction therapy?
5. Does induction therapy with additional Etoposide lead to a different $\underline{EFS_{\mathbf{D}}}$ than the standard induction therapy?
6. Does induction therapy with additional Etoposide lead to a different $\underline{OS_{\mathbf{R}}}$ than the standard induction therapy?

7. Does induction therapy with additional Etoposide lead to a different $\underline{OS_D}$ than the standard induction therapy?

Cox regression model

The following variables are checked with reference to their influence on the $\underline{PFS_R}$ and $\underline{EFS_R}$ with multivariable methods by Cox regression:

Histopathology

- Markers of proliferation (e.g. Ki 67 / MIB-1) (% positive cells)
- Molecular-pathologic markers (e.g. p 53 mutation) (% positive cells)
  (Quantification of markers is not standardized yet. The panel of pathologists will group these markers according to the current interpretation of their presence. See section 8.3.)

Tumor

- Tumor size preoperatively (two-/three dimensional, diameters in cm)
- Tumor size postoperatively (two-/three dimensional, diameters in cm)
- Extent of surgery (see section 16.2)
- Localization and extent within the supratentorial midline for visual pathway glioma (Dodge classification)

Dissemination

- primary/secondary
  (primary: present at diagnosis, secondary: diagnosis during follow-up)
- Type and extent of dissemination
  (nodular or leptomeningeal, descriptive extent (see section 16.1.))

Symptoms Severe, visual or neurologic symptoms relevant for the decision to start non-surgical therapy (see section 10.) will be described according to their presence or absence:

- Visual symptoms
- Neurologic symptoms
- Increased intracranial pressure
- Diencephalic syndrome

Age To investigate the „young“ and „older“ age groups according to the present strategy patients are divided into the following age groups:

- < 8 and ≥ 8 years (the young age group will be further divided into those younger than 1 year and those 1-8 years)
- To be comparable to previous trials patients are divided into the following age groups:
  < 1 year, 1 to 4, 5 to 10, > 10 years.
- Age will be analysed as a continuous variable also.

Sex

- male / female

Observation time following diagnosis before starting therapy (continuous variable)

The delay between diagnosis and the time to commence treatment has been the strongest prognostic factor in the previous trial in that those that were treated within a short period of time did worse than those that were treated after a period of observation. However, the decision to treat or not to treat was often arbitrarily taken and therefore this parameter will be studied prospectively, but will not be stratified. In the present study the indications to start non-surgical therapy (see section 10.) shall be strictly observed, in order to avoid such inaccuracies. Analysis will consider whether start of protocol therapy was according to the indications or chosen arbitrarily.

Response at week 24

Adding "response at week 24" as a possible important factor for PFS_R for the Cox regression, its influence upon the PFS_R is tested. Thus it is tested, if the "response at week 24" is suitable for predicting PFS_R. Response definitions according to section 16.3. are used.

Induction therapy (I or II) – main analysis at the time points defined

Statistical analysis

• The analysis will be done according to the intention-to-treat principle.
• Additionally, a per-protocol analysis will be performed for explorative reasons.
Per-protocol-patients are defined as follows:

Every child should receive the type of chemotherapy and the amount of chemotherapy to which it was allocated. Treatment-modifications or interruptions for toxicity are no violation of the protocol.
Children developing Carboplatin hypersensitivity will continue treatment according to protocol recommendations. This change in chemotherapy is no violation of the treatment assigned.
Premature termination due to toxicity is no protocol violation, but there should be no unreasonable or unexplained termination. Children who received more than 75 % of the possible doses are included as being treated "per protocol". Children who for other reasons than progression or toxicity have interrupted treatment early have to be censored.

• The main question will be analyzed on a significance level of α=0,05. The p-values corresponding to the secondary questions are regarded as explorative.
Additionally, the analyses will be performed separately for the group of children with chiasmatic tumors (Dodge II and III), for the group of children with all other tumors of the supratentorial midline and the group of children with tumors of all other sites outside of the supratentorial midline. These analyses are regarded as explorative.

According to the questions of the trial the following null hypothesis and test statistics follow:

1. Null hypothesis: The <u>PFS_R</u> of children on intensified induction does not differ from the <u>PFS_R</u> of children on standard induction.
This hypothesis will be analyzed by a two sided log-rank test on difference. For descriptive reasons the Kaplan Meier curves of the <u>PFS_R</u>, the quartiles of the <u>PFS_R</u> with the 95 %

confidence intervals, the PFS_R at 24 weeks, 1 year, 3 years and 5 years with the 95 % confidence intervals will be illustrated.

2. Null hypothesis: The response at week 24 does not depend upon the type of preceding induction therapy (intensified or standard induction).
This hypothesis will be analyzed by a two-sided Chi squared test. For descriptive reasons the respective frequency table will be illustrated.

3. Null hypothesis: The PFS_D of children on intensified induction does not differ from the PFS_R of children on standard induction.
This hypothesis will be analyzed by a two sided log-rank test on difference. For descriptive reasons the Kaplan Meier curves of the PFS_D, the quartiles of the PFS_D with the 95 % confidence intervals, the PFS_D at 24 weeks, 1 year, 3 years and 5 years with the 95 % confidence intervals will be illustrated.

4. Null hypothesis: The EFS_R of children on intensified induction does not differ from the EFS_R of children on standard induction.
This hypothesis will be analyzed by a two sided log-rank test on difference. For descriptive reasons the Kaplan Meier curves of the EFS_R, the quartiles of the EFS_R with the 95 % confidence intervals, the EFS_R at 24 weeks, 1 year, 3 years and 5 years with the 95 % confidence intervals will be illustrated.
For testing the null hypothesis, that the number of early progressions of the two induction therapies are not different, we will use the generalized Wilcoxon test (Breslow). The EFS_R at 24 weeks and the respective 95 % confidence intervals for both induction therapies will illustrate this.

5. Null hypothesis: The EFS_D of children on intensified induction does not differ from the EFS_D of children on standard induction.
This hypothesis will be analyzed by a two sided log-rank test on difference. For descriptive reasons the Kaplan Meier curves of the EFS_D, the quartiles of the EFS_D with the 95 % confidence intervals, the EFS_D at 24 weeks, 1 year, 3 years and 5 years with the 95 % confidence intervals will be illustrated.

6. Null hypothesis: The OS_R of children on intensified induction does not differ from the OS_R of children on standard induction.
This hypothesis will be analyzed by a two sided log-rank test on difference. For descriptive reasons the Kaplan Meier curves of the OS_R, the quartiles of the OS_R with the 95 % confidence intervals, the OS_R at 24 weeks, 1 year, 3 years and 5 years with the 95 % confidence intervals will be illustrated.

7. Null hypothesis: The OS_D of children on intensified induction does not differ from the OS_D of children on standard induction.
This hypothesis will be analyzed by a two sided log-rank test on difference. For descriptive reasons the Kaplan Meier curves of the OS_D, the quartiles of the OS_D with the 95 % confidence intervals, the OS_D at 24 weeks, 1 year, 3 years and 5 years with the 95 % confidence intervals will be illustrated.

Interim analyses and final analysis, stopping rule

Analyses will be performed after 1/3, 2/3 and all expected events occurred, unless the trial was stopped before. Both induction therapy arms are added up to evaluate the number of occurred events with respect to the expected number of events.

With an accrual period of 6 years, a follow-up period of 2 years, an accrual rate of 60 children per year, a 3-year $\underline{PFS_R}$ of 50% having standard induction therapy and 65 % having the intensified induction, a 5-year drop-out rate of 10% and the assumption of exponential distributed $\underline{PFS_R}$ and independent exponential distributed drop-out-times ($\lambda = 0.0211$), a total number of 198 events is expected. Therefore the first interim analysis is scheduled to take place after 66 events and second after 132 events.
The trial will be terminated after an interim analysis, if the main question can already be answered at this interim analysis or the chance to answer the main question is low while continuing the trial.
The criteria for stopping the trial after an interim analysis are given by a 3-step group sequential plan according to Pampallona & Tsiatis with the possibility to stop the trial in favor for the alternative and the null hypothesis [Jennison 2000]. The bounds of the 3-step group sequential design result from α=5%, power=90%, hazard ratio =1,609, progression free survival rate after 3 years of 50% and 65% for the two groups and an α-spending approach according to O'Brien & Fleming [1979] ($\Delta = 0$).

Stopping for toxicity and overall progression

Whenever a toxicity event occurs, the toxicity rate will be newly calculated. The toxicity rate will be computed as ratio of the number of study patients, which already had an event until this moment and the number of patients, which were recruited until this moment.
Relevant toxicities for this analysis are the WHO and / or CDC III° and IV° non-hematological organ-toxicities of the kidneys, the liver, the inner ear, and of the central and peripheral nervous system as well as death from toxicity.

The trial has to be stopped, if the probability for a toxic event exceeds 25 %. A probability for a toxic event of 10 % is acceptable. Having a sequential design according to Wald the trial shall be stopped, if the observed number of toxicities exceeds 6.085+0,166 x number of recruited patient. If 360 patients are recruited after 6 years, simulations show that the trial will be stopped in 99%, if the probability of a toxic event is 25%, and the trial will be stopped in 0.077% of the simulations, if the probability of a toxic event is 10 %.

This criterion has to be checked after each toxicity event.

Additionally, the $\underline{PFS_{CT}}$ (measured from the time of the start of chemotherapy) pooled over both randomized groups will be checked by an independent Data Monitoring Committee to identify a possible increase of progressions between the 6th and 12th month of chemotherapy, where therapy is given in 6-week cycles as compared to 4-week intervals as in the SIOP-LGG 1 study.
For this reason the Kaplan-Meier curves of the $\underline{PFS_{CT}}$ will be estimated. The estimates of the $\underline{PFS_{CT}}$ at 1/2 year and at 1 year will be compared with the 95 % confidence intervalls to the known $\underline{PFS_{CT}}$ at 1/2 and 1 year of the historical control groups, which were 90 % for the 1/2 year and 81 % for the 1 year point of time (Perilongo 2000).

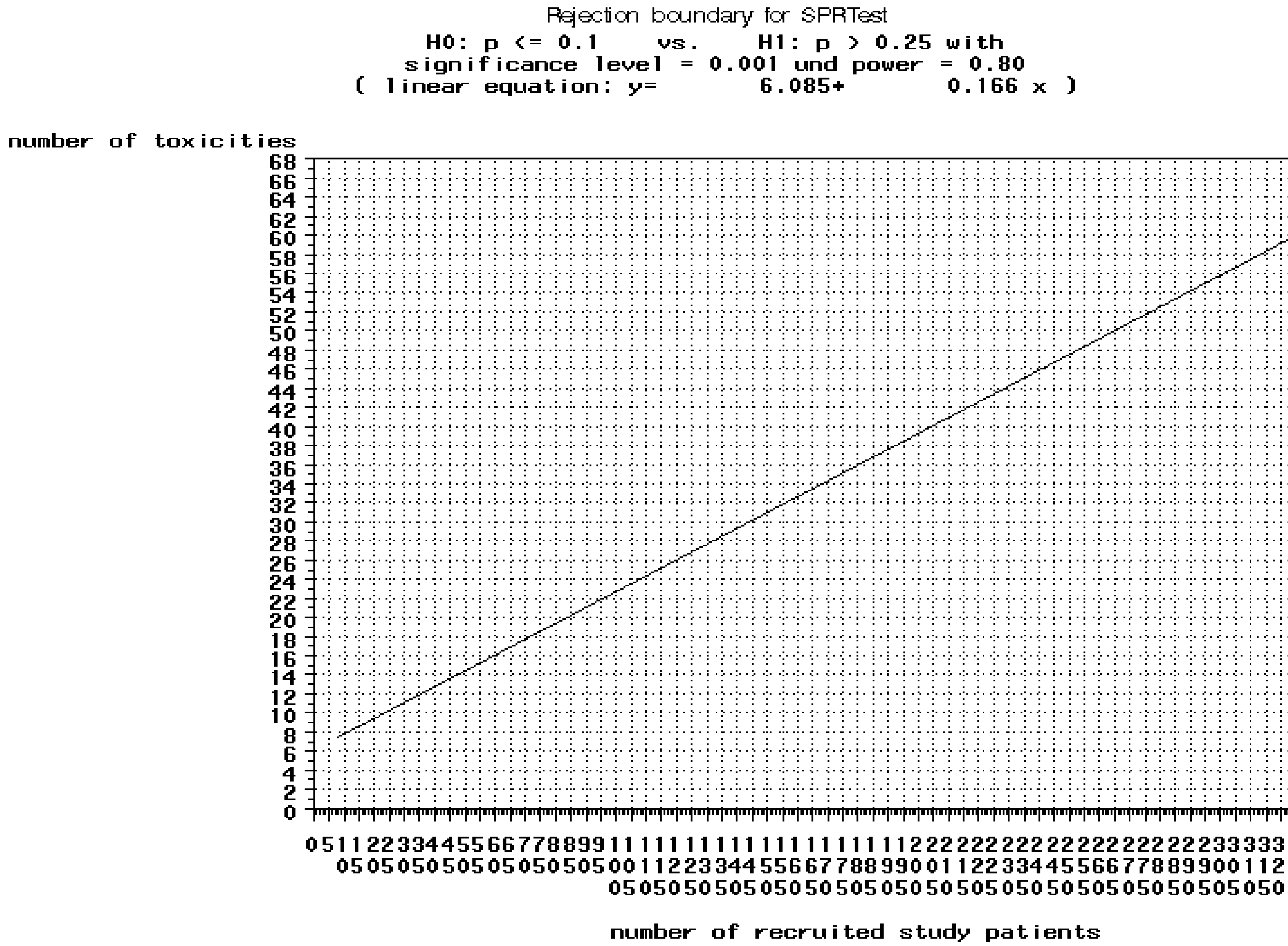

Sample size calculation

By means of this trial the use of an intensified induction chemotherapy shall be investigated. The 3 year $\underline{PFS_R}$ for the standard induction chemotherapy is supposed to be 50%. The 3 year $\underline{PFS_R}$ for the intensified therapy is estimated to be 65%. With a significance level of 5 %, an accrual period of 6 years, a follow-up period of 2 years, a supposed drop-out rate after 5 years of 10% and on the assumption of exponential distributed $\underline{PFS_R}$ and independent exponential distributed drop-out times ($\lambda = 0.0211$), 360 patients are necessary to obtain a power of 90% while performing a three step group sequential design according to Pampallona & Tsiatis explained above ("Interim analyses anddinal analysis, stopping rule")for the two-sided log-rank-test on difference. This corresponds with an annual recruitment rate of 60 patients. The sample size was calculated for an one-step design with nQuery Advisor 3.0 and the sample size was adapted to the 3-step group sequential design according to [1].

Estimated recruitment rate per year and country:

It is predicted that the annual recruitment rates for the participating national groups for children unaffected by NF I with glioma in and outside of the supratentorial midline would be:

Group 1:	Germany	15	per year
	United Kingdom	15	
	Italy	11	
	Nordic countries	6	
Group 2:	approximately 30 % of the group 1		

No exact numbers can be calculated for the other participating countries.

Modifications of the protocol

The design of this trial may be changed, if necessary, in case of new important discoveries. Modifications of the protocol will be made only in form of written amendments and with agreement of the study committee. The respective ethic commissions have to be informed of the modifications. The patient information has to be changed according to the modifications of the protocol.

If an adaptation of the group sequential design is necessary – e.g. because of a low recruitment rate – the respective changes of the time points, number of interim analyses, maximal sample size and α-spending function will be done according to the conditional rejection error probability method by Schäfer und Müller [2001]. The modifications can be done during a planned or unplanned interim analysis on the basis of the observed data collected so far. The corresponding conditional rejection error probability functions are defined by Schäfer [2001]. If a design change is made the time point, the data file of the trial, all calculations and the description of the new group sequential design have to be recorded in the amendment.

17.1.2. Low grade glioma of all sites in children affected by Neurofibromatosis NF I (group 3 according to section 12.)

Design of the trial

Chemotherapy according to this protocol is applied to delay or obviate the start of radiotherapy compared with a historical control group.
In the trial SIOP - LGG 1 the NF1-patients younger than 5 years were treated with a 12 months chemotherapy, which was shorter than the 18 months chemotherapy of this protocol. For children older than 5 years primary radiotherapy was recommended, but only a small proportion of the older children did proceed with primary radiotherapy and had chemotherapy instead. This cohort is defined as the historical control group.

This therapy optimization trial is multinational, multicenter , prospective and historically controlled.
The accrual period of the trial amounts to 6 years, followed by an observation period of 2 years.

End points

Definitions of PFS, EFS and OS are according to section I.

1. RFS_D: Radiotherapy free survival: Interval starting with the day of diagnosis and ending with the start of radiotherapy or death of the patient independently of its

cause.For analysis of radiotherapy-free survival the event “death” is counted as an event as well.

2. PFS_{CE}: Time from the end of the chemotherapy up to an event:
   Definition of event: death (for all reasons)
   progression of a residual tumor (section 16.3.)
   relapse following previous complete remission (section 16.3.)
   appearance of new or progression of existing metastasis
   This end point is only defined for those patients who will not have a progression until the end of the chemotherapy.

Questions of the trial

Explorative questions:

1. Does the prolonged chemotherapy (18 months) lead to a different PFS_D for the whole group of patients with NF1 - in comparison with a historical control group, who received radiotherapy or a shorter chemotherapy.

2. Does the prolonged chemotherapy lead to a different EFS_D for the whole group of patients with NF1 - in comparison with a historical control group, who received radiotherapy or a shorter chemotherapy.

3. Does the prolonged chemotherapy (18 months) lead to a different RFS_D - for patients with NF1 younger than 5 years - in comparison with a historical control group (NF I, younger than 5 years), who received a shorter chemotherapy (12 months).

4. Does the prolonged chemotherapy (18 months) lead to a different PFS_D - for patients with NF1 younger than 5 years - in comparison with a historical control group (NF1, younger than 5 years), who received a shorter chemotherapy (12 months)?

5. Does the prolonged chemotherapy leads to another PFS_D – for patients with NF1, with an age above 5 years – in comparison with a historical control group (NF1, age above 5 years), who received radiotherapy?

6. Does the prolonged chemotherapy (18 months) lead to a different EFS_D – for patients with NF I younger than 5 years – in comparison with a historical control group (NF I, younger than 5 years), who received a shorter chemotherapy (12 months)?

7. Does the prolonged chemotherapy (18 months) lead to a different EFS_D – for patients with NF I age above 5 years – in comparison with a historical control group (NF I, age above 5 years), who received radiotherapy?

8. What is the RFS_D of the whole group (NF1, all ages, only new trial) ?

9. Does the strategy of this protocol lead to a different OS_D for the children with NF I of all ages as compared to the previous protocol ?

10. Does the prolonged chemotherapy reduce / prevent the occurrence of progression after the end of the chemotherapy – for children with NF1 younger than 5 years – in comparison to a historical control group (NF1, younger than 5 years), who received a shorter chemotherapy?

Statistical analysis

It is anticipated that the SIOP-LGG 2004 strategy will maintain the good results for NF I children from the previous trials, with less therapy for the French children and only marginally more for the others.

The analysis will be done according to the intention-to-treat principle. Additionally there will be made a per-protocol analysis.
Per-protocol-patients are defined as follows: Children that received either intensified induction treatment and/or consolidation option B although recommendation for this treatment group is standard induction with consolidation option A. Aside the definitions from part I apply.

All analyses will be performed exploratively. Therefore the repective p-values are regarded as descriptive and no significance level is given.

According to the questions of the trial the following analyses will be done:

1. Null hypothesis: The $\underline{PFS_D}$ of children on the prolonged chemotherapy does not differ from the $\underline{PFS_D}$ of children of the historical control group, who received radiotherapy or a shorter chemotherapy.
   This hypothesis will be analyzed by a two sided log-rank test on difference. For descriptive reasons the Kaplan Meier curves of the $\underline{PFS_D}$, the quartiles of the $\underline{PFS_D}$ with the 95 % confidence intervals, the $\underline{PFS_D}$ at 24 weeks, 1 year, 3 years and 5 years with the 95 % confidence intervals will be illustrated.

2. Null hypothesis: The $\underline{EFS_D}$ of children on the prolonged chemotherapy does not differ from the $\underline{EFS_D}$ of children of the historical control group, who received radiotherapy or a shorter chemotherapy.
   This hypothesis will be analyzed by a two sided log-rank test on difference. For descriptive reasons the Kaplan Meier curves of the $\underline{EFS_D}$, the quartiles of the $\underline{EFS_D}$ with the 95 % confidence intervals, the $\underline{EFS_D}$ at 24 weeks, 1 year, 3 years and 5 years with the 95 % confidence intervals will be illustrated.

3. Null hypothesis: The $\underline{RFS_D}$ of children on the prolonged chemotherapy (younger than 5 years) does not differ from the $\underline{RFS_D}$ of children of the historical control group (younger than 5 years, NF1), who received a shorter chemotherapy.
   This hypothesis will be analyzed by a two sided log-rank test on difference. For descriptive reasons the Kaplan Meier curves of the $\underline{RFS_D}$, the quartiles of the $\underline{RFS_D}$ with the 95 % confidence intervals, the $\underline{RFS_D}$ at 24 weeks, 1 year, 3 years and 5 years with the 95 % confidence intervals will be illustrated.

4. Null hypothesis: The $\underline{PFS_D}$ of children on the prolonged chemotherapy (younger than 5 years) does not differ from the $\underline{PFS_D}$ of children of the historical control group (younger than 5 years, NF1), who received a shorter chemotherapy.
   This hypothesis will be analyzed by a two sided log-rank test on difference. For descriptive reasons the Kaplan Meier curves of the $\underline{PFS_D}$, the quartiles of the $\underline{PFS_D}$ with the 95 % confidence intervals, the $\underline{PFS_D}$ at 24 weeks, 1 year, 3 years and 5 years with the 95 % confidence intervals will be illustrated.

5. Null hypothesis: The $\underline{PFS_D}$ of children on the prolonged chemotherapy (older than 5 years) does not differ from the $\underline{PFS_D}$ of children of the historical control group (older than 5 years, NF1), who received radiotherapy.
   This hypothesis will be analyzed by a two sided log-rank test on difference. For descriptive reasons the Kaplan Meier curves of the $\underline{PFS_D}$, the quartiles of the $\underline{PFS_D}$

with the 95 % confidence intervals, the $\underline{PFS_D}$ at 24 weeks, 1 year, 3 years and 5 years with the 95 % confidence intervals will be illustrated.

6. Null hypothesis: The $\underline{EFS_D}$ of children on the prolonged chemotherapy (younger than 5 years) does not differ from the $\underline{EFS_D}$ of children of the historical control group (younger than 5 years, NF1), who received a shorter chemotherapy.
   This hypothesis will be analyzed by a two sided log-rank test on difference. For descriptive reasons the Kaplan Meier curves of the $\underline{EFS_D}$, the quartiles of the $\underline{EFS_D}$ with the 95 % confidence intervals, the $\underline{EFS_D}$ at 24 weeks, 1 year, 3 years and 5 years with the 95 % confidence intervals will be illustrated.

7. Null hypothesis: The $\underline{EFS_D}$ of children on the prolonged chemotherapy (older than 5 years) does not differ from the $\underline{EFS_D}$ of children of the historical control group (older than 5 years, NF1), who received radiotherapy.
   This hypothesis will be analyzed by a two sided log-rank test on difference. For descriptive reasons the Kaplan Meier curves of the $\underline{EFS_D}$, the quartiles of the $\underline{EFS_D}$ with the 95 % confidence intervals, the $\underline{EFS_D}$ at 24 weeks, 1 year, 3 years and 5 years with the 95 % confidence intervals will be illustrated.

8. For descriptive reasons the Kaplan Meier curves of the $\underline{RFS_D}$, the quartiles of the $\underline{RFS_D}$ with the 95 % confidence intervals, the $\underline{RFS_D}$ at 24 weeks, 1 year, 3 years and 5 years with the 95 % confidence intervals will be illustrated.

9. Null hypothesis: The $\underline{OS_D}$ of children on the prolonged chemotherapy (all ages) does not differ from the $\underline{OS_D}$ of children of the historical control group (all ages, NF1), who received radiotherapy or a shorter chemotherapy.
   This hypothesis will be analyzed by a two sided log-rank test on difference. For descriptive reasons the Kaplan Meier curves of the $\underline{OS_D}$, the quartiles of the $\underline{OS_D}$ with the 95 % confidence intervals, the $\underline{OS_D}$ at 24 weeks, 1 year, 3 years and 5 years with the 95 % confidence intervals will be illustrated.

10. Null hypothesis: The $\underline{PFS_{CE}}$ of children on the prolonged chemotherapy (younger than 5 years) does not differ from the $\underline{PFS_{CE}}$ of children of the historical control group (younger than 5 years, NF1), who received a shorter chemotherapy.
    This hypothesis will be analyzed by a two sided log-rank test on difference. For descriptive reasons the Kaplan Meier curves of the $\underline{PFS_{CE}}$, the quartiles of the $\underline{PFS_{CE}}$ with the 95 % confidence intervals, the $\underline{PFS_{CE}}$ at 24 weeks, 1 year, 3 years and 5 years with the 95 % confidence intervals will be illustrated.

17.2. Radiotherapy Group

17.2.1. Low grade glioma of all sites in children not affected by Neurofibromatosis NF I (group 1 and 2 according to section 12.)

Design of the trial

The aim of the trial is to assess outcome in children with low grade glioma of all sites necessitating radiotherapy as non-surgical therapy. (according to patient eligibility criteria

(section 9.) and indication for non-surgical therapy (section 10.) as well as brachytherapy irrespective of these criteria).
This therapy optimization trial is multinational, multicenter, prospective and historically controlled.
The study patients of the SIOP-LGG 1996 study who received radiotherapy (primary or secondary radiotherapy) are defined as the historical control group.
The accrual period of the trial is 6 years followed by an observation period of 2 years.
It is expected that 240 patients will be recruited during 6 years.

End points

According to the different questions the following end points are defined:
For definition of progression and relapse referral is made to protocol section 16.3.

1. $\underline{PFS_{RT}}$: Progression free survival measured from the time of start of radiotherapy (brachytherapy): Time of start of radiotherapy (brachytherapy) up to one of the following events:
   - death (for all reasons)
   - progression of a residual tumor (section 16.3.)
   - relapse following previous complete remission (section 16.3.)
   - appearance of new or progression of existing metastasis (section 16.3.)

2. $\underline{PFS_{D}}$: Progression free survival measured from the time of diagnosis: Time from diagnosis up to an event defined in 1.

3. $\underline{EFS_{RT}}$: Event free survival measured from the time of start of radiotherapy (brachytherapy): Time of start of radiotherapy (brachytherapy) up to one of the following events:
   - death (for all reasons)
   - progression of a residual tumor (section 16.3.)
   - relapse following previous complete remission (section 16.3.)
   - appearance of new or progression of existing metastasis (section 16.3.)
   - severe adverse event / toxicity (section 16.4.)
   - appearance of secondary malignant neoplasm (section 16.4.)

4. $\underline{EFS_{D}}$: Event free survival measured from the time of diagnosis: Time from diagnosis up to an event defined in 3.

5. $\underline{OS_{RT}}$: Overall survival measured from the time of start of radiotherapy (brachytherapy): Interval starting with the day of start of radiotherapy (brachytherapy) and ending with the death of the patient independently of its cause.

6. $\underline{OS_{D}}$: Overall survival measured from the time of diagnosis: Interval starting with the day of diagnosis and ending with the death of the patient independently of its cause.

7. Radiological and clinical response (vision, neurological functions) measured after end of radiotherapy (brachytherapy) and at 6 and 12 months: Complete, partial, objective responses and stable disease will be considered positive responses in this protocol. Response definitions according to section 16.3. are used.

8. Time to maximal radiological and clinical response (vision, neurological functions).

   <u>Subgroups</u>:

- Patients receiving external radiotherapy (excluding craniospinal irradiation)
- Patients receiving brachytherapy
- Patients receiving craniospinal irradiation

Questions of the trial

By means of this trial the following questions shall be investigated exploratively:

1. Does the use of modern treatment techniques in radiotherapy lead to a different PFS_{RT} (EFS_{RT}, OS_{RT}, PFS_D, EFS_D, OS_D) in comparison with the radiotherapy of the historical control group (SIOP-LGG 1996)?
(Subgroups: patients who receive external radiotherapy and patients who receive brachytherapy)

2. Is PFS_{RT} (EFS_{RT}, OS_{RT}, OS_D) different between patients who receive primary radiotherapy and patients who receive radiotherapy after chemotherapy has failed?
(Subgroups: patients who receive external radiotherapy and patients who receive brachytherapy)

3. What is the PFS_{RT} (EFS_{RT}, OS_{RT}, PFS_D, EFS_D, OS_D) of patients who started craniospinal irradiation after metastatic disease?

4. What are the rates of radiological and clinical response measured after 3 months (end of radiotherapy) and 6 and 12 months after end of radiotherapy?
(Subgroups: patients who receive external radiotherapy, patients who receive brachytherapy and patients who receive craniospinal irradiation)

5. Is the radiological and clinical response (vision, neurological functions) measured after 3 months (end of radiotherapy (brachytherapy)) and at 6 and 12 months different between primary treatment or after chemotherapy has failed?
(Subgroups: patients who receive external radiotherapy, patients who receive brachytherapy and patients who receive craniospinal irradiation)

6. What is the rate of maximal radiological and clinical response?
(Subgroups: patients who receive external radiotherapy, patients who receive brachytherapy and patients who receive craniospinal irradiation)

7. What is the time to maximal radiological and clinical response for patients on primary treatment or for patients, who receive radiotherapy after chemotherapy has failed?
(Subgroups: patients who receive external radiotherapy and patients who receive brachytherapy)

8. Are modern treatment techniques associated with marginal or out of field treatment failures?

Cox regression model

The following variables are checked with reference to their influence on the <u>PFS_{RT}</u> and <u>EFS_{RT}</u> with multivariable methods by Cox regression:

Histopathology

Markers of proliferation (e.g. Ki 67 / MIB-1) (% positive cells)

Molecular-pathologic markers (e.g. p 53 mutation) (% positive cells)

(Quantification of markers is not standardized yet. The panel of pathologists will group these markers according to the current interpretation of their presence. See section 8.3.)

Tumor

Tumor size preoperatively (Product of the two largest diameters in cm)

Tumor size postoperatively (Product of the two largest diameters in cm)

Extent of surgery (see section 16.2)

Localization and extent within the supratentorial midline for visual pathway glioma (Dodge classification)

Treatment

Radiotherapy as primary treatment / as salvage treatment

Brachytherapy as primary / salvage treatment

Dissemination

primary/secondary

(primary: present at diagnosis, secondary: diagnosis during follow-up)

Type and extent of dissemination

(nodular or leptomeningeal, descriptive extent (see section 16.1.))

Symptoms

Severe, visual or neurologic symptoms relevant for the decision to start non-surgical therapy (see section 10.) will be described according to their presence or absence:

Visual symptoms

Neurologic symptoms

Increased intracranial pressure

Diencephalic syndrome

Age

To investigate the „young“ and „older“ age groups according to the present strategy patients are divided into the following age groups:

< 1 year, 1-8 years, ≥ 8 years

To be comparable to previous trials patients are divided into the following age groups:

< 1 year, 1 to 4, 5 to 10, > 10 years.

Age will be analysed as a continuous variable also.

Sex

male / female

Observation time following diagnosis before starting therapy (continuous variable)

The delay between diagnosis and the time to commence treatment has been the strongest prognostic factor in the previous trial in that those that were treated within a short period of time did worse than those that were treated after a period of observation. However, the decision to treat or not to treat was often arbitrarily taken and therefore this parameter will be studied prospectively.

In the present study the indications to start non-surgical therapy (see section 10.) shall be strictly observed, in order to avoid such inaccuracies.

Last known response (before an event according to the definition of PFS_{RT} occurs)

Patients, in whom an event occurred before the first radiological examination to evaluate the response was given, will be censored.
Response definitions according to section 16.3. are used.

Statistical analysis

The analysis will be done according to the intention-to-treat principle. Additionally there will be made a per-protocol analysis.
Per-protocol-patients are defined as follows: Every child that received radiotherapy (or brachytherapy) according to the eligibility criteria. Treatment-modifications or interruptions for toxicity are no violation of the protocol. Premature termination due to toxicity is no protocol violation, but there should be no unreasonable or unexplained termination. Children who received a total dose which vary only less than 10% or more than 7% the defined dose prescriptions are included as being treated “per protocol”.

All analyses will be performed exploratively. Therefore the respective p-values are regarded as descriptive and no significance level is given.

According to the questions of the trial the following analyses will be done:

1. Null hypothesis: PFS_{RT} (EFS_{RT}, OS_{RT}, PFS_D, EFS_D, OS_D) of children treated according to protocol SIOP-LGG 2004 does not differ from PFS_{RT} (EFS_{RT}, OS_{RT}, PFS_D, EFS_D, OS_D) of children treated according protocol SIOP-LGG 1996.
   This hypothesis will be analyzed by a two-sided log-rank test on difference. For descriptive reasons the Kaplan Meier curves of PFS_{RT} (EFS_{RT}, OS_{RT}, PFS_D, EFS_D, OS_D), the quartiles of PFS_{RT} (EFS_{RT}, OS_{RT}, PFS_D, EFS_D, OS_D) with the 95 % confidence intervals, PFS_{RT} (EFS_{RT}, OS_{RT}, PFS_D, EFS_D, OS_D) rates at 24 weeks, 1 year, 3 years and 5 years with the 95 % confidence intervals will be given.
   These analyses will be done including all study patients treated according to protocol SIOP-LGG 1996 and SIOP-LGG 2004, who received radiotherapy. In a second step this analysis will be done separately for study patients, who received brachytherapy and for patients, who received external radiotherapy.

2. Null hypothesis: PFS_{RT} (EFS_{RT}, OS_{RT}, OS_D) of children on primary radiotherapy does not differ from PFS_{RT} (EFS_{RT}, OS_{RT}, OS_D) of patients who receive radiotherapy after chemotherapy has failed?
   This hypothesis will be analyzed by a two-sided log-rank test on difference. For descriptive reasons the Kaplan Meier curves of PFS_{RT} (EFS_{RT}, OS_{RT}, OS_D), the quartiles of PFS_{RT} (EFS_{RT}, OS_{RT}, OS_D) with the 95 % confidence intervals, PFS_{RT} (EFS_{RT}, OS_{RT}, OS_D) rates at 24 weeks, 1 year, 3 years and 5 years with the 95 % confidence intervals will be given.
   These analyses will be done including all study patients treated according to protocol SIOP-LGG 1996 and SIOP-LGG 2004, who received radiotherapy. In a second step this analysis will be done separately for study patients, who received brachytherapy and for patients, who received external radiotherapy.

3. For the subgroup of patients, who receive craniospinal irradiation because of metastatic disease the following descriptive analyses will be done:
   For descriptive reasons the Kaplan Meier curves of PFS_{RT} (EFS_{RT}, OS_{RT}, PFS_D, EFS_D, OS_D), the quartiles of PFS_{RT} (EFS_{RT}, OS_{RT}, PFS_D, EFS_D, OS_D) with the 95 % confidence intervals, PFS_{RT} (EFS_{RT}, OS_{RT}, PFS_D, EFS_D, OS_D) rates at 24 weeks, 1 year, 3 years and 5 years with the 95 % confidence intervals will be given.

4. For descriptive reasons the frequencies of radiological and clinical response at month 3, 6 and 12 with 95 % confidence intervals will be illustrated. This analysis will be done separately for study patients who received brachytherapy, for patients, who received external radiotherapy, and for patients who received craniospinal irradiation.

5. Null hypothesis: The radiological and clinical response measured after 3 months (end of radiotherapy) and at 6 and 12 months after the end of radiotherapy does not differ between patients receiving primary radiotherapy and patients receiving radiotherapy after chemotherapy has failed.
   For each time of evaluation this hypothesis will be analyzed by a two-sided Chi-squared test. For descriptive reasons the respective frequency table and the corresponding 95 % confidence intervals will be given.
   This analysis will be done separately for study patients who received brachytherapy, for patients, who received external radiotherapy, and for patients who received craniospinal irradiation.

6. For descriptive reasons the frequencies of maximal radiological and clinical response measured in the first year (MRI at month 3, 6 12) after end of radiotherapy will be illustrated.
   This analysis will be done separately for study patients who receive brachytherapy, for patients, who receive external radiotherapy, and for patients who receive craniospinal irradiation.

Radiolog. Response	MRI 3 month	MRI 6 month	MRI 12 month	Best of MRI 3,6,12 month
CR				
PR				
OR				
SD				
PD				
	100%	100%	100%	100%

7. For the subgroup of patients who reach CR (PR, OR, SD, PD) as best response in the first year after the end of radiotherapy the frequencies of the time points when this response was reached will be given.

Best response	Reached	Reached at month 3 for the first time	Reached at month 6 for the first time	Reached at month 12 for the first time
CR				
PR				
OR				
SD				
PD				

This analysis will be done separately for patients on primary radiotherapy and for patients who receive radiotherapy after chemotherapy has failed.
Additionally this analysis will be done separately for patients who receive external radiotherapy and for patients who receive brachytherapy.

8. The rates of marginal or out of field treatment failures will be given.

Additionally, the analyses will be performed separately for the group of children with pure chiasmatic tumors (Dodge II), for the group of children with all other tumors of the supratentorial midline, the group of children with tumors of all other sites outside of the supratentorial midline.

Stopping for toxicity

The toxicity of children, who receive craniospinal irradiation because of metastatic LGG, will be observed.
Whenever a toxicity event occurs, the toxicity rate will be newly calculated. The toxicity rate will be computed as ratio of the number of study patients, who already had an event until this moment and the number of patients, who were recruited until this moment.
Relevant toxicities for this analysis are the WHO and / or CDC III° and IV° non-hematological organ-toxicities of the skin, mucosa, inner ear, and of the central and peripheral nervous system as well as death from toxicity.

The trial has to be stopped, if the probability for a toxic event exceeds 30 %. A probability for a toxic event of 10 % is acceptable. Having a sequential design according to Wald the trial shall be stopped, if the observed number of toxicities exceeds 1,706 + 0,186 x number of recruited patients. If 20 patients are recruited after 6 years, simulations show that the trial will be stopped in 69 %, if the probability of a toxic event is 30%, and the trial will be stopped in 4 % of the simulations, if the probability of a toxic event is 10 %.
This criteria has to be checked after each toxicity event.

Additionally, the trial has to be stopped, if more than one patient dies because of radiotherapy.

Since the effectiveness of the stopping rule given above depends on the recruited number of patients (which is small) the final decision to stop the trial is incumbent upon the study committee.

18. Organisational Issues SIOP LGG 2004

18.1. Institutional commitment

All institutions participating in the study must declare their commitment to do so according to the guidelines of the joined national study groups.
If individual centers from countries, whose national group does not take part as a whole, want to join the study, they shall link the national data center of one of the participating pediatric onocology groups.
All patients diagnosed with a low grade glioma by the participating institutions have to be registered and treated according to the guidelines of this protocol during the study period.

18.2. Study period

The study will be activated on April 1st, 2004. Patient recruitment during the main study phase will extend for 6 years depending on the actual rate of enrolment. A two year follow-up phase is planned. Following evaluation of the late first interim analysis the international trial committee decided to prolong the recruitment period for 2 years.

18.3. Protocol organisation

One common international protocol will be used by all national groups. This master protocol is written in English and is kept by members of the core committee. National groups or centers may provide translations of the English protocol.

Each national group is responsible to distribute the protocol to the members/institutions within their group.

Subsequent to finalisation, any amendments to the protocol must be agreed by all co-operative groups. The coordinating center and the core committee members will issue a revised version of the protocol, if and when required.
Addenda may be added independently by any groups to adress local needs, provided they have no bearing on the essential aims of the international protocol.

18.4. Study forms

One common set of forms will be used by all cooperative groups. The English language master version of the study forms will be held at the coordinating center and by the core committee members. Translations are within the responsibility of the national study centers.

The study center of each national group will be responsible for distribution of forms to institutions within that national group.

In case the international study does require additional information, amendments to forms have to be agreed upon by all cooperative groups. The central data center will be responsible to distribute the amended forms.
Additional forms may be produced within national study groups for data collections that are specific for that national group and exceed the international data set.

18.5. Documentation and data handling

Patients may be registered for the Low grade glioma study LGG 2004 only after he/she and/or his/her legal guardian has consented to registration and data saving. The appropriate forms for

registration procedure from the addendum of this protocol have to be used by the institutions and forwarded to the national data center. All forms must indicate the institution, name and signature of the physician responsible.

Each national group shall hold the database for its own patients and shall be responsible for data quality according to local practice. Forms returned from the treating institutions will be stored at the respective national data centers for time periods conformal to national law.
The content of the national database shall be identical to the data collected on the study forms.

All data from the national databases required for the conduct of the international study will be transferred by information transfer tecchniques to the international data center in 3-monthly intervals.
The International data base will be held at the Istituto Oncologico Veneto, Clinical Trials & Biostatistic Unit, "SIOP-LGG 2004", Busonera Hospital, I-35128 Padova, Italy.

It is most probable that future developments of information transfer will change the ways data are entered at the level of the participating institutions, the national study offices and the international study office during the currency term of this study. Especially the possibilities of remote data entry (RDE) will alter the traditional paper-based flow of data. If access to RDE is a realistic option, the national study members and the members of the core committee will discuss the implications of this technique and present the results to all national groups before action is taken.

If RDE is adopted, a high standard level of data confidentiality and security should be guaranteed.
In detail:

- The International common data base will not contain individual personal information
- All traffic with the server will be encrypted.
- Each user at each site will have its own User ID and Password.

The system will ensure:

- appropriate and regular backup on electronic media of all data, to permit restoration in case of loss or damage of the data base,
- operation tracking log (for each user: registration of any operation),
- electronic data audit trails (creation of a data base of original entries/modifications with identification of date, time, source and user identity),
- disaster recovery procedures.

18.6. Confidentiality of patient data

The use of patient names for identification on paper forms and in each data base will follow national practice. An abbreviated patient identifier will be used for data transfer and for the master database.

National and European legal rules concerning data handling will be observed.

18.7. Data quality control

On receipt of forms at each data center, common range and logical checks will be carried out on the data prior to entering into the national or to transfer into the international database. Criteria for this check or their changes/amendments will be agreed upon by the represented national groups

Errors noted in the national and/or master data base will be reported back to the center/institution of origin. Corrections can only be made using query forms.

18.8. Data analysis and monitoring

Reports on the international study progress will be prepared yearly, describing accrual of the patients, distribution among the strategy groups, local therapy modalities and toxicity of the treatments given. Data will be published as abstracts at each SIOP meeting.

The international study committee shall meet as appropriate to consider patient accrual, eligibility, treatment allocation and outcome and ensure a smooth conduct of the study.

Results of the interim analysis of response and progression free survival and of toxicity shall be reported to the International Data Monitoring Committee (IDMC) as scheduled by the protocol. The IDMC may recommend early stopping, continuation of or extension of the study to the international study committee.

18.9. Documentation of adverse events

Any life threatening event must be reported immediately by the treating physician to the national data center, i.e. within the next working day, and followed-up by the treating institution, regardless of whether or not it falls within the categories listed in section 16.4. The information must be relayed to the other data centers for further reporting according to GCP guidelines. Toxicity criteria are applied according to the publication of common toxicity criteria uniformly for all national groups.

18.10. Independent Data Monitoring and Safety Committee (DMSC)

An independent data monitoring committee composed of four international experts will monitor the progress of the study on ethical and scientific grounds.

The role of the IDMC will be:

- To review the accrual rate and to be involved with all interim analyses according to the statistical plan.

Each interim analysis will be reported to the DMSC. These interim analyses will remain confidential.
On the basis of these analyses the DMSC will recommend whether the study can continue, whether it has to be extended or changed or terminated prematurely.

- To monitor toxicity of all treatments, but especially toxicity of the chemotherapy arms and severe ad verse events.

Every 6 months a report of toxicity will be prepared by the international study center and the statistician of the study and circulated among the participating national groups and to the DMSC.
The DMSC will review these interim toxicity data and any relevant information will be forwarded to each study coordinator. Problems and patterns of major toxicity shall be analysed to prevent major toxicity endangering the conduct of the study.

- To compare the results of the on-going study to reports from other related study groups or institutions which may have implications for the aims of the study.

The DMSC will review reports of related studies performed by other groups or organisations to determine whether such information materially affects the aims or preliminary findings of the trial. In case that interim analyses or the results of other studies implicate that the study questions have been answered, the DMSC has to decide in conjunction with the international study committee about the continuation of the current study.

- Other

The DMSC will be asked to review any major modification to the study proposed by the study committee prior to its implementation.

18.11. Follow-up

All registered patients shall be followed up by the national cooperative group study centers during and after completion of treatment according to the study. This also refers to patients off-study for any reason (e.g. toxicity).

18.12. Institutional/local ethical approval and patient's consent

Institutional / local ethical approval must follow national practice. The national and/or local ethics committee has to be contacted and a positiv vote has to be obtained prior to starting patient recruitment. The ethics committee has to be informed about major toxic events (severe adverse events - study section 18.9., documentation of adverse events 16.4. severe adverse events, including second malignant neoplasm.)
Accepted national procedures for patient consent as documented are to be used.

The patient's and/or parent's written consent to participate in the study must be obtained after a full explanation has been given of the treatment options including the conventional and generally accepted methods of treatment and the manner of treatment allocation.
If the patient is a minor, the treatment must be explained to and consent received from his/her guardian. Additionally the child should receive an explanation as to his/her means of understanding and should give consent as well, if he/she is able to do so. Enough time and the opportunity to discuss participation before the decision for and start of treatment have to be given. The right of a patient to refuse to participate without giving reasons must be respected.

Consent for participation in the study and for data management will be obtained separately.

After the patient has entered the trial the physician must be free to give alternative treatment to that specified in the protocol at any stage, if he/she feels it to be in the best interest of the patient, but the reasons for doing so should be recorded, and the patient will need to remain in the study for the purpose of follow-up and data analysis according to the treatment option to which he/she had been allocated.
Similarly the patient must remain free to withdraw at any time from the study and the protocol treatment or to withdraw his/her data from the study without giving reasons and without prejudicing his/her further treatment.

All patients and/or their parents must give written consent to inclusion into the trial, data processing and – if applicable – to sending diagnostic material to reference institutions, which in all participating countries has to conform to the national data protection legislation.
Administrative documents, consent forms and copies of the study documentation of a study patient have to be kept according to set archival terms.

This study will observe the rules for clinical research set out in the declaration of Helsinki in its latest form (Edinburgh, Scotland, 2000), the WHO and EC rules of “Good Clinical Practice” (ICH GCP: International Conference on Harmonisation – Good Clinical Practice, effective 17.01.1997), and the involved countries’ laws.

18.13. Publication policy

Data relating to the present study SIOP - LGG 2004 must not be reported or published without prior consultation of the study chairmen, but side topics may be reviewed separately. Any publication arising from this study will have to acknowledge the contributing members/hospitals besides the regular listing of the authors of the paper. Additionally the specific requirements for listing of authors in different journals have to be respected.

A final report of SIOP - LGG 2004 will be provided within 5 years after the completion of the projected patient accrual by the “International Consortium on low grade glioma ” and all contributors be listed with their individual contributions in an appendix.

18.14. Associated research

Associated research is encouraged by the international and national study groups. Projects will mostly include limited numbers of patients or limited material, but hopefully will help to further understand the naturally erratic biologic behaviour of these tumors. Some projects are listed in this protocol, others may emerge during the conduct of the study. Participation in these studies is highly appreciated. Further information is available from the study centers.

19. Associated Research SIOP LGG 2004

SIOP

1. A phase II study of vinblastine sulphate injection in children with recurrent or refractory low grade glioma.

Investigator: Eric Bouffet, Toronto, Canada

2. Validation of methods of visual assessment in children with optic pathwa tumors.

Investigator: Ian Simmons, Alistair Fielder, Susan Picton, Adam Glaser, United Kingdom

Inquiry:

3. Analysis of tumor tissue of Disseminated low grade glioma by molecular genetic techniques (Comparative genomic hybridisation)

Investigator: Uri Tabori, Tel Hashomer, Israel

Germany

1. Atypical and clinically malignant pilocytic astrocytoma in children.

Investigator: T. Pietsch, H. Radner, Bonn

2. Treatment associated late effects following radiation therapy of malignancies in childhood and adolescents.

Investigator: N. Willich, A. Schuck, Münster

20. Literature SIOP LGG 2004

Besides all papers cited in the protocol, this list contains a number of references of additional interest for various aspects of low grade glioma that cannot be detailed in the frame of this therapy protocol.

Aaronson N, Ahmedzai S, Bergman B *et al.* QLQ-C30: a quality of life instrument for use in international clinical trials in oncology. J Natl Cancer Inst 1993;85:365-376.

Abdel-Wahab M, Corn B, Wolfson A, Raub W, Gaspar LE, Curran W, Jr., Bustillo P, Rubinton P, Markoe A. Prognostic factors and survival in patients with spinal cord gliomas after radiation therapy. Am J Clin Oncol 1999; 22: 344-351

Abdollahzadeh M, Hoffman HJ, Blazer SI, Becker LE, Humphreys RP, Drake JM, Rutka JT. Benign cerebellar astrocytoma in childhood: experience at the Hospital for Sick Children 1980-1992. Child Nerv Sys 1994; 10: 380-383.

Abdulrauf SJ, Edvardsen K, Ho KL, Yang XY, Rock JP, Rosenblum ML. Vascular endothelial growth factor expression and vascular density as prognostic markers of survival in patients with low grade astrocytoma. J Neurosurg 1998; 88: 513-520.

Acar Z, Tanriover N, Kafadar AM, Gazioglu N, Oz B, Kuday C. Chiasmatic low grade glioma presenting with sacral intradural spinal metastases. Child Nerv Sys 2000; 16: 309-311.

Adan L, Trivin C, Sainte-Rose C, Zucker JM, Hartmann O, Brauner R. GH deficiency caused by cranial irradiation during childhood: factors and markers in young adults. J Clin Endocrinol Metab 2001; 86: 5245-5251

Albright AL, Price RA, Guthkelch AN. Diencephalic gliomas of children. Cancer 1985; 55: 2789-2793

Albright AL, Guthkelch AN, Packer RJ, Price RA, Routeke LB. Prognostic factors in pediatric brain-stem gliomas. J Neurosurg 1986; 65: 751-755.

Albright AL, Packer RJ, Zimmerman R, Rorke LB, Boyett J, Hammond GD. Magnetic resonance scans should replace biopsies for the diagnosis of diffuse brainstem gliomas: A report from the Children's Cancer Group. Neurosurgery 1993; 33(6): 1026-1030.

Allen JC. Initial management of children with hypothalamic and thalamic tumors and the modifying role of neurofibromatosis-1. Pediatr Neurosurg 2000;32:154-162.

Alvord EC Jr, Lofton S. Gliomas of the optic nerve or chiasm. Outcome by patients' age, tumor site, and treatment. J Neurosurg 1988; 68: 85-98.

Ammirati M, Mizai S, Samii M. Transient mutism following removal of a cerebellar tumor. Child Nerv Sys 1989; 5: 12-14.

Aquino VM, Fort DW, Kamen BA. Carboplatin for the treatment of children with newly diagnosed optic chiasm gliomas: a phase II study J Neuro-Oncology 1999; 41: 255-259.

Aristizabal S, Caldwell WL, Avila J. The relationship of time-dose fractionation factors to complications in the treatment of pituitary tumors by irradiation. Int J Radiat Oncol Biol Phys 1977; 10: 667-673.

Atkinson AB, Allen IV, Gordon DS, Hadden DR, Maguire CJ, Trimble ER, Lyons AR. Progressive visual failure in acromegaly following external pituitary irradiation. Clin Endocrinol Oxf 1979; 10: 469-479.

Austin EJ, Alvord EC. Recurrences of cerebellar astrocytomas: a violation of Collins' law. J Neurosurg 1988; 68: 41-47.

Awad IA, Rosenfeld J, Ahl J, Hahn JF, Luders H. Intractable epilepsy and structural lesions of the brain: mapping, resection strategies, and seizure outcome. Epilepsia 1991; 32: 179-186.

Bakardijiev AI, Barnes PD, Goumnerova LG, Black PMcL, Scott RM, Pomeroy SL, Billett A, Loeffler JS, Tarbell NJ. Magnetic Resonance Imaging Changes after Stereotactic Radiation Therapy for Childhood Low Grade Astrocytoma Cancer 1996; 78: 864-873.

Balestrini MR, Zanette M, Micheli R, Fornari M, Solero CL, Broggi G. Hemispheric cerebral tumors in children. Long-term prognosis concerning survival rate and quality of life - considerations on a series of 64 cases operated upon. Child Nerv Sys 1990; 6: 143-147.

Bamberg M., Hess CF., Kortmann RD. Zentralnervensystem. In: Scherer E., Sack H. (eds.) Strahlentherapie / Radiologische Onkologie 4. Heidelberg: Springer Verlag, 1998: 763-808.

Barkovich AJ, Krischer J, Kun LE, Packer RJ, Zimmerman RA, Freeman CR, Wara WM, Albright L, Allen JC, Hoffman JH. Brain stem gliomas: A classification system based on magnetic resonance imaging. Pediatr Neurosurg 1991;.16:.73-83.

Bataini JP, Delanian S, Ponvert D. Chiasmal gliomas: results of irradiation management in 57 patients and review of the literature. Int J Radiat Oncol Biol Phys 1991; 21: 615-623.

Becker G., Major J., Christ G., Duffner F., Bamberg M. Stereotaxic convergent-beam irradiation. Initial experiences with the SRS 200 system. Strahlenther Onkol 1996; 172: 9-18.

Benk V., Clark BG., Souhami L., Algan O., Bahany J., Podgorsak EB., Freeman CR. Stereotactic radiation in primary brain tumors in children and adolescents. Pediatr Neurosurg 1999; 31: 59-64.

Berg K, Grundmann U, Villena-Heinsen C, Wilhelm W, Mertzlufft F: Lebensbedrohliche Anaphylaxie nach wiederholter Cisplatin-Gabe: Fallbericht und neue Therapiekonzepte (Life threatening anaphylaxis after repeated cisplatin administration: case report and new therapy concepts). Zentralblatt f. Gynäkologie 1996; 118 (12); 684-688.

Berger MS, Baumeister B, Geyer JR, Milstein J, Kanev PM, LeRoux PD. The risks of metastases from shunting in children with primary central nervous system tumors. J Neurosurg 1991; 74: 872-877.

Berger MS, Keles E, Geyer JR. Cerebral hemispheric tumors of childhood. Pediatric Neurooncology 1992; 3:839-852.

Berger MS, Deliganis AV, Dobbins J, Keles GE. The effect of extent of resection on recurrence in patients with low grade cerebral hemispheric gliomas. Cancer 1994; 74: 1784-1791.

Berger MS. The impact of technical adjuncts in the surgical management of cerebral hemispheric low grade gliomas of childhood. J Neuro-Oncology 1996; 28: 129-155.

Bernards A. Neurofibromatosis type I and ras-mediated signaling: filling in the GAP's. Biochim Biophys Acta 1995; 1242: 43-59.

Blaney SM, Philipps PC, Packer RJ, Heideman RL, Berg SL, Adamson PC, Allen JC, Sallan SE, Jakacki RJ, Lange JB, Reaman GH, Horowitz ME, Poplack DG, Balis FM. Phase II evaluation of topotecan for pediatric central nervous system tumors. Cancer 1996; 78: 527-531

Bodey B., Bodey B Jr., Siegel SE., Kaiser HE. Fas (Apo-1, CD95) Receptor expression in childhood astrocytomas. Is it a marker of the major apoptotic pathway or a signaling receptor for immune escape of neoplastic cells? in vivo 1999; 13: 357-374.

Boos J., Real E., Schule-Westhoff P., Wolff J., Euting T., Jürgens H. Investigation of the variability of etoposide pharmacokinetics in children. Int J clin Pharmacol Ther Toxicol 1992; 30: 495-497.

Boos J., Krumpelmann S., Schulze westhoff P., Euting T., Berthold F., Jürgens H. Steady-state levels and bone marrow toxicity of etoposide in children and infants: does etoüposide require age-dependent dose calculation ? J Clin Oncol 1995; 13: 2954-2960.

Bouffet E., Amat D., Devaux Y., Desuzinges C. Chemotherapy for spinal cord astrocytoma. Med Pediatr Oncol 1997; 29: 560-562.

Bouffet E., Pierre-Kahn A., Marchal JC., Jouvet A., Kalifa C., Choux M., Dhellemmes P., Guérin J., Tremoulet M., Mottolese C. Prognostic factors in pediatric spinal cord astrocytoma. Cancer 1998; 83: 2391-2399.

Bowers DC., Georgiades C., Aronson LJ., Carson BS., Weingart JD., Wharam MD., Melhem ER., Burger PC., Cohen KJ. Tectal gliomas: natural history of an indolent lesion in pediatric patients. Pediatr Neurosurg 2000; 32: 24-29.

Braffman BH., Bilaniuk LT., Zimmerman RA. The central nervous system manifestations of the phacomatosis on MR. Radiol Clin North Am 1988; 26: 773-800.

Braun-Fischer A., Romeike BFM., Eymann R., Glas B., Riesinger P., Reiche W. Pilozytisches Astrocytom mit subarachnoidaler Dissemination. Radiologe 1997; 37: 899-904.

Brock PR., Bellman SC., Yeomans Ec., Pinkerton CR., Pritchard J. Cisplatin ototoxicity in children: a practical grading system. Med Pediatr Oncol 1991; 19: 295-300.

Brown MT., Friedman HS., Oakes J., Boyko OB., Hockenberger B., Schold SC. Chemotherapy for pilocytic astrocytoma. Cancer 1993; 71: 3165-3172.

Brown WD., Tavaré CJ., Sobel EL., Gilles FH. The applicability of Collins' law to childhood brain tumors and its usefulness as a predictor of survival. Neurosurgery 1995; 36: 1093-1096.

Bruggers CS., Friedman HS., Phillips PC., Wiener MD., Hockenberger B., Oakes WJ., Buckley EG. Leptomeningeal Dissemination of Optic Pathway Gliomas in Three Children. American Journal of Ophthalmology 1991; 111/6: 719-723.

Burger PC., Shibata T., Kleihues P. The use of the monoclonal antibody Ki 67 in the identification of proliferating cells: application to surgical neuropathology. Am J Surg Pathol 1986; 10: 611-617.

Calvert AH., Newell DR., Gumbrell LA., O'Reilly S., Burnell M., Boxall FE et al. Carboplatin dosage: prospective evaluation of a simple formula based on renal function. J Clin Oncol 1989; 7: 1748-1756.

Campbell JW., Pollack IF. Cerebellar astrocytomas in children. J Neurooncology 1996; 28: 223-231.

Cappelli C., Grill J., Raquin M., Pierre-Kahn A., Lellouch-Tubiana A., Terrier-Lacombe MJ., Habrand JL., Couanet D., Brauner R., Rodriguez D., Hartmann O., Kalifa C. Long-term follow up of 69 patients treated for optic pathway tumors before the chemotherapy era. Arch Dis Child 1998; 79: 334-338.

Cascino GD. Epilepsy and brain tumors: Implications for treatment. Epilepsia 1990; 31: S37-S44.

Castello MA., Schiavetti A., Varrasso G., Clerico A., Capelli C. Chemotherapy in low grade astrocytoma management. Child Nerv Sys 1998; 14: 6-9.

Castello MA., Schiavetti A., Padula A., Varrasso G., Properzi E., Trasimeni G., Operamolla P., Gualdi GF., Clerico A. Does chemotherapy have a role in low grade glioma management ? Med Pediatr Oncol 1995; 25: 102-108.

Cattoretti G., Becker MHG., Key G., Duchrow M., Schlueter C., Galle J., Gerdes J. Monoclonal antibodies against recombinant parts of the Ki67 antigen (MIB1 and MIB3) detect proliferating cells in microwave-processed formalin-fixed paraffin sections. J Pathol 1992: 168: 357-363.

Chadderton RD., West CGH., Schulz S., Quirke DC., Gattamaneni R., Taylor R. Radiotherapy in the treatment of low grade astrocytomas: II. The physical and cognitive sequelae. Child Nerv Sys 1995; 11: 443-448.

Chamberlain MC., Grafe MR. Recurrent chiasmatic-hypothalamic glioma treated with oral etoposide. J Clin Oncol 1995; 13: 2072-2076.

Chamberlain MC. Recurrent cerebellar gliomas: salvage therapy with oral etoposide. J child Neurol 1997; 12: 200-204.

Chan MY, Foong AP, Heisey DM, Harkness W, Hayward R, Michalski A. Potential prognostic factors of relapse-free survival in childhood optic pathway glioma: a multivariate analysis. Pediatr-Neurosurg 1998; 29: 23-28.

Chang SM. et al: Carboplatin Hypersensitivity in Children. Cancer 1995; 75; 1171-1175.

Chiesielski-Carlucci C., Leong P., Jacobs C.: Case report of anaphylaxis from cisplatin/paclitaxel and a review of their hypersensitivity reaction profiles. American J of Clinical oncology 1997; 20 (4); 373-5.

Chun HC, Schmidt-Ullrich RK, Wolfson A, Tercilla OF, Sagerman RH, King GA. External beam radiotherapy for primary spinal cord tumors. J Neurooncol 1990; 9: 211-217

Chutorian AM., Schwartz JF., Evans RA., Carter S. Optic gliomas in children. Neurology 1964; 14: 83-95.

Civitello LA., Packer RJ., Rorke LB., Siegel K., Sutton LN., Schut L. Leptomeningeal dissemination of low grade gliomas in children. Neurology 1988; 38: 562-566.

Coakley KJ., Huston J 3rd., Scheithauer BW., Forbes G., Kelly PJ. Pilocytic astrocytomas: well demarcated magnetic resonance appearance despite frequent infiltration histologically. Mayo Clin Proc 1995; 70: 747-751.

Coffey RJ, Lunsford LD. Stereotactic surgery for mass lesions of the midbrain and pons. Neurosurgery 1985; 17: 12-18.

Cohen AR., Wisoff JH., Allen JC., Epstein F. Malignant astrocytomas of the spinal cord. J Neurosurg 1989; 70: 50-54.

Constantini S., Epstein F. Intraspinal tumors in children and infants. In: Youmans JR., Becker DP., Dunsker SB., et al (eds): Neurological Surgery, ed 4. Philadelphia: WB Saunders, 1996: 3123-3133.

Constantini S., Miller D., Allen J., Rorke L., Freed D., Epstein F. Pediatric intramedullary spinal cord tumors: surgical morbidity and long-term follow-up. Child Nerv Sys 1998; 14: 484 (Meeting abstract).

Constantini S., Miller DC., Allen JC., Rorke LB., Freed D., Epstein FJ. Radical excision of intramedullary spinal cord tumors: surgical morbidity and long-term follow-up evaluation in 164 children and young adults. J Neurosurg 2000; 93: 183-193.

Cummings TJ., Provenzale JM., Hunter SB., Friedman AH., Klintworth GK., Bigner SH., McLendon RE. Gliomas of the optic nerve: histological, immunohistochemical (MIB-1 and p53), and MRI analysis. Acta Neuropathol (Berl) 2000; 99: 563-570.

Czech T., Slavc I., Aichholzer M., Haberler C., Dietrich W., Dieckmann K., Koos W., Budka H. Proliferative activity as measured by MIB-1 labeling index and long-term outcome of visual pathway astrocytomas in children. J Neurooncol 1999; 42: 143-150.

Danoff BF., Kramer S., Thompson N. The radiotherapeutic management of optic gliomas of children. Int J Radiat Oncol Biol Phys 1980;.6:.45-50.

Debus J., Kocagoncu KO., Hoss A., Wenz F., Wannenmacher M. Fractionated stereotactic radiotherapy (FSRT) for optic glioma (see comments). Int J Radiat Oncol Biol Phys 1999; 44: 243-248.

Deley MC., Raquin MA., Leblanc T. Chemotherapy, radiation dose and risk of secondary haematological malignancy (SHM) after solid tumor (ST) occuring in childhood : a case control study by the French Society of Pediatric Oncology (SFOP). Med Ped Oncol 1999;

Dhodapkar K., Wisoff J., Sanford R., Holmes E., Sposto R., Finlay J. Patterns of relapse and survival for newly-diagnosed childhood low grade astrocytoma: Initial reults of CCG9891/POG 9130. Med Pediatr Oncol 1999; 33: 205 (Meeting abstract).

Dirven CMF., Mooij JJA., Molenaar WM. Cerebellar pilocytic astrocytoma: a treatment protocol based upon analysis of 73 cases and review of the literature. Chilc Nerv Sys 1997; 13: 17-23.

Dirven CMF., Kondstaal J., Mooij JJA., Molenaar WM. The proliferative potential of the pilocytic astrocytoma: the relation between MIB-1 labeling and clinical and neuro-radiological follow-up. J Neuro-oncol 1998; 37: 9-16.

Dodge HW., Lowe JG., Craig WM., et al. Gliomas of the optic nerves. Archives of Neurology and Psychiatry 1958; 79: 607-621.

Doireau V., Grill J., Chastagner P., Zerah M., Terrier-Lacombe MJ., Couanet D., Raquin M., Kalifa C. Chemotherapy for intramedullary glial tumors. CNS 1998; 14: 484-485 (Meeting abstract).

Doireau V., Grill J., Zerah M., Lellouch-Tubiana A., Couanet D., Chastagner P., Marchal JC., Grignon Y., Chouffai Z., Kalifa C. Chemotherapy for unresectable and recurrent intramedullary glial tumors in children. Brain tumors subcommittee of the french society of pediatric oncology (SFOP). Br. J Cancer 1999; 81: 835-840.

Dosoretz DE., Blitzer PH., Wang CC., Linggood RM. Management of glioma of the optic nerve and/or chiasm: an analysis of 20 cases. Cancer 1980; 45: 1467-1471.

Due-Tonnessen BJ et al.: Long term outcome after resection of benign cerebellar astrocytomas in children and young adults (0-19 years). Report of 110 consecutive cases. Pediatr Neurosurg 2002; 37: 71-80.

Dunbar SF., Tarbell NJ., Kooy HM., Alexander E-3., Black PM., Barnes PD., Goumnerova L., Scott M., Pomeroy SL., La Vally B., Sallan SE., Loeffler JS. Stereotactic radiotherapy for pediatric and adult brain tumors: preliminary report. Int J Radiat Oncol Biol Phys 1994; 30: 531-539.

Dutton JJ. Gliomas of the anterior visual pathway. Surv Ophthalmol 1994; 38: 427-452.

Edwards MSB., Wara WM., Urtasun RC., Prados M., Levin VA., Fulton D., Wilson CB., Hannigan J., Silver P. Hyperfractionated radiation therapy for brainstem gliomas: a phase I-II trial. J Neurol 1989; 64: 11-14.

Edwards MS., Wara WM., Ciricillo SF., Barkovich AJ. Focal brain-stem astrocytomas causing symptoms of involvement of the facial nerve nucleus: long-term survival in six pediatric cases. J Neurosurg 1994; 80: 20-25.

Eggers H., Jokobiec FA., Jones IS. Optic nerve gliomas. In: Duane TD., Jaeger EA. (eds.) Clinical Ophthalmology, volume 2. New York: Harper and Row, 1985: 1-17.

Epstein F.J., Epstein N. Surgical treatment of spinal cord astrocytomas of childhood. A series of 19 patients. J Neurosurg 1982; 57: 685-689.

Epstein F., McCleary El. Intrinsic brain-stem tumors of childhood: surgical indications. J Neurosurg 1986; 64: 11-14.

Epstein F., Farmer JP., Freed D. Adult intramedullary astrocytomas of the spinal cord. J Neurosurg 1992; 77: 355-359.

Epstein F., Constantini S. Spinal cord tumors of childhood. In: Pang D (ed.): Disorders of the Pediatric Spine. New York: Raven Press, 1994: 55-76.

Epstein FJ. Spinal cord tumors in children. J Neurosurg 1995; 82: 516-517 (Letter).

Erkal HS., Serin M., Cakmak A. Management of optic pathway and chiasmatic-hypothalamic gliomas in children: tumor volume response to radiation therapy. Radiother Oncol 1997; 45: 11-15.

Farwell J.R., Dohrmann GJ., Flannery JT. Central nervous system tumors in Children. Cancer 1977; 40: 3123-3132.

Feeny D, Furlong W, Boyle M, Torrance GW. Multi-attribute health status classification systems: Health Utilities Index. Pharmacoeconomics 1995;7:490-502.

Fisher BJ., Bauman GS., Leighton CE., Stitt L., Cairncross JG., Macdonald DR. low grade gliomas in children: tumor volume response to radiation. J Neurosurg 1998; 88: 969-974.

Fisher BJ, Leighton CC, Vujovic O, Macdonald DR, Stitt L. Results of a policy of surveillance alone after surgical management of pediatric low grade gliomas. Int J Radiat Oncol Biol Phys 2001; 51: 704-710

Fletcher WA., Imes RK., Hoyt WF. Chiasmatic gliomas: appearance and long-term changes demonstrated by computed tomography. J Neurosurg 1986; 65: 154-159.

Flickinger JC., Torres C., Deutsch M. Management of low grade gliomas of the optic nerve and chiasm. Cancer 1988; 61: 635-642.

Foreman NK., Hay T.C., Handler M. Chemotherapy for spinal cord astrocytoma. Med Pediatr Oncol 1998; 30: 311-312 (Letter).

Forsyth PA., Shaw EG., Scheithauer BW., O'Fallon JR., Layton DD Jr. Katzman JA. Supratentorial pilocytic astrocytomas. A clinicopathologic, prognostic, and flow cytometric study of 51 patients. Cancer 1993; 72: 1335-1342.

Fort DW., Packer RJ., Kirkpatrick GB., Kuttesch JF Jr., Ater JL. Carboplatin and vincristine for pediatric primary spinal cord astrocytomas. Child Nerv Sys 1998; 14: 484 (Meeting abstract).

Fouladi M., Jones-Wallace D., Langston JW., Mulhern R., Gajjar A., Sanford RA., Merchant E., Jenkins JJ., Kun LE., Heideman L. Long-term survival and funtional outcome of children with hypothalamic/chiasmatic (H/C) tumors. Proceedings of ASCO, Orlando, Florida, May 18-21, 2002; Abstract 1575, page 394.

Franzini A., Allegranza A., Melcarne A., Giorgi C., Ferrarsci S., Broggi G. Serial stereotactic biopsy of brain stem expanding lesions. Consideration on 45 consecutive cases. Acta Neurochir [Suppl] (Vienna) 1988; 42: 170-176.

Freeman CR., Krischner JP., Sanford A., Cohen ME., Burger PC., del Carpio R., Halperin EC., Munoz L., Friedman HS., Kun LE. Final results of a study of escalating doses of hyperfractionated radiotherapy in brain stem tumors in children: a Pediatric Oncology Group study. Int J Rad Oncol Biol Phys 1993; 27: 197-206.

Friedman HS., Krischer JP., Burger P., Oakes WJ., Hockenberger B., Weiner MD., Falletta JM., Norris D., Ragab AH., Mahoney DH., Whitehead MV., Kun LE. Treatment of children with progressive or recurrent brain tumors with carboplatin or iproplatin: a Pediatric Oncology Group randomized phase II study. J Clin Oncol 1992; 10: 249-256.

Friedman JM., Birch P. An association between optic glioma and other tumors of the central nervous system in neurofibromatosis type I. Neuropediatrics 1997; 28: 131-132.

Furuya Y, Uemura K, Ryu H, Nakajima S, Sato K, Yokoyama T, Kaneko M. Optic glioma decreasing in size after irradiation. J Child Neurol 1986; 1: 173-175

Gajjar A., Bhargava R., Jenkins JJ., Heideman R., Sanford RA., Langston JW., Walter AW., Kuttesch JF., Muhlbauer M., Kun LE. low grade astrocytoma with neuroaxis dissemination at diagnosis. J Neurosurg 1995; 83: 67-71.

Gajjar A., Sanford RA., Heideman R., Jenkins JJ., Walter A., Li Y., Langston JW., Muhlbauer M., Boyett JM., Kun LE. low grade astrocytoma: A decade of experience at St. Jude Children's Research Hospital. J Clin Oncol 1997; 15: 2792-2799.

Ganz JC., Smievoll AI., Thorsen F. Radiosurgical treatment of gliomas of the diencephalon. Acta Neurochir Suppl (Wien) 1994; 62: 62-66.

Garcia DM, Fulling KH, Marks JE. The value of radiation therapy in addition to surgery for astrocytomas of the adult cerebrum. Cancer 1985; 55: 919-927

Garcia DM., Latifi HR., Simpson JR., Picker S. Astrocytomas of the cerebellum in children. J Neurosurg 1989; 71: 661-664.

Garcia DM., Marks JE., Latifi HR., Kliefoth AB. Childhood cerebellar astrocytomas: is there a role for postoperative irradiation? Int J Radiation Oncology Biol. Phys 1990; 18: 815-818.

Garré ML., Perilongo G., Zanetti I., Walker D., Scarzello G., Gnekow A. Optic pathways gliomas (OPG) in children with Neurofibromatosis type NF I: Natural history and results of treatment (TX). The experience of the SIOP low grade glioma study. Proceedings of the 10th International Symposium on Pediatric Neurooncology, London, 2002 (Meeting abstract)

Garvey M., Packer RJ. An integrated approach to the treatment of chiasmatic-hypothalamic gliomas. J Neuro-Oncology 1996; 28: 167-183.

Ghim T. Efficacy of Combination Chemotherapy in Children with Recurrent low grade Astrocytoma. Proc Annu Meet Am Soc Clin Oncol 1993; 12: A1444 (Meeting Abstract)

Gjerris F., Klinken L. Long term prognosis in children with benign cerebellar astrocytoma. J Neurosurg 1978; 49: 179-184.

Gjerris F, Harmsen A, Klinken L, Reske-Nielsen E Incidence and long term survival of children with intracranial tumors treated in Denmark 1935-1959. Br J Cancer 1978; 38: 442-451

Glaser JS., Hoyt WF., Corbett J. Visual morbidity with chiasmal glioma. Arch Ophthalmol 1971; 85: 3-12.

Glaser A, Kennedy CR, Punt J, Walker DA. A standardised strategy for qualitative assessment of brain tumor survivors treated within clinical trials in childhood. Int J Can 1999; S12:77-82.

Glauser TA., Packer RJ. Cognitive deficits in long term survivors of childhood brain tumors. Child NervSys 1991; 7: 2-12.

Gnekow AK. Recommendations of the brain tumor subcommittee for the reporting of trials. Med Pediatr Oncol 1995; 24: 104-108.

Gnekow AK., Kaatsch P., Kortman R., Wiestler OD. HIT-LGG: effectiveness of carboplatin-vincristine in progressive low grade gliomas of childhood - an interim report. Klin Padiatr 2000; 212: 177-184.

Gol, A. Cerebral astrocytomas in childhood: A clinical study. J Neurosurg 1962; 19: 577-582.

Goldberg A., Altaras MM., Mekori YA., Beyth Y., Confino-Cohen R: Anaphylaxis to cisplatin: diagnosis and value of pretreatment in prevention of recurrent allergic reactions. Annals of Allergy 1994; 78 (3); 271-2.

Goodman R. The Strengths And Difficulties Questionnaire: a research note. J Child Psychol Psychiatr 1994;38:581-586.

Gould RJ, Hilal SK, Chutorian AM. Efficacy of radiotherapy in optic gliomas. Pediatr Neurol 1987; 3: 29-32

Grabb PA., Lunsford LD.; Albright AL.; Kondziolka D.; Flickinger J.C. Stereotactic Radiosurgery for Glial Neoplasms of Childhood. Neurosurgery 1996; 38 (4): 696-702.

Grabenbauer GG., Schuchardt U., Buchfelder M., Roedel CM., Gusek G., Marx M., Doerr HG., Fahlbusch R., Huk WJ., Wenzel D., Sauer R. Radiation therapy of optico-hypothalamic gliomas (OHG) - radiographic response, vision and late toxicity. Radiother Oncol 2000; 54: 239-245.

Grabenbauer GG, Roedel CM, Paulus W, Ganslandt O, Schuchardt U, Buchfelder M, Schrell U, Fahlbusch R, Huk WJ, Sauer R. Supratentorial low grade glioma: results and prognostic factors following postoperative radiotherapy. Strahlenther Onkol 2000a; 176: 259-264

Griffin TW., Beaufait D., Blasko JC. Cystic cerebellar astrocytomas in childhood. Cancer 1979; 44: 276-280.

Grill J., Laithier V., Rodriguez D., Raquin MA., Pierre-Kahn A., Kalifa C. When do children with optic pathway tumors need treatment ? An oncological perspective in 106 patients treated in a single centre. Eur J Peediatr 2000; 159: 692-696.

Grill J., Couanet D., Capelli C., Habrand JL., Rodriguez D., Sainte-Rose C., Kalifa C. Radiation induced cerebral vasculopathy in children with neurofibromatosis and optic pathway glioma. Ann Neurol 1999; 45: 393-396.

Gropman AL, Packer RJ, Nicholson HS, Vezina LG, Jakacki R, Geyer R, Olson JM, Phillips P, Needle m, Broxson EH jr, Reaman G, Finlay J. Treatment of diencephalic syndrome with chemotherapy: growth, tumor response, and long term control. Cancer 1998, 83: 166-72.

Gururangan S., Cavazos CM., Ashley D., Herndon JE 2nd, Bruggers CS., Moghrabi A., Scarcella DL., Watral M., Tourt-Uhlig s., Reardon D., Friedman HS. Phase II study of carboplatin in children with progressive low grade gliomas. J Clin Oncol 2002; 20: 2951-2958.

Habrand JL., Crevoisier R de. Radiation therapy in the management of childhood brain tumors. Child's Nerv Syst 2001; 17: 121-133.

Hardison HH., Packer RJ., Rorke LB., Schut L., Sutton LN., Bruce DA. Outcome of children with primary intramedullary spinal cord tumors. Childs Nerv Syst 1987; 3: 89-92.

Harisiadis L, Chang CH: Medulloblastoma in children: A correlation between staging and results of treatment. Int J Radiat Oncol Biol Phys 1977; 2: 833-

Harris JR., Levene MB. Visual complications following irradiation for pituitary adenomas and craniopharyngiomas. Radiology 1976; 120: 167-171.

Hawkins MM., Wilson LM., Stovall MA., et al. Epipodophyllotoxins, alkylating agents and radiation and risk of secondary leukemia after childhood cancer. BMJ 1992; 304: 951-958.

Hayostek CJ., Shaw EG., Scheithauer B., O'Fallon JR., Weiland TL., Schomberg PJ., Kelly PJ., Hu TC. Astrocytomas of the cerebellum: a comparative clinicopathologic study of pilocytic and diffuse astrocytomas. Cancer 1993; 72: 856-869.

Heideman RL., Douglass EC., Langston JA., Krischer JP., Burger PC., Kovnar EH., Kun LE., Friedman HS., Kadota R. A phase II study of every other day high-dose ifosfamide in pediatric brain tumors: a Pediatric Oncology Group study. J Neurooncol 1995; 25: 77-84.

Hirsch J-F., Rose CS., Pierre-Kahn A., Pfister A., Hoppe-Hirsch E. Benign astrocytic and oligodendrocytic tumors of the cerebral hemispheres in children. J Neurosurg 1989; 70: 568-572.

Hoffman HJ., Soloniuk DS., Humphreys RP., Drake JM., Becker LE., de Lima BO., Piatt, JH Jr. Management and outcome of low grade astrocytomas of the midline in children: A retrospective review. Neurosurgery 1993; 33: 964-971.

Holdener EE et al: Cancer Res 96: 188-196, 1994.

Horwich A., Bloom HJ. Optic gliomas: radiation therapy and prognosis. Int J Radiat Oncol Biol Phys 1985; 11: 1067-1079.

Hoshi, M., Yoshida K., Shimazaki K., Sasaki H., Otani M., Kawase T. Correlation between MIB 1-staining indices and recurrence in low grade astrocytomas. Brain Tumor Pathol 1997; 14: 47-51.

Hoyt WF, Baghdassarian SA. Optic glioma of childhood. Natural history and rationale for conservative management. Br J Ophthalmol 1969; 53: 793-798.

Hug EB, Muenter MW, Archambeau JO, DeVries A, Liwnicz B, Loredo LN, Grove RI, Slater JD. Conformal proton radiation therapy for pediatric low grade astrocytoma. Strahlentherapie und Onkologie 2002, 178: 10-7.

Huson SM. Neurofibromatosis 1: a clinical and genetic overview. In: Huson SM., Hughes RAC. (eds.) The Neurofibromatoses. London: Chapman Hall Medical, 1994: 160-203.

Huson SM., Upadhyaya M. Neurofibromatosis 1: clinical management and genetic counselling. In: Huson SM., Hughes RAC. (eds.) The Neurofibromatoses. London: Chapman Hall Medical, 1994: 355-381.

Ishii N., Tada M., Hamou MF., Janzer RC., Meagher-Villemure K., Wiestler OD., Tribolet N., Van Meir EG. Cells with TP53 mutations in low grade astrocytic tumors evolve clonally to malignancy and are an unfavorable prognostic factor. Oncogene 1999; 18: 5870-5878.

Jakobi G., Kornhuber B. Malignant brain tumors in children. In: Jellinger K. (ed.) Therapy of malignant brain tumors. Wien: Springer, 1987: 396-493.

Janss A., Hiehle JF., Yachnis AT. Neurofibromatosis type 1. Med Pediatr Oncol 1995; 25: 213-222.

Janss AJ., Grundy R., Cnaan A., Savina PJ., Packer RJ., Zackai EH., Goldwein JW., Sutton LN., Radcliffe J., Molloy PT., Phillips PC., Lange BJ. Optic pathway and hypothalamic/chiasmatic gliomas in children younger than age 5 years with a 6-year follow-up. Cancer 1995; 75: 1051-1059.

Jenkin D., Angyalfi S., Becker L., Berry M., Bunice R., Chan H., Doherty M., Drake J., Greenberg M., Hendrick B., Hoffman H., Humphreys R., Weitzman S. Optic glioma in children: Surveillance, Resection, or irradiation ? Int J Radiation Oncology Biol Phys 1993; 25: 215-225.

Jenkin RD., Boesel C., Ertel E., Evans A., Hittle R., Ortega J., Sposto R., Wara W., Wilson C., Anderson J. et al. Brain-stem tumors in childhood: a prospective randomized trial of irradiation with and without adjuvant CCNU, VCR, and prednisone. A report of the Children's Cancer Study Group. J Neurosurg 1987; 66: 227-233.

Jennison C., Turnbull BW. Group sequential methods with applications to clinical trials. Chapman & Hall / CRC (2000) chapter 5.2.

Jeremic B., Shibamotu Y., Grujicic D., Milicic B., Stojanovic M., Nikolic N., Dagovic A. Hyperfractionated radiation therapy for incompletely resected supratentorial low grade glioma. A. phase II study. Radiotherapy and Oncology 1998; 49: 49-54.

Johnson JH., Hariharan S., Berman J., Sutton LN., Rorke LB., Molloy P., Phillips PC. Clinical outcome of pediatric gangliogliomas: Ninety-nine cases over 20 years. Pediatr Neurosurg 1997; 27: 203-207.

Józwiak S., Schwartz RA., Janniger CK., Bielicka-Cymerman J. Usefulness of diagnostic criteria of tuberous sclerosis complex in pediatric patients. J Child Neurol 2000; 15: 652-659.

Jyothirmayi R., Madhavan J., Nair MK., Rajan B. Conservative surgery and radiotherapy in the treatment of spinal cord astrocytoma. J Neurooncol 1997; 33: 205-211.

Kaatsch P., Rickert CH., Kühl J., Schütz J., Michaelis J. Population-based epidemiologic data on brain tumors in German children. Cancer 2001; 92: 3155-3164.

Kadota RP., Kun LE., Langston JW., Burger PC., Cohen ME., Mahoney DH., Walter AW., Rodman JH., Parent A., Buckley E., Kepner JL., Friedman HS. Cyclophosphamide for the treatment of progressive low grade-astrocytoma: A pediatric oncology group phase II study. J Pediatr Hematology/Oncology 1999; 21: 198-202.

Kadota RP., Stewart CF., Horn M., Kuttesch JF Jr., Burger PC., Kepner J., Kun LE., Friedman HS., Heideman RL. Topotecan for the treatment of progressive central nervous system tumors - a pediatric oncology group phase II study. J Neurooncol 1999; 43: 43-47.

Kadota RP, Mandell LR., Fontanesi L., Kovnar EH., Krischer J., Kun LE., Friedman HS. Hyperfractionated irradiation and concurrent cisplatin in brain stem tumors: a Pediatric Oncology Group pilot study 9139. Pediatr Neurosurg 1994; 20: 221-225.

Kalifa C., Ernest C., Rodary C., Sarrazin D., Bloch, Michel E., Lemerle J. [Optic glioma in children. A retrospective study of 57 cases treated by irradiation (authors' translation)]. Arch Fr Pediatr 1981; 38: 309-313.

Karim AB., Maat B., Hatlevoll R., Menten J., Rutten EH., Thomas DG., Mascarenhas F., Horiot JC., Parvinen LM., van Reijn M., Jager JJ., Fabrini MG., van Alphen AM., Hamers HP., Gaspar L., Noordman E., Pierart M., van Glabbeke M. A randomized trial of dose-response in radiation therapy of low grade cerebral glioma: European Organisation for Research and Treatment of Cancer (EORTC) Study 22844. Int J Radiat Oncol Biol Phys 1996; 36: 263-270.

Karim AB, Afra D, Cornu P, Bleehan N, Schraub S, De Witte O, Darcel F, Stenning S, Pierart M, van Glabbeke M. Randomized trial on the efficacy of radiotherapy for cerebral low grade glioma in the adult: European

Organization for Research and Treatment of Cancer Study 22845 with the Medical Research Council study BRO4: an interim analysis. Int J Radiat Oncol Biol Phys 2002; 52: 316-324

Kazner E., Wende S., Grumme T., Stochdorph O., Felix R., Claussen C. Computed tomography and magnetic resonance tomography of intracranial tumors: a clinical perspective. Berlin: Springer, 1989.

Kernan J.C., Horgan MA., Piatt JH., D'Agostino A. Spontaneous involution of a diencephalic astrocytoma. Pediatr. Neurosurgery 1998; 29: 149-153.

Kim JH., Guimaraes PO., Shen MY., Masukawa L-M., Spencer DD. Hippocampal neuronal density in temporal lobe epilepsy with and without gliomas. Acta Neuropathol 1990; 80: 41-45.

Kleihues P., Cavenee WK. Pathology and genetics of tumors of the nervous system. Lyon: International Agency for Research on Cancer (IARC) Press, 2000.

Kocks W., Kalff R., Reinhardt V., Grote W., Hilke J. Spinal metastasis of pilocytic astrocytoma of the chiasma opticum. Child Nerv Syst 1989; 5: 118-120.

Kortmann RD., Hess CF., Jany R., Bamberg M. Repeated CT-examinations in limited volume irradiation of brain tumors: quantitative analysis of individualized (CT-based) treatment plans. Radiotherapy and Oncology 1994; 30: 171-174.

Kortmann RD., Timmermann B., Becker G., Kuehl J, Bamberg M. Advances in treatment techniques and time/dose schedules in external radiation therapy of brain tumors in childhood. Klin Pediatr 1998; 210: 220-226.

Kortmann RD., Becker G., Perelmouter J., Buchgeister M., Meisner C., Bamberg M. Geometric accuracy of field alignment in fractionated stereotactic conformal radiotherapy of brain tumors. Int J Radiat Oncol Biol Phys 1999; 43: 921-926.

Kortmann RD., Zanetti I., Mueller S., Taylor RE., Scarzello G., Perilongo G., Walker DA., Gnekow AK., Garré ML. Radiotherapy in low grade glioma: an interim analysis of the SIOP low grade glioma study. IXth Symposium Pediatric Neuro-Oncology 2000 (meeting abstract).

Kortmann RD, Jeremic B, Bamberg M (2000a) Radiotherapy in the management of low grade glioma. In Combined modality therapy for central nervous system tumors, Petrowich ZBLWAML (ed) Springer: Berlin, Heidelberg, New York 2000a; pp 317-326.

Kotagal S. Increased intracranial pressure. In: Swaiman KF., Ashwal S. (eds.) Pediatric Neurology. St. Louis: Mosby, 1999: 945-953.

Kovalic JJ., Grigsby PW., Shepard MJ., Fineberg BB., Thomas PR. Radiation therapy for gliomas of the optic nerve and chiasm. Int J Radiat Oncol Biol Phys 1990; 18: 927-932.

Kreth FW, Faist M, Warnke PC, Rosner R, Volk B, Ostertag CB. Interstitial radiosurgery of low grade gliomas. J Neurosurg 1995; 82: 418-429.

Kretschmar CS., Tarbell NJ., Barnes PD., Krischer JP., Burger PC., Kun L. Pre-irradiation chemotherapy and hyperfractionated radiation therapy 66 Gy for children with brain stem tumors: a phase II study of the Pediatric Oncology Group, protocol 8833. Cancer 1993; 72: 1404-1413.

Kushner et al: Proceedings of ASCO 17: 3041, 1998.

Lacaze E., Kieffer V., Streri A., Gentaz E., Kalifa C., Hartmann O., Grill J. Neuropsychological outcome of children with optic pathway tumors treated with BBSFOP chemotherapy as first line treatment. (in preparation)

Laithier V., Raquin MA., Couanet D., Doz F., Gentet JC., Frappaz D., Chastagner P., Lellouch-Tubiana A., Kalifa C., for the SFOP. Chemotherapy for children with optic pathway glioma: results of a prospective study by the French society of Pediatric Oncology (SFOP). Med Ped Oncol 2000; 35: 190 (Meeting abstract).

Landgraf JM, Abetz L, Ware JE. The CHQ user's manual. 2000, Boston,MA: Health Act.

Lavery MA., O'Neil JF., Chu FC., Martyn LJ. Acquired nystagmus in early childhood: a presenting sign of intracranial tumor. Ophthalmology 1984;91:425-435.

Lee TC., Hook CC., Long HJ.: Severe exfoliative dermatitis associated with hand ischemia during cisplatin therapy. Mayo Clinic Proceedings 1994; 69 (1); 80-82.

Lee RR. MR imaging of intradural tumors of the cervical spine. Magn Reson Imaging Clin N Am 2000; 8: 529-540.

Lesage F., Grill J., Cinalli G., Lellouch-Tubiana A., Cuanet, Kalifa C. Metastatic low grade glioma in 16 children: presentation, treatment and outcome. Child Nerv Syst 1998; 14: 483.

Lewis RA., Gerson LP., Axelson KA., Riccardi VM., Whitford RP. Von Recklinghausen Neurofibromatosis. II. Incidence of optic gliomata. Ophthalmology 1984; 91: 929-935.

Li FP., Fraumeni JF Jr. Prospective study of a familiy cancer syndrome. JAMA 1982; 247: 2692-2694.

Lim YJ., Leem W. Two cases of Gamma Knife radiosurgery for low grade optic chiasm glioma. Stereotact Funct Neurosurg 1996; 66 Suppl 1: 174-183.

Linstadt DE., Wara WM., Leibel SA., Gutin PH., Wilson CB., Sheline GE. Postoperative radiotherapy of primary spinal cord tumors. Int J Radiation Oncol Biol Phys 1989; 16: 1397-1403.

Lisch K. Ueber Beteiligung der Augen, insbesondere das Vorkommen von Irisknötchen bei der Neurofibromatose (Recklinghausen). Z Augenheilkd 1937; 93: 137-143.

Listernick R., Charrow J., Greenwald MJ., Esterly NB. Optic gliomas in children with neurofibromatosis type 1. J Pediatr 1989; 114: 788-792.

Listernik R., Charrow J., Greenwald M., Mets M. Natural history of optic pathway tumors in children with neurofibromatosis type 1: A longitudinal study. J Pediatr 1994; 25: 63-66.

Listernick R., Darling C., Greenwald M., Strauss L., Charrow J. Optic pathway tumors in children: the effect of neurofibromatosis type 1 on clinical manifestations and natural history. J Pediatr 1995; 127: 718-722.

Listernick R., Louis DN., Packer RJ., Gutmann D.H. Optic pathway gliomas in children with neurofibromatosis 1: consensus statement from the NF I optic pathway glioma task force. Annals of Neurology 1997; 41: 143-149.

Longee DC., Friedman HS., Albright RE., Burger PC., Oakes WJ., Moore JO., Schold SC. Treatment of patients with recurrent gliomas with cyclophosphamide and vincristine. J Neurosurg 1990; 72: 583-588.

Longee D. Activity of High-dose Cyclophosphamide in the Treatment of Disseminated Juvenile Pilocytic Astrocytoma (Meeting Abstract) 7th International Symposium on Pediatric Neuro-Oncology, Washington, 1996

Lorenz M., Graf N., König J., Ruprecht KW., Käsmann-Kellner B. Augenbefunde bei Kindern mit Hirntumor – Datenbasis für einen Nachsorgeplan. (Eye findings in pediatric brain tumor – data basis for a follow-up proposal) Klein Pädiatr 2002; 214: 117-125.

Louis DN., Stemmer-Raichamimov AO., Wiestler OD. Neurofibromatosis type 2. In: Kleihues P., Cavenee WK (eds.) Pathology and genetics: tumors of the nervous system. Lyon: IARC Press, 2000: 231-234.

Lowis SP., Pizer BL., Coakham H., Nelson RJ., Bouffet E. Chemotherapy for spinal cord astrocytoma: can natural history be modified? Child Nerv Syst 1998; 14: 317-321.

Lubs MLE., Bauer MS., Formas ME., Djokic B. Lisch nodules in neurofibromatosis type 1. NEJM 1991; 324: 1264-1266.

Ludwig C.L., Smith MT., Godfrey AD. A clinico-pathological study of oligodendrogliomas. Ann Neurol 1986; 19: 15-21.

Lynch HT., Katz DA., Bogard PJ., Lynch JF. The sarcoma, breast cancer, lung cancer, and adrenocortical carcinoma syndrome revisited: childhood cancer. Am J Dis Child 1985; 139: 134-136.

Malkin D., Li FP., Strong LC., Fraumeni JF Jr., Nelson CE., Kim DH., Kassel J., Magdalena AG., Bischoff FZ., Tainsky MA., Friend SH. Germ line p53 mutations in a familial syndrome of breast cancer, sarcomas, and other neoplasms. Science 1990; 250: 1233-1238.

Mamelak AN., Prados MD., Obana WG., Cogan PH., Edwards MSB. Treatment Options and Prognosis for Multicentric Juvenile Pilocytic Astrocytoma. J Neurosurg 1994; 81: 24-30.

Mansur DB., Hekmatphanah J., Wollman R., Macdonald L., Nicholas K., Beckmann E., Mundt AJ. Low grade gliomas treated with adjuvant radiation therapy in the modern imaging era. Am J Clin Oncol 2000; 23: 222-226.

Marcus RB Jr., Million RR. The incidence of myelitis after irradiation of the cervical spinal cord. Int J Radiation Onco Biol Phys 1990; 19: 3-8.

Mathew P., Look T., Luo X., Ashmun R., Nash M., Gajjar A., Walter A., Kun L., Heideman RL. DNA Index of glial tumors in children. Correlation with tumor grade and prognosis. Cancer 1996; 78: 881-886.

McCormick PC., Torres R., Post KD., Stein BM. Intramedullary ependymoma of the spinal cord. J Neurosurg 1990; 72: 523-532.

McCowage GR., Longee D., Fuchs H., Friedman HS. Treatment of High-grade Gliomas and Metastatic Pilocytic Astrocytomas with High-dose Cyclophosphamide. Proc Annu Meet Am Soc Clin Oncol 1995; 14: A290 (Meeting Abstract).

McCowage G., Tien R., McLendon R., Felsberg G., Fuchs H., Graham M.L., Kurtzberg J., Moghrabi A., Ferrell L., Kerby T., Duncan-Brown M., Stewart E., Robertson P.L., Colvin O.M., Golembe B., Bigner D.D., Friedman H.S. Successful Treatment of Childhood Pilocytic Astrocytomas Metastatic to the Leptomeninges With High-Dose Cyclophosphamide. Med Pediatr Oncol 1996; 27: 32-39.

McCunniff AJ., Liang MG. Radiation tolerance of the cervical spinal cord. Int J Radiat Oncol Biol Phys 1989; 16: 675-678.

Medlock MD, Scott RM. Optic chiasm astrocytomas of childhood. 2. Surgical management. Pediatr Neurosurg 1997; 27: 129-136

Merchant TE., Kiehna EN., Thompson SJ., Heidman RL., Sanford RA., Kun LE. Pediatric low grade and ependymal spinal cord tumors. Pediatr Neurosurg 2000; 32: 30-36.

Merchant TE, Goloubeva O, Pritchard DL, Gaber MW, Xiong X, Danish RK, Lustig RH. Radiation dose-volume effects on growth hormone secretion. Int J Radiat Oncol Biol Phys 2002a; 52: 1264-1270

Merchant TE, Zhu Y, Thompson SJ, Sontag MR, Heideman RL, Kun LE. Preliminary results from a Phase II trail of conformal radiation therapy for pediatric patients with localised low grade astrocytoma and ependymoma. Int J Radiat Oncol Biol Phys 2002b; 52: 325-332

Mercuri S., Russo A., Palma L. Hemispheric supratentorial astrocytomas in children. Long term results in 29 cases. J Neurosurg 1981; 55: 170-173.

Michaelis J., Kaletsch U., Kaatsch P. Epidemiology of childhood brain tumors. Zentralbl Neurochir 2000; 61: 80-87.

Miettinen H., Kononen J., Sallinen P., Alho H., Helen P., Helin H., Kalimo H., Paljaervi L., Isola J., Haapasalo H. CDKN2/p16 predicts survival in oligodendrogliomas: comparison with astrocytomas. J Neuro-Oncol 1999; 41: 205-211.

Milstein JM., Geyer JR., Berger MS., Bleyer WA. Favorable prognosis for brainstem gliomas in neurofibromatosis. J Neuro-Oncol 1989;7(4):367-371.

Minehan KJ., Shaw EG., Scheithauer BW., Davis DL., Onofrio BM. Spinal cord astrocytoma: pathological and treatment considerations. J Neurosurg 1995; 83: 590-595.

Mishima K., Nakamura M., Nakamura H., Nakamura O., Funata N., Shitara N. Leptomeningeal dissemination of cerebellar pilocytic astrocytoma. Case report. J Neurosurg 1992; 77: 788-791.

Mitchell AE., Elder JE., Mackey DA., Waters KD., Ashley DM. Visual improvement despite radiologically stable disease after treatment with carboplatin in children with progressive low grade optic/thalamic gliomas. J Pediatr Hematol Oncol 2001; 23: 572-577.

Moghrabi A., Friedman HS., Burger PC., Tien R., Oakes WJ. Carboplatin treatment of progressive optic pathway gliomas to delay radiotherapy. J Neurosurg 1993; 79: 223-227.

Molenkamp G., Riemann B., Kuwert T., Strater R., Kurlemann G., Schober O., Jurgens H., Wolff JE. Monitoring tumor activity in low grade glioma of childhood. Klin Padiatr 1998; 210: 239-242.

Montgomery AB., Griffin T., Parker RG., Gerdes AJ. Optic nerve glioma: the role of radiation therapy. Cancer 1977; 40: 2079-2080.

Morota N., Sakamoto K., Kobayashi N., Hashimoto K. Recurrent low grade glioma in children with special reference to computed tomography findings and pathological changes. Child Nerv Syst 1990; 6: 155-160.

Mulne AF., Ducore JM., Elterman RD., Friedman HS., Krischer JP., Kun LE., Shuster JJ., Kadota RP. Oral methotrexate for recurrent brain tumors in children: a Pediatric Oncology Group study. J Pediatr Hematol Oncol 2000; 22: 41-44.

National Institutes of Health Consensus Development Conference. Neurofibromatosis: conference statement. Arch Neurol 1988; 45: 575-578.

Nishio S., Morioka T., Fujii K., Inamura T., Fukui M. Spinal cord gliomas: management and outcome with reference to adjuvant therapy. J Clin Neurosci 2000: 7: 20-23.

nQuery Advisor® Release 3.0, Statistical Solutions Ltd., Cork, Ireland.

Obana WG., Cogen PH., Davis RL., Edwards MSB. Metastatic juvenile pilocytic astrocytoma. J Neurosurg 1991; 75: 972-975.

O'Brien PC, Fleming TR. A multiple testing procedure for clinical trials. Biometrics 35; 1979: 549-556.

Ohgaki K., Schauble B., zur Hausen A., von Ammon K., Kleihues P. Genetic alterations associated with the evolution and progression of astrocytic brain tumors. Virchows Arch 1995; 427: 113-118.

Ohgaki K. Vital A., Kleihues P., Hainaut P. Li Fraumeni syndrome and TP53 germline mutations. In: Kleihues P., Cavenee WK. (eds.) Pathology and genetics: Tumors of the nervous system. Lyon: IARC Press, 2000: 231-234.

Onoyama Y., Umezu T., Kuriaki Y., Honda N.: Hypersensitivity reactions to cisplatin following multiple uncomplicated courses: A report on two cases. J of Obstetrics and Gynaecology Research 1997; 23 (4); 347-352.

Orr, L.C., Fleitz J, McGavran L, Wyarr-Ashmead J, Handler M, Foreman NK. Cytogenetics in pediatric low grade astrocytomas. Med Pediatr Oncol 2002; 38: 173-177.

Ostertag Ch.B.: Stereotactic interstitial radiotherapy for brain tumors. J of Neurosurg Sciences 1989; 33, 1: 83-89.

O'Sullivan C., Jenkin D., Doherty MA., Hoffman HJ., Greenberg M. Spinal cord tumors in children: long-term results of combined surgical and radiation treatment. J Neurosurg 1994; 81: 507-512.

Packer RJ., Bilaniuk LT., Cohen BH., Braffman BH., Obringer AC., Zimmerman RA., Siegel KR., Sutton LN., Savino PJ., Zackai EH., Meadows AT. Intracranial visual pathway gliomas in children with neurofibromatosis. Neurofibromatosis 1988a; 1: 212-222.

Packer RJ., Sutton LN., Bilaniuk LT., Radcliffe J., Rosenstock JG., Siegel KR., Bunin GR., Savino PJ., Bruce DA., Schut L. Treatment of chiasmatic/hypothalamic gliomas of childhood with chemotherapy: an update. Ann Neurol 1988b; 23: 79-85.

Packer RJ., Nicholson HS., Johnson DL., Vezina G. Dilemmas in the management of childhood brain tumors; brainstem gliomas. Pediatr Neurosurg 1992; 17: 37-43.

Packer RJ., Lange B., Ater J., Nicholson J., Allen J., Walker R., Prados M., Jakacki R., Reaman GR., Needles MN., Phillips PC., Ryan J., Boyett JM., Geyer R., Finlay J. Carboplatin and vincristine for progressive low grade gliomas of childhood. J Clin Oncol 1993;11:850-857.

Packer RJ., Boyett JM., Zimmerman RA., Albright AL., Kaplan AM., Rorke LB., Selch MT., cherlow JM., Finlay JL., Wara WM. Outcome of children with brain stem gliomas after treatment with 7800 cGy of hyperfractionated radiotherapy. A Children's Cancer Group phase I/II trial. Cancer 1994; 74: 1827-34.

Packer RJ., Prados M., Phillips P., Nicholson HS., Boyett JM., Goldwein J., Rorke LB., Needle MN., sutton LN., Zimmerman RA., Fitz CR., Vezina LG., Etcubanas E., Wallenberg JC., Reaman G., Wara W. Treatment of children with newly diagnosed brain stem gliomas with intravenous recombinant-interferon and hyperfractionated radiation therapy: a Children's Cancer Group phase I/II study. Cancer 1996; 77(10): 2150-2156.

Packer RJ, Ater J, Allen J, Phillips P, Geyer R, Nicholson HS, Jakacki R, Kurczynski E, Needle M, Finlay J, Reaman G, Boyett JM. Carboplatin and vincristine chemotherapy for children with newly diagnosed progressive low grade gliomas. J Neurosurg 1997; 86: 747-754.

Packer RJ. Chemotherapy: low grade gliomas of the hypothalamus and thalamus. Pediatr Neurosurg 2000; 32: 259-263.

Panitch ES., Berg BO. Brain stem tumors of childhood and adolescence. Am J Dis Children 1970; 119: 465-472.

Pencalet P., Maixner W., Sainte-Rose C., Lellouch-Tubiana A., Cinalli G., Zerah M., Pierre-Kahn A., Hoppe-Hirsch E., Bourgeois M., Renier D. Benign cerebellar astrocytomas in children. J Neurosurg 1999; 90: 265-273.

Perilongo G., Carollo C., Salviati L., Murgia A., Pillon M., Basso G., Gardiman M., Laverda A.M. Diencephalic syndrome and disseminated juvenile pilocytic astrocytomas of the hypothalamic-optic chiasm region. Cancer 1997; 80: 142-146.

Perilongo G., Moras P., Carollo C., Battistella A., Clementi M., Laverda AM., Murgia A. Spontaneous partial regression of low grade glioma in children with neurofibromatosis-1: A real possibility. J Child Neurol 1999; 14: 352-356.

Perilongo G., Walker DA., Taylor RE., Zanetti I., Gnekow AK., Garré ML., Kuhl J., Robinson K. Vincristine (VCR) Carboplatin (CBDCA) in hypothalamic-chiasmatic low grade glioma (HC-LGG). SIOP-LGG study report. Med Pediatr Oncol 2000; 35: 190 (meeting abstract).

Petronio J., Edwards MSB., Prados M., Freyberger S., Rabbitt J., Silver P., Levin VA. Management of chiasmal and hypothalamic gliomas of infancy and childhood with chemotherapy. J Neurosurg 1991; 74: 701-708.

Pierce SM., Barnes PD., Loeffler JS., McGinn C., Tarbell NJ. Definitive radiation therapy in the management of symptomatic patients with optic glioma. Cancer 1990; 65: 45-52.

Pollack IF., Hurtt M., Pang D., Albright A.L. Dissemination of low grade intracranial astrocytomas in children. Cancer 1994; 73: 2869-2878.

Pollack, IF. Brain tumors in children. New Engl J Med 1994a; 331 1500-1507.

Pollack IF., Pang D., Albright AL. The long-term outcome in children with late-onset aqueductal stenosis resulting from benign intrinsic tectal tumors. J Neurosurg 1994; 80: 20-25.

Pollack IF., Claassen D., Al-Shboul Q., Janosky JE., Deutsch M. low grade gliomas of the cerebral hemispheres in children: an analysis of 71 cases. J Neurosurg 1995; 82: 536-547.

Pollack IF., Shultz B., Mulvihill JJ. The management of brainstem gliomas in patients with neurofibromatosis 1. Neurology 1996; 46: 1652-1660.

Pollack IF. The role of surgery in pediatric gliomas. J Neuro-Oncology 1999; 42: 271-288.

Pons MA., Finlay JL., Walker RW., Puccetti D., Packer RJ., McElwain M. Chemotherapy with vincristine and etoposide in children with low grade astrocytoma. J Neuro-Oncol 1992; 14: 151-158.

Prados M., Krouwer HG, Edwards MS, Cogen PH, Davis RL, Hoshino T. Proliferative potential and outcome in pediatric astrocytic tumors. J Neurooncol 1992; 13: 277-282.

Prados MD., Edwards MSB., Rabbitt J., Lamborn K., Davis RL., Levin VA. Treatment of pediatric low grade gliomas with a nitrosourea-based multiagent chemotherapy regimen. J Neuro-Oncol 1997; 32: 235-241.

Przybylski GJ., Albright AL., Martinez AJ. Spinal cord astrocytomas: long-term results comparing treatments in children. Child Nerv Syst 1997; 13: 375-382.

Ravens-Sieberer, Calaminus G. PEDQOL – introducing a European quality of life (QOL) instrument for children with cancer.Psycho-Oncology 1998,254.

Razack N., Baumgartner J., Bruner J. Pediatric oligodendroglioma. Pediatr Neurosurg 1998; 28: 121-129.

Rhodes R.H. Biological evaluation of biopsies from adult cerebral astrocytomas: cell growth/cell suicide ratios and their relationship to patient survival. J Neuropathol Exp Neurol 1998; 57: 746-757.

Riccardi V.M. Von Recklinghausen Neurofibromatosis. NEJM 1981; 305: 1617-1627.

Riccardi V.M. Neurofibromatosis: past, present and future. NEJM 1991; 324: 1283-1285.

Riccardi VM. Neurofibromatosis: phenotype, natural history, and pathogenesis. Baltimore: Johns Hopkins University Press; 1992: 1-450.

Riccardi VM. Histogenesis control genes and neurofibromatosis 1. Eur J Pediatr 2000; 159: 475-476.

Riffaud L., Vinchon M., Ragragui O., Delestret I., Ruchoux MM., Dhellemmes P. Hemispheric cerebral gliomas in children with NF I: arguments for a long term follow-up. Child's Nerv Syst 2002; 18: 43-47.

Rilliet B., Vernet O. Gliomas in children: a review. Child's Nerv Syst 2000; 16: 735-741.

Roach ES., Gomez MR., Northrup H. Tuberous sclerosis complex consensus conference: revised diagnostic criteria. J Child Neurol 1998; 13: 624-628.

Robertson PL., Muraszko KM., Brunberg JA., Axtell RA., Dauser RC., Turrisi AT. Pediatric midbrain tumors: a benign subgroup of brainstem gliomas. Pediatr Neurosurg 1995; 22: 65-73.

Rodman JH., Murry DJ., Madden T., Santana VM. Altered etoposide pharmacokinetics and time to engraftment in pediatric patients undergoing autologous transplantation. J Clin Oncol 1994; 12: 2390-2397.

Rodriguez LA., Edwards MS., Levin VA. Management of hypothalamic gliomas in children: an analysis of 33 cases. Neurosurgery 1990; 26: 242-246.

Rodrigues GB, Waldron JN, Wong CS, Laperriere NJ. A retrospective analysis of 52 cases of spinal cord glioma managed with radiation therapy. Int J Radiat Oncol Biol Phys 2000; 48: 837-842

Rollins NK., Lowry PA, Shapiro KN. Comparison of gadolinium-enhanced MR and thallium-201 single photon emission computed tomography in pediatric brain tumors. Pediatr Neurosurg 1995; 22: 8-14.

Rollins NK., Shapiro KN. The use of early postoperative MR in detecting residual juvenile cerebellar pilocytic astrocytoma. Am J Neuroradiol 1998; 19: 151-156.

Rosenstock JG., Evans AE., Schut L. Response to vincristine of recurrent brain tumors in children. J Neurosurg 1976; 45: 135-140.

Rossitch E Jr., Zeidman SM., Burger PC., Curnes JT., Harsh C., Anscher M., Oakes WJ. Clinical and pathological analysis of spinal cord astrocytomas in children. Neurosurgery 1990; 27: 193-196.

Rush JA., Young BR., Campbell RJ., MacCarthy CS. Optic glioma, long-term follow-up of 85 histopathologically verified cases. Ophthalmology 1982; 89: 1213-1219.

Russell DS., Rubinstein L. Pathology of tumors of the nervous system. Baltimore: Williams & Wilkens, 1989.

Rutka JT., George RE., Davidson G., Hoffmann HJ. Low grade astrocytoma of the tectal region as an unusual cause of knee pain: case report. Neurosurgery 1991; 29: 608-612.

Saran FH., Baumert BG., Khoo VS., Adams EJ., Garre ML., Warrington AP., Brada M. Stereotactically guided conformal radiotherapy for progressive low grade gliomas of childhood. Int J Radiat Oncol biol Phys 2002; 53: 43-51.

Sasaki H., Yoshida K., Ikeda E., Ason H., Inaba M., Otani M., Kawase T. Expression of the neural cell adhesion molecule in astrocytic tumors: an inverse correlation with malignancy. Cancer 1998; 82: 1921-1931.

Sasaki M., Kuwabara Y., Yoshida T., Nakagawa M., Fukumura T., Mihara F., Morioka T., Fukui M., Masuda K. A comparative study of thallium-201 SPET, carbon-11 mthionine PET and fluorine-18 fluorodeoxyglucose PET for the differentiation of astrocytic tumors. Eur J Nucl Med 1998; 25: 1261-1269.

Saunders MP., Denton CP., O'Brian ME., Blake P., Gore M., Wiltshaw E.: Hypersensitivity reactions to cisplatin and carboplatin - a report on six cases. Annals Of Oncology 1992, 3 (7), 574-6.

Scaradovou et al: Cancer 76: 1860-67, 1995.

Schäfer H., Müller H-H. Modification of the sample size and the schedule of interim analyses in survival trials based on data inspections. Statistics in Medicine 2001; 20: 3741-3751.

Schaetz CR., Kreth FW., Faist M., Warnke PC., Volk B., Ostertag CB. Interstitial 125-Iodine Radiosurgery of low grade gliomas of the insula of Reil. Acta Neurochirurgica 1994; 130: 80-89.

Schmandt SM., Packer RJ., Vezina LG., Jane J. Spontaneous regression of low grade astrocytomas in childhood. Pediatr Neurosurg 2000; 32: 132-136.

Schneider JH., Raffel C., McComb JG. Benign cerebellar astrocytomas of childhood. Neurosurgery 1992; 30: 58-63.

Schwartz AM., Ghatak NG. Malignant transformation of benign cerebellar astrocytoma. Cancer 1990; 65: 333-336.

Schütz J., Kaatsch P. Epidemiology of pediatric tumors of the central nervous system. Expert Rev Neurotherapeutics 2002; 2: 469-479.

Setty SN., Miller DC., Camras L., Charbel F., Schmidt M.L. Desmoplastic infantile astrocytoma with metastases at diagnosis. Mod Pathol 1997; 10(9): 945-951.

Shaw EG., Daumas-Duport C., Scheithauer BW., Gilbertson DT., O'Fallon JR., Earle JD., Laws ER Jr., Okazaki H. Radiation therapy in the management of low grade supratentorial astrocytomas. J Neurosurg 1989; 70: 853-861.

Shen MH., Harper PS., Upadhyaya M. Molecular genetics of neurofibromatosis type 1 (NF I). J Med Genet 1996; 33: 2-17.

Shibamato Y., Kitakabu Y., Takahashi M., Yamashita J., Oda Y., Kikuchi H., Abe M. Supratentorial low grade astrocytoma. Correlation of computed tomography findings with effect of radiation therapy and prognostic variables. Cancer 1993; 72: 190-195.

Shirato H., Kamada T., Hida K., Koyanagi I., Iwasaki Y., Miyasaka K., Abe H. The role of radiotherapy in the management of spinal cord glioma. Int J Radiat Oncol Biol Phys 1995; 33: 323-328.

Shlebak AA., Clark PI., Green JA. Hypersensitivity and cross-reactivity to Cisplatin and analogues. Cancer Chemotherapy and Pharmacology 1995; 35 (4); 349-51.

Sidransky D., Mikkelsen T., Schwechheimer K. Rosenblum ML., Cavenee W., Vogelstein B. Clonal expansion of p53 mutant cells is associated with brain tumor progression. Nature 1992; 355: 846-847.

Silva MM., Goldman S., Keating G., Marymont MM., Kalapurakal J., Tomita T. Optic pathway hypothalamic gliomas in children under three years of age: the role of chemotherapy. Pediatr Neurosurg 2000; 33: 151-158.

Smith MA, Rubenstein L, Anderson JR, Arthur D, Catalano Pj, Freidlin B, Heyn R, Khayat A, Krailo M, Land VJ, Miser J, Shuster J, Vena D. Secondary leukemia or myelodysplastic syndrome after treatment with epipodophyllotoxins. J Clin Oncol 1999; 17 (2) 569-577

Smith JS., Perry A., Borell TJ., Lee HK., O'Fallon J., Hosek SM., Kimmel D., Yates A., Burger PC., Scheithauer BW., Jenkins RB. Alterations of chromosome arms 1p and 19q as predictors of survival in oligodendrogliomas, astrocytomas, and mixed oligoastrocytomas. J Clin Oncol 2000 18: 636-645.

Smoots DW., Geyer JR., Lieberman DM., Berger MS. Predicting disease progression in childhood cerebellar astrocytoma. Child Nerv Ssyt 1998; 14: 636-648.

So E.L. Integration of EEG, MRI, and SPECT in localizing the seizure focus for epilepsy surgery. Epilepsia 2000; 41 (Suppl.3): S48-S54.

Somaza SC., Kondziolka D., Lunsford LD., Flickinger JC., Bissonette DJ.; Albright AL. Early outcomes after stereotactic radiosurgery for growing pilocytic astrocytomas in children. Pediatric Neurosurgery 1996; 25: 109-115.

Souweidane MM., Hoffman HJ. Current treatment of thalamic gliomas in children. J Neuro-Oncology 1996; 28: 157-166.

Steen RG, Spence D, Wu S, Xiong X, Kun LE, Merchant TE. Effect of therapeutic ionizing radiation on the human brain. Ann Neurol 2001; 50: 787-795.

Stiller CA., Nectoux J. International Incidence of Childhood Brain and Spinal Tumors. Int J Epidemiology 1994; 23: 458-464.

Stroink AR., Hoffman JH., Hendrick EB., Humphreys RP., Davidson G. Transependymal benign dorsally exophytic brain stem gliomas in childhood: Diagnosis and treatment recommendations. Neurosurgery 1987; 20: 439-444.

Strojan P., Petric-Grabnar G., Zupancic N., Jereb B. Concomitant chemoradiotherapy for incompletely resected supratentorial low grade astrocytoma in children: preliminary report. Med Pediatr Oncol 1999; 32: 112-116.

Sung DI. Suprasellar tumors in children: a review of clinical manifestations and managements. Cancer 1982; 50: 1420-1425.

Sutton LN, Molloy PT, Sernyak H, Goldwein J, Phillips PL, Rorke LB, Moshang T Jr, Lange B, Packer RJ. Long-term outcome of hypothalamic/chiasmatic astrocytomas in children treated with conservative surgery. J Neurosurg 1995, Oct; 83(4): 583-589.

Sutton LN., Cnaan A., Klatt L., Zhao BSH., Zimmerman R., Needle M., Molloy P., Philips P. Postoperative surveillance imaging in children with cerebellar astrocytoma. J Neurosurg 1996; 84: 721-725.

Tabor PA. Drug induced fever. Drug Intelligence and Clinical Pharmacy 1986; 20 (6); 413-420.

Takeuchi H., Kabuto M., Sato K. Kubota T. Chiasmal gliomas with spontaneous regression: Proliferation and apoptosis. Child's Nerv Syst 1997; 13: 229-233.

Tallman MS, Gray R, Bennett JM, Variakojis D, Robert N, Wood WC, Rowe JM, Wiernik PH. Leukemogenic potential of adjuvant chemotherapy for early-stage breast cancer: the Eastern Cooperative Oncology Group experience. J Clin Oncol 1995 Jul; 13(7): 1557-63.

Tamura M., Zama A., Kurihara H., Fujimaki H., Imai H., Kano T., Saitoh F. Management of recurrent pilocytic astrocytoma with leptomeningeal dissemination in childhood. Child Nerv Syst 1998; 14: 617-622.

Tao ML, Barnes PD, Billett AL, Leong T, Shrieve DC, Scott RM, Tarbell NJ. Childhood optic chiasm gliomas: radiographic response following radiotherapy and long-term clinical outcome. Int J Radiat Oncol Biol Phys 1997; 39: 579-587

Tarbell NJ., Loeffler JS. Recent trends in the radiotherapy of pediatric gliomas. J Neuro-Oncol 1996; 28: 233-244.

Taveras J., Lester A., Wood E. The value of radiation therapy in the management of glioma of the optic nerves and chiasma. Radiology 1956; 66: 518-528 (Abstract).

Tenny RT, Laws ER, Jr., Younge BR, Rush JA. The neurosurgical management of optic glioma. Results in 104 patients. J Neurosurg 1982; 57: 452-458

Ter Schihorst C., Bousquet J., Menardo JL. et al: Desensibilisation specifique au cis-Dichloro-Diamino-Platinum (DDP) chez un malade allergique. Presse Medicale 1986; 15 (26); 1242.

Therasse P, Arbuck SG, Eisenhauer EA, Wanders J, Kaplan RS, Rubinstein L, Verweij J, van Glabbeke M, van Oosterom AT, Christian MC, Gwyther SG: New guidelines to evaluate the response to treatment in solid tumors. J.Nat Cancer Inst, February 2000, Vol.92, 205-216.

Tihan T., Fisher PG, Kepner JL, Godfraind C, McComb RD, Goldthwaite PT, Burger PC. Pediatric astrocytoma with monormphous pilomyxoid features and a less favourable outcome. J Neuropath Exp Neurol 58: 1061-68, 1999.

Tomita T., Cortes RF. Astrocytomas of the cerebral peduncle in children: surgical experience in seven patients. Child's Nerv Syst 2002; 18: 225-230.

Trigg M., Swanson JD., Letellier MA. Metastasis of an optic glioma through a ventricularperitoneal shunt. Cancer 1983; 52: 599-601.

Undijan S., Marinov M., Georgiev K. Long-term follow-up after surgical treatment of cerebellar astrocytomas in 100 children. Child Nerv Syst 1989; 5: 99-101.

Ushio Y., Kochi M. Intrathecal perfusion chemotherapy against subarachnoid dissemination of glioma in children. (Meeting Abstract) 6th International Symposium on Pediatric Neuro-Oncology, Houston, 1994.

Valagussa P, Moliterni A, Terenziani M, Zambetti M, Bonadonna G. Second malignancies following CMF-based adjuvant chemotherapy in resectable breast cancer. Ann Oncol 1994 Nov; 5(9): 803-8

Van Arsdel PP. Jr.: Drug reactions: Allergy and near-allergy. Annals of Allergy 1986; 57 (5); (305-312).

Vandertop WP., Hoffman JH., Drake JM., Humphreys RP., Rutka JT., Armstrong DC., Becker LE. Focal midbrain tumors in children. Neurosurgery 1992; 31: 186-194.

Varni JW, Rode CA, Seid M, Katz ER, Friedman-Bender A, Quiggins DJ. The Pediatric Cancer of Life Inventory-32 (PCQL-32). II Feasibility and range of measurement. J Behav Med 1999;22:397-406.

Versari P., Talamonti G., D'Aliberti G., Fontana R., Colombo N., Casadei G.: Leptomeningeal Dissemination of Juvenile Pilocytic Astrocytoma: Case report. Surgical Neurology 1994; 41: 318-321.

Vinchon M., Soto-Ares G., Ruchoux MM., Dhellemmes P. Cerebellar gliomas in children with NF I: pathology and surgery. Child Nerv Syst 2000; 16: 417-420.

Voges J., Sturm V., Berthold F., Pastyr O., Schlegel W., Lorenz WJ. Interstitial irradiation of cerebral gliomas in childhood by permanently implanted 125-Iodine - preliminary results. Klin Pediatr 1990; 202: 270-274.

Von Bossanyi P., Sallaba J., Dietzmann K., Warich-Kirches M., Kirches E. Correlation of TGF-alpha and EGF-receptor expression with proliferative activity in human astrocytic glioma. Pathol Res Practice 1998; 194: 141-147.

Von Deimling A., Louis DN., Menon AG., Ellison D., Wiestler OD., Seizinger BR. Allelic loss on the long arm of chromosome 17 in pilocytic astrocytoma. Acta Neuropathol 1993; 86: 81-85.

Walker DA., Taylor RE., Perilongo G., Zanetti I., Gnekow AK. Vincristine (VCR) carboplatin (CBDCA) in low grade glioma : an interim report of the international consortium on low grade glioma (ICLGG). IXth Symposium Pediatric NeuroOncology . 2000 (meeting abstract).

Wallner KE., Gonzales MF., Edwards MSB., Wara WM., Sheline GE. Treatment results of juvenile pilocytic astrocytoma. J. Neurosurg 1988; 69: 171-176.

Warrington AP., Laing RW., Brada M. Quality assurance in fractionated stereotactic radiotherapy. Radiother Oncol 1994; 30: 239-246.

Watson PR., Guthrie TH Jr., Caruana RJ.: Cisplatin-associated hemolytic-uremic syndrome. Successful treatment with a staphylococcal protein A column. Cancer 1989; 64 (7); 1400-3.

Weiss L., Sagerman RH., King GA., Chung CT., Dubowy RL. Controversy in the management of optic nerve glioma. Cancer 1987; 59: 1000-1004.

West CGH., Gattamaneni R., Blair V. Radiotherapy in the treatment of low grade astrocytomas. I. A survival analysis. Child Nerv Syst 1995; 11: 438-442.

Willert JR, Daneshvar L, Sheffieled VC, Cogen PH. Deletion of Chromosome Arm 17 p DNA sequences in pediatric high-grade an juvenile pilocytic astrocytomas. Genes Chromosomes Cancer 1995; 12: 165-172.

Wisoff JH., Epstein FJ. Pseudobulbar palsy after posterior fossa operation in children. Neurosurgery 1984; 15: 707-709.

Wisoff JH, Abbott R, Epstein F. Surgical management of exophytic chiasmatic-hypothalamic tumors of childhood. J Neurosurg 1990; 73: 661-666.

Wolff JEA., Däumling E., Dirksen A., Dabrock A., Hartmann M., Jürgens H. Fertigkeitenskala Münster-Heidelberg. Ein Meßinstrument zum globalen Vergleich von Krankheitsfolgen. Klin Pädiatr 1996; 208:1-5.

Wong JY., Uhl V., Wara WM., Sheline GE. Optic gliomas. A reanalysis of the University of California, San Francisco experience. Cancer 1987; 60: 1847-1855.

Yasunari T., Shiraki K., Hattori H., Miki T. Frequency of choroidal abnormalities in neurofibromatosis type 1. Lancet 2000; 356: 988-992.

Yu DY., Dahl GV., Shames RS., Fisher PG. Weekly dosing of carboplatin increases risk of allergy in children. J Pediatr Hematol Oncol 2001; 23 (6): 349-352.

Zweizig S., Roman LD., Muderspach LI.: Death from anaphylaxis to cisplatin: a case report. Gynecologic oncology 1994; 53 (1); 121-122.

21. Addendum SIOP - LGG 2004

21.1. Approval from the ethics committee

21.2. Declaration of center participation

21.3. Patient information

21.4. Consent forms for
21.4.1. Study participation
21.4.2. Data registration
21.4.3. Tumor tissue bank

21.5. Patient study registration
21.5.1. Basic patient data
21.5.2. Basic therapy information

21.6. Randomisation forms
21.6.1. Randomisation form
21.6.2. Result of randomisation

21.7. Forms for administration / prescription of chemotherapy
21.7.1. Induction
I. Vincristin/Carboplatin
II. Vincristin/Carboplatin/Etoposide
21.7.2. Consolidation
(regular protocol consolidation and alternative consolidation following allergy
or early progression)
I. Vincristin/Carboplatin
II. Vincristin/Cisplatin
III. Vincristin/Cyclophosphamid

21.8. Documentation of chemotherapy
21.8.1. Induction
I. Vincristin/Carboplatin – week 1 - 24
II. Vincristin/Carboplatin/Etoposide – week 1 - 24
21.8.2. Consolidation
Regular protocol consolidation – week 25 – 54
Regular protocol consolidation – week 55 - 85
Alternative consolidation post allergy – week 1 - 30
Alternative consolidation post allergy – week 31 - 61
21.8.3. Response assessment
21.8.4. Documentation of toxicity

21.9. Documentation of radiotherapy
21.9.1. Patient data / Maximal acute morbidity during RT

21.9.2. Maximal acute morbidity at the end of RT
21.9.3. Myelotoxicity during craniospinal irradiation
21.9.4. Dose prescription / Treatment technique: primary tumor site
21.9.5. Dose prescription: Craniospinal axis
21.9.6. Treatment technique craniospinal axis: Helmet technique, Spinal axis
21.9.7. Radiotherapy of Metastatic deposits
21.9.8. Brachytherapy: Technique / dose prescription
21.9.9. Tumor response
21.9.10.Late effects of Radiotherapy
21.9.11.Relapse form

21.10. Histopathology report forms

21.11. Common toxicity criteria

21.12. Report of serious adverse event

21.13. Follow-up forms
21.13.1. Investigations during follow-up
21.13.2. Patient status report
21.13.3. Event report form
21.13.4. Assessment at registration: Neurology, endocrinology and education
21.13.5. Endocrinology – Follow up
21.13.6. Ophthalmology data form
21.13.7. Post treatment: Late effects and education

21.14. List of participating centers

GCP: Instructions to fill out the forms and to make corrections:

- Please fill in the forms with permanent ink or ball-pen only. Entries with pencil are not acceptable.
- Corrections have to be made the following way: The wrong information is crossed out with a straight line, the correct information is written beside and signed by the investigator with date and, if needed, with the reason for the correction.
- In case data are missing and fields have to be left blank, a comment should be made.
- Forms should be completed in a timely manner, should be controlled by the investigator and subsequently signed including the date and sent to the study center.

Dates

All dates should be given in a uniform manner with day of the month followed by the month of the year and then the year: dd mm yyyy (day month year).

21.1. Vote of the Ethics Committee

SIOP - LGG 2004

Institutional/local ethicical approval must follow national practice.
Each national study group has to ensure, that the existing guidelines are respected and the study protocol not be activeted before this approval has been obtained.

21.2. Declaration of Participation in the SIOP-LGG 2004-Study

Therapy of low grade glioma according to the SIOP-LGG 2004 protocol

Name of physician responsible for SIOP - LGG 2004:

____

Hospital_______________________Country:_______________

- Patients aged 0-18 (and older in individual cases) with a low grade glioma of any location will be registered for the study.
- Protocol instructions will be observed considering medical responsibility in each individual case.
- Requested information will be given for each patient, as well as pathologic material and fresh frozen tissue be sent (for molecular biologic investigations).

		**Signature**
**Pediatric oncologist**	name	
	address..	
	phone.:................./...................... FAX....................../..........................	
	e-mail...	
**Neurosurgeon**	name	
	address..	
	phone.:................./...................... FAX....................../..........................	
	e-mail...	
**Radiotherapist**	name	
	address..	
	phone.:................./...................... FAX....................../..........................	
	e-mail...	
**Pathologist**	name	
	address..	
	phone.:................./...................... FAX....................../..........................	
	e-mail...	

Please return to: (national coordinating center)

21.3. Patient Information SIOP - LGG 2004

Declaration of Consent to
21.4.1. Study participation
21.4.2. Data registration
21.4.3. Release of tumor tissue for tumor tissue bank

Accepted national procedures for patient consent are to be used. Therefore these forms have to be designed seperately by each participating national group.

The patient's and/or parent's written consent to participate in the study must be obtained after a full explanation has been given of the treatment options including the conventional and generally accepted methods of treatment and the manner of treatment allocation.
If the patient is a minor, the treatment must be explained to and consent received from his/her guardian. Additionally the child should receive an explanation as to his/her means of understanding and should give consent as well, if he/she is able to do so. Enough time and the opportunity to discuss participation before the decision for and start of treatment have to be given. The right of a patient to refuse to participate without giving reasons must be respected.

Consent for participation in the study and for data management will be obtained separately. If applicable, consent for sending diagnostic material, especially tumor tissue, to reference institutions and tissue banks should be obtained.

21.5.1. Patient Registration - Basic patient data

SIOP - LGG 2004
Page 1/3

(National Coordinating center):

Surname, Prename I______________________________________I
Pat.-No. I_I_I_I_I
Hospital I__I__I__I__I__I
Patient Identity No. I__I I__I__I__I__I__I__I I__I__I__I__I__I__I
sex:1=m,2=f date of birth

! Please respect, that written informed consent has to be obtained before this form is forwarded and the information is saved!

Participation in the study:: ❒ (preliminary) observation group ❒ therapy group ❒ no participation

Hereditary disease::
❒ Neurofibromatosis type NFI ❒ no ❒ yes: ❒ familial ❒ sporadic
❒ features present, but international criteria not met
❒ Tuberous Sclerosis ❒ no ❒ yes
❒ other:________________________

Circumstances of diagnosis:: ❒ Symptoms of disease
❒ Accidental finding during investigation for another disease
❒ Other:____

Earliest manifestation of symptoms clearly related to tumor:
before I__I__I weeks / I__I__I months: Tick as appropriate and fill in details:

Symptoms of increased intracranial pressure: ❒ no ❒ yes ___________________________________
Neurologic symptoms: ❒ no ❒ yes ✱ complete neurology form 21.13.4.
Visual disturbances: ❒ no ❒ yes ✱ complete ophthalmology form 21.13.6.
Endocrinologic symptoms ❒ no ❒ yes ✱ complete endocrinology form 21.13.4.
Diencephalic symptoms: ❒ no ❒ yes ___________________________________
Other: ❒ no ❒ yes ___________________________________

Any other symptoms at diagnosis (please detail):____
____
____

Neuro-Radiology at diagnosis (preoperative) - local

Date of diagnosis by imaging: I__I__I . I__I__I . I__I__I__I__I

❒ **MRI** ❒ with contrast enhancement ❒ without contrast enhancement

❒ **CT** ❒ with contrast enhancement ❒ without contrast enhancement

Tumor size: I__I__I cm x I__I__I cm x I__I__I cm (solid part + cystic part, if present)

Cystic part: ❒ no ❒ yes

Size of the cyst: I__I__I cm x I__I__I cm x I__I__I cm

Ventricular dilatation: ❒ no ❒ yes

Mass effect: ❒ no ❒ yes: ❒ local ❒ midline shift

Histology at first operation (2/3)

❒ **No histology**

❒ **Date of diagnosis by histology:** I__I__I . I__I__I . I__I__I__I__I

Neuropathology (local): ______________________________ **E-No:**__________________________________

Central pathologic review: ❒ **No** ❒ **Yes** **R-No:**__________________________________

Discrepancy of diagnosis: ❒ **No** ❒ **Yes, histology ref. center:**__________________________________

Histopathologic classification and grading according to WHO (Kleihus a. Cavanee, 2000) - local:

1. ❒ Astrocytic tumors
1.1. ❒ Pilocytic astrocytoma
1.1.1. ❒ pilomyxoid variant
1.2. ❒ Astrocytoma nos.
1.2.1. ❒ fibrillary astrocytoma
1.2.2. ❒ protoplasmatic astrocytoma
1.2.3. ❒ gemistocytic astrocytoma
1.3. ❒ Pleomorphic xanthoastrocytoma
1.4. ❒ Subependymal large cell astrocytoma

2. ❒ Oligodendro-glial tumors
2.1. ❒ Oligodendroglioma not otherwise specified

3. ❒ Mixed glioma
3.1. ❒ Oligo-astrocytoma
3.2. ❒ other mixed glioma

4. ❒ Neuronal and mixed glial-neuronal tumors
4.1. ❒ DIGG/DIA - desmoplastic, infantile ganglioglioma/-astrocytoma
4.2. ❒ DNT - dysembryoplastic, neuroepithelial tumor
4.3. ❒ Ganglioglioma
4.4. ❒ atypical myxomatous neuroepithalial tumor

5. ❒ Non-study diagnoses:
❒ Pineocytoma
❒ Choroid plexus papilloma
❒ Neurinoma
❒ acoustic neurinoma (❒ NF II)
❒ Gangliocytoma
❒ other:__________________________________

➨ **Malignancy according to WHO-classification**: ❒ **°I** ❒ **°II** ❒ **°III** ❒ **°IV** (if applicable)

LOCALISATION

(Please underline main localisation and indicate all structures involved)

1. ❒ Cerebral hemisphere
1.1. ❒ frontal lobe
1.2. ❒ parietal lobe
1.3. ❒ temporal lobe
1.4. ❒ occipital lobe

2. ❒ Supratentorial midline
2.1. ❒ Anterior part of optic nerve (including orbital part)
2.2. ❒ Optic chiasm
2.3. ❒ Optic tract
2.4. ❒ Diencephalon
2.4.1. ❒ Hypothalamus
2.4.2. ❒ 3rd ventricle
2.4.3. ❒ Thalamus
2.4.4. ❒ Basal ganglia
2.4.5. ❒ Corpus callosum
2.4.6. ❒ Hypophysis
2.4.7. ❒ Limbic system/Fornix
2.5. ❒ Mesencephalon
2.5.1. ❒ Crus cerebri
2.5.2. ❒ Tegmentum
2.5.3. ❒ Tectum/Lamina quadrigemina
2.5.4. ❒ Pineal region

3. ❒ Cerebellum
3.1. ❒ vermis
3.2. ❒ cerebello-pontine angle
3.3. ❒ hemisphere

4. ❒ Caudal brainstem
4.1. ❒ IVth Ventricle
4.2. ❒ Pons focal
4.3. ❒ Pons intrinsic
4.4. ❒ Medulla oblongata
4.5. ❒ cranio-spinal junction

5. ❒ Spinal canal
5.1. ❒ intraspinal, extradural
5.2. ❒ subdural, extramedullary
5.3. ❒ intramedullary

ad 5.1.-5.3.:
Segments involved:_____________

6. ❒ Lateral ventricle
(Giant cell astrocytoma with tuberous sclerosis)

Supplementary to localisation:
____
____
____

Dodge-classification of optic pathway glioma:
❒ **I (Optic nerve only)** ❒ **II (Chiasm ± optic nerve)** ❒ **III (Chiasma + diencephalic extension)**

Side of main tumor localisation: ❒ right ❒ left ❒ on both sides ❒ midline

Primary metastases: ❒ No ❒ Yes, where:__________________________________
(Section 16.1) ❒ M 1 ❒ M 2 ❒ M 3 ❒ M 4

Primary surgical intervention (3/3)

Shunt implantation before/after tumor operation: ❒ no ❒ yes, date: I__I__I . I__I__I . I__I__I__I__I

Type of shunt:____

Date of surgery: I__I__I . I__I__I . I__I__I__I__I **Hospital, name of surgeon:**_____________________

____

Extent of resection:

❒ S1 total resection (no visible residual tumor)
❒ S2 subtotal resection (residual tumor < 1,5 cm ³ , local invasion)
❒ S3 partial resection (residual tumor > 1,5 cm³)
❒ S4 biopsy ❒ open ❒ stereotactic ❒ endoscopic

Neuro-Radiology early postoperatively (within 72 hours)

Date: I__I__I . I__I__I . I__I__I__I__I **Technique** ❒ MRI ❒ CT
Contrast enhancement: ❒ no ❒ yes

Size of residual tumor: I__I__I cm x I__I__I cm x I__I__I cm

Finding:
❒ R1 no residual tumor
❒ R2 contrast enhancement, but small, not measurable
❒ R3 residual tumor of a measurable size
❒ R4 no change of size as compared to preoperative size (minimal change)

Definit extent of surgery: (SIOP-classification 1995)

	Radiology	Surgery
❒ total resection	R1	S1
❒ subtotal	R1 / R2	S2
❒ partial	R3	S1 / S2 / S3
❒ biopsy	R4	S4

Complete remission achieved? ❒ yes ❒ no

Postoperative management

❒ **Observation (wait and see)**
❒ **Therapy, which:** ❒ **Chemotherapy** ❒ **Radiotherapy** ❒ **Other**

____

In case of therapy: Please send the appropriate form "Basic therapy information" (21.5.2.) immediately to your national coordinator.
Start of postoperative therapy: I__I__I . I__I__I . I__I__I__I__I

Last date of follow-up: I__I__I . I__I__I . I__I__I__I__I ❒ patient alive ❒ patient dead

<u>Please send for central data management::</u>
❒ pre- and postoperative MRI and CT findings ❒ Histology (local and reference)
❒ surgical report

Remarks:

______________________ ______________________ ______________________

Stamp **Date** **Signature**

21.5.2. Patient Registration: Basic Therapy Information

SIOP - LGG 2004
Page 1/1

National coordinating center:

Surname, Prename Pat.-No. Hospital Patient Identity No.
I____I I_I_I_I_I I__I__I__I__I I__I I__I__I__I__I__I__I I__I__I__I__I__I
date of birth

❒ **Indication for therapy following clinical diagnosis or biopsy**
❒ **Indication for therapy following partial or subtotal resection**

Tick as appropriate:		
- Diencephalic syndrome	❒ No	❒ Yes
- Focal neurologic deficits secondary to tumor growth	❒ No	❒ Yes
- Seizures secondary to tumor growth	❒ No	❒ Yes
- Increased intracranial pressure secondary to tumor growth	❒ No	❒ Yes
- Definite history of visual deterioration	❒ No	❒ Yes
- Borderline vision ("threat to vision")	❒ No	❒ Yes
- Nystagmus due to visual impairment in infants	❒ No	❒ Yes
- Symptomatic metastases	❒ No	❒ Yes

- Radiological finding: The presence of a postoperative residual tumor is not an indication to therapy by its own.

❒ **Indication to therapy following observation**

- Definite history of visual deterioration	❒ No	❒ Yes
- Manifestation of new neurologic symptoms secondary to tumor growth	❒ No	❒ Yes
- Development of diencephalic syndrome	❒ No	❒ Yes
- Visual deterioration	❒ No	❒ Yes
- Any loss of vision in the second eye, if the other eye is blind	❒ No	❒ Yes
- Increase of tumor size of >25% (increase of the diameter of the optic nerve)	❒ No	❒ Yes
- Involvement of previously uninvolved areas of the brain	❒ No	❒ Yes
- Manifestation of new lesions (including symptomatic or progressive metastases)	❒ No	❒ Yes

Date of last neuroimaging before the start of therapy: I__I__I . I__I__I . I__I__I__I__I (ddmmyyyy)

Tumor size: I__I__I , I__I cm x I__I__I , I__I cm x I__I__I , I__I cm

MRI-images sent for central review? ❒ **Yes** ❒ **No** ❒ **Arranged**

Start of therapy: I__I__I . I__I__I . I__I__I__I__I (ddmmyyyy)

Patient receives: ❒ **Chemotherapy (following randomisation)** ❒ **Radiotherapy**

❒ Induction I + Consolidation
❒ Induction II + Consolidation

❒ conventional radiotherapy
❒ 125-Jodine-Seed-implantation
❒ other: ______________________
____

Which hospital provides therapy? ____

____________________ ____________________ ____________________
stamp date signature

21.6.1. Central Randomisation **SIOP LGG 2004**
Page 1/1

(National coordinating center)

Randomisation of Induction-Therapy

Pat.-Identity-Number: I__I I__I__I__I__I__I__I I__I__I__I__I__I

Treatment center / -town: ________________________________

Patient (Surname, Prename): ________________________________

Date of birth: I__I__I . I__I__I . I__I__I__I__I

Neurofibromatosis Type NF I ❒ **No** ❒ Not clear yet

Age of the patient: ❒ < 1 year ❒ < 8 years ❒ ≥ 8 years

Registration form sent? ❒ **Yes** ❒ No ⇒ not eligible

Localisation:

❒ **cerebral hemispheres**
❒ **cerebellum**
❒ **caudal brain stem**
❒ **spinal canal**
❒ **lateral ventricle**

❒ **supratentorial midline**
extension in the case of optic pathway glioma:
❒ Dodge I (optic nerve only) ⇒ not eligible
❒ Dodge II (Chiasma + optic nerve)
❒ Dodge III (Chiasma + extensions)

Date of original diagnosis: ❒ clinical ❒ histological I__I__I . I__I__I . I__I__I__I__I

Date of last resection before chemotherapy: I__I__I . I__I__I . I__I__I__I__I

Previous chemo- or radiotherapie ❒ **No** ❒ **Yes** ⇒ not eligible

Histopathologic diagnosis
Material sent for central review: ❒ No ❒ Yes, date of sending: I__I__I . I__I__I . I__I__I__I__I
❒ Arranged

Histological diagnosis at first operation:________________________________

Histological diagosis at last operation:________________________________

WHO-Classification: ❒ °I ❒ °II

MRI (pre- and early postoperatively resp. before start of therapy)
MRI sent for central review: ❒ No ❒ Yes, date of sending: I__I__I . I__I__I . I__I__I__I__I
❒ Arranged

Therapy at: ❒ **diagnosis** ❒ **progression (following observation)**

FAX-No. for response:..

______________________ ______________ ______________________________
stamp date signature

21.6.2. Result of Randomisation **SIOP - LGG 2004**
Page 1/1

Treatment center:

FAX-No. of the treatment center:

UPN-Nummer I__I I__I__I__I__I__I__I I__I__I__I__I__I SIOP-Study.-No.

Surname Prename Date of birth Hospital

Result of Randomisation

❐ **Induction I:** **Carboplatin / Vincristin**

❐ **Induction II:** **Carboplatin, Vincristin, Etoposide (VP 16)**

Date:__________________ Signature:______________________________________

We thank for the registration of your new patient who will receive chemotherapy according to our current study SIOP-LGG-2004.
Please respect the result of randomisation. We had declared our willingness to conduct therapy according to the randomisation at the start of the study. Only in substantiated exceptions this should be changed following consultation of the national study coordinator-

International study office for randomisation:

FAX-Number:

21.7. Chemotherapy SIOP - LGG 2004
Therapy details – flow sheets

The following pages contain basic recommendations for the application of chemotherapy within the setting of this study. It is acknowledged that locally standardised procedures for the combination therapy of this protocol exist. The therapy details of the SIOP - LGG 2004 protocol may well be adopted to these local procedures. It is the responsibility of the individual physician to assure patient safety while giving treatment, the protocol only offers a framework of orientation.

21.7.1. Induction

21.7.1.1. Vincristin/Carboplatin
21.7.1.2. Vincristin/Carboplatin/Etoposide

21.7.2. Consolidation

21.7.2.1. Vincristin/Carboplatin
21.7.2.2. Vincristin/Cisplatin
21.7.2.3. Vincristin/Cyclophosphamid

21.7.1.1. Induction I: Vincristin/Carboplatin SIOP - LGG 2004

Name:______________________________ date of birth:_______________

Weight:_______________ Length/Height:____________ BSA:_______ m²

Therapy Week:_____________

week	1	2	3	4	5	6	7	8	9	10	13	17	21	24
	**V**	**V**	**V**	**V**	**V**	**V**	**V**	**V**	**V**	**V**	**V**	**V**	**V**	
	**C**			**C**			**C**			**C**	**C**	**C**	**C**	
														MRI

I. Hydration: starting 3-6 hours prior to until up to 24 hours following Carboplatin:

Glucose 5 % / NaCl 0,9 % 1:1 2000 – 3000 ml / m² / 24 h = __________ ml

Per 500 ml: + 10 ml K Cl 7,45 % = __________ ml
+ 6 ml Magnesium 20 % = __________ ml
+ 10 ml Ca-Gluconat 10 % = __________ ml

II. Vincristin 1,5 mg / m² iv-bolus (max. 2 mg) = __________ mg
Day 1 – Week:_______________

III. Carboplatin 550 mg / m² 60 minutes-infusion in 200 ml Glucose 5 % = __________ mg
Day 1 – Week:__________

IV. Supportive Care:

Mannitol 20 % 40 ml / m² as short term infusion = __________ ml
if urine output falls below 2/3 of fluid input

Antiemetics:________________________________ = __________ mg
Starting 30‘ prior to chemotherapy.

Steroids may impair the efficacy of Platinum compounds on glial cells, they should be used restrictively:
Dexamethasone 0,15 mg / kg iv-bolus = __________ mg
If increased intracerebral pressure is manifest prior to or during therapy and shunting is not indicated.

Respect dose modifications due to toxicity and recommendations for supportive care (14.2.-3)

21.7.1.2. Induction II: Vincristin/Carboplatin/Etoposide

SIOP - LGG 2004

Name:___________________________________ date of birth:_________________

Weight:_________________ Length/Height:______________ BSA:________ m²

Therapy Week:________________

week	1	2	3	4	5	6	7	8	9	10	13	17	21	24
	V	V	V	V	V	V	V	V	V	V	V	V	V	
	C			C			C			C	C	C	C	
	Ex3			Ex3			Ex3			Ex3				
														MRI

I. Hydration: starting 3-6 hours prior to until up to 24 hours following Carboplatin:

Glucose 5 % / NaCl 0,9 % 1:1 2000 – 3000 ml / m² / 24 h = ___________ ml

Per 500 ml: + 10 ml K Cl 7,45 % = ___________ ml

+ 6 ml Magnesium 20 % = ___________ ml

+ 10 ml Ca-Gluconat 10 % = ___________ ml

II. Vincristin 1,5 mg / m² iv-bolus (max. 2 mg) = ___________ mg

Day 1 - Week: ____________

III. Carboplatin 550 mg / m² 60 minutes-infusion in 200 ml Glucose 5 % = ___________ mg

Day 1 – Week: ____________

IV. Etoposide 100 mg / m² 60 minutes infusion = ___________ mg

in _______ mlNa Cl 0,9 %

at a final concentration of 0,4 mg/ml

Day 1, 2, 3 – Week:________

V. Supportive Care:

Mannitol 20 % 40 ml / m² as short term infusion = ___________ ml

If urine output falls below 2/3 of fluid input

Antiemetics:____________________________________ **= ___________ mg**

Starting 30‘ prior to chemotherapy

Steroids may impair the efficacy of Platinum compounds on glial cells, they should be used restrictively:

Dexamethasone 0,15 mg / kg iv-bolus = ___________ mg

If increased intracerebral pressure is manifest prior to or during therapy and shunting is not indicated.

Respect dose modifications due to toxicity and recommendations for supportive care (14.2.-3)

21.7.2.1. Consolidation: Vincristin/Carboplatin

SIOP - LGG 2004

Name:____________________ date of birth:__________

Weight:__________ Length/Height:__________ BSA:________ m²

Therapy Week:________

Week:	Day1	Day 8	Day 15
	VCR<br>Carboplatin	VCR	VCR

I. Hydration: starting 3-6 hours prior to until up to 24 hours following Carboplatin:

Glucose 5 % / NaCl 0,9 % 1:1 2000 – 3000 ml / m² / 24 h = ___________ ml

Per 500 ml: + 10 ml K Cl 7,45 % = ___________ ml
+ 6 ml Magnesium 20 % = ___________ ml
+ 10 ml Ca-Gluconat 10 % = ___________ ml

II. Vincristin 1,5 mg / m² iv-bolus (max. 2 mg) = ___________ mg
Day 1, 8, 15

III. Carboplatin 550 mg / m² 60 minutes-infusion in 200 ml Glucose 5 % = ___________ mg
Day 1

IV. Supportive Care:

Mannitol 20 % 40 ml / m² as short term infusion = ___________ ml
If urine output falls below 2/3 of fluid input
Antiemetics:____________________ = ___________ mg
Starting 30‘ prior to chemotherapy.

Steroids may impair the efficacy of Platinum compounds on glial cells, they should be used restrictively:
Dexamethasone 0,15 mg / kg iv-bolus = ___________ mg
If increased intracerebral pressure is manifest prior to or during therapy and shunting is not indicated.

Respect dose modifications due to toxicity and recommendations for supportive care (14.2.-3)

21.7.2.2. Consolidation : Vincristin/Cyclophosphamide **SIOP - LGG 2004**

Name:_________________________ date of birth:_______________

Weight:_______________ Length/Height:_____________ BSA:_______ m²

Therapy Week:_______

Week:	Day1	Day 8	Day 15
	VCR<br>Cyclophosphamide	VCR	VCR

I. Hydration: starting 3-6 hours prior to until 24 hours following Cyclophosphamide:

Glucose 5 % / NaCl 0,9 % 1:1 2000 – 3000 ml / m² / 24 h = __________ ml

Per 500 ml: + 10 ml K Cl 7,45 % = __________ ml

II. Vincristin 1,5 mg / m² iv-bolus (max. 2 mg) = __________ mg
Day 1, 8, 15

III. MESNA 500 mg /m² iv-bolus = __________ mg
Before the start of Cyclophosphamide

IV. Cyclophosphamide 1500 mg / m² 60 minutes-infusion in 250 ml Na Cl 0,9 % = __________ mg
Day 1

V. MESNA* 500 mg /m² iv-bolus = __________ mg
4 and 8 hours after the start of Cyclophosphamide

VI. Supportive Care:

Furosemide 0,5 – 1 mg /kg as short term infusion = __________ mg
If urine output falls below 2/3 of fluid input at 6 hourly registration of fluid balance.

Antiemetics:_________________________ = __________ mg
Starting 30‘ prior to chemotherapy.

Dexamethasone 0,15 mg / kg iv-bolus = __________ mg
If increased intracerebral pressure is manifest prior to or during therapy and shunting is not indicared.

* Mesna can be given as a continuous infusion 1500 mg / m² / d as well and oral application is possible alternatively.

Respect dose modifications due to toxicity and recommendations for supportive care (14.2.-3)

21.7.2.3. Consolidation: Vincristin/Cisplatin **SIOP - LGG 2004 Page 1/1**

Name:_________________________________ **date of birth:**________________

Weight:________________ Length/Height:______________ BSA:________ m²

Therapy Week:________

Week:	Day1	Day 2	Day 8	Day 15
	VCR<br>Cisplatin	-<br>Cisplatin	VCR	VCR

I. Pre-Hydration: starting 12 hours prior to Cisplatin:

Glucose 5 % / NaCl 0,9 % 1:1 1000 – 1500 ml / m² / 12 h = ___________ ml

Per 500 ml: + 10 ml K Cl 7,45 % = ___________ ml
+ 6 ml Magnesium 20 % = ___________ ml
+ 10 ml Ca-Gluconat 10 % =
___________ ml

II. Vincristin 1,5 mg / m² iv-bolus = ___________ mg
Day 1, 8, 15

III. Mannitol 20 % 40 ml / m² 15 minutes short-infusion = ___________ ml
Immediately before the start of Cisplatin-infusion

IV. Cisplatin 30 mg / m² 180 minutes-infusion in 250 ml Na Cl 0,9 % = ___________ mg
Day 1 and 2

V. Post - Hydration: until 24 hours following Cisplatin:

Glucose 5 % / NaCl 0,9 % 1:1 2000 – 3000 ml / m² / 24 h = ___________ ml

Per 500 ml: + 10 ml K Cl 7,45 % = ___________ ml
+ 6 ml Magnesium 20 % = ___________ ml
+ 10 ml Ca-Gluconat 10 % =
___________ ml
+ 30 ml Mannitol 20 % = ___________ ml

VI. Supportive Care:
Mannitol 20 % 40 ml / m² as short term infusion = ___________ ml
If urine output falls below 2/3 of fluid input at 6 hourly registration of fluid balance.
Antiemetics:____________________________________ **= ___________ mg**
Starting 30‘ prior to chemotherapy

Steroids may impair the efficacy of Platinum compounds on glial cells, they should be used restrictively:
Dexamethasone 0,15 mg / kg iv-bolus = ___________ mg
If increased intracerebral pressure is manifest prior to or during therapy and shunting is not indicated.

Respect dose modifications due to toxicity and recommendations for supportive care (14.2.-3)

21.8. Documentation of Chemotherapy SIOP - LGG 2004

Documentation of chemotherapy

21.8.1 Induction
21.8.1.1. Vincristin/Carboplatin – week 1 - 24
21.8.1.2. Vincristin/Carboplatin/Etoposide – week 1 – 24

21.8.2. Consolidation
21.8.2.1. Regular protocol consolidation – week 25 – 54
21.8.2.2. Regular protocol consolidation – week 55 - 85
21.8.2.3. Alternative consolidation post allergy – week 1 - 30
21.8.2.4. Alternative consolidation post allergy – week 31 - 61

21.8.3. Documentation of toxicity

21.8.4. Response assessment

21.8.1.1. - Chemotherapy - Induction I

SIOP - LGG 2004
Page 1/1

National Coordinating Center:

Surname, Prename Pat.-No. Hospital Patient Identity No.
I______________________________________I I_I_I_I_I I__I__I__I__I __I I__I **I__I__I__I__I__I__I** I__I__I__I__I__I__I
date of birth

At start of chemotherapy:

Height: I__I__I__I cm **Weight:** I__I__I , I__I kg **Body surface area:** I__I , I__I__I m²

week	1	2	3	4	5	6	7	8	9	10	13	17	21	24
**Vincristin 1,5 mg/m²**	**V**	**V**	**V**	**V**	**V**	**V**	**V**	**V**	**V**	**V**	**V**	**V**	**V**	
**Carboplatin 550 mg/m²**	**C**			**C**			**C**			**C**	**C**	**C**	**C**	
														**MRI**

**week**	**date**	**Vincristin Dose (mg)**	**Carboplatin Dose (mg)**	**modification of the dose of: VCR**	**Carboplatin**	**Toxicity:** (Grade 3 or 4, type)
**1**	I__I__I__I__I__I__I	I__I , I__I__I	I__I__I__I , I__I	❐	❐	❐
2	I__I__I__I__I__I__I	I__I , I__I__I		❐	❐	❐
3	I__I__I__I__I__I__I	I__I , I__I__I		❐	❐	❐
**4**	I__I__I__I__I__I__I	I__I , I__I__I	I__I__I__I , I__I	❐	❐	❐
5	I__I__I__I__I__I__I	I__I , I__I__I		❐	❐	❐
6	I__I__I__I__I__I__I	I__I , I__I__I		❐	❐	❐
**7**	I__I__I__I__I__I__I	I__I , I__I__I	I__I__I__I , I__I	❐	❐	❐
8	I__I__I__I__I__I__I	I__I , I__I__I		❐	❐	❐
9	I__I__I__I__I__I__I	I__I , I__I__I		❐	❐	❐
**10**	I__I__I__I__I__I__I	I__I , I__I__I	I__I__I__I , I__I	❐	❐	❐
**13**	I__I__I__I__I__I__I	I__I , I__I__I	I__I__I__I , I__I	❐	❐	❐
**17**	I__I__I__I__I__I__I	I__I , I__I__I	I__I__I__I , I__I	❐	⑨	⑨
**21**	I__I__I__I__I__I__I	I__I , I__I__I	I__I__I__I , I__I	⑨	❐	❐
24	I__I__I__I__I__I__I			**MRI** (send report to nat. coordinator) (send images for central review)		

(Please, add documentation of toxicity for each cycle and response following MRI!)

Interruption of therapy: ❐ no ❐ yes, date I__I__I__I__I__I__I
reason: ❐ Progression ❐ Compliance
❐ Toxicity ❐ Allergy
❐ other:______________________

....

Date Signature

21.8.1.2. Chemotherapy - Induction II

SIOP - LGG 2004
Page 1/1

National Coordinating Center:

Surname, Prename I______________________________I Pat.-No. I_I_I_I_I Hospital I__I__I__I__I Patient Identity No. I__I **I__I__I__I__I__I__I** I__I__I__I__I__I
date of birth

At start of chemotherapy:

height: I__I__I__I cm **weight:** I__I__I,I__I kg **Body surface area:** I__I,I__I__I m²

week	1	2	3	4	5	6	7	8	9	10	13	17	21	24
**Vincristin 1,5 mg/m²**	V	V	V	V	V	V	V	V	V	V	V	V	V	
**Carboplatin 550 mg/m²**	C			C			C			C	C	C	C	
**Etoposide 100 mg/m²**	Ex3			Ex3			Ex3			Ex3				
														**MRI**

**week**	**date**	**Vincristin Dose(mg)**	**Carboplatin Dose (mg)**	**Etoposide Dose (mg)**	**modification of the dose of: V**	**C**	**E**	**Toxicity** (Grade 3 or 4, type)
**1**	I__I__I__I__I__I__I	I__I,I__I__I	I__I__I__I,I__I	d1 I__I__I__I,I__I<br>d2 I__I__I__I,I__I<br>d3 I__I__I__I,I__I	❒	❒	❒	❒
2	I__I__I__I__I__I__I	I__I,I__I__I			❒	❒	❒	❒
3	I__I__I__I__I__I__I	I__I,I__I__I			❒	❒	❒	❒
**4**	I__I__I__I__I__I__I	I__I,I__I__I	I__I__I__I,I__I	d1 I__I__I__I,I__I<br>d2 I__I__I__I,I__I<br>d3 I__I__I__I,I__I	❒	❒	❒	❒
5	I__I__I__I__I__I__I	I__I,I__I__I			❒	❒	❒	❒
6	I__I__I__I__I__I__I	I__I,I__I__I			❒	❒	❒	❒
**7**	I__I__I__I__I__I__I	I__I,I__I__I	I__I__I__I,I__I	d1 I__I__I__I,I__I<br>d2 I__I__I__I,I__I<br>d3 I__I__I__I,I__I	❒	❒	❒	❒
8	I__I__I__I__I__I__I	I__I,I__I__I			❒	❒	❒	❒
9	I__I__I__I__I__I__I	I__I,I__I__I			❒	❒	❒	❒
**10**	I__I__I__I__I__I__I	I__I,I__I__I	I__I__I__I,I__I	d1 I__I__I__I,I__I<br>d2 I__I__I__I,I__I<br>d3 I__I__I__I,I__I	❒	❒	❒	❒
**13**	I__I__I__I__I__I__I	I__I,I__I__I	I__I__I__I,I__I		❒	❒	❒	❒
**17**	I__I__I__I__I__I__I	I__I,I__I__I	I__I__I__I,I__I		❒	❒	❒	❒
**21**	I__I__I__I__I__I__I	I__I,I__I__I	I__I__I__I,I__I		❒	❒	❒	❒
24	I__I__I__I__I__I__I				**MRI** (send report to nat. coordinator) (send images for central review)			

Please, add documentation of toxicity for each cycle and response following MRI!

Interruption of therapy: ❒ no ❒ yes, date: I__I__I__I__I__I__I
reason: ❒ Progression ❒ Compliance
❒ Toxicity ❒ Allergy
❒ other:_______________

....
Date Signature

21.8.2.1. Chemotherapy – Protocol Consolidation Week 25 – 54

SIOP - LGG 2004
1/1

National Coordinating Center:

Surname, Prename I________________________________I Pat.-No. I_I_I_I_I Hospital I_I_I_I_I Patient Identity No. I__I **I__I__I__I__I__I__I__I** I__I__I__I__I__I
date of birth

height: I__I__I__I cm
weight: I__I__I,I__I kg
Body surface area: I__I,I__I__I m²

Vincristin 1,5 mg/m² iv-Bolus - day 1, 8, 15
Carboplatin 550 mg/m² 1 h iv - day 1

**week**	**25**	**31**	**37**	**43**	**49**	**54**
	55	61	67	73	79	85
	**VVV C**	**VVV C**	**VVV C**	**VVV C**	**VVV C**	**MRI**

**week**	**date**	**Vincristin Dose (mg)**	**Carboplatin Dose (mg)**	**modification of the dose of: V**	**C**	**toxicity:** (Grade 3 or 4, type)
**25**	I__I__I__I__I__I__I	I__I,I__I__I	I__I__I__I,I__I	❒	❒	❒
26	I__I__I__I__I__I__I	I__I,I__I__I		❒		❒
27	I__I__I__I__I__I__I	I__I,I__I__I		❒		❒
**31**	I__I__I__I__I__I__I	I__I,I__I__I	I__I__I__I,I__I	❒	❒	❒
32	I__I__I__I__I__I__I	I__I,I__I__I		❒		❒
33	I__I__I__I__I__I__I	I__I,I__I__I		❒		❒
**37**	I__I__I__I__I__I__I	I__I,I__I__I	I__I__I__I,I__I	❒	❒	❒
38	I__I__I__I__I__I__I	I__I,I__I__I		❒		❒
39	I__I__I__I__I__I__I	I__I,I__I__I		❒		❒
**43**	I__I__I__I__I__I__I	I__I,I__I__I	I__I__I__I,I__I	❒	❒	❒
44	I__I__I__I__I__I__I	I__I,I__I__I		❒		❒
45	I__I__I__I__I__I__I	I__I,I__I__I		❒		❒
**49**	I__I__I__I__I__I__I	I__I,I__I__I	I__I__I__I,I__I	❒	❒	❒
50	I__I__I__I__I__I__I	I__I,I__I__I		❒		❒
51	I__I__I__I__I__I__I	I__I,I__I__I		❒		❒
**53**	I__I__I__I__I__I__I			**MRI** (send report to nat. coordinator) (send images for central review)		

Interruption of therapy: ❒ No ❒ Yes, date: I__I__I . I__I__I . I__I
reason: ❒ Progression ❒ Compliance ❒ Toxicity ❒ Allergy ❒ other:________________________

Please, add documentation of toxicity for each cycle and response following MRI!

.... Date

.... Signature

21.8.2.2. Chemotherapy – Regular Consolidation Week 55 – 85

SIOP - LGG 2004
1/1

National Coordinating Center:

Surname, Prename I______________________________I Pat.-No. I_I_I_I_I Hospital I_I_I_I_I Patient Identity No. I__I I__I__I__I__I__I__I I__I__I__I__I__I
date of birth

height: I__I__I__I cm
weight: I__I__I,I__I kg
Body surface area: I__I,I__I__I m²

Vincristin 1,5 mg/m² iv-Bolus - day 1, 8, 15
Carboplatin 550 mg/m² 1 h iv - day 1

**week**	25	31	37	43	49	54
	**55**	**61**	**67**	**73**	**79**	**85**
	**VVV C**	**VVV C**	**VVV C**	**VVV C**	**VVV C**	**MRI**

**week**	**date**	**Vincristin Dose (mg)**	**Carboplatin Dose (mg)**	**modification of the dose of: V C**	**toxicity:** (Grade 3 or 4, type)
**55**	I__I__I__I__I__I__I	I__I,I__I__I	I__I__I__I,I__I	❒	❒
56	I__I__I__I__I__I__I	I__I,I__I__I		❒	❒
57	I__I__I__I__I__I__I	I__I,I__I__I		❒	❒
**61**	I__I__I__I__I__I__I	I__I,I__I__I	I__I__I__I,I__I	❒	❒
62	I__I__I__I__I__I__I	I__I,I__I__I		❒	❒
63	I__I__I__I__I__I__I	I__I,I__I__I		❒	❒
**67**	I__I__I__I__I__I__I	I__I,I__I__I	I__I__I__I,I__I	❒	❒
68	I__I__I__I__I__I__I	I__I,I__I__I		❒	❒
69	I__I__I__I__I__I__I	I__I,I__I__I		❒	❒
**73**	I__I__I__I__I__I__I	I__I,I__I__I	I__I__I__I,I__I	❒	❒
74	I__I__I__I__I__I__I	I__I,I__I__I		❒	❒
75	I__I__I__I__I__I__I	I__I,I__I__I		❒	❒
**79**	I__I__I__I__I__I__I	I__I,I__I__I	I__I__I__I,I__I	❒	❒
80	I__I__I__I__I__I__I	I__I,I__I__I		❒	❒
81	I__I__I__I__I__I__I	I__I,I__I__I		❒	❒
**85**	I__I__I__I__I__I__I			**MRI** (send report to nat. coordinator) (send images for central review)	

Interruption of therapy: ❒ No ❒ Yes, date: I__I__I__I__I__I__I
reason: ❒ Progression ❒ Compliance
❒ Toxicity ❒
ergy
❒ other:____________________________

Please, add documentation of toxicity for each cycle and response following MRI!

....
Date Signature

21.8.2.3. Chemotherapy – Alternative Consolidation Week 1 – 30

SIOP - LGG 2004
1/1

National Coordinating Center:

Surname, Prename I_________________________________I
Pat.-No. I_I_I_I_I
Hospital I_I_I_I
Patient Identity No. I__I **I__I__I__I__I__I__I__I** I__I__I__I__I__I
date of birth

height I__I__I__I cm
weight: I__I__I,I__I kg
Body surface area: I__I,I__I__I m²

V = Vincristin 1,5 mg/m² iv-Bolus - day 1, 8, 15
Cyc = Cyclophosphamide 1500 mg/m² 1 h iv, day 1
Cis = Cisplatin 30 mg/m² 3 h iv - day 1 + 2

week					
**1**	**30**	**7**	**13**	**19**	
**25**		31	37	43	
49		55			61
**VVV Cyc**	**MRI**	**VVV Cis**	**VVV Cyc**	**VVV Cis**	**MRI**

week	date	Vincristin Dose (mg)	Cyc / Cis Dose (mg)	modification of the dose of: V Cyc/Cis	Toxicity: (Grade 3 or 4, type)
**1**	I__I__I__I__I__I__I	I__I,I__I__I	**1** Cyc I__I__I__I__I,I__I	❐	❐
2	I__I__I__I__I__I__I	I__I,I__I__I		❐	❐
3	I__I__I__I__I__I__I	I__I,I__I__I		❐	❐
**7**	I__I__I__I__I__I__I	I__I,I__I__I	**7** Cis day 1: I__I__I,I__I	❐	❐
8	I__I__I__I__I__I__I	I__I,I__I__I	day 2: I__I__I,I__I	❐	❐
9	I__I__I__I__I__I__I	I__I,I__I__I		❐	❐
**13**	I__I__I__I__I__I__I	I__I,I__I__I	**13** Cyc I__I__I__I__I,I__I	❐	❐
14	I__I__I__I__I__I__I	I__I,I__I__I		❐	❐
15	I__I__I__I__I__I__I	I__I,I__I__I		❐	❐
**19**	I__I__I__I__I__I__I	I__I,I__I__I	**19** Cis day 1: I__I__I,I__I	❐	❐
20	I__I__I__I__I__I__I	I__I,I__I__I	day 2: I__I__I,I__I	❐	❐
21	I__I__I__I__I__I__I	I__I,I__I__I		❐	❐
**25**	I__I__I__I__I__I__I	I__I,I__I__I	**25** Cyc I__I__I__I__I,I__I	❐	❐
26	I__I__I__I__I__I__I	I__I,I__I__I		❐	❐
27	I__I__I__I__I__I__I	I__I,I__I__I		❐	❐
**30**	I__I__I__I__I__I__I			**MRI** (send report to nat. coordinator) (send images for central review)	

Please, add documentation of toxicity for each cycle and response following MRI!

Interruption of therapy: ❐ No ❐ Yes, date I__I__I__I__I__I__I
reason: ❐ Progression ❐ Compliance
❐ Toxicity ❐ Allergy
❐ other:____________________

.... Date

.... Signature

21.8.2.4. Chemotherapy – Alternative Consolidation Week 31 - 61

SIOP - LGG 2004
1/1

National Coordinating Center:

Surname, Prename I_______________________________________I Pat.-No. I_I_I_I_I Hospital I_I_I_I Patient Identity No. I__I **I__I__I__I__I__I__I** I__I__I__I__I__I
date of birth

height I__I__I__I cm
weight: I__I__I,I__I kg
Body surface area: I__I,I__I__I m²

V = Vincristin 1,5 mg/m² iv-Bolus - day 1, 8, 15
Cis = Cisplatin 30 mg/m² 3 h iv - day 1 + 2
Cyc = Cyclophosphamide 1500 mg/m² 1 h iv, day 1

**week**	1		7	13	19	
	25	30	**31**	**37**	**43**	
	**49**		**55**			**61**
	**VVV Cyc**	**MRI**	**VVV Cis**	**VVV Cyc**	**VVV Cis**	**MRI**

**week**	**date**	**Vincristin Dose (mg)**	**Cyc / Cis Dose (mg)**	**modification of the dose of: V Cyc/Cis Toxicity:** (Grade 3 or 4, type)
**31**	I__I__I__I__I__I__I	I__I,I__I__I	**31** Cis day 1: I__I__I,I__I	❐ ❐ ❐
32	I__I__I__I__I__I__I	I__I,I__I__I	day 2: I__I__I,I__I	❐ ❐ ❐
33	I__I__I__I__I__I__I	I__I,I__I__I		❐ ❐
**37**	I__I__I__I__I__I__I	I__I,I__I__I	37 Cyc I__I__I__I__I,I__I	❐ ❐ ❐
38	I__I__I__I__I__I__I	I__I,I__I__I		❐ ❐ ❐
39	I__I__I__I__I__I__I	I__I,I__I__I		❐ ❐
**43**	I__I__I__I__I__I__I	I__I,I__I__I	**43** Cis day 1: I__I__I,I__I	❐ ❐ ❐
44	I__I__I__I__I__I__I	I__I,I__I__I	day 2: I__I__I,I__I	❐ ❐ ❐
45	I__I__I__I__I__I__I	I__I,I__I__I		❐ ❐
**49**	I__I__I__I__I__I__I	I__I,I__I__I	49 Cyc I__I__I__I__I,I__I	❐ ❐ ❐
50	I__I__I__I__I__I__I	I__I,I__I__I		❐ ❐ ❐
51	I__I__I__I__I__I__I	I__I,I__I__I		❐ ❐
**55**	I__I__I__I__I__I__I	I__I,I__I__I	**55** Cis day 1: I__I__I,I__I	❐ ❐ ❐
56	I__I__I__I__I__I__I	I__I,I__I__I	day 2: I__I__I,I__I	❐ ❐ ❐
57	I__I__I__I__I__I__I	I__I,I__I__I		❐ ❐
**61**	I__I__I__I__I__I__I			**MRI** (send report to nat. coordinator) (send images for central review)

Please, add documentation of toxicity for each cycle and response following MRI!

Interruption of therapy: ❐ No ❐ Yes, date I__I__I__I__I__I__I
reason: ❐ Progression ❐ Compliance
❐ Toxicity ❐ Allergy
❐ other:____________________________

....
Date Signature

21.8.3. Documentation of Toxicity

(abbreviated version of Common Toxicity Criteria for documentation)

SIOP - LGG 2004
Page 1/2

Patient:______________________________ Date of birth: I__I__I.I__I__I.I__I__I ID-Nr. ________________

Please complete for every course of chemotherapy:

❐ Induction Vincristin/Carboplatin ❐ Consolidation Vincristin/Carboplatin **week: I__I__I**
❐ Induction Vincristin/Carboplatin/Etoposide ❐ Consolidation Vincristin/Cisplatin from I_I_I..I_I_I.I_I_I
❐ Consolidation Vincristin/Cyclophosphamide to I_I_I.I_I_I..I_I_I

Please tick the appropriate field for each parameter (maximal toxicity). For more details see addendum 21.11.

**Degree of toxicity**	**0**	**I**	**II**	**III**	**IV**
**Blood**					
Hemoglobin (g/l)	WNL	<LLN - 100	≥80 - <100	≥65 - <80	< 65
Leukocytes (mm³)	WNL	<LLN - 3000	≥2000 - <3000	≥1000 - <2000	< 1000
Granulocytes (mm³)	WNL	<LLN - 1500	≥1000 - <1500	≥500 - <1000	< 500
Platelets (mm³)	WNL	<LLN - 75000	≥50000 - <75000	≥10000 - <50000	< 10000
**Auditory/Hearing**					
Inner ear / hearing	normal	hearing loss on audiometry only	Tinnitus	Tinnitus, correct-able with hearing aid	severe hearing loss, not correctable
Bilateral hearing loss (Brock et al, 1991)	< 40 dB at all frequencies	> 40 dB at 8000 Hz only	> 40 dB at 4000 Hz only	> 40 dB at 2000 Hz only	> 40 dB at 1000 Hz only
**Neurology**					
Neuropathy-cranial	absent	-	present, not inter-fering with activi-ties	present, interfering with activities	life-threatening, dis-abling
Neuropathy-motor	normal	subjective weak-ness	mild objective weakness	objective weakness	paralysis
Neuropathy-sensory	normal	loss of deep tendon reflexes or paresthesia	objective sensory loss or paresthesia	functionally relevant sensory loss or paresthesia	permanent sensory loss that interferes with function
Seizure(s)	non	-	seizure(s) selfli-mited,conscious-ness preserved	seizure(s) with al-tered conscious-ness	Prolonged seizure(s) of any type (e.g. status epilepticus)
Abdominal pain or cramping	non	mild pain, not interfering with function	moderate pain not interfering with activities	severe pain interfering with activities of daily living	disabling
**Infection**					
Infection	none	mild	moderate	severe	life-threatening, septic shock
Fever	non	38,0 - 39,0°C	39,1 - 40,0 °C	> 40 °C for < 24 h	> 40°C for > 24 h
**Renal**					
Hematuria	none	microscopic only	intermittent gross bleeding, no clots	persistent gross bleeding or clots	open surgery or necro-sis or deep bladder ulceration
Creatinine (x ULN)	WNL	>ULN - 1,5	> 1,5 - 3,0	> 3,0 - 6,0	> 6,0
Proteinuria (g/24 hrs)	normal or < 0,15	1+ or 0,15-1,0	2+ to 3+,;1,0-3,5	4+ ; > 3,5	nephrotic syndrome
Creatinine-clearance (ml/min + 1,73 m²)	≥ 90	60 - 89	40 - 59	20 - 39	≤ 19
**Nausea/Vomiting**					
Nausea	none	Nausea, but still able to eat	oral intake signifi-cantly decreased	no significant intake, requiring IV fluids	-
Vomiting (number of episodes/24 h)	none	1	1-5	≥ 6	requiring parenteral nutrition
**Constitutional Symptoms**					
Weight loss	< 5%	5% - < 10%	10% - <20%	≥ 20%	-
Anorexia	none	loss of appetite	oral intake signifi-cantly decreased	requiring IV fluids	requiring feeding tube or parenteral nutrition

Patient:____________________________________

Alopecia	normal	mild hair loss	pronounced hair loss	-	-
Fatigue	none	increased fatigue over baseline	moderate, causing difficulty	Severe, loss of ability to perform some activities	bedridden or disabling
**Allergic reaction / hypersensitivity**	none	transient rash, drug fever < 38°C	urticaria, drug fever ≥ 38°C and/or asymptomatic brochospasm	symptomatic bronchospasm with or without urticaria, allergy-related edema/angioedema	anaphylaxis
**Gastrointestinal**					
Mucositis	none	eythema of the mucosa	patchy pseudomembranous reaction	confluent pseudomembranous reaction	necrosis or deep ulceration; bleeding not induced by minor trauma or abrasion
Stomatitis/Pharyngitis	none	painless ulcers, erythema or mild soreness in the absence of lesions	painful erythema, edema or ulcers, but can eat or swalllow	painful erythema, edema or ulcers requiring IV hydration	severe ulceration or requires parenteral or enteral nutritional support or prophylactic intubation
Diarrhea	none	increase of <4 stools/day over pre-treatment	increase of 4-6 stools/day or nocturnal stools	increase of ≥7 stools/day or incontinence requiring IV hydration	physiologic consequences requiring intensive care or hemodynamic collapse
Constipation	none	requiring stool softener or dietary modification	requiring laxatives	obstipation requiring manual evacuation or enema	obstruction or toxic megacolon
**Dermatology/Skin**					
Radiation dermatitis	none	faint erythema or dry desquamation	moderate to brisk erythema or patchy moist desquamation, confined to skin folds and creases; moderate edema	confluent moist desquamation ≥1,5 cm diameter and not confined to skin folds; pitting edema	skin necrosis or ulceration of full thickness dermis; may include bleeding not induced by minor trauma or abrasion
**Hepatic**					
Bilirubin (xULN)	WNL	> ULN - 1,5	> 1,5 - 3,0	> 3,0 - 10,0	> 10,0
SGOT/SGPT (xULN)	WNL	> ULN - 2,5	> 2,5 - 5,0	> 5,0 - 20,0	> 20,0
**Pulmonary**					
Dyspnea	normal	-	dyspnea on exertion	dyspnea at normal leves of activity	dyspnea at rest or requiring ventilator support
**Cardiovascular**					
Cardiac left ventricular function	normal	asymptomatic decline of resting ejection fraction of ≥ 10% but <20% of baseline value; shortening fraction ≥ 24% but <30%	asymptomatic, but resting ejection fraction below LLN for laboratory or decline of resting ejection fraction ≥20% of baseline value; <24% shortening fraction	CHF responsive to treatment	severe or refractory CHF or requiring intubation
LV-EF Echocardiography	> 30%	26% - 30%	21% - 25%	16% - 20%	< 16%

Date Stamp Signature

21.8.4. Response Assessment SIOP - LGG 2004

National Coordinating Center:

Surname, Prename Pat.-No. Hospital Patient Identity No.
I____________________________________I I_I_I_I_I I__I__I__I__I I__I **I__I__I__I__I__I__I__I** I__I__I__I__I__I
date of birth

Tumorresponse: ❒ week 24 / ❒ week 54 / ❒ week 85 / ❒ ...... months / ❒ ..... years

**Date of response assessment:**	**Neurologic response**<br>1 = better<br>2 = existing unchanged<br>3 = progression of existing symptoms<br>4 = emergence of new symptoms<br>9 = not done	**Ophtalmological finding (vision)**<br>1 = better<br>2 = stable<br>3 = worse<br>4 = not applicable<br>9 = not done	**Radiological finding**<br>CR=complete remission<br>PR= partial remission, residual size < 50%<br>OR=objective remission,residual size 50-75%<br>SD=stable disease, tumor size reduction of <25%, but no sign of PD<br>PD=progressive disease, tumor size increase of > 25%	
I_I_I . I_I_I . I_I_I	I___I	I___I	I__I__I	size of tumor (cm x cm x cm)<br>I__I__I x I__I__I x I__I__I<br>(Include report for national data center!)

MRI-images sent for central review? ❒ yes ❒ no ❒ arranged

Allergy

❒ **no** ❒ **yes:** Date of first symptoms: I__I__I . I__I__I . I__I__I__I__I
in therapy week I__I__I

Reaction to:
❒ Carboplatin
❒ Cyclophosphamid
❒ Cisplatin
❒ Actinomycin D
❒ VP 16
❒ _________________

Symptoms of allergy:
❒ itching (I°)
❒ (mild) rash (I°)
❒ urticaria (II°)
❒ bronchospasm (III°)
❒ anaphylaxia (IV°)
❒ other: ___________________

Management of allergy:
____
____
____
____
____

Treatment consequence: ❒ dose modified ❒ interruption of therapy
❒ change of therapy to_________________ ❒ other:_____________________

Remarks (Please detail response):

______________________ ______________ __________________________
stamp **date** **signature**

21.9.1. Radiotherapy data form SIOP - LGG 2004

Patient data - Maximal acute morbidity – during RT Page 1/11

PATIENT - DATA Study-ID I____________________I SIOP-ID I________________________I

Surname ______________________ Prename__________________ date of birth I__I__I . I__I__I . I__I__I

Sex I__I (male= 1, female= 2) Diagnosis____

Treatment Center ____ (Code number :_____________)

Radiotherapy/primary treatment I__I (yes=1,no=2), **after failure of CT I__I** (yes=1, no=2)

Start of treatment I__I__I . I__I__I . I__I__I **End** of treatment I__I__I . I__I__I . I__I__I

For Brachytherapy see page 8/11,use page 1 and 2 (toxicity data) also for brachytherapy

TARGET VOLUME	*Total dose (Gy)*	*Single dose (Gy)*	*Fractions/week*
**Tumor site**			

Interruption of treatment I__I (yes= 1, no= 2) Discontinuation of treatment I__I (yes= 1, no= 2)

Interruption / interval: from I__I__I . I__I__I . I__I__I to I__I__I . I__I__I . I__I__I = I__I__I days

Reason: ____

Please fill in Grade "0", if there is no toxicity!

MAXIMAL ACUTE MORBIDITY DURING RADIOTHERAPY / CTCAE v. 3.0*					
CTCAE v. 3.0	**1**	**2**	**3**	**4**	**Grade**
**Neurotoxicity**					
Headache	mild pain not interfering with function	moderate pain, pain or analgesics interfering with function, but not interfering with activity in daily life (ADL)	severe pain, pain or analgesics severely interfering with ADL	disabling	☐
Nausea/Vomiting	mild	moderate	severe	life-threatening, disabling	☐
Seizure	-	one brief generalized seizure, seizure(s) well controlled by anticonvulsants or infrequent focal motor seizures not interfering with ADL	seizure(s) in which consciousness is altered, poorly controlled seizure disorder, with breakthrough generalized seizures despite medival intervention	seizures of any kind which are prolonged, repetitive or difficult to control (e.g. status epilepticus, intractable epilepsy)	☐
Somnolence	-	somnolence or sedation interfering with function, but not interfering with ADL	obtundation or stupor; difficult to arouse; interfering with ADL	coma	☐
**Infections**	mild	moderate	severe	life-threatening, disabling	☐
Please specify	1 bacterial, 2 viral, 3 fungal, 4 combination, 5 others, 6 not isolated				☐
**Skin**	faint erythema or dry desquamation	moderate to brisk erythema, patchy moist desquamation, mostly confined to skin foods and creases, moderate edema	moist desquamation other to skin folds and creases, bleeding induced by minor trauma or abrasion	skin necrosis or ulceration of full thickness dermis, spontaneous bleeding from involved site	☐
**Mucosa**	erythema of the mucosa	patchy ulcerations or pseudomembranes	confluent ulcerations or pseudomembranes, bleeding with minor trauma	tissue necrosis, significant spontaneous bleeding, life threatening consequences	☐
**External ear**	external otitis with erythema or dry desquamation	external otitis with moist desquamation, edemy, enhanced cerumen or discharge, tympanic membrane perforation, tympanostomy	external otitis with mastoiditis, stenosis or osteomyelitis	necrosis of soft tissue or bone	☐

Any other toxicity:____

Date: ________________________ **Signature/Stamp:**____________________________________

! Address/Fax to National Radiotherapy and to National Data Centre within 1 month !

* modified January 2006

21.9.2. Radiotherapy data form SIOP - LGG 2004
Maximal acute morbidity – end of RT Page 2/11

Surname, prename, date of birth Study-ID SIOP-ID

I____I I_________________I I________________________I

Please fill in Grade "0", if there is no toxicity!

MAXIMAL ACUTE MORBIDITY AT THE END OF RADIOTHERAPY / CTCAE v. 3.0*					
CTCAE v. 3.0	**1**	**2**	**3**	**4**	**Grade**
**Neurotoxicity**					
Headache	mild pain not interfering with function	moderate pain, pain or analgesics interfering with function, but not interfering with activity in daily life (ADL)	severe pain, pain or analgesics severely interfering with ADL	disabling	☐
Nausea/Vomiting	mild	moderate	severe	life-threatening, disabling	☐
Seizure	-	one brief generalized seizure, seizure(s) well controlled by anticonvulsants or infrequent focal motor seizures not interfering with ADL	seizure(s) in which consciousness is altered, poorly controlled seizure disorder, with breakthrough generalized seizures despite medival intervention	seizures of any kind which are prolonged, repetitive or difficult to control (e.g. status epilepticus, intractable epilepsy)	☐
Somnolence	-	somnolence or sedation interfering with function, but not interfering with ADL	obtundation or stupor; difficult to arouse; interfering with ADL	coma	☐
**Infections**	mild	moderate	severe	life-threatening, disabling	☐
Please specify	1 bacterial, 2 viral, 3 fungal, 4 combination, 5 others, 6 not isolated				☐
**Skin**	faint erythema or dry desquamation	moderate to brisk erythema, patchy moist desquamation, mostly confined to skin foods and creases, moderate edema	moist desquamation other to skin folds and creases, bleeding induced by minor trauma or abrasion	skin necrosis or ulceration of full thickness dermis, spontaneous bleeding from involved site	☐
**Mucosa**	erythema of the mucosa	patchy ulcerations or pseudomembranes	confluent ulcerations or pseudomembranes, bleeding with minor trauma	tissue necrosis, significant spontaneous bleeding, life threatening consequences	☐
**External ear**	external otitis with erythema or dry desquamation	external otitis with moist desquamation, edemy, enhanced cerumen or discharge, tympanic membrane perforation, tympanostomy	external otitis with mastoiditis, stenosis or osteomyelitis	necrosis of soft tissue or bone	☐

Any other toxicity: ____

Date: ________________________ **Signature/Stamp:**_______________________________________

! Address/Fax to National Radiotherapy and to National Data Centre within 1 month !

* modified January 2006

21.9.3. Radiotherapy data form
Myelotoxicity during craniospinal irradiation

SIOP - LGG 2004
Page 3/11

Surname, prename, date of birth Study-ID SIOP-ID

I_______________________________________I I____________________I I________________________I

MAXIMAL MYELOTOXICITY DURING RADIOTHERAPY
(only to be completed after craniospinal irradiation)

	Date<br>D D M M Y Y Y Y	Value
Blood count at start of **treatment**<br>WBC	└┴┘ └┴┘ └┴┴┴┘	└┴┴┴┴┴┘ ($x10^9/l$)
Platelets		└┴┴┴┴┴┘ ($x10^9/l$)
Blood count nadir during **treatment**<br>WBC	└┴┘ └┴┘ └┴┴┴┘	└┴┴┴┴┴┘ ($x10^9/l$)
Platelets-Nadir	└┴┘ └┴┘ └┴┴┴┘	└┴┴┴┴┴┘ ($x10^9/l$)
Blood count at the end of **treatment**<br>*WBC*	└┴┘ └┴┘ └┴┴┴┘	└┴┴┴┴┴┘ ($x10^9/l$)
Platelets		└┴┴┴┴┴┘ ($x10^9/l$)
Administration of growth factors	Yes ❐ No ❐	
Transfusion/Erythrocytes	Yes ❐ No ❐	
Transfusion/Platelets	Yes ❐ No ❐	

Address/Fax to National Radiotherapy and to National Data Centre within 1 month

Date: Signature/Stamp:

21.9.4. Radiotherapy data form — SIOP - LGG 2004
Treatment technique – primary tumor — Page 4/11

Surname, prename, date of birth Study-ID SIOP-ID
I____I I____________________I I________________________________I

TREATMENT TECHNIQUE

TUMOR SITE

Linear accelerator (energy) ______ MV CO-60 I__ I(yes= 1,no= 2)

CT-scan-assisted plan I__I (yes= 1,no= 2) 3-D treatment planning I__I (yes= 1,no= 2)

IMRI I__I (yes= 1,no= 2) MR for treatment planning I__I (yes= 1,no= 2)

Lat.opp.fields I__I (yes= 1,no= 2) Multi-field technique I__I (yes= 1,no= 2)

Wedge filters : I__I (yes= 1,no= 2) Rotation I__I (yes= 1,no= 2)

Dose/Reference-point_____Gy max._____% Min._____% „hot spot“ ______%

Image fusion (MR /CT-scan) for treatment planning: I__I (yes=1,no=2)
Protons: I__I (yes= 1,no= 2)

Dose to critical organs (Dose - volume histograms) in Gy

Organ	***maximum***	***minimum***	***mean***
Pituitary gland			
Optic chiasm			
Hypothalamus			
Brain stem			
Left optic nerve			
Right optic nerve			
Left eye lens			
Right eye lens			
Left inner ear			
Right inner ear			

POSITIONING AIDS

Individual, conventional face mask: I__I (yes= 1, no= 2)

Rigid head fixation using face mask: I__I (yes= 1, no= 2)

Stereotactic immobilization device (i.e. Gill-Thomas-Cosman ring): I__I (yes= 1, no= 2)

Others : I__I (yes= 1, no= 2)

Others / detail ____

REMARKS:

Address/Fax to National Radiotherapy and to National Data Centre within 1 month

Date: Signature/Stamp:

21.9.5. Radiotherapy data form — SIOP - LGG 2004
Dose prescription – craniospinal axis — Page 5/11

Surname, prename, date of birth I______________________I Study-ID I______________I SIOP-ID I________________I

DOSE PRESCRIPTION / CRANIOSPINAL AXIS

Total Dose / fractionation

Target volume	Total dose	Gy	Single dose	Gy	Fractions / day	Treatment days
Whole brain	⌴⌴ · ⌴	Gy	⌴⌴ · ⌴	Gy	⌴⌴	⌴⌴
Spinal canal	⌴⌴ · ⌴	Gy	⌴⌴ · ⌴	Gy	⌴⌴	⌴⌴
Boost metastatic deposits	⌴⌴ · ⌴	Gy	⌴⌴ · ⌴	Gy	⌴⌴	⌴⌴
Boost tumor site	⌴⌴ · ⌴	Gy	⌴⌴ · ⌴	Gy	⌴⌴	⌴⌴

Dose to critical organs (Dose - volume histograms) in Gy

Organ	*maximum*	*minimum*	*mean*
Pituitary gland			
Optic chiasm			
Hypothalamus			
Brain stem			
Left optic nerve			
Right optic nerve			
Left eye lens			
Right eye lens			
Left inner ear			
Right inner ear			

Address/Fax to National Radiotherapy and to National Data Centre within 1 month

Date: Signature/Stamp:

21.9.6. Radiotherapy data form — SIOP - LGG 2004
Treatment technique – craniospinal RT — Page 6/11

Surname, prename, date of birth Study-ID SIOP-ID
I_______________________________________I I____________________I I________________________I

TREATMENT TECHNIQUE : CRANIOSPINAL RADIOTHERAPY

I. "HELMET - TECHNIQUE"

Start of RT |__|__| . |__|__| . |__|__| End of RT |__|__| . |__|__| . |__|__| **DD/MM/YY**

Linear accelerator (energy) I__I__I MV

Lateral, isocentric opposed fields I__I (yes= 1, no= 2)

If 2, further details:____

POSITIONING AIDS

Individual face mask: I__I (yes= 1, no= 2)

Vacuum pillows: I__I (yes= 1, no= 2)

Immobilization cast: I__I (yes= 1, no= 2)

Others: ____

Prone: I__I Supine: I__I

II. SPINAL AXIS

Start of RT |__|__| . |__|__| . |__|__| End of RT |__|__| . |__|__| . |__|__| **DD/MM/YY**

Linear accelerator (energy): I__I__I MV

Dose at reference point for upper field: I__I__I cm

Dose at reference point for lower field: I__I__I cm

SSD : I__I__I__I__I cm

Maximum dose: I__I__I , I__I Gy

Minimum dose I__I__I , I__I Gy

Electron fields: I__I (yes= 1, no= 2)

Energy : I__I__I MeV

Dose specification : I__I__I % Isodose (Electron-Field)

POSITIONING AIDS

Individual face mask: I__I (yes= 1, no= 2)

Vacuum pillows: I__I (yes= 1, no= 2)

Immobilization cast: I__I (yes= 1, no= 2)

Others: ____

Prone: I__I Supine: I__I

Address/Fax to National Radiotherapy and to National Data Centre within 1 month

Date: Signature/Stamp:

21.9.7. Radiotherapy data form
Radiotherapy of metastatic deposits

SIOP - LGG 2004
Page 7/11

Surname, prename, date of birth | Study-ID | SIOP-ID

I____I I____________________I I_________________________I

TUMOR SITE

METASTATIC DEPOSITS (Localisation) ____

(Duplicate this form according to number of irradiated sites, if necessary)

Start of RT |__|__| . |__|__| . |__|__| End of RT |__|__| . |__|__| . |__|__| **DD/MM/YY**

Linear accelerator (energy) I__I__I MV

CT-scan-assisted plan I__I (yes= 1, no= 2)

3-D treatment planning I__I (yes= 1, no= 2)

MR for planning I__I (yes= 1, no= 2)

IMRI I__I (yes= 1, no= 2)

Image fusion (MR /CT-scan) for treatment planning: I__I (yes= 1, no= 2)

Lat.opp.fields I__I (yes= 1, no= 2)

Multi-field technique I__I (yes= 1, no= 2)

Wedge filters : I__I (yes= 1, no= 2)

Dose/Reference-point I__I__I , I__I Gy

max. I__I__I__I , I__I % Min. I__I__I__I , I__I % "hot spot" I__I__I__I%

POSITIONING AIDS

Individual face mask: I__I (yes= 1, no= 2)

Vacuum pillows: I__I (yes= 1, no= 2)

Immobilization cast: I__I (yes= 1, no= 2)

Others: ____

Prone: I__I Supine: I__I

Address/Fax to National Radiotherapy and to National Data Centre within 1 month

Date: Signature/Stamp:

21.9.8. Brachytherapy data form **SIOP - LGG 2004**
Technique / dose prescription **Page 8/11**

Surname, prename, date of birth Study-ID SIOP-ID
I_____________________________________I I___________________I I______________________I

Sex I__I (male= 1, female= 2).

Diagnosis____

Treating Centre __________________________(Code number :____________)

Brachytherapy as primary treatment I__I (yes= 1, no= 2),

Brachytherapy after failure of CT: I__I (yes= 1, no= 2)

Date of diagnosis : |__|__| . |__|__| . |__|__| **DD/MM/YY**

Location / extent of tumor :____

Biopsy : I__I (yes= 1, no= 2)

Isotope :______________________

Permanent : I__I (yes= 1, no= 2) temporary : I__I (yes= 1, no= 2)

Implant of seeds : |__|__| . |__|__| . |__|__| **DD/MM/YY**

Removal of seeds |__|__| . |__|__| . |__|__| **DD/MM/YY**

Total dose to tumor margin : : | ___ | ___ | ___ | . | ___ | ___ | Gy

Dose rate : | ___ | ___ | . | ___ | cGy per hour

Perioperative complications : I__I (yes= 1, no= 2)

If yes ; which :_______________________________________

DESCRIPTION OF TECHNIQUE

REMARKS:

Address/Fax to National Radiotherapy and to National Data Centre within 1 month

Date: Signature/Stamp:

21.9.9. Radiotherapy data form — Tumor response

SIOP - LGG 2004
Page 9/11

Surname, prename, date of birth Study-ID SIOP-ID
I_____________________________________I I___________________I I_______________________I

Type of radiotherapy

External radiotherapy : I__I **Brachytherapy : I__I**

Date of MRI :I__I__I . I__I__I . I__I__I DD/MM/YY

Please tick
MRI 3 months after end of Radiotherapy / Brachytherapy I__I
MRI 6 months after end of Radiotherapy / Brachytherapy I__I
MRI 12 months after end of Radiotherapy / Brachytherapy I__I
MRI I__I__I months after end of Radiotherapy / Brachytherapy I__I
(duplicate these forms as necessary)

**Date of response assessment:**	**Neurologic response**<br>1 = better<br>2 = existing unchanged<br>3 = progression of existing symptoms<br>4 = emergence of new symptoms<br>9 = not done	**Ophtalmological finding (vision)**<br>1 = better<br>2 = stable<br>3 = worse<br>4 = not applicable<br>9 = not done	**Radiological finding**<br>CR=complete remission<br>PR= partial remission, residual size < 50%<br>OR=objective remission, residual size 50-75%<br>SD=stable disease, tumor size reduction of <25%, but no sign of PD<br>PD=progressive disease, tumor size increase of > 25%	
I_I_I . I_I_I . I_I_I	I___I	I___I	I__I__I	size of tumor (cm x cm x cm)<br>I__I__I x I__I__I x I__I__I<br>(Include report for national data center!)

MRI-images sent for central review? ❐ yes ❐ no ❐ arranged

Comments: ____

____

____

Address/Fax to National Radiotherapy and to National Data Centre within 1 month

Date: Signature/Stamp:

21.9.10. Radiotherapy data form **SIOP - LGG 2004**
Late effects of radiotherapy **Page 10/11**

Surname, prename, date of birth Study-ID SIOP-ID
I____I I____________________I I________________________I

LATE EFFECTS OF RADIOTHERAPY / LENT – SOMA Score Criteria (yearly)

1. year ❐ 2. year ❐ 3. year ❐ 4. year ❐ 5.year ❐
(duplicate this form according to time schedule)

Skin	Grade 0	Grade 1	Grade 2	Grade 3	Grade 4
1. Alopecia (scalp)	Normal hair growth	Thinning	Patchy, permanent	Complete, permanent	-----
2. Pigmentation change	None	Transitory, slight	Permanent, marked	-----	------
3. Teleangiectasia	None	Minor	Moderate (< 50% of irradiated skinarea)	Gross (> 50% of irradiated skinarea)	------
4. Fibrosis / Scar	None	Present , Assymptomatic	Symptomatic	Secondary dysfunction	Total dysfunction
5. Ulcer / necrosis	None	Epidermal only	Dermal ❑	Subcutaneous	Bone exposed

Please fill in the corresponding grading number

1. Alopecia: ❐ **2. Pigmentation** ❐ **3. Teleangiecasia** ❐

4. Fibrosis /scar ❐ **5. Ulcer / necrosis** ❐

Mucosa (Oral / Pharyngeal)	Grade 0	Grade 1	Grade 2	Grade 3	Grade 4
1. Integrity of mucosa	Normal	Patchy atrophy or teleangiectasia	Diffuse atrophy or teleangiectasia, superficial ulcer	Deep ulcer no bone or cartilage exposure	Deep ulcer with bone or cartilage exposure
2. Dysphagia	Normal	Difficulties eating solid food	Difficulties eating soft food	Can take liquids only	Totally unable to swallow

Please fill in the corresponding grading number

1. Integrity of mucosa ❐ **2. Dysphagia** ☐

Address/Fax to National Radiotherapy and to National Data Centre within 1 month

Date: Signature/Stamp:

21.9.11. Radiotherapy data form — SIOP - LGG 2004
Relapse form — **Page 11/11**

Surname, prename, date of birth Study-ID SIOP-ID
I______________________________________I I___________________I I_______________________I

Type of previous radiotherapy

External: I__I **Brachytherapy :** I__I

Date of diagnosis of relapse / progressive disease I__I__I . I__I__I . I__I__I **DD/MM/YY**

- ***LOCAL RELAPSE LOCATION (ANY RELAPSE WITHIN IPSILATERAL HEMISHERE / SAME SUPRATENTIORIAL / INFRATENTORIAL / SPINAL COMPARTMENT)***

The relapse is within the primary tumor site: I__I (yes= 1, no= 2)

If No ↓

Field margin I__I (yes= 1, no= 2)

In field growing out field I__I (yes= 1, no= 2)

Beyond field margin I__I (yes= 1, no= 2)

If yes :
Distance of relapse to primary tumor (at start of radiotherapy) ____cm____mm
(minimum edge to edge distanc)

Relapse is in the └┴┘ % isodose

Cumulative total dose at relapse: └┴┘ Gy

- ***DISTANT RELAPSE LOCATION (ANY RELAPSE TO OTHER COMPARTMENT)***

Initial tumor location (please specify):______________

Site of relapse (please specify): __________________

Address/Fax to National Radiotherapy and to National Data Centre within 1 month

Date: Signature/Stamp:

21.10. Histopathology Report SIOP - LGG 2004

According to details of national arrangements pathologic material of those children entered into the randomized trial has to be sent for a reference report. This report has to be present at randomisation.
It is recommended that central pathologic review should be instituted for all children with low grade glioma following national policies.
The usual forms can be used within the national study groups, but a copy of the reports should be sent to the national data/coordinating center.

21.11. Common Toxicity Criteria SIOP - LGG 2004
Classification of acute side-effects according to CTC Page 1/4

Cancer Therapy Evaluation Program
Common Toxicity Criteria, Version 2,0
Publish Date: April 30, 1999

Blood

Grade	0	1	2	3	4
**Hemoglobin (g/l)**	WNL	<LLN - 100	≥80 - <100	≥65 - <80	<65
**Leukocytes (mm³)**	WNL	<LLN - 3000	≥2000 - <3000	≥1000 - <2000	<1000
**Granulocytes (mm³)**	WNL	<LLN - 1500	≥1000 - <1500	≥500 - <1000	<500
**Platelets (mm³)**	WNL	<LLN - 75,000	≥50,000 - <75,000	≥10,000 - <50,000	<10,000

Auditory/Hearing

Grade	0	1	2	3	4
**Inner ear / hearing**	normal	hearing loss on audiometry only	tinnitus or hearing loss, not requiring hearing aid or treatment	tinnitus or hearing loss, correctable with hearing aid or treatment	severe unilateral or bilateral hearing loss (deafness), not correctable
**Bilateral hearing loss** *(Brock et al, 1991)*	< 40 dB at all frequencies	> 40 dB at 8000 Hz only	> 40 dB at 4000 Hz only	> 40 dB at 2000 Hz only	> 40 dB at 1000 Hz only

Neurology

Grade	0	1	2	3	4
**Neuropathy-cranial**	absent	-	present, not interfering with activities of daily living	present, interfering with activities of daily living	life-threatening, disabling
**Neuropathy-motor**	normal	subjective weakness but no objective findings	mild objective weakness interfering with function, but not interfering with activities of daily living	objective weakness interfering with activities of daily living	paralysis
**Neuropathy-sensory**	normal	loss of deep tendon reflexes or paresthesia (including tingling) but not interfering with function	objective sensory loss or paresthesia (including tingling), interfering with function, but not interfering with activities of daily living	sensory loss or paresthesia interfering with activities of daily living	permanent sensory loss that interferes with function
**Seizure(s)**	none	-	seizure(s) self-limited and consciousness is preserved	seizure(s) in which consciousness is altered	seizure(s) of any type which are prolonged, repetitive of difficult to control (e.g., status epilepticus, intractable epilepsy)

**Abdominal pain or cramping**	none	mild pain not interfering with function	moderate pain: pain or analgesics interfering with function, but not interfering with activities of daily living	severe pain: pain or analgesics severely interfering with activities of daily living	disabling

Infection

**Grade**	**0**	**1**	**2**	**3**	**4**
**Infection**	none	mild, no active treatment	moderate, localized infection, requiring antibiotic treatment	severe, systemic infection, requiring IV antibiotic or antifungal treatment, or hospitalization	life-threatening sepsis (e.g., septic shock)
**Fever (in the absence of neutropenia, where neutropenia is defined as AGC < 1,0 x 10^9/L)**	none	38,0 - 39,0°C	39,1 - 40,0°C	>40,0°C for < 24 hrs	>40,0°C for >24 hrs

Renal

**Grade**	**0**	**1**	**2**	**3**	**4**
**Hematuria**	none	microscopic only	intermittent gross bleeding, no clots	persistent gross bleeding or clots; may require catheterization or instrumentation, or transfusion	open surgery or necrosis or deep bladder ulceration
**Creatinine (x ULN)**	WNL	> ULN - 1,5	> 1,5 - 3,0	> 3,0 - 6,0	> 6,0
**Proteinuria (g/24 hrs)**	normal or <0,15	1+ or 0,15 - 1,0	2+ to 3+ or 1,0 - 3,5	4+ or >3,5	nephrotic syndrome
**Creatinine-clearance (ml/min + 1,73 m²)**	≥ 90	60 - 89	40 - 59	20 - 39	≤ 19

Nausea/Vomiting

**Grade**	**0**	**1**	**2**	**3**	**4**
**Nausea**	none	able to eat	oral intake significantly decreased	no significant intake, requiring IV fluids	-
**Vomiting (number of episodes/24 h)**	none	1 over pretreatment	1-5 over pretreatment	≥ 6 over pretreatment; or need for IV fluids	requiring parenteral nutrition; or physiologic consequences requiring intensive care, hemodynamic collapse

Constitutional Symptoms

**Grade**	**0**	**1**	**2**	**3**	**4**
**Weight loss**	< 5%	5 - <10%	10 - <20%	≥ 20%	-
**Alopecia**	normal	mild hair loss	pronounced hair loss	-	-
**Fatigue**	none	increased fatigue over baseline, but	moderate (e.g., decrease in per-	severe (e. g., decrease in per-	bedridden or disabling

		not altering normal activities	formance status by 1 ECOG level or 20% Karnofsky or Lansky) or causing difficulty performing some activities	formance status by ≥2 ECOG levels) or loss of ability to perform some activities	
**Anorexia**	none	loss of appetite	oral intake significantly decreased	requiring IV fluids	requiring feeding tube or parenteral nutrition

Allergy

**Allergic reaction / hypersensitivity**	none	transient rash, drug fever <38°C (y< 100,4°F)	urticaria, drug fever ≥38°C (≥100,4°F) and/or asymptomatic bronchospasm	symptomatic brochospasm, requiring parenteral medication(s) with or without urticaria, allergy-related edema/angioedema	anaphylaxis

Gastrointestinal

**Grade**	**0**	**1**	**2**	**3**	**4**
**Mucositis**	none	erythema of the mucosa	patchy pseudomembranous reaction (patches generally ≤ 1,5 cm in diameter and non-contiguous)	confluent pseudomembranous reaction (contiguous patches generally > 1,5 cm in diameter)	necrosis or deep ulceration; may include bleeding not induced by minor trauma or abrasion
**Stomatitus/pharyngitis**	none	painless ulcers, erythema, or mild soreness in the absence of lesions	painful erythema, edema or ulcers, but can eat or swallow	painful erythema, edema or ulcers requiring IV hydration	severe ulceration or requires parenteal of enteral nutritional support or prophylactic intubation
**Diarrhea**	none	increase of <4 stools/day over pre-treatment	increase of 4-6 stools/day or nocturnal stools	increase of ≥7 stools/day or incontinence; or need for parenteral support for dehydration	physiologic consequences requiring intensive care, or hemodynamic collapse
**Constipation**	none	requiring stool softener or dietary modification	requiring laxatives	obstipation requiring manual evacuation or enema	obstruction or toxic megacolon

Dermatology/Skin

**Grade**	**0**	**1**	**2**	**3**	**4**
**Radiation dermatitis**	none	faint erythema or dry desquamation	moderate to brisk erythema or a patchy moist desquamation, mostly confined to skin folds and creases; moderate edema	confluent moist desquamation ≥ 1,5 cm diameter and not confined to skin folds; pitting edema	skin necrosis or ulceration of full thickness dermis; may include bleeding not induced by minor trauma or abrasion

4/4

Hepatic

**Grade**	**0**	**1**	**2**	**3**	**4**
**Bilirubin** (x ULN)	WNL	> ULN - 1,5	> 1,5 - 3,0	> 3,0 - 10,0	> 10,0
**SGOT/SGPT** (x ULN)	WNL	> ULN - 2,5	> 2,5 - 5,0	> 5,0 - 20,0	> 20,0

Pulmonary

**Grade**	**0**	**1**	**2**	**3**	**4**
**Dyspnea**	normal	-	dyspnea on exer-tion	dyspnea at nor-mal level of activity	dyspnea at rest or requiring ventilator support

Cardiovascular

**Grade**	**0**	**1**	**2**	**3**	**4**
**Cardiac left ventri-cular function**	normal	asymptomatic decline of resting ejection fraction of ≥ 10% but < 20% of baseline value; shortening fraction ≥24'% but < 30%	asymptomatic but resting ejec-tion fraction below LLN for laboratory or decline of resting ejection fraction ≥ 20% of base-line valuie; <24% shortening fraction	CHF responsive to treatment	severe or refrac-tory CHF or requi-ring intubation
**LV-EF Echocardiography**	> 30%	26% - 30%	21% - 25%	16% - 20%	< 16%

Abbreviations:

LLN: lower limit of normal values
LV-EF: left ventricular ejection fraction
ULN: upper limit of normal values
WNL: within normal limits

21.12. UNEXPECTEDSERIOUSADVERSEEVENTS

SIOP - LGG 2003
PAGE 1/1

National Coordinating Center:

Surname, Prename | Pat.-Nr. | Hospital | Pat.-Identity-Nr.

I_______________________________I I_I_I_I_I I_I_I_I_I I__I I__I__I__I__I__I__I I_I__I__I__I__I

Date of birth

Unexpected, serious adverse events during treatment must be reported immediately to the national coordinating center, i. e. within the next working day, and followed up by the treating institution.

FAX-Number:

Reason for SAE-report:

- Therapy-related death (-> please fill out „Patient Status Report" form 21.13.2. ❒ yes ❒ no
- Event, leading to a permanent, relevant handicap. ❒ yes ❒ no
- Life-threatening event ❒ yes ❒ no
- Unexpected, serious adverse, which cannotbe documented on the toxicity form ❒ yes ❒ no
- Grade 4 toxicity (renal, hepatic, cardiac, skin, nervous systems) symptoms ❒ yes ❒ no

Grading of SAE after NCI-CTC: ❒ 1 ❒ 2 ❒ 3 ❒ 4 ⑨ unknown /not assign to

Start or rather proof of SAE on I__I__I . I__I__I . I__I__I__I__I

During / after which element of therapy?

❒ Induction I ❒ Induction II ❒ Consolidation ❒ after end of therapy ❒ radiotherapy ❒ others______________________

Please, describe the event and the adopted measures:
(kind, start, period of time, expression/severity, causality. Coherent symptoms and laboratory test results should be summed up to only one)

____
____
____

Drugs at start of SAE:

	**drug**	**dose/day**	**mode of application**	**day of therapy (from / to)**	**Relation between drug and SAE**					
					**none**	**impro-bable**	**possible**	**probable**	**sure**	**no decision, insufficient data**
**1.**					❒	❒	❒	❒	❒	❒
**2.**					❒	❒	❒	❒	❒	❒
**3.**					❒	❒	❒	❒	❒	❒
**4.**					❒	❒	❒	❒	❒	❒
**5.**					❒	❒	❒	❒	❒	❒
**6.**					❒	❒	❒	❒	❒	❒

- Is there a relation between SAE and the drugs, which have been applicated before SAE? ❒ no ❒ yes,______________________
- Have one or several drugs been discontinued? ❒ no ❒ yes, Nr.___________________
- Did the reaction wear off after stopping these? ❒ no ❒ yes, Nr.___________________
- Have one or several drugs been restarted? ❒ no ❒ yes, Nr.___________________
- Has the reaction reappeard after this drug's restart? ❒no ❒ yes, Nr.___________________
- Have one or more drug dosages been changed? ❒ no ❒ yes, Nr.___________________

What do you assume to cause of the start of SAE?

❒ complication of tumor ❒ other, concommittant disease
❒ complicationen of therapy with SIOP-LGG 2004 protocol ❒ other, concommittant therapy
❒ other known or possible reasons; explaining:____

Course

❒(still) continuing SAE ❒ recovery without sequelae ❒ recovery with sequelae
❒ dead following SAE ❒dead, no connection to SAE
Date of recovery or dead: I__I__I.I__I__I.I__I__I <u>or</u> ❒ not applicable (still continuing)
remarks:____

date stamp/signature name in blockletter

21.13.1. Investigations during therapy and follow-up

SIOP - LGG 2004
Page 1/2

I. Investigations required for central data base from children receiving chemotherapy in the randomised study:

	Diagnosis	Commencement of treatment Time: 0	After start of treatment: 6 months	12 months	18 months	Follow-up (see III)
Tumor status	✔	✔	✔	✔	✔	✔
Imaging	✔	✔	✔	✔	✔	✔
Vision	✔	✔	✔	✔	✔	✔
Symptoms/ Neurology	✔	✔	✔	✔	✔	✔
Indication for therapy		✔				
Toxicity	---	---	✔	✔	✔	---
Neuropsychology	✔	✔	---	---	✔	✔
Health status	✔	✔	---	---	✔	✔

II. Recommended frequency of ophthalmological examination during treatment and follow-up for all children with low grade glioma, especially with hypothalamic-chiasmatic lesions (Lorenz 2002):

At diagnosis				
Surgery	before	after	2 weeks after	each surgical intervention
Chemotherapy	before	3-monthly		During chemotherapy
Radiotherapy	before		3 months	After the end of radiotherapy
Follow-up	1st year	3 monthly		
	2nd year:	3-6 monthly		More frequently, if indicated
	3rd year	6 monthly		More frequently, if indicated
	4th year and later:	6-12 monthly		More frequently, if indicated

Page 2/2

III. Follow-up investigations for children being initially observed and for children after termination of chemo- or radiotherapy:

	**First, second and third year**	**Fourth and fifth year**	**Sixth to tenth year**
Physical examination and neurological examination, including anthropometric measurements;	Every 3 months	Every 6 months	Annually
Ophthalmological examination	Year 1: 3 monthly<br>Year 2: 3-6 monthly<br>Year 3: 6 monthly	Every 6 to 12 months	Annually, yet six-monthly in OPG
Contrast enhanced cerebral and spinal (if indicated) MRI	Every 6 months	Every 6 months	Annually
Audiogram – pure tone where possible age 3 years or over otherwise free field testing or otoacustic emissions	Every 6 months	Not indicated if previously repetitively normal	---
Glomerular filtration rate (GFR)	6 months after CT, then yearly, if not indicated otherwise	Not indicated if previously repetitively normal	---
Endocrinologic investigation and, if indicated, bone age and hypothalamic-pituitary functioning test	Yearly if not indicated otherwise	As indicated by stage of growth and puberty and previous chemo- or radiotherapy	As indicated by stage of growth and puberty and previous chemo- or radiotherapy

21.13.2. Patient status report

SIOP - LGG 2004
Page 1/2

National Coordinating Center:

UPN-No. SIOP-Stud.-No.

Surname	Prename	date of birth	hospital

Date of last information:

▶

Date of last examination(at reporting institution): I__I__I . I__I__I . I__I__I__I__I

Date of last neuro-imaging: I__I__I . I__I__I . I__I__I__I__I
❒ MRI ❒ with contrast enhancement ❒ without contrast enhancement
❒ CT ❒ with contrast enhancement ❒ without contrast enhancement
(Please enclose report)

Tumor size: I__I__I cm x I__I__I cm x I__I__I cm

Patient status at last documentation:

▶

Current patient status

❒ Complete remission, no tumor **(CR)** ❒ Stable disease / residual tumor without change of size**(SD)**
❒ Residual tumor in regression **(PR)** ❒ Progression **(PD)**

Diagnosis of relapse / progression / dissemination::

❒ no ❒ yes, on I__I__I . I__I__I . I__I__I__I__I
❒ Primary tumor ❒ metastases, where:________________________

Histologic diagnosis at last information:

▶

Change of histological diagnosis:

no ❒ yes, :____

Therapy at last documentation:

▶

Commencement of new specific therapy?
❒ **no** ❒ **yes**
❒ **surgery** Date: I__I__I . I__I__I . I__I__I__I__I
❒ biopsy (❒ open ❒ stereotactic)
❒ resection (❒ partial ❒ subtotal ❒ total)
❒ shunt (type:_____________________________________)

❒ **chemotherapy** start of therapy: I__I__I . I__I__I . I__I__I__I__I
❒ Induction I ❒ alternative Consolidation
❒ Induction II
❒ other____

❒ **radiotherapy** start of therapy: I__I__I . I__I__I . I__I__I__I__I
❒ external XRT (total dose ___ gy, dose per fracton ___ Gy)
❒ interstitial radiotherapy 125-Jod, ❒ other:____________________)

Serious complications:
❒ **no** ❒ **yes**, manifestation ❒ during ❒ following
❒ surgery
❒ chemotherapy
❒ Induction ❒ Consolidation
❒ radiotherapy
which (CTC-Grade):____
____
____

Patient status report

2/2

Significant changes of neurologic findings as compared to last documentation:

____

____

____

____

In case of death:

date of death: I__I__I . I__I__I . I__I__I__I__I

Reason of death:

❒ caused by primary tumor ❒ other cause of death
❒ caused by relapse/metastases ❒ relation to tumor cannot be judged
❒ therapy related (please explain, send SAE form)

____

autopsy: ❒ no ❒ yes

In case the last examination dates back more than 2 years: ❒ **no** ❒ **yes**

If yes, do you know, where treatment / follow-up is done?

❒ No ❒ Yes: ....
....
....

If yes, do you know the treating physician?

❒ No ❒ Yes: ....
....
....

Remarks:

____

____

____

____

____

**Stamp**	**Date**	**Signature**<br>(Please sign readable in case of further inquiries)

21.13.3. Event Report Form

SIOP - LGG 2004
Page 1/1

National Coordinating Center:

Surname, Prename I__________________________________I Pat.-Nr. I_I_I_I_I Hospital I_I_I_I_I Pat.-Identity-Nr. I__I I__I__I__I__I__I__I I_I__I__I__I__I

Date of birth

Type of Event (Please complete patient status form as well):

MRI/CT-scan (date) I__I__I . I__I__I . I__I__I__I__I (Send report to national coordinator)

❒ **Progression of residual tumor**
- ❒ clinical progression
- ❒ radiological progression (>25%)
- ❒ dissemination:___________________

❒ **Relapse after complete remission**

❒ **Second malignant neoplasm**

❒ **Death** ⇒ send Patient status form 21.13.2.

Previous Therapy:

❒ Observation („wait and see“) ❒ Chemotherapy ❒ Radiotherapy

Treatment following tumor progression / relapse / second malignant neoplasm:

❒ **No therapy** (continue „wait and see-strategy“)

❒ **Surgical intervention**: Date of surgery I__I__I . I__I__I . I__I__I__I__I

Extent: ❒ complete ❒ subtotal ❒ partial ❒ biopsy (❒ open ❒ stereotactic)

Implantation of shunt (date) I_I_I . I_I_I .I_I_I_I_I Type of shunt____________________

❒ **Indication for non-surgical therapy**

⇒ send: Basic therapy information“ (Addendum 21.5.2.) to national study-coordinator!

Date of start of therapy I__I__I . I__I__I . I__I__I__I__I

❒ **Chemotherapy** (Treatment center _______________________________________)

❒ SIOP LGG 2004 CT ⇒ Randomisation of induction therapy (Addendum 21.6.1.)

❒ other

____

❒ **Radiotherapy** (Radiotherapist:____)

❒ conventional radiotherapy

❒ 125-Jodine-interstitial radiotherapy

❒ other ____

❒ **Other therapy**____

Remarks: ____

____

Date: _____________________ Stamp/Signature: ________________________________

21.13.4. Assessment at registration **SIOP - LGG 2004**
Neurology, endocrinology and education **Page 1/3**

National coordinating center:

Surname, Prename Pat.-Nr. Hospital Pat.-Identity-Nr.
I___________________________________I I_I_I_I_I I_I_I_I_I I__I I__I__I__I__I__I__I__I I__I__I__I__I__I__I
Date of birth

Hospital... Country...

1. Is there any history, prior to the child´s tumor related illness, of the child having delay in early developmental milestones or special educational needs or any pre-existing disability?

❒ no ❒ yes If yes, please specify:…………………………………………………

2. Status immediately pre-resection (assessed retrospectively) or before start of any treatment

Date I__I__I I__I__I I__I__I__I__I
D D M M Y Y Y Y

	a) pre-resection	b) before start of treatment	c) change between pre- and post-resection
		circle the most appropriate response !	
**consciousness**	full/impaired/unknown	full/impaired/ unknown	same/better/worse/ unknown
**vision for age** (complete 21.13.6.)	normal/impaired/ unknown	normal/impaired/ unknown	same/better/worse/ unknown
**impairment of nerve III, IV, VI**	yes/no/ unknown	yes/no/ unknown	same/better/worse/ unknown
**facial weakness**	yes/no/ unknown	yes/no/ unknown	same/better/worse/ unknown
**impairment of nerve IX,X,XI**	yes/no/ unknown	yes/no/ unknown	same/better/worse/ unknown
**ataxia of arm(s)**	yes/no/ unknown	yes/no/ unknown	same/better/worse/ unknown
**truncal ataxia**	yes/no/ unknown	yes/no/ unknown	same/better/worse/ unknown
**limb weakness**	yes/no/ unknown	yes/no/ unknown	same/better/worse/ unknown

If you answered 'yes' to limb weakness, please specify pattern (e.g. left hemiplegia):

pre-resection pattern of weakness:______________________

post-resection pattern of weakness:_____________________

Stamp **Date** **Signature**

21.13.4 Assessment at registration Neurology, endocrinology and education

SIOP - LGG 2004
Page 2/3

Surname, Prename I_________________________________I Pat.-Nr. I_I_I_I_I Hospital I_I_I_I_I Pat.-Identity-Nr. I__I I__I__I__I__I__I__I I__I__I__I__I__I

Date of birth

Hospital.. Country...……………

3. If surgery, post-operative complications within 30 days from surgery
tick all that apply!

readmission to intensive care	❒ yes	❒ no	❒unknown
cerebellar mutism syndrome	❒ yes	❒ no	❒ unknown
seizures	❒ yes	❒ no	❒ unknown
intracranial haemorrhage	❒ yes	❒ no	❒ unknown
hydrocephalus requiring operation	❒ yes	❒ no	❒ unknown
CNS infection	❒ yes	❒ no	❒ unknown
other infection	❒ yes	❒ no	❒ unknown
subcutaneous CSF collection	❒ yes	❒ no	❒ unknown

Days between surgery and full recovery of consciousness *(GCS =15)* days

Days in intensive care unit days

4. SDQ questionnaire done ? ❒ yes ❒ no

5. QoL questionnaire done? PEDQOL ❒ yes ❒ no

CHQ-PF 28 ❒ yes ❒ no

6. Educational information for children in school

a) Did he/she receive any extra help
- in his/her most recent school year? ❒ **Yes** ❒ **No**

- in any previous school year? ❒ **Yes** ❒ **No**

b) Is/was he/she at a mainstream school? ❒ **Yes** ❒ **No**

if 'no', what category of special school is/was it?…………………………........................

c) Is the patient still in full time or part time education?
if 'yes', is he/she at: **School** ❒ **Yes** ❒ **No**
Other ❒ **Yes** ❒ **No**
if 'other', please specify………………………………………..............................

7. Audiology (see 21.8.4.) ❒ done ❒ not done *audiology is graded on best ear:*
Audiogram ❒ grade 0 ❒ grade 1 ❒ grade 2 ❒ grade 3 ❒ grade 4

Stamp **Date** **Signature**

21.13.4 Assessment at registration Neurology, endocrinology and education

SIOP - LGG 2004
Page 3/3

Surname, Prename Pat.-Nr. Hospital Pat.-Identity-Nr.
I___________________________________I I_I_I_I_I I_I_I_I_I I__I I__I__I__I__I__I__I I__I__I__I__I__I
Date of birth

Hospital.. Country...

8. Growth and puberty

Height . cm Date: └┴┘ └┴┘ └┴┴┴┘
DD MM YYYY

Sitting height . cm Weight . kg

Maternal height . cm ❒ measured ❒ reported ❒ unknown

Paternal height . cm ❒ measured ❒ reported ❒ not known

Female

Breast stage (Tanner) *
1 **2 3 4 5** *circle*
→pubertal

Menarche? ❒ no ❒ yes

Male

Genital stage (Tanner) *
1 **2 3 4 5** *circle*
→pubertal

Testicular volumes (ml) *circle*
Right 0/1 2 3 **4 6 8 10 12 15 20 25 30**
Left 0/1 2 3 **4 6 8 10 12 15 20 25 30**
→ pubertal

Last menstrual period └┴┘ └┴┘ └┴┴┴┘ (*for interpretation of hormone levels*)
DD MM YYYY

** Tanner staging described on Form 21.13.5*

9. Current hormone levels

fT4 . pmol/L (normal reference for lab . - . pmol/L)

TSH . mU/L

if patient > 8 years old :

FSH . iu/L : **LH** . iu/L Date: └┴┘ └┴┘ └┴┴┴┘
DD MM YYYY

Estradiol . pmol/L **Testosterone** . nmol/L

On any hormone replacement therapy or hormonal contraceptive pill? ❒ yes ❒ no
-if yes , please specify name(s) ....

Stamp **Date** **Signature**

21.13.5. Endocrinology – Follow-up Growth, puberty and hormone treatment

SIOP - LGG 2004
Page 1/3

Send to National coordinating center:

copy and use each year/as indicated

❒ at end of treatment ❒ 2y; ❒ 3y; ❒ 4y; ❒ 5y ❒ __ y after treatment ❒ at age 20 years

Surname, Prename I__________________________________I Pat.-Nr. I_I_I_I_I Hospital I_I_I_I_I Pat.-Identity-Nr. I__I I__I__I__I__I__I__I I__I__I__I__I__I

Date of birth

Hospital.. Country...……………

1. Auxiology

Height . cm Date: └┴┘ └┴┘ └┴┴┴┘

Sitting height . cm

Weight . kg Date: └┴┘ └┴┘ └┴┴┴┘

2. Puberty

❒ Female

Breast stage (Tanner)
1 **2 3 4 5** *circle*
→ **pubertal**

Menarche?
❒ no
❒ yes, during past year

Regular menstrual cycle?
❒ no ❒ yes ❒ unknown

❒ Male

Genital stage (Tanner)
1 **2 3 4 5** *circle*
→ **pubertal**

Testicular volumes (ml) *circle*
Right 0/1 2 3 **4 6 8 10 12 15 20 25 30**
Left 0/1 2 3 **4 6 8 10 12 15 20 25 30**
→ **pubertal**

Girls Breast development **Stage 1:** Elevation of papilla only. **Stage 2:** Breast bud stage; elevation of breast papilla as small mound. Enlargement of areola diameter. **Stage 3:** Further enlargement and elevation of breast and areola, with no separation of their contours. **Stage 4:** Projection of areola and papilla to form secondary mound above the level of the breast. **Stage 5:** Mature stage; projection of papilla only, due to recession of the areola to the general contour of the breast.

Boys Genital development **Stage 1**: Testis, scrotum and penis are of about same size as early childhood. **Stage 2**: Enlargement of scrotum, and testes (to 4 mls) with skin of scrotum reddening and changing in texture. No change in penis at this stage. **Stage 3**: Enlargement of penis, mainly in length, with further growth of testis (around 10 mls) and scrotum**Stage 4**: Increased penile size (breadth) and development of glans. Testes and scrotum larger;
darkened scrotal skin. **Stage 5**: Genitalia adult in size and shape *(average testes 20ml, 12-30 ml).* Testicular size should be recorded as testicular volume assessed by palpation and compared to Prader orchidometer.

21.13.5. Endocrinology – Follow-up Growth, puberty and hormone treatment

SIOP - LGG 2004
Page 2/3

copy and use each year/as indicated

❐ at end of treatment ❐ 2y; ❐ 3y; ❐ 4y; ❐ 5y ❐ __ y after treatment ❐ at age 20 years

Surname, Prename I___________________________________I Pat.-Nr. I_I_I_I_I Hospital I_I_I_I_I Pat.-Identity-Nr. I__I I__I__I__I__I__I__I I__I__I__I__I__I

Date of birth

Hospital.. Country...……………

3. Hormone supplementation- since last recording? ❐ **no** ❐ **yes, see below**

a) thyroxine ? ❐ yes, ❐ continuing start MM YYYY stop MM YYYY

pre-treatment **fT4** . pmol/L (normal range for lab . - .)

TSH . mU/L

b) growth hormone? ❐ yes, ❐ continuing start MM YYYY stop MM YYYY

pre-treatment:

Height . cm; **Sitting height** . cm; **Weight** . kg

c) hydrocortisone? ❐ yes, ❐ continuing start MM YYYY stop MM YYYY

d) GnRH analogues? (to suppress puberty) ❐ yes, ❐ continuing start MM YYYY stop MM YYYY

e) sex steroid? (specify)…………………….......... ❐ yes, ❐ continuing start MM YYYY stop MM YYYY

f) other(s) ? …………… (specify).................................. ❐ yes, ❐ continuing start MM YYYY stop MM YYYY

21.13.5. Endocrinology – Follow-up
Growth, puberty and hormone treatment
SIOP - LGG 2004
Page 3/3

POST TREATMENT hormone levels

copy and use each year/as indicated

❒ at end of treatment ❒ 2y; ❒ 3y; ❒ 4y; ❒ 5y ❒ __ y after treatment ❒ at age 20 years

Surname, Prename I__________________I Pat.-Nr. I_I_I_I_I Hospital I_I_I_I_I Pat.-Identity-Nr. I__I__I__I__I__I__I__I__I I__I__I__I__I__I

Date of birth

Hospital.. Country...

1. Thyroid hormone levels

fT4 . pmol/L (normal range for lab . - . pmol/L)

TSH . mU/L Date: └┴┘ └┴┘ └┴┴┴┘ D D M M Y Y Y Y

2. Gonadotrophins and sex steroids if patient > 8 years old

LH . iu/L Date: └┴┘ └┴┘ └┴┴┴┘ D D M M Y Y Y Y

FSH . iu/L

Estradiol . pmol/L

Testosterone . nmol/L

If female and post-menarcheal, record first day of last menstrual period

(for interpretation of hormone levels) Date: └┴┘ └┴┘ └┴┴┴┘ D D M M Y Y Y Y

**Stamp**	**Date**	**Signature**

21.13.6. Ophthalmology data form

SIOP - LGG 2004
Page 1/1

National coordinating center:

Surname, Prename | Pat.-Nr. | Hospital | Pat.-Identity-Nr.

I__________________________________I I_I_I_I_I I_I_I_I_I I__I I__I__I__I__I__I__I I__I__I__I__I__I

Date of birth

Hospital... Country...

Date of examination I___I___I . I___I___I . I___I___I___I___I

(for details see back side and section 8.6.)

**Current status**	**Follow up assessment***
☐ n.d. **Visual Acuity:** RE _____ LE _____ Method: ________________<br>☐ n.d. **Refraction:** RE _____ LE _____ in mydriasis: ❑ yes ❑ no	same / better / worse
☐ n.d. **Visual Fields** RE _____ LE _____ grade ____<br>(please attach copy) Method: _______________________	same / better / worse
☐ n.d. **Colour Vision** RE _____ LE _____ grade ____<br>Method:______________________<br>Which colour predominantly lost? I___I<br>(1 = blue; 2 = red; 3 = yellow; 4 = green)	same / better / worse
☐ n.d. **Contrast Sensitivity**: RE _____ LE _____ grade ____<br>Method: ___________________	same / better / worse
☐ n.d. **Ocular Motility** RE_______ LE________	same / better / worse
☐ n.d. **Squint** RE_______ LE ________ alternating _________<br>(1 = none, 2 = convergent, 3 = divergent, 4 = vertical)<br>**Angle of Squint:** distance ________ near ________ (in prism dpt)	same / better / worse
☐ n.d. **Nystagm:** ❑ present ❑ not present<br>direction: RE I__I LE I__I (1 = horizontal, 2 = vertical, 3 = rotatory)<br>type: RE I__I LE I__I (1 = jerk., 2 = pendular, 3 = gaze evoked)	same / better / worse
☐ n.d **Pupillary reaction:** RE ______ LE ______<br>**RAPD:** ❑ present ❑ not present	same / better / worse
☐ n.d. **Fundoscopy:** RE _______ LE _______<br>**Optic atrophy:** I__I (1=optic atrophy generalised, 2=*"Bow Tie"*, 3=no optic atrophy)<br>*If possible, please document state of the papilla and other pathologic findings by fotodocumentation*	same / better / worse
☐ n.d **VEP:** (please send copy, if available)<br>Latency: RE__________ LE __________<br>Amplitude: RE__________ LE __________	same / better / worse

General judgement of examination ❑ same ❑ better ❑ worse

Date ____________ Stamp ____________ Signature ____________

(RE = right eye, LE = left eye, n.d. = not done, RAPD = relative afferent pupillary defect)

* modified January 2006

21.13.7. Post Treatment Late effects and education

SIOP - LGG 2004
Page 1/3

National coordinating center:

copy and use each year/as indicated

❒ at end of treatment ❒ 2y; ❒ 3y; ❒ 4y; ❒ 5y ❒ __ y after treatment ❒ at age 20 years

Surname, Prename I__________________________________I Pat.-Nr. I_I_I_I_I Hospital I_I_I_I_I Pat.-Identity-Nr. I__I I__I__I__I__I__I__I I__I__I__I__I__I
Date of birth

Hospital.. Country..……………

Date of this follow-up assessment └┴┘ └┴┘ └┴┴┴┘
D D M M Y Y Y Y

1. Does the patient have any restriction or lack of ability to perform an activity due to any motor problem, cranial nerve palsy or poor vision? ❒ Yes ❒ No

If 'yes', please indicate which of the following are present:

vision impaired	❒ Yes	❒ No	❒ unknown
extraocular palsy	❒ Yes	❒ No	❒ unknown
facial weakness	❒ Yes	❒ No	❒ unknown
impairment of nerve IX, X, XI	❒ Yes	❒ No	❒ unknown
ataxia of arm(s)	❒ Yes	❒ No	❒ unknown
truncal ataxia	❒ Yes	❒ No	❒ unknown
limb stiffness/weakness	❒ Yes	❒ No	❒ unknown
abnormal unwanted movements	❒ Yes	❒ No	❒ unknown
tremor or trembling	❒ Yes	❒ No	❒ unknown

If you answered 'yes' to limb stiffness/weakness, please specify pattern of weakness (e.g. left hemiplegia):……………………………………………………………..

2. **Is the patient on anticonvulsant therapy?** ❒ Yes ❒ No ❒ **unknown**

3. **FMH (Germany)**	❒ done	❒ not done
4. **SDQ**	❒ done	❒ not done
5. **QLQ-30 (only at age 20 years)**	❒ done	❒ not done

6. **QoL questionnaire for parent and child**

PEDQOL	❒ done	❒ not done
CHQ-PF28	❒ done	❒ not done

_______________ _______________________ _________________________________
Date Stamp Signature

21.13.7. Post Treatment
Late effects and education

SIOP - LGG 2004
Page 2/3

National coordinating center:

copy and use each year/as indicated

❒ at end of treatment ❒ 2y; ❒ 3y; ❒ 4y; ❒ 5y ❒ __ y after treatment ❒ at age 20 years

Surname, Prename I___________________________________I Pat.-Nr. I_I_I_I_I Hospital I_I_I_I_I Pat.-Identity-Nr. I__I I__I__I__I__I__I__I I__I__I__I__I__I

Date of birth

Hospital.. Country...

7. Audiology (according CTC criteria)

Audiometry ❒ grade 0 ❒ grade 1 ❒ grade 2 ❒ grade 3 ❒ grade 4

8. Vision ❒ normal ❒ slightly impaired (no influence on daily live) ❒ impaired (CTC grade 3) ❒ blind

If impaired, please complete ophthalmology form 21.13.6.

9. Skin pigmentation change ❒ none ❒ transitory, slight ❒ permanent, marked

10. Mucosa
(oral /pharyngeal)

- ❒ normal
- ❒ patchy atrophy or teleangiectasia
- ❒ diffuse atrophy or teleangiectasia, superficial ulcer
- ❒ deep ulcer no bone or cartilage exposure
- ❒ deep ulcer with bone or cartilage exposure

11. Dysphagia

- ❒ normal
- ❒ difficulties eating solid food
- ❒ difficulties eating soft food
- ❒ can take liquids only
- ❒ totally unable to swallow

Date ____________ Stamp ____________ Signature ____________

21.13.7. Post Treatment Late effects and education

SIOP - LGG 2004
Page 3/3

National coordinating center:

copy and use each year/as indicated

❐ at end of treatment ❐ 2y; ❐ 3y; ❐ 4y; ❐ 5y ❐ __ y after treatment ❐ at age 20 years

Surname, Prename I__________________________________I Pat.-Nr. I_I_I_I_I Hospital I_I_I_I_I Pat.-Identity-Nr. I__I **I__I__I__I__I__I__I** I__I__I__I__I__I

Date of birth

Hospital.. Country...……………

12) Educational and social information

a) Did he/she receive any extra help

- in his/her most recent school year? ❐ Yes ❐ No

- in any previous school year? ❐ Yes ❐ No

b) Is/was he/she at a mainstream school? ❐ Yes ❐ No

if 'no', what category of special schoolis/was it?………………………….

c) Is the patient still in full time or part time education? ❐ Yes ❐ No

if 'yes', is he/she at: **School** ❐ Yes ❐ No

Other ❐ Yes ❐ No

if 'other', please specify…………………………………..

d) If the patient is no longer attending school,

i) how old was he/she when he/she left? □□ years

ii) where does the patient live?

in the family/parental home? ❐ Yes ❐ No
in sheltered accommodation? ❐ Yes ❐ No
independently (maybe with friends or partner)? ❐ Yes ❐ No
other? ❐ Yes ❐ No
If "other", please specify...

iii) is he/she

in further/higher education ❐ Yes ❐ No
If "yes", please specify...
in full or part time employment? ❐ Yes ❐ No
If "yes", is it a normal job without special adaptation? ❐ Yes ❐ No
If "no", please specify..

Date Stamp Signature

21.14. Participating Centers SIOP-LGG 2004

Centers, having participated in SIOP/GPOH LGG 1, are expeceted to continue their participation throughout SIOP-LGG 2004. However, additional national groups will be joining the International Consortium on Childhood Low Grade Glioma by formal declaration. So at this stage it is not feasable to give a list of participating centers.

22. Addendum 1 SIOP-LGG 2004
für die Behandlungszentren der GPOH Deutschland und GPOH Österreich

1. **SIOP-LGG 2004 Studienkommission der Gesellschaft für pädiatrische Onkologie und Hämatologie (GPOH Deutschland und GPOH Österreich)**

2. **SIOP-LGG 2004 Therapieablauf: Übersicht (Flow chart)**

3. **SIOP-LGG 2004 Zusammenfassung (Abschnitt 1. des Studienprotokolles)**

4. **Unterschriftenseite**

1. SIOP-LGG 2004 Studienkommission der Gesellschaft für Pädiatrische Onkologie und Hämatologie

Arbeitsgruppe für Hirntumoren im Kindesalter

I. Studienleitung

**Dr. med Astrid K. Gnekow**<br>I. Klinik für Kinder und Jugendliche<br>Klinikum Augsburg<br>Stenglinstrasse 2<br>D-86156 Augsburg<br><br>gnekow.hit-lgg@klinikum-augsburg.de<br>Tel. (0049) – 0821 – 400 3615<br>FAX (0049) – 0821 – 400 3616	**Prof. Dr. med. Rolf-D. Kortmann**<br>Klinik für Strahlentherapie<br>Universitätsklinik Leipzig<br>Johannisallee 34<br>04103 Leipzig<br><br>rolf-dieter.kortmann@medizin.uni-leipzig.de<br>Tel. 0049 (0)341 9718542<br>Fax. 0049-(0)3419718549
**Neurochirurgisches Referenzzentrum**<br><br>**Dr. med. Jürgen.Krauss**<br>Klinik für Neurochirurgie<br>Universitätskliniken<br>Josef-Schneider-Strasse 11<br>D-97080 Würzburg<br><br>krauss.j@nch.uni-wuerzburg.de<br>Tel. (0049) – 0931 – 201 – 24841<br>FAX (0049) – 0931 – 201 - 24540	**Biometrie**<br><br>**Dr. rer. nat. Andreas Faldum**<br>Institut für Medizinische Biometrie,<br>Epidemiologie und Informatik<br>Universität Mainz<br>D-55131 Mainz<br><br>faldum@imsd.uni-mainz.de<br>Tel. (0049) – 06131 – 17-3938<br>FAX (0049) – 06131 – 17-473938
**Hirntumorreferenzzentrum**<br><br>**Prof. Dr. med. Torsten Pietsch**<br>Institut für Neuropathologie<br>der Universität<br>Siegmund-Freud-Strasse 25<br>D-53105 Bonn<br><br>referenzzentrum@uni-bonn.de<br>Tel. (0049) – 0228 – 287 6606<br>FAX (0049) – 0228 – 287 4331	**Neuroradiologisches Referenzzentrum**<br><br>**Prof. Dr. med. Monika Warmuth-Metz**<br>Abt. für Neuroradiologie<br>Universitätskliniken<br>Josef-Schneider-Strasse 11<br>D-97080 Würzburg<br><br>hit@neuroradiologie.uni-wuerzburg.de<br>Tel. (0049) – 0931 – 201 – 34799<br>FAX (0049) – 0931 – 201 - 34685

II. Mitglieder der Studienkommission (GPOH Deutschland und GPOH Österreich)

**Neuroradiologie**	**Neuropathologie**	**Neurochirurgie**
Bison, Würzburg<br>Prayer, Wien<br>Warmuth-Metz, Würzburg	Hainfellner, Wien<br>Hans, Bielefeld<br>Pietsch, Bonn	Czech, Wien<br>Krauss, Würzburg<br>Sörensen, Oldenburg<br>Van Velthoven, Freiburg
**Pädiatrische Onkologie**		
Berthold, Köln<br>Gnekow, Augsburg<br>Graf, Homburg<br>Grotzer, Zürich<br>(Studienleitung Schweiz)<br>Jorch, Bielefeld	Kordes, Hamburg<br>Kramm, Halle<br>Peters, Regensburg<br>Pfister, Heidelberg<br>Rutkowski, Hamburg<br>Scheurlen, Nürnberg	Slavc, Wien (Studienleitung Österreich)<br>Urban, Graz<br>Vorwerk, Magdeburg<br>Witt, Heidelberg<br>Wolff, Houston

**Strahlentherapie**	**Neuropädiatrie**
Bamberg, Tübingen<br>Diekmann, Wien<br>Kortmann, Leipzig<br>Timmermann, Essen	Brockmann, Göttingen<br>Ebinger, Heidelberg<br>Hernaiz-Driever, Berlin<br>Sträter, Münster

**Spätfolgen/Lebensqualität**	**Neuroendokrinologie**	**Ophthalmologie**
Calaminus, Düsseldorf<br>Lackner, Graz	Müller, Oldenburg	Wabbels, Bonn
**Statistik**		
Faldum, Mainz<br>(Hauptstudie)<br>Schneider, Mainz		

STUDIENSEKRETARIAT UND DOKUMENTATION
Sabine Breitmoser-Greiner, Marina Geh
I. Klinik für Kinder und Jugendliche
Klinikum Augsburg
Stenglinstrasse 2
D-86156 Augsburg

Tel. (0049) – 0821 – 400 3615
FAX (0049) – 0821 – 400 3616
gnekow.hit-lgg@klinikum-augsburg.de

2. SIOP-LGG 2004 Therapieablauf: Übersicht

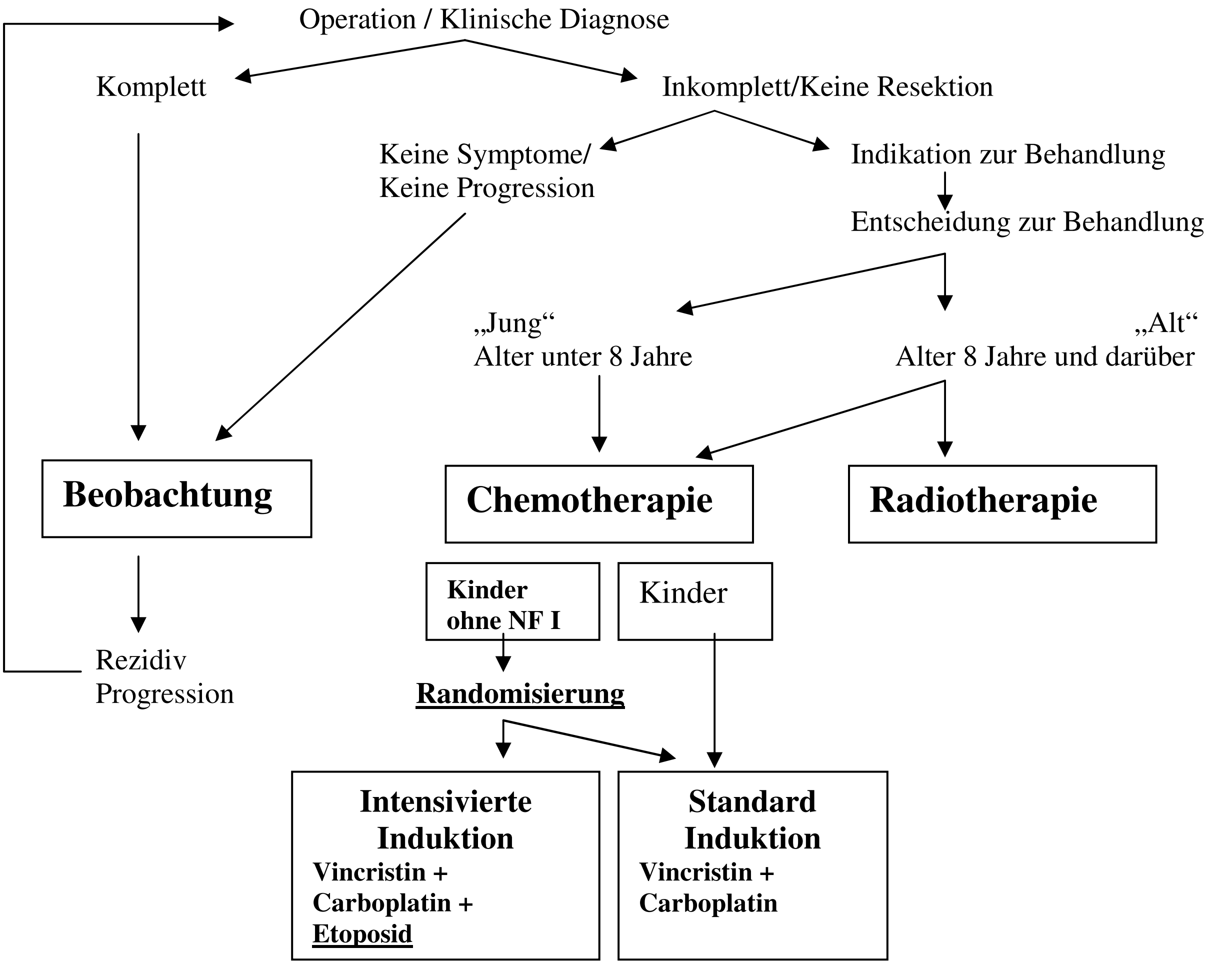

Die Studie SIOP-LGG 2004 sieht für alle Kinder und Jugendliche, bei denen referenz-histologisch (gemäß WHO-Kriterien) oder referenz-radiomorphologisch die Diagnose eines Gliomes niedrigen Malignitätsgrades bestätigt wird, eine einheitliche Therapiestrategie vor. Nach vollständiger Tumorresektion ist ausschließlich eine Beobachtung vorgesehen. Nach unvollständiger Resektion, bei nicht-resezierbarem Rezidiv oder bei Progression eines nicht-resezierbaren Tumors ist in Abhängigkeit von definierten Indikationen eine nicht-chirurgische Therapie vorgesehen.

Ältere Kinder (≥ 8 Jahre) oder Kinder, bei denen eine Brachytherapie möglich ist, erhalten primär eine Radiotherapie, bei besonderen Ausgangsbedingungen können sie auch mit Chemotherapie behandelt werden. Moderne Therapieplanungs- und -durchführungstechniken sollen das Ausmaß radiogener Langzeitschäden am umgebenden gesunden Hirngewebe reduzieren. Jüngere Kinder (< 8 Jahre) erhalten primär eine Chemotherapie. Unabhängig von Alter und Tumorsitz sollen Kinder mit Neurofibromatose I mit Chemotherapie behandelt werden. Die Chemotherapie wird für alle Kinder auf 18 Monate verlängert. Für Kinder ohne NF I (stratifiziert nach Alter und Tumorlokalisation) wird die Induktionstherapie randomisiert als Standardinduktion mit Vincristin und Carboplatin oder als intensivierte Induktion mit Vincristin, Carboplatin und zusätzlichem Etoposid gegeben, um zu prüfen, ob sich das progressionsfreie Überleben zwischen den beiden Induktionsregimen unterscheidet. Zusätzlich soll die Verteilung der Tumorresponse im Anschluß an die Induktion einheitlich zu Woche 24 geprüft werden. In der Konsolidierungsphase wird die Chemotherapie mit 10 Carboplatin/Vincristin-Pulsen im 6-Wochen-Rhythmus fortgesetzt.

Ausgewertet wird für alle Kinder das Gesamtüberleben, das progressionsfreie Überleben und das ereignisfreie Überleben. Der Einfluss klinischer und histologischer Merkmale auf diese Parameter wird überprüft. Das Ausmaß an Folgeschäden durch Grunderkrankung und Therapie wird prospektiv erfasst.

3. SIOP-LGG 2004 Zusammenfassung

Beginn der Hauptphase: 01.04.2004
Voraussichtliches Ende der Hauptphase: 31.03.2010, Verlängerung bis 31.03.2012
Voraussichtliches Studienende: 31.03.2014

EudraCT-Nr: 2005-005377-29, NCI-PDQ Database Code ID: SIOP-LGG 2004 trial EU-20555

1. Organisation

1.1. Studienbezeichnung: SIOP-LGG 2004 – Internationale, kooperative, multizentrische Studie für Kinder und Jugendliche mit Gliomen niedriger Malignität.

1.2. Internationale Studienkommission

Chemotherapie-Protokoll:

Dr. Astrid K. Gnekow
I. Klinik für Kinder und Jugendliche, Klinikum Augsburg
Stenglinstrasse 2
D-86156 Augsburg, Deutschland

Dr. Giorgio Perilongo
Clinica di Oncoematologia Pediatrica e Centro Leucemie Infantili
Via Giustiniani 3
I-35128 Padova, Italien

Dr. David A. Walker
Children's Brain Tumor Research Centre - Queen's Medical Centre
University of Nottingham
Nottingham, NG7 2UH, Großbritannien

Dr Jacques Grill
Département de Cancérologie de l'Enfant et de l'Adolescent
Institut Gustave Roussy
39 rue Camille Desmoulins,
F-94805 Villejuif, Frankreich

Radiotherapie-Protokoll:

Dr. Roger E. Taylor
Department of Radiotherapy and Oncology - Cookridge Hospital
Hospital Lane
Leeds / Cookridge West Yorkshire LS16 6QB, Großbritannien

Prof. Dr. Rolf - D. Kortmann
Abt. Strahlentherapie der Universität Leipzig
Johannisallee 34
D - 04103 Leipzig, Deutschland

Dr. Giovanni Scarzello
Department of Radiotherapy - Padua General Hospital
Via Giustiniani 2
I-35100 Padua, Italien

Biometrie:

Datenanalyse:
Dr. Andreas Faldum
Institut für Medizinische Biometrie,
Epidemiologie und Informatik
University of Mainz
D-55101 Mainz, Germany

Datenmanagement:
Dr. Gian Luca De Salvo
Clinical Trials & Biostatistic Unit
Istituto Oncologico Veneto
Busonera Hospital
Via Gattemelata 64
I-35128 Padova, Italy

Nationale Studienkoordination für die teilnehmenden nationalen Studiengruppen:

Deutschland:	Astrid K. Gnekow, Augsburg
Frankreich:	Jacques Grill, Villejuif
Großbritannien:	Sue Picton, Leeds
Italien:	Giorgio Perilongo, Padua
Norwegen:	Tore Stokland, Tromsø
Österreich:	Irene Slavc, Wien
Spanien:	Ofelia Cruz, Bilbao
Schweden:	Per Eric Sandstrom

Internationales Datenzentrum:

SIOP-LGG 2004 International Data Centre
Clinical Trials & Biostatistic Unit
Istituto Oncologico Veneto
Busonera Hospital
Via Gattemelata 64
I-35128, Italy
Tel: 0039-049-8215704
Fax: 0039-049-8215706
email: siop-lgg2004@istitutoncologicoveneto.it

1.3. Primäre Studienziele (Abschnitt 6 und 7)

1.3.1. Vorgabe eines einheitlichen, standardisierten Konzeptes für die Behandlung von Kindern und Jugendlichen mit einem Gliom niedrigen Malignitätsgrades.

1.3.2. Verbesserung des progressionsfreien Überlebens nach nicht-chirurgischer Therapie für die Studiengruppe der Kinder ohne Neurofibromatose NF I mit niedrigmalignen Gliomen durch die Überprüfung standardisierter Therapieempfehlungen

Gruppe 1: Tumoren der supratentoriellen Mittellinie
Gruppe 2: Tumoren der cerebralen Hemisphären, des Kleinhirnes und kaudalen Hirnstammes und des Rückenmarkes

- Therapiearm: Radiotherapie
  Einsatz moderner Techniken für Planung und Behandlung
- Therapiearm: Chemotherapie
  Verlängerung der Therapie für alle Kinder.
  Randomisierte Überprüfung der Intensivierung der Induktionstherapie.
  Gemeinsame Konsolidierung für alle Kinder mit einer Alternative bei früher Progression oder Allergie.

1.3.3. Überprüfung von standardisierten Therapieempfehlungen für die nicht-chirurgische Therapie in der Studiengruppe mit Kindern mit Neurofibromatose NF I und niedrigmalignen Gliomen aller Lokalisationen.

1.3.4. Verminderung der Häufigkeit und des Schweregrades möglicher Spätfolgen der Therapie:

- Durch die Schonung von Risikoorganen durch optimierte Planung und Durchführung der Strahlentherapie
- Durch die Verschiebung des Beginnes oder die Vermeidung der Strahlentherapie für junge Kinder und für Kinder mit Neurofibromatose durch die Wahl einer Chemotherapie-Strategie.

2. Eingangskriterien (Abschnitt 9.1.)

2.1. Alter: Kinder und Jugendliche bis zum Alter von 18 Jahren

2.2. Histologie: Gliome niedrigen Malignitätsgrades (ICD O-Code)[4]

Pilocytisches Astrocytom I°	9421/1
Subependymales Riesenzell-Astrocytom I°	9384/1
Dysembryoplastischer neuroepithelialer Tumor I°	9413/0
Desmoplastisches infantiles Gangliogliom I°	9412/1
Gangliogliom I° und II°	9505/1
Pleomorphes Xanthoastrocytom II°	9424/3
Oligodendrogliom II°	9450/3
Oligoastrocytom II°	9382/3
Astrocytom II°	9400/3
Fibrilläres Astrocytom II°	9420/3
Protoplasmatisches Astrocytom II°	9410/3
Gemistocytisches Astrocytom II°	9411/3

In der randomisierten Studie werden alle Histologien randomisiert, da bislang keine Daten einen Ausschluss von Subgruppen, z. B. Kinder mit Oligodendrogliom, begründen.
Bei Vorliegen bestimmter neuroradiologischer Kriterien ist es erlaubt, die Diagnose eines niedriggradigen, chiasmatisch-hypothalamischen Tumors ohne Biopsie zu stellen (Abschnitt 8.5.).

2.3. Primäre Tumorlokalisation: Intrakraniell und Rückenmark

2.4. Disseminierung: Kinder mit disseminierten, niedrigmalignen Gliomen sind Studienpatienten.

2.5. Assoziierte Erkrankungen: Das Vorhandensein assoziierter genetischer Erkrankungen ist kein Ausschlussgrund.

2.6. Primäre Tumordiagnose: Der Tumor sollte nicht mit Chemo- oder Radiotherapie vorbehandelt sein.

2.7. Einverständniserklärung: Der Patient und/oder seine Erziehungsberechtigten (Eltern) müssen schriftlich ihr Einverständnis zur Studienteilnahme erklären.

<u>Randomisierung:</u> Alle Patienten ohne Neurofibromatose, die als erste nicht-chirurgische Therapie eine Chemotherapie erhalten, sollen im Rahmen dieser Studie randomisiert werden.

3. Ausschlusskriterien (Abschnitt 9.2.)

3.1. Primäre Tumorlokalisation: Diffuse, intrinsische Ponstumoren, auch wenn histologisch ein Astrocytom WHO °II diagnostiziert wird. Ausnahme: Ponsgliome °II bei NF-I-Patienten können an die Studie gemeldet werden.

---

[4] ICD-Codes korrigiert (Juli 2004)

3.2. Sonderdiagnosen: Kinder, bei denen seltene intrakraniale Tumoren niedriger Malignität vorliegen, die nicht glialen Ursprungs sind. Ihre Daten sollten registriert werden, um Erkenntnisse darüber zu gewinnen, welche therapeutischen Entscheidungen sich als nutzbringend für diese Patienten erwiesen haben und um spezielle Strategien in der Zukunft zu entwickeln. Plexus chorioideus-Papillome sollten der Studie SIOP-CPT zugeführt werden.

3.3. Vorbehandlung: Kinder, die mit Chemo- oder Radiotherapie behandelt wurden, bevor sie in die LGG-Studie eingebracht wurden, werden gesondert ausgewertet. Die Vorbehandlung mit Steroiden wird nicht als Chemotherapie-Behandlung angesehen.

3.4. Vorerkrankungen, die die Durchführung der Studie unmöglich oder ethisch bedenklich erscheinen lassen.

3.5. Schwangerschaft und Stillzeit.

Falls der Patient parallel zur Studienteilnahme an der SIOP-LGG 2004 Studie an einer anderen klinischen Studie teilnimmt, deren Inhalt jedoch nicht mit den Zielen der vorgegebenen Therapiestrategie interferiert (z.B. Hormonsubstitution, antikonvulsive Therapie), sollte dies dem nationalen Studienkoordinator mitgeteilt werden.

Begleitmedikation für Folgeerkrankungen oder andere unabhängige Erkrankungen (z.B. Hormonsubstitution, antikonvulsive Therapie) sollte gemeldet werden, stellt jedoch keinen Ausschlußgrund dar.

4. Therapieübersicht (Abschnitt 12)

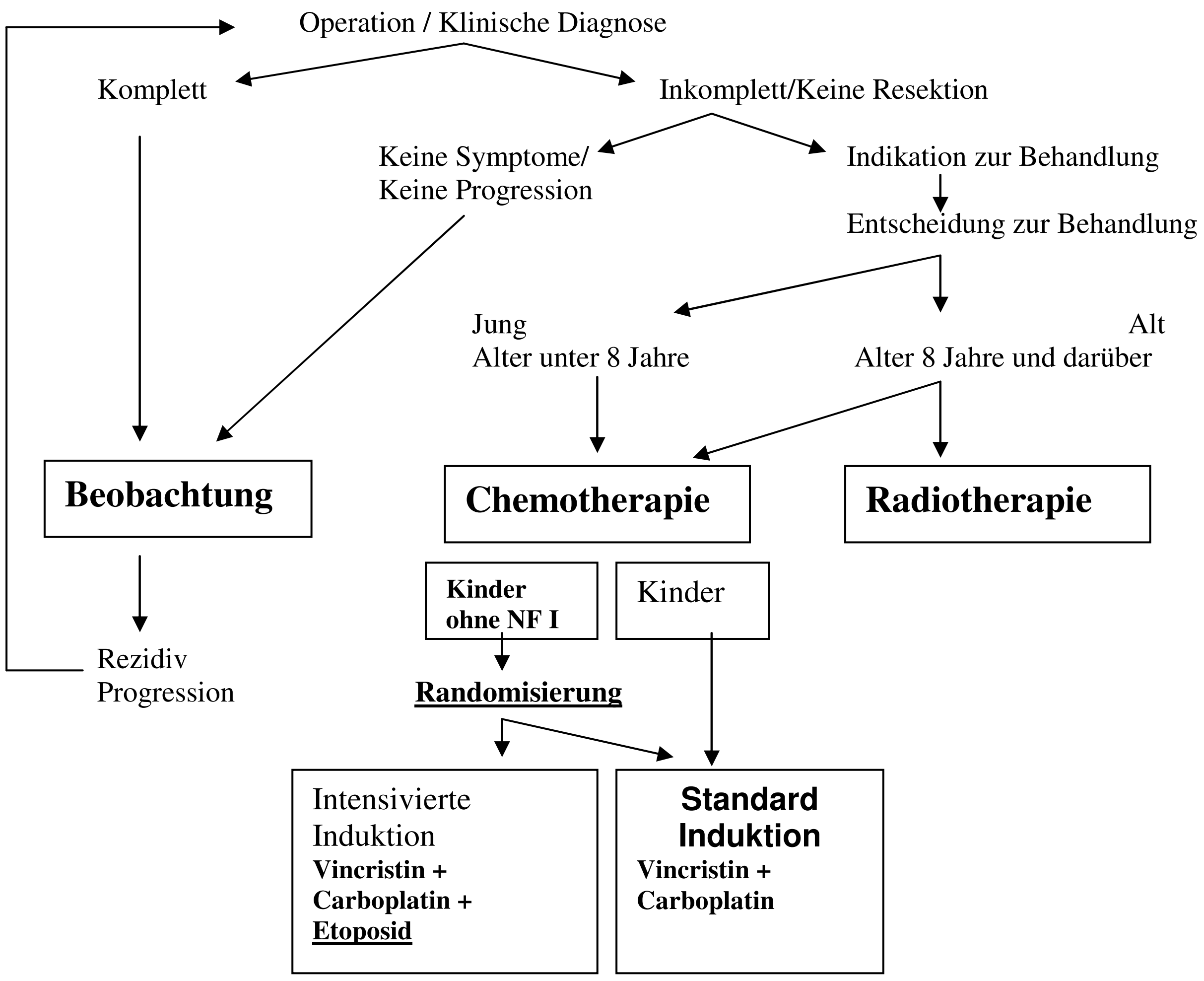

4.1. Grundlegender Behandlungsablauf

Alle Patienten mit einem Gliomen niedrigen Malignitätsgrades, die die Eingangskriterien entsprechend Abschnitt 9 erfüllen, sollen in die laufende Studie aufgenommen werden. Sie folgen bezüglich der nicht-chirurgischen Therapie einer einheitlichen Strategie. Abhängig von der primären Tumorlokalisation und dem Vorhandensein oder Nichtvorhandensein einer Neurofibromatose NF I werden die Patienten in drei Studiengruppen aufgeteilt:
Gruppe 1: Keine NF I, supratentorielle Mittellinie (Abschnitt 12.1.)
Gruppe 2: Keine NF I, cerebrale Hemisphären, Kleinhirn, kaudaler Hirnstamm, Rückenmark, N. opticus (Abschnitt 12.2.)
Gruppe 3: NF I, alle Lokalisationen (Abschnitt 12.3.)

4.2. Therapieuntergruppen

Die Indikation für eine nichtchirurgische Therapie im Anschluss an die Diagnose wird für einen Patienten mit niedrigmalignem Gliom von dem Ausmaß der chirurgischen Resektion, dem Vorhandensein oder dem Fehlen einer schweren neurologischen Symptomatik und dem Vorhandensein oder dem Fehlen einer klinischen und/oder neuroradiologischen Progression während einer Beobachtungsperiode abhängig gemacht. Innerhalb aller Studiengruppen gibt es daher drei Therapieuntergruppen:

4.2.1. Beobachtungsgruppe: Tumor komplett reseziert
- Tumor nicht oder inkomplett reseziert, keine schweren Symptome
- Tumor nicht oder inkomplett reseziert, keine Progression

4.2.2. Behandlungsgruppe bei Diagnosestellung:
- Schwere neurologische Symptome
- Schwere ophthalmologische Symptome

4.2.3. Behandlungsgruppe nach Beobachtungszeit:
- Progrediente neurologische Symptome
- Progrediente ophthalmologische Symptome
- Neuroradiologische Progression einschließlich Disseminierung

4.3. Stratifizierung der nicht-chirurgischen Therapie

In jeder Studiengruppe gibt es spezifische Angaben für eine altersbezogene Stratifizierung der nicht-chirurgischen Therapie:

Primäre Chemotherapie:	„Junge“ Kinder, Alter < 8 Jahre
	Alle Kinder mit NF I
Primäre Radiotherapie:	„Ältere“ Kinder, Alter ≥ 8 Jahre
	Kinder aller Altersgruppen, deren Tumor mit interstitieller Radiotherapie behandelt werden kann (Brachytherapie)

4.4. Indikation zum Beginn der nicht-chirurgischen Therapie (Abschnitt 10)

Klinische und ophthalmologische Symptome werden erfasst und regelmäßig werden neuroradiologische Untersuchungen durchgeführt, um im Anschluss an die Diagnosestellung zu entscheiden, ob es eine Indikation zur nicht-chirurgischen Therapie gibt.

4.4.1. Indikation zum Beginn der nicht-chirurgischen Therapie bei Diagnosestellung im Anschluss an eine subtotale oder partielle Resektion.

Schwere, vorbestehende Einschränkungen der Sehfähigkeit (Abschnitt 8.6.)
- Grenzwertiger Visus in beiden Augen („threat to vision“)

Dokumentierte Visusverschlechterung
Nystagmus in Folge erheblicher Visuseinschränkungen (dies ist insbesondere bei Kleinkindern bis zum Alter von zwei Jahren ein Zeichen eingeschränkter Sehfähigkeit)

Klinische Indikation
Diencephales Syndrom
Symptomatische Metastasen

Hinweis: Das Vorliegen eines postoperativen Resttumors ist keine eigenständige Therapieindikation

4.4.2. Indikation zum Beginn einer nicht-chirurgischen Therapie zum Zeitpunkt der Diagnose ohne vorherige Tumorresektion (im Anschluss an eine Biopsie oder radiologische Diagnose).

Schwere Sehstörungen (Abschnitt 8.6.)
Grenzwertiger Visus in beiden Augen („threat to vision“)
Dokumentierte Visusverschlechterung
Nystagmus in Folge erheblicher Visuseinschränkungen (dies ist insbesondere bei Kleinkindern bis zum Alter von zwei Jahren ein Zeichen eingeschränkter Sehfähigkeit)

Schwere neurologische Symptome
Diencephales Syndrom
Fokale neurologische Ausfälle in Folge des Tumorwachstums
Symptome erhöhten intrakranialen Druckes in Folge Tumorwachstum
(Fokale) Krampfanfälle in Folge des Tumorwachstums
Symptomatische Metastasen

Hinweis: Das Vorliegen des Tumors selbst ist keine eigenständige Therapieindikation.

4.4.3. Indikation zum Beginn einer nicht-chirurgischen Therapie nach einer Beobachtungsperiode, wenn eine chirurgische Intervention nicht möglich ist.

Progrediente neurologische Symptome
Entwicklung neuer neurologischer Symptome
Entwicklung eines diencephalen Syndroms

Progrediente Sehstörungen
Verminderung/Verlust des Visus oder des Gesichtsfeldes
Jede Visusreduktion im zweiten Auge, wenn das andere Auge bereits erblindet ist.

Neuroradiologische Progression
Eindeutige Zunahme der Tumorgröße (Zunahme des Durchmessers des N. opticus)
Einbeziehung zuvor nicht einbezogener Hirnabschnitte
Entwicklung einer Tumordisseminierung (einschließlich symptomatischer oder progredienter Metastasen, symptomatischer leptomeningealer Disseminierung)

4.5. Chemotherapie (Abschnitt 14)

4.5.1. Induktionstherapie

Die Induktionstherapie wird zwischen der Standard- und der intensivierten Induktion für die Gruppen 1 und 2 randomisiert (keine NF I, 1: Tumoren der supratentoriellen Mittellinie, 2: Niedrigmaligne Gliome aller anderen Lokalisationen).

Therapiegruppe 3 (NF I, niedrigmaligne Gliome aller Lokalisationen) erhält die Standardinduktion

Standard-Induktion:

1	2	3	4	5	6	7	8	9	10	13	17	21	24	Woche
**V**	**V**	**V**	**V**	**V**	**V**	**V**	**V**	**V**	**V**	**V**	**V**	**V**		
**C**			**C**			**C**			**C**	**C**	**C**	**C**		
													**MRT**	

Intensivierte Induktion:

1	2	3	4	5	6	7	8	9	10	13	17	21	24	Woche
**V**	**V**	**V**	**V**	**V**	**V**	**V**	**V**	**V**	**V**	**V**	**V**	**V**		
**C**			**C**			**C**			**C**	**C**	**C**	**C**		
**Ex3**			**Ex3**			**Ex3**			**Ex3**					
													**MRT**	

V	Vincristin	1,5 mg/m²	iv-Bolus – T 1
C	Carboplatin	550 mg/m²	1h iv – T 1
E	Etoposid	100 mg/m²	1h iv – T 1 – 3

Die neuroradiologische Untersuchung nach 24 Wochen wird zentral ausgewertet zur Erfassung der Response.

4.5.2. Konsolidierungstherapie:
Alle Patienten erhalten eine einheitliche Konsoliderungstherapie.

25	31	37	43	49	Woche
55	61	67	73	79	
**VVV**	**VVV**	**VVV**	**VVV**	**VVV**	
**C**	**C**	**C**	**C**	**C**	

V	Vincristin	1,5 mg/m²	iv-Bolus	Tag 1, 8 und 15 jedes Therapiezyklus
C	Carboplatin	550 mg/m²	1h-Infusion	Tag 1 jeder Therapiewoche

4.5.3. Randomisation
Therapiegruppe 1 und 2: Patienten ohne NF I und mit niedrigmalignen Gliomen der supratentoriellen Mittellinie, bzw. der cerebralen Hemisphären, des Kleinhirns, des kaudalen Hirnstammes und des Rückenmarkes werden zentral randomisiert zwischen der Standard- und intensivierten Induktionstherapie.
Die Randomisation wird stratifiziert für die Altersgruppen (< 1 Jahr, 1-8 Jahre, ≥ 8 Jahre) und gemäß des primären Tumorsitzes (reine chiasmatische Tumoren – (Dodge 2), alle anderen supratentoriellen Mittellinientumoren, Tumoren aller anderen Lokalisationen außerhalb der supratentoriellen Mittellinie).
Therapiegruppe 3: Patienten mit NF I und Tumoren aller Lokalisationen werden nicht randomisiert. Sie erhalten die Standardinduktionstherapie und die Konsolidierung.

4.6. Radiotherapie (Abschnitt 15)

Kinder, bei denen eine Radiotherapie durchgeführt wird, sollen eine Behandlung erhalten, die sich an modernen Therapieplanungs- und –Durchführungsempfehlungen bezüglich der Felder und Dosen (Gesamt und pro Fraktion) orientiert.

	Gesamtherd dosis	Dosis/Fraktion	Therapiezeit
Ältere Kinder: Gehirn	54	1,8 Gy	6 Wochen
Rückenmark	50,4	1,8 Gy	5 ½ Wochen
“Junge” Kinder: Gehirn	Die Rücksprache mit den nationalen Radiotherapiezentren wird empfohlen.		
Rückenmark			

5. Studienendpunkte

Alle Studienpatienten:	Durchführbarkeit der Therapie<br>Gesamtüberleben, progressionsfreies Überleben nach Diagnose
Beobachtungsgruppe:	Langzeitfolgen, Gesundheitsstatus, Lebensqualität
Therapiegruppe:	Progressionsfreies Überleben, ereignisfreies Überleben, Gesamtüberleben<br>Response auf die nicht-chirurgische Therapie<br>Langzeitfolgen, Gesundheitsstatus, Lebensqualität

6. Statistik

6.1. Kinder mit niedrigmalignen Gliomen aller Lokalisationen, die nicht an einer Neurofibromatose NF I leiden.

Das Ziel der Studie ist die **Standard-Induktionstherapie** mit Vincristin und Carboplatin mit der **intensivierten Induktionstherapie** mit Vincristin, Carboplatin und Etoposid zu vergleichen. Dies geschieht in Bezug auf das progressionsfreie Überleben bei Kindern ohne Neurofibromatose NF I, deren niedrigmalignes Gliomen gleich welcher Lokalisation eine Chemotherapie als nicht-chirurgische Behandlung erforderlich macht (entsprechend den Eingangskriterien (Abschnitt 9) und Indikationen für die nicht-chirurgische Therapie (Abschnitt 10)).

Diese Therapieoptimierungsstudie ist multinational, multizentrisch, nicht geblindet, randomisiert und prospektiv.

Der Rekrutierungszeitraum der Studie ist 8 Jahre gefolgt von einer Beobachtungszeit von 2 Jahren. Die Hauptfrage (progressionsfreies Überleben) wird auf einem Signifikanzniveau von $\alpha = 0{,}05$ analysiert. Die P-Werte für die Nebenfragestellungen werden als explorativ angesehen. Im Rahmen einer COX-Regression werden zuvor definierte Variablen auf ihren Einfluss in Bezug auf die Überlebensvariablen geprüft.

6.2. Kinder, die an einer Neurofibromatose erkrankt sind mit niedrigmalignen Gliomen aller Tumorlokalisationen:

Die Chemotherapie gemäß dem vorliegenden Protokoll wird eingesetzt, um den Beginn der Strahlentherapie hinauszuzögern oder eine Strahlentherapie zu vermeiden. Die statistische Analyse findet im Vergleich mit einer historischen Kontrollgruppe statt und kann nur beschreibend sein.

7. Ethische Aspekte

Tumorerkrankungen im Kindes- und Jugendalter sind selten. In den vergangenen zwanzig bis fünfundzwanzig Jahren wurden Fortschritte in der Behandlung dieser Leukämien und soliden Tumoren im wesentlichen durch sog. kooperative, multizentrische Therapieoptimierungsstudien erreicht, die zum einen garantiert haben, dass die Patienten die jeweils beste aktuelle Behandlung erhielten, und zum anderen durch die randomisierte Prüfung zusätzlicher, alternativer Ansätze eine Weiterentwicklung / Verbesserung einleiteten.
Kinder und Jugendliche, die nach Diagnose eines niedrigmalignen Glioms gemäß der Strategie der SIOP-LGG 2004-Studie behandelt werden, folgen einem heute international akzeptierten Studienansatz.

Da vor allem die Prognose der jüngeren Kinder auch mit Chemotherapie bezüglich der Tumorprogression mit ihren neurologischen Folgen noch unbefriedigend ist, erscheint es unter Abwägung der Belastungen und Nebenwirkungen gegen den potentiellen Nutzen einer Chemotherapie ethisch gerechtfertigt, den Wert der frühen Behandlungsintensivierung in randomisierter Form zu prüfen. Geeignete Überwachungsinstrumente stellen sicher, dass eine Gefährdung der Patienten durch die Studientherapie frühzeitig erkannt wird und der entsprechende Therapiarm dann geschlossen wird.

8. Ethikkommissionsvotum, Anzeige bei der Überwachungsbehörde

Die Gesamtstudie SIOP-LGG 2004 wurde den Ethikkommissionen des Klinikums Augsburg und der Ludwig-Maximiliansuniversität München (LMU) vorgelegt. Am 02.09.2004 erging ein positives Votum beider Kommissionen an die Studienleitung der GPOH Deutschland. Die von der Ethikkommission der LMU München gewünschten Ergänzungen wurden in den entsprechenden Protokollabschnitten vorgenommen.

Die teilnehmenden Behandlungszentren haben sicherzustellen, dass vor Aufnahme des ersten Patienten in die Studie ein für sie gültiges Ethikkommissionsvotum vorliegt. Da es sich bei dem vorgelegten Therapieoptimierungsprotokoll nicht um eine Arzneimittelstudie handelt, ist eine Meldung an die regional zuständigen Überwachungsbehörden nicht vorgesehen.

9. Erfassung unerwünschter schwerer Ereignisse (SAE) und Therapietoxizität

Bei Patienten, die eine nichtchirurgische Therapie erhalten, ist die Durchführung dieser Therapie (sowohl der Radiotherapie wie auch der Chemotherapie) auf den entsprechenden Formblättern zu dokumentieren.
Bei regulärem Therapieverlauf ist die maximale Ausprägung der Toxizität nach jedem Chemotherapiezyklus bzw. nach jeder Radiotherapieserie auf den zugehörigen Dokumentationsbögen vorzunehmen. Dabei werden die Common Toxicity Criteria (CTC) zugrunde gelegt, die im Abschnitt 22.11. detailliert aufgeführt sind. Für die Chemotherapie- und Radiotherapiedokumentation kann die Kurzversion der CTC-Kriterien, die den jeweiligen Dokumentationsbögen zugeordnet ist, genutzt werden.

Alle lebensbedrohenden therapiebezogenen Komplikationen, d. h. WHO/CTC-Toxizitäten °IV der folgenden Kategorien werden als schwere, unerwünschte Ereignisse (SAE) angesehen:

- Peripheres Nervensystem
- Zentrales Nervensystem
- Nieren
- Leber
- Herz
- Haut.

Zusätzlich werden folgende Komplikationen als SAE gewertet:

- Dauerhafte, relevante Behinderung, die einer anderen Toxizität folgt.
- Medikamentenüberdosierung.

Jedes schwere, unerwünschte Ereignis muss unmittelbar der nationalen Studienzentrale gemeldet werden, d. h. innerhalb des nächsten Arbeitstages. Das behandelnde Zentrum ist für die lückenlose Dokumentation des weiteren Verlaufes verantwortlich.
Schwerwiegende unerwünschte Ereignisse (SAE) sind unter Nutzung des Formblattes 22.12. an die Studienleitung zu melden:

Frau Dr. Astrid K. Gnekow
I. Klinik f. Kinder u. Jugendliche
Klinikum Augsburg
Stenglinstr. 2
86156 Augsburg
Tel.: 0821 - 400 - 3615
Fax: -3616
Email: gnekow.hit-lgg@klinikum-augsburg.de

Das Auftreten schwerer unerwünschter Ereignisse wird von der nationalen Studienleitung an das internationale Datenzentrum weitergeleitet und den anderen nationalen Studienleitungen von dort übermittelt. Gemäß den GCP-Richtlinien wird die für die Studienleitung zuständige Ethikkommission über das Auftreten schwerer unerwünschter Ereignisse informiert werden. Entsprechend seinen Aufgaben wird das Data Monitoring and Safety Committee (DMSC) in sechsmonatigen Intervallen über die unter Therapie auftretenden Toxizitäten informiert.

10. Patientenversicherung

Das Protokoll wird zur Förderung der Deutschen Kinderkrebsstiftung vorgelegt. Für diese Förderung ist die Erlangung des Qualitätssiegels A der Deutschen Krebsgesellschaft erforderlich. Mit Erteilung des Gütesiegels A wird die Probandenversicherung über die Gruppenversicherung der Deutschen Krebsgesellschaft beantragt.

4. Unterschriftenseite SIOP-LGG 2004

Studienleiter der SIOP-LGG 2003 Studie der GPOH Deutschland – Pädiatrische Onkologie

Frau Dr. Astrid K. Gnekow
I. Klinik für Kinder und Jugendliche
Klinikum Augsburg
Stenglinstr. 2
86156 Augsburg
Telefon: 0049 – (0)821 – 400 3615
Fax: 0049 – (0)821 – 400 3616
Email: KZVA.HIT-LGG@t-online.de

Studienleiter der SIOP-LGG 2003-Studie der GPOH Deutschland - Strahlentherapie

Herr Dr. R. D. Kortmann
Abt. Strahlentherapie „CRONA“
Universitätsklinikum Tübingen
Hoppe-Seyler-Str. 3
72076 Tübingen
Telefon: 0049 – (0)7071 – 29 82166
Fax: 0049 – (0)7071 – 29 5894
Email: rdkortma@med.uni-tuebingen.de

Verantwortlicher Biometriker:

Herr Dr. Andreas Faldum
~~Deutsches Kinderkrebsregister am~~IMBEI
der Universität Mainz
55101 Mainz
Telefon: 0049 – (0)6131 – 17 3938
Fax: 0049 – (0)6131 – 17 473938
Email: hitbiometrie@imbei.uni-mainz.de

22. Addendum 2 SIOP-LGG 2004

22.1. Votum der Ethikkommission

22.2. Teilnahmeerklärung des Therapiezentrums

22.3. Patienteninformation

22.4. Einverständniserklärungen
- 22.4.1. zur Studienteilnahme und Therapie
- 22.4.2. zur Datenweitergabe
- 22.4.3. zur Tumorgewebeasservierung

22. 5. Patientenregistration
- 22.5.1. Ersterhebungsbogen
- 22.5.2. Therapiebasisinformation

22. 6. Randomisierung
- 22.6.1. Randomisierung der Induktionstherapie
- 22.6.2. Randomisierungsergebnis

22.7. Chemotherapie: Therapiepläne
- 22.7.1. Induktionstherapie
  - I. Vincristin / Carboplatin
  - II. Vincristin / Carboplatin / Etoposid
- 22.7.2. Konsolidierung
  (Protokoll-Konsolidierung und alternative Konsolidierung bei Allergie oder früher Progression)
  - I. Vincristin / Carboplatin
  - II. Vincristin / Cisplatin
  - III. Vincristin / Cyclophosphamid

22.8. Chemotherapie: Dokumentation
- 22.8.1 Induktion
  - I. Vincristin / Carboplatin – Woche 1 – 24
  - II. Vincristin / Carboplatin / Etoposid – Woche 1 – 24
- 22.8.2. Konsolidierung
  - Protokoll-Konsolidierung – Woche 25 – 53
  - Protokoll-Konsolidierung – Woche 55 – 85
  - Alternative Konsolidierung nach Allergie – Woche 1 - 30
  - Alternative Konsolidierung nach Allergie – Woche 31 - 61
- 22.8.3. Toxizitätsdokumentation
- 22.8.4. Responsebeurteilung

22.9. Radiotherapie: Dokumentation
- 22.9.1. Patientendaten. Maximale, akute Toxizität während der Strahlentherapie
- 22.9.2. Maximale akute Morbidität am Ende der Strahlentherapie
- 22.9.3. Myelotoxizität während der kranio-spinalen Bestrahlung
- 22.9.4. Therapietechnik – Primärtumor.
- 22.9.5. Dosisverschreibung – kraniospinale Achse
- 22.9.6. Therapietechnik – kraniospinale Bestrahlung
- 22.9.7. Strahlentherapie für metastatische Absiedlungen
- 22.9.8. Brachytherapie: Therapietechnik / Dosisverschreibung

22.9.9. Tumorresponse
22.9.10. Spätfolgen der Radiotherapie
22.9.11. Rezidivmeldung

22.10. Materialversand
22.10.1. Neuropathologie
22.10.1.1. Anleitung zur Asservierung von Tumorgewebe
22.10.1.2. Einsendebogen Hirntumorreferenzzentrum Bonn
22.10.1.3. Einsendebogen Tumorbank
22.10.2. Neuroradiologie
22.10.2.1. Einsendebogen Referenzradiologie – Erstuntersuchung
22.10.2.2. Einsendebogen Referenzradiologie – frühpostoperative Untersuchung
22.10.2.3. Einsendebogen Referenzradiologie - Verlaufsuntersuchung

22.11. Toxizitätsklassifikation laut CTC

22.12. Meldung schwerwiegender unerwünschter Ereignisse

22.13. Follow-up-Formblätter
22.13.1 Verlaufsdiagnostik
22.13.2. Statusabfrage
22.13.3. Ereignismeldung
22.13.4. Befunderhebung bei Diagnose: Neurologie, Endokrinologie und Entwicklung
22.13.5. Endokrinologie – Verlauf
22.13.6. Ophthalmologische Dokumentation – Initial und im Verlauf
22.13.7. Neurologische Befunderhebun - (Initial und) im Verlauf
22.13.8. Befunderhebung nach Behandlungsende: Spätfolgen und Entwicklung
21.13.9. Anhang: Funktion, Lebensqualität Verhalten: Fertigkeitenskala FMH

22.14. Wissenschaftliche Begleituntersuchungen
22.14.1. Pilocytische Astrocytome mit klinisch atypischem Verlauf – Histologisch atypische und maligne Formen. (T.Pietsch, Bonn)
22.14.2. Gesundheitsbezogene Lebensqualität bei Kindern und Jugendlichen, die mit SIOP-LGG 2004 behandelt werden (G. Calaminus, Düsseldorf)
22.14.3. Therapieassoziierte Spätfolgen nach Strahlentherapie maligner Erkrankungen im Kindes- und Jugendlichenalter (N. Willich, A. Schuck, Münster))

22.15. Liste der (bisher) teilnehmenden Kliniken

Anleitung für Korrekturen der Eintragungen auf Erhebungsbögen

Die Bögen sind mit Tinte oder Kugelschreiber auszufüllen, Bleistifteintragungen sind nicht erlaubt. Korrekturen sind wie folgt vorzunehmen: Der falsche Eintrag wird mit einer einfachen Linie durchgestrichen, die korrekte Information daneben eingetragen und vom Prüfarzt mit Datum paraphiert und ggf. mit Angabe des Grundes der Korrektur versehen. Datenfelder, die wegen fehlender Information nicht ausgefüllt werden können, sind zu kommentieren. Die Bögen sind zeitnah auszufüllen, mit Datum zu unterschreiben und der Studienzentrale zuzuleiten.

Datumsangaben

Kalenderdaten sollen gemäß den Vorgaben auf den Erhebungsbögen notiert werden. Im allgemeinen erfolgt zunächst die Angabe des Tages, dann des Monats und zuletzt des Jahres, wobei die Jahreszahlen vierstellig ausgeschriebenen werden sollen, außer wenn anders vorgegeben: TT MM JJJJ.

22.1. Votum der Ethikkommission SIOP-LGG 2004

Klinikum Augsburg
Akademisches Lehrkrankenhaus der Ludwig-Maximilians-Universität München

DER VORSITZENDE DER ETHIKKOMMISSION

Frau
OÄ Dr. A.K. Gnekow
I. Klinik für Kinder und
Jugendliche

Am 02.09.2003/P

VOTUM

Antrag an die Ethikkommission von Frau OÄ Dr. A.K. Gnekow, I. Klinik für Kinder und Jugendliche,
„HIT-LGG-Studie – Low-grade-glioma-Study“

Die Mitglieder der Ethikkommission stimmen nach Prüfung der ethischen und rechtlichen Probleme Ihrem Antrag zu.

Unabhängig von der Zustimmung der Ethikkommission machen wir Sie aber darauf aufmerksam, daß die ethische und rechtliche Verantwortung für die Durchführung einer klinischen Prüfung bei dem Leiter und bei allen an der Prüfung teilnehmenden Ärzten liegt. Außerdem weisen wir Sie darauf hin, daß die Ethikkommission über die Beendigung der Studie, einen Studienabbruch und über schwerwiegende unerwünschte Ergebnisse im Verlauf der Studie zu unterrichten ist.

Mit freundlichem Gruß

Prof. Dr. med. A. Wischnik
Vorsitzender der Ethikkommission

23 03 05.01 Art.-Nr. 400-7646

Anschriften
Zentralklinikum Augsburg
Stenglinstraße 2
86156 Augsburg

Klinik für Kinder und Jugendliche
Stenglinstraße 2
86156 Augsburg

Krankenhaus Haunstetten
Sauerbruchstraße 6
86179 Augsburg

Fon/Fax Zentralen
Zentralklinikum und Klinik für Kinder und Jugendliche
Telefon 0821 - 400 - 01/02
Telefax 0821 - 400 - 20 20

Krankenhaus Haunstetten
Telefon 0821 - 400 - 03
Telefax 0821 - 400 - 72 08

Kommunalunternehmen Zentralklinikum Augsburg & Krankenhaus Haunstetten • Anstalt des öffentlichen Rechts

Ethikkommission der Medizinischen Fakultät
der Ludwig-Maximilians Universität
Vorsitzender: Prof. Dr. Gustav Paumgartner

Ludwig-Maximilians-Universität München

Klinikum der Universität München – Großhadern
Marchioninistr. 15 81377 München

Marchioninistr. 15
81377 München
Tel: (089) 7095 4609
Fax: (089) 7095 7609
e-mail: Ethikkommission@med2.med.uni-muenchen.de

07.10.2003

Herrn
Prof. Dr. A. Wischnik
Ethikkommission Klinikum Augsburg
Stenglinstr. 2
86156 Augsburg

Antrag an die Ethikkommission Augsburg von Frau Oberärztin Dr. A. K. Gnekow, Klinik für Kinder und Jugendliche
„HIT-LGG-Studie – Low-grade-glioma-Study"

Sehr geehrter Herr Kollege Wischnik,

Besten Dank für Ihr Schreiben vom 2.9.2003 mit der Zusendung des o.g. Antrags von Frau Dr. A. K. Gnekow zusammen mit den entsprechenden Unterlagen und dem positivem Votum Ihrer Ethikkommission.

Dem positiven Votum Ihrer Kommission kann zugestimmt werden. Gleichzeitig werden folgende Verbesserungen angeregt:

1. Da in der Studie außer jüngeren (> 8 Jahre) auch ältere (< 8 Jahre) Kinder und Jugendlich eingeschlossen werden sollen und damit prinzipiell auch eine mögliche Schwangerschaft zu berücksichtigen ist, sollte angesichts der langen Chemotherapie-Behandlung der entsprechende Passus im Antrag und in der Patienten/Elterninformation wie folgt ergänzt werden:

   a. Bei konzeptionsfähigen Patientinnen muss vor Studieneinschluß ein HCG-Schangerschaftstest durchgeführt werden.

   b. Es muss durch geeignete Antikonzeptionsmethoden (detailliert aufführen) eine Schwangerschaft während der Studie zuverlässig verhindert werden.

2. Es sollte die gewählte Pseudonymisierungsmethode vor allem wegen der Einspeisung von Daten in ein überregionales Kompetenznetz exakter beschrieben und mit den heutzutage möglichen Konzepten des Datenschutzes in wissenschaftlichen Netzwerken abgeglichen werden.

FR 3 325/00 Mä.

Prof.Dr.A.Wischnik, Antrag HIT-LGG-Studie vom 7.10.03

Mit freundlichen Grüßen

Prof. Dr. G. Paumgartner
Vorsitzender der Ethikkommission

cc. Mitglieder der Ethikkommission
Prof. Dr. Eckhard Held
Prof. Dr. Detlef Kunze
Dr. Viktoria Mönch
PD Dr. V. Nüßler
Prof. Dr. Randolph Penning

P.S.: Für zukünftige Anträge an die EK siehe: Richtlinien für den Antrag an die Ethikkommission (Version 21.06.2002). Internetadresse: http://www.med.uni-muenchen.de/Ethikkommission .

22.2. Teilnahmeerklärung des Therapiezentrums an der SIOP-LGG 2004-Studie

Behandlung des niedriggradigen Glioms entsprechend der Therapiestudie SIOP–LGG-2004

Name des an der Klinik tätigen und für die SIOP-LGG 2004 verantwortlichen Arztes:

____

Klinik: ______________________________________

- Patienten im Alter von 0 – 18 Jahren mit einem niedrig-gradigen Gliom unabhängig von dessen Lokalisation werden in die Studie eingebracht.
- Die Protokollrichtlinien werden eingehalten unter Berücksichtigung der ärztlichen Verantwortung im Einzelfall.
- Die angeforderten Informationen, Präparate und Frischmaterialen (molekularbiologische Untersuchungen) werden von jedem Patienten zur Verfügung gestellt.

		Unterschrift
**Kinderonkologe**	Name	
	Adresse..	
	Tel.:................./..................... Fax....................../......................	
	E-mail..	
**Neurochirurg**	Name..	
	Adresse..	
	Tel.:................./..................... Fax....................../......................	
	E-mail..	
**Radiotherapeut**	Name	
	Adresse..	
	Tel.:................./..................... Fax....................../......................	
	E-mail..	
**Pathologe**	Name	
	Adresse..	
	Tel.:................./..................... Fax....................../......................	
	E-mail..	

Bitte zurück an: SIOP-LGG 2004 – Studie, Frau Dr. Gnekow, Stenglinstr. 2, 86156 Augsburg
FAX: 0821 – 400 – 3616

22.3. Patienteninformation SIOP-LGG 2004

Sie sind heute darüber informiert worden,
dass bei Ihnen / Ihrer Tochter / Ihrem Sohn ....
die Diagnose eines ....
gestellt wurde.

Dieser Tumor gehört zur Gruppe der niedrigmalignen Gliome, für die eine Therapiestrategie gemäß den Empfehlungen der Gesellschaft für pädiatrische Onkologie und Hämatologie (GPOH) in Zusammenarbeit mit anderen nationalen Studiengruppen innerhalb der Internationalen Gesellschaft für Pädiatrische Onkologie und Hämatologie (SIOP) erstellt wurde. Bevor Sie einwilligen, dass Sie/Ihr Kind im Rahmen der Studie SIOP-LGG 2004 behandelt werden/wird, lesen Sie bitte aufmerksam die folgenden Informationen über die Grundlagen, Ziele und die Durchführung der Studie. Markieren Sie Abschnitte, die Sie nicht verstanden haben, und die im Aufklärungsgespräch noch einmal besonders erklärt werden müssen.

I. Grundlagen

Hirntumoren sind mit ca. 20% aller Krebserkrankungen die häufigsten Tumoren im Kindesalter. In der Bundesrepublik erkranken jährlich etwa 350 Kinder unter 15 Jahren neu an einem Hirntumor. Die Ursache für das Entstehen eines Hirntumors (z. B. Krebsgene) und die Hintergründe ihres Verhaltens sind noch weitgehend unbekannt. Weitergehende wissenschaftliche Untersuchungen an Tumormaterial, Blut und Hirn-Rückenmark-Flüssigkeit (Liquor) sind daher erforderlich.
Die größte Gruppe von Hirntumoren im Kindesalter sind mit 30-40% die sogenannten Gliome niedrigen Malignitätsgrades (was als „geringere Bösartigkeit“ übersetzt werden kann). Sie sind charakterisiert durch ihr langsames Wachstum und auch durch längere Perioden ohne Wachstumstendenz. Einige Tumoren können jedoch zeitweise ein schnelleres Wachstumsverhalten aufweisen mit zunehmenden und schweren neurologischen Symptomen.

Niedrigmaligne Gliome treten in allen Altersgruppen auf, am häufigsten zwischen 6 und 11 Jahren. Einzelne Diagnosegruppen häufen sich jedoch in bestimmten Altersgruppen. Eine Besonderheit der niedrigmalignen Gliome ist ihr Auftreten im Rahmen von sogenannten Phakomatosen (Sammelbegriff für angeborene Missbildungskrankheiten mit Fleckenbildungen an Augen, Haut und anderem Gewebe). Bei der Neurofibromatose Typ NF I werden z. B. laut Literaturangaben bei 15-20% aller Patienten Tumoren der Sehbahn entdeckt. Die Häufigkeit anderer gleichzeitig oder nachfolgend entdeckter Tumoren im Gehirn ist nicht sicher bekannt. Bei der tuberösen Sklerose treten bevorzugt sogenannte Riesenzellastrozytome in den Seitenhohlräumen des Gehirnes auf.

Auch Gliome niedrigen Malignitätsgrades wachsen in gesundes Hirngewebe hinein und können sich entlang der Hirnhäute und über die Hirn-Rückenmark-Flüssigkeit ausbreiten. Oft gibt es eine klare Grenzschicht zwischen dem Tumor und seiner Umgebung. Dann kann die alleinige operative Entfernung (Resektion) des Tumors zu einer Heilung führen. Die Ausbreitung (Metastasierung) im Nervensystem ist selten bei Diagnosestellung, häufiger jedoch im Rückfall und wird mit 5-10% angegeben.

Ein nicht unerheblicher Teil der niedrigmalignen Gliome ist in Gehirnarealen lokalisiert, in denen eine vollständige Operation (siehe unten) nicht möglich ist. Für sie müssen daher andere Behandlungsmöglichkeiten gewählt werden. Da besonders bei jungen Kindern das noch nicht voll ausgereifte Gehirn sehr empfindlich gegenüber schädigenden Einflüssen des Tumors wie

auch der Behandlung ist, sind Langzeitfolgen, wie z. B. Störungen der geistigen Entwicklung, des Kurzzeitgedächtnisses und der Konzentration sowie der normalen Hormonbildung mit der Folge einer Verminderung des Körperlängen-Wachstums oder einer Schilddrüsenunterfunktion bei ihnen besonders häufig und ausgeprägt. Dadurch wird die Lebensqualität erheblich beeinträchtigt. Da die großräumige Bestrahlung des Gehirns eine der Ursachen dieser Langzeitfolgen ist, ist man derzeit im Rahmen von Therapiestudien bestrebt, die Bestrahlung durch eine Chemotherapie bei möglichst vielen jungen Kindern vollständig oder teilweise zu ersetzen.

Kinder mit Krebserkrankungen sollten immer im Rahmen von kontrollierten Studien behandelt werden, da in den Studien die bestmögliche Untersuchungs- und Behandlungsverfahren nach dem neuesten Stand internationaler Erkenntnisse eingesetzt werden. Die Behandlung der niedrig-bösartigen Hirntumoren besteht in der Regel aus der Operation, im Einzelfall kombiniert mit Bestrahlung und/oder Chemotherapie. Die meisten Patienten mit niedrigmalignen Gliomen überleben für lange Jahre. Besonders bei nicht vollständig operablen Tumoren entwickeln sich Tumorwachstums- und Tumorstillstandsphasen jedoch über Jahre und machen eine vernünftige Therapieplanung von Anfang an erforderlich. Um für alle Kinder zu einer einheitlichen Therapiestrategie zu kommen, entschlossen sich die Kinderkrebsspezialisten in der Bundesrepublik gemeinsam mit denen anderer europäischer Länder innerhalb einer Arbeitsgruppe, für Kinder mit niedrigmalignen Gliomen ein verbindliches Therapiekonzept zu erstellen.

Die Studie SIOP-LGG 2004 sieht vor, allen Kindern und Jugendlichen mit niedrigmalignen Gliomen eine nach dem derzeitigen Stand der Erkenntnisse bestmögliche und ausgewogene Behandlung zu bieten. Dazu baut die Studie auf den Ergebnissen der vorangegangenen Studie SIOP/GPOH HIT-LGG 1996 und anderen internationalen Studienergebnissen auf.

II. Operation

Die sofortige Operation ist bei Kindern, die sich durch den Tumor in einer lebensbedrohlichen Lage befinden, von großer Bedeutung. Am so gewonnenen Tumormaterial kann dann durch die feingewebliche Untersuchung die Art des Tumors festgestellt werden. Ziel der Operation ist die operationsmikroskopisch vollständige Entfernung des Tumors. Abhängig von der Größe des Tumors, seinem Ausgangspunkt und seiner Ausdehnung kann aber häufig ein chirurgischer Eingriff bei niedrigmalignen Gliomen keine vollständige Entfernung erreichen. Denn ein schonungsloses neurochirurgisches Vorgehen, das zu schweren, lebenslangen Schäden führt, verbietet sich. Im Zweifelsfall muss eher ein Resttumor belassen werden, der dann mit Chemo- oder Strahlentherapie behandelt werden kann, wenn er eine neuerliche Wachstumstendenz aufweist oder schwere Symptome vorliegen. Manchmal ist sogar nur eine Probeentnahme möglich, unter bestimmten Umständen kann auch auf einen chirurgischen Ersteingriff verzichtet werden und die Diagnose aufgrund der Befunde der Bildgebung erhoben werden.

Das Ausmaß und die Technik eines chirurgischen Ersteingriffes werden mit Dir / Ihnen ausführlich besprochen. Frühzeitig nach der Operation ist eine bildgebende Untersuchung mit Magnetresonanztomographie (MRT) oder in Ausnahmefällen Computertomographie (CT) erforderlich, um das Ausmaß dieses Eingriffes zu dokumentieren.

III. Nicht-chirurgische Therapie

In Abhängigkeit von dem Grad der erreichten Tumorentfernung sowie dem Vorhandensein oder dem Fehlen schwerer neurologischer Symptome und dem Vorhandensein oder dem Fehlen klinischer oder neurologischer Zeichen des Tumorwachstums wird über die weitere Behandlung entschieden. Somit ergibt sich eine Gruppierung der Patienten in drei Untergruppen:

1.) Patienten, die zunächst nur beobachtet werden
2.) Patienten, die eine sog. nicht-chirurgische Behandlung nach Diagnosestellung benötigen und
3.) Patienten, die zunächst beobachtet werden können, dann jedoch bei Verschlechterung neurologischer Symptome oder Vergrößerung des Tumors (im Rahmen von regelmäßigen bildgebenden Kontrolluntersuchungen) behandelt werden müssen.

1.) Beobachtungsgruppe:
Wenn durch den neurochirurgischen Ersteingriff der Tumor vollständig entfernt werden konnte, ist eine abwartende Haltung gerechtfertigt, da der größte Teil dieser Kinder und Jugendlichen im Folgenden keiner zusätzlichen Behandlung bedarf. Wenngleich bei den meisten dieser Kinder die Tumorfreiheit mit Heilung gleichgesetzt werden kann, muss bei einem kleinen Prozentsatz mit einem Rückfall gerechnet werden. Da derartige Rezidive auch noch nach Jahren auftreten können, ist eine Überwachung für mindestens 8 – 10 Jahre anzuraten.

Bei Kindern mit inkompletter Tumorentfernung, aber ohne schwere Symptome und ohne Zeichen des Tumorwachstums kann ebenfalls zunächst beobachtet werden. Allerdings muss bei den meisten Kindern mit späterem Tumorwachstum gerechnet werden. In allen Fällen ist eine regelmäßige Verlaufsdiagnostik mit bildgebenden Untersuchungsverfahren notwendig, um das Wachstum oder das Wiederauftreten des Tumors zu einem späteren Zeitpunkt rechtzeitig zu erkennen. Es hat sich nicht als nachteilig herausgestellt, auch Kinder mit Resttumoren zunächst zu beobachten, da das Wachstumsverhalten dieser Tumoren nur sehr langsam zu Veränderungen führt und somit auch ein Fortschreiten der Erkrankung rechtzeitig durch die bildgebenden Untersuchungen erfasst wird.

2.) Patienten, die eine sogenannte nicht-chirurgische Behandlung nach Diagnosestellung benötigen:
Selbst wenn davon ausgegangen werden kann, dass ein Resttumor nach einer Tumoroperation nicht oder nur sehr langsam wächst, so gibt es klinische Ausgangsbedingungen, bei denen gemäß aller Erfahrung eine Chemo- oder Radiotherapie erforderlich ist. Hierzu gehören z. B. Tumoren, die zu dem klinischen Bild eines sogenannten diencephalen Syndroms geführt haben. Das ist eine Befundkonstellation, die durch einen besonderen Typ der körperlichen Auszehrung charakterisiert ist. Auch Tumoren, die bereits bei Diagnosestellung Absiedlungen (Metastasen) hatten, die sich durch neurologische Ausfälle bemerkbar machen, benötigen eine unmittelbare Behandlung.

3.) Bei einem erheblichen Teil der Kinder, die nur eine Tumorteilentfernung hatten, kann zunächst eine Beobachtung an die Operation angeschlossen werden. Dieses beobachtende und abwartende Verhalten setzt jedoch voraus, dass regelmäßige Befundkontrollen in einem pädiatrisch-onkologischen Zentrum durchgeführt werden. Änderungen, wie radiologisch nachweisbares Tumorwachstum oder die zunehmende klinische Symptomatik begründen dann den verzögerten Beginn einer nicht-chirurgischen Therapie.

Die Behandlung mit Zellgiften (Chemotherapie)
Die Chemotherapie von Gliomen niedrigen Malignitätsgrades hat das Ziel, eine eventuell notwendige Strahlentherapie hinauszuschieben oder gänzlich zu vermeiden. Gemäß den Erfahrungen der Vorläuferstudie und den Literaturdaten ist bei einem großen Teil der Patienten (über 80%) mit einem Ansprechen auf diese Chemotherapie zu rechnen. Dabei kommt es bei etwa der Hälfte der ansprechenden Patienten zu einer tatsächlichen Tumorvolumenverkleinerung, während bei der anderen Hälfte der Kinder ein Gleichbleiben der Tumorgröße erreicht wird. Ein kleiner Teil der Patienten spricht nicht auf die erste gewählte

Chemotherapie an. Eine Verhinderung des Tumorwachstums gelingt für die meisten Kinder über 3-5 Jahre, im Einzelfall ist dies jedoch sehr schlecht vorauszusehen.
Bislang wurden sehr viele verschiedene Chemotherapie-Kombinationen bei niedrigmalignen Gliomen eingesetzt. In Europa hat sich die Behandlung mit den Medikamenten Carboplatin und Vincristin als Standard etabliert. Für viele Patienten, besonders wenn die Tumoren im Bereich des Zwischenhirnes angesiedelt sind, kommt es jedoch sehr bald wieder zu einem Tumorwachstum, so dass die Langzeitergebnisse mit dieser Standardbehandlung noch nicht befriedigend sind. Andererseits ist unklar, auf welchem Weg die Wachstumstendenz niedrigmaligner Tumoren am besten verhindert werden kann. Auch Therapiestudien mit weniger und höher intensiven Chemotherapie-Kombinationen haben vergleichbare Ergebnisse wie die SIOP/GPOH HIT-LGG-1996-Studie erbracht. Es deutet sich an, dass eine Therapieverlängerung günstig ist. Daher soll im Vergleich zur Vorläuferstudie die Behandlung für alle Kinder verlängert werden. Um aber insbesondere das frühzeitige Weiterwachsen (Progression) der Tumoren zu verhindern, soll geprüft werden, ob die zusätzliche Gabe eines dritten Medikamentes in den ersten zehn Therapiewochen die spätere progressionsfreie Zeit verlängert.

Uns ist bewusst, dass die Chemotherapie nicht ohne Nebenwirkungen durchführbar ist. Nach unserer Einschätzung ist jedoch die Chance, dass kleine Kinder durch eine Chemotherapie einen Vorteil gegenüber einer frühzeitigen Strahlentherapie haben, größer, als dass ihnen mit dieser Therapie bleibend geschadet wird.

Da Chemotherapie während einer Schwangerschaft zu Schädigungen der Frucht führen kann, muss bei konzeptionsfähigen Patientinnen zu Beginn einer Chemotherapie das Vorliegen einer eventuellen Schwangerschaft durch einen Schwangerschaftstest ausgeschlossen und für die gesamte Dauer der Therapie der Eintritt einer Schwangerschaft zuverlässig verhindert werden. Der Einsatz geeigneter Methoden (z. B. Minipille, hormonhaltiger Scheidenring, Hormonpflaster) sind unter Berücksichtigung des Einzelfalles gesondert zu besprechen.

Die Nebenwirkungen der Chemotherapie
Bei der Chemotherapie werden hochwirksame Zellgifte verabreicht, die den ganzen Organismus des Kindes und Jugendlichen treffen. Außer Haarausfall können folgende Organe in ihrer Funktion gestört werden: Schleimhäute, Knochenmark (Blutbildung), Infektabwehr, Nieren, Gehör, Gehirn und Nervensystem, Leber, Lunge und Eierstöcke/Hoden. Sehr selten können nach einer solchen Behandlung auch Zweittumoren auftreten.

Bei bis zu 30% der Patienten kann sich zu irgendeinem Zeitpunkt der Therapie, meist erst in der Erhaltungstherapiephase (Konsolidierung), eine Allergie gegen Carboplatin entwickeln, die eine Weiterbehandlung mit diesem Medikament nicht erlaubt. Da eine Gesamtbehandlungszeit von 18 Monaten angestrebt wird, wird die Therapiefortsetzung mit alternativen Medikamentenkombinationen empfohlen. Die Wirksamkeit dieser Alternativmedikamente wurde bereits belegt. Cis-Platin und Cyclophosphamid sollen nach Auftreten einer Carboplatinallergie im Wechsel mit Vincristin bei Beibehaltung der Zyklusintervalle gegeben werden.

Den möglichen Nebenwirkungen einer Chemotherapie wird durch eine Dosierung, die sich nach dem Alter und der Körperoberfläche richtet und eine zeitlich genaue Abfolge der Medikamentengaben Rechnung getragen. Vorbeugende Maßnahmen (z. B. gegen Übelkeit und Erbrechen) sollen die Nebenwirkungen in erträglichen Grenzen halten oder teilweise völlig verhindern. Die Nebenwirkungen und möglichen Komplikationen der Chemotherapie müssen jedoch vor dem Hintergrund in Kauf genommen werden, dass die Zeit, in der der Tumor nach

Operation und Chemotherapie nicht wächst, verlängert werden soll/muss und die Langzeitfolgen nach der Bestrahlung bei jungen Kindern nicht akzeptabel sind.

Die Strahlentherapie
Eine auf den Tumor gerichtete Bestrahlung wurde lange Jahre als einzige Möglichkeit gesehen, das Tumorwachstum niedrigmaligner Gliome zu stoppen. Bei den oft großen Tumoren mussten dabei große Felder bestrahlt werden, da erhebliche Sicherheitssäume um die Tumoren gezogen wurden. Damit wurden auch große Areale gesunden Gehirnes mitbestrahlt. Dies führte insbesondere bei den im Zwischenhirn gelegenen Tumoren zu den eingangs beschriebenen Spätfolgen.

Es ist nun vorstellbar, dass durch neuere Anwendungsmöglichkeiten der Strahlentherapie bei gleich guter Tumorwirkung die Belastung des gesunden, umgebenden Gehirngewebes vermindert werden kann. Neuere Techniken erlauben eine bessere Strahlentherapieplanung. Da die niedrigmalignen Gliome meist nur wenige Millimeter in umgebendes Gehirngewebe einwachsen, ist ein breiter Sicherheitssaum auch nicht erforderlich. Somit kann bei entsprechender Lagerung des Patienten und fester Haltung die heutige Strahlentherapie sehr zielgenau gegeben werden. Diese Bestrahlungstechniken sind daher ein besonderer Vorteil bei kleinen Tumoren und älteren Kindern. Bei kleinen Kindern kann in Ausnahmefällen bei Versagen der Chemotherapie eine Strahlentherapie mit ähnlicher Technik vorgesehen werden. Die Frage, ob ein Tumor zusätzlich oder ausschließlich durch radioaktives, implantierbares Material (interstitielle Radiotherapie / Brachytherapie) behandelt werden kann, hängt sehr stark von der Größe des Tumors, seinem Sitz und dem Wachstumsverhalten des Tumors ab.

Das Vorhandensein einer Neurofibromatose Typ NF I scheint das Ausmaß und den Schweregrad von strahlenbedingten Spätschäden zu erhöhen, so dass für diese Patientengruppe die Vermeidung der Bestrahlung in allen Altersgruppen gerechtfertigt ist.

Die Nebenwirkungen der Strahlenbehandlung
Zu Beginn der Bestrahlung können durch die Reizung der Hirnhäute Hirndrucksymptome auftreten, die sich durch Kopfschmerzen, Übelkeit und Erbrechen äußern können. Neben lokalen Reizerscheinungen der Haut im Bestrahlungsfeld, wie Trockenheit und Rötung, kommt es im Bereich der behaarten Haut zumeist zum Haarausfall. In Abhängigkeit von den Feldgrößen und der Lokalisation können auch andere Reizsymptome auftreten. Die Knochenmarkfunktion kann bei Behandlung des gesamten zentralen Nervensystems (z. B. bei Metastasen) eingeschränkt werden. An strahlenbedingten Spätfolgen können altersabhängige Verkürzungen des Längenwachstums bei Bestrahlungen der Wirbelsäule auftreten, ferner sind auch Langzeitnebenwirkungen an den gesunden, jedoch mitbestrahlten Organen Gehirn, Auge, Ohr, Gefäße und Hirnanhang– sowie Schilddrüse trotz optimierter Bestrahlungsplanung und exakter Dosierung nicht vollständig auszuschließen. Dies kann möglicherweise die lebenslange Einnahme von Medikamenten, psychointellektuelle Entwicklungsstörungen oder neurologische Einschränkungen einschließlich Lähmungen zur Folge haben. Selten können nach einer Bestrahlung auch Zweittumoren auftreten.

Der verantwortliche Radiotherapeut wird zusätzlich ein studienbezogenes Informationsgespräch führen. Das Einverständnis für die Strahlentherapie muss mit den üblichen Aufklärungsbögen zusätzlich und getrennt eingeholt werden.

Ziele dieser Therapiestudie
Mit dieser Studie soll zunächst für alle Kinder mit niedrigmalignen Gliomen von vornherein eine umfassende Therapieplanung möglich sein. Die meisten Kinder mit niedrigmalignen Gliomen überleben für viele Jahre. Kinder nach vollständiger Tumorentfernung haben nur ein

geringes Rückfallrisiko und können bei Rezidivfreiheit nach 6-10 Jahren als geheilt angesehen werden.

Für den großen Teil der Kinder mit nicht vollständig entfernbaren Tumoren sollen die Chancen, ohne schwere Langzeitschäden zu überleben, verbessert werden. Bei Kindern, die einer Bestrahlung zugeführt werden, soll dies erreicht werden durch die Anwendung neuester Strahlentherapieplanungssysteme und Bestrahlungstechniken. Bei Kindern, die zunächst eine Chemotherapie erhalten, soll dies durch eine Verlängerung der Chemotherapie bei allen Kindern sowie die Prüfung der Auswirkungen einer Intensivierung der Anfangstherapie erreicht werden. Insgesamt wird eine vergleichsweise geringe Rate und Ausprägung möglicher Spätfolgen der Therapie mit einer gebesserten Lebensqualität der Patienten angestrebt, besonders durch den Verzicht auf eine Bestrahlung bei jüngeren Kindern.

Eine Qualitätskontrolle der Behandlungsarten Operation, Bestrahlung und Chemotherapie, eine standardisierte Diagnostik und Nachsorge gewährleisten für alle Kinder eine hohe Behandlungsqualität. Die Aspekte der Langzeitfolgen und Lebensqualität sollen Informationen darüber liefern, wie sich die durchgeführten Behandlungsmaßnahmen auf das weitere Leben der Kinder auswirken, da zu diesem Aspekt wenig systematische Untersuchungen vorliegen.

Ethische Aspekte

Tumorerkrankungen im Kindes- und Jugendalter sind selten. In den vergangenen zwanzig bis fünfundzwanzig Jahren wurden Fortschritte in der Behandlung dieser Leukämien und soliden Tumoren im wesentlichen durch sog. kooperative, multizentrische Therapieoptimierungsstudien erreicht, die zum einen garantiert haben, dass die Patienten die jeweils beste aktuelle Behandlung erhielten, und zum anderen durch die randomisierte Prüfung zusätzlicher, alternativer Ansätze eine Weiterentwicklung / Verbesserung einleiteten.

Kinder und Jugendliche, die nach Diagnose eines niedrigmalignen Glioms gemäß der Strategie der SIOP-LGG 2004-Studie behandelt werden, folgen einem heute international akzeptierten Studienansatz.

Da vor allem die Prognose der jüngeren Kinder auch mit Chemotherapie bezüglich der Tumorprogression mit ihren neurologischen Folgen noch unbefriedigend ist, erscheint es unter Abwägung der Belastungen und Nebenwirkungen gegen den potentiellen Nutzen einer Chemotherapie ethisch gerechtfertigt, den Wert der frühen Behandlungsintensivierung in randomisierter Form zu prüfen. Geeignete Überwachungsinstrumente stellen sicher, dass eine Gefährdung der Patienten durch die Studientherapie frühzeitig erkannt wird und der entsprechende Therapiarm dann geschlossen wird.

Die Behandlungspläne

Die Behandlung erfolgt nicht für alle Studienpatienten einheitlich, sondern richtet sich nach dem Tumorsitz, dem Alter und dem Vorhandensein oder Fehlen einer Neurofibromatose Typ NF I. Bitte beachten Sie daher insbesondere die Hinweise zur Behandlung für die Patientengruppe, der Sie / Ihr Kind zugeordnet wurden/wurde.

1.) Niedrigmaligne Gliome des Zwischenhirnes bei Kindern ohne Neurofibromatose.

Die sogenannten hypothalamisch-chiasmatischen Gliome oder Sehbahngliome sind eine relativ homogene Gruppe niedrigmaligner Gliome. Zusätzlich werden die kleine Anzahl von Tumoren der Basalganglien, des Thalamus und des Mittelhirnes hierzu gezählt, da sie ähnliche therapeutische Schwierigkeiten darstellen. Die Tumoren dieser Lokalisation sind meist nicht operativ zu entfernen. Es handelt sich meist um sehr kleine Kinder, so dass der größte Teil behandlungsbedürftiger Patienten eine Chemotherapie erhält. Um die Strahlentherapie möglichst effektiv hinauszuschieben, soll geprüft werden, ob eine Intensivierung der

Anfangsbehandlung (Induktion) das Risiko des späteren Weiterwachsens des Tumors (Progression) vermindert, d. h. ob sich diese Progressionsrate unterscheidet von der, die durch die Standardinduktion erreicht wird.

Die Zuordnung zur Standardinduktion oder intensivierten Induktion erfolgt durch eine zentrale Randomisierung. Eine solche zufällige Verteilung von Patienten durch Losentscheidung auf zwei Behandlungsarme, die miteinander verglichen werden sollen, ist erforderlich, um eine Verfälschung der Studienergebnisse durch äußere Einflüsse zu vermeiden.

Es kann derzeit nicht vorausgesagt werden, ob das progressionsfreie Überleben dieser Patientengruppe durch die intensivierte Induktion tatsächlich verbessert werden kann im Vergleich zur Standardinduktion. Sollte sich jedoch im Verlauf der Studie ein deutlicher Unterschied ergeben, der sowohl die Wirkung wie auch die Nebenwirkungen betreffen kann, wird die Randomisierung abgebrochen und alle Patienten werden in den erfolgreicheren/schonenderen Behandlungsarm aufgenommen. Im Standardinduktionsarm erhalten alle Kinder wöchentliches Vincristin und 3-wöchiges Carboplatin über zehn Wochen. Im intensivierten Induktionsarm wird zusätzlich zu Carboplatin in 3-wöchigen Intervallen VP16/Etoposid über jeweils 3 Tage gegeben. Zusätzlich wird geprüft, wie sich nach insgesamt 6-monatiger Behandlungszeit der Typ der Induktionstherapie auf die Reaktion im Tumor (Tumorresponse) auswirkt.
Um diese Analysen durchführen zu können, ist die Randomisation von insgesamt 360 Patienten vorgesehen.

Für die anschließende Erhaltungstherapie (Konsolidierung) wird die Behandlung mit Carboplatin und Vincristin bis zur Gesamtbehandlungszeit von 18 Monaten fortgesetzt. Allerdings werden die Abstände zwischen den Carboplatingaben auf 6 Wochen verlängert und zusätzliche Vincristingaben an Tag 8 und 15 eines jeden 6-Wochen-Zyklus verabreicht. Mit dieser Konsolidierungstherapie soll die nach 6 Monaten erreichte Therapieresponse gehalten werden. Einige Tumoren zeigten in der Vergangenheit auch während der Konsolidierungsphase noch eine Tumorvolumenverminderung.

Als Nebenwirkung während der langen Therapie kann eine Carboplatinallergie auftreten. In einem derartigen Fall wird der Wechsel auf andere Medikamentenkombinationen empfohlen (Vincristin / Cyclophosphamid und Vincristin / Cisplatin), die im Wechsel bei gleichem Rhythmus gegeben werden sollen. Ziel ist es, die Gesamtbehandlungszeit von 18 Monaten zu erreichen.

<u>2. Niedrigmaligne Gliome in anderen Tumorlokalisationen bei Kindern ohne Neurofibromatose NF I.</u>

Bei Kindern mit Tumoren im Bereich der Großhirnrinde oder des Kleinhirns kann in einem großen Teil der Fälle eine vollständige Tumorresektion erreicht werden. Daher ist nur in Ausnahmefällen eine zusätzliche, nicht-chirurgische Behandlung erforderlich. Bei kleinen Kindern sollte eine Chemotherapie eingesetzt werden. Sie folgt den Empfehlungen für Kinder mit Tumoren des Zwischenhirnes und wird ebenfalls in der Induktionstherapiephase randomisiert.

In beiden Therapiegruppen sollten ältere Kinder mit einer Strahlentherapie behandelt werden, die Altersgrenze wird aufgrund von Vorerfahrungen bei 8 Jahren festgelegt. Kinder mit Tumoren, die sich für eine interstitielle Radiotherapie eignen können, können unabhängig vom Alter mit Brachytherapie behandelt werden.

<u>3. Niedriggradige Gliome aller Lokalisationen bei Kindern mit Neurofibromatose Typ NF I.</u>

Es gibt Hinweise, dass sich niedriggradige Gliome bei Kindern mit Neurofibromatose biologisch anders verhalten als bei Kindern ohne Neurofibromatose. Die Neurofibromatose ist eine genetische Erkrankung, zu deren Diagnose klinische Merkmale herangezogen werden. Genetisch handelt es sich um Veränderungen im sogenannten Neurofibromingen, das auf dem langen Arm von Chromosom 17 lokalisiert ist. Bislang konnten typische Veränderungen des NF-Gens jedoch nicht mit klinischen Verlaufsformen korreliert werden, so dass bislang die genetische Untersuchung keinen Einfluss auf die evtl. Notwendigkeit einer Chemo- oder Radiotherapie hatte.
Es ist bekannt, dass die Strahlentherapie bei Patienten mit Neurofibromatose zu verstärkten Nebenwirkungen führt. Da zudem bei einem Teil der Patienten Einschränkungen der psychointellektuellen Leistungsfähigkeit Bestandteil der Grunderkrankung sind, soll besonders für die häufigen Tumoren des Sehbahnsystems, aber auch bei allen anderen Tumoren unabhängig vom Alter der Kinder zunächst auf eine Strahlentherapie verzichtet werden. Da Patienten mit Neurofibromatose besonders gut auf die Standardchemotherapie anzusprechen scheinen und andererseits das Risiko von Zweittumorentstehungen nach bestimmten Chemotherapien erhöht zu sein scheint, ist eine Teilnahme der Kinder an der randomisierten Prüfung der Intensivierung der Induktionstherapie nicht vorgesehen. Kinder mit Neurofibromatose erhalten wie die anderen Gruppen eine verlängerte Gesamttherapie von 18 Monaten. Sie sollten erst bestrahlt werden, wenn Chemotherapieoptionen oder eine Tumorresektion versagt haben.

Langzeitbeobachtung

Da auch bei Kindern deren Tumoren vollständig entfernt werden konnten in einem geringen Prozentsatz der Fälle noch nach vielen Jahren Rückfälle auftreten können, bedürfen sie ebenso wie Kinder, deren Tumoren nicht oder nur teilweise operativ entfernt werden konnten und die eventuell zusätzlich eine nicht-chirurgische Therapie erhielten, einer langjährigen Nachbeobachtung, teilweise über die Kindheit und Jugend hinaus bis ins Erwachsenenalter. Während dieser Langzeitbeobachtung müssen in regelmäßigen Abständen klinische und bildgebende Kontrolluntersuchungen durchgeführt werden. Zusätzliche Diagnostik (Hörtests, Sehtests, Hormonuntersuchungen etc.) sind in Abhängigkeit von Tumorsitz und durchgeführter Therapie auch unabhängig von dieser Therapiestudie erforderlich.

Es ist das Ziel der Behandlungsstrategie bei niedrig-malignen Gliomen das Ausmaß therapieinduzierter Folgeschäden möglichst gering zu halten. Allerdings gibt es bislang kaum Studien, die die Frage beantworten, wie der Gesundheitsstatus und die Lebensqualität überlebender Kinder nach Jahren zu bewerten ist. Daher ist für die Patienten dieser Therapieoptimierungsstudie die langfristige Erfassung von Parametern der Funktion, des Verhaltens und der Lebensqualität vorgesehen. Diese Erkenntnisse werden geeignet sein, im Einzelfall rechtzeitig Förder- und Unterstützungsmaßnahmen einzuleiten. Sie werden aber auch in die Konzeption nachfolgender Therapiestudien einfließen.

Teilnahme an der Studie

Ihre Teilnahme an der Studie ist natürlich freiwillig. Sollten Sie sich entschließen, nicht teilzunehmen, wird dieser Entschluss keine Nachteile für Sie / Ihr Kind haben. Sollten Sie einwilligen, können Sie trotzdem jederzeit die Teilnahme zurückziehen, ohne eine Erklärung abgeben zu müssen.

Als bewährte Therapiealternative steht die Behandlung gemäß dem Protokoll SIOP/GPOH HIT-LGG 1996 zur Verfügung, dessen Ergebnisse die Grundlage des dargelegten Behandlungskonzeptes sind.

Nach dem ausführlichen Aufklärungsgespräch stehen Dir/Ihnen die behandelnden Ärzte Deines/Ihres Therapiezentrums für weitere Fragen zur Verfügung. Diese Patienteninformation wird Dir/Ihnen ebenso wie eine Kopie der unterschriebenen Einverständniserklärungen zum Verbleib ausgehändigt.

Die Ethikkommissionen der Ludwig-Maximilians-Universität München und des Klinikums Augsburg haben die Behandlungsstudie SIOP-LGG-2004 begutachtet und ihr positives Votum abgegeben.

Bei Patientinnen im gebärfähigen Alter ist eine Empfängnisverhütung notwendig, da die Bestrahlung und die Chemotherapie zur Schädigung des neuentstehenden Lebewesens führen können.

Für die Patienten des randomisierten Studienarms besteht über einen Gruppenvertrag der Deutschen Krebsgesellschaft e.V. eine Haftpflichtversicherung für Personenschäden aus der durchgeführten Studie. Versicherer ist die Gothaer Versicherung, Niederlassung Köln (Haftpflichtversicherung Nr: 37.907.585352).

Die gesamte Studie oder einzelne Studienarme können geschlossen werden, wenn sich z. B. eine deutliche Überlegenheit eines Therapie-/Randomisierungszweiges zeigt oder wenn neue Erkenntnisse aus internationalen Studien ein anderes Vorgehen nahelegen. Die teilnehmenden Kliniken und Studienpatienten werden in adäquater Form darüber informiert werden.

Verantwortlicher Arzt:

Name:... Funktion:......................................

Klinik:..

Erreichbar (Telefon):...

......................................, den .... (Datum)

....
Sorgeberechtigte Mutter Sorgeberechtigter Vater

....
Patient/in zuständiger Arzt / Ärztin

22.4.1. Einwilligungserklärung zur Teilnahme an der Behandlungsstudie SIOP-LGG 2004

durch Unterschrift durch den Patienten bzw. Sorge-berechtigten nach dem Aufklärungsgespräch Seite 1/2

Patient/in: ____ ____________________
Name, Vorname Geburtsdatum

Aufklärung am: _____________ durch____
Datum Name, Dienstbezeichnung

Ich/Wir bestätige/n durch meine/unsere Unterschrift, dass ich/wir heute durch den unten genannten Arzt ausführlich über die bei mir / meinem Kind vorliegende Erkrankung, deren Heilungsaussichten, das Ergebnis der Operation und die vorgesehene Behandlung aufgeklärt worden bin. Die Patienten-/Elterninformation als Grundlage für das Aufklärungsgespräch wurde mir/uns schriftlich ausgehändigt und ausführlich mündlich erläutert. Ich hatte Gelegenheit, alle Fragen zu stellen und ausreichende Bedenkzeit vor meiner/unserer Einwilligungserklärung. Ich/Wir fühle/n mich/uns genügend informiert und habe/n keine weiteren Fragen.

Ich/Wir erkläre/n mich/uns damit einverstanden, dass die Behandlung entsprechend den Richtlinien der Therapiestudie SIOP-LGG 2004 durchgeführt wird. Ich/Wir kann/können diese Einverständniserklärung jederzeit formlos widerrufen.

Betrifft Patienten ohne Neurofibromatose, die eine Chemotherapie erhalten:

Einverständnis zur Randomisierung der Chemotherapie

❒ Ich bin/Wir sind mit der Randomisierung der Chemotherapie einverstanden.

❒ Ich bin mit der Randomisierung der Chemotherapie nicht einverstanden. Die Behandlung erfolgt nach dem Standard-Chemotherapiearm.

(Das Zutreffende bitte ankreuzen, das Unzutreffende bitte durchstreichen).

Die Einwilligung zur Weitergabe und Verarbeitung von Patientendaten und Untersuchungsmaterial sowie die Einverständniserklärung für eine Bestrahlung und/oder eine weitere Operation erfolgen gesondert.

....
Ort

....
Patient/in* Name, Vorname Datum Unterschrift
(*zwingend bei mind. 16-jährigen oder vorhandener Einsichtsfähigkeit jüngerer Patienten)

....
Gesprächsführende(r) Arzt/Ärztin Datum Unterschrift
Name, Vorname

22.4.1. Einwilligungserklärung zur Teilnahme an der Behandlungsstudie SIOP-LGG 2004

durch Unterschrift durch den Patienten bzw. Sorgeberechtigten nach dem Aufklärungsgespräch Seite 2/2

.

....
Zeuge/Zeugin Name, Vorname Datum Unterschrift

<u>Einwilligung der gesetzlichen Vertreter</u>
Vorstehende Aufklärung über die vorgesehene Behandlung bei ....
habe(n) ich/wir als gesetzliche(r) Vertreter des Patienten an dessen Stelle erhalten.

( ) Anwesenheit beider Elternteile
Wir versichern, sorgeberechtigt zu sein und erteilen unsere Einwilligung.

....
Name, Vorname Datum Unterschrift

....
Name, Vorname Datum Unterschrift

( ) Anwesenheit nur eines Elternteils
Ich versichere, dass ich allein sorgeberechtigt bin bzw. mit Zustimmung meiner(es)
.... die Einwilligung erteile.

....
Name, Vorname Datum Unterschrift

( ) Vormund/Pfleger
Ich versichere als .... eingesetzt zu sein und erteile meine Einwilligung.

....
Name, Vorname Datum Unterschrift

22.4.2. Einwilligungserklärung zur Weitergabe und Verarbeitung von Patientendaten und Untersuchungsmaterial SIOP-LGG 2004 1/2

Ich/Wir erkläre/n mich/uns damit einverstanden, dass meine personenbezogenen Daten (Name, Geburtsdatum, Wohnort, Diagnose mit Befunderhebung und andere medizinische Daten) bzw. die personenbezogenen Daten meines Sohnes / meiner Tochter

____ ___________________

Name, Vorname Geburtsdatum

verarbeitet werden (Speicherung und Übermittlung).

Das Verarbeiten der Daten dient der medizinischen Dokumentation im Rahmen der Zusammenarbeit mehrerer Kliniken. Eine enge und rasche Zusammenarbeit der Kliniken, Studienleitungen und Institute untereinander soll dadurch gewährleistet werden. Diese Zusammenarbeit dient auch der Qualitätssicherung der Diagnostik und Therapie. Eine solche Dokumentation ist daher als ein wichtiges Hilfsmittel einer zeitgemäßen Behandlung anzusehen. Die Daten werden hierzu an folgende Zentren regelmäßig übermittelt:

- Klinikum Augsburg (Dr. med. A. K. Gnekow), Stenglinstr. 2, 86156 Augsburg (Studienleitung der Hirntumorstudie SIOP-LGG-2004)
- Deutsches Kinderkrebsregister am IMBEI, Universität Mainz (Pädiatrische Onkologie: Dr. P. Kaatsch)
- Institut für Medizinische Biometrie, Epidemiologie und Informatik, Universität Mainz (Biometrie, Dr. rer.nat. A. Faldum)
- Hirntumorreferenzzentrum (Prof. Dr. med. T. Pietsch), Institut für Neuropathologie, Universitäts-Kliniken Bonn, Sigmund-Freud-Str. 25, 53105 Bonn
- Abteilung für Strahlentherapie der Universität Leipzig (Prof. Dr. med. R.D. Kortmann), Härtelstr. 16, 04107 Leipzig
- Abteilung für Neuroradiologie der Universität Würzburg (Frau PD Dr. Monika Warmuth-Metz), Josef-Schneider-Str. 11, 97080 Würzburg

gegebenenfalls auch an:

- HIT-GBM-Studienleitung, Kinderklinik St. Hedwig, Regensburg
- HIT-2000-Studienleitung, Universitäts-Kinderklinik Würzburg
- HIT-REZ-Studienleitung, Universitätskinderklinik Bonn
- HIT-ENDO-Studienleitung, Kinderklinik Oldenburg
- LESS Spätfolgenerfassungsstudie, Universitäts-Kinderklinik Erlangen
- Strahlentherapie: Spätfolgenerfassungsstudie, Universitätsstrahlenklinik Münster
- Lebensqualitätsstudie, Universitäts-Kinderklinik Düsseldorf
- das zuständige Landeskrebsregister____________________________________

22.4.2. Einwilligungserklärung zur Weitergabe und Verarbeitung von Patientendaten und Untersuchungsmaterial SIOP-LGG 2004 2/2

Die in Deutschland durchgeführte LGG-2004-Therapiestudie ist eingebunden in die Studie der internationalen Gesellschaft für pädiatrische Onkologie. Die Daten werden gemeinsam ausgewertet im internationalen Datenmanagementzentrum:

Dr. Gian Luca De Salvo
SIOP-LGG 2004 International Data Centre
Clinical Trials & Biostatistic Unit
Istituto Oncologico Veneto
Busonera Hospital
Via Gattemelata 64
I-35128 Padova, Italy

Die Auswertungen erfolgen unter voller Wahrung der ärztlichen Schweigepflicht und des Datenschutzes. Dein/Ihr Einverständnis zu der Datenverarbeitung ist freiwillig. Für den Fall, dass Du/Sie Deine/Ihre Mitwirkung versagen, entsteht Dir/Ihnen bzw. Ihrem Kind hieraus kein Nachteil. Du kannst/Sie können Dein/Ihr Einverständnis jederzeit widerrufen.

Hiermit erteile ich als Patient(in)/gesetzliche(r) Vertreter (s. Einwilligung zur Studienteilnahme) meine Einwilligung zur Weitergabe und Verarbeitung von Patientendaten und Untersuchungsmaterial.

____
Ort

Patient/in: Name, Vorname	Unterschrift Datum
Sorgeberechtigte/Mutter: Name, Vorname	Unterschrift Datum
Sorgeberechtigter/Vater: Name, Vorname	Unterschrift Datum
Aufklärende/r Ärztin/Arzt: Name, Vorname	Unterschrift Datum
Zeugin / Zeuge: Name, Vorname	Unterschrift Datum

22.4.3. Einwilligungserklärung zur Asservierung von Tumorgewebe SIOP -LGG 2004

Seite 1/1

„Ich bin damit einverstanden, dass Tumorgewebe meines Kindes zur Erforschung der Krankheit in ihren molekularen, genetischen, immunologischen und anderen, mit der Krankheit direkt verbundenen Merkmalen untersucht und gegebenenfalls für die Entwicklung neuer Behandlungsverfahren eingesetzt wird. Die Entnahme des Tumorgewebes erfolgt schmerzlos im Rahmen der für mein Kind notwendigen chirurgischen Tumorentfernung bzw. während der zur Diagnosestellung erforderlichen Probeentnahme aus dem Tumor.
Falls bei der Tumorentfernung aus medizinisch chirurgischen Notwendigkeiten gesundes Gewebe mitentfernt werden muss, darf dieses als Vergleichsgewebe für die Tumoreigenschaften eingesetzt werden. Eine medizinisch nicht notwendige Erweiterung des chirurgischen Eingriffes erfolgt dazu nicht.
Zugestimmt wird der Entnahme einer Blutprobe während der Narkose (je nach Alter 2-10 ml) als Vergleichsmaterial für die Eigenschaften des Tumors. Tumor, Vergleichsgewebe und Vergleichsblut werden zentral in einer Tumorbank der GPOH gelagert und kostenfrei und anonymisiert Wissenschaftlern, die in universitären Einrichtungen oder in Krankenhäusern tätig und in GPOH-Studien kooperativ eingebunden sind, aber auch unabhängigen Wissenschaftlern, für die obengenannten krankheitsbezogenen Untersuchungen zur Verfügung gestellt. Ein unabhängiger Aufsichtsrat entscheidet über eingereichte Anträge und die entsprechende Materialvergabe.

Auf diese Weise sollen die Diagnosestellung sicherer gemacht werden, das biologische Verständnis der Erkrankung verbessert und neue therapeutische Ansätze gefunden werden."

Im Rahmen der SIOP-LGG-2004-Studie erfolgt die zentrale Asservierung von Tumormaterial in der Bundesrepublik in der Tumorbank für Hirntumoren, kommissarisch: Prof. Dr. Pietsch, Institut für Neuropathologie, Universitätskliniken Bonn, Sigmund-Freud-Str. 25, 53105 Bonn.

Hiermit erteile ich als Patient(in)/gesetzliche(r) Vertreter (s. Einwilligung zur Studienteilnahme) meine Einwilligung zurAsservierung von restlichem Material ❐

Ich bin mit der Asservierung von restlichem Material nicht einverstanden.
Das Material soll vernichtet werden. ❐

	____________________ Ort
____________________ Patient/in Name, Vorname	____________________ Unterschrift Datum
____________________ Sorgeberechtigte/Mutter Name, Vorname	____________________ Unterschrift Datum
____________________ Sorgeberechtigte/Vater Name, Vorname	____________________ Unterschrift Datum
____________________ Aufklärende/r Ärztin/Arzt Name, Vorname	____________________ Unterschrift Datum
____________________ Zeugin / Zeuge, Name, Vorname	____________________ Unterschrift Datum

22.5.1. Tumoren im Kindesalter – Kooperative Dokumentation

In Zusammenarbeit mit dem Deutschen Kinderkrebsregister am IMBEI, 55101 Mainz,
Tel. 06131/17-3227, Fax 06131/17-4462

SIOP-LGG 2004 - Ersterhebung - 1/3

Studienleitung: **Frau Dr. Astrid K. Gnekow**, I. Klinik f. Kinder u. Jugendliche, Klinikum Augsburg, Stenglinstr. 2, 86156 Augsburg, Tel.: 0049 - (0) 821 - 400 - 3615, Fax: -3616, Email: gnekow.hit-lgg@klinikum-augsburg.de

Name, Vorname Pat.-Nr. Klinik Pat.-Identifikationszahl

I______________________________I I_I_I_I_I I_I_I_I_I I__I I__I__I__I__I__I__I I__I__I__I__I__I__I

Geschlecht 1= m,2= w Geb. Datum

GPOH-PID: I__I__I__I__I__I__I__I__I__I

! Bitte beachten Sie, dass vor der Weiterleitung dieses Bogens die schriftliche Einwilligung ! zur Übermittlung der Daten und zur dortigen Speicherung vorliegen muss!

Teilnahme an der Studie: ❐ (vorläufig) in der Beobachtungsgruppe ❐ in der Therapiegruppe ❐ keine Teilnahme

Heriditäre Grunderkrankungen:

❐ Neurofibromatose Typ NFI ❐ nein ❐ ja: ❐ familiär ❐ sporadisch
❐ Merkmale vorhanden, aber internationale Kriterien nicht erfüllt

❐ tuberöse Hirnsklerose ❐ nein ❐ ja

❐ andere:______________________

Anlass der Erfassung: ❐ Tumorsymptomatik führte zum Arztbesuch (siehe unten)
❐ Vorsorgeuntersuchung (U1-U9)
❐ Befund bei anderweitiger Untersuchung

Frühestes Auftreten von eindeutig auf den Tumor zu beziehenden Symptomen:

vor I__I__I Wochen / I__I__I Monaten: (Bitte kreuzen Sie das entsprechende Feld an)

Hirndrucksymptomatik:	❐ nein	❐ ja______________________	
Neurologische Symptomatik:	❐ nein	❐ ja	➜ Formblatt „Neurologischer Befund" ergänzen
Sehstörungen:	❐ nein	❐ ja	➜ Formblatt „Ophthalmologischer Befund" ergänzen
Endokrinol. Symptomatik:	❐ nein	❐ ja	➜ Formblatt „Endokrinologischer Befund" ergänzen
Diencephales Syndrom:	❐ nein	❐ ja	______________________
Sonstige:	❐ nein	❐ ja	______________________

Andere Symptome bei Diagnosestellung (bitte ausführen): ______________________

____

____

Neuro-Radiologie bei Diagnosestellung (präoperativ) – Lokale Befundung

Datum der Diagnosestellung anhand bildgebender Verfahren: I__I__I . I__I__I . I__I__I__I__I

❐ **MRT** ❐ mit KM ❐ ohne KM

❐ **CT** ❐ mit KM ❐ ohne KM **Tumorgröße:** I__I__I cm x I__I__I cm x I__I__I cm

Zystischer Anteil: ❐ nein ❐ ja **Zystengröße:** I__I__I cm x I__I__I cm x I__I__I cm

Ventrikelerweiterung: ❐ nein ❐ ja

Raumforderung: ❐ nein ❐ ja: ❐ lokal
❐ Mittellinienverlagerung

Histologie (2/3)

❒ **Keine Histologie**

❒ **Datum d. Diagnosestellung anhand histologischer Sicherung:** I__I__I . I__I__I . I__I__I __I__I

Neuropathologie (Ort): ______________________________ **E-Nr.** ____________________________________

Referenzpathologie Bonn ❒ **Nein** ❒ **Ja,** **R-Nr.**_____________________________________

Diskrepante Diagnose ❒ **Nein** ❒ **Ja, Histologie**

Bonn:____

Histopathologische Klassifikation und Grading nach WHO (Kleihus u. Cavanee, 2000) – Lokale Befundung:

1. ❒ Astrozytische Tumoren
1.1. ❒ Pilozytisches Astrozytom
1.1.1. ❒ pilomyxoide Variante
1.2. ❒ Astrozytom ohne nähere Angaben
1.2.1. ❒ Fibrilläres Astrozytom
1.2.2. ❒ Protoplasmatisches Astrocytom
1.2.3. ❒ Gemistozytisches Astrozytom
1.3. ❒ Pleomorphes Xanthoastrozytom
1.4. ❒ Subependymales großzelliges Astrozytom

2. ❒ Oligodendrogliale Tumoren
2.1. ❒ Oligodendrogliom ohne nähere Angabe

3. ❒ Gemischte Gliome
3.1. ❒ Oligo-Astrozytom
3.2. ❒ Andere gemischte Gliome

4. ❒ Neuronale u. gemischt neuronale-gliale Tumoren
4.1. ❒DIGG/DIA - desmoplast., infantiles Gangliogliom/-Astrozytom
4.2. ❒ DNT - dysembryoplastischer, neuroepithelialer Tumor
4.3. ❒ Gangliogliom
4.4. ❒ atyp., myxomat., neuroepith. Tumor

5. ❒ Nicht-Studien-Diagnosen:
❒ Pineocytom
❒ Plexuspapillom
❒ Neurinom
❒ Akustikusneurinom (❒ NF II)
❒ Gangliozytom
❒ andere:_________________________

➡ **Malignitätsgrad nach WHO-Klassifikation**: ❒ **°I** ❒ **°II** ❒ **°III** ❒ **°IV**

LOKALISATION

(Hauptlokalisation bitte unterstreichen, alle involvierten Strukturen markieren)

1. ❒ Cerebrale Hemisphären
1.1. ❒ Frontallappen
1.2. ❒ Parietallappen
1.3. ❒ Temporallappen
1.4. ❒ Occipitallappen

2. ❒ Supratentorielle Mittellinie
2.1. ❒ Vorderer Abschnitt des N. opticus (einschl. Orbita)
2.2. ❒ Chiasma opticum
2.3. ❒ Diencephalon
2.3.1 ❒ Hypothalamus
2.3.2. ❒ III. Ventrikel
2.3.3 ❒ Thalamus
2.3.4 ❒ Basalganglien
2.3.5. ❒ Balken
2.3.6. ❒ Hypophyse
2.3.7. ❒ Limb. System/Fornix
2.4. ❒ Mesencephalon
2.4.1. ❒ Crus cerebri
2.4.2 ❒ Tegmentum
2.4.3. ❒ Tectum/Lamina quadrigemina
2.4.4. ❒ Pinealisregion

3. ❒ Cerebellum
3.1. ❒ Wurm
3.2. ❒ Brückenwinkel
3.3. ❒ Hemisphären

4. ❒ caudaler Hirnstamm
4.1. ❒ IV. Ventrikel
4.2. ❒ Pons fokal
4.3. ❒ Pons intrinsisch
4.4. ❒ Medulla oblongata
4.5. ❒ cranio-spinaler Übergang

5. ❒ Spinal
5.1. ❒ intraspinal, extradural
5.2. ❒ subdural, extramedullär
5.3. ❒ intramedullär

ad 5.1.-5.3.:
Segmente:_________________

6. ❒ Seitenventrikel
(Riesenzellastrozytome) bei tuberöser Sklerose)

<u>Ergänzungen zur Lokalisation:</u>
____
____
____

Dodge-Klassifizierung der Sehbahngliome:
❒ **I (nur N. opticus)** ❒ **II (Chiasma ± N. opticus)** ❒ **III (Chiasma + dienceph. Extension)**

Seite der Hauptlokalisation: ❒ rechts ❒ links ❒ beidseits ❒ Mitte

Primäre Metastasen: ❒ Nein ❒ Ja, wo:____

❒ M 1 ❒ M 2 ❒ M 3 ❒ M 4

Primär chirurgisches Vorgehen (3/3)

Shuntanlage vor/nach Operation: ❐ Nein ❐ Ja, am: I__I__I . I__I__I . I__I__I__I__I

Art des Shunts:____

Datum der Operation: I__I__I . I__I__I . I__I__I__I__I **Ort, Operateur:** ________________________

____

Ausmaß der Operation:

❐ S1 totale Resektion (kein erkennbarer Resttumor)
❐ S2 subtotale Resektion (Resttumor < 1,5 cm ³ , evtl. örtliche Invasion)
❐ S3 partielle Resektion (Resttumor > 1,5 cm³)
❐ S4 Biopsie ❐ offen ❐ stereotaktisch ❐ endoskopisch

Neuro-Radiologie frühpostoperativ (innerhalb von 72 Stunden)

Datum I__I__I . I__I__I . I__I__I__I__I **Verfahren:** ❐ MRT ❐ CT
mit KM: ❐ Nein ❐ Ja

Größe des Resttumors: I__I__I cm x I__I__I cm x I__I__I cm

Befund: ❐ R1 kein Hinweis auf Resttumor
❐ R2 Kontrastmittelanreicherung vorhanden, aber nicht ausmessbar
❐ R3 Resttumor einer ausmessbaren Größe
❐ R4 keine erkennbare Veränderung im Vergleich zur präoperativen Größe (minimale Veränderungen)

Definitives Operationsergebnis: (SIOP-Klassifikation 1995)

		Radiologie	Chirurgie
❐	Totale Resektion	R1	S1
❐	subtotal	R1 / R2	S2
❐	partiell	R3	S1/ S2 / S3
❐	Biopsie	R4	S4

Vollremission erreicht? ❐ Ja ❐ Nein

Postoperatives Vorgehen

❐ **Beobachtung (wait and see)**
❐ **Therapie, welche:** ❐ **Chemotherapie** ❐ **Strahlentherapie** ❐ **Sonstige** ________________

Bitte umgehend Doku-Bogen "Therapie-Basisinformation" an Studienleitung schicken!

Postoperativer Therapiebeginn: I__I__I . I__I__I . I__I__I__I__I

Letztes follow-up-Datum: I__I__I . I__I__I . I__I__I__I__I ❐ Patient lebt ❐ Patient verstorben

Bitte unbedingt mitschicken:
❐ prae- und postoperative MRT-/CT-Befunde ❐ örtliche Histologie und Referenzhistologie
❐ Op-Bericht/e

Bemerkung:

________________________ ____________ ________________________
Name des dokumentierenden Arztes (Stempel) Datum Unterschrift

22.5.2. Tumoren im Kindesalter – Kooperative Dokumentation

In Zusammenarbeit mit dem Deutschen Kinderkrebsregister am IMBEI, 55101 Mainz,
Tel. 06131/17-3227, Fax 06131/17-4462

SIOP-LGG 2004 - Therapie – Basisinformationen - 1/1

Studienleitung: **Frau Dr. Astrid K. Gnekow**, I. Klinik f. Kinder u. Jugendliche, Klinikum Augsburg, Stenglinstr. 2, 86156 Augsburg, Tel.: 0049 - (0) 821 - 400 - 3615, Fax: -3616, Email: gnekow.hit-lgg@klinikum-augsburg.de

Name, Vorname Pat.-Nr. Klinik Pat.-Identifikationszahl
I________________________________I I_I_I_I_I I_I_I_I_I I__I I__I__I__I__I__I__I I__I__I__I__I__I
GPOH-PID: I__I__I__I__I__I__I__I__I__I Geb. Datum

❒ **Therapieindikation bei Diagnosestellung nach klin. Diagnosestellung oder Biopsie**
❒ **Therapie bei Diagnosestellung nach partieller oder subtotaler Resektion** (Bitte ankreuzen)

- Diencephales Syndrom	❒ Nein	❒ Ja
- Fokale neurologische Ausfälle infolge von Tumorwachstum	❒ Nein	❒ Ja
- Krampfanfälle infolge von Tumorwachstum	❒ Nein	❒ Ja
- Hirndruckzeichen infolge von Tumorwachstum	❒ Nein	❒ Ja
- Definitive anamnestische Sehverschlechterung	❒ Nein	❒ Ja
- Grenzwertiger Visus ("Threat to vision")	❒ Nein	❒ Ja
- Nystagmus infolge Visusverlust bei Säuglingen und Kleinkindern	❒ Nein	❒ Ja
- Symptomatische Metastasen	❒ Nein	❒ Ja

- Radiologische Befunde: Der alleinige radiologische Resttumornachweis stellt keine sofortige Therapieindikation dar.

❒ **Therapieindikation nach Beobachtungsphase**

- Definitive anamnestische Sehverschlechterung	❒ Nein	❒ Ja
- Auftreten neuer neurologischer Symptome	❒ Nein	❒ Ja
- Neuauftreten eines diencephalen Syndroms	❒ Nein	❒ Ja
- Verschlechterung eines nutzbaren Visus/Gesichtsfeldes	❒ Nein	❒ Ja
- Jede Visusreduktion bei Zustand nach Erblindung des anderen Auges	❒ Nein	❒ Ja
- Zunahme d. Tumorvolumens um > 25 % (bzw. d. Durchmessers d. Sehnerven)	❒ Nein	❒ Ja
- Übergreifen auf zuvor nicht-involvierte Hirnareale	❒ Nein	❒ Ja
- Auftreten neuer Läsionen (+/- symptomatische/progrediente Metastasen)	❒ Nein	❒ Ja

Letzte Bildgebung vor Therapiebeginn: I__I__I . I__I__I . I__I__I__I__I (ttmmjjjj)

Tumorgröße: I__I__I , I__I cm x I__I__I , I__I cm x I__I__I , I__I cm

MRT-Bilder zur zentralen Beurteilung versandt? ❒ Ja ❒ Nein ❒ Veranlasst

Beginn der Therapie: I__I__I . I__I__I . I__I__I__I__I (ttmmjjjj)

Patient erhält: ❒ **Chemotherapie (gem. vorheriger Randomisierung)** ❒ **Radiotherapie**

❒ Induktion I + Konsolidierung
❒ Induktion II + Konsolidierung

❒ konventionelle Strahlentherapie
❒ 125-Jod-Seed-Implantation
❒ andere: ______________________
____

In welcher Klinik erfolgt die Therapie? ____

_______________________ _________ ______________________________
Name des dokumentierenden Arztes (Stempel) Datum Unterschrift

22.6.1. Zentrale Randomisierung

SIOP LGG 2004
Seite 1/1

Studienleitung: **Frau Dr. Astrid K. Gnekow**, I. Klinik f. Kinder u. Jugendliche, Klinikum Augsburg, Stenglinstr. 2, 86156 Augsburg, Tel.: 0049 - (0) 821 - 400 - 3615, Fax: -3616 , Email: gnekow.hit-lgg@klinikum-augsburg.de

Randomisierung der Induktionstherapie

Pat.-Identifikations-Nummer I__I I__I__I__I__I__I__I I__I__I__I__I__I

GPOH-PID: I__I__I__I__I__I__I__I__I

Behandelnde Klinik / Ort: ______________________________

Patient (Nach- und Vorname): ______________________________

Geburtsdatum: I__I__I . I__I__I . I__I__I__I__I

Neurofibromatose Typ NF I ❐ **Nein** ❐ nicht geklärt

Alter des Patienten: ❐ < 1 Jahr ❐ 1- 8 Jahre ❐ ≥ 8 Jahre

Lokalisation:

❐ **Cerebrale Hemisphären**
❐ **Cerebellum**
❐ **kaudaler Hirnstamm**
❐ **Spinal**
❐ **Seitenventrikel**

❐ **Supratentorielle Mittellinie**
Extension bei Sehbahngliomen:
❐ Dodge I (nur N. opticus) ➪ keine Randomisierung
❐ Dodge II (Chiasma + N. opticus)
❐ Dodge III (Chiasma + Extensionen)

Diagnosedatum: ❐ klinisch ❐ histologisch I__I__I . I__I__I . I__I__I__I__I

Operationsdatum vor Beginn der Chemotherapie: I__I__I . I__I__I . I__I__I__I__I

Vorausgegangene Chemo- oder Radiotherapie: ❐ Nein ❐ Ja ➪ keine Randomisierung

Histopathologische Diagnosesicherung
Material an Ref.zentrum verschickt ❐ nein ❐ ja am: I__I__I . I__I__I . I__I__I__I__I ❐ veranlasst

Histologischer Befund nach erster Operation: ______________________________

Histologischer Befund nach zweiter Operation: ______________________________

WHO-Klassifikation: ❐ °I ❐ ° II

MRT (prä- und früh postoperativ sowie vor Therapiebeginn)
an Studienzentrale verschickt: ❐ nein ❐ ja am: I__I__I . I__I__I . I__I__I__I__I ❐ veranlasst

Therapie bei: ❐ **Diagnosestellung** ❐ **Progredienz (nach Beobachtung)**

Einwilligung zur Randomisierung liegt vor: ❐ **Ja** ❐ **Nein**

Fax-Nr. für Rückantwort: ______________________

______________________ ____________ ______________________
Name des dokumentierenden Arztes (Stempel) Datum Unterschrift

22.6.2. Randomisierungsergebnis (1/1) **SIOP -LGG 2004**

Anschrift der behandelnden Klinik m. Ansprechpartner:

FAX-Nummer der behandelnden Klinik:

Pat.-Identifikations-Nummer I__I I__I__I__I__I__I__I__I I__I__I__I__I__I__I GPOH-PID: |__|__|__|__|__|__|__|__|__|

Name	Vorname	Geb. Datum	Klinik

Randomisierungs-Ergebnis

❒ **Induktion I:** **Carboplatin / Vincristin**

❒ **Induktion II:** **Carboplatin, Vincristin, Etoposid (VP 16)**

Datum:____________________ Unterschrift:____

Vielen Dank für die Meldung Ihres neuen Patienten, der im Rahmen der SIOP-LGG 2004-Studie nach Randomisation mit Chemotherapie behandelt werden soll. **Bitte beachten Sie das Ergebnis der Randomisierung!** Ihre Klinik hatte sich zu Beginn der Studie bereit erklärt, die Therapie entsprechend den Protokollvorgaben durchzuführen. Nur in speziellen Ausnahmefällen darf davon nach Rücksprache mit der Studienleitung abgegangen werden.

Studienleitung:

Frau Dr. Astrid K. Gnekow
I. Klinik f. Kinder und Jugendliche
Klinikum Augsburg
Stenglinstr. 2
86156 Augsburg
Tel.: 0049 - (0)821 - 400 – 3615
FAX: 0049 – (0)821 – 400 – 3616
gnekow.hit-lgg@klinikum-augsburg.de

22.7. Chemotherapie Therapiepläne

SIOP -LGG 2004

Die nachfolgenden Seiten enthalten Empfehlungen für die Durchführung der Chemotherapie im Rahmen dieser Studie. Für die Kombinationstherapie dieses Protokolls können durchaus örtliche, standardisierte Durchführungsrichtlinien vorliegen, an die die folgenden Empfehlungen angepasst werden können. Es liegt in der Verantwortung jedes einzelnen Therapeuten, für die Sicherheit des einzelnen Patienten unter Therapie Sorge zu tragen. Das Therapieprotokoll kann nur eine Orientierungshilfe anbieten.

22.7.1. Induktion

22.7.1.1. Vincristin / Carboplatin
22.7.1.2. Vincristin / Carboplatin / Etoposid

22.7.2. Konsolidierung

22.7.2.1. Vincristin / Carboplatin
22.7.2.2. Vincristin / Cisplatin
22.7.2.3. Vincristin / Cyclophosphamid

22.7.1.1. Induktion I: Vincristin / Carboplatin **SIOP-LGG 2004**

Name:_______________________________________ Geb.datum: I__I__I.I__I__I.I__I__I__I__I

Gewicht:_________________ Größe:_______________ KOF:________ m²

Therapiewoche:________

Woche	1	2	3	4	5	6	7	8	9	10	13	17	21	24
	**V**	**V**	**V**	**V**	**V**	**V**	**V**	**V**	**V**	**V**	**V**	**V**	**V**	
	**C**			**C**			**C**			**C**	**C**	**C**	**C**	
														MRT

I. Spülung: Beginn 3-6 h vor; bis zu 24 h nach Carboplatin:

Glucose 5 % / NaCl 0,9 % 1:1 2000 – 3000 ml / m² / 24 h = ___________ ml

Per 500 ml: + 10 ml K Cl 7,45 % = ___________ ml
+ 6 ml Magnesium 20 % = ___________ ml
+ 10 ml Ca-Gluconat 10 % = ___________ ml

II. Vincristin 1,5 mg / m² iv-Bolus (max. 2 mg) = ___________ mg
Tag 1 – Woche:_________________

III. Carboplatin 550 mg / m² 60 Minuten-Infusion = ___________ mg
in 200 ml Glucose 5 %
Tag 1 – Woche:___________

IV. Supportive Therapie:

- **Mannit 20 % 40 ml / m² als Kurzinfusion = ___________ ml**
  Falls Urinausfuhr auf < 2/3 der Flüssigkeitszufuhr zurückgeht

- **Antiemese:_______________________________________ = ___________ mg**
  Beginn 30' vor Chemotherapie

- **Dexamethason 0,15 mg / kg , iv-Bolus** = ___________ **mg**
  Steroide können die Wirksamkeit von Platin auf gliale Zellen beeinträchtigen. Sie sollten nur bei Notwendigkeit eingesetzt werden.
  Bei erhöhtem Hirndruck vor oder während der Therapie ohne Indikation zur Anlage eines Shunts

Dosismodifikationen auf Grund von Toxizität und Empfehlungen für weitere supportive Therapiemaßnahmen beachten (siehe Abschnitt 14.2.-3.).

22.7.1.2. Induktion II: Vincristin / Carboplatin / Etoposid SIOP -LGG 2004

Name:____________________________________ Geb.datum: I__I__I.I__I__I.I__I__I__I__I

Gewicht:________________ Größe:______________ KOF:________ m²

Therapiewoche:________

Woche	1	2	3	4	5	6	7	8	9	10	13	17	21	24
	V	V	V	V	V	V	V	V	V	V	V	V	V	
	C			C			C			C	C	C	C	
	Ex3			Ex3			Ex3			Ex3				
														MRT

I. Spülung: Beginn 3-6 h vor; bis zu 24 h nach Carboplatin:

Glucose 5 % / NaCl 0,9 % 1:1 2000 – 3000 ml / m² / 24 h = ___________ ml

Per 500 ml: + 10 ml K Cl 7,45 % = ___________ ml
+ 6 ml Magnesium 20 % = ___________ ml
+ 10 ml Ca-Gluconat 10 % = ___________ ml

II. Vincristin 1,5 mg / m² iv-Bolus (max. 2 mg) = ___________ mg
Tag 1 - Woche: ____________

III. Carboplatin 550 mg / m² 60 Minuten-Infusion = ___________ mg
in 200 ml Glucose 5 %
Tag 1 – Woche: ____________

IV. Etoposid 100 mg / m² 60 Minuten-Infusion = ___________ mg
in _______ ml/Na Cl 0,9 %
Endkonzentration von ≤ 0,4 mg/ml
Tag 1, 2, 3 – Woche:_________

V. Supportive Therapie:

- **Mannit 20 % 40 ml / m² als Kurzinfusion = ___________ ml**
  Falls Urinausfuhr auf < 2/3 der Flüssigkeitszufuhr zurückgeht
- **Antiemese:___________________________________ = ___________ mg**
  Beginn 30' vor Chemotherapie
- **Dexamethason 0,15 mg / kg , iv-Bolus** = ___________ **mg**
  Steroide können die Wirksamkeit von Platin auf gliale Zellen beeinträchtigen. Sie sollten nur bei Notwendigkeit eingesetzt werden.
  Bei erhöhtem Hirndruck vor oder während der Therapie ohne Indikation zur Anlage eines Shunts.

Dosismodifikationen auf Grund von Toxizität und Empfehlungen für weitere supportive Therapiemaßnahmen beachten (siehe Abschnitt 14.2.-3.).

22.7.2.1. Konsolidierung: Vincristin / Carboplatin **SIOP -LGG 2004**

Name:______________________________________ Geb.datum: I__I__I.I__I__I.I__I__I__I__I

Gewicht:_________________ Größe:______________ KOF:________ m²

Therapiewoche:________

Woche:	Tag 1	Tag 8	Tag 15
	VCR<br>Carboplatin	VCR	VCR

I. Spülung: Beginn 3-6 h vor; bis zu 24 h nach Carboplatin:

Glucose 5 % / NaCl 0,9 % 1:1 2000 – 3000 ml / m² / 24 h = ___________ ml

Per 500 ml: + 10 ml K Cl 7,45 % = ___________ ml
+ 6 ml Magnesium 20 % = ___________ ml
+ 10 ml Ca-Gluconat 10 % = ___________ ml

II. Vincristin 1,5 mg / m² iv-Bolus (max. 2 mg) = ___________ mg
Tag 1, 8, 15 – Woche:_________________

III. Carboplatin 550 mg / m² 60 Minuten-Infusion = ___________ mg
in 200 ml Glucose 5 %
Tag 1 – Woche:___________

IV. Supportive Therapie:

- **Mannit 20 % 40 ml / m² als Kurzinfusion = ___________ ml**
  Falls Urinausfuhr auf < 2/3 der Flüssigkeitszufuhr zurückgeht
- **Antiemese:_______________________________________ = ___________ mg**
  Beginn 30' vor Chemotherapie
- **Dexamethason 0,15 mg / kg , iv-Bolus = ___________ mg**
  Steroide können die Wirksamkeit von Platin auf gliale Zellen beeinträchtigen. Sie sollten nur bei Notwendigkeit eingesetzt werden.
  Bei erhöhtem Hirndruck vor oder während der Therapie ohne Indikation zur Anlage eines Shunts.

Dosismodifikationen auf Grund von Toxizität und Empfehlungen für weitere supportive Therapiemaßnahmen beachten (siehe Abschnitt 14.2.-3.).

22.7.2.2. Konsolidierung: Vincristin / Cyclophosphamid SIOP -LGG 2004

Name:_______________________________________ Geb.datum: I__I__I.I__I__I.I__I__I__I__I

Gewicht:_________________ Größe:_______________ KOF:________ m²

Therapiewoche:________

Woche:	Tag 1	Tag 8	Tag 15
	VCR<br>Cyclophosphamid	VCR	VCR

I. Spülung: Beginn 3-6 h vor; bis 24 h nach Cyclophosphamid:

Glucose 5 % / NaCl 0,9 % 1:1 2000 – 3000 ml / m² / 24 h = ___________ **ml**

Per 500 ml: + 10 ml K Cl 7,45 % = ___________ **ml**

II. Vincristin 1,5 mg / m² iv-Bolus (max. 2 mg) = ___________ ***mg***
Tag 1, 8, 15 – Woche _____________

III. MESNA 500 mg /m² iv-Bolus = ___________ ***mg***
Vor dem Beginn der Cyclophosphamid Infusion

IV. Cyclophosphamid 1500 mg / m² 60 Minuten-Infusion in 250 ml Na Cl 0,9 % = ___________ **mg**
Tag 1 – Woche __________________

V. MESNA* 500 mg /m² iv-Bolus = ___________ ***mg***
4 und 8 Stunden nach dem Beginn der Cyclophosphamid-Therapie

VI. Supportive Therapie:

- **Furosemid 0,5 – 1 mg /kg als Kurzinfusion** = ___________ **mg**
  Falls die Urinausfuhr unter 2/3 der Flüssigkeitszufuhr abfällt bei regelmäßiger 6-stündlicher Bilanzierung.
- **Antiemese:**___________________________________ = ___________ **mg**
  Beginn 30’ vor Beginn der Chemotherapie.
- **Dexamethason 0,15 mg / kg iv-Bolus** = ___________ **mg**
  Bei erhöhtem Hirndruck vor oder während der Therapie ohne Indikation zur Anlage eines Shunts.

- MESNA kann als fortlaufende Infusion mit 1500 mg / m^2 gegeben werden. Orale Applikation ist eine mögliche Alternative.

Dosismodifikationen auf Grund von Toxizität und Empfehlungen für weitere supportive Therapiemaßnahmen beachten (siehe Abschnitt 14.2.-3.).

22.7.2.3. Konsolidierung: Vincristin / Cisplatin SIOP -LGG 2004

Name:____ Geburtsdatum: I__I__I.I__I__I.I__I__I__I__I

Gewicht:__________________ Größe:_______________ KOF:________ m²

Therapiewoche:________

Woche	Tag 1	Tag 2	Tag 8	Tag 15
	VCR<br>Cisplatin	-<br>Cisplatin	VCR	VCR

I. **Vorspülung:** Beginn 12 h vor Cisplatin:

Glucose 5 % / NaCl 0,9 % 1:1 1000 – 1500 ml / m² / 12 h = ___________ ml

Per 500 ml: + 10 ml K Cl 7,45 % = ___________ ml
+ 6 ml Magnesium 20 % = ___________ ml
+ 10 ml Ca-Gluconat 10 % = ___________ ml

II. Vincristin 1,5 mg / m² iv-Bolus = ___________ ***mg***
Tag 1, 8, 15 – Woche ___________

III. Mannit 20 % 40 ml / m² 15 Minuten Kurzinfusion = ___________ ***ml***
Unmittelbar vor dem Beginn der Cisplatin-Infusion

IV. Cisplatin 30 mg / m² 180 Minuten-Infusion = ___________ **mg**
in 250 ml NaCl 0,9 %
Tag 1 und 2 – Woche ___________

V. **Nachspülung:** bis 24 h nach Cisplatin:

Glucose 5 % / NaCl 0,9 % 1:1 2000 – 3000 ml / m² / 24 h = ___________ ml

Per 500 ml: + 10 ml K Cl 7,45 % = ___________ ml
+ 6 ml Magnesium 20 % = ___________ ml
+ 10 ml Ca-Gluconat 10 % = ___________ ml
+ 30 ml Mannit 20 % = ___________ ml

VI. Supportive Therapie:

- **Mannit 20 % 40 ml / m² als Kurzinfusion** = ___________ **ml**
  Falls Urinausfuhr auf < 2/3 der Flüssigkeitszufuhr zurückgeht
- **Antiemese:**_____________________________________ = ___________ **mg**
  Beginn 30’ vor Chemotherapie
- **Dexamethason 0,15 mg / kg , iv-Bolus** = ___________ mg
  Steroide können die Wirksamkeit von Platin auf gliale Zellen beeinträchtigen. Sie sollten nur bei Notwendigkeit eingesetzt werden.
  Bei erhöhtem Hirndruck vor oder während der Therapie ohne Indikation zur Anlage eines Shunts

Dosismodifikationen auf Grund von Toxizität und Empfehlungen für weitere supportive Therapiemaßnahmen beachten (siehe Abschnitt 14.2.-3.).

22.8. Chemotherapie-Dokumentation SIOP-LGG 2004

Chemotherapiedokumentation

22.8.1. Induktion

22.8.1.1. Vincristin/Carboplatin	-	Woche 1 – 24
22.8.1.2. Vincristin/Carboplatin/Etoposid	-	Woche 1 – 24

22.8.2. Konsolidierung

22.8.2.1. Protokoll-Konsolidierung	–	Woche 25 – 54
22.8.2.2. Protokoll-Konsolidierung	–	Woche 55 – 85
22.8.2.3. Alternativ-Konsolidierung nach Allergie	–	Woche 1 - 30
22.8.2.4. Alternativ-Konsolidierung nach Allergie	–	Woche 31 - 61

22.8.3. Toxizitätsdokumentation

Die Angaben zur Toxizität sollten die maximal erreichte Toxizität während eines Therapiezyklus zusammenfassen. Als „Zyklus" gilt der Abstand zwischen zwei Carboplatin-Pulsen (bzw. Cyclophsophamid oder Cisplatin-Pulsen). Infolge der im Therapieverlauf zunehmenden Zykluslängen umfassen die zeitlichen Abschnitte zur Toxizitätsdokumentation daher während der Induktion 3 bzw. 4 Wochen, während der Konsolidierung 6 Wochen.

22.8.4. Responsebeurteilung

22.8.1.1. Tumoren im Kindesalter – Kooperative Dokumentation

In Zusammenarbeit mit dem Deutschen Kinderkrebsregister am IMBEI, 55101 Mainz,
Tel. 06131/17-3227, Fax 06131/17-4462

SIOP-LGG 2004 - Chemotherapie - Induktion I 1/1

Studienleitung: **Frau Dr. Astrid K. Gnekow**, I. Klinik f. Kinder u. Jugendliche, Klinikum Augsburg, Stenglinstr. 2, 86156 Augsburg, Tel.: 0049 - (0) 821 - 400 - 3615, Fax: -3616 , Email: gnekow.hit-lgg@klinikum-augsburg.de

Name, Vorname I__________________________________I Pat.-Nr. I_I_I_I_I Klinik I_I_I_I_I Pat. Identifikationszahl I__I I__I__I__I__I__I__I I__I__I__I__I__I

GPOH-PID: I__I__I__I__I__I__I__I__I__I Geb. Datum

Zu Beginn der Chemotherapie:

Körpergröße: I__I__I__I cm **Körpergewicht:** I__I__I,I__I kg **Körperoberfläche:** I__I,I__I__I m²

Woche	1	2	3	4	5	6	7	8	9	10	13	17	21	24
**Vincristin 1,5 mg/m²**	**V**	**V**	**V**	**V**	**V**	**V**	**V**	**V**	**V**	**V**	**V**	**V**	**V**	
**Carboplatin 550 mg/m²**	**C**			**C**			**C**			**C**	**C**	**C**	**C**	**MRT**

**Woche**	**Datum**	**Vincristin Dosis (mg)**	**Carboplatin Dosis (mg)**	**Dosismodifikation von: Vincristin**	**Carbo**	**Toxizität:** (Grad 3 oder 4,Art)
**1**	I__I__I__I__I__I__I	I__I,I__I__I	I__I__I__I,I__I	❐	❐	❐
2	I__I__I__I__I__I__I	I__I,I__I__I		❐		❐
3	I__I__I__I__I__I__I	I__I,I__I__I		❐		❐
**4**	I__I__I__I__I__I__I	I__I,I__I__I	I__I__I__I,I__I	❐	❐	❐
5	I__I__I__I__I__I__I	I__I,I__I__I		❐		❐
6	I__I__I__I__I__I__I	I__I,I__I__I		❐		❐
**7**	I__I__I__I__I__I__I	I__I,I__I__I	I__I__I__I,I__I	❐	❐	❐
8	I__I__I__I__I__I__I	I__I,I__I__I		❐		❐
9	I__I__I__I__I__I__I	I__I,I__I__I		❐		❐
**10**	I__I__I__I__I__I__I	I__I,I__I__I	I__I__I__I,I__I	❐	❐	❐
**13**	I__I__I__I__I__I__I	I__I,I__I__I	I__I__I__I,I__I	❐	❐	❐
**17**	I__I__I__I__I__I__I	I__I,I__I__I	I__I__I__I,I__I	❐	❐	❐
**21**	I__I__I__I__I__I__I	I__I,I__I__I	I__I__I__I,I__I	❐	⑨	❐
24	I__I__I__I__I__I__I			**MRT** (Befund bitte an Studienleitung, Bilder an rad. Referenzzentrum)		

Therapieabbruch: ❐ Nein ❐ Ja, Datum I__I__I.I__I__I.I__I__I__I__I
Grund: ❐ Progression ❐ Compliance ❐ Toxizität ❐ Allergie ❐ andere:____________________________

Bitte Toxizitätsbogen für jeden Zyklus und Response-bogen nach MRTausfüllen!

.... Datum

.... Unterschrift/Stempel (des dokumentierenden Arztes)

22.8.1.2. Tumoren im Kindesalter – Kooperative Dokumentation

In Zusammenarbeit mit dem Deutschen Kinderkrebsregister am IMBEI, 55101 Mainz,
Tel. 06131/17-3227, Fax 06131/17-4462

SIOP-LGG 2004 - Chemotherapie - Induktion II 1/1

Studienleitung: **Frau Dr. Astrid K. Gnekow**, I. Klinik f. Kinder u. Jugendliche, Klinikum Augsburg, Stenglinstr. 2, 86156 Augsburg, Tel.: 0049 - (0) 821 - 400 - 3615, Fax: -3616 , Email: gnekow.hit-lgg@klinikum-augsburg.de

Name, Vorname I____I Pat.-Nr. I_I_I_I_I Klinik I_I_I_I_I Pat.-Identifikationszahl I__I I__I__I__I__I__I__I I__I__I__I__I__I

GPOH-PID: I__I__I__I__I__I__I__I__I__I Geb. Datum

Zu Beginn der Chemotherapie:

Körpergröße: I__I__I__I cm **Körpergewicht:** I__I__I,I__I kg **Körperoberfläche:** I__I,I__I__I m²

Woche	1	2	3	4	5	6	7	8	9	10	13	17	21	24
**Vincristin 1,5 mg/m²**	V	V	V	V	V	V	V	V	V	V	V	V	V	
**Carboplatin 550 mg/m²**	C			C			C			C	C	C	C	
**Etoposid 100 mg/m²**	Ex3			Ex3			Ex3			Ex3				
														MRT

Woche	Datum	Vincristin Dosis(mg)	Carboplatin Dosis (mg)	Etoposid Dosis (mg)	Dosismodifikation von: V	C	E	Toxizität: (Grad 3 oder 4, Art)
**1**	I__I__I__I__I__I__I	I__I,I__I__I	I__I__I__I,I__I	Tag 1I__I__I__I,I__I<br>Tag 2I__I__I__I,I__I<br>Tag 3I__I__I__I,I__I	❒	❒	❒	❒
2	I__I__I__I__I__I__I	I__I,I__I__I			❒			❒
3	I__I__I__I__I__I__I	I__I,I__I__I			❒			❒
**4**	I__I__I__I__I__I__I	I__I,I__I__I	I__I__I__I,I__I	Tag 1I__I__I__I,I__I<br>Tag 2I__I__I__I,I__I<br>Tag 3I__I__I__I,I__I	❒	❒	❒	❒
5	I__I__I__I__I__I__I	I__I,I__I__I			❒			❒
6	I__I__I__I__I__I__I	I__I,I__I__I			❒			❒
**7**	I__I__I__I__I__I__I	I__I,I__I__I	I__I__I__I,I__I	Tag 1I__I__I__I,I__I<br>Tag 2I__I__I__I,I__I<br>Tag 3I__I__I__I,I__I	❒	❒	❒	❒
8	I__I__I__I__I__I__I	I__I,I__I__I			❒			❒
9	I__I__I__I__I__I__I	I__I,I__I__I			❒			❒
**10**	I__I__I__I__I__I__I	I__I,I__I__I	I__I__I__I,I__I	Tag 1I__I__I__I,I__I<br>Tag 2I__I__I__I,I__I<br>Tag 3I__I__I__I,I__I	❒	❒	❒	❒
**13**	I__I__I__I__I__I__I	I__I,I__I__I	I__I__I__I,I__I		❒	❒		❒
**17**	I__I__I__I__I__I__I	I__I,I__I__I	I__I__I__I,I__I		❒	❒		❒
**21**	I__I__I__I__I__I__I	I__I,I__I__I	I__I__I__I,I__I		❒	❒		❒
**24**	I__I__I__I__I__I__I				**MRT** (Befund an Studienleitung) (Bilder an radiol.Ref.zentrum)			

Bitte Toxizitätsbogen für jeden Zyklus und Responsebogen nach MRT ausfüllen!

Therapieabbruch: ❒ Nein ❒ Ja, Datum I__I__I.I__I__I.I__I__I__I__I

Grund: ❒ Progression ❒ Compliance
❒ Toxizität ❒ Allergie
❒ andere:________________________

.... Datum

.... Unterschrift/Stempel (des dokumentierenden Arztes)

22.8.2.1. Tumoren im Kindesalter – Kooperative Dokumentation

Apr. 2004

In Zusammenarbeit mit dem Deutschen Kinderkrebsregister am IMBEI, 55101 Mainz,
Tel. 06131/17-3227, Fax 06131/17-4462

SIOP-LGG 2004 - Chemotherapie – Protokoll-Konsolidierung
Woche 25 – 54

1/1

Studienleitung: **Frau Dr. Astrid K. Gnekow**, I. Klinik f. Kinder u. Jugendliche, Klinikum Augsburg, Stenglinstr. 2, 86156 Augsburg, Tel.: 0049 - (0) 821 - 400 - 3615, Fax: -3616 , Email: gnekow.hit-lgg@klinikum-augsburg.de

Name, Vorname I________________________________I   Pat.-Nr. I_I_I_I_I   Klinik I_I_I_I_I   Pat. Identifikationszahl I__I **I__I__I__I__I__I__I** I__I__I__I__I__I

GPOH-PID: I__I__I__I__I__I__I__I__I__I   Geb. Datum

Körpergröße: I__I__I__I cm
Körpergewicht: I__I__I,I__I kg
Körperoberfläche: I__I,I__I__I m²

**Woche**	**25**	**31**	**37**	**43**	**49**	**54**
	55	61	67	73	79	85
	**VVV C**	**VVV C**	**VVV C**	**VVV C**	**VVV C**	**MRT**

Vincristin 1,5 mg/m² iv-Bolus - Tag 1, 8, 15
Carboplatin 550 mg/m² 1 h iv - Tag 1

**Woche**	**Datum**	**Vincristin Dosis (mg)**	**Carboplatin Dosis (mg)**	**Dosismodifikation von: V**	**C**	**Toxizität:** (Grad 3 oder 4, Art)
**25**	I__I__I__I__I__I__I	I__I,I__I__I	I__I__I__I,I__I	❒	❒	❒
26	I__I__I__I__I__I__I	I__I,I__I__I		❒		❒
27	I__I__I__I__I__I__I	I__I,I__I__I		❒		❒
**31**	I__I__I__I__I__I__I	I__I,I__I__I	I__I__I__I,I__I	❒	❒	❒
32	I__I__I__I__I__I__I	I__I,I__I__I		❒		❒
33	I__I__I__I__I__I__I	I__I,I__I__I		❒		❒
**37**	I__I__I__I__I__I__I	I__I,I__I__I	I__I__I__I,I__I	❒	❒	❒
38	I__I__I__I__I__I__I	I__I,I__I__I		❒		❒
39	I__I__I__I__I__I__I	I__I,I__I__I		❒		❒
**43**	I__I__I__I__I__I__I	I__I,I__I__I	I__I__I__I,I__I	❒	❒	❒
44	I__I__I__I__I__I__I	I__I,I__I__I		❒		❒
45	I__I__I__I__I__I__I	I__I,I__I__I		❒		❒
**49**	I__I__I__I__I__I__I	I__I,I__I__I	I__I__I__I,I__I	❒	❒	❒
50	I__I__I__I__I__I__I	I__I,I__I__I		❒		❒
51	I__I__I__I__I__I__I	I__I,I__I__I		❒		❒
**53**	I__I__I__I__I__I__I			MRT (Befund bitte an Studienleitung u. Bilder an rad. Referenzzentrum)		

Bitte Toxizitätsbogen für jeden Zyklus und Responsebogen nach MRTausfüllen!

Therapieabbruch: ❒ Nein ❒ Ja, Datum I__I__I . I__I__I . I__I__I__I__I

Grund: ❒ Progression ❒ Compliance
❒ Toxizität ❒ Allergie
❒ andere:______________________________

....
Datum

....
Unterschrift/Stempel (des dokumentierenden Arztes)

Apr. 2004

22.8.2.2. Tumoren im Kindesalter – Kooperative Dokumentation

In Zusammenarbeit mit dem Deutschen Kinderkrebsregister am IMBEI, 55101 Mainz, Tel. 06131/17-3227, Fax 06131/17-4462

SIOP-LGG 2004 - Chemotherapie – Protokoll-Konsolidierung
Woche 55 – 85 1/1

Studienleitung: **Frau Dr. Astrid K. Gnekow**, I. Klinik f. Kinder u. Jugendliche, Klinikum Augsburg, Stenglinstr. 2, 86156 Augsburg, Tel.: 0049 - (0) 821 - 400 - 3615, Fax: -3616, Email: gnekow.hit-lgg@klinikum-augsburg.de

Name, Vorname I____________________________________I Pat.-Nr. I_I_I_I_I Klinik I_I_I_I_I Pat. Identifikationszahl I__I I__I__I__I__I__I__I I__I__I__I__I__I

GPOH-PID: I__I__I__I__I__I__I__I__I__I Geb. Datum

Körpergröße: I__I__I__I cm
Körpergewicht: I__I__I,I__I kg
Körperoberfläche: I__I,I__I__I m²

Woche	25 / **55**	31 / **61**	37 / **67**	43 / **73**	49 / **79**	54 / **85**
	**VVVC**	**VVVC**	**VVVC**	**VVVC**	**VVVC**	**MRT**

Vincristin 1,5 mg/m² iv-Bolus - Tag 1, 8, 15
Carboplatin 550 mg/m² 1 h iv - Tag 1

**Woche**	**Datum**	**Vincristin Dosis (mg)**	**Carboplatin Dosis (mg)**	**Dosismodifikation von: V**	**C**	**Toxizität:** (Grad 3 oder 4, Art)
**55**	I__I__I__I__I__I__I	I__I,I__I__I	I__I__I__I,I__I	❐	❐	❐
56	I__I__I__I__I__I__I	I__I,I__I__I		❐		❐
57	I__I__I__I__I__I__I	I__I,I__I__I		❐		❐
**61**	I__I__I__I__I__I__I	I__I,I__I__I	I__I__I__I,I__I	❐	❐	❐
62	I__I__I__I__I__I__I	I__I,I__I__I		❐		❐
63	I__I__I__I__I__I__I	I__I,I__I__I		❐		❐
**67**	I__I__I__I__I__I__I	I__I,I__I__I	I__I__I__I,I__I	❐	❐	❐
68	I__I__I__I__I__I__I	I__I,I__I__I		❐		❐
69	I__I__I__I__I__I__I	I__I,I__I__I		❐		❐
**73**	I__I__I__I__I__I__I	I__I,I__I__I	I__I__I__I,I__I	❐	❐	❐
74	I__I__I__I__I__I__I	I__I,I__I__I		❐		❐
75	I__I__I__I__I__I__I	I__I,I__I__I		❐		❐
**79**	I__I__I__I__I__I__I	I__I,I__I__I	I__I__I__I,I__I	❐	❐	❐
80	I__I__I__I__I__I__I	I__I,I__I__I		❐		❐
81	I__I__I__I__I__I__I	I__I,I__I__I		❐		❐
**85**	I__I__I__I__I__I__I			MRT (Befund bitte an Studienleitung u. Bilder an rad. Referenzzentrum)		

Bitte Toxizitätsbogen für jeden Zyklus und Responsebogen nach MRT ausfüllen!

Therapieabbruch: ❐ Nein ❐ Ja, Datum I__I__I.I__I__I.I__I__I__I__I

Grund: ❐ Progression ❐ Compliance
❐ Toxizität ❐ Allergie
❐ andere:______________________________

.... Datum

.... Unterschrift/Stempel (des dokumentierenden Arztes)

Version Juli 2004

22.8.2.3. Tumoren im Kindesalter – Kooperative Dokumentation

In Zusammenarbeit mit dem Deutschen Kinderkrebsregister am IMBEI, 55101 Mainz,
Tel. 06131/17-3227, Fax 06131/17-4462

SIOP-LGG 2004 - Chemotherapie – Alternative Konsolidierung Woche 1 – 30 nach Carboplatin-Allergie oder PD

1/1

Studienleitung: **Frau Dr. Astrid K. Gnekow**, I. Klinik f. Kinder u. Jugendliche, Klinikum Augsburg, Stenglinstr. 2, 86156 Augsburg, Tel.: 0049 - (0) 821 - 400 - 3615, Fax: -3616 , Email: gnekow.hit-lgg@klinikum-augsburg.de

Name, Vorname I____I Pat.-Nr. I_I_I_I_I Klinik I_I_I_I_I Pat.-Identifikationszahl I__I I__I__I__I__I__I__I__I I__I__I__I__I__I__I

GPOH-PID: I__I__I__I__I__I__I__I__I__I Geb. Datum

Körpergröße: I__I__I__I cm
Körpergewicht: I__I__I,I__I kg
Körperoberfläche: I__I,I__I__I m²

V = Vincristin 1,5 mg/m² iv-Bolus - Tag 1, 8, 15
Cis = Cisplatin 30 mg/m² 3 h iv - Tag 1 + 2
Cyc = Cyclophosphamid 1500 mg/m² 1 h iv, Tag 1

**Woche**					
**1** / **25** / 49	**30**	**7** / 31 / 55	**13** / 37	**19** / 43	61
**VVV Cyc**	**MRT**	**VVV Cis**	**VVV Cyc**	**VVV Cis**	**MRT**

**Wo-che**	**Datum**	**Vincristin Dosis (mg)**	**Cyc / Cis Dosis (mg)**	**Dosismodifikation von: V**	**Cis/Cyc**	**Toxizität:** (Grad 3 oder 4, Art)
**1**	I__I__I__I__I__I__I__I	I__I,I__I__I	**1** Cyc I__I__I__I__I,I__I	❐	❐	❐
2	I__I__I__I__I__I__I__I	I__I,I__I__I		❐		❐
3	I__I__I__I__I__I__I__I	I__I,I__I__I		❐		❐
**7**	I__I__I__I__I__I__I__I	I__I,I__I__I	**7** Cis Tag 1: I__I__I,I__I	❐	❐	❐
8	I__I__I__I__I__I__I__I	I__I,I__I__I	Tag 2:I__I__I,I__I	❐	❐	❐
9	I__I__I__I__I__I__I__I	I__I,I__I__I		❐		❐
**13**	I__I__I__I__I__I__I__I	I__I,I__I__I	**13** Cyc I__I__I__I__I,I__I	❐	❐	❐
14	I__I__I__I__I__I__I__I	I__I,I__I__I		❐		❐
15	I__I__I__I__I__I__I__I	I__I,I__I__I		❐		❐
**19**	I__I__I__I__I__I__I__I	I__I,I__I__I	**19** Cis Tag 1: I__I__I,I__I	❐	❐	❐
20	I__I__I__I__I__I__I__I	I__I,I__I__I	Tag 2: I__I__I,I__I	❐	❐	❐
21	I__I__I__I__I__I__I__I	I__I,I__I__I		❐		❐
**25**	I__I__I__I__I__I__I__I	I__I,I__I__I	**25** Cyc I__I__I__I__I,I__I	❐	❐	❐
26	I__I__I__I__I__I__I__I	I__I,I__I__I		❐		❐
27	I__I__I__I__I__I__I__I	I__I,I__I__I		❐		❐
**30**	I__I__I__I__I__I__I__I			MRT (Befund bitte an Studienleitung, Bilder an Referenzzentrum).		

Bitte Toxizitätsbogen für jeden Zyklus und Responsebogen nach MRT ausfüllen!

Therapieabbruch: ❐ Nein ❐ Ja, Datum I__I__I . I__I__I . I__I__I__I__I
Grund: ❐ Progression ❐ Compliance
❐ Toxizität ❐ Allergie
❐ andere:______________________________

.... Datum

.... Unterschrift/Stempel (des dokumentierenden Arztes)

Version Apr. 2004

22.8.3.Tumoren im Kindesalter – Kooperative Dokumentation

In Zusammenarbeit mit dem Deutschen Kinderkrebsregister am IMBEI, 55101 Mainz,
Tel. 06131/17-3227, Fax 06131/17-4462

SIOP-LGG 2004 - Chemotherapie - Alternative Konsolidierung Woche 31 - 61 nach Carboplatin Allergie oder PD 2/1

Studienleitung: **Frau Dr. Astrid K. Gnekow**, I. Klinik f. Kinder u. Jugendliche, Klinikum Augsburg, Stenglinstr. 2, 86156 Augsburg, Tel.: 0049 - (0) 821 - 400 - 3615, Fax: -3616 , Email: gnekow.hit-lgg@klinikum-augsburg.de

Name, Vorname I____________________________________I Pat.-Nr. I_I_I_I_I Klinik I_I_I_I_I Pat.-Identifikationszahl I__I I__I__I__I__I__I__I I__I__I__I__I__I__I

GPOH-PID: I__I__I__I__I__I__I__I__I__I Geb. Datum

Körpergröße: I__I__I__I cm
Körpergewicht: I__I__I,I__I kg
Körperoberfläche: I__I,I__I__I m²
V = Vincristin 1,5 mg/m² iv-Bolus - Tag 1, 8, 15
Cis = Cisplatin 30 mg/m² 3 h iv - Tag 1 + 2
Cyc = Cyclophosphamid 1500 mg/m² 1 h iv, Tag 1

**Woche**	1		7	13	19	
	25	30	**31**	**37**	**43**	
	**49**		**55**			**61**
	**VVV Cyc**	**MRI**	**VVV Cis**	**VVV Cyc**	**VVV Cis**	**MRI**

**Wo -che**	**Datum**	**Vincristin Dosis (mg)**	**Cyc / Cis Dosis (mg)**	**Dosismodifikation von: V Cyc/Cis Toxizität:** (Grad 3-4, Art)
**31**	I__I__I__I__I__I__I	I__I,I__I__I	**31** Cis Tag 1: I__I__I,I__I	❐ ❐ ❐
32	I__I__I__I__I__I__I	I__I,I__I__I	Tag 2: I__I__I,I__I	❐ ❐ ❐
33	I__I__I__I__I__I__I	I__I,I__I__I		❐ ❐
**37**	I__I__I__I__I__I__I	I__I,I__I__I	**37** Cyc I__I__I__I__I,I__I	❐ ❐ ❐
38	I__I__I__I__I__I__I	I__I,I__I__I		❐ ❐
39	I__I__I__I__I__I__I	I__I,I__I__I		❐ ❐
**43**	I__I__I__I__I__I__I	I__I,I__I__I	**43** Cis Tag 1: I__I__I,I__I	❐ ❐ ❐
44	I__I__I__I__I__I__I	I__I,I__I__I	Tag 2: I__I__I,I__I	❐ ❐
45	I__I__I__I__I__I__I	I__I,I__I__I		❐ ❐
**49**	I__I__I__I__I__I__I	I__I,I__I__I	**49** Cyc I__I__I__I__I,I__I	❐ ❐ ❐
50	I__I__I__I__I__I__I	I__I,I__I__I		❐ ❐
51	I__I__I__I__I__I__I	I__I,I__I__I		❐ ❐
**55**	I__I__I__I__I__I__I	I__I,I__I__I	**55** Cis Tag 1: I__I__I,I__I	❐ ❐ ❐
56	I__I__I__I__I__I__I	I__I,I__I__I	Tag 2: I__I__I,I__I	❐ ❐ ❐
57	I__I__I__I__I__I__I	I__I,I__I__I		❐ ❐
**61**	I__I__I__I__I__I__I			MRI (Befund bitte an Studienleitung, Bilder an Referenzzentrum).

Bitte Toxizitätsbogen für jeden Zyklus und Response-bogen nach MRTausfüllen!

Therapieabbruch: ❐ Nein ❐ Ja, Datum I__I__I . I__I__I . I__I__I__I__I
Grund: ❐ Progression ❐ Compliance
❐ Toxizität ❐ Allergie
❐ andere:______________________________

.... Datum

.... Unterschrift/Stempel (des dokumentierenden Arztes)

22.8.3. Toxizitätsdokumentation

SIOP-LGG 2004 April 2004

Seite 1/2

Bitte für jeden Therapiezyklus ausfüllen und gemeinsam mit der Chemotherapie-Dokumentation einreichen!

Patient:______________________________________ Geb. Datum: I__I__I.I__I__I.I__I__I__I__I

❒ Induktion Vincristin/Carboplatin ❒ Konsolidierung Vincristin/Carboplatin **Woche: I__I__I**

❒ Induktion Vincristin/Carboplatin/Etoposid ❒ Konsolidierung Vincristin/Cisplatin von: I_I_I.I_I_I.I_I_I

❒ Konsolidierung Vincristin/Cyclophosphamid bis : I_I_I.I_I_I.I_I_I

Bitte kreuzen Sie das entsprechende Feld bzw. Parameter an (maximale Toxizität). Details unter Sektion 22.11.

**Grad der Toxizität**	**0**	**I**	**II**	**III**	**IV**
**Hämatologie**					
Hämoglobin (g/l)	Altersnorm	>100	100 - 80	79 - 65	< 65
Leukozyten (G/l)	≥ 4,0	<4,0 – 3,0	2,9 - 2,0	1,9 – 1,0	< 1,0
Granulozyten (G/l)	≥ 2,0	<2,0 – 1,5	1,4 - 1,0	0,9 – 0,5	< 0,5
Thrombozyten (G/l)	≥ 100	<100 - 75	74 - 50	49 - 10	< 10
**Gehör / Audiologie**					
Hörvermögen	normal	nur audiometrischer Hörverlust	Tinnitus, geringe Hypakusis, keine Hörgeräte notwendig	Tinnitus oder Hörverlust mit Hörgeräten korrigierbar	nicht korrigierbare Ertaubung
Beidseitiger Hörverlust (Brock et al, 1991)	<40 dB, alle Frequenzen	>40 dB bei 8000 Hz	>40 dB bei 4000 Hz	>40 dB bei 2000 Hz	>40 dB bei 1000 Hz
**Neurologie**					
Zentrale Neurotoxizität	normal	Vorübergehende Lethargie	moderate Somnolenz oder Agitation	zusätzlich Konfusion, Desorientiertheit, Halluzination	Koma, Krämpfe, toxische Psychose
Neuropathie, Motorik	normal	subjektive Schwäche, kein objektiver Befund	milde objektive Schwäche	objektive Schwäche mit Funktionsstörung	Paralyse
Neuropathie, Sensorik	normal	milde Parästhesien, Verlust tiefer Sehnenreflexe	moderate Parästhesien, objekt. sensorische Funktionsstörung ohne Beeinträchtigung im Alltag	Sensor. Funktionsstörung oder Parästhesie mit Beeinträchtigung im Alltag	bleibender sensorischer Verlust m. Funktionsstörung
Krampfanfälle	keine	-	Krampfanfälle mit erhaltenem Bewußtsein	Krampfanfälle mit gestörtem Bewußtsein	Prolongierte Anfälle (z.B. Status epilepticus)
Abdominale Schmerzen oder Krämpfe	keine	Milde Schmerzen ohne Beeinträchtigung des Allgemeinzustandes	Mäßige Schmerzen ohne Einschränkung im Alltag	Schwere Schmerzen mit Einschränkung im Alltag	Schwere Schmerzen mit schwerwiegender Beeinträchtigung im Alltag
**Infektion/Fieber**					
Infektion	Keine	Leicht	Mäßig schwer	Schwere	Lebensbedrohliche Sepsis, Schock
Fieber	keines	38,0 - 39,0°C	39,1 - 40,0 °C	>40°C für <24 Std.	>40°C für >24 Std.
**Niere**					
Hämaturie	keine	mikroskopisch	Makrohämaturie ohne Koagel	Makrohämaturie mit Koagel	Nekrosen oder tiefe Ulzeration, Transfusion erforderlich
Kreatinin	Altersnorm	< 1,5 x N	> 1,5 - 3,0 x N	> 3,0 - 6,0 x N	> 6,0 x N
Proteinurie (g/24 hrs)	normal bzw. < 0,15	1+ oder 0,15-1,0	2+ - 3+,;1,0-3,5	4+ ; > 3,5	nephrotisches Syndrom
Glomeruläre Filtrationsrate (GFR) (ml/min/1,73 m²)	≥ 90	60 - 89	40 - 59	20 - 39	≤ 19
**Übelkeit/Erbrechen**					
Übelkeit	keine	kann essen	deutlich verminderte Nahrungsaufnahme	praktisch keine Nahrungsaufnahme	-

Erbrechen (Anzahl der Episoden in 24 Std.)	keines	1	2-5	≥ 6 – 10	> 10 oder TPN erforderlich
**Allgemeinsymptome**					
Gewichtsverlust	< 5%	5% - < 10%	10% - <20%	≥ 20%	-
Alopezie	normal	milder Haarausfall	fortgeschrittener Haarausfall	-	-
Essverhalten	normal	Appetitverlust	Orale Nahrungs-aufnahme reduziert	IV-Flüssigkeits-zufuhr erforderlich	Ernährung über naso-gastrale Sonde oder parenteral
Müdigkeit (Fatigue)	keine	Vermehrte Müdigkeit	Mäßige Müdigkeit, Einschränkungen	Erhebl. Aktivitäts-einschränkungen	Bettlägerig, schwere Einschränkung
Allergische Reaktion / Überempfindlichkeits-reaktion	keine	Flüchtiges Exan-them, Fieber < 38°C	Urtikaria, Fieber ≥38°C, asymptom. Bronchospasmus	Symptomat. Bronchospasmus ±Urticaria, Angioödem	Anaphylaxie
**Gastroenterologie**					
Mukositis	keine	Erythem der Mukosa	ungleichmäßige pseudomembranös e Läsionen (≤ 1,5 cm Durchmesser, nicht konfluierend)	konfluierende pseudomembranös e Läsionen (> 1,5 cm Durchmesser)	Nekrosen oder tiefe Ulzerationen; Blutungsneigung ohne mechanische Einwirkung
Stomatitis / Pharyngitis	keine	schmerzloses Ulkus, Erythem	schmerzhaftes Erythem oder Ulkus, Nahrungs-aufnahme möglich	schmerzhaftes Erythem, Ulzera, i. v.-Substitution erforderlich	schwere Ulzera, TPN erforderlich
Diarrhöe (Anstieg der Stuhlfrequenz)	keine	2-3 Stühle / Tag	4-6 Stühle / Tag oder nächtlicher Stühle	Anstieg auf ≥7 Stühle / Tag oder Inkontinenz oder parenterale Flüssigkeit wegen Dehydration	≥ 10 Stühle / Tag, blutige Durchfälle oder Kreislaufkollaps, physiologische Aus-wirkungen erfordern Intensivbehandlung
Obstipation	keine	Geringe Obstipation	Mäßige Obstipation	Starke Obstipation, beginnender Subileus	Obstruktion, Ileus > 96 Stunden
**Dermatologie**					
Radiogene Dermatitis	keine	schwaches Erythem oder trockene Desquamation	Mäßiges bis deut-lichesErythem oder fleckige, feuchte Desquamation, beschränkt auf Hautfalten, mäßiges Ödem	konfluierende feuchte Desquama-tion, ≥1,5 cm Durchmesser, nicht auf Hautfalten begrenzt, deutliches Ödem	Exfoliative Dermatitis, Hautnekrosen oder Ulzerationen bis in die Tiefe der Dermis, Blutungsneigung ohne signifikantes Trauma oder Abschürfung
**Leber**					
Bilirubin (x N)	Altersnorm	≤ 1,5 x N	> 1,5 - 3,0 x N	> 3,0 - 10,0 x N	> 10,0 x N
SGOT/SGPT (x N)	Altersnorm	≤ 2,5 x N	> 2,5 - 5,0 x N	> 5,0 - 20,0 x N	> 20,0 x N
**Lunge**					
Dyspnoe	keine	Keine Symptome, patholog. Lungen-funktionstest	Dyspnoe unter starker Belastung	Dyspnoe unter normaler Belastung	Ruhedyspnoe oder mechan. Beatmung notwendig
**Herz**					
Kardiomyopathie (linksventrikuläre Funktion)	normal	Asymptomatischer Rückgang der Ejektionsfraktion in Ruhe um ≥ 10%, aber <20% des Ausgangswertes	Asymptomatischer Rückgang der Ejektionsfraktion in Ruhe um ≥20% des Ausgangswertes	Milde Kardiomyopathie, medikamentös kompensiert	Schwere oder refraktäre Kardiomyopathie
LV-FS Echokardiographie	> 30%	26% - 30%	21% - 25%	16% - 20%	< 16%

_______________ _______________ _______________

Datum Stempel Unterschrift

Apr. 2004

22.8.4. Tumoren im Kindesalter – Kooperative Dokumentation

In Zusammenarbeit mit dem Deutschen Kinderkrebsregister am IMBEI, 55101 Mainz,
Tel. 06131/17-3227, Fax 06131/17-4462

SIOP-LGG 2004 - Chemotherapie - Responsebeurteilung

Studienleitung: **Frau Dr. Astrid K. Gnekow**, I. Klinik f. Kinder u. Jugendliche, Klinikum Augsburg, Stenglinstr. 2, 86156 Augsburg, Tel.: 0049 - (0) 821 - 400 - 3615, Fax: -3616 , Email: gnekow.hit-lgg@klinikum-augsburg.de

Name, Vorname I_______________________________I Pat.-Nr. I_I_I_I_I Klinik I_I_I_I_I Pat.-Identifikationszahl I__I I__I__I__I__I__I__I I__I__I__I__I__I

GPOH-PID: I__I__I__I__I__I__I__I__I__I Geb. Datum

Tumorresponse: zu Woche 24 ❒ / 54 ❒ /85 ❒ / nach ..... Monaten ❒ / nach ..... Jahren ❒

Datum der Bildgebung I_I_I . I_I_I . I_I_I_I_I **Datum des klinischen Befundes** I_I_I . I_I_I . I_I_I_I_I

Neurologische Response (Bezug zu vorbestehenden Symptomen)

❒ Rückgang ❒ Progression

❒ Stabilisierung ❒ Neuauftreten v. Symptomen ❒ nicht durchgeführt

Ophthalmologischer Befund (Visus)

❒ besser ❒ unverändert ❒ schlechter ❒ nicht durchgeführt

Radiologischer Befund

❒ CR (komplette Remission) ❒ SD (Tumorreduktion um < 25%, aber kein Hinweis für PD)

❒ PR (part. Remission, Tumorred. > 50%)

❒ OR (Tumorreduktion um 25-50%) ❒ PD (Tumorprogression von > 25%)

Tumorgröße (cm x cm x cm) I__I__I x I__I__I x I__I__I (Bitte Befund an die Studienleitung mitschicken!)

MRT-Bilder zur zentralen Beurteilung versandt? ❒ Ja ❒ Nein ❒ Veranlasst

Auftreten von Allergien

❒ **Nein** ❒ **Ja:** Erstmals aufgetreten am: I__I__I . I__I__I . I__I__I__I__I

in Therapiewoche I__I__I

**Reaktion auf:**	**Aufgetretene Nebenwirkungen**:	**Therapie der Allergie:**
❒ Carboplatin	❒ Juckreiz (I°)	______________________
❒ Cyclophosphamid	❒ Exanthem (I°)	______________________
❒ Cisplatin	❒ Urticaria (II°)	______________________
❒ Actinomycin D	❒ Bronchospasmus (III°)	______________________
❒ VP 16	❒ Anaphylaxie (IV°)	______________________
❒ ____________________	❒ andere: __________________	______________________

Im Verlauf: ❒ Dosis modifiziert ❒ Therapie abgebrochen

❒ Therapieumstellung auf ________________ ❒ andere:______________________

Bemerkungen:

______________________ ______________ ______________________________

Stempel Datum Unterschrift des dokumentierenden Arztes

22.9.1. Tumoren im Kindesalter – Kooperative Dokumentation

SIOP-LGG 2004 - Radiotherapie-Dokumentation 1/11

- Patientendaten . Maximale, akute Toxizität während der Strahlentherapie -

Studienleitung: **Herr Prof Dr. R.-D. Kortmann, Studien-u. Referenzzentrum** Universitätsklinik Leipzig, Johannisallee 34, 04103 Leipzig, Tel. 0049 (0)341 9718542, Fax. 0049-(0)341 9718549 Email: rolf-dieter.kortmann@medizin.uni-leipzig.de

Name, Vorname Pat.-Nr. Klinik Pat.-Identifikationszahl

I____I I_I_I_I_I I_I_I_I_I I__I **I__I__I__I__I__I__I__I** I__I__I__I__I__I

GPOH-PID: |__|__|__|__|__|__|__|__|__| Geb. Datum

Sex I__I (männlich= 1, weiblich= 2) Diagnose____

Behandlungszentrum ____ (Klinikscode :_____________)

Radiotherapie: Erstbehandlung I__I (ja=1,nein=2), **nach Chemotherapie I__I** (ja=1, nein=2)

Therapiebeginn I__I__I . I__I__I . I__I__I **Therapieende** I__I__I . I__I__I . I__I__I

Dokumentation der Brachytherapie Seite 8/11, zur Dokumentation der Toxizität der Brachytherapie Seite 1 und 2/11 verwenden.

ZIELVOLUMEN	*Gesamtdosis (Gy)*	*Einzeldosis (Gy)*	*Fraktionen/Woche*
**Primärtumor**			

Unterbrechung der Therapie I__I (ja= 1, nein= 2) Abbruch der Therapie I__I (ja= 1, nein= 2)

Unterbrechung/Intervall von I__I__I . I__I__I . I__I__I bis I__I__I . I__I__I . I__I__I = I__I__I Tage

Grund: ____

Bitte Grad "0" eintragen , wenn keine Toxizität vorlag!

**MAXIMALE AKUTE TOXIZITÄT WÄHREND DER STRAHLENTHERAPIE/ DEUTSCHE VERS. NACH CTC (5/97)***					
	**1**	**2**	**3**	**4**	**Grade**
**Neurotoxizität**					
Kopfschmerzen	Gering, kurzfristig	Mäßig bis stark, aber vorübergehend	Sehr stark und langfristig anhaltend	Lebensbedrohlich	☐
Übelkeit/ Erbrechen	Gering, normale Nahrungsauf-Nahme möglich	Mäßig, Nahrungsaufnahme vermindert	Stark, keine Nahrungsaufnahme möglich	Lebensbedrohlich, oder parenterale Ernährung	☐
Cerebrale Krampfanfälle	-	Einzelner, kurzer Krampfanfall der durch ein Antikonvulsivum gut kontrollierbar ist oder seltene motorische fokale Anfälle	Krampfanfälle, mit Bewusstseinsstörung, schlecht kontrol-lierbare Krampfanfälle	Krampfanfälle jeglicher Form, die wiederkehrend oder schlecht kontrollierbar sind (z.B. Status Epilepticus, therapie-resistente Epilepsie)	☐
Bewusstseins-störung	Leichte Somnolenz oder agitierte Stimmungslage	Mäßige Somnolenz oder agitierte Stimmungslage	Starke Somnolenz, Agitiertheit, Desorientierung oder Halluzinationen	Koma, Anfälle oder toxische Psychose	☐
**Infektionen**	Gering, nicht therapiebedürftig	Mäßig, orale Antibiotika nötig	Stark, i.V. Antibiotika/Antimykotika	Lebensbedrohliche Sepsis	☐
Bitte spezifizieren	1 bakteriell, 2 viral, 3 Pilzinfection, 4 Kombination, 5 andere, 6 kein Keim isoliert				☐
**Haut**	Geringes Erythem, Epilation, trockene Dequamaation,red. Schweißsekretion	Mäßiges Erythem, vereinzelt feuchte Epitheliolyse (< 50%),mäßiges Ödem, lokale Therapie nötig	Ausgeprägtes Erythem, konfluierende feuchte Epitheliolyse (>50%), starkes Ödem, intensive lokale Therapie nötig	Tiefe Ulzera, Hämorrhagie oder Nekrose, operative Therapie nötig	☐
**Schleimhäute**	Geringes Erythem, Beläge oder Schmerzen, keine Therapie nötig	Fleckige, serosanguinöse Mukositis oder Schmerzen ohne Narkotikabedarf	Konfluent fibrinöse Mukositis, Ulzeration oder Narkotika zur Schmerzbehandlung nötig	Nekrose, tiefe Ulzera oder Hämorrhagie, parenterale Ernährung	☐
**Ohr/Gehörgang**	Geringes Erythem. Otitis externa, Pruritus, keine Therapie	Mäßige (seröse) Otitis externa et media, lokale Therapie nötig	Starke serosanguinöse Otitis externa et media, intensive Therapie nötig	Weichteil und/oder Knochennekrose	☐

Nicht aufgeführte Toxizität: ____

Datum: ________________________ **Unterschrift/Stempel:**____________________________________

* modified January 2006

22.9.2. Tumoren im Kindesalter – Kooperative Dokumentation

In Zusammenarbeit mit dem Deutschen Kinderkrebsregister am IMBEI, 55101 Mainz, Tel. 06131/17-3227, Fax 06131/17-4462

SIOP-LGG 2004 – Radiotherapie-Dokumentation 2/11

- Maximale akute Morbidität am Ende der Strahlentherapie -

Studienleitung: **Herr Prof Dr. R.-D. Kortmann, Studien-u. Referenzzentrum**, Universitätsklinik Leipzig, Johannisallee 34, 04103 Leipzig, Tel. 0049 (0)341 9718542, Fax. 0049-(0)341 9718549 Email: rolf-dieter.kortmann@medizin.uni-leipzig.de

Name, Vorname Pat.-Nr. Klinik Pat.-Identifikationszahl

I____I I_I_I_I_I I_I_I_I_I I__I I__I__I__I__I__I__I I__I__I__I__I__I

GPOH-PID: I__I__I__I__I__I__I__I__I__I Geb. Datum

Bitte Grad "0" eintragen , wenn keine Toxizität vorlag!

MAXIMALE AKUTE TOXIZITÄT AM ENDE DER STRAHLENTHERAPIE/ DEUTSCHE VERS. NACH CTC (5/97) *					
	**1**	**2**	**3**	**4**	**Grade**
**Neurotoxizität**					
Kopfschmerzen	Gering, kurzfristig	Mäßig bis stark, aber vorübergehend	Sehr stark und langfristig anhaltend	Lebensbedrohlich	☐
Übelkeit/ Erbrechen	Gering, normale Nahrungsauf-Nahme möglich	Mäßig, Nahrungsaufnahme vermindert	Stark, keine Nahrungsaufnahme möglich	Lebensbedrohlich, oder parenterale Ernährung	☐
Cerebrale Krampfanfälle	-	Einzelner, kurzer Krampfanfall der durch ein Antikonvulsivum gut kontrollierbar ist oder seltene motorische fokale Anfälle	Krampfanfälle, mit Bewusstseinsstörung, schlecht kontrol-lierbare Krampfanfälle	Krampfanfälle jeglicher Form, die wiederkehrend oder schlecht kontrollierbar sind (z.B. Status Epilepticus, therapieresistente Epilepsie)	☐
Bewusstseins-störung	Leichte Somnolenz oder agitierte Stimmungslage	Mäßige Somnolenz oder agitierte Stimmungslage	Starke Somnolenz, Agitiertheit, Desorientierung oder Halluzinationen	Koma, Anfälle oder toxische Psychose	☐
**Infektionen**	Gering, nicht therapiebedürftig	Mäßig, orale Antibiotika nötig	Stark, i.V. Antibiotika/Antimykotika	Lebensbedrohliche Sepsis	☐
Bitte spezifizieren	1 bakteriell, 2 viral, 3 Pilzinfection, 4 Kombination, 5 andere, 6 kein Keim isoliert				☐
**Haut**	Geringes Erythem, Epilation, trockene Dequamaation,red. Schweißsekretion	Mäßiges Erythem, vereinzelt feuchte Epitheliolyse (< 50%),mäßiges Ödem, lokale Therapie nötig	Ausgeprägtes Erythem, konfluierende feuchte Epitheliolyse (>50%), starkes Ödem, intensive lokale Therapie nötig	Tiefe Ulzera, Hämorrhagie oder Nekrose, operative Therapie nötig	☐
**Schleimhäute**	Geringes Erythem, Beläge oder Schmerzen, keine Therapie nötig	Fleckige, serosanguinöse Mukositis oder Schmerzen ohne Narkotikabedarf	Konfluent fibrinöse Mukositis, Ulzeration oder Narkotika zur Schmerzbehandlung nötig	Nekrose, tiefe Ulzera oder Hämorrhagie, parenterale Ernährung	☐
**Ohr/Gehörgang**	Geringes Erythem. Otitis externa, Pruritus, keine Therapie	Mäßige (seröse) Otitis externa et media, lokale Therapie nötig	Starke serosanguinöse Otitis externa et media, intensive Therapie nötig	Weichteil und/oder Knochennekrose	☐

Nicht aufgeführte Toxizität: ____

____

! Bitte Rücksendung an das Referenzzentrum für Strahlentherapie innerhalb eines Monats !

Datum: ________________________ **Unterschrift/Stempel:**___________________________________

* modified January 2006

Apr. 2004

22.9.3. Tumoren im Kindesalter – Kooperative Dokumentation

In Zusammenarbeit mit dem Deutschen Kinderkrebsregister am IMBEI, 55101 Mainz,
Tel. 06131/17-3227, Fax 06131/17-4462

SIOP-LGG 2004 – Radiotherapie-Dokumentation 3/11

- Myelotoxizität während der kranio-spinalen Bestrahlung -

Studienleitung: **Herr Prof Dr. R.-D. Kortmann, Studien-u. Referenzzentrum**, Universitätsklinik Leipzig, Johannisallee 34, 04103 Leipzig, Tel. 0049 (0)341 9718542, Fax. 0049-(0)3419718549 Email: rolf-dieter.kortmann@medizin.uni-leipzig.de

Name, Vorname Pat.-Nr. Klinik Pat.-Identifikationszahl

I____I I_I_I_I_I I_I_I_I_I I__I I__I__I__I__I__I__I I__I__I__I__I__I

GPOH-PID: I__I__I__I__I__I__I__I__I Geb. Datum

Maximale Myelotoxizität während der Strahlentherapie

(Auszufüllen nur nach kranio-spinaler Strahlentherapie)

	DATUM<br>T T M M J J J J	BEFUND
BLUTBILD ZU BEGINN DER THERAPIE	└┴┘ └┴┘ └┴┴┴┘	
Leukozyten		└┴┴┴┴┘ (x10^9/L)
Thrombozyten		└┴┴┴┴┘ (x10^9/L)
BLUTBILD – NADIR WÄHREND DER THERAPIE		
Leukozyten	└┴┘ └┴┘ └┴┴┴┘	└┴┴┴┴┘ (x10^9/L)
Thrombozyten	└┴┘ └┴┘ └┴┴┴┘	└┴┴┴┴┘ (x10^9/L)
BLUTBILD AM ENDE DER THERAPIE	└┴┘ └┴┘ └┴┴┴┘	
Leukozyten		└┴┴┴┴┘ (x10^9/L)
Thrombozyten		└┴┴┴┴┘ (x10^9/L)
Gabe von hämatopoetischen Wachstumsfaktoren	Ja ☐ Nein ☐	
Transfusionen / Erythrozyten	Ja ☐ Nein ☐	
Transfusionen / Thrombozyten	Ja ☐ Nein ☐	

Datum: Unterschrift/Stempel:

22.9.4. Tumoren im Kindesalter – Kooperative Dokumentation

In Zusammenarbeit mit dem Deutschen Kinderkrebsregister am IMBEI, 55101 Mainz,
Tel. 06131/17-3227, Fax 06131/17-4462

SIOP-LGG 2004 – Radiotherapie-Dokumentation 4/11

- Therapietechnik – Primärtumor -

Studienleitung: **Herr Prof Dr. R.-D. Kortmann, Studien-u. Referenzzentrum**, Universitätsklinik Leipzig, Johannisallee 34, 04103 Leipzig, Tel. 0049 (0)341 9718542, Fax. 0049-(0)3419718549 Email: rolf-dieter.kortmann@medizin.uni-leipzig.de

Name, Vorname Pat.-Nr. Klinik Pat.-Identifikationszahl
I_______________________________________I I_I_I_I_I I_I_I_I_I I__I **I__I__I__I__I__I__I** I__I__I__I__I__I
GPOH-PID: |__|__|__|__|__|__|__|__| Geb. Datum

THERAPIETECHNIK: PRIMÄRTUMOR:

Linear beschleuniger (Energie) _______ MeV I__I (ja= 1,nein= 2)
CO-60 I__I (ja= 1,nein= 2)
CT-basierter Plan I__I (ja= 1,nein= 2) 3-D Behandlungsplanung I__I (ja= 1,nein= 2)
IMRT I__I (ja= 1,nein= 2) Planungs-MRT I__I (ja= 1,nein= 2)
seitliche Gegenfelder I__I (ja= 1,nein= 2) Mehrfeldertechnik I__I (ja= 1,nein= 2)
Keilfilter: I__I (ja= 1,nein= 2) Rotation I__I (ja= 1,nein= 2)
Dosis / Referenzpunkt _______Gy Max.______% Min.______% „hot spot" ______%
Fusion von MRT und CT für die Behandlungsplanung I__I (ja= 1,nein= 2)
Protonen: I__I (ja= 1, nein= 2)

Dosisbelastung von kritischen Nachbarorganen (Dosisvolumen-Histogramm) in Gy:

Organ	***Maximum***	***Minimum***	***Mittel***
Hypophyse			
Chiasma opticum			
Hypothalamus			
Hirnstamm			
linker Nervus opticus			
rechter Nervus opticus			
linke Augenlinse			
rechte Augenlinse			
linkes Innenohr			
rechtes Innenohr			

Lagerungshilfen:

Individuelle, konventionelle Gesichtsmaske I__I (ja= 1, nein= 2)

Rigide Fixation des Kopfes mittels einer Gesichtsmaske I__I (ja= 1, nein= 2)

Stereotaktische Immobilisierung (z. B. Gill-Thomas-Cosman Ring) I__I (ja= 1, nein= 2)

Andere: I__I (ja= 1, nein= 2)

Bitte erläutern____

Bemerkungen:

Datum Unterschrift/Stempel (des dokumentierenden Arztes)

22.9.5. Tumoren im Kindesalter – Kooperative Dokumentation

In Zusammenarbeit mit dem Deutschen Kinderkrebsregister am IMBEI, 55101 Mainz,
Tel. 06131/17-3227, Fax 06131/17-4462

SIOP-LGG 2004 – Radiotherapie-Dokumentation 5/11

- Dosisverschreibung – kraniospinale Achse -

Studienleitung: **Herr Prof Dr. R.-D. Kortmann, Studien-u.Referenzzentrum**, Universitätsklinik Leipzig, Johannisallee 34, 04103 Leipzig, Tel. 0049 (0)341 9718542, Fax. 0049-(0)341 9718549 Email: rolf-dieter.kortmann@medizin.uni-leipzig.de

Name, Vorname Pat.-Nr. Klinik Pat.-Identifikationszahl
I____I I_I_I_I_I I_I_I_I_I I__I I__I__I__I__I__I__I I__I__I__I__I__I
GPOH-PID: I__I__I__I__I__I__I__I__I__I Geb. Datum

Kraniospinale Achse

GESAMTDOSEN / FRAKTIONIERUNG:

Zielvolumen	Gesamtherddosis		Einzelherddosis		Fraktionen/Tag	Behandlungstage
Gehirn	└┴┘·└┘	Gy	└┴┘·└┘	Gy	└┴┘	└┴┘
Spinalkanal	└┴┘·└┘	Gy	└┴┘·└┘	Gy	└┴┘	└┴┘
Boost auf Metastasen	└┴┘·└┘	Gy	└┴┘·└┘	Gy	└┴┘	└┴┘
Boost auf Primärtumor	└┴┘·└┘	Gy	└┴┘·└┘	Gy	└┴┘	└┴┘

Dosisbelastung von kritischen Nachbarorganen (Dosisvolumen-Histogramm) in Gy:

Organ	*Maximum*	*Minimum*	*Mittel*
Hypophyse			
Chiasma opticum			
Hypothalamus			
Hirnstamm			
linker Nervus opticus			
rechter Nervus opticus			
linke Augenlinse			
rechte Augenlinse			
linkes Innenohr			
rechtes Innenohr			

Datum Unterschrift/Stempel (des dokumentierenden Arztes)

22.9.6. Tumoren im Kindesalter – Kooperative Dokumentation

In Zusammenarbeit mit dem Deutschen Kinderkrebsregister am IMBEI, 55101 Mainz,
Tel. 06131/17-3227, Fax 06131/17-4462

HIT-LGG – Radiotherapie-Dokumentation 6 / 11

- Therapietechnik – kraniospinale Strahlentherapie -

Studienleitung: **Herr Prof Dr. R.-D. Kortmann, Studien-u. Referenzzentrum,** Universitätsklinik Leipzig, Johannisallee 34, 04103 Leipzig, Tel. 0049 (0)341 9718542, Fax. 0049-(0)341 9718549 Email: rolf-dieter.kortmann@medizin.uni-leipzig.de

Name, Vorname Pat.-Nr. Klinik Pat.-Identifikationszahl
I____I I_I_I_I_I I_I_I_I_I I__I I__I__I__I__I__I__I__I I__I__I__I__I__I__I
GPOH-PID: |__|__|__|__|__|__|__|__|__| Geb. Datum

I. "Helm-Technik"

Beginn der Strahlentherapie |__|__| . |__|__| . |__|__| Ende der Strahlentherapie |__|__| . |__|__| . |__|__|

Linearbeschleuniger (Energie) I__I__I MeV

Seitliche, isozentrische Gegenfelder I__I (ja= 1, nein= 2)

Falls nein, bitte erläutern:____

Lagerungshilfen:

Individuelle Gesichtsmaske: I__I (ja= 1, nein= 2)

Vakuum-Kissen: I__I (ja= 1, nein= 2)

Schale zur Immobilisierung: I__I (ja= 1, nein= 2)

Andere: ____

Bauchlage: I__I (ja= 1,nein= 2)

Rückenlage: I__I (ja= 1, nein= 2)

II. Spinalachse

Beginn der Strahlentherapie |__|__| . |__|__| . |__|__| Ende der Strahlentherapie |__|__| . |__|__| . |__|__|

Linearbeschleuniger (Energie): I__I__I MeV

Dosis am Referenzpunkt für das obere Feld: I__I__I cm

Dosis am Referenzpunkt für das untere Feld: I__I__I cm

SSD : I__I__I__I__I cm

Maximaldosis : I__I__I , I__I Gy

Minimaldosis I__I__I , I__I Gy

Elektronenfelder: I__I (ja= 1, nein= 2)

Energie: I__I__I MeV

Dosisspezifikation : I__I__I % Isodose (Elektronenfeld)

Lagerungshilfen

Individuelle Gesichtsmaske: I__I (ja= 1, nein= 2)

Vakuumkissen: I__I (ja= 1, nein= 2)

Schale zur Immobilisierung: I__I (ja= 1, nein= 2)

Andere: ________________________ Bauchlage: I__I Rückenlage: I__I

____

Datum Unterschrift/Stempel (des dokumentierenden Arztes)

22.9.7. Tumoren im Kindesalter – Kooperative Dokumentation

In Zusammenarbeit mit dem Deutschen Kinderkrebsregister am IMBEI, 55101 Mainz,
Tel. 06131/17-3227, Fax 06131/17-4462

SIOP-LGG 2004 – Radiotherapie-Dokumentation 7/11

- Strahlentherapie für metastatische Absiedelungen -

Studienleitung: **Herr Prof Dr. R.-D. Kortmann, Studien-u. Referenzzentrum**, Universitätsklinik Leipzig, Johannisallee 34, 04103 Leipzig, Tel. 0049 (0)341 9718542, Fax. 0049-(0)341 9718549 Email: rolf-dieter.kortmann@medizin.uni-leipzig.de

Name, Vorname Pat.-Nr. Klinik Pat.-Identifikationszahl
I_______________________________________I I_I_I_I_I I_I_I_I_I I__I **I__I__I__I__I__I__I** I__I__I__I__I__I
GPOH-PID: I__I__I__I__I__I__I__I__I__I Geb. Datum

PRIMÄRTUMORSITZ____

Metastasen (Lokalisation:____)
(Bitte Kopien anfertigen entsprechend der Zahl bestrahlter Metastasen, falls erforderlich)

Beginn der Bestrahlung I__I__I . I__I__I . I__I__I Ende der Bestrahlung I__I__I . I__I__I . I__I__I

Linearbeschleuniger (Energie) I__I__I MeV

CT-assistierte Planung I__I (ja= 1, nein= 2)

3-D Behandlungsplanung I__I (ja= 1, nein= 2)

Planungs-MRT I__I (ja= 1, nein= 2)

IMRT I__I (ja= 1, nein= 2)

Bildfusion (MR/CT) für die Behandlungsplanung: I__I (ja= 1, nein= 2)

Seitliche Gegenfelder I__I (ja= 1, nein= 2)

Mehrfeldertechnik I__I (ja= 1,nein= 2)

Keilfilter : I__I (ja= 1, nein= 2)

Dosis am Referenzpunkt I__I__I , I__I Gy

Max. I__I__I__I , I__I % Min. I__I__I__I , I__I % "hot spot" I__I__I__I%

Lagerungshilfen

Individuelle Gesichtsmaske: I__I (ja= 1, nein= 2)

Vakuumkissen: I__I (ja= 1, nein= 2)

Schale zur Immobilisierung: I__I (ja= 1, nein= 2)

Andere: ____

Bauchlage: I__I Rückenlage: I__I

____

Datum Unterschrift/Stempel (des dokumentierenden Arztes)

22.9.7. Tumoren im Kindesalter – Kooperative Dokumentation

In Zusammenarbeit mit dem Deutschen Kinderkrebsregister am IMBEI, 55101 Mainz,
Tel. 06131/17-3227, Fax 06131/17-4462

SIOP-LGG 2004 - Brachytherapie-Dokumentation 8/11
- Technik – Dosisverschreibung -

Studienleitung: **Herr Prof Dr. R.-D. Kortmann, Studien-u. Referenzzentrum** , Universitätsklinik Leipzig, Johannisallee 34, 04103 Leipzig, Tel. 0049 (0)341 9718542, Fax. 0049-(0)341 9718549 Email: rolf-dieter.kortmann@medizin.uni-leipzig.de

Name, Vorname Pat.-Nr. Klinik Pat.-Identifikationszahl
I____I I_I_I_I_I I_I_I_I_I I__I **I__I__I__I__I__I__I** I__I__I__I__I__I
GPOH-PID: I__I__I__I__I__I__I__I__I__I Geb. Datum

Geschlecht I__I (männlich= 1, weiblich= 2).

Diagnose____

behandelndes Zentrum ____*(Kliniks-Code) :____________)*

Brachytherapie als Erstbehandlung I__I (ja= 1, nein= 2)

Brachytherapie nach Chemotherapie: I__I (ja= 1, nein= 2)

Diagnosedatum : I__I__I . I__I__I . I__I__I TT/MM/JJ

Lokalisation/Ausmaß des Tumors :____

Biopsie : I__I (ja= 1, nein= 2)

Isotop: :______________________________

Permanent : I__I (ja= 1, nein= 2) temporär : I__I (ja= 1, nein= 2)

Seed-Implantation : I__I__I . I__I__I . I__I__I TT/MM/JJ

Seed-Entfernung I__I__I . I__I__I . I__I__I TT/MM/JJ

Gesamtherddosis am Tumorrand : | ___ | ___ | ___ | . | ___ | ___ | Gy

Dosierung : | ___ | ___ | . | ___ | Gy pro Stunde

Komplikationen vor Op: | __ | (ja= 1, nein= 2)

Falls ja, welche:____

Beschreibung der Technik:

Bemerkungen:

Datum Unterschrift/Stempel (des dokumentierenden Arztes)

22.9.7. Tumoren im Kindesalter – Kooperative Dokumentation

In Zusammenarbeit mit dem Deutschen Kinderkrebsregister am IMBEI, 55101 Mainz,
Tel. 06131/17-3227, Fax 06131/17-4462

SIOP-LGG 2004 – Radiotherapie-Dokumenation 9/11
- Tumorresponse -

Studienleitung: **Herr Prof Dr. R.-D. Kortmann, Studien-u. Referenzzentrum**, Universitätsklinik Leipzig, Johannisallee 34, 04103 Leipzig, Tel. 0049 (0)341 9718542, Fax. 0049-(0)341 9718549 Email: rolf-dieter.kortmann@medizin.uni-leipzig.de

Name, Vorname Pat.-Nr. Klinik Pat.-Identifikationszahl
I______________________________________I I_I_I_I_I I_I_I_I_I I__I **I__I__I__I__I__I__I** I__I__I__I__I__I
GPOH-PID: I__I__I__I__I__I__I__I__I__I Geb. Datum

Art der vorausgegangenen Radiotherapie:

Externe Radiotherapie : I__I Brachytherapy : I__I

Tumorresponse nach Ende der Radiotherapie / Brachytherapie
nach ❒ 3 / ❒ 6 / ❒ 12 / ❒ .... Monaten / nach ❒ .... Jahren

Datum der Bildgebung I_I_I . I_I_I . I_I_I_I_I **Datum des klinischen Befundes** I_I_I . I_I_I . I_I_I_I_I

Neurologische Response (Bezug zu vorbestehenden Symptomen)
❒ Rückgang ❒ Progression
❒ Stabilisierung ❒ Neuauftreten v. Symptomen ❒ nicht durchgeführt

Ophthalmologischer Befund (Visus)
❒ besser ❒ unverändert ❒ schlechter ❒ nicht durchgeführt

Radiologischer Befund
❒ CR (komplette Remission) ❒ SD (Tumorreduktion um < 25%, aber kein Hinweis für PD)
❒ PR (part. Remission, Tumorred. > 50%)
❒ OR (Tumorreduktion um 25-50%) ❒ PD (Tumorprogression von > 25%)

Tumorgröße: (cm x cm x cm) I__I__I x I__I__I x I__I__I

(Bitte Befund an die Studienleitung mitschicken!)

MRT-Bilder zur zentralen Beurteilung versandt? ❒ Ja ❒ Nein ❒ Veranlasst

Kommentar:____

____

____

Datum: Unterschrift: / Stempel (des dokumentierenden Arztes)

22.9.8. Tumoren im Kindesalter – Kooperative Dokumentation

In Zusammenarbeit mit dem Deutschen Kinderkrebsregister am IMBEI, 55101 Mainz,
Tel. 06131/17-3227, Fax 06131/17-4462

SIOP-LGG 2004 – Radiotherapie-Dokumentation 10/11
- Spätfolgen der Radiotherapie -

Studienleitung: **Herr Prof Dr. R.-D. Kortmann, Studien-u. Referenzzentrum**, Universitätsklinik Leipzig, Johannisallee 34, 04103 Leipzig, Tel. 0049 (0)341 9718542, Fax. 0049-(0)341 9718549 Email: rolf-dieter.kortmann@medizin.uni-leipzig.de

Name, Vorname Pat.-Nr. Klinik Pat.-Identifikationszahl
I____I I_I_I_I_I I_I_I_I_I I__I I__I__I__I__I__I__I__I I__I__I__I__I__I
GPOH-PID: I__I__I__I__I__I__I__I__I__I Geb. Datum

Spätfolgen der Radiotherapie / LENT – SOMA - Bewertungskriterien (jährlich)
1. Jahr ☐ 2. Jahr ☐ 3. Jahr ☐ 4. Jahr ☐ 5. Jahr ☐ nach Therapieende
(Bitte Kopien anfertigen entsprechend der Beobachtungszeit)

Bitte den Ausprägungsgrad der entsprechenden Spätfolgen ankreuzen:

**Haut**	Grad 0	Grad 1	Grad 2	Grad 3	Grad 4
1. Alopezie (Schädel)	❒ Normaler Haarwuchs	❒ Ausdünnung	❒ Fleckiger Haarverlust, dauerhaft	❒ Vollständiger Haarverlust, permanent	-----
2. Änderung der Pigmentation	❒ keine	❒ vorüber-gehend, leicht	❒ permanent, ausgeprägt	-----	------
3. Teleangiekta-sien	❒ keine	❒ wenige	❒ mäßig (< 50% der bestrahlten Hautfläche)	❒ ausgeprägt (> 50% der bestrahlten Hautfläche)	------
4. Fibrose / Vernarbung	❒ keine	❒ vorhanden: asymptomaisch	❒ symptoma-tisch	❒ sekundäre Dysfunktion	❒ vollständige Dysfunktion
5. Nekrosen / Ulzeration	❒ keine	❒ nur epidermal	❒ dermal	❒ subkutan	❒ tiefreichend, Knochen freilegend

**Schleimhaut** **(Oral / Pharyngeal)**	Grad 0	Grad 1	Grad 2	Grad 3	Grad 4
1.Intaktheit der Schleimhaut	❒ normal	❒ fleckige Atrophie oder Teleangiektasien	❒ oberflächliche Ulzeration	❒ tiefe Ulzeration ohne Exposition von Knochen oder Knorpel	❒ tiefe Ulzeration mit Exposition von Knorpel oder Knochen
2. Dysphagie	❒ keine	❒ Schwierigkeiten beim Essen fester Nahrung	❒ Schwierigkeiten beim Essen weicher Nahrung	❒ nur Flüssigkeiten können geschluckt werden	❒ völlige Unmöglichkeit zu schlucken

Datum Unterschrift/Stempel (des dokumentierenden Arztes)

22.9.8. Tumoren im Kindesalter – Kooperative Dokumentation

In Zusammenarbeit mit dem Deutschen Kinderkrebsregister am IMBEI, 55101 Mainz,
Tel. 06131/17-3227, Fax 06131/17-4462

SIOP-LGG 2004 – Radiotherapie- Dokumentation 11/11
- Rezidivmeldung -

Studienleitung: **Herr Prof Dr. R.-D. Kortmann, Studien-u. Referenzzentrum**, Universitätsklinik Leipzig, Johannisallee 34, 04103 Leipzig, Tel. 0049 (0)341 9718542, Fax. 0049-(0)341 9718549 Email: rolf-dieter.kortmann@medizin.uni-leipzig.de

Name, Vorname Pat.-Nr. Klinik Pat.-Identifikationszahl
I_____________________________________I I_I_I_I_I I_I_I_I_I I__I **I__I__I__I__I__I__I** I__I__I__I__I__I
GPOH-PID: I__I__I__I__I__I__I__I__I__I Geb. Datum

I Art der vorangegangenen Radiotherapie
Externe Radiotherapie : I__I **Brachytherapie : I__I**

II. Datum der Diagnose des Rezidives / der Progression I__I__I . I__I__I . I__I__I__I__I
TT/MM/JJJJ

III. Lokalrezidiv ❒ Ja ❒ Nein
(jedes Rezidiv innerhalb der ipsilateralen Hemisphären bzw. im selben supratentoriellen / infratentoriellen / spinalen Kompartiment)

Rezidiv innerhalb des Primärtumorfeldes: I__I (ja= 1, nein= 2)

Falls nein:
- am Feldrand I__I (ja= 1, nein= 2)
- im Feld mit Wachstum, in Richtung
- nach außerhalb des Feldes I__I (ja= 1, nein= 2)
- außerhalb der Feldgrenzen I__I (ja= 1, nein= 2)

Falls außerhalb der Feldgrenzen:
Abstand des Rezidives zum Primärtumor: ____cm____mm
(geringste Distanz bei Beginn der Radiotherapie

Rezidiv innerhalb der └┴┘ % Isodose

Kumulative Gesamtdosis im Bereich des Rezidives: └┴┘ Gy

IV. Fernmetastase ❒ Ja ❒ Nein
(jedes Rezidiv in einem anderen Kompartiment)

Initiale Tumorlokalisation (bitte Einzelheiten angeben):_____________________________________

Lokalisation des Rezidives/der Progression (bitte Einzelheiten angeben): ______________________

____

____
Datum Unterschrift / Stempel (des dokumentierenden Arztes)

22.10. Materialversand SIOP-LGG 2004

22.10.1. Neuropathologie
22.10.1.1. Anleitung zur Asservierung von Tumorgewebe
22.10.1.2. Einsendebogen Hirntumorreferenzzentrum Bonn
22.10.1.3. Einsendebogen Tumorbank

22.10.2. Neuroradiologie
22.10.2.1. Einsendebogen Referenzradiologie – Erstuntersuchung
22.10.2.2. Einsendebogen Referenzradiologie – frühpostoperative Untersuchung
22.10.2.3. Einsendebogen Referenzradiologie - Verlaufsuntersuchung

22.10.1. Anleitung zur Asservierung von Tumorgewebe SIOP-LGG 2004

Anleitung zur Asservierung von Tumorgewebe

A. Benötigtes Material

1. Diese Anleitung

2. Tumorgewebe-Set:
- 20 Superfrost-Objektträger für Tumortupfpräparate
- 5 Objektträger-Boxen
- 1 100 ml Becher für das Handling mit flüssigem Stickstoff
- 7 1,8 ml Standröhrchen für tiefgefrorenes Frischgewebe
  (6 x ROT für Tumor, 1 x GRÜN für Normalgewebe)
- 1 5 ml Citrat-Monovette für Vergleichsblut (DNA-Extraktion)
- 1 4ml Glasmonovette (blau-schwarzer Stopfen) für Leukozytenisolation
- 1 Einsendebogen

3. Bleistift und Permanentmarker (fein) zum Beschriften von Objektträgern und Röhrchen

4. Tumorbox

5. *Sterile* Kompressen, Skalpell, Pinzette, Handschuhe, Deuwer für Stickstoff
Die Sicherheitsvorschriften beim Arbeiten mit flüssigem Stickstoff müssen eingehalten werden.

B. Vorgehensweise

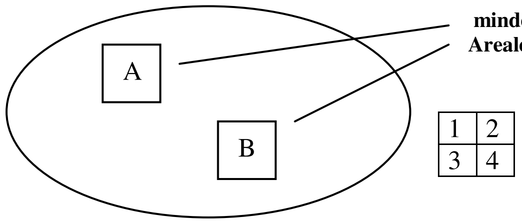

mindestens zwei Tumorstücke A/ B sollten von morphologisch unterschiedlichen Arealen entnommen werden. A und B werden jeweils in vier Stücke geteilt

1 Bevor das Tumorstück in Formalin fixiert wird (für Histologie, örtlicher Pathologe), mind. 10 Tupfpräparate (z. B. für FISH)
2,3,4 Einfrieren in flüssigem Stickstoff

Resektabler Tumor:

1. ***Aufteilen des Tumormaterials***
   Gemeinsam mit dem zuständigen Pathologen Tumor aufschneiden und Gewebeprobe aus unterschiedlichen, aber mindestens zwei repräsentativen Arealen gewinnen **A** und **B** (Größe 1 cm^3, wenn möglich mehr: **C**, **D** etc.; nicht vom Tumorrand, kein Bindegewebe, keine nekrotischen Bezirke asservieren, beim Neuroblastom nodale Areale beachten). Falls mehr Stücke (**C**, **D**) gewonnen werden, neues Tumor-Röhrchenset verwenden. Die Stücke dann jeweils in 4 repräsentative Stücke **A1**, **A2**, **A3**, **A4** und **B1**, **B2**, **B3**, **B4** (**C1**, **C2**, **C3**, **C4** etc.) teilen. Vor der Weiterverarbeitung vorsichtig steril Blut vom Tumorgewebe abtupfen. So schnell wie möglich verarbeiten (optimal: innerhalb von 20 Minuten nach der chirurgischen Entnahme). Übriges Tumorgewebe für den Pathologen zur Diagnostik in Formalin geben.
   Falls bei einem größeren Operationspräparat der Pathologe nicht das gesamte restliche Tumorgewebe zur Diagnostik braucht, übrig gebliebenes Tumorgewebe kleinschneiden, in 50 ml Becher einfrieren und versenden. Welches Tumorgewebe zusätzlich eingefroren werden kann, entscheidet der Pathologe!

2. ***Frischgewebe schockgefrieren***
   50 ml Becher mit flüssigem Stickstoff füllen und Deckel locker auflegen, damit die Verdunstung gering bleibt, jedoch auch kein Druck entsteht.
   1,8 ml Standröhrchen (rot) mit Namen, Geburtsdatum, Operationsdatum und Tumorlokalisation (**A2**, usw.) beschriften.
   Danach aufschrauben. Deckel auf sterile Kompresse legen, Röhrchen im Deuwer mit flüssigem Stickstoff vorkühlen.
   Kompressen, Pinzette und Skalpell steril auspacken und bereitlegen.
   Sterile Handschuhe anziehen (zum Schutz des Gewebes vor RNAsen an den Händen und zur Erhaltung der Sterilität)
   Tumorteile **A, B** in 4 Teile **A1**, **A2**, **A3**, **A4**, **B1**, **B2**, **B3** und **B4** teilen (s. Skizze) und **A2**, **A3**, **A4**, **B2**, **B3**, und **B4** rasch, steril und RNAse-frei schockgefrieren. Falls die Stücke nicht in die Röhrchen passen teilen bzw. in kleine Stücke schneiden.
   Schockgefrieren des Gewebes durch Einfallen-Lassen der Tumorstücke in den flüssigen Stickstoff (im 50ml Becher). Dabei *nicht* mit der Pinzette eintauchen, weil dabei das Tumorgewebe an der Pinzette haften bliebe. Darauf achten, dass die Gewebestücke *nicht* an der Wand des 50 ml Bechers haften.
   Aus vorgekühlten 1,8 ml Röhrchen flüssigen Stickstoff dekantieren. Dabei darauf achten, dass sich kein flüssiger Stickstoff mehr im 1,8ml Röhrchen befindet.
   Schockgefrorenes Tumorgewebe aus dem 50ml Becher in die roten 1,8 ml Röhrchen transferieren, dabei nach **A** und **B** trennen, verschließen (Schraubdeckel) und im flüssigen Stickstoff gefroren halten.
   Auf dem Einsendebogen die Dauer vom Zeitpunkt der Entnahme des Tumorgewebes bis zum Einfrieren notieren.

3. ***Herstellung von Tupfpräparaten und Formalinfixierung von Gewebe***
   2 Gefäße für die Histologie mit Namen, Geburtsdatum und Operationsdatum beschriften und mit gepufferter 4%iger Formalinlösung füllen. (Diese Gefäße sind nicht im Tumorgewebe-Set enthalten, aber in jedem Operationssaal vorhanden.)
   Von den Tumorteilen **A1** und **B1** jeweils zehn Tumortupfpräparate herstellen. *Behutsames* Abtupfen der oberflächlichen Zellschicht der Tumorprobe auf *Superfrost-Objektträger* (ca. 6 Tupfungen pro Schnittfläche, max. 10 Objektträger pro Stück, nicht wischen). Präparate beschriften und *lufttrocknen*.
   Danach die Tumorteile **A1** und **B1** unzerkleinert (!) in je 1 Histologiegefäß mit 4%iger Formalin-Lösung einbringen für den örtlichen Pathologen zur Diagnostik und Bestimmung des Tumoranteils.

Nichtresektabler Tumor:

1. ***Aufteilung des Tumormaterials***
   Zusammen mit dem Pathologen von unterschiedlichen Arealen möglichst 2 repräsentative Tumorstücke **A** und **B** (Größe ca. $1cm^3$) entnehmen (nicht vom Tumorrand, möglichst kein Bindegewebe, keine nekrotischen Bezirke asservieren). Vor der Weiterverarbeitung vorsichtig und steril Blut vom Tumorgewebe abtupfen. **A** und **B** in jeweils 4 repräsentative Tumorstücke **A1**, **A2**, **A3**, **A4** und **B1**, **B2**, **B3**, **B4** teilen (s. o.). Bei kleineren Biopsien gemeinsam mit dem Pathologen besprechen wieviel Tumorgewebe eingefroren werden kann.

2. ***und 3.***
   Verfahren wie bei resektablem Tumor.

C. Gewinnen von Vergleichs-DNA und Leukozyten aus Citratblut und/oder Normalgewebe

Blut:
5-10 ml Begleitblut vom Patienten in Vacutainer-Citrat-Monovetten **(grün)** gewinnen, gut durchmischen (nicht schütteln) und unsepariert im Thermogefäß mit flüssigem Stickstoff einfrieren.
Tumorarten: alle

Glasmonovette **(blau-schwarzer Stopfen)** mit 4ml Blut füllen. Durchmischen und 1x abzentrifugieren: 20 Minuten bei Raumtemperatur, **1650 x g** (Dies entspricht bei einer Zentrifuge mit einem **Radius von 25-30cm** etwa **2300** Umdrehungen/min). Diese Glasmonovette **NICHT** tieffrieren, sondern im Deckel der Tumorbox (zusammen mit Tumortupf) verschicken.
Tumorarten: alle

Normalgewebe:
Wenn bei der gleichen Operation (z.B. Nephrektomie, Leberteilresektion) normales Gewebe aus chirurgisch technischen Gründen mitentfernt werden muß, eignet sich dies als Vergleichsgewebe noch besser. ***Das darf aber keinesfalls zu einer zusätzlichen Resektion oder Erweiterung der Resektionsränder führen.***
Tumorarten: alle
Das Vergleichsgewebe wird wie das Tumorgewebe zerkleinert und im grünen Röhrchen in flüssigem Stickstoff eingefroren.

D. Versand

1. Einsendebogen vollständig ausfüllen und mit dem Material in der Tumorbox an das zuständige Labor senden.
2. Tumorteile **A1** und **B1** bzw. **C1**, **D1** usw. (in 4% Formalin) und übriges Tumorgewebe vom zuständigen örtlichen Pathologen befunden lassen, evtl. mit Bitte um Referenzhistologie.
3. Schockgefrorene Tumorteile **A2**, **A3**, **A4** sowie **B2**, **B3**, **B4** (evtl. **C2**, **C3**, **C4** etc.) und Vergleichsblut bzw. Normalgewebe bis zum Versand bei -70°C oder in flüssigem Stickstoff lagern. Der Versand erfolgt tiefgefroren auf Trockeneis in der Tumorbox an das zuständige molekulargenetische Labor. Die gesamte Kammer der Tumorbox muss mit Trockeneis aufgefüllt werden. Zehn luftgetrocknete Tumortupfpräparate, Glasmonovette und evtl. Serum, Knochenmark im Deckel der Tumorbox (nicht auf Trockeneis) beilegen.

E. Adressen:

Hirntumoren:
Prof. Dr. T. Pietsch
Institut für Neuropathologie der Universität Bonn
Sigmund-Freud-Str. 25
D-53105 Bonn
Tel.: 0228-287 4398

22.10.1.2. Einsendebogen Hirntumorreferenzzentrum SIOP-LGG-2004

Hirntumor-Referenzzentrum

im Auftrag der Deutschen Gesellschaft für Neuropathologie & Neuroanatomie

Leiter: Prof. Dr. T. Pietsch

Institut für Neuropathologie
Universitätsklinikum Bonn

Sigmund-Freud-Straße 25
D – 53105 Bonn
Telefon (0228) 287 6602
Telefax (0228) 287 4331
E-mail: neuropath@uni-bonn.de

Die umrandeten Felder sind vom Einsender auszufüllen

Einsender-Stempel

Telefon:
Telefax:

Therapie-Studie

HIT-LGG

Einsender-Nummer

Eingangsdatum Ref.zentrum

Registernummer

R-

Patientendaten

Name	Vorname	♂	♀	Geburtsdatum	Alter	familiäre Erkrankung

Biopsie	Stereot.	Rezidiv	Autopsie	Liquor	Anamnesedauer	Radiatio ja	Radiatio nein	Chemoth. ja	Chemoth. nein

Lokalisation

☐ supratentoriell	☐ GH-Hemisph.	☐ Stammganglien	☐ Ventrikel	☐ Hirnbasis
☐ infratentoriell	☐ Kleinhirn	☐ Brücke	☐ Medulla obl.	☐ KHBW
☐ Rückenmark	☐ intramedullär	☐ intradural	☐ extradural	☐ Höhe

weitere Angaben zur Lokalisation: ______________________

Diagnose Einsender

Diagnose Referenzzentrum

Bemerkungen ______________________

Sofortige Benachrichtigung per FAX ☐

22.10.1.3. Einsendebogen Tumorbank SIOP-LGG 2004

Hirntumor-Referenzzentrum

Prof. Dr. T. Pietsch

Institut für Neuropathologie
Universitätsklinikum Bonn

Sigmund-Freud-Straße 25
D – 53105 Bonn
Telefon (0228) 287 4398

Tumorbank-Einsendebogen

Patientendaten: Patientenetikett:

Name:..

Vorname:...

Geburtsdatum:..

Geschlecht: [ ]w [ ]m

OP-Datum:...

Diagnose:..

[ ] Erstdiagnose [ ] Verlaufskontrolle [ ] Rezidiv [ ] nach Chemotherapie [ ] nach KMT

Therapie-Studie:...

Bemerkungen (z.B. 2. Rez.):...

....

Untersuchungsmaterial: Entnahme-Datum:.................................

Bitte ankreuzen: Lokalisation:

0 Tumor ....

0 Tumortupfpräparate ....

0 Blut (Monovette grün) für DNA-Extraktion

0 Blut (Glasmonovette) für Leukozytenisolation und Serum

0 Serum

0 Normalgewebe ....

0 tumorzellhaltiges Knochenmark für Molekularbiologie (nicht Immunfluoreszenz!, für IF per Express übersenden)

0 Sonstiges: ....

Ansprechpartner (+ Telefon-Nr.): ....

....

Datum Stempel Unterschrift

22.10.2.1. Begleitschein für MRT/CT –Bilder SIOP-LGG 2004

Frau
PD Dr. med. Monika Warmuth-Metz

Referenzzentrum HIT-LGG

Universitätskliniken Würzburg
Abteilung für Neuroradiologie
Josef-Schneider-Str. 11
97080 Würzburg
Telefon (0931) 201 34799 / 34626
Telefax (0931) 201 34685
E-Mail: hit@neuroradiologie.uni-wuerzburg.de

Erstuntersuchung

Die schattierten Felder sind vom Einsender auszufüllen

Name		Histologie	
Vorname			
Geburtsdatum	❒ männlich ❒ weiblich	Alle Bilder versandt?	❒ ja ❒ nein
Bisherige Therapie:			

**Kranielles**	**❒ MRT oder ❒ CT**	❒ nativ	❒ mit Kontrast	❒ ohne und mit Kontrast
**Spinales**	**❒ MRT oder ❒ CT**	❒ nativ	❒ mit Kontrast	❒ ohne und mit Kontrast

Untersuchungsdatum: I__I__I . I__I__I . I__I__I__I__I

**Tumorlokalisation**						
**Ausdehnung**						
**Ursprung**						
**Größe a/c/s**	cm x cm x cm		cm^2		cm^3	
**Zystengröße**	cm x cm x cm		cm^2		cm^3	
**Begrenzung**	❒ scharf (≥ 90%)		❒ mäßig scharf (≥ 50%)		❒ unscharf < 50%)	
**Ödem**	❒ ipsilateral		❒ kontralateral		Cm	
**Zysten**	❒ Ja	❒ Nein	❒ wie Liquor	❒ heller als Liquor		
**Hydrocephalus**	❒ Nein	❒ leichtgradig	❒ mittelgradig	❒ schwergradig	❒Shunt	
**KM-Enhancement**	❒ kräftig	❒ mittelstark	❒ leicht	❒ kein		
**Anreichernder Tumoranteil**	❒ homogen	❒ überwiegend homogen	❒ überwiegend inhomogen		❒inhomogen	
	❒ 0-25%	❒ 26-50%	❒ 51-75%	❒ 76-100%		
**Meningeose**	❒ nein	❒ fraglich	❒ M2a	❒ M2b	❒ M3a	❒ M3b
**Metastase wo:**						

**Tumorstaging**				
❒ T1	❒ T2	❒ T3 / T3a	❒ T3b	❒ T4
❒ M0	❒ M2	❒ M3		

**⑨ MRT**				
T2	❒ nicht	❒ hyperintens	❒ isointens	❒ hypointens
	❒ homogen	❒ überwiegend homogen	❒ überwiegend inhomogen	❒ inhomogen
T1	❒ nicht	❒ hyperintens	❒ isointens	❒ hypointens
	❒ homogen	❒ überwiegend homogen	❒ überwiegend inhomogen	❒ inhomogen

**⑨ CT**				
Dichte	❒ hypodens	❒ isodens	❒ hyperdens	❒ Blut
	❒ homogen	❒ überwiegend homogen	❒ überwiegend inhomogen	❒ inhomogen
Verkalkungen	❒ nein	❒ grob	❒ fein	

Freitext:

.... Datum

.... Unterschrift / Stempel

22.10.2.2. Begleitschein für MRT/CT –Bilder SIOP-LGG 2004

Frau
PD Dr. med. Monika Warmuth-Metz

Referenzzentrum HIT-LGG

Universitätskliniken Würzburg
Abteilung für Neuroradiologie
Josef-Schneider-Str. 11
97080 Würzburg
Telefon (0931) 201 34799 / 34626
Telefax (0931) 201 34685
E-Mail: hit@neuroradiologie.uni-wuerzburg.de

Früh-postoperative Untersuchung

Die schattierten Felder sind vom Einsender auszufüllen

Name		Histologie
Vorname		
Geburtsdatum	❐ männlich ❐ weiblich	Alle Bilder versandt? ❐ ja ❐ nein
Bisherige Therapie:		

Op – Datum: I__I__I . I__I__I . I__I__I__I__I

Kranielles **❐ MRT oder ❐ CT** ❐ nativ ❐ mit Kontrast ❐ ohne und mit Kontrast
Spinales **❐ MRT oder ❐ CT** ❐ nativ ❐ mit Kontrast ❐ ohne und mit Kontrast

Untersuchungsdatum vom: I__I__I . I__I__I . I__I__I__I__I

**Tumorrest**	❐ nein	❐ Ring	❐ <1,5 cm	❐ > 1,5 cm	❐ Inf. HS	❐ S4
**Größe a/c/s**	cm x cm x cm		cm^2		cm^3	
**Zystengröße**	cm x cm x cm		cm^2		cm^3	
**Hydrocephalus**	❐ Nein	❐ leichtgradig	❐ mittelgradig	❐ schwergradig	❐ Shunt	
**KM-Enhancement**	❐ ja	❐ nein				
**Anreichernder Tumoranteil**	❐ homogen	❐ überwiegend homogen	❐ überwiegend inhomogen	❐ inhomogen		
	❐ 0-25%	❐ 26-50%	❐ 51-75%	❐ 76-100%		
**Meningeose**	❐ nein	❐ fraglich	❐ M2a	❐ M2b	❐ M3a	❐ M3b
**Metastase wo:**						

**Resttumorstaging**				
❐ R0	❐ R1	❐ R2	❐ R3	❐ R4
❐ M0	❐ M2	❐ M3		

Freitext:

....
Datum Unterschrift / Stempel

22.10.2.3. Begleitschein für MRT/CT –Bilder SIOP-LGG 2004

Frau
PD Dr. med. Monika Warmuth-Metz

Referenzzentrum HIT-LGG

Universitätskliniken Würzburg
Abteilung für Neuroradiologie
Josef-Schneider-Str. 11
97080 Würzburg
Telefon (0931) 201 34799 / 34626
Telefax (0931) 201 34685
E-Mail: hit@neuroradiologie.uni-wuerzburg.de

Verlaufsuntersuchung

Die schattierten Felder sind vom Einsender auszufüllen

Name		Histologie
Vorname		
Geburtsdatum	❐ männlich ❐ weiblich	Alle Bilder versandt? ❐ ja ❐ nein
Bisherige Therapie:		

Kranielles **❐ MRT oder ❐ CT** ❐ nativ ❐ mit Kontrast ❐ ohne und mit Kontrast
Spinales **❐ MRT oder ❐ CT** ❐ nativ ❐ mit Kontrast ❐ ohne und mit Kontrast

Untersuchungsdatum vom I__I__I . I__I__I . I__I__I__I__I

**Tumorlokalisation**						
**Ausdehnung**						
**Größe a/c/s**	cm x cm x cm		cm^2		cm^3	
**Zystengröße**	cm x cm x cm		cm^2		cm^3	
**Rest/Rezidivtumor**	❐ ja	❐ fraglich	❐ nein			
**Begrenzung**	❐ scharf (≥ 90%)		❐ mäßig scharf (≥ 50%)		❐ unscharf < 50%)	
**Ödem**	❐ zunehmend		❐ gleich		❐ abnehmend	
**Zysten**	❐ zunehmend		❐ gleich		❐ abnehmend	
**Hydrocephalus**	❐ zunehmend	❐ gleich	❐ abnehmend		❐ Shunt	
**KM-Enhancement**	❐ zunehmend	❐ gleich	❐ abnehmend		❐ kein	
**Meningeose**	❐ nein	❐ fraglich	❐ M2a	❐ M2b	❐ M3a	❐ M3b
**Metastase wo:**						

**Staging**					
❐ CR	❐ PR > 50%	❐ IMP 50-25%	❐ SD < 25%	❐ PD > 25% oder neu	❐ unbestimmt
❐ M0	❐ M2	❐ M3			

**Freitext**:

....
Datum Unterschrift / Stempel

22.11. Toxizitätsklassifikation gemäß CTC

SIOP-LGG 2004
Seite 1/3

Hämatologie

Grad	0	1	2	3	4
**Hämoglobin (g/l)**	Altersnorm	>100	100 – 80	79 – 65	< 65
**Leukozyten (G/l)**	≥ 4,0	< 4,0 – 3,0	2,9 – 2,0	1,9 – 1,0	< 1,0
**Granulozyten (G/l)**	≥ 2,0	< 2,0 – 1,5	1,4 – 1,0	0,9 – 0,5	< 0,5
**Thrombozyten (G/l)**	≥ 100	< 100 - 75	74 – 50	49 – 10	< 10

Audiologie

Grad	0	1	2	3	4
**Hörvermögen**	normal	nur audiometrischer Hörverlust	Tinnitus, Hörverlust, keine Hörgeräte notwendig	Tinnitus, oder Hörverlust, mit Hörgeräten korrigierbar	nicht korrigierbare Ertaubung
**Beidseitiger Hörverlust (Brock et al. 1991)**	< 40 dB in allen Frequenzen	> 40 dB bei 8000 Hz	> 40 dB bei 4000 Hz	> 40 dB bei 2000 Hz	> 40 dB bei 1000 Hz

Neurologie

Grad	0	1	2	3	4
**Neurotoxizität, kortical**	normal	Vorübergehende Lethargie	moderate Somnolenz oder Agitation	zusätzlich Konfusion, Desorientiertheit, Halluzination	Koma, Anfälle, toxische Psychose
**Neuropathie, Motorik**	normal	subjekt. Schwäche, kein objektiver Befund	milde objektive Schwäche	objektive Schwäche mit Funktionsstörung	Paralyse
**Neuropathie,Sensorik**	normal	milde Parästhesien, Verlust tiefer Sehnenreflexe	moderate Parästhesien, objekt. Sensorische Funktionsstörung ohne Beeinträchtigung im Alltag	schwerer objekt. sensorischer Verlust m. Funktionsstörung mit Beeinträchtigung im Alltag	bleibender sensorischer Verlust m. Funktionsstörung
**Krampfanfälle**	keine	-	Krampfanfälle mit erhaltenem Bewusstsein	Krampfanfälle mit gestörtem Bewusstsein	Prolongierte Anfälle (z. B. Status epilepticus)
**Abdominale Schmerzen oder Krämpfe**	keine	Milde Schmerzen ohne Funktionsbeeinträchtigung	Mäßige Schmerzen: Schmerzen oder Analgetika beeinträchtigen die Funktion, aber nicht den Alltag	Schwere Schmerzen: schwerwiegende Beeinträchtigung im Alltag durch Schmerzen oder Analgetika	Schwerste Einschränkung

Infektiologie

Grad	0	1	2	3	4
**Infektion**	keine	leichte	Mäßig schwere, lokale oder orale Behandlung	schwere, systemische Infektion, i.v. antibiot./antimykot. Therapie	lebensbedrohliche Sepsis, Schock
**Fieber (ohne Neutropenie < 1,0 x 10^9/L)**	keines	38,0 - 39,0°C	39,1 - 40,0°C	>40,0°C für < 24 Std.	>40,0°C für > 24 Std. oder mit Hypotonie

2/3

Nephrologie

Grad	0	1	2	3	4
**Hämaturie**	keine	nur mikroskopisch	Makrohämaturie ohne Koagel	Anhaltende Makrohämaturie oder Koagel, die Katheterisierung, oder Transfusion erforderlich machen können.	Nekrosen oder tiefe Ulzeration, Transfusion erforderlich
**Kreatinin (x N)**	Altersnorm	< 1,5 x N	1,5 - 3,0 x N	3,0 - 6,0 x N	> 6,0 x N
**Proteinurie (Stix oder g/24 h)[A]**	normal oder <0,15 g/24h	1+ oder 0,15 - 1,0 g/24h	2+ bis 3+ oder 1,0 - 3,5 g/24h	4+ oder >3,5 g/24h	Nephrotisches Syndrom
**Glomeruläre Filtrationsrate (GFR) (ml / min / 1,73 m²)**	≥ 90	60 - 89	40 - 59	20 - 39	≤ 19

Übelkeit / Erbrechen

Grad	0	1	2	3	4
**Übelkeit**	keine	Fast normale Nahrungsaufnahme möglich	deutlich verminderte Nahrungsaufnahme	keine nennenswerte Nahrungsaufnahme	-
**Erbrechen (Anzahl der Episoden/24 Std.)**	0	1 Episode / 24 h	2-5 Episoden / 24 h	6-10 Episoden / 24 h	>10 Episoden/24 h parenterale Ernährung nötig

Allgemeinsymptome

Grad	0	1	2	3	4
**Gewichtsverlust**	< 5%	5 - <10%	10 - <20%	≥ 20%	-
**Alopezie**	normal	milder Haarausfall	Ausgeprägter oder kompletter Haarausfall	-	-
**Müdigkeit (Fatigue)**	keine	vermehrte Müdigkeit, aber keine Einschränkung altersgemäßer Aktivitäten	Mäßig (zB Rückgang um 1 ECOG-Stufe oder von 20 % im Karnofsky oder Lansky Index), verursacht Schwierigkeiten bei der Durchführung von Aktivitäten	Schwer (zB. Rückgang um ≥ 2 ECOG Stufen oder Verlust der Fähigkeit Aktivitäten durchzuführen)	Bettlägerig, schwere Einschränkung
**Essverhalten**	normal	Appetitverlust	orale Nahrungsaufnahme signifikant reduziert	iv-Flüssigkeitszufuhr erforderlich	Ernährung über nasogastrale Sonde oder parenteral

**Allergische Reaktion / Überempfindlichkeits reaktion**	keine	Flüchtiges Exanthem, vorübergehendes Fieber < 38°C	Urtikaria, Fieber ≥ 38°C, asymptomatischer Bronchospasmus	Symptomat., iv-therapiebedürftiger Bronchospasmus, Angioödem	Anaphylaxie

Gastroenterologie

Grad	0	1	2	3	4
**Mukositis**	keine	Erythem der	ungleichmäßige	konfluierende	Nekrosen oder

		Mukosa	pseudomembranöse Läsionen (≤ 1,5 cm Durchmesser, nicht konfluierend)	pseudomembranöse Läsionen (≥ 1,5 cm Durchmesser)	tiefe Ulzerationen; Blutungsneigung ohne mechanische Einwirkung
**STOMATITIS/ Pharyngitis**	keine	schmerzlose Ulzera, Erythem	schmerzhaftes Erythem, Ulzera, Nahrungsaufnahme möglich	schmerzhaftes Erythem, Ulzera, i.v.-Substitution erforderlich	schwere Ulzera, parenterale Ernährung erforderlich
**Diarrhoe (Anstieg der Stuhlfrequenz)**	keine	2-3 Stühle/Tag (mehr als vor therapie)	4-6 Stühle / Tag oder nächtliche Stuhlentleerungen	Anstieg auf ≥7 Stühle/Tag oder Inkontinenz oder parenterale Flüssigkeit wegen Dehydration	≥ 10 Stühle/Tag, blutige Durchfälle oder Kreislaufkollaps, physiologische Auswirkungen erfordern Intensivbehandlung
**Obstipation**	keine	geringe Obstipation, diät. Anpassungen erforderlich	Mäßige Obstipation, Laxantien erforderlich	Starke Obstipation, beginnender Subileus, Einläufe erforderlich	Obstruktion oder toxisches Megacolon, Ileus > 96 Stunden

Dermatologie

**Grad**	**0**	**1**	**2**	**3**	**4**
**Radiologische Dermatitis**	keine	schwaches Erythem oder trockene Desquamation	Mäßiges bis deutliches Erythem oder fleckige, feuchte Desquamation, beschränkt auf Hautfalten, mäßkges Ödem	konfluierende feuchte Desquamation, ≥1,5 cm Durchmesser, nicht auf Hautfalten begrenzt, deutliches Ödem	Exfoliative Dermatitis, Hautnekrosen oder Ulzerationen bis in die Tiefe der Dermis, Blutungsneigung ohne signifikantes Trauma oder Abschürfung

Leber

**Grad**	**0**	**1**	**2**	**3**	**4**
**Bilirubin** (x N)	Altersnorm	≤ 1,5	>1,5 – 3,0	> 3,0 – 10,0	> 10,0
**SGOT/SGPT** (x N)	Altersnorm	≤ 2,5	> 2,5 - 5,0	> 5,0 - 20,0	> 20,0

Pulmonologie

**Grad**	**0**	**1**	**2**	**3**	**4**
**Dyspnoe**	keine	Keine Symptome, patholog. Lungenfunktions -test	Dyspnoe unter starker Belastung	Dyspnoe unter normaler Belastung	Ruhedyspoe oder mechanische Beatmung notwendig

Kardiologie

**Grad**	**0**	**1**	**2**	**3**	**4**
**Kardiomyopathie (linksventrikuläre Funktion)**	normal	Asymptomatischer Rückgang der Ejektionsfraktion in Ruhe um ≥10%, aber <20% des Ausgangswertes	Asymptomatischer Rückgang der Ejektionsfraktion in Ruhe um ≥20% des Ausgangswertes	Milde Kardiomyopathie/Herzinsuffizienz medikamentös kompensiert	Schwere oder refraktäre Kardiomyopathie/ Herzinsuffizienz
**LV-FS-Ratio Echokardiographie**	> 30%	26% - 30%	21% - 25%	16% - 20%	< 16%

Cancer Therapy Evaluation Program, Common Toxicity Criteria, Version 2,0, Erscheinungsdatum: 30. April 1999
N: Altersnormwert, die Anpassungen für pädiatrische Patienten sind zu beachten. A: bei Diskrepanzen zwischen den Ablesungen am Urin-Stix und den Absolutwerten, gelten die Absolutwerte für die Gradierung.

22.12. Meldung schwerwiegender, unerwünschter Ereignisse (SAE) SIOP – LGG 2004

Studienleitung: **Frau Dr. Astrid K. Gnekow**, I. Klinik f. Kinder u. Jugendliche, Klinikum Augsburg, Stenglinstr. 2, 86156 Augsburg, Tel.: 0049 - (0) 821 - 400 - 3615, Fax: -3616, Email: gnekow.hit-lgg@klinikum-augsburg.de

Name, Vorname Pat.-Nr. Klinik Pat.-Nr.
I____________________________________I I_I_I_I_I I_I_I_I_I I__I I__I__I__I__I__I__I I_I__I__I__I__I
GPOH-PID: I I I I I I I I I Geb. Datum

Schwerwiegende, unerwartete Ereignisse während der Behandlung müssten sofort durch die behandelnde Klinik gemeldet werden, spätestens jedoch am nächsten Arbeitstag.

FAX-Nummer: 0049 – (0)821 – 400 3616

Gründe für die Meldung des SAE:

- Therapie-assoziierter Todesfall (⇒ bitte Dokumentationsbogen „22.13.2. Pat.Status" ausfüllen) ❒ ja ❒ nein
- Bleibende Schäden/Behinderung als Folge des Ereignisses ❒ ja ❒ nein
- Lebensbedrohliches Ereignis ❒ ja ❒ nein
- Unerwartete, schwere Nebenwirkung, die nicht auf dem Toxizitätsbogen dokumentiert werden kann ❒ ja ❒ nein
- WHO Grad 4 Toxizität: Leber, Herz, Niere, Haut, Nervensystem ❒ ja ❒ nein

Toxizitätsgrad des SAE nach NCI-CTC: ❒ 1 ❒ 2 ❒ 3 ❒ 4 ❒ unbekannt /nicht einzuordnen

Beginn bzw. Nachweis des SAE am I__I__I . I__I__I . I__I__I__I__I

Während / nach welchem Therapieelement?

❒ Induktion I ❒ Induktion II ❒ Konsolidierung ❒ nach Therapieende ❒ Radiotherapie ❒ Sonstige_______________

Bitte beschreiben Sie das Ereignis und die betroffenen Maßnahmen:
(Symptome, Lokalisation, Labor-Befunde, Diagnostik, Dauer, Therapie und Verlauf; falls nötig bitte zusätzliches Blatt beilegen)

____

____

Medikation bei Auftreten des SAE:

	Medikament	Tagesdosis	Applikation	Therapietage (von / bis)	Beziehung zwischen Medikament und SAE					
					keine	unwahr-scheinlich	möglich	wahr-schein-lich	sicher	nicht ent-scheidbar, unzureichende Daten
1.					❒	❒	❒	❒	❒	❒
2.					❒	❒	❒	❒	❒	❒
3.					❒	❒	❒	❒	❒	❒
4.					❒	❒	❒	❒	❒	❒
5.					❒	❒	❒	❒	❒	❒
6.					❒	❒	❒	❒	❒	❒

- Ist eine Beziehung zwischen dem SAE und der Medikation, die vor Auftreten des SAEs verabreicht wurde, denkbar? ❒ nein ❒ ja,_____________________
- Wurden ein oder mehrere Medikamente abgesetzt? ❒ nein ❒ ja, Nr.__________________
- Klang die Reaktion nach Absetzen des/der Medikamente(s) ab? ❒ nein ❒ ja, Nr.__________________
- Wurden ein oder mehrere Medikamente wieder angesetzt? ❒ nein ❒ ja, Nr.__________________
- Erschien die Reaktion nach der Wiedereinführung erneut? ❒ nein ❒ ja, Nr.__________________
- Wurden ein oder mehrere Dosierungen geändert? ❒ nein ❒ ja, Nr.__________________

Was bedingte Ihrer Ansicht nach das Auftreten des SAE?

❒ Komplikation des Tumors ❒ andere, begleitende Erkrankung
❒ Komplikationen der Therapie nach SIOP-LGG 2004 ❒ andere, begleitende Therapie
❒ andere bekannte oder mögliche Gründe; bitte erläutern:________________________________

Verlauf

❒ (noch) anhaltendes SAE ❒ Genesung ohne Nachwirkungen ❒ Genesung mit Nachwirkungen
❒ Tod durch SAE ❒ Tod, nicht in Verbindung mit dem SAE
Datum der Genesung oder des Todes: I__I__I.I__I__I.I__I__I <u>oder</u> ❒ nicht anwendbar (noch anhaltend)
Bemerkung:____

Datum Unterschrift/Stempel des dokumentierenden Arztes Name in Druckbuchstaben

22.13.1. Verlaufsdiagnostik SIOP LGG 2004

Nach Eingang von Melde- und Ersterhebungsbogen sowie gegebenenfalls der Therapiedokumentation werden die Dokumentationsbögen für das Langzeit-follow-up von der Studienleitung zu den geplanten Erhebungszeitpunkten zugeschickt.

I. Untersuchungsprogramm für Kinder, die im Rahmen der randomisierten Studie Chemotherapie erhalten (Mindestprogramm für die zentrale Datenerfasung):

	**Diagnose**	**Bei Therapie-beginn Zeit: 0**	**Nach Therapiebeginn**			**Nach Therapie-ende (siehe III)**
			**6 Monate**	**12 Monate**	**18 Monate**	
Tumorstatus	✔	✔	✔	✔	✔	✔
Bildgeb. Diagnostik	✔	✔	✔	✔	✔	✔
Ophthalmol. Diagnostik	✔	✔	✔	✔	✔	✔
klinische Symptome / Neurostatus	✔	✔	✔	✔	✔	✔
Indikation z. Therapie-beginn	---	✔	---	---	---	---
Dokument. d. Toxizität	---	---	✔	✔	✔	---
Neuropsych. Befund	✔	✔	---	---	✔	✔
Funktion Verhalten LQ	✔	✔	---	---	✔	✔

II. Empfohlene Untersuchungsfrequenz für die ophthalmologische Diagnostik während Therapie und Nachsorge für alle Kinder, besonders mit chiasmatisch-hypothalamischen Tumoren (Lorenz 2002)

Bei Diagnose				
Operation	vor	nach	2 Wochen danach	jedem chirurgischen Eingriff
Chemotherapie	vor	3-monatlich		während Chemotherapie
Radiotherapie	vor	nach	2 Wochen danach	
Nachsorge	1. Jahr	3-monatlich		
	2. Jahr	3-6-monatlich		häufiger, falls erforderlich
	3. Jahr	6-monatlich		häufiger, falls erforderlich
	4. Jahr und später	6-monatlich bis jährlich		häufiger, falls erforderlich

III. Nachsorgeuntersuchungsplan für Beobachtungspatienten und für Kinder nach Ende von Chemo- oder Radiotherapie

(Für die Dokumentation gegenüber der Studienleitung, die in Abhängigkeit von Verlauf und Befunden in größeren Intervallen erforderlich ist, sollten die jeweils angegebenen Bögen verwendet werden).

	**1., 2. und 3. Jahr**	**4. und 5. Jahr**	**6. bis 10. Jahr**
Körperliche und neurologische Untersuchung einschl. anthropometrischer Daten<br>A: (Bogen 22.13.7.und 8.)<br>B: (Bogen 22.13.7.)	Alle 3 Monate	Alle 6 Monate	Jährlich
Ophthalmologische Untersuchung<br>(Bogen 22.13.6.)	Alle 6 Monate. Häufiger, wenn erforderlich	Alle 6 Monate	Jährlich, jedoch halbjährlich bei Sehbahngliomen
Craniales und spinales (wenn erforderlich) MRT ohne und mit KM<br>(Bogen 22.10.2.-3.)	Alle 6 Monate	Alle 6 Monate	Jährlich
Audiogramm<br>Reinton Audiogr. wenn möglich ab dem Alter von 3 Jahren, sonst Freifeld Audiogr. oder oto-akustische Emissionen<br>(Bogen 22.13.8.)	Alle 6 Monate	Nicht erforderlich, wenn zuvor mehrfach normal	---
Glomeruläre Filtrationsrate (GFR)	6 Monate nach Chemotherapie, danach jährlich, falls nicht anders erforderlich	Nicht erforderlich, wenn zuvor mehrfach normal	---
Funktion, Verhalten, LQ<br>(Bogen 22.13.8.)	Jährlich	Jährlich	Bei Bedarf und bei Erreichen des 20. Lebensjahres
Endokrine Untersuchungen und (falls indiziert) Bestimmung des Knochenalters und hypothalamo-hypophysäre Funktionstests<br>(Bogen 22.13.5. und Bogen 22.13.8.)	Jährlich, wenn nicht anders erforderlich	Je nach den Erfordernissen von Wachstumsverlauf und Pubertätsentwicklung und vorausgegangener Chemo- oder Radiotherapie	Je nach den Erfordernissen von Wachstumsverlauf und Pubertätsentwicklung und vorausgegangener Chemo- oder Radiotherapie

A: für Kinder mit Tumoren der Supratentoriellen Mittellinie
B: für Kinder mit Tumoren aller anderen Lokalisationen

22.13.2. Tumoren im Kindesalter - Kooperative Dokumentation

In Zusammenarbeit mit dem Deutschen Kinderkrebsregister am IMBEI, 55101 Mainz,
Tel. 06131/17-3227, Fax 06131/17-4462

SIOP - LGG 2004 – Statusabfrage (1/2)

Studienleitung: **Frau Dr. Astrid K. Gnekow**, I. Klinik f. Kinder u. Jugendliche, Klinikum Augsburg, Stenglinstr. 2, 86156 Augsburg, Tel. 0821/400-3615, Fax :-3616, Email gnekow.hit-lgg@klinikum-augsburg.de

Name, Vorname Pat.-Nr. Klinik Pat.-Identifikationszahl

I____I I_I_I_I_I I_I_I_I_I I__I I__I__I__I__I__I__I__I I__I__I__I__I__I__I

GPOH-PID: I__I__I__I__I__I__I__I__I__I Geb. Datum

Letzte Information in der Studienzentrale vom:<br><br>➢	**Datum der letzten Untersuchung in der betreuenden Klinik:**<br>I__I__I . I__I__I . I__I__I__I__I<br><br>**Datum der letzten bildgebenden Kontrolle:** I__I__I . I__I__I . I__I__I__I__I<br>❐ MRT ❐ mit KM ❐ ohne KM<br>❐ CT ❐ mit KM ❐ ohne KM<br>(Bitte Befund beilegen)<br><br>**Tumorgröße:** I__I__I cm x I__I__I cm x I__I__I cm
Zuletzt dokumentierter Status des Patienten:<br><br>➢	**Aktueller Zustand des Patienten**:<br><br>❐ Vollremission/ tumorfrei **(CR)** ❐ Stillstand/Resttumor ohne Größenänderung **(SD)**<br>❐ Resttumor in Regression **(PR)** ❐ Progression **(PD)**<br><br>**Auftreten von Rezidiv/Progression/Disseminierung:**<br><br>❐ Nein ❐ Ja, am I__I__I . I__I__I . I__I__I__I__I<br>❐ Primärtumor ❐ Fernmetastase, wo:_________________________
Bisherige histologische Diagnose:<br><br>➢	**Änderung der bisherigen histologischen Diagnose:**<br><br>❐ Nein ❐ Ja, Befund:____
Bisher bekannte Therapiemaßnahmen:<br><br>➢	**Neue spezifische Therapien?**<br>❐ **Nein** ❐ **Ja**<br>❐ **Operation** Datum: I__I__I . I__I__I . I__I__I__I__I<br>❐ Biopsie (❐ offen ❐ stereotaktisch)<br>❐ Resektion (❐ partiell ❐ subtotal ❐ total)<br>❐ Shunt (Art :_____________________________________)<br><br>❐ **Chemotherapie** Beginn: I__I__I . I__I__I . I__I__I__I__I<br>❐ Induktion I ❐ alternative Konsolidierung<br>❐ Induktion II<br>❐ andere____<br><br>❐ **Radiotherapie** Beginn: I__I__I . I__I__I . I__I__I__I__I<br>❐ externe Radiotherapie (Gesamtdosis ___ Gy, Einzeldosis ___ Gy)<br>❐ interstitielle Radiotherapie (❐ Jod, ❐ andere _________________)<br><br>**Schwerwiegende Komplikationen:**<br>❐ **Nein** ❐ **Ja**, aufgetreten ❐ während ❐ nach<br>❐ Operation<br>❐ Chemotherapie<br>❐ Induktionsphase ❐ Konsolidierungsphase<br>❐ Radiotherapie<br>welche (ggf. WHO-Grad): ____<br>____<br>____

22.13.2. Statusabfrage SIOP-LGG 2004-Studie

Seite 2/2

Signifikante Änderungen des Neurostatus im Vergleich zur letzten Dokumentation:

____

____

____

____

Bei Verstorbenen: Sterbedatum: I__I__I . I__I__I . I__I__I__I__I

Todesursache:
❐ bedingt durch Primärtumor ❐ andere Todesursache
❐ bedingt durch Rezidiv/ Metastase ❐ Tumorabhängigkeit nicht entscheidbar
❐ bedingt durch Therapie
Autopsie: ❐ Nein ❐ Ja

Liegt die letzte Untersuchung länger als 2 Jahre zurück: ❐ Nein ❐ Ja

Wenn ja, ist Ihnen das weiterbehandelnde Zentrum bekannt?
❐ Nein ❐ Ja: ....
....
....

Wenn ja, ist Ihnen der weiterbehandelnde Hausarzt bekannt?

❐ Nein ❐ Ja: ....
....
....

Bemerkungen:

____

____

____

____

____

________________________ ____

Datum Unterschrift / Stempel (des dokumentierenden Arztes)

22.13.2. Tumoren im Kindesalter – Kooperative Dokumentation

In Zusammenarbeit mit dem Deutschen Kinderkrebsregister am IMBEI, 55101 Mainz,
Tel. 06131/17-3227, Fax 06131/17-4462

SIOP-LGG 2004– Ereignismeldung 1/1

Studienleitung: **Frau Dr. Astrid K. Gnekow**, I. Klinik f. Kinder u. Jugendliche, Klinikum Augsburg, Stenglinstr. 2, 86156 Augsburg, Tel.: 0049 - (0) 821 - 400 - 3615, Fax: -3616 , Email: gnekow.hit-lgg@klinikum-augsburg.de

Name, Vorname I____________________________________I Pat.-Nr. I_I_I_I_I Klinik I__I__I__I__I Pat.-Identifikationszahl I__I I__I__I__I__I__I__I I__I__I__I__I__I

GPOH-PID: I__I__I__I__I__I__I__I__I__I Geb. Datum

Ereignis:

MRT vom I__I__I . I__I__I . I__I__I__I__I (Bitte Befund beilegen)

❒ **Progression des bekannten Tumors**
- ❒ Klinische Progression
- ❒ Radiologische Progression (>25%)
- ❒ Disseminierung_________________

❒ **Rezidiv des bekannten Tumors**

❒ **Zweittumor**

❒ **Tod** (Statusbogen 22.13.2. versenden)

Bisherige Therapie:

❒ Beobachtung („wait and see") ❒ Chemotherapie ❒ Radiotherapie

Procedere nach Tumorprogression / Rezidiv / Zweitmalignom:

❒ **Keine Therapie** (weiter „wait and see"-Strategie)

❒ **Chirurgische Intervention**

1.) Resektion am I__I__I . I__I__I . I__I__I__I__I

Ausmaß: ❒ komplett ❒ subtotal ❒ partiell ❒ Biopsie (❒ offen ❒ stereotaktisch)

2.) Shuntanlage am I_I_I . I_I_I .I_I_I_I_I Art des Shuntes__________________________________

❒ **Indikation zur nicht-chirurgischen Therapie**

⇨ Therapiebasisinformation (Addendum 22.5.2.) an Studienleitung!

Therapiebeginn am I__I__I . I__I__I . I__I__I__I__I

❒ **Chemotherapie** (Behandlungszentrum:___________________________________)

- ❒ SIOP LGG 2004 CR ⇨ Randomisation der Induktionstherapie (Addendum 22.6.1.)
- ❒ andere ____

❒ **Radiotherapie** (verantwortlicher Radiotherapeut:_________________________________)

- ❒ konventionelle Radiotherapie
- ❒ interstitielle Radiochirurgie
- ❒ sonstige_____________________

❒ **Sonstige Therapie**____

Bemerkungen (insbesondere bei Abweichungen von der Studienstrategie):
____
____
____

....

Datum Stempel Unterschrift (des dokumentierenden Arztes)

22.13.4. Befunderhebung bei Diagnose: SIOP - LGG 2004
Neurologie, Endokrinologie, Entwicklung Seite 1/3

Name, Vorname Pat.-Nr. Klinik ID.-Nr.
I__________________________________I I_I_I_I_I I_I_I_I_I I__I I__I__I__I__I__I__I__I I__I__I__I__I__I
GPOH-PID: I__I__I__I__I__I__I__I__I__I Geb. Datum

Klinik.. Land...

1.) Lagen vor der Tumorerkrankung des Kindes Hinweise darauf vor, dass das Kind eine Verzögerung der frühkindlichen Entwicklung aufwies oder gab es einen spezifischen Förderbedarf oder gab es irgendeine vorbestehende körperliche Einschränkung?

❒ Nein ❒ Ja Wenn ja, bitte erläutern:..
....

2.) Befund unmittelbar vor dem chir. Eingriff (nachträglich bewertet) oder vor dem Beginn einer Therapie? **Datum** I_I_I I_I_I I_I_I_I_I
T T M M J J J J

Bitte markieren Sie die am ehesten zutreffende Antwort:

	a) vor Resektion	b) Vor Behandlungsbeginn	c) Veränderung zwischen dem Befund vor und nach Resektion
Bewusstsein	**vollständig/eingeschränkt/nn***	**vollständig/eingeschränkt/nn**	**gleich/besser/schlechter/nn**
altersbezogene Sehfähigkeit	**normal/eingeschränkt/nn**	**normal/eingeschränkt/nn**	**gleich/besser/schlechter/nn**
Funktionsstörungen der Nerven III, IV, VI	**Ja / Nein / unbekannt**	**Ja / Nein / unbekannt**	**gleich/besser/schlechter/nn**
Schwäche der Gesichtsmuskulatur	**Ja / Nein / unbekannt**	**Ja / Nein / unbekannt**	**gleich/besser/schlechter/nn**
Schwäche der Nerven IX,X,XI	**Ja / Nein / unbekannt**	**Ja / Nein / unbekannt**	**gleich/besser/schlechter/nn**
armbetonte Ataxie	**Ja / Nein / unbekannt**	**Ja / Nein / unbekannt**	**gleich/besser/schlechter/nn**
Rumpfataxie	**Ja / Nein / unbekannt**	**Ja / Nein / unbekannt**	**gleich/besser/schlechter/nn**
Schwäche der Extremitäten	**Ja / Nein / unbekannt**	**Ja / Nein / unbekannt**	**gleich/besser/schlechter/nn**

Falls die Antwort bei der Frage nach Extremitätenschwäche “ja” war, bitte erläutern Sie (z. B. Hemiparese links):

Art der Schwäche vor Resektion: ____

Art der Schwäche nach Resektion:____

**Datum**	**Stempel**	**Unterschrift**

* nn = nicht bekannt

22.13.4 Befunderhebung bei Diagnose: SIOP - LGG 2004
Neurologie, Endokrinologie, Entwicklung Seite 2/3

Name, Vorname Pat.-Nr. Klinik ID-Nr.
I__________________________________I I_I_I_I_I I_I_I_I_I I__I I__I__I__I__I__I__I__I I__I__I__I__I__I__I
GPOH-PID: I__I__I__I__I__I__I__I__I__I Geb.Datum

Klinik... Land...……………

3.) Postoperative Komplikationen (bitte alles Zutreffende ankreuzen):
(Falls eine Operation erfolgte: postoperative Komplikationen innerhalb von 30 Tagen.)

Wiederaufnahme auf der Intensivstation	❒ Ja	❒ Nein	❒ unbekannt
Cerebellärer Mutismus	❒ Ja	❒ Nein	❒ unbekannt
Krampfanfälle	❒ Ja	❒ Nein	❒ unbekannt
intrakranielle Blutung	❒ Ja	❒ Nein	❒ unbekannt
shuntpflichtiger Hydrocephalus	❒ Ja	❒ Nein	❒ unbekannt
ZNS Infektion	❒ Ja	❒ Nein	❒ unbekannt
andere Infektion	❒ Ja	❒ Nein	❒ unbekannt
subkutanes Liquorkissen	❒ Ja	❒ Nein	❒ unbekannt

Anzahl der Tage zwischen Operation und vollständiger Wiedererlangung des Bewusstseins

(GCS >=15 ?)* ☐☐ Tage (* Glasgow Coma Scale)

Tage auf der Intensivstation ☐☐ Tage

4.) SDQ Fragebogen ausgefüllt? Eltern ❒ Ja ❒ Nein
Patient ❒ Ja ❒ Nein

5.) QoL Fragebogen ausgefüllt?
PEDQOL ❒ Ja ❒ Nein
Eltern ❒ Ja ❒ Nein
Patient ❒ Ja ❒ Nein
CHQ-PF 28 ❒ Ja ❒ Nein
❒

6.) Bisherige Entwicklung bei Schulkindern:

a) Erhielt er/sie spezielle Hilfe
- in seinem/ihrem letzten Schuljahr? ❒ **Ja** ❒ **Nein**

- in irgendeinem früheren Schuljahr? ❒ **Ja** ❒ **Nein**

b) Ist/War er/sie in einer Regelschule? ❒ **Ja** ❒ **Nein**

wenn nein, welcher Schultyp?…......................……………………….......................

c) Befindet sich der Patient noch ganz oder teilweise in Ausbildung? ❒ **Ja** ❒ **Nein**
wenn ja: Besuch einer Schule ❒ **Ja** ❒ **Nein**
Andere ❒ **Ja** ❒ **Nein**

wenn andere, bitte erläutern…………………………………..............................

7.) Audiometrie (siehe 22.8.4.) ❒ durchgeführt ❒ nicht durchgeführt
Ergebnis auf dem besten Ohr: ❒ Grad 0 ❒ Grad 1 ❒ Grad 2 ❒ Grad 3 ❒ Grad 4

Datum Stempel Unterschrift

22.13.4 Befunderhebung bei Diagnose Neurologie, Endokrinologie, Entwicklung

SIOP - LGG 2004 Seite 3/3

Name, Vorname Pat.-Nr. Klinik ID-Nr.
I___________________________________I I_I_I_I_I I_I_I_I_I I__I I__I__I__I__I__I__I I__I__I__I__I__I__I
GPOH-PID: I__I__I__I__I__I__I__I__I__I Geb. Datum

Klinik... Land...……………

8.) Wachstum und Pubertät

Größe . cm Datum: └┴┘ └┴┘ └┴┴┴┘
T T M M J J J J

Sitzhöhe . cm Gewicht . kg

Größe der Mutter . cm ❒ gemessen ❒ berichtet ❒ unbekannt

Größe des Vaters . cm ❒ gemessen ❒ berichtet ❒ unbekannt

❒ weiblich

Bruststadium (Tanner) *
1 **2 3 4 5** *(einkreisen)*
→pubertär

Menarche? ❒ Nein ❒ Ja

❒ männlich

Genitalstadium (Tanner) *
1 **2 3 4 5** *(einkreisen)*
→pubertär

Testes-Volumen (ml) *(einkreisen)*
Rechts 0/1 2 3 **4 6 8 10 12 15 20 25 30**
Links 0/1 2 3 **4 6 8 10 12 15 20 25 30**
→ pubertär

Letzte Periode └┴┘ └┴┘ └┴┴┴┘ (*für die Interpretation der Hormonspiegel*)
T T M M J J J J

** Pubertätsstadien nach Tanner werden beschrieben auf Blatt 22.13.5*

9.) Aktuelle Hormonwerte

fT4 . pmol/L (Labor-Normalwert . - . pmol/L)

TSH . mU/L

Patient > 8 Jahre:

FSH . iu/L : **LH** . iu/L Datum: └┴┘ └┴┘ └┴┴┴┘
T T M M J J J J

Östradiol . pmol/L **Testosteron** . nmol/L

Erfolgt irgendeine Hormonersatztherapie oder hormonale Kontrazeption? ❒ Ja ❒ Nein
falls ja, bitte Einzelheiten angeben: …………………………………………………........

Datum **Stempel** **Unterschrift**

22.13.5. Endokrinologie – Follow up SIOP - LGG 2004
Wachstum, Pubertät, Hormonbehandlung Seite 1/3

Studienleitung: **Frau Dr. Astrid K. Gnekow**, I. Klinik f. Kinder u. Jugendliche, Klinikum Augsburg, Stenglinstr. 2, 86156 Augsburg, Tel.: 0049 - (0) 821 - 400 - 3615, Fax: -3616 , Email: gnekow.hit-lgg@klinikum-augsburg.de

Kopieren und für jedes Jahr verwenden (bitte ankreuzen)
❒ Behandlungsende; ❒ 2 Jahre; ❒ 3 Jahre; ❒ 4 Jahre; ❒ 5 Jahre; ❒ __ Jahre nach Behandlung ; ❒ Alter 20 J.

Name, Vorname Pat.-Nr. Klinik ID-Nr.
I___________________________________I I_I_I_I_I I_I_I_I_I I__I I__I__I__I__I__I__I I__I__I__I__I__I__I
GPOH-PID: I__I__I__I__I__I__I__I__I Geb. Datum

Klinik.. Land..……………

1.) Auxiologie

Größe . cm Datum: └┴┘ └┴┘ └┴┴┴┘

Sitzhöhe . cm

Gewicht . kg Datum: └┴┘ └┴┘ └┴┴┴┘

2.) Pubertät

❒ weiblich

Bruststadium (Tanner)
1 **2 3 4 5** *(einkreisen)*
→ **pubertär**

Menarche?
❒ Nein
❒ Ja, während vergangenem Jahr

Menstuationszyklus regelmäßig?
❒ Nein ❒ Ja ❒ Unbekannt

❒ männlich

Genitalstadium (Tanner)
1 **2 3 4 5** *(einkreisen)*
→ **pubertär**

Testesvolumen (ml) *circle*
Rechts 0/1 2 3 **4 6 8 10 12 15 20 25 30**
Links 0/1 2 3 **4 6 8 10 12 15 20 25 30**
→**pubertär**

Mädchen: Brustdrüsenentwicklung:
B1: Präpuberal, keine palpablen Drüsen.
B2: Brustdrüse und Warzenhof leicht erhaben, Brustknospung.
B3: Brustdrüse größer als Warzenhof, Form wie Erwachsenenbrust.
B4: Drüse im Warzenhof hebt sich von der übrigen Brust ab.
B5: Vorwölbung im Warzenhof weicht in die runde Kontur der erwachsenen Brust.

Jungen: Genitalentwicklung:
G1: Präpuberal, Penis, skrotum und Testes entsprechen in Form und Größe der frühen Kindheit.
G2: Skrotum, Testes vergrößert, Skrotalhaut verändert, keine Veränderung des Penis.
G3: Wachstum von Skrotum und Testes (um 10 ml), Penis nimmt an Länge weniger an Umfang zu.
G4: Penislänge und Umfang haben zugenommen, deutliche Glans-Kontur, weiteres Wachstum von Skrotum und Testes
G5: Voll entwickeltes Genitale (normale Testesvolumina 20ml, 12-30 ml).
Die testikuläre Größe sollte als Volumen angegeben werden (Prader orchidometer)

22.13.5. Endokrinologie – Follow up Wachstum, Pubertät, Hormonbehandlung

SIOP - LGG 2004
Seite 2/3

Kopieren und für jedes Jahr verwenden (bitte ankreuzen):
❒ Behandlungsende; ❒ 2 Jahre; ❒ 3 Jahre; ❒ 4 Jahre; ❒ 5 Jahre; ❒ __ Jahre nach Behandlung ; ❒ Alter 20 J.

Name, Vorname Pat.-Nr. Klinik ID-Nr.
I___________________________________I I_I_I_I_I I_I_I_I_I I__I I__I__I__I__I__I__I I__I__I__I__I__I
GPOH-PID: I__I__I__I__I__I__I__I__I__I Geburtsdatum

Klinik.. Land..……………

3.) Hormonale Substitution seit der letzten Dokumentation? ❒ Nein ❒ Ja, siehe unten

a) L-Thyroxin ? ❒ Ja, Beginn └┴┘ └┴┴┴┘ Ende └┴┘ └┴┴┴┘
❒ fortlaufend M M J J J J M M J J J J

Vor Behandlung:

fT4 . /L (Labor-Normalwert . - .) **TSH** . mU/L

b) Wachstumshormone? ❒ Ja Beginn └┴┘ └┴┴┴┘ Ende └┴┘ └┴┴┴┘
❒ fortlaufend M M J J J J M M J J J J

Vor Therapiebeginn:

Größe . cm; **Sitzhöhe** . cm; **Gewicht** . kg

c) Hydrocortison? ❒ Ja Beginn └┴┘ └┴┴┴┘ Ende └┴┘ └┴┴┴┘
❒ fortlaufend M M J J J J MM J J J J

d) GnRH Analoga? ❒ Ja Beginn └┴┘ └┴┴┴┘ Ende └┴┘ └┴┴┴┘
(bei Pubertas praecox) ❒ fortlaufend M M J J J J M M J J J J

e) Sexualsteroide? ❒ Ja Beginn └┴┘ └┴┴┴┘ Ende └┴┘ └┴┴┴┘
(Bitte Einzelheiten) ❒ fortlaufend M M J J J J M M J J J J

f) Andere? ❒ Ja Beginn └┴┘ └┴┴┴┘ Ende └┴┘ └┴┴┴┘
(Bitte Einzelheiten) ❒ fortlaufend M M J J J J M M J J J J

Erläuterungen:

Datum Stempel Unterschrift

22.13.5. Endokrinologie – Follow up **SIOP - LGG 2004**
Wachstum, Pubertät und Hormonbehandlung Seite 3/3

Hormonbehandlung nach Therapie

Kopieren und für jedes Jahr verwenden (bitte ankreuzen):
❒ Behandlungsende; ❒ 2 Jahre; ❒ 3 Jahre; ❒ 4 Jahre; ❒ 5 Jahre; ❒ __ Jahre nach Behandlung ; ❒ Alter 20 J.

Name, Vorname Pat.-Nr. Klinik ID-Nr.
I___________________________________I I_I_I_I_I I_I_I_I_I I__I I__I__I__I__I__I__I I__I__I__I__I__I
GPOH-PID: I__I__I__I__I__I__I__I__I__I Geburtsdatum

Klinik... Land..……………

1.) Schilddrüsen-Hormonspiegel

fT4 . pmol/L (Labor-Normalwert . - . pmol/L)

TSH . mU/L Datum: └─┴─┘ └─┴─┘ └─┴─┴─┴─┘
T T M M J J J J

2.) Gonadotropine und Sexualsteroide (bei Patienten > 8 Jahre)

LH . IU/L Datum: └─┴─┘ └─┴─┘ └─┴─┴─┴─┘
T T M M J J J J

FSH . iu/L

Östradiol . pmol/L

Testosteron . nmol/L

Bei Mädchen jenseits der Menarche bitte den ersten Tag der letzten Menstruationsperiode zur Interpretation der Hormonspiegel angeben:

Datum: └─┴─┘ └─┴─┘ └─┴─┴─┴─┘
T T M M J J J J

_______________ _______________ _______________
Datum **Stempel** **Unterschrift**

22.13.6. Ophthalmologische Dokumentation SIOP - LGG 2004 Seite 1/1

Studienleitung: **Frau Dr. Astrid K. Gnekow**, I. Klinik f. Kinder u. Jugendliche, Klinikum Augsburg, Stenglinstr. 2, 86156 Augsburg, Tel.: 0049 - (0) 821 - 400 - 3615, Fax: -3616, Email: gnekow.hit-lgg@klinikum-augsburg.de

Name, Vorname Pat.-Nr. Klinik ID-Nr.

I________________________________I I_I_I_I_I I_I_I_I_I I__I I__I__I__I__I__I__I__I I__I__I__I__I__I__I

GPOH-PID: I I I I I I I I I Geb. Datum

Klinik... Land..

Untersuchungsdatum I___I___I . I___I___I . I___I___I___I___I

(Details siehe Protokollabschnitt 8.6.)

Aktueller Befund	Verlaufsbeurteilung*
☐ n.d. **Sehschärfe:** RA _____ LA _____ Methode: _______________<br>☐ n.d. **Refraktion:** RA _____ LA _____ in Mydriasis: ❑ ja ❑ nein	gleich / besser /schlechter
☐ n.d. **Gesichtsfeld** RA _____ LA _____ Grad ____<br>(Kopien beifügen) Methode: ______________________	gleich / besser /schlechter
☐ n.d. **Farbsehen** RA _____ LA _____ Grad ___<br>Methode:______________________<br>Welche Farbwahrnehmung ist vornehmlich verloren? I___I<br>(1 = Blau; 2 = Rot; 3 = Gelb; 4 = Grün)	gleich / besser /schlechter
☐ n.d. **Kontrastempfindlichkeit**: RA _____ LA _____ Grad ___<br>Methode: __________________	gleich / besser /schlechter
☐ n.d. **Augenmotilität** RA_______ LA________	gleich / besser /schlechter
☐ n.d. **Strabismus** RA_______ LA ________ alternans _________<br>(1 =keiner, 2 = convergent, 3 = divergent, 4 = vertikal)<br>**Schielwinkel:** Ferne ________ Nähe ________ (in Prismen-dpt)	gleich / besser /schlechter
☐ n.d. **Nystagmus:** ❑ vorhanden ❑ nicht vorhanden<br>Richtung: RA I__I LA I__I (1 horizontal, 2 vertikal, 3 rotatorisch)<br>Typ: RA I__I LA I__I (1Ruckn., 2Pendeln., 3Blickrichtungsn.)	gleich / besser /schlechter
☐ n.d **Pupillenreaktion:** RA ______ LA ______<br>**RAPD:** ❑ vorhanden ❑ nicht vorhanden	gleich / besser /schlechter
☐ n.d. **Fundoskopie** RA ________ LA _______<br>**Optikusatrophie:** I___I (1 vollständige, 2 *"Bow Tie"*, 3 keine)<br>(Falls möglich, **Foto**dokumentation der Papille und von sonstigen Abweichungen)	gleich / besser /schlechter
☐ n.d **VEP:** (Falls vorhanden, in Kopie beifügen)<br>Latenz: RA__________ LA __________<br>Amplitude: RA__________ LA __________	gleich / besser /schlechter
**Gesamtbeurteilung der Untersuchung** ❑ gleich ❑ besser ❑ schlechter	

______________ ______________________ ______________________

Datum Stempel Unterschrift

(RA = rechtes Auge, LA = linkes Auge, n. d. = nicht durchgeführt, RAPD = relativer Afferenzdefekt der Pupille

* modified January 2006

22.13.7. und 22.13.8. Verlaufsdiagnostik SIOP-LGG 2004
Neurologie, Spätfolgen und Entwicklung

Erhebung und Dokumentation des klinisch-neurologischen Befundes

Der klinisch-neurologische Befund von Kindern und Jugendlichen, die nach Diagnosestellung lediglich beobachtet werden, aber auch von Kindern und Jugendlichen während der Chemo- und Radiotherapie kann mit dem einseitigen Bogen 22.13.7. verfolgt werden. Die umfangreichere Dokumentation auf dem Bogen 22.13.8. sollte besonders im Langzeit-follow-up für die Kinder nach Chemo- und Radiotherapie genutzt werden,

22.13.7.
Zu markanten Zeitpunkten (Erstuntersuchung bei Diagnosestellung, vor OP, eine Woche nach OP, vor Entlassung, alle 6 Monate, vor Beginn und nach Abschluss von Chemo- oder Radiotherapie, usw.) sollte der klinisch-neurologische Befund des Kindes von einem neurologisch qualifizierten Pädiater erhoben und auf einem DIN-A4 Vordruck dokumentiert. Die auf diesem Statusbogen abgefragten Items geben ein vereinfachtes, aber repräsentatives Bild der neurologischen Situation des Kindes und berücksichtigen Psychopathologie, Hirndrucksymptome, cerebrale Anfälle, Hirnnervenläsionen, pyramidale, extrapyramidale, cerebelläre und neuroendokrinologische Symptome sowie den Verlauf seit der letzten Voruntersuchung. Mit Hilfe dieser Daten wird bei Studienabschluss eine Korrelation der neuroradiologischen und histologischen Merkmale sowie der Therapiemodalitäten mit den prägnanten neurologischen Symptomen bei Diagnosestellung und nach Abschluss der Therapie, also dem klinischen Verlauf, hergestellt werden können.

22.13.7. Tumoren im Kindesalter - Kooperative Dokumentation

In Zusammenarbeit mit dem Deutschen Kinderkrebsregister am IMBEI, 55101 Mainz,
Tel. 06131/17-3227, Fax 06131/17-4462

SIOP-LGG 2004 – Neurologischer Befund

Studienleitung HIT-LGG-Studie:
Frau Dr. Astrid K. Gnekow, I. Klinik f. Kinder u. Jugendliche, Klinikum Augsburg, Stenglinstr. 2, 86156 Augsburg, Tel. 0821/400-3615, Fax :-3616 , Email gnekow.hit-lgg@klinikum-augsburg.de

Name, Vorname Pat.-Nr. Klinik Pat.-Identifikationszahl
I____I I_I_I_I_I I_I_I_I_I I__I I__I__I__I__I__I__I I__I__I__I__I__I
GPOH-PID: I__I__I__I__I__I__I__I__I__I Geb. Datum

(bei * bitte erläutern)

Diagnose: ……………………………………………………………………………………………

Untersuchung am: I_I_I . I_I_I . I_I_I_I_I

Anlass:
□ Erstuntersuchung
□ praeoperativ
□ 1 Woche postoperativ
□ Entlassung
□ sonst:........................
□ vor Chemotherapie
□ nach Chemotherapie
□ vor Radiotherapie
□ nach Radiotheapie

Anamnese:
Seit letzter Voruntersuchung in I_I_I / I_I_I: AZ □ schlechter □ idem □ besser

Neue neurologische Symptome?	□ Nein	□ Ja:……………………………………........................*
Hirndrucksymptome?	□ Nein	□ Kopfschmerzen Erbrechen □...........................*
Cerebrale Anfälle?	□ Nein	□ Ja: □ fokal □ generalisiert
Anfall Folge des Tumors?	□ Nein	□ Ja □ Unklar
AE-Dauermedikation ?	□ Nein	□ Ja

Befund: □ Wach □ Schläfrig □ Komatös

Altersentsprechend orientiert ?	□ Nein	□ Ja ....
Gesichtsfeld-Defekt ?	□ Nein	□ Ja:..*
Neuropsychologische Störung ?	□ Nein	□ Ja:..*
Stauungspapille?	□ Nein	□ Ja ....
Visusminderung ?	□ Nein	□ Ja:..*
Hörminderung ?	□ Nein	□ Ja:..*
Andere Hirnnerven-Läsion ?	□ Nein	□ Ja: ....*
Paresen ?	□ Nein	□ Ja:..*
Koordinationsstörung ?	□ Nein	□ Ja:..*
Extrapyramidale Bewegungsstörung ?	□ Nein	□ Ja:..*
Sensibilitätsstörung ?	□ Nein	□ Ja:..*
Neuroendokrinologische Symptome ?	□ Nein	□ Ja:..*

Körperlänge I_I_I_I,I_I cm Gewicht I_I_I,I_I kg Kopfumfang I_I_I;I_I cm Sitzhöhe I_I_I;I_I cm

Sonstiges und kurze Zusammenfassung des neurologischen Syndroms:

....

....

....

....
Stempel Datum Name/Unterschrift

22.13.8. Nach Behandlungsende: Spätfolgen und Entwicklung

SIOP - LGG 2004
Seite 1/3

Studienleitung: **Frau Dr. Astrid K. Gnekow**, I. Klinik f. Kinder u. Jugendliche, Klinikum Augsburg, Stenglinstr. 2, 86156 Augsburg, Tel.: 0049 - (0) 821 - 400 - 3615, Fax: -3616,
Email: gnekow.hit-lgg@klinikum-augsburg.de

Kopieren und für jedes Jahr verwenden (bitte ankreuzen):
❒ Behandlungsende; ❒ 2 Jahre; ❒ 3 Jahre; ❒ 4 Jahre; ❒ 5 Jahre; ❒ __ Jahre nach Behandlung ; ❒ Alter 20 J.

Name, Vorname Pat.-Nr. Klinik ID-Nr.
I___________________________________I I_I_I_I_I I_I_I_I_I I__I I__I__I__I__I__I__I__I I__I__I__I__I__I__I
GPOH-PID: |__|__|__|__|__|__|__|__|__| Geburtsdatum

Klinik... Land...

Letztes Untersuchungsdatum |__|__| |__|__| |__|__|__|__|
T T M M J J J J

1.) Hat der Patient/die Patientin irgendwelche Einschränkungen oder fehlt die Fähigkeit,Beschäftigungen nachzugehen aufgrund von motorischen Einschränkungen, Hirnnervenparesen oder eingeschränkter Sehfähigkeit? ❒ Ja ❒ Nein

Falls ja, bitte angeben, welche der folgenden Einschränkungen vorliegt:

Eingeschränkte Sehfähigkeit	❒ Ja	❒ Nein	❒ unbekannt
Augenmuskelparesen	❒ Ja	❒ Nein	❒ unbekannt
Facialisschwäche	❒ Ja	❒ Nein	❒ unbekannt
Schwäche der Nerven IX, X, XI	❒ Ja	❒ Nein	❒ unbekannt
armbetone Ataxie	❒ Ja	❒ Nein	❒ unbekannt
Rumpfataxie	❒ Ja	❒ Nein	❒ unbekannt
Extremitätenspastizität/-schwäche	❒ Ja	❒ Nein	❒ unbekannt
krankhafte, unbeeinflussbare Bewegungen	❒ Ja	❒ Nein	❒ unbekannt
Zittern oder Tremor	❒ Ja	❒ Nein	❒ unbekannt

Falls Sie "ja" geantwortet haben bei Spastizität/Schwäche der Extremitäten, bitte erläutern Sie die Art der Auffälligkeit (z. B. linksseitige Hemiparese):
....

2.) Erhält der Patient eine antikonvulsive Therapie? ❒ Ja ❒ Nein ❒ unbekannt

**3.) FMH (Deutschland)**		**❒ durchgeführt**	**❒ nicht durchgeführt**
**4.) SDQ**	**Eltern:**	**❒ durchgeführt**	**❒ nicht durchgeführt**
	**Patient:**	**❒ durchgeführt**	**❒ nicht durchgeführt**
**5.) QLQ-30 (nur über 20 Jahre)**		**❒ durchgeführt**	**❒ nicht durchgeführt**
**6.) PEDQOL**	**Eltern**	**❒ durchgeführt**	**❒ nicht durchgeführt**
	**Patient:**	**❒ durchgeführt**	**❒ nicht durchgeführt**
**CHQ-PF28**		**❒ durchgeführt**	**❒ nicht durchgeführt**

_______________ _______________ _______________
Datum Stempel Unterschrift

22.13.8. Nach Behandlungsende: Spätfolgen und Entwicklung

SIOP - LGG 2004
Seite 2/3

Kopieren und für jedes Jahr verwenden (bitte ankreuzen):
❒ Behandlungsende; ❒ 2 Jahre; ❒ 3 Jahre; ❒ 4 Jahre; ❒ 5 Jahre; ❒ __ Jahre nach Behandlung ; ❒ Alter 20 J.

Name, Vorname Pat.-Nr. Klinik ID-Nr.
I___________________________________I I_I_I_I_I I_I_I_I_I I__I I__I__I__I__I__I__I__I I__I__I__I__I__I__I
GPOH-PID: I__I__I__I__I__I__I__I__I__I Geburtsdatum

Klinik.. Land...……………

7.) Audiometrie (in Anlehnung an die CTC Kriterien 22.11.)

Audiometrie (am besten Ohr) ❒ Grad 0 ❒ Grad 1 ❒ Grad 2 ❒ Grad 3 ❒ Grad 4

8.) Sehfähigkeit

❒ normal
❒ eingeschränkt (CTC Grad 3)
❒ leicht eingeschränkt (ohne Einfluss auf den Alltag
❒ blind

Falls eingeschränkt, bitte den Ophthalmologie-Dokumentationsbogen 22.13.6. ausfüllen

9.) Haut: Änderung der Pigmentation

❒ keine ❒ vorübergehend, leichte ❒ andauernde, ausgeprägte

10.) Schleimhäute (oral /pharyngeal)

❒ normal
❒ fleckige Atrophie oder Teleangiektasie
❒ diffuse Atrophie oder Teleangiektasie, oberflächliche Ulzeration
❒ tiefe Ulzeration ohne Exposition von Knochen oder Knorpel
❒ tiefe Ulzeration mit Exposition von Knochen oder Knorpel

11.) Dysphagie

❒ normal
❒ Probleme beim Essen fester Nahrung
❒ Probleme beim Essen weicher Nahrung
❒ kann nur Flüssigkeiten trinken
❒ vollständig unfähig zum Schlucken

Datum Stempel Unterschrift

22.13.8. Nach Behandlungsende Spätfolgen und Entwicklung

SIOP - LGG 2004
Seite 3/3

Kopieren und für jedes Jahr verwenden (bitte ankreuzen):
❒ Behandlungsende; ❒ 2 Jahre; ❒ 3 Jahre; ❒ 4 Jahre; ❒ 5 Jahre; ❒ __ Jahre nach Behandlung ; ❒ Alter 20 J.

Name, Vorname Pat.-Nr. Klinik ID-Nr.
I___________________________________I I_I_I_I_I I_I_I_I_I I__I I__I__I__I__I__I__I__I I__I__I__I__I__I

GPOH-PID: I__I__I__I__I__I__I__I__I__I Geburtsdatum

Klinik.. Land..……………

12) Ausbildungs- und Sozialinformationen:

a) Erhielt er/sie spezielle Hilfe

- in seinem/ihren letzten Schuljahr? ❒ Ja ❒ Nein

- in irgendeinem vorherigen Schuljahr? ❒ Ja ❒ Nein

b) Ist/war er/sie in einer Regelschule ? ❒ Ja ❒ Nein

falls nein, zu welcher Schulkategorie gehört die Schule?……………………………........................

c) Ist der Patient noch ganz oder teilweise in Ausbildung? ❒ Ja ❒ Nein

wenn ‚ja‘, er/sie ist auf welcher **Schule:...** ❒ Ja ❒ Nein

Andere: ❒ Ja ❒ Nein

wenn ‚andere“, bitte erläutern…………………………..……

d) Wenn der Patient/die Patientin keine Schule mehr besucht,

i) wie alt war er/sie, als er/sie die Schule verließ? ☐☐ Jahre

ii) wo lebt der Patient/die Patientin?

in der Familie / zu Hause? ❒ Ja ❒ Nein
in einem beschützten Wohnheim? ❒ Ja ❒ Nein
selbständig (vielleicht mit Freunden oder einem Partner)? ❒ Ja ❒ Nein
andere? ❒ Ja ❒ Nein
Wenn ‚andere‘, bitte erläutern ....

iii) ist er/sie:
in weiterer / höherer Ausbildung ❒ Ja ❒ Nein
Wenn ‚ja‘, bitte erläutern..
in Voll- oder Teilzeitbeschäftigung? ❒ Ja ❒ Nein
Wenn ‚ja‘, ist es ein normaler Beruf ohne speziell auf Behinderungen zugeschnitten zu sein? ❒ Ja ❒ Nein
Wenn ‘nein’, bitte erläutern...

Datum Stempel Unterschrift

22.13.9. Anhänge: Funktion, Lebensqualität, Verhalten SIOP - LGG 2004
22.13.9.1. Fertigkeitenskala FMH Seite 1/2

Name, Vorname Pat.-Nr. Klinik ID-Nr.
I__________________________________I I_I_I_I_I I_I_I_I_I I__I **I__I__I__I__I__I__I** I__I__I__I__I__I
GPOH-PID: I__I__I__I__I__I__I__I__I Geburtsdatum

Untersuchungsdatum: I__I__I . I__I__I . I__I__I__I__I

Geschlecht: ❐ männlich ❐ weiblich
Muttersprache: ❐ deutsch ❐ andere_________________
Liegt eine Krankheit oder Behinderung vor ? ❐ Ja ❐ Nein
Wenn „Ja“, welche ?____

Bitte zutreffendes ankreuzen:	**Ja**	**Nein**
**Fortbewegung:**		
kann beide Arme und Beine bewegen	❐	❐
kann sich vom Bauch auf den Rücken drehen	❐	❐
kann sich allein aufsetzen und sitzt frei	❐	❐
kann allein Rollstuhl fahren	❐	❐
kann mit Hilfe (Stock, Geländer) Treppen hinauf- und hinuntergehen	❐	❐
kann ohne Hilfsmittel gehen	❐	❐
geht frei eine Treppe hinauf und hinunter	❐	❐
kann ohne Stützräder Rad fahren	❐	❐
kann allein mit Bus oder Bahn fahren	❐	❐
hat einen Führerschein für Auto und/oder Motorrad	❐	❐
kann in einer fremden Großstadt Auto fahren	❐	❐
**Essen / Trinken:**		
kann Eßbares von nicht Eßbarem unterscheiden	❐	❐
kann allein aus einer Tasse/Becher trinken	❐	❐
kann allein mit einem Löffel essen	❐	❐
kann sich ein Brot schmieren	❐	❐
kann ein einfaches Gericht selbst kochen	❐	❐
achtet allein auf eine ausgeglichene Ernährung	❐	❐
**Körperpflege:**		
ist tags trocken	❐	❐
ist nachts trocken	❐	❐
kann sich selbst die Hände waschen	❐	❐
kann leicht zu schließende Knöpfe zu machen	❐	❐
kann alleine die Toilette benutzen	❐	❐
kann ohne fremde Hilfe baden oder duschen	❐	❐
zieht sich selbst vollständig an	❐	❐
kann sich selbst die Nägel schneiden	❐	❐

22.13.9.1. Fertigkeitenskala FMH

SIOP - LGG 2004
Seite 2/2

Name, Vorname Pat.-Nr. Klinik ID-Nr.
I__________________________________I I_I_I_I_I I_I_I_I_I I__I I__I__I__I__I__I__I I__I__I__I__I__I__I
GPOH-PID: I__I__I__I__I__I__I__I__I Geburtsdatum

Untersuchungsdatum: I__I__I . I__I__I . I__I__I__I__I

Bitte zutreffendes ankreuzen:	**Ja**	**Nein**
**Allgemeine Unabhängigkeit:**		
kann sehen	❒	❒
kann tags mindestens eine Stunde allein bleiben	❒	❒
kann bei geschlossener Tür ohne Licht schlafen	❒	❒
kennt die eigene Adresse	❒	❒
geht allein einkaufen	❒	❒
kann eine ganze Nacht allein bleiben	❒	❒
kann allein Behördengänge ausführen	❒	❒
wohnt unabhängig von Eltern/Erziehern oder Pflegekräften	❒	❒
verdient selbstständig Geld	❒	❒
leitet eine Abteilung oder ein Unternehmen	❒	❒
**Verständigung:**		
kann hören	❒	❒
versteht unmittelbar bevorstehende Ereignisse (z.B. „nach dem Essen gehen wir spazieren“)	❒	❒
sagt einzelne Worte	❒	❒
kann einfache Sätze bilden	❒	❒
spricht von sich selbst als „ich“	❒	❒
benutzt richtig Vergangenheit und Zukunft	❒	❒
ruft an und führt ein Telefongespräch	❒	❒
kann eine längere Geschichte erzählen	❒	❒
kann die Uhr lesen	❒	❒
kann mit Argumenten und Gegenargumenten diskutieren	❒	❒
kann eine Fremdsprache sprechen	❒	❒
**Schreiben/Lesen/Rechnen:**		
unterscheidet eins und viele	❒	❒
kann bis drei zählen	❒	❒
versteht Bildergeschichten	❒	❒
schreibt einfache Wörter ohne abzuschreiben	❒	❒
kann im Zahlenraum bis 100 rechnen	❒	❒
liest einfache Lesebücher	❒	❒
kann ein Datum richtig angeben	❒	❒
kann einen kurzen Brief schreiben	❒	❒
hat im letzten Jahr etwas für die Öffentlichkeit geschrieben (z.B. einen Aufsatz veröffentlicht)	❒	❒
kann Wahrscheinlichkeitsrechnungen durchführen	❒	❒

_____________________ _____________________ _____________________

Datum Stempel Unterschrift

22.14.1 Wissenschaftliche Begleitprojekte: „Pilocytische Astrocytome mit klinisch atypischem Verlauf – Histologisch atypische und maligne Formen“ SIOP-LGG 2004

Trotz guter histologischer Differenzierung unterscheiden sich einige pilocytische Astrocytome in ihrem klinischen Verhalten vom ansonsten „gutartigen“, rezidivfreien Verlauf durch eine rasche Rezidivierung oder auch Malignisierung. Faktoren, welche ein solches Verhalten induzieren, sind bislang nicht gut definiert. Vor allem konnten bislang keine eindeutigen histopathologischen Merkmale identifiziert werden, die eine Prognosestellung erlauben. Dazu könnten eine erhöhte Proliferationsaktivität oder Atypien, vor allem Nekrosen, bis hin zur verifizierten malignen Transformation zum anaplastischen Astrocytom gehören.

Daher sollen im Rahmen der SIOP-LGG-2004-Studie histologische Dignitätskriterien mit dem klinischen Verlauf der Patienten systematisch überprüft, präzisiert und, wenn möglich, durch weitere histomorphologische und immunhistochemische Parameter ergänzt werden. Ziel ist, distinkte histologische Subtypen mit differentem klinischen Verlauf abzugrenzen. Zu einem derartigen Subtyp könnten pilomyxoide Astrocytome WHO °I gehören (Tihan et al 1999). Besondere Zielgruppen der Untersuchung sind somit Kinder, bei denen pilocytische Astrocytome diagnostiziert wurden, die aber einen klinisch atypischen Verlauf nehmen.
Zu diesen gehören pilocytische Astrocytome, die nach vollständiger Resektion (neurochirurgische und neuroradiologische Bestätigung) oder nach subtotaler Resektion schon nach relativ kurzer Zeit rezidivieren. Darüber hinaus können in diese Gruppe Kinder gehören, bei denen das Rezidiv als anaplastisches pilocytisches Astrocytom auftritt. Zusätzliche Einflüsse bei diesen Patienten durch eine postoperative adjuvante Therapie sind zu berücksichtigen. Außerdem gehören Kinder, deren pilocytisches Astrocytom primär oder sekundär zur Disseminierung führt, in die Gruppe atypisch verlaufender pilocytischer Astrocytome.

Zudem soll überprüft werden, welchen Verlauf diejenigen niedrigmalignen Gliome nehmen, bei denen durch moderne immunhistochemische Marker eine erhöhte Proliferationsaktivität nachgewiesen werden kann. Die bisherige Literatur zeigt eine uneinheitliche Einschätzung der Bedeutung des Ki67/MIB-1-Index. Inwieweit die diskrepante Einschätzung der Höhe des Ki67/MIB-1-Index auch durch niedrige Patientenzahlen in den Studien hervorgerufen wird, muss gegenwärtig offenbleiben. An der vergleichsweise großen Patientenzahl der HIT-LGG-Studie soll nunmehr prospektiv die Bedeutung dieses Markers untersucht werden.

Hinsichtlich der Molekulargenetik pilocytischer Astrocytome liegen in der Literatur vergleichsweise noch wenige Daten über pathologische Alterationen vor. Wie die typischen Prädilektionsstellen Phakomatose-assoziierter Tumoren vermuten lassen, ist die molekulare Pathogenese pilocytischer Astrocytome in unterschiedlichen Tumorlokalisationen möglicherweise sehr verschieden. Bei ausreichendem Tumormaterial soll geklärt werden, ob sich molekular-neuropathologisch atypische und maligne Varianten abgrenzen lassen. Molekularpathologische Befunde sollen mit histomorphologischen Parametern, wie Proliferationsindex, Vaskularisierungsgrad, Nekrosetendenz etc., aber auch dem Wachstumsmuster der Lokalisation und dem klinischen Verlauf einschließlich Rezidivneigung und einer malignen Transformation korreliert werden.

Prof. Dr. T. Pietsch
Institut für Neuropathologie der Universität Bonn
Sigmund-Freud-Str. 25
53105 Bonn
Tel.: 0228-287 4398

22.14.2. Wissenschaftliche Begleitprojekte: SIOP-LGG 2004

Gesundheitsbezogene Lebensqualität bei Kindern und Jugendlichen , die mit SIOP-LGG 2004 behandelt werden

Gegenstand und Bedeutung

Kinder und Jugendliche mit Neubildungen im Bereich des Kopfes haben schon aufgrund der Tumorlage vielfältige Probleme. Die niedriggradigen Gliome bilden mit 30-40% die größte Erkrankungsgruppe. Bei dieser Tumorentität spielen vielfältige Variablen eine Rolle: Dies sind Tumorsitz, Resektabilität und Wachstumstendenz. Der Einfluß, der an diese Variablen angepassten Behandlung, die von einer beobachtetenden Haltung über eine reine operativen Behandlung, bis hin zur chemotherapeutischen und oder Strahlenbehandlung reicht auf die Lebensqualität der Patienten und auch die Auswirkung von krankheits- bzw. therapieassozierten Spätfolgen ist bisher nicht untersucht. Ebenso ist bisher ungeklärt, wie sich die Erkrankung und Behandlung auf die somatische und psychosoziale Entwicklung der Kinder und Jugendlichen auswirken und inwieweit Folgen sowohl von Krankheit wie auch der Therapie die Lebensqualität der Patienten in all ihren Facetten nachhaltig beeinflussen. Dazu gehören auch die soziale Reintegration und Rehabilitation in Schule und Beruf, wie auch die Auswirkungen auf ihre langfristige Lebensplanung. Die gesundheitsbezogene Lebensqualität ist als ein multidimensionales Konstrukt, das körperliche, emotionale, mentale, soziale und verhaltensbezogene Komponenten des Wohlbefindens und der Funktionsfähigkeit aus der Sicht des Patienten und/oder von Beobachtern definiert.

Die Erfassung der gesundheitsbezogenen Lebensqualität bei Kindern mit Krebserkrankung ist durch die Tatsache kompliziert, daß eine große Anzahl unterschiedlicher Faktoren berücksichtigt werden müssen. Kinderonkologische Erkrankungen erfassen eine große Altersspanne, bezogen auf die unterschiedlichen Erkrankungen. 60% aller Neuerkrankungen betreffen Kinder unter 8 Jahren. Es wird jedoch generell davon ausgegangen, daß Kinder unterhalb dieses Alters nur begrenzt aufgrund der fehlenden Sprachmöglichkeiten selbst Auskunft über ihre Lebensqualität geben können.

Dies bedeutet, das altersspezifische Meßinstrumente benötigt werden, und das für Kinder unterhalb eines bestimmten Alters Proxies (meist die Eltern) befragt werden müssen, wobei zwar durch die Eltern die physische Funktionalität des Kindes beurteilt werden kann, über die emotionale Funktionalität jedoch nur eine unzureichende Aussage gemacht werden kann.

Ziel

Ziel dieses Projektes soll es sein, mit Hilfe von Fragebögen sowohl von der Seite der Patienten als auch über die Eltern eine Einschätzung der Lebensqualität der betroffenen Kinder mit niedriggradig- malignen Gliomen, die im Rahmen der SIOP-LGG 2004 - Studie betreut werden zu befragen Dies sollen zu verschieden Zeitpunkten in der Behandlung erfolgen: bei Diagnose/vor OP, nach Chemo/Radiobehandlung innerhalb des Follow-up. Verglichen werden sollen die generierten Ergebnisse auch mit dokumentierten klinischen Veränderungen. Ein besonderes Augenmerk gilt dabei den Bereichen Sehen, Hören, Neurologie, Kognition, Endokrinologie.

Fragestellungen

Grundsätzlich sollen folgende Fragen in diesem Zusammenhang beantwortet werden:

- Wie wirkt sich die Erkrankung und die Behandlung von Patienten mit niedriggradigen Gliomen auf die Lebensqualität und auf die somatische und psychosoziale Entwicklung der Kinder und Jugendlichen aus?
- Wie korrelieren Sehvermögen, endokrinologischer und neurologischer Status mit der bewerteten Lebensqualität (besonders ausgerichtet auf die Kinder mit Gliomen im Bereich des Hypothalamus/Chiasma Opticus)
- Wie bewerten Kinder mit schweren neurologischen Ausfällen (z.B. bei spinalen Tumoren) ihre Lebensqualität)

- Gibt es Hinweise das bestimmte verfolgte Behandlungsoptionen von den Betroffenen positiver bewertet werden im Sinne der LQ als Andere bei gleicher Überlebenswahrscheinlichkeit.?

Eingesetzte Instrumentarien zur Lebensqualitätsevaluation

Im Deutschen Sprachraum soll ein Instrumentenset eingesetzt werden, das sowohl eine Befragung der Patienten als auch der Eltern ermöglicht (PEDQOL), zusätzlich soll noch für die Kinder und Elternbefragung ein Bogen zu Stärken und Schwächen des Kindes eingesetzt werden, der ein gute Ergänzung zu dem benutzten LQ- Instrument gibt (SDQ). Für die Vergleichbarkeit im internationalen Kontext wird der CHQ_PF 28 benutzt, der über alle Altersgruppen von den Eltern ausgefüllt wird.

Somit sind die eingesetzten Instrumente: **PEDQOL 8-18 Jahre in der Kinder- und Elternversion, PEDQOL 4-7 Jahre in der Kinderversion, SDQ in der Kinder- und Elternversion, CHQ-PF-28 als Elternfragebogen**

- **PEDQOL (Pediatric Quality of Life Questionnaire); deutsche Version G. Calaminus 1998, modifiziert 1999.**

Nach der Definition der gesundheitsbezogenen Lebensqualität sind die wesentlichen Dimensionen dieses Fragebogens die körperliche Verfassung, das psychische Befinden, die sozialen Beziehungen und die Funktionsfähigkeit im Alltagsleben, erfasst als Selbstbericht der Kinder und Jugendlichen in der Altersgruppe der 8 bis 18 Jährigen. Diesen Basisdimensionen wurde noch die Dimension „Autonomie“ hinzugefügt. Das eingesetzte krebsspezifische Instrument PEDQOL soll entsprechend den oben gemachten Ausführungen einem krankheitsspezifischen Messansatz eines patientenzentrierten Lebensqualitätskonzepts folgen. Der Fragebogen hat insgesamt 50 Items, die sich auf das Erleben und Verhalten der Kinder beziehen. Die Kinder werden gebeten nachzudenken, wie sie sich in der letzten Woche gefühlt haben, da dieser Zeitraum sich als optimale Referenz für den Selbstbericht von Erlebnisinhalten bzw. Funktionszuständen in der Evaluationsforschung etabliert hat. Als Antwortmöglichkeit auf die Frage wurde eine fünfstufige Likertskala im Häufigkeitsbereich gewählt, die nach jedem Statement eine Beantwortung zwischen „nie“, „selten“, „häufig“ oder „immer“ erfordert.
Das Instrument ist nach psychometrischen Gütekriterien entwickelt und bereits an gesunden und krebskranken Kindern auf Reliabilität, Komparabilität und Praktikabilität getestet und validiert.

- **Die Elternversion des Child Health Questionaire: Ursprungsversion von J. Landgraf und J. Ware 1993. Deutsche Version in der Kurzform CHQ-PF28 übersetzt und validiert von M. Bullinger und Mitarbeitern 1994.**

Bei dem CHQ handelt es sich um ein generisches Instrument zur Erfassung von Lebensqualität bei Kindern und Jugendlichen. Er ist angelegt als Selbstbefragungsinstrument ab einem Alter von 5;0-15;0 Jahren und liegt zur Fremdbefragung als Elternversion vor. Der Fragebogen besteht aus einer allgemeinen Gesundheitseinschätzung auf einer stufenlosen Skala zwischen „ausgezeichnet“ und „schlecht“ und bezieht sich auf körperliche Fähigkeiten des Kindes, Schularbeiten, Schmerzen, Beschwerden, Zurechtkommen mit anderen, allgemeines Wohlbefinden, Zufriedenheit mit verschiedenen Lebensbereichen und Einschätzung des Gesundheitszustandes. Zusätzlich kann die Mutter eine Einschätzung ihrer allgemeinen Gesundheit und der Wirkung ihres Kindes auf sie hinsichtlich ihrer Befindlichkeit und seines Gesundheitszustandes durchführen.

- **Der SDQ: Strength and Difficulties Questionaire: Von Goodman 1999 entwickelt.**

Es handelt sich um ein kurzes Instrument das Verhalten und Verhaltensauffälligkeiten bewertet. Das Instrument wird in vielfältiger Weise eingesetzt und ist auch Bestandteil der LQ- Evaluation in internationalen Pädiatrischen Hirntumorstudien (HIT/PNET IV)

Die benutzten Instrumente zur Erfassung der Lebensqualität umfassen die zu erwartende Alterspannbreite der Kinder mit niedrig-gradig malignen Gliomen. Ihre Sensitivität für Veränderungen im Zeitverlauf ist überprüft und berücksichtigt so die entwicklungsbedingten Veränderungen der Kinder. Die Instrumente sind an Populationen gesunder Kinder getestet und haben den notwendigen Hintergrund zum Vergleich der „Norm“ mit den im Projekt zu erfassenden Abweichungen bei den Patienten. Das krankheitsspezifische Instrument (PEDQOL) ergänzt Fragen, die sich spezifischer auf die Erkrankung der Kinder und deren Folgen beziehen. Aus den angewandten Instrumentarien soll anhand der aus dem Projekt gemachten Erfahrungen durch Skalenselektion ein weiter optimiertes Basisinstrumentarium entstehen, das für Kinder mit niedrigmalignen Gliomen spezifische Informationen zur LQ generieren hilft und das zu Korrelationsuntersuchungen mit Ergebnissen aus Messungen somatischer, wie auch intellektell/neurokognitiver Spätfolgen der krebskranken Kinder unter Therapie wie auch im Bereich der Nachsorge benutzt werden kann.

Frau Dr. G. Calaminus
Klinik für Päd. Onkologie/Hämatologie und Immunologie
Heinrich-Heine-Universität
Moorenstr. 5
40225 Düsseldorf
e-mail: pedqol@uni-duesseldorf.de
Tel.: 0211-811 9108/6567
Fax: 0211-811 6206

Eltern-Information für Begleitstudie

Gesundheitsbezogene Lebensqualität bei Kindern und Jugendlichen , die mit SIOP-LGG 2004 behandelt werden

Liebe Eltern,

Sie sind noch nicht lange in der Klinik und haben in dieser Zeit bereits viele stark belastende Informationen über die Erkrankung und die zukünftige Behandlung Ihres Kindes erhalten. Sicher ist es sehr schwierig, die Ängste und Sorgen, die damit verbunden sind, auszuhalten und damit umgehen zu lernen. Auch stehen für Sie sicher noch einige organisatorische Probleme an, die erst noch gelöst werden müssen.

Ihr Kind wird einer Behandlung eines niedrig-gradig malignen Glioms des Kopfes unterzogen. Die Erkrankung, ihre Behandlung und die Heilung werden unterschiedlich verarbeitet. Manche Eltern, deren Kind geheilt wurde, berichten uns, daß sich das Verhalten ihres Kindes nach der Behandlung verändert hat oder daß Schwierigkeiten beim Lernen in der Schule oder der Ausbildung aufgetreten sind. Diese Veränderungen müssen nicht vorhanden sein oder können so gering ausgeprägt sein, daß sie kaum auffallen. Auch wird vermutet, daß Erkrankung und Behandlung sich akut und auch langfristig auf die Lebensqualität Ihres Kindes auswirken können.

Wir möchten Ihr Kind und Sie daher zu– genau festgelegten - Zeitpunkten der Behandlung zurm Wohlbefinden (Lebensqualität) befragen, um solche Veränderungen möglichst früh zu erkennen. Diese Befragung wird nicht nur an dieser Klinik, sondern an verschiedenen Kliniken in ganz Deutschland und Österreich durchgeführt.

Ziel der Untersuchung ist es, in Zukunft die Behandlung für ein niedrigmalignes Gliom noch weiter zu verbessern und Erkenntnisse zu bekommen, die die Grundlage für eine gezielte Förderung darstellen können.
Eine Teilnahme an dieser Untersuchung könnte nicht nur für Ihr Kind von Vorteil sein, sondern auch Kindern, die in Zukunft an einem niedriggradig-malignen Gliom, helfen. Deshalb bitten wir Sie um Ihre Zustimmung und Mithilfe bei den vorgeschlagenen Untersuchungen.

Die Resultate dieser Untersuchungen sowie die in der Klinik erhobenen Daten werden selbstverständlich streng vertraulich behandelt und unterliegen der ärztlichen Schweigepflicht und dem Datenschutz..Auch wenn Sie jetzt Ihr Einverständnis für die Untersuchung gegeben haben, können Sie die Teilnahme jederzeit beenden.Die Untersuchungen unterliegend den gleichen versicherungsrechtlichen Bedingungen wie Ihre sonstigen stationären bzw. ambulanten Kontrollen.
Für weitere Fragen stehen wir Ihnen, die für die Studie verantwortlichen Mitarbeiter der Klinik, gerne jederzeit zur Verfügung.

Einverständniserklärung zur Teilnahme am Projekt Lebensqualität und Spätfolgen bei Kindern und Jugendlichen, die nach HIT-LGG behandelt werden

Hiermit erklären wir uns bereit, dass unser Sohn / unsere Tochter

____

an dem Projekt „Lebensqualität und Spätfolgen bei Kindern und Jugendlichen mit Behandlung nach HIT-LGG“ teilnimmt.

Wir wissen, dass wir jederzeit das Recht besitzen, unser hiermit gegebenes Einverständnis zurückzuziehen.

Über das Projekt sind wir umfassend unterrichtet worden.

Die im Rahmen dieser Untersuchungsreihe erhobenen Daten und Ergebnisse dürfen an die Projektleitung zur dortigen Speicherung und wissenschaftlichen Auswertung übermittelt werden. Die erhobenen Daten dienen ausschließlich der Identifikation im Rahmen der Studie und werden darüberhinaus nicht weitergegeben werden!

Die Erhebung wie auch die Auswertung erfolgen unter voller Wahrung der ärztlichen Schweigepflicht!

	______________________ Ort
______________________ Patient/in: Name, Vorname	______________________ Unterschrift Datum
______________________ Sorgeberechtigte/Mutter: Name, Vorname	______________________ Unterschrift Datum
______________________ Sorgeberechtigter/Vater: Name, Vorname	______________________ Unterschrift Datum
______________________ Aufklärende/r Ärztin/Arzt: Name, Vorname	______________________ Unterschrift Datum
______________________ Zeugin / Zeuge: Name, Vorname	______________________ Unterschrift Datum

22.14.3. Wissenschaftliche Begleitprojekte: SIOP-LGG 2004

Behandlungsassoziierte Spätfolgen nach Strahlentherapie maligner Erkrankungen im Kindes- und Jugendalter

Hintergrund:

Die Radiotherapie ist eine wesentliche Therapiemodalität in der Behandlung von Tumoren im Kindes- und Jugendalter, speziell auch bei ZNS-Tumoren. Wie bei jedem therapeutischen Verfahren wird beim Einsatz der Strahlentherapie eine Abwägung zwischen zu erwartendem Nutzen und in Kauf zu nehmenden Nebenwirkungen getroffen. Insofern kommt neben der Erfassung der lokalen Kontrollraten der Erfassung der radiogen induzierten Nebenwirkungen, vor allem der Spätnebenwirkungen, eine wesentliche Rolle zu. In der Arbeitsgemeinschaft pädiatrische Radioonkologie (APRO) wurde das nachfolgende Konzept zur Erfassung radiogener Nebenwirkungen im Kindes- und Jugendalter studienübergreifend erarbeitet. Die zentrale Erfassung der Dokumentation wird im „Register für radiogene Spätnebenwirkungen bei Kindern und Jugendlichen“ (RISK) in Münster durchgeführt (Adresse s.u.).

Konzept zur Erfassung radiogener Spätfolgen:

Bei der Durchführung der Radiotherapie wird vom Radioonkologen eine Dokumentation der Technik der Strahlentherapie sowie der Bestrahlungsdosen an Risikoorganen durchgeführt und an das zentrale Register eingesendet (siehe Tabelle).

2 Monate nach Abschluss der Radiotherapie sowie in der Folge in jährlichem Abstand erfolgt die Wiedervorstellung beim behandelnden Strahlentherapeuten zur Erfassung radiogener Nebenwirkungen, die nach dem Strahlentherapie-spezifischen RTOG/EORTC-Score klassifiziert werden (siehe Tabelle). Die Dokumentation wird ebenfalls an das zentrale Register eingesendet.

Dieses Vorgehen erlaubt die Korrelation von Bestrahlungsdosen an Risikoorganen mit der Inzidenz von Strahlentherapie-induzierten Spätfolgen. Die tumorbezogene Nachsorge bleibt weiterhin ausschließlich in der Hand des betreuenden Pädiaters.

ZEITPUNKT	Dokumentationsbogen	Besonderheiten
RADIOTHERAPIE	**Basisdaten** (Dokumentationsbögen s.u.):<br><br>Angaben zur Technik der Strahlentherapie	
	**Organdosen** (Dokumentationsbögen s.u.)<br><br>Angaben zu Dosisbelastungen an Risikoorganen	1. Dosisvolumenhistogramm für sensible ZNS-Strukturen (Hirnstamm, Chiasma u.a.)<br>2. Dosimetrie Schilddrüse (3 Messpunkte)
2 Monate nach Abschluss der Radiotherapie: Nachsorge	Dokumentation der während oder nach Radiatio maximal aufgetretenen **akuten Nebenwirkungen nach RTOG / EORTC** (Dokumentationsbögen s.u.)	ausschließliche klinische Untersuchung
1.-10. Jahr nach Radiatio: Nachsorge 1x / Jahr	**chronische Nebenwirkungen nach RTOG/EORTC** (Dokumentationsbögen s.u.)	ausschließliche klinische Untersuchung, notwendige Laborwerte bei Pädiatern erfragbar

Dokumentationsbögen unter:

http://medweb.uni-muenster.de/institute/radonk/radtox.htm oder im Register

Dokumentationsunterlagen bitte einsenden an das:

„Register für radiogene Spätnebenwirkungen bei Kindern und Jugendlichen" (RISK)
Klinik für Strahlentherapie –Radioonkologie- des Universitätsklinikum Münster
Albert-Schweitzer-Str. 33
48129 Münster

Tel: 0251/8347384
Fax: 0251/8347355
E-Mail: radtox@uni-muenster.de
(Ansprechpartner Prof. Dr. N. Willich, PD Dr. A. Schuck)

422.15. Liste der (bisher) teilnehmenden Kliniken SIOP-LGG 2004

**Deutschland:**	
Aachen, Uni-Kinderklinik (041)	Homburg/Saar, Uni-Kinderklinik (017)
Augsburg, Klinikum, I. Kinderklinik (001)	Jena, Uni-Kinderklinik (118)
Bayreuth, Städt. Krankenanstalten (095)	Karlsruhe, Städt. Kinderklinik (011)
Berlin, Charite Campus Virchow-Klinikum (016)	Kassel, Städt. Kinderklinik (023
Berlin, Kinderklinik Buch (110)	Kassel, Kinderkrankenhaus (056)
Berlin, Uni-Klinik Benjamin-Franklin (200)	Kiel: Uni-Kinderklinik (026)
Bielefeld-Bethel (033)	Neurochirurgie (142)
Bochum, Uni-Kinderklinik St.-Josef-Spital (200)	Koblenz, Städt. Kinderklinik (021)
Böblingen, Kreis-KH (200)	Köln, Uni-Kinderklinik (058)
Bonn, Uni-Kinderklinik (085)	Krefeld, Städt. Kinderklinik (013)
Braunschweig, Städt. Kinder-KH (042)	Leipzig, Uni-Kinderklinik (119)
Bremen, Prof. Hess-Kinderklinik (004)	Lübeck, Uni-Kinderklinik (031)
Chemnitz, Klinikum Chemnitz (111)	Lüdenscheid, Kreis-KH (148)
Cottbus, Carl-Thiem-Klinikum (112)	Ludwigsburg, Klinikum, Kinderklinik (200)
Datteln, Vestische Kinderklinik (024)	Magdeburg, Uni-Kinderklinik (120)
Dessau, Städt. Klinikum/Kinderklinik (200)	Mainz, Uni-Kinderklinik (002)
Dortmund, Städt. Kliniken (076)	Mannheim, Uni-Kinderklinik (028)
Dresden, Uni-Kinderklinik (113)	Marburg, Uni-Kinderklinik (062)
Dresden–Neustadt, Städt. KH (114)	Minden, Kinderklinik (132)
Duisburg: Städt. Kinderklinik (060)	München, Dr. von Hauner'sches (015)
Neurochirurgie (200)	München, Neurochirurgie Großhadern (200)
Düsseldorf, Uni-Kinderklinik (040)	Münster, Uni-Kinderklinik (034)
Erfurt, Klinikum Erfurt (115)	Nürnberg, Cnopf'sche Kinderklinik (063)
Erlangen, Uni-Kinderklinik (059)	Nürnberg II, Klinikum Süd Kinderklinik (075)
Essen, Uni-Kinderklinik (012)	Oldenburg, Städt. Kliniken (053)
Frankfurt/Oder, Klinikum (200)	Passau, Kinderklinik Dritter Orden (200)
Freiburg i. Br., Uni-Kinderklinik (019)	Ravensburg, Oberschwabenklinik (200)
Gießen, Uni-Kinderklinik (008)	Regensburg, St. Hedwig-Klinik (061)
Göttingen: Uni-Klinik (54)	Sankt Augustin, Johanniter-Kinderklinik (030)
Neuropädiatrie (102)	Schweinfurt, Leopoldina-Krankenhaus (092)
Greifswald, Uni-Kinderklinik (116)	Siegen, DRK-Kinderklinik (078)
Günzburg, (200)	Stuttgart, Olga-Hospital (065)
Halle, Uni-Kinderklinik (117)	Suhl, Zentralklinikum Südthüringen (200)
Hamburg, Uni-Klinik Eppendorf (052)	Trier II, Mutterhaus der Borromaeerinnen (086)
Hamburg, Klinikum Nord (200)	Tübingen: Uni-Kinderklinik (032)
Hamm, evang. KH (77)	Radioonkologie (832)
Hanau, Klinikum Stadt Hanau (200)	Ulm: Uni-Kinderklinik (071)
Hannover, Kinderklinik der MHH (045)	Kinderneurologie (200)
Hannover, Kinder-KH a. d. Bult (069)	Unna, Kinderneurologie (138)
Heide, Westküstenklinikum (200)	Vechta, St. Marienhospital (003)
Heidelberg, Uni-Kinderklinik (005)	Wolfsburg, Stadt-KH (105)
Heidelberg, DKFZ (200)	Würzburg, Uni-Kinderklinik (018)
Herdecke, Gemeinschaft-KH (081)	
**Österreich:**	**Schweiz:**
Feldkirch (724)	Aarau (711)
Graz (721)	Basel (713)
Innsbruck (722)	Lausanne (710)
Klagenfurt (726)	Luzern (715)
Linz, Barmherz. Schwestern (720)	St. Gallen (714)
Linz, LKK (725)	Zürich (712)
Salzburg (723)	**Belgien:**
Wien (500)	Leuven (700)
