# Supplementary material for: A European randomised controlled trial of the addition of etoposide to standard vincristine and carboplatin induction as part of an 18-month treatment programme for childhood (≤16 years) low grade glioma – A final report
Source: Eur J Cancer. 2017 Aug;81:206–25. doi: 10.1016/j.ejca.2017.04.019 (PMC5517338; doi:10.1016/j.ejca.2017.04.019)
Supplement: Supplementary file 2 [file mmc2.zip › SIOP-LGG 2004 - Toxicity Amendment.pdf]

Cooperative Multicenter Low Grade Glioma Trial
SIOP-LGG 2004
Toxicity Amendment

When the current SIOP-LGG 2004 trial was conceived, no trustworthy data concerning toxicity of this treatment existed. Therefore the committee assumed that the probability for a toxic event of 10 % would be acceptable and that the trial should be stopped, if the probability for a toxic event exceeded 25 %.
At that time the choice of "relevant toxicities" was adapted from other clinical trials and non-haematologic toxicities of the kidneys, the liver, the inner ear and of the central and peripheral nervous system and infections WHO and/or CTC °III and IV were included.

With respect to neurotoxicity it was not considered, however, that in contrast to other clinical trials the majority of low grade glioma patients already suffered from pre-existing neurological impairment rendering them susceptible to additional neurologic symptoms and making it difficult to separate the effect of primary disease and additional toxicity.

Amendment/Clarification for the documentation of neurotoxicity:

- **Documentation of neurotoxicity has therefore to be restricted to those symptoms that can be related definitely to therapy and not to prior tumour or tumour progression.**

- **Those neurotoxicities that have already been reported and included in DMSC reports shall be reinvestigated and regraded for their relation to therapy and be maintained, if therapy related only.**

Additionally, some of the listed toxicities have no known relation to the drugs under investigation in the randomized trial. Their appearance may have a multitude of aetiologies and is not relevant for the conduct of the trial. This applies to liver toxicity as measured by a rise in transaminases, Grade III CTC. The protocol section on drug modification subsequent to toxicity does not consider the elevation of transaminases.

Amendment/Clarification for the documentation of liver toxicity:

- **The results of liver function tests should be documented, considering those elevations of liver serum enzymes which persist prior to continuation of therapy.**
- **Grade III CTC results will be recorded in the data bank, but not be considered as relevant for the conduct of the trial.**
- **Grade IV CTC results will remain part of the stopping rule.**

This document was discussed and accepted from the representatives of all participating national groups at the occasion of the SIOP-BTC plenary meeting in Genoa, May 14th, 2009.

For the international trial committee

Astrid K. Gnekow David Walker Jacques Grill Giorgio Perilongo
