# Supplementary material for: A European randomised controlled trial of the addition of etoposide to standard vincristine and carboplatin induction as part of an 18-month treatment programme for childhood (≤16 years) low grade glioma – A final report
Source: Eur J Cancer. 2017 Aug;81:206–25. doi: 10.1016/j.ejca.2017.04.019 (PMC5517338; doi:10.1016/j.ejca.2017.04.019)
Supplement: Supplementary file 2 [file mmc2.zip › SIOP-LGG 2004 Age eligibility amendment.pdf]

SIOP-LGG 2004
Cooperative multicenter Study for Children and Adolescents With Low Grade Glioma

International Consortium on Low Grade Glioma - ICLGG
of the International Society of Pediatric Oncology - SIOP

Age eligibility

Background:

When the SIOP-LGG 2004 protocol was conceived, the cooperating groups agreed upon a number of eligibility criteria. A general consensus was made to restrict the study population to children and adolescents under the age of 16. At that time the age limit was chosen on the grounds of that paediatric wards only accepted children and adolescents up to 15-16 years and accordingly childhood cancer registries only supplied data on the age group up to 15 years. Meanwhile several countries or individual hospitals give care for older adolescents in their paediatric or specialized adolescent units and in some countries treatment of adolescents with cancer up to 18 years in paediatric cancer units is even mandatory (e.g. Germany).
Thus, according to national or local policies, children being 16 years or older may be registered and enrolled for the non-surgical treatment arms. They will not be part of the study population, however, since their number will not be representative for the complete age group. Additionally, it is to be expected, that low grade brain tumours in this older age group will represent a biologically “transitional” group towards the adult type tumours and thus the paediatric approach may not be suitable. Registering and evaluating the results from this group will enable us to learn about these biological features and find and improve adequate treatment strategies.

Amendment

- The eligibility criteria shall be modified in that according to national practice adolescents of age 16 or older can be registered into the SIOP-LGG 2004 trial.
- Since there is not enough information to anticipate the biologic behaviour of their types of low grade glioma, these patients may follow the basic LGG treatment strategy, but remain not-eligible for the randomized part of the trial. This amendment therefore does not touch the study questions.

Procedure

- This amendment will be presented at the SIOP-BTC annual meeting to the LGG working group/international committee meeting.
- Upon acceptance this amendment will be distributed to all national trial coordinators to be incorporated into the respective national protocols.

Accepted: Lyon, April 27th, 2007	Distributed:	Signature:
