# Supplementary material for: A European randomised controlled trial of the addition of etoposide to standard vincristine and carboplatin induction as part of an 18-month treatment programme for childhood (≤16 years) low grade glioma – A final report
Source: Eur J Cancer. 2017 Aug;81:206–25. doi: 10.1016/j.ejca.2017.04.019 (PMC5517338; doi:10.1016/j.ejca.2017.04.019)
Supplement: Supplementary file 2 [file mmc2.zip › SIOP-LGG 2004 Biostatistical amendment.pdf]

SIOP-LGG 2004
Cooperative multicenter Study for Children and Adolescents With Low Grade Glioma

International Consortium on Low Grade Glioma - ICLGG
of the International Society of Paediatric Oncology - SIOP

Biostatistical Amendment: Trial Prolongation

Rationale to Prolong the SIOP-LGG 2004 Trial

The aim of the SIOP-LGG 2004 sub trial for children, who are not affected by Neurofibromatosis (type NF I), with low grade glioma of all sites necessitating chemotherapy as non-surgical therapy is to compare standard induction therapy with Vincristin and Carboplatin with the intensified induction therapy with Vincristin, Carboplatin and Etoposide.

This therapy optimization trial is multinational, multicentre, non-blinded, randomized and prospective. By means of this trial the following main question shall be answered: Does intensified induction therapy with additional Etoposide lead to a different progression free survival PFS_R measured form the time of randomization than the standard induction therapy?

Three analyses – two interim analyses and the final analysis – were planned to answer the main question. **The three analyses are scheduled after 66, 132, and 198 events pooled over both therapy arms.** The criteria for stopping the trial after an interim analysis are given by a 3-step group sequential plan according to Pampallona & Tsiatis with the possibility to stop the trial in favor of the alternative and the null hypothesis. The bounds of the 3-step group sequential design result from α=5%, power=90%, and an α-spending approach according to O'Brien & Fleming.

In the study protocol the following assumptions were originally stated: accrual rate of 60 children per year, 3-year PFS_R of 50% having standard induction therapy and 65% having the intensified induction, 5-year drop-out rate of 10% and exponentially distributed PFS_R and independent exponentially distributed drop-out-times. As a consequence, the accrual period of the trial were intended to be 6 years followed by an observation period of 2 years.

The accrual rate of the hitherto study meets the expectation. **Fortunately, the pooled PFS_R, observed so far, is higher than originally planned.** Consequently, **the planned accrual period and the observation time are too short** to observe 198 events pooled over both therapy arms. **Therefore, the observed study success leads to prolonged study duration.**

In comparison to the historical data, the pooled PFS of the actual study is superior to the historical data. A prolongation of the trial could prove that this **effect is constant over the time.**

Moreover, the **protocol of the subsequent trial will not be available** until the originally planned end of the recruitment of the SIOP-LGG 2004 trial. Terminating the trial before the start of a new study could result in a **loss of recruiting centres** and organisational structure established so far. Particularly, the **quality of a controlled clinical trial is superior** to the quality in an observational study.

Certainly, a trial can be prolonged only if patients are not put at risk i.e. none of the therapies has proven to be better. The first interim analysis of the SIOP-LGG 2004 **did not show a significant superiority** of the standard or intensified induction therapy. In addition, the **rate of toxicity is comparable** between both therapy arms. **A prolongation of the trial allows getting better estimators of the PFS and the rate of toxicity.**

Altogether, we recommend prolonging the accrual period and/or observation time of the SIOP-LGG trial. This allows observing the scheduled number of events in the final analysis and results in **higher study power, better estimators for the survival rates and more information about adverse effects**.

SIOP-LGG 2004 Protocol

Version April 2004 (corr. July´04, Jan´06), chapter 17.1.1., page 165-6:
Interim analyses and final analysis, stopping rule
Analyses will be performed after 1/3, 2/3 and all expected events occurred, unless the trial was stopped before. Both induction therapy arms are added up to evaluate the number of occurred events with respect to the expected number of events.
With an accrual period of 6 years, a follow-up period of 2 years, an accrual rate of 60 children per year, a 3-year PFS_R of 50% having standard induction therapy and 65 % having the intensified induction, a 5-year drop-out rate of 10% and the assumption of exponential distributed PFS_R and independent exponential distributed drop-out-times ($\lambda = 0.0211$), a total number of 198 events is expected. Therefore the first interim analysis is scheduled to take place after 66 events and second after 132 events.
The trial will be terminated after an interim analysis, if the main question can already be answered at this interim analysis or the chance to answer the main question is low while continuing the trial.
The criteria for stopping the trial after an interim analysis are given by a 3-step group sequential plan according to Pampallona & Tsiatis with the possibility to stop the trial in favour
for the alternative and the null hypothesis [Jennison 2000]. The bounds of the 3-step group sequential design result from α=5%, power=90%, hazard ratio =1,609, progression free survival rate after 3 years of 50% and 65% for the two groups and an α-spending approach according to O'Brien & Fleming [1979] ($\Delta = 0$).

Amended, new version:
Interim analyses and final analysis, stopping rule
Three analyses will be performed to answer the main question, unless the trial was stopped before. The trial will be terminated after an interim analysis, if the main question can already be answered at this interim analysis or the chance to answer the main question is low while continuing the trial.
The criteria for stopping the trial after an interim analysis are given by the inverse normal method corresponding to a 3-step group sequential plan according to Pampallona & Tsiatis with the possibility to stop the trial in favour of the alternative and the null hypothesis [Jennison 2000, Wassmer 2006]. The bounds of the 3-step group sequential design result from α=5%, power=90%, hazard ratio =1,609, progression free survival rate after 3 years of 50% and 65% for the two groups, an alpha-spending approach according to O'Brien & Fleming [1979] ($\Delta = 0$), and equally weighted independent increments of the inverse normal test statistic.

Both induction therapy arms are added up to evaluate the number of occurred events with respect to the expected number of events. With an accrual period of 6 years, a follow-up period of 2 years, an accrual rate of 60 children per year, a 3-year PFS_R of 50% having standard induction therapy and 65 % having the intensified induction, a 5-year drop-out rate of 10% and the assumption of exponential distributed PFS_R and independent exponentially distributed drop-out-times ($\lambda = 0.0211$), a total number of 198 events is expected. Therefore the first interim analysis is intended to take place after 66 events.
After each interim analysis a data dependent sample size calculation may be performed. Then, the accrual period, the observation time and the schedule of the second interim and final analysis (required number of events) can be adapted.

Wassmer G. Planning and Analyzing Adaptive Group Sequential Survival Trials. Biometrical Journal 2006; 48; 714-729.

Accepted: Paris, December 6th, 2009	Distributed:	Signature:
