# Supplementary material for: A European randomised controlled trial of the addition of etoposide to standard vincristine and carboplatin induction as part of an 18-month treatment programme for childhood (≤16 years) low grade glioma – A final report
Source: Eur J Cancer. 2017 Aug;81:206–25. doi: 10.1016/j.ejca.2017.04.019 (PMC5517338; doi:10.1016/j.ejca.2017.04.019)
Supplement: Supplementary file 2 [file mmc2.zip › SIOP-LGG 2004 SAE-SUSAR amendment.pdf]

SIOP-LGG 2004
Cooperative multicenter Study for Children and Adolescents
With Low Grade Glioma
International Consortium on Low Grade Glioma - ICLGG
of the International Society of Pediatric Oncology - SIOP

Reporting SAE and SUSAR within the SIOP-LGG 2004 study

With the implementation of the directive 2001/20/EC and the detailed Guidance ENTR/CT 3 into national legislation within the European Union more detailed requirements were made for the reporting of serious adverse events (SAE) and suspected unexpected serious adverse reactions (SUSAR). A synchronous initiation of the SIOP-LGG 2004 trial was not possible throughout the participating countries and for other European countries there are country specific regulations.
Reporting of SAEs is within section 16.4 (page 159) of the protocol. This amendment will give additional and more detailed information to SAE/SUSAR reporting within the SIOP-LGG 2004 study.

Procedures of SAE / SUSAR report

The investigator has the responsibility to report all serious events (SAE) within 24 hours or one business day to the sponsor except for those that the protocol or investigator's brochures identifies as not requiring immediate reporting. This initial report has to be followed by detailed, written reports.

Single Cases of Serious, Unexpected AEs

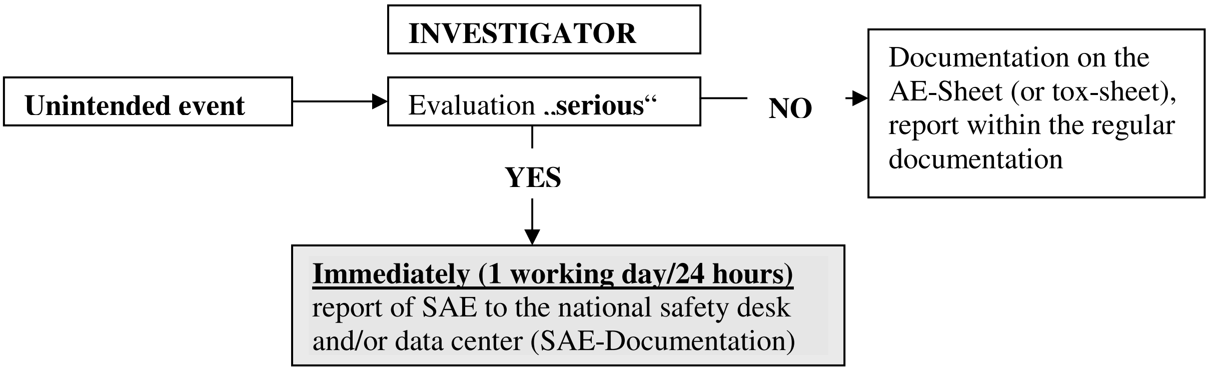

- The national safety desk/data center (according to how nationally regulated) will record and evaluate the reported SAE with respect to its seriousness, relatedness, expectedness or other safety issues.
- The investigator has to supply the national safety desk/data center or the Ethics Committee with any additional requested information.
- Regulatory agencies should be notified as soon as possible
    - For fatal of life-threatening unexpected adverse drug reactions: no later than **7 calendar days** after first knowledge, followed by as complete report as possible within **8 additional calendar days.**
    - All other serious unexpected adverse drug reactions: no later than **15 calendar days** after first knowledge.

- **Expedited reporting:** The national data center provides new, important information on serious reactions periodically to the local investigator (e.g. newsletter).

Definition of Serious Adverse Event/Reaction: Any untoward medical occurrence in a study patient treated with chemotherapy according to SIOP-LGG 2004 protocol that is both "unexpected" and "serious":

- results in death,
- is life-threatening,

- requires inpatient hospitalization or prolongation of existing hospitalization,
- results in persistent or significant disability/incapacity, or
- is a congenital anomaly/birth defect
- any other medically important condition such as abnormal biological or vital signs´ and secondary malignancies (cancer) – For exceptions see below

Time frame: after the first administration of the study drug until 12 months after termination of chemotherapy.

Definitions and Exceptions for SIOP-LGG 2004:

• **All deaths** including death due to disease progression during protocol treatment and for 30 days after the last protocol treatment will be reported as an SAE.

Exception: Death due to progression of disease will not constitute a SAE if it occurs later than 30 days after the last protocol treatment.

• The term "**life-threatening**" refers to an event where the patient is at IMMEDIATE risk of death at the time of the event (e.g. requires IMMEDIATE intensive care treatment). It does not refer to an event which hypothetically might cause death if it were more severe

• **Hospitalization** is defined as at least one overnight admission

Exception:

- Hospitalization for chemotherapy is **not** reported as an SAE. In addition expected side effects of chemotherapy, which are listed in the product information, will not be reported on an SAE form for the purposes of this clinical trial unless in the opinion of the investigator they unexpectedly prolonged the hospitalization or required intensive care therapy.
- Hospitalization for procedures required by the protocol e.g. biopsy or surgery are **not** considered serious adverse events until one of the above criteria are met.
- Hospitalization due to signs and symptoms associated with disease progression are **not** considered an SAE unless outcome leads to DEATH
- Elective hospitalization for a pre-existing condition that has not worsened does **not** constitute a SAE.

• **Disability** is defined as a substantial disruption in a person's ability to conduct normal life functions (e.g. blindness, deafness).

Exception: Disability resulting from tumor surgery does not constitute an SAE within the context of this trial.

• **Other medically important conditions** are important medical events that in the opinion of the investigator may not be immediately life-threatening or result in death or hospitalization, but may jeopardize the patient or may require intervention to prevent one of the other outcomes listed in the definition above. Examples of such events are intensive treatment in an emergency room or at home for allergic bronchospasm or convulsions. Secondary malignancies are also considered to be medically important and are reportable on an SAE at any time. Abnormal biological or vital signs commonly occur under chemotherapy and will only be reported as serious when considered CLINICALLY RELEVANT BY THE INVESTIGATOR (unexpected) e.g. severe nephrotoxicity (CTCAE Grade 4) or severe cardiac toxicity (CTCAE Grade 4). Expected serious adverse reactions (SAR) are hematological toxicity.

SUSARS within SIOP-LGG 2004

Definition: An adverse reaction, the nature or severity of which is not consistent with the applicable product information. Expected effects and expected side effects of the drugs of the SIOP-LGG 2004 trial are listed under **section 14** in the study protocol. WHO grade IV toxicities other than hematologic are regarded as serious and have to be reported to the data center immediately. These toxicities are defined according to CTC 2.0 and are listed below.
Note: with regard to nervous system toxicities signs and symptoms of the underlying or progressive tumor have to be distinguished from serious drug reactions!

- Carboplatin: life-threatening allergic shock
- Drug overdose
- Death under treatment
- Secondary malignant neoplasmen

Categories regarded as a serious drug reaction (SDR):

**Nervous system**	
**Neuropathy-sensory**	permanent sensory loss that interferes with function
**Neuropathy-motor**	Paralysis
**Neuropathy-cranial**	life-threatening, disabling
**Seizures**	seizure(s) of any type which are prolonged, repetitive of difficult to control (e.g., status epilepticus, intractable epilepsy)
**Abdominal pain or cramping**	disabling

**Renal**	
**hematuria**	open surgery or necrosis/deep bladder ulceration
**creatinine**	> 6,0 (x ULN)
**proteinuria**	nephrotic syndrome
**creatinine clearance**	≤19 (ml/min + 1,73 m²)

**Hepatic**	
**hepatic bilirubin**	> 10,0 (x ULN)
**SGOT/SGPT**	> 20,0 (x ULN)

**Cardiac**	cardiac severe or refractory CHF or requiring intubation

**Skin**	Skin necrosis or ulceration of full thickness dermis may include bleeding not induced by minor trauma or abrasion
